# Supplementary material for: Manganese-mediated reductive functionalization of activated aliphatic acids and primary amines
Source: Nat Commun. 2020 Oct 7;11:5036. doi: 10.1038/s41467-020-18834-6 (PMC7542462; doi:10.1038/s41467-020-18834-6)
Supplement: Supplementary file 1 — Supplementary Information [file 41467_2020_18834_MOESM1_ESM.pdf]

Supporting Information

Manganese-Mediated Reductive Functionalization of Activated Aliphatic Acids and Primary Amines

Li et al

Supplementary Methods

General Information

Unless otherwise noted, all reactions were carried out under an atmosphere of nitrogen in flame-dried glassware. If reaction was not carried out at room temperature, reaction temperatures are reported as the temperature of the bath surrounding the vessel unless otherwise stated. The dry solvents used were purified by distillation over the drying agents indicated in parentheses and were transferred under nitrogen. Commercially available chemicals were obtained from commercial suppliers and used without further purification unless otherwise stated.

Proton NMR (^{1}H) were recorded at 400/500 MHz, and Carbon NMR (^{13}C) at 101/126 MHz NMR spectrometer unless otherwise stated. The following abbreviations are used for the multiplicities: s: singlet, d: doublet, t: triplet, q: quartet, m: multiplet, br s: broad singlet for proton spectra. Coupling constants (*J*) are reported in Hertz (Hz).

High-resolution mass spectra (HRMS) were recorded on a BRUKER VPEXII spectrometer with EI and ESI mode unless otherwise stated.

Analytical thin layer chromatography was performed on Polygram SIL G/UV254 plates. Visualization was accomplished with short wave UV light, or $KMnO_4$ staining solutions followed by heating. Flash column chromatography was performed using silica gel (200-300 mesh or 600-800 mesh) with solvents distilled prior to use.

Synthesis of Starting Materials

Synthesis of NHPI (*N*-Hydroxyphthalimide) Esters (General procedure 1)

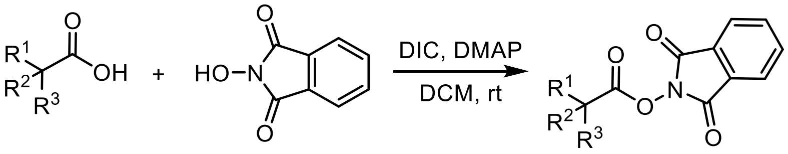

NHPI esters were prepared according to the literature reported procedures: A round-bottomed flask was charged with carboxylic acid (1.0 equiv), *N*-hydroxyphthalimide (1.1 equiv) and dimethylaminopyridine (DMAP) (0.1 equiv). Dichloromethane was then added (0.1 M), and the mixture was stirred vigorously for 5 min. After that, *N*,*N'*-diisopropylcarbodiimide (DIC) (1.2 equiv) was added and the mixture was allowed to stir at room temperature until the acid was consumed (determined by TLC, typically 0.5-24 h). The mixture was cooled down in freezer (-20 ^{o}C) and then filtrated under reduced pressure. The filtrate was concentrated and subjected to flash column chromatography to afford the desired NHPI ester.

We and others have previously reported the synthesis of redox-active esters shown below. **SI-1-1**[1], **SI-1-3**[1], **SI-1-4**[1], **SI-1-7**[1], **SI-1-8**[1], **SI-1-10**[1], **SI-1-11**[2], **SI-1-12**[2], **SI-1-13**[3], **SI-1-14**[4], **SI-1-15**[1], **SI-1-16**[5], **SI-1-21**[1], **SI-1-22**[6], **SI-1-23**[1], **SI-1-25**[1], **SI-1-26**[6], **SI-1-28**[3], **SI-1-29**[1], **SI-1-30**[6], **SI-1-31**[3], **SI-1-33**[3], **SI-1-34**[3], **SI-1-36**[1], **SI-1-37**[7], **SI-1-38**, **SI-1-39** were synthesized according to the above general procedure, and all the spectroscopic data matched those reported.

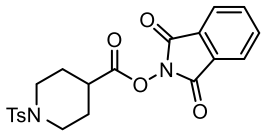
SI-1-1 (1)
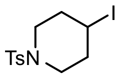
1-I
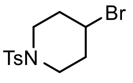
1-Br
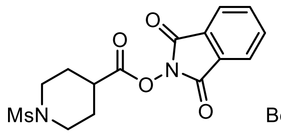
SI-1-2
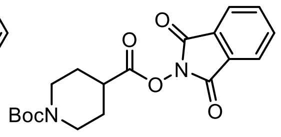
SI-1-3

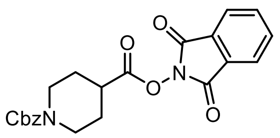
SI-1-4
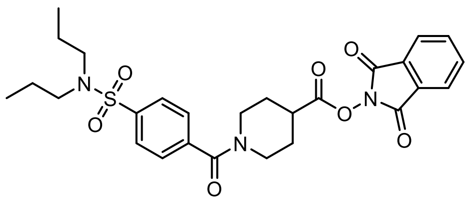
SI-1-5
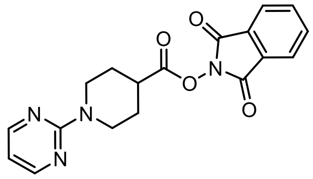
SI-1-6

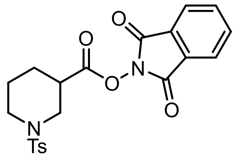
SI-1-7
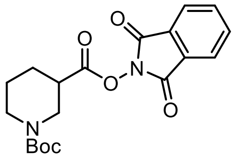
SI-1-8
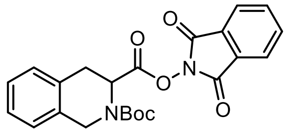
SI-1-9

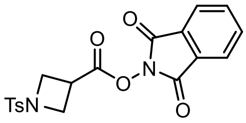
SI-1-10
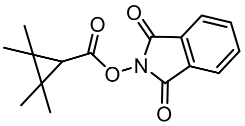
SI-1-11
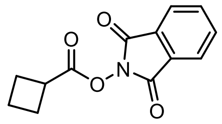
SI-1-12

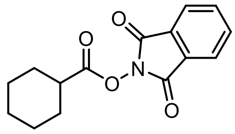
SI-1-13 (125)
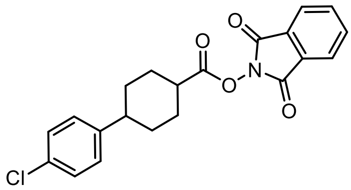
SI-1-14
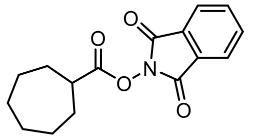
SI-1-15

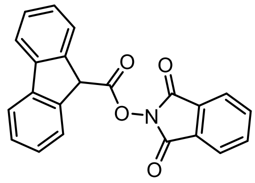
SI-1-16
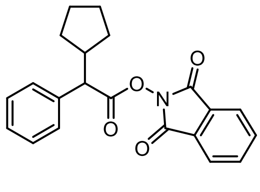
SI-1-17
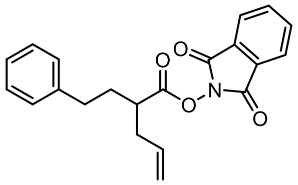
SI-1-18

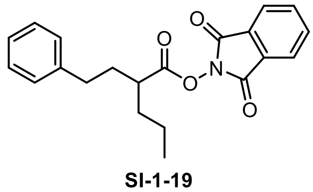
SI-1-19
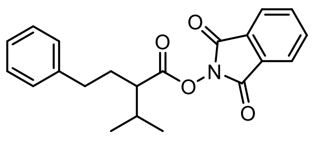
SI-1-20
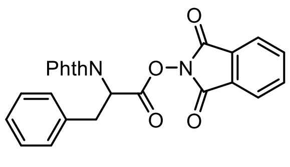
SI-1-21

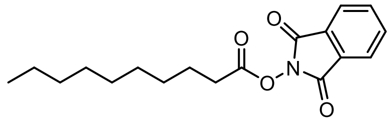
SI-1-22
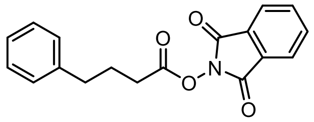
SI-1-23

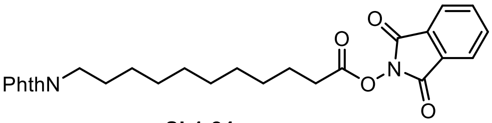

SI-1-24

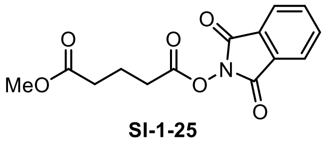

SI-1-25

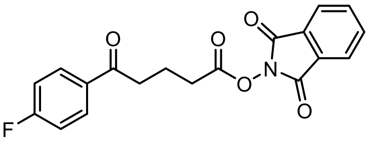

SI-1-26

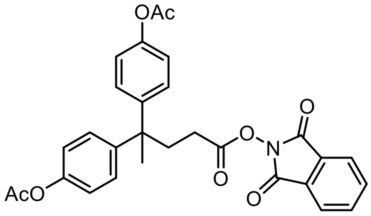

SI-1-27

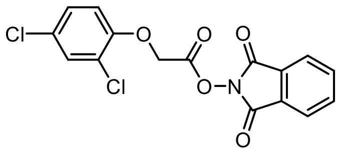

SI-1-28

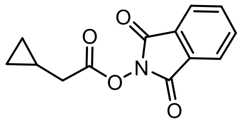

SI-1-29 (130)

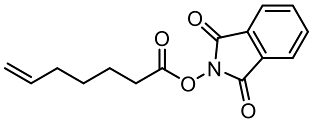

SI-1-30 (134)

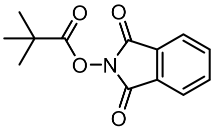

SI-1-31

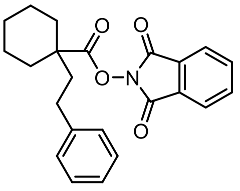

SI-1-32

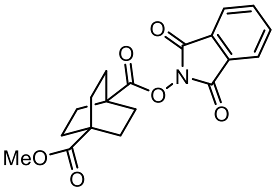

SI-1-33

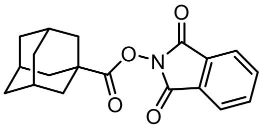

SI-1-34

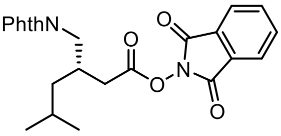

SI-1-35

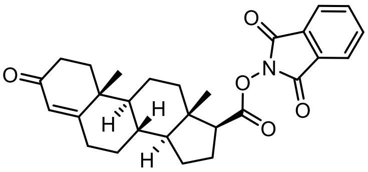

SI-1-36

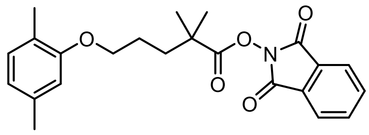

SI-1-37

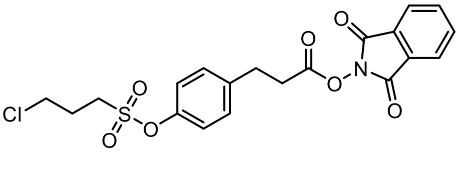

SI-1-38

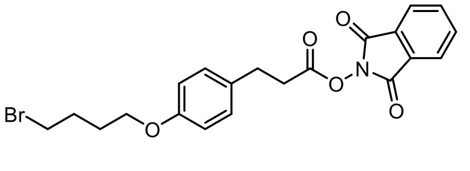

SI-1-39

Synthesis of N-alkylpyridinium salts (General procedure 2)

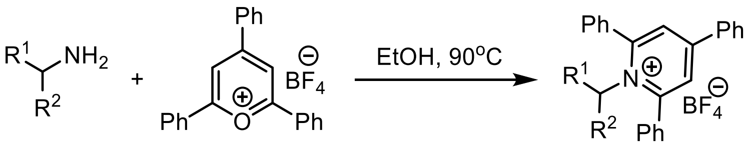

Primary amine (1.2 equiv) was added to a suspension of 2,4,6-triphenylpyrylium tetrafluoroborate (1.0 equiv) in EtOH (1.0 M) in a long Schlenk tube. The mixture was stirred and heated at reflux in an oil bath at 90 °C for 4 h. The mixture was then allowed to cool to room temperature. If product precipitation occurred after cooling to room temperature, the solid was filtered, washed with EtOH and then Et_2O, and dried

under high vacuum. If product precipitation did not occur during reflux, the solution was diluted with Et_2O (2–3 × volume of EtOH used) and vigorously stirred for 1 h to induce trituration. The resulting solid pyridinium salt was filtered and washed with Et_2O. If the pyridinium salt failed to precipitate at this point, it was subjected to flash column chromatography, eluting with acetone/DCM. The corresponding amine hydrochloride salts can also be used by following the modified procedure: Et_3N (1.2 equiv) was added to a mixture of the corresponding alkyl ammonium hydrochloride salt (1.2 equiv) and 2,4,6-triphenylpyrylium tetrafluoroborate (1 equiv) in EtOH (1.0 M). The mixture was stirred and heated at reflux in an oil bath at 90 ^{o}C for 4 h. The mixture was then allowed to cool to room temperature and the EtOH was removed in vacuo. The residue was dissolved in a small amount of DCM and subjected to flash column chromatography, eluting with acetone/DCM.

The pyridinium salts: **SI-2-1[8]**, **SI-2-2[8]**, **SI-2-3[8]**, **SI-2-4[8]**, **SI-2-5[10]**, **SI-2-6[9]**, **SI-2-7[9]**, **SI-2-8[8]**, **SI-2-9[11]**, **SI-2-10[9]**, **SI-2-11[9]** were synthesized according to the above general procedure, and all the spectroscopic data matched those reported.

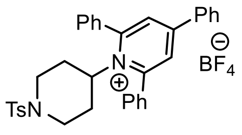
SI-2-1 (4)

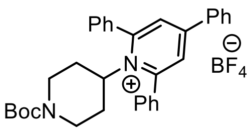
SI-2-2 (122)

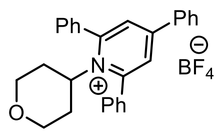
SI-2-3

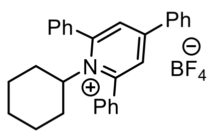
SI-2-4 (128)

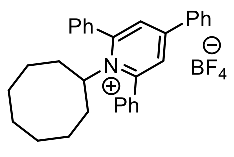
SI-2-5

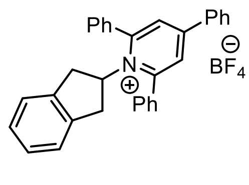
SI-2-6

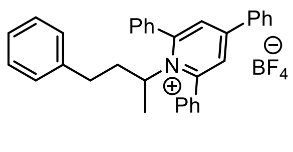
SI-2-7

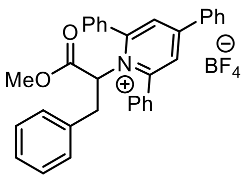
SI-2-8

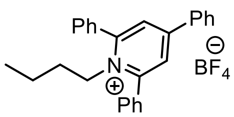
SI-2-9

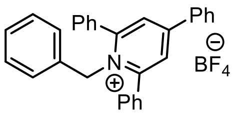
SI-2-10

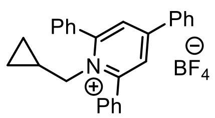
SI-2-11 (138)

Synthesis of disulfide compounds

Disulfides **SI-3-1**, **SI-3-3**, **SI-3-4**, **SI-3-6**, **SI-3-7**, **SI-3-8**, **SI-3-9**, **SI-3-12**, **SI-3-13**, **SI-3-14**, **SI-3-15**, **SI-3-17**, **SI-3-18** were purchased from Sigma Aldrich, Acros, TCI, Alfa Aesar, J&K, or Adamas. Other disulfides including **SI-3-2**[12], **SI-3-5**[12],

SI-3-10[13], **SI-3-11**[12], **SI-3-16**[13] and **SI-3-19**[12] were prepared by the same procedure as described in the literature.

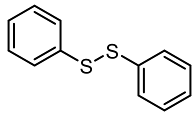

SI-3-1 (2)

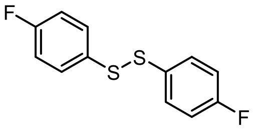

SI-3-2

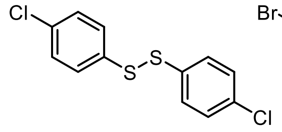

SI-3-3

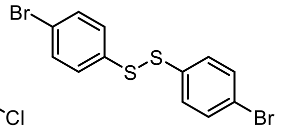

SI-3-4

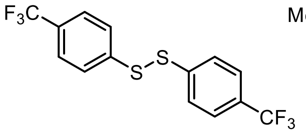

SI-3-5

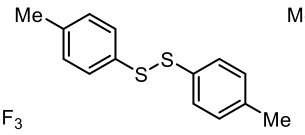

SI-3-6

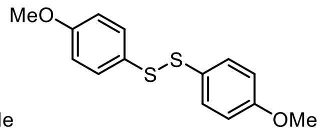

SI-3-7 (132)

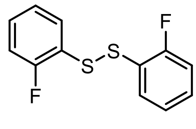

SI-3-8

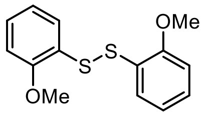

SI-3-9

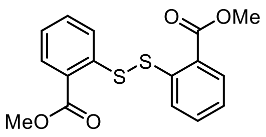

SI-3-10

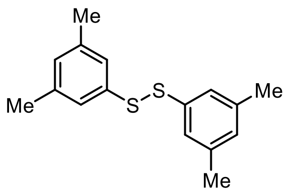

SI-3-11

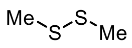

SI-3-12

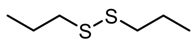

SI-3-13

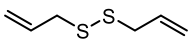

SI-3-14

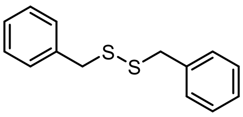

SI-3-15

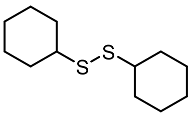

SI-3-16

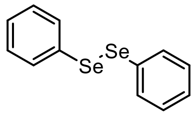

SI-3-17

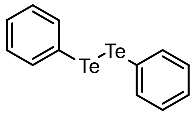

SI-3-18

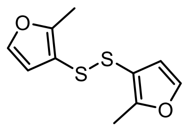

SI-3-19

Synthesis of benzensulfonothioates compounds(General procedure 3)

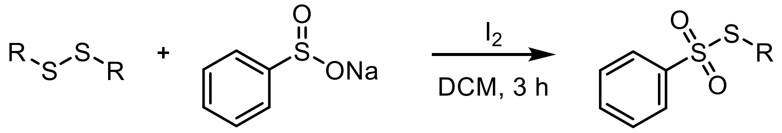

I_2 (2.0 equiv) was added to a mixture of sodium benzenesulfinate (3.2 equiv) and disulfide (1.0 equiv) in CH_2Cl_2. The mixture was allowed to stir at room temperature until the disulfide was consumed (determined by TLC, typically 3 h). Then the reaction was quenched with saturated $Na_2S_2O_3$. The aqueous layer was extracted with DCM for 3 times. The combined organic layer was dried over anhydrous Na_2SO_4 and concentrated and subjected to flash column chromatography to afford the desired substrate.

We and others have previously reported the synthesis of redox-active esters shown

below. **SI-4-1**[14], **SI-4-2**[14], **SI-4-3**[14], **SI-4-4**[14], **SI-4-5**[14], **SI-4-6**[14],**SI-4-7**[15], **SI-4-8**[16], **SI-4-9**[14], **SI-4-11**[15], **SI-4-12**[17], **SI-4-13**[14], **SI-4-14**[14], **SI-4-15**[17], **SI-4-16**[17], **SI-4-17**[18], **SI-4-18**[15], **SI-4-19**[19], were synthesized according to the above general procedure, and all the spectroscopic data matched those reported.

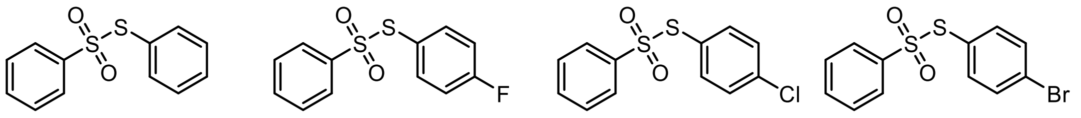

SI-4-1 (5) **SI-4-2** **SI-4-3** **SI-4-4**

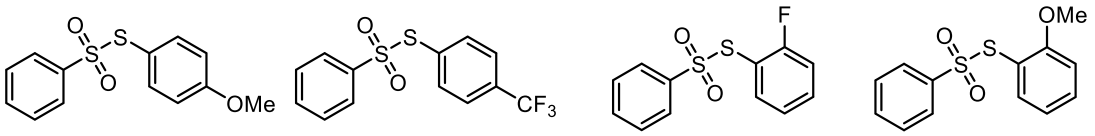

SI-4-5 (138) **SI-4-6** **SI-4-7** **SI-4-8**

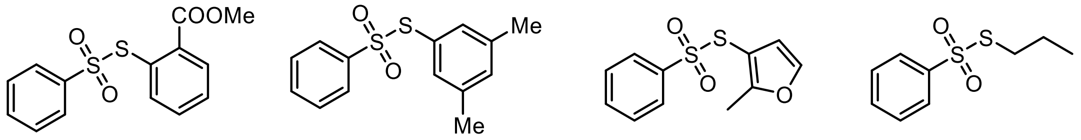

SI-4-9 **SI-4-10** **SI-4-11** **SI-4-12**

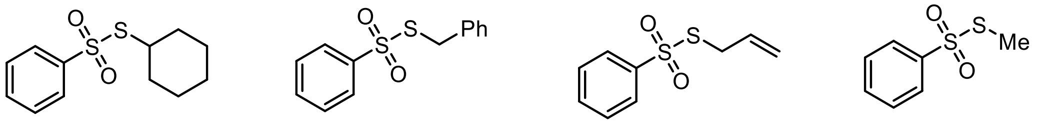

SI-4-13 **SI-4-14** **SI-4-15** **SI-4-16**

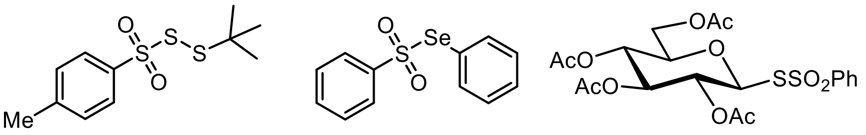

SI-4-17 **SI-4-18** **SI-4-19**

Synthesis of vinyl bromide

The (2-bromovinyl)benzene (**SI-5-1**) was purchased from Energy Chemical. Vinyl bromides **SI-5-2**[20], **SI-5-3**[20] were prepared according to procedures reported by Reisman and coworkers. Vinyl bromides **SI-5-4**[21], **SI-5-5**[21], **SI-5-6**[21] **SI-5-7**[22]were prepared according to a procedure by Alexakis and coworkers.

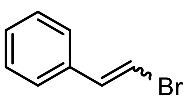

SI-5-1

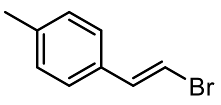

SI-5-2

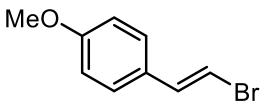

SI-5-3

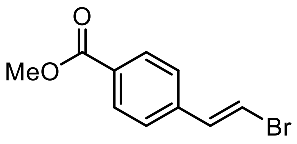

SI-5-4

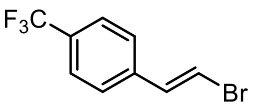

SI-5-5

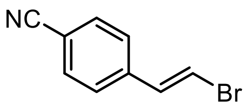

SI-5-6

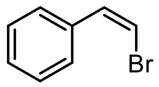

SI-5-7

Synthesis of a-(trifluoromethyl)styrenes (General procedure 4)

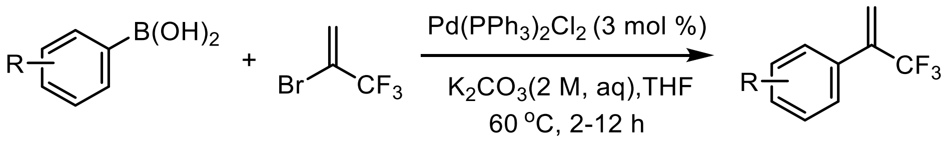

In a Schlenk tube equipped with a stirring bar, arylboronic acids (10 mmol) and $Pd(PPh_3)_2Cl_2$ (0.3 mmol, 210.6 mg) were added. The vessel was evacuated and filled with argon (three cycles), then aqueous K_2CO_3 (2.0 M, 20 mL) and THF (30 mL) were added. After the addition of 2-bromo-3,3,3-trifluoropropene (2.0 equiv, 20 mmol, 2.1 mL), the solution was stirred at 60 °C for 2-12 hours (TLC tracking detection). The mixture was purified by column chromatography to afford the corresponding trifluoromethyl alkenes. **SI-6-1**[23], **SI-6-3**[23], **SI-6-4**[24], **SI-6-5**[23], **SI-6-6**[23],**SI-6-7**[25], **SI-6-8**[26], **SI-6-9**[26], **SI-6-10**[23], **SI-6-11**[25], **SI-6-13**[23], were known compounds, and the data match the reported ones.

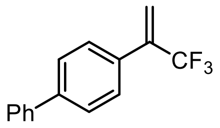

SI-6-1

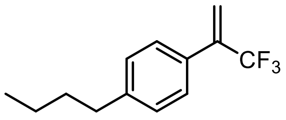

SI-6-2

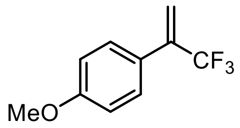

SI-6-3

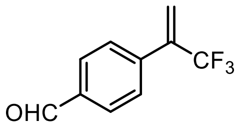

SI-6-4

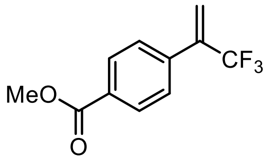

SI-6-5

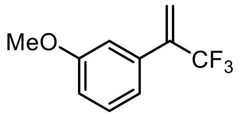

SI-6-6

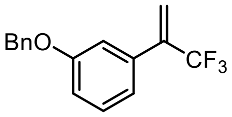

SI-6-7

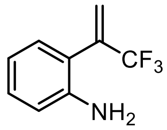

SI-6-8

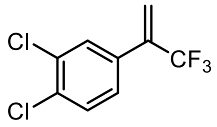

SI-6-9

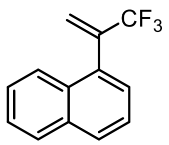

SI-6-10

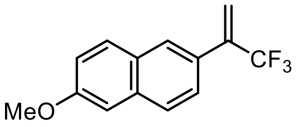

SI-6-11

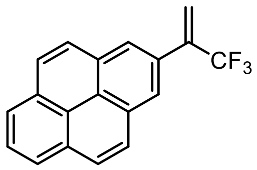

SI-6-12

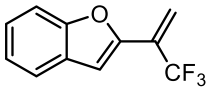

SI-6-13

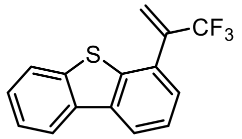

SI-6-14

General Procedure for Decarboxylative and Deaminative Functionalizations

Decarboxylative thiolation (General procedure 5)

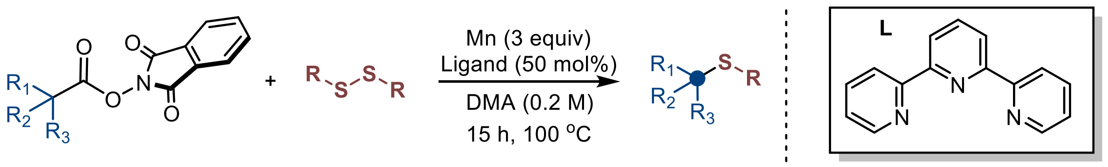

Reactions were set up in a N_2 filled glove box. To a 10 mL reaction tube equipped with a stirring bar, were added NHPI ester (0.3 or 0.4 mmol, 1.5 or 2 equiv), disulfide (0.2 mmol, 1.0 equiv), Mn (0.6 mmol, 3 equiv) and 2,2′:6′,2″-terpyridine (0.1 mmol, 0.5 equiv), DMA (1.0 mL, 0.2 M) under N_2 atmosphere. After that, the resulting mixture was sealed with a screw cap and carried out of the glove box, then the resulting mixture was stirred at 100 ^{o}C for 15 h. Then saturated aqueous NH_4Cl was added to quench the reaction. The aqueous layer was then extracted with diethyl ether, and the combined organic layers were washed with brine, dried over anhydrous

Na_2SO_4, filtered and concentrated. The residue was purified by flash column chromatography on silica gel using Petroleum ether /EtOAc as eluant.

Deaminative thiolation (General procedure 6)

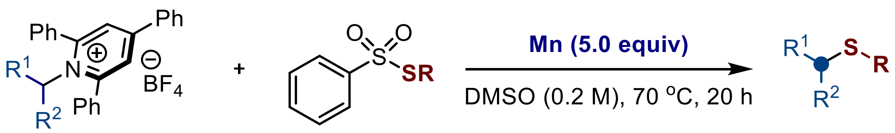

Reactions were set up in a N_2 filled glove box. To a 10 mL reaction tube equipped with a stirring bar, were added pyridinium salts (0.1 mmol, 1.0 equiv), benzensulfonothioates (0.25 mmol, 2.5 equiv), Mn (0.5 mmol, 5 equiv), DMSO (0.5 mL, 0.2 M) under N_2 atmosphere. After that, the resulting mixture was sealed with a screw cap and carried out of the glove box, then the resulting mixture was stirred at 70 °C for 20 h. Then saturated aqueous NH_4Cl was added to quench the reaction. The aqueous layer was then extracted with diethyl ether, and the combined organic layers were washed with brine, dried over anhydrous Na_2SO_4, filtered and concentrated. The residue was purified by flash column chromatography on silica gel using Petroleum ether /EtOAc as eluant.

Decarboxylative hydrogenation (General procedure 7)

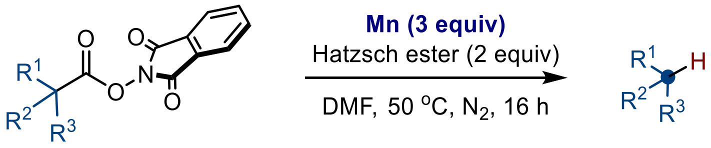

Reactions were set up in a N_2 filled glove box. To a 10 mL reaction tube equipped with a stirring bar, were added NHPI ester (0.2 mmol), diethyl 1,4-dihydro-2,6-dimethyl-3,5-pyridinedicarboxylate (0.4 mmol, 2.0 equiv), Mn (0.6 mmol, 3 equiv) DMF (0.5 mL, 0.4 M) under N_2 atmosphere. After that, the resulting mixture was sealed with a screw cap and carried out of the glove box, then the resulting mixture was stirred at 50 °C for 16 h. Then saturated aqueous NH_4Cl was added to quench the reaction. The aqueous layer was then extracted with diethyl ether, and the combined organic layers were washed with brine, dried over anhydrous Na_2SO_4, filtered and concentrated. The residue was purified by flash column chromatography on silica gel using Petroleum ether /EtOAc as eluant.

Decarboxylative vinylation (General procedure 8)

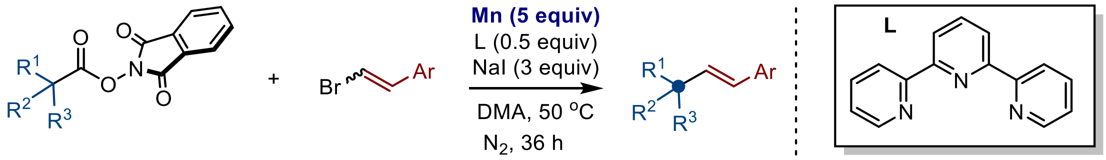

Reactions were set up in a N_2 filled glove box. To a 10 mL reaction tube equipped with a stirring bar, were added NHPI ester (0.2 mmol), vinyl bromide (0.6 mmol, 3.0 equiv), Mn (1.0 mmol, 5.0 equiv), 2,2':6',2''-terpyridine (0.1 mmol, 0.5 equiv), DMA (0.5 mL, 0.4 M) under N_2 atmosphere. After that, the resulting mixture was sealed with a screw cap and carried out of the glove box, then the resulting mixture was stirred at 50 °C for 36 h. Then saturated aqueous NH_4Cl was added to quench the reaction. The aqueous layer was then extracted with diethyl ether, and the combined organic layers were washed with brine, dried over anhydrous Na_2SO_4, filtered and concentrated. The residue was purified by flash column chromatography on silica gel using Petroleum ether /EtOAc as eluant.

Deaminative allylation (General procedure 9)

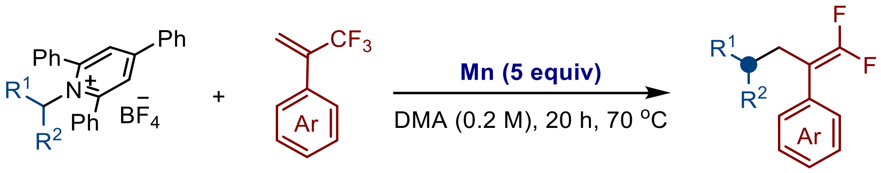

Reactions were set up in a N_2 filled glove box. To a 10 mL reaction tube equipped with a stirring bar, were added pyridinium salt (0.26 mmol, 1.3 equiv), α-(trifluoromethyl)styrene (0.2 mmol, 1.0 equiv), Mn (1.0 mmol, 5.0 equiv), DMA (1.0 mL, 0.2 M) under N_2 atmosphere. After that, the resulting mixture was sealed with a screw cap and carried out of the glove box, then the resulting mixture was stirred at 70 °C for 20 h. Then saturated aqueous NH_4Cl was added to quench the reaction. The aqueous layer was then extracted with diethyl ether, and the combined organic layers were washed with brine, dried over anhydrous Na_2SO_4, filtered and concentrated. The residue was purified by flash column chromatography on silica gel using Petroleum ether /EtOAc as eluant.

Selected Results of Reaction Optimization

Supplementary Table 1. Decarboxylative thiolation

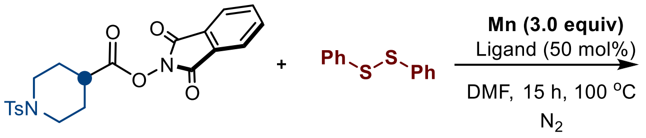

1 **2** **3**

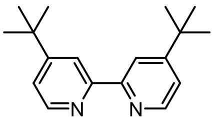

L1

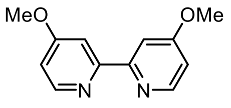

L2

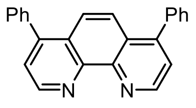

L3

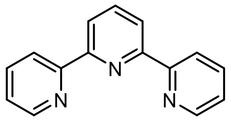

L4

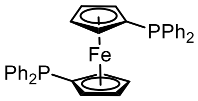

L5

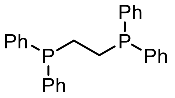

L6

Entry	Ratio (**1**/**2**)	M (x equiv)	Ligand (0.5 equiv)	Additive	Solvent (0.2 M)	Temperature (°C)	Yield (%)
1	2/1	Mn (5)	-	-	DMF	100	63
2	2/1	Mn (5)	L1	-	DMF	100	52
3	2/1	Mn (5)	L2	-	DMF	100	66
4	2/1	Mn (5)	L3	-	DMF	100	69
5	2/1	Mn (5)	L4	-	DMF	100	79
6	2/1	Mn (5)	L5	-	DMF	100	45
7	2/1	Mn (5)	L6	-	DMF	100	49
8	2/1	Mn (5)	L4	-	DMA	100	80
9	2/1	Mn (5)	L4	-	DMPU	100	0
10	2/1	Mn (5)	L4	-	DMSO	100	62
11	2/1	Mn (5)	L4	-	THF	100	46
12	2/1	Mn (5)	L4	-	NMP	100	43
13	2/1	Mn (5)	L4	-	CH_3CN	100	38
14	2/1	Mn (4)	L4	-	DMA	100	78
15	2/1	Mn (3)	L4	-	DMA	100	83
**16**	**1.5/1**	**Mn (3)**	**L4**		**DMA**	**100**	**81**
17	1.5/1	-	L4	-	DMA	100	0
18	1.5/1	-	-	-	DMA	100	0
19	1.5/1	Mn (3)	L4	LiBr (1 equiv)	DMA	100	35
20	1.5/1	Mn (3)	L4	NaBr (1 equiv)	DMA	100	53
21	1.5/1	Mn (3)	L4	KBr (1 equiv)	DMA	100	48
22	1.5/1	Mn (3)	L4	NaOAc (1 equiv)	DMA	100	52
23	1.5/1	Mn (3)	L4	TMSCl (0.5 equiv)	DMA	100	58
24	1.5/1	Mn (3)	L4	-	DMA	80	0
25	1.5/1	-	L4	-	DMA	100	0

26	1.5/1	Mn (3)	-	-	DMA	100	60
27	1.5/1	Mn (2)	L4	-	DMA	100	58
28	1/1.5	Mn (3)	L4	-	DMA	100	61
29	1.5/1	Mn (3)	L4	ZnCl2 (0.5 equiv)	DMA	100	51
30	1.5/1	Mn (3)	L4		DMA	100	0 [a]
31	1.5/1	Mn (3)	L4		DMA	100	76 [b]
32	1.5	Fe (3)	L4	-	DMA	100	0
33	1.5	Co (3)	L4	-	DMA	100	23
34	1.5	Ni (3)	L4	-	DMA	100	8
35	1.5	Cu (3)	L4	-	DMA	100	0
36	1.5	Zn (3)	L4	-	DMA	100	5

Reaction scale: 0.2 mmol; isolated yield.

a: inert atmosphere air

b: inert atmosphere N_2

(The reaction of entry 30 and 31 performed without using glove box, and have performed the reaction on bench.)

Supplementary Table 2. Deaminative thiolation.

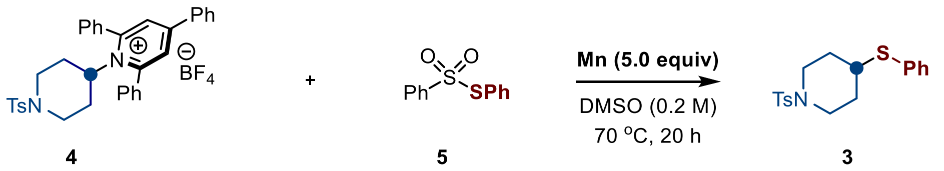

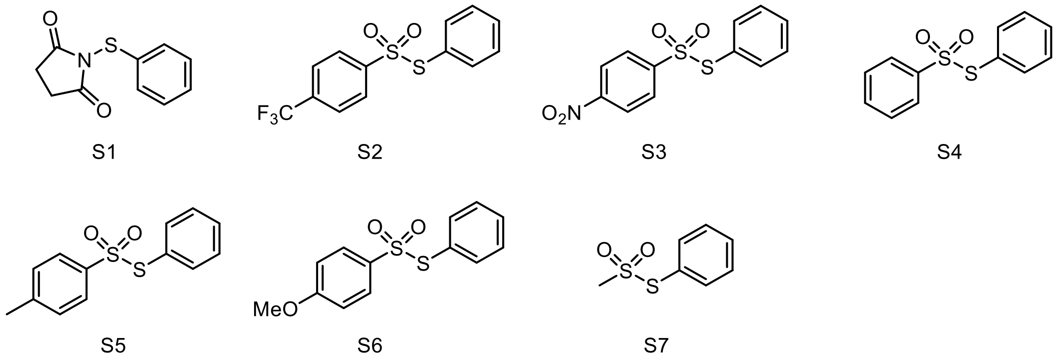

Entry	5 (x equiv)	S-reagent	M (y equiv)	Solvent	Temperature (°C)	Yield (%)
1	2	S1	Mn (5)	DMF	25	<5
2	2	S2	Mn (5)	DMF	25	17
3	2	S3	Mn (5)	DMF	25	<5
4	2	S4	Mn (5)	DMF	25	35
5	2	S5	Mn (5)	DMF	25	43
6	2	S6	Mn (5)	DMF	25	55
7	2	S7	Mn (5)	DMF	25	32
8	2	S6	Mn (5)	DMF	50	23
9	2	S6	Mn (5)	DMF	70	29
10	2.0	S6	Mn (5)	DMSO	70	60
11	2.5	S6	Mn (5)	DMSO	70	77
12	2.5	S6	Mn (5)	DMA	70	71
13	2.5	S6	Mn (5)	DMF	70	31
**14**	**2.5**	**S4**	**Mn (5)**	**DMSO**	**70**	**93**
15	2.5	S5	Mn (4)	DMSO	70	77
16	2.5	S4	Mn (4)	DMSO	70	78
17	2.5	S4	Mn (3)	DMSO	70	63
18	2.5	S4	-	DMSO	70	0

Reaction scale: 0.1 mmol; isolated yield.

Supplementary Table 3. Decarboxylative hydrogenation.

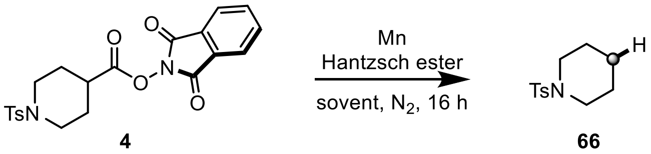

Entry	Mn (x equiv)	Hantzsch ester (y equiv)	Solvent (0.4 M)	Temperature (°C)	Yield (%)
1	3	2	DMF	25	35
2	3	2	THF	25	5
3	3	2	CH_3CN	25	23
4	3	2	DCM	25	19
5	3	2	DMA	25	30
**6**	**3**	**2**	**DMF**	**50**	**85**
7	2	2	DMF	50	78
8	3	1.5	DMF	50	80
9	4	2	DMF	50	83
10	5	2	DMF	50	86
11	-	2	DMF	50	15
12	3	-	DMF	50	17

Reaction scale: 0.2 mmol; isolated yield.

Supplementary Table 4. Decarboxylative vinylation**.**

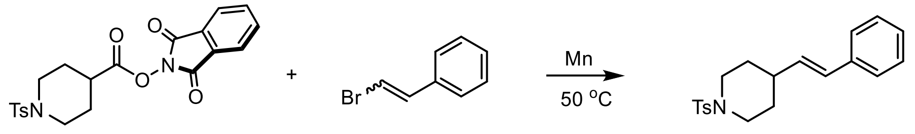

1 SI-5-1

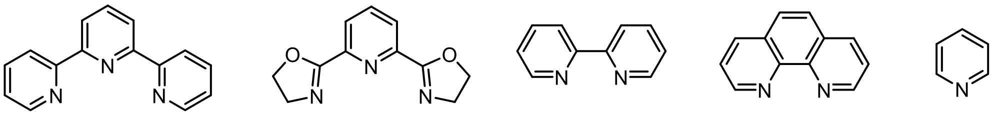

L1 L2 L3 L4 L5

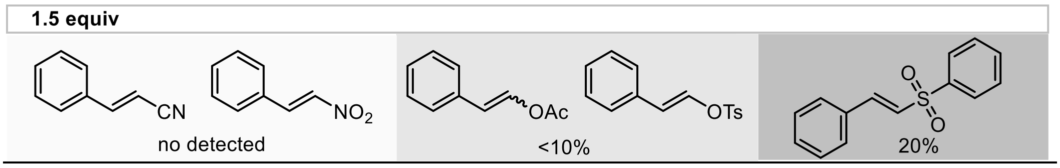

Entry	SI-5-1 (x eq)	Mn (y equiv)	Ligand	additive	Solvent	Yield (%)
1	1.5	Mn (5)	-	-	DMF	22
2	1.5	Mn (5)	L1	-	DMF	35
3	1.5	Mn (5)	L2	-	DMF	23
4	1.5	Mn (5)	L3	-	DMF	15
5	1.5	Mn (5)	L4	-	DMF	17
6	1.5	Mn (5)	L5	-	DMF	20
7	1.5	Mn (5)	L1	--	DMA	41
8	1.5	Mn (5)	L1	-	DMPU	28
9	1.5	Mn (5)	L1	-	HMPA	<5
10	1.5	Mn (5)	L1	-	NMP	37
11	1.5	Mn (5)	L1	-	CH_3CN	trace
12	1.5	Mn (5)	L1	-	THF	0
13	1.5	Mn (5)	L1	TBAI(1.0 equiv)	DMA	37
14	1.5	Mn (5)	L1	$MgBr_2$(1.0 equiv)	DMA	18
15	1.5	Mn (5)	L1	NaI(1.0 equiv)	DMA	44
16	1.5	Mn (5)	L1	NaI(1.5 equiv)	DMA	43
17	1.5	Mn (5)	L1	NaI(2.0 equiv)	DMA	47
18	2.0	Mn (5)	L1	NaI(2.0 equiv)	DMA	51
**19**	**3.0**	**Mn (5)**	**L1**	**NaI(2.0 equiv)**	**DMA**	**59**
20	3.0	Mn (3)	L1	NaI(2.0 equiv)	DMF	49

Reaction scale: 0.2 mmol; isolated yield.

Supplementary Table 5. Deaminative difluoroallylation.

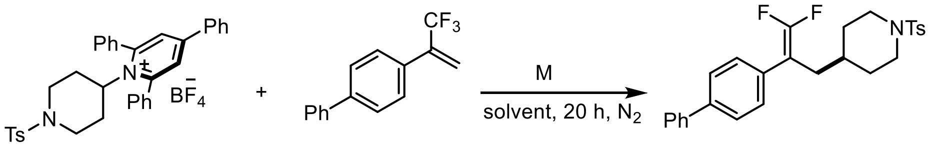

4 SI-6-1

Entry	M (x equiv)	Solvent(0.2 M)	4/SI-6-1	Temperature(°C)	Yield(%)
**1**	Mn (5)	DMF	2/3	25	51
**2**	Zn (5)	DMF	2/3	25	34
**3**	Mn (5)	DMA	2/3	25	60
**4**	Mn (5)	DMPU	2/3	25	26
**5**	Mn (5)	NMP	2/3	25	49
**6**	Mn (5)	CH_3CN	2/3	25	49
**7**	Mn (5)	THF	2/3	25	0
**8**	Mn (5)	1,4-dioxane	2/3	25	0
**9**	Mn (5)	DMSO	2/3	25	56
**10**	Mn (5)	DCE	2/3	25	Trace
**11**	Mn(5)	DMA(0.1 M)	2/3	25	34
**12**	Mn(5)	DMA	1.3/1	25	70
**13**	Mn(5)	DMA	1.3/1	50	84
**14**	**Mn(5)**	**DMA**	**1.3/1**	**70**	**95**
**15**	Mn(4)	DMA	1.3/1	70	85
**16**	Mn(3)	DMA	1.3/1	70	63

Reaction scale: 0.2 mmol; isolated yield.

Mechanistic Studies

Radical-clock experiments:

a Decarboxylative thiolation of RAEs

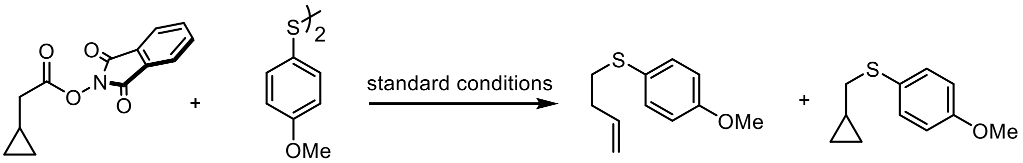

130 (0.3 mmol) **131** (0.2 mmol) **132** 72% **133** no detected

Reactions were set up in a N_2 filled glove box. To a 10 mL reaction tube equipped with a stirring bar, were added NHPI ester (**130**, 0.3 mmol, 1.5 equiv), disulfide (**131**, 0.2 mmol, 1.0 equiv), Mn (0.6 mmol, 3 equiv) and 2,2′:6′,2″-terpyridine (0.1 mmol, 0.5 equiv), DMA (1.0 mL, 0.2 M) under N_2 atmosphere. After that, the resulting mixture was sealed with a screw cap and carried out of the glove box, then the resulting mixture was stirred at 100 ^{o}C for 15 h. Then saturated aqueous NH_4Cl was added to quench the reaction. The aqueous layer was then extracted with diethyl ether, and the combined organic layers were washed with brine, dried over anhydrous Na_2SO_4, filtered and concentrated. The residue was purified by flash column chromatography on silica gel (Petroleum ether/EtOAc = 100/1) to give the desired products **132** (white solid, 28 mg, 72%)

Rf = 0.60 (EtOAc/Petroleum Ether = 1/50)

1H NMR (400 MHz, $CDCl_3$) δ 7.36 (d, J = 8.8 Hz, 2H), 6.85 (d, J = 8.8 Hz, 2H), 6.00 – 5.66 (m, 1H), 5.12 – 4.83 (m, 2H), 3.80 (s, 3H), 3.03 – 2.79 (m, 2H), 2.33 (q, J = 7.3 Hz, 2H).

^{13}C NMR (101 MHz, $CDCl_3$) δ 159.0, 136.7, 133.5, 126.4, 116.2, 114.7, 55.5, 35.3, 33.7.

HRMS (ESI): m/z calculated for $C_{11}H_{14}OS$ $[M+K]^+$, 233.0397; found, 233.0402.

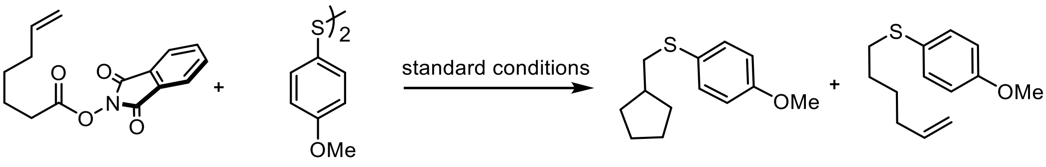

134 (0.3 mmol) **131** (0.2 mmol) **135** 57% **136** 7%

Reactions were set up in a N_2 filled glove box. To a 10 mL reaction tube equipped with a stirring bar, were added NHPI ester (**134**, 0.3 mmol, 1.5 equiv), disulfide (**131**, 0.2 mmol, 1.0 equiv), Mn (0.6 mmol, 3 equiv) and 2,2′:6′,2″-terpyridine (0.1 mmol, 0.5 equiv), DMA (1.0 mL, 0.2 M) under N_2 atmosphere. After that, the resulting mixture was sealed with a screw cap and carried out of the glove box, then the resulting mixture was stirred at 100 ^{o}C for 15 h. Then saturated aqueous NH_4Cl was added to quench the reaction. The aqueous layer was then extracted with diethyl ether, and the combined organic layers were washed with brine, dried over anhydrous Na_2SO_4, filtered and concentrated. The residue was purified by flash column chromatography on silica gel (Petroleum ether/EtOAc = 100/1) to give the desired products **135 and 136** (white solid, 28 mg)

Compound 135

^{1}H NMR (500 MHz, Chloroform-*d*) δ 7.34 (d, *J* = 8.7 Hz, 2H), 6.84 (d, *J* = 8.8 Hz, 2H), 3.79 (s, 3H), 2.83 (d, *J* = 7.3 Hz, 2H), 2.10 – 1.96 (m, 1H), 1.82 (dq, *J* = 11.9, 7.0 Hz, 2H), 1.66 – 1.60 (m, 2H), 1.55 – 1.49 (m, 2H), 1.26 (dq, *J* = 15.5, 7.8 Hz, 2H).

^{13}C NMR (126 MHz, Chloroform-*d*) δ 158.6, 132.7, 127.4, 114.5, 55.3, 42.1, 39.5, 32.3, 25.3.

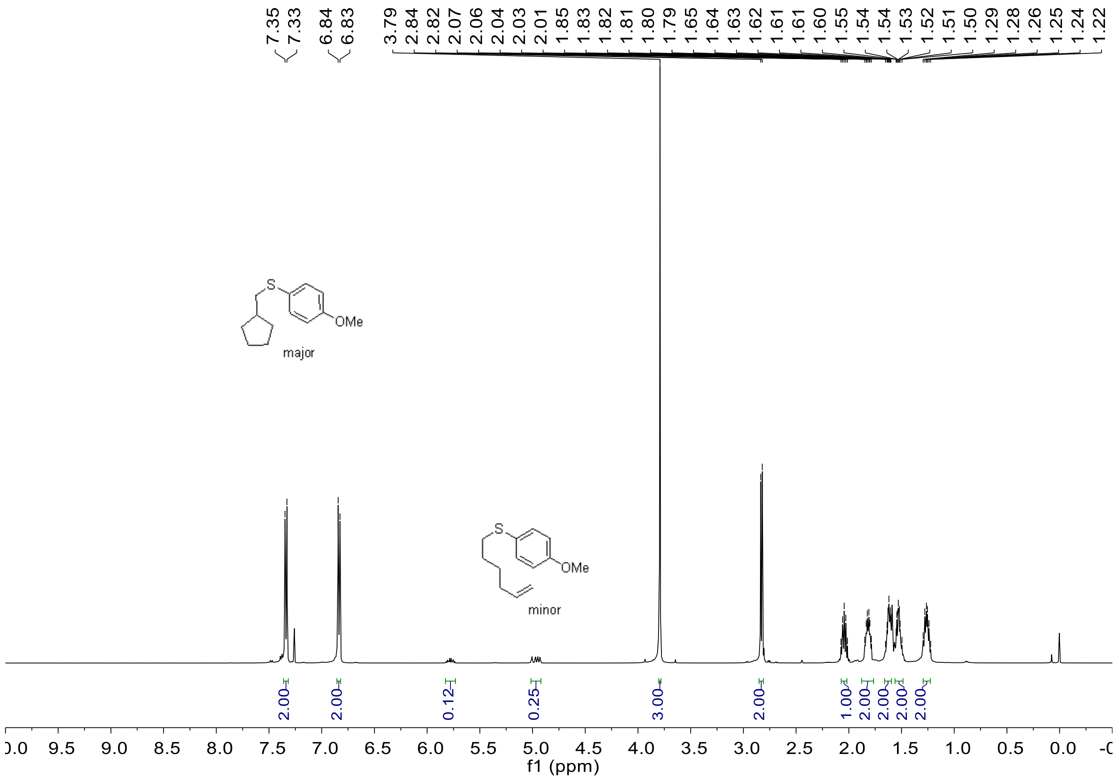

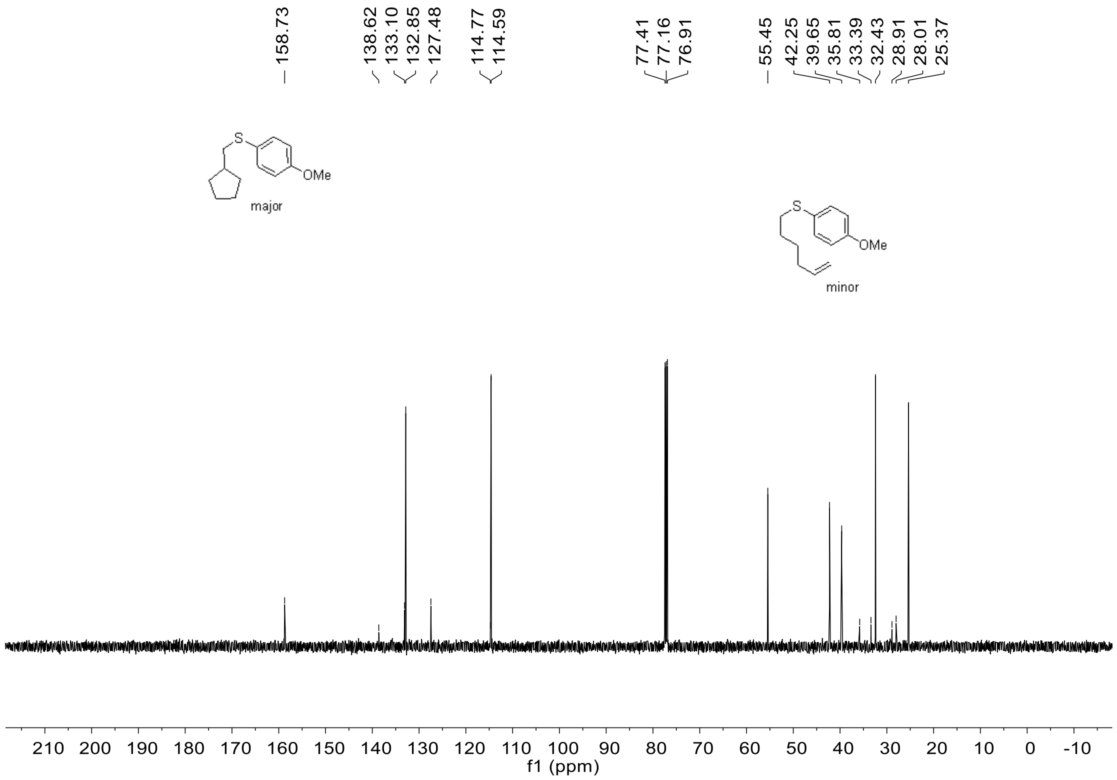

b Deaminative thiolation of *N*-alkylpyridinium salts

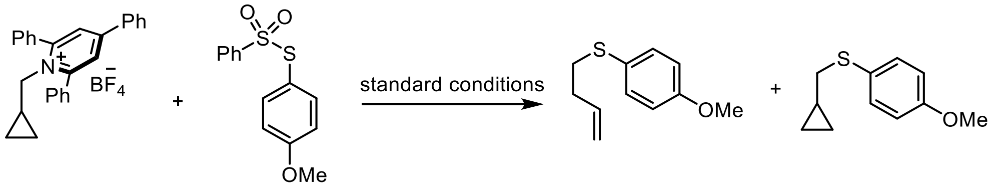

Reactions were set up in a N_2 filled glove box. To a 10 mL reaction tube equipped with a stirring bar, were added pyridinium salt (**137**, 0.1 mmol, 1.0 equiv), benzensulfonothioate (**138**, 0.25 mmol, 2.5 equiv), Mn (0.5 mmol, 5 equiv), DMSO (0.5 mL, 0.2 M) under N_2 atmosphere. After that, the resulting mixture was sealed with a screw cap and carried out of the glove box, then the resulting mixture was stirred at 70 ^{o}C for 20 h. Then saturated aqueous NH_4Cl was added to quench the reaction. The aqueous layer was then extracted with diethyl ether, and the combined organic layers were washed with brine, dried over anhydrous Na_2SO_4, filtered and concentrated. The residue was purified by flash column chromatography on silica gel (Petroleum ether/EtOAc = 100/1) to give the desired products **132** (white solid, 10 mg, 26%)

Radical-trapping experiments

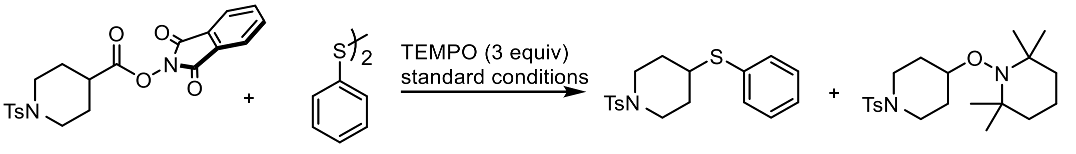

1 (0.3 mmo)l | 2 (0.2 mmol) | 3 no detected | 139 20%

Reactions were set up in a N_2 filled glove box. To a 10 mL reaction tube equipped with a stirring bar, were added NHPI ester (**1**, 0.3 mmol, 1.5 equiv), disulfide (**2**, 0.2 mmol, 1.0 equiv), Mn (0.6 mmol, 3 equiv) and 2,2′:6′,2″-terpyridine (0.1 mmol, 0.5 equiv), DMA (1.0 mL, 0.2 M) under N_2 atmosphere. After that, the resulting mixture was sealed with a screw cap and carried out of the glove box, then the resulting mixture was stirred at 100 ^oC for 15 h. Then saturated aqueous NH_4Cl was added to quench the reaction. The aqueous layer was then extracted with diethyl ether, and the combined organic layers were washed with brine, dried over anhydrous Na_2SO_4, filtered and concentrated. The residue was purified by flash column chromatography on silica gel (Petroleum ether/EtOAc = 100/1) to give the desired products **139** (white solid, 16 mg, 20% (compared disulfide))

Compound 139[27] (CAS: 2114323-18-9)

^1H NMR (400 MHz, Chloroform-*d*) δ 7.64 (d, *J* = 8.2 Hz, 2H), 7.31 (d, *J* = 8.0 Hz, 2H), 3.66 – 3.52 (m, 3H), 2.49 – 2.40 (m, 5H), 2.08 – 1.98 (m, 2H), 1.69 – 1.62 (m, 3H), 1.44 -1.37 (m, 4H), 1.29 – 1.27 (m, 1H), 1.05 (s, 12H).

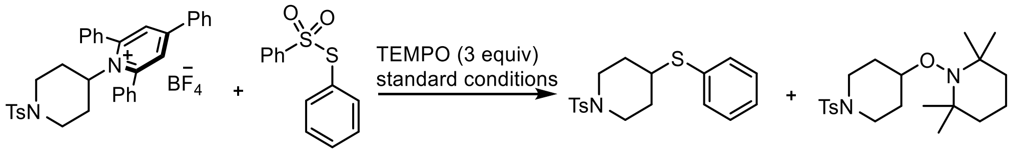

4 (0.1 mmol) | 5 (0.15 mmol) | 3 12% | 4 18%

Reactions were set up in a N_2 filled glove box. To a 10 mL reaction tube equipped with a stirring bar, were added pyridinium salt (**4**, 0.1 mmol, 1.0 equiv), benzensulfonothioates (**5**, 0.25 mmol, 2.5 equiv), Mn (0.5 mmol, 5 equiv), TEMPO (94 mg, 0.6 mmol), DMSO (0.5 mL, 0.2 M) under N_2 atmosphere. After that, the resulting mixture was sealed with a screw cap and carried out of the glove box, then the resulting mixture was stirred at 70 ^oC for 20 h. Then saturated aqueous NH_4Cl was added to quench the reaction. The aqueous layer was then extracted with diethyl ether,

and the combined organic layers were washed with brine, dried over anhydrous Na_2SO_4, filtered and concentrated. The residue was purified by flash column chromatography on silica gel (petroleum ether/ethyl acetate) to give the products **3** (white solid, 4 mg, 12%) and 4 (white solid, 7 mg, 18%).

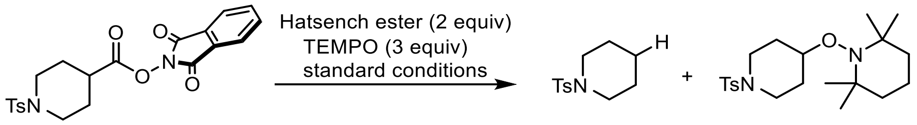

1 (0.2 mmol) **66** trace **139** detected

Reactions were set up in a N_2 filled glove box. To a 10 mL reaction tube equipped with a stirring bar, were added NHPI ester (0.2 mmol, 1.0 equiv), diethyl 1,4-dihydro-2,6-dimethyl-3,5-pyridinedicarboxylate (0.4 mmol, 2.0 equiv), Mn (0.6 mmol, 3 equiv), TEMPO (94 mg, 0.6 mmol), DMF (0.5 mL, 0.4 M) under N_2 atmosphere. After that, the resulting mixture was sealed with a screw cap and carried out of the glove box, then the resulting mixture was stirred at 50 ^{o}C for 16 h. Then diethyl ether and saturated aqueous NH_4Cl was added to quench the reaction. After, the mixture of diethyl ether was detected by HRMS and TLC.

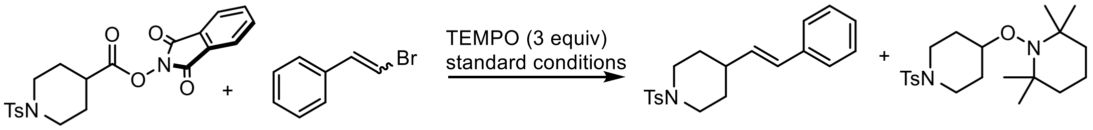

1 (0.2 mmol) **SI-5-1** (0.6 mmol) **85** no detected **139** 8%

Reactions were set up in a N_2 filled glove box. To a 10 mL reaction tube equipped with a stirring bar, were added NHPI ester (0.2 mmol, 1.0 equiv), Vinyl bromine (0.6 mmol, 3.0 equiv), Mn (1.0 mmol, 5.0 equiv), 2,2',:6',2''-terpyridine (0.1 mmol, 0.5 equiv), TEMPO (94 mg, 0.6 mmol), DMA (0.5 mL, 0.4 M) under N_2 atmosphere. After that, the resulting mixture was sealed with a screw cap and carried out of the glove box, then the resulting mixture was stirred at 50 ^{o}C for 36 h. Then saturated aqueous NH_4Cl was added to quench the reaction. The aqueous layer was then extracted with diethyl ether, and the combined organic layers were washed with brine, dried over anhydrous Na_2SO_4, filtered and concentrated. The residue was purified by flash column chromatography on silica gel (petroleum ether/ethyl acetate) to give the products **139** (white solid, 6 mg, 8%).

Other experiments

a

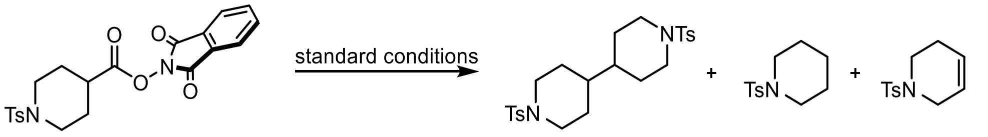

1 detected detected no detected

Reactions were set up in a N_2 filled glove box. To a 10 mL reaction tube equipped with a stirring bar, were added NHPI ester (**1**) (0.3 mmol, 1.5 equiv), Mn (0.6 mmol, 3 equiv) and 2,2′:6′,2″-terpyridine (0.1 mmol, 0.5 equiv), DMA (1.0 mL, 0.2 M) under N_2 atmosphere. After that, the resulting mixture was sealed with a screw cap and carried out of the glove box, then the resulting mixture was stirred at 100 ^{o}C for 5 h. Then saturated aqueous NH_4Cl was added to quench the reaction. The aqueous layer was then extracted with diethyl ether. After, the mixture of diethyl ether was detected by HRMS.

b

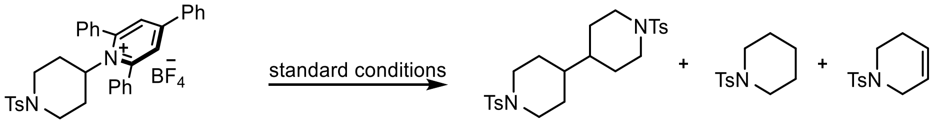

4 no detected detected detected

Reactions were set up in a N_2 filled glove box. To a 10 mL reaction tube equipped with a stirring bar, were added pyridinium salt (4) (0.1 mmol, 1.0 equiv), benzensulfonothioates (0.25 mmol, 2.5 equiv), Mn (0.5 mmol, 5 equiv), DMSO (0.5 mL, 0.2 M) under N_2 atmosphere. After that, the resulting mixture was sealed with a screw cap and carried out of the glove box, then the resulting mixture was stirred at 70 ^{o}C for 5 h. Then saturated aqueous NH_4Cl was added to quench the reaction. The aqueous layer was then extracted with diethyl ether. After, the mixture of diethyl ether was detected by HRMS.

c

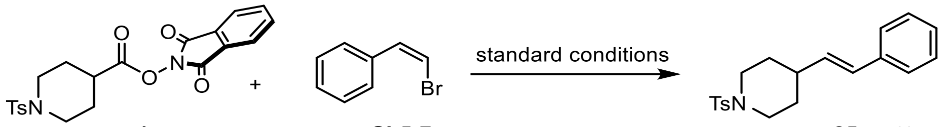

1 **SI-5-7** **85**, 54%

Reactions were set up in a N_2 filled glove box. To a 10 mL reaction tube equipped with a stirring bar, were added NHPI ester (0.2 mmol, 1.0 equiv), Vinyl bromine (**SI-5-7**, 0.6 mmol, 3.0 equiv), Mn (1.0 mmol, 5.0 equiv), 2, 2', 6', 2''-terpyridine (0.1 mmol, 0.5 equiv), DMA (0.5 mL, 0.4 M) under N_2 atmosphere. After that, the resulting mixture was sealed with a screw cap and carried out of the glove box, then the resulting mixture was stirred at 50 ^oC for 36 h. Then saturated aqueous NH_4Cl was added to quench the reaction. The aqueous layer was then extracted with diethyl ether, and the combined organic layers were washed with brine, dried over anhydrous Na_2SO_4, filtered and concentrated. The residue was purified by flash column chromatography on silica gel (petroleum ether/ethyl acetate) to give the desired products **85** (white solid, 37 mg, 54%).

d

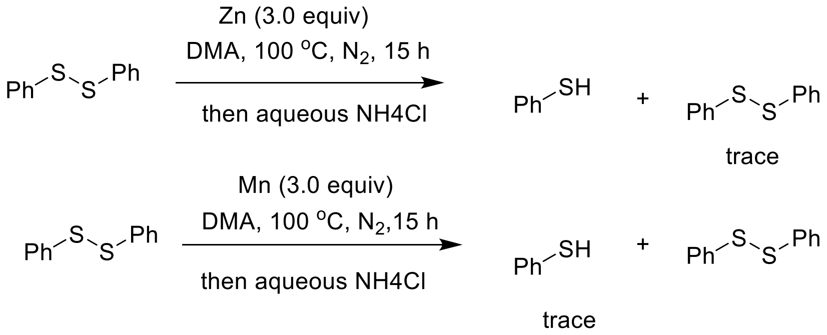

The low yield when zinc was used is probably due to the undesired reduction of disulfide to the corresponding thiolate. This was observed experimentally when heating disulfide with zinc or manganese. The reaction outcomes were determined by GC-MS.

e

We have prepared two analogs of NHPI ester **1**: bromide **1-Br** and iodide **1-I**. Interestingly, both substrates showed good reactivity in this thiolation reaction under the standard reaction conditions. An explanation to this observation is that the disulfide may be reduced to a thiolate, and then a S_N2 substitution reaction took place to form the thiolation product. Separate experiments showed that the reaction of **1-Br** or **1-I** with thiolate gave a moderate yield of the thiolated product, confirming our hypothesis.

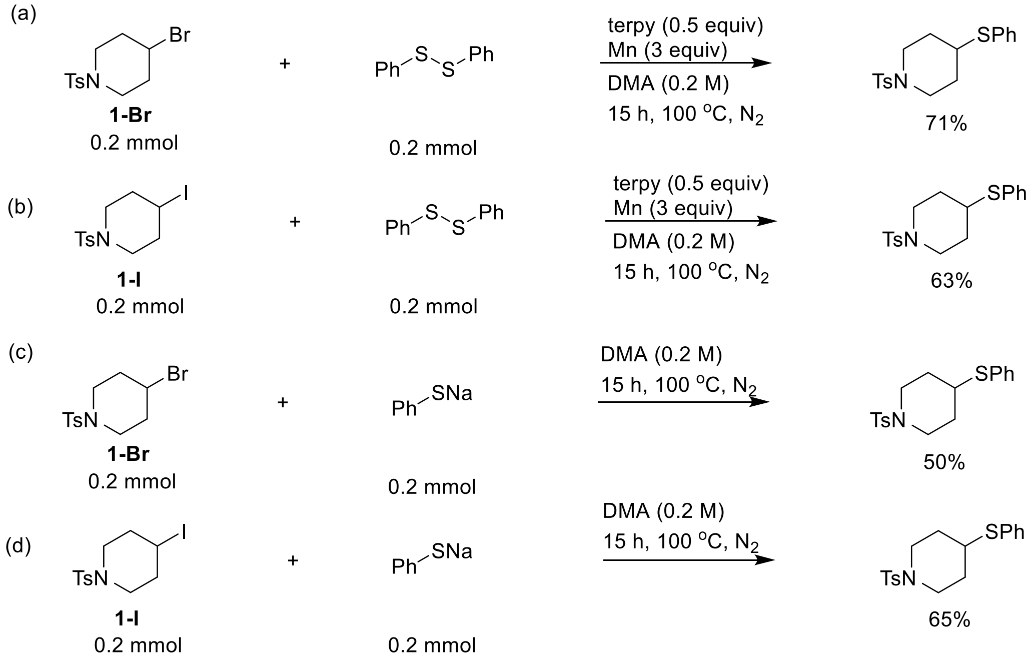

f

Two halogen-containing (Br, Cl) NHPI ester**s were also prepared**. The reaction of both substrates showed that the thiolation on both the halogen and NHPI ester sites were observed. Nevertheless, under the decarboxylative hydrogenation conditions, chemoselective hydrogenation of the NHPI ester was detected.

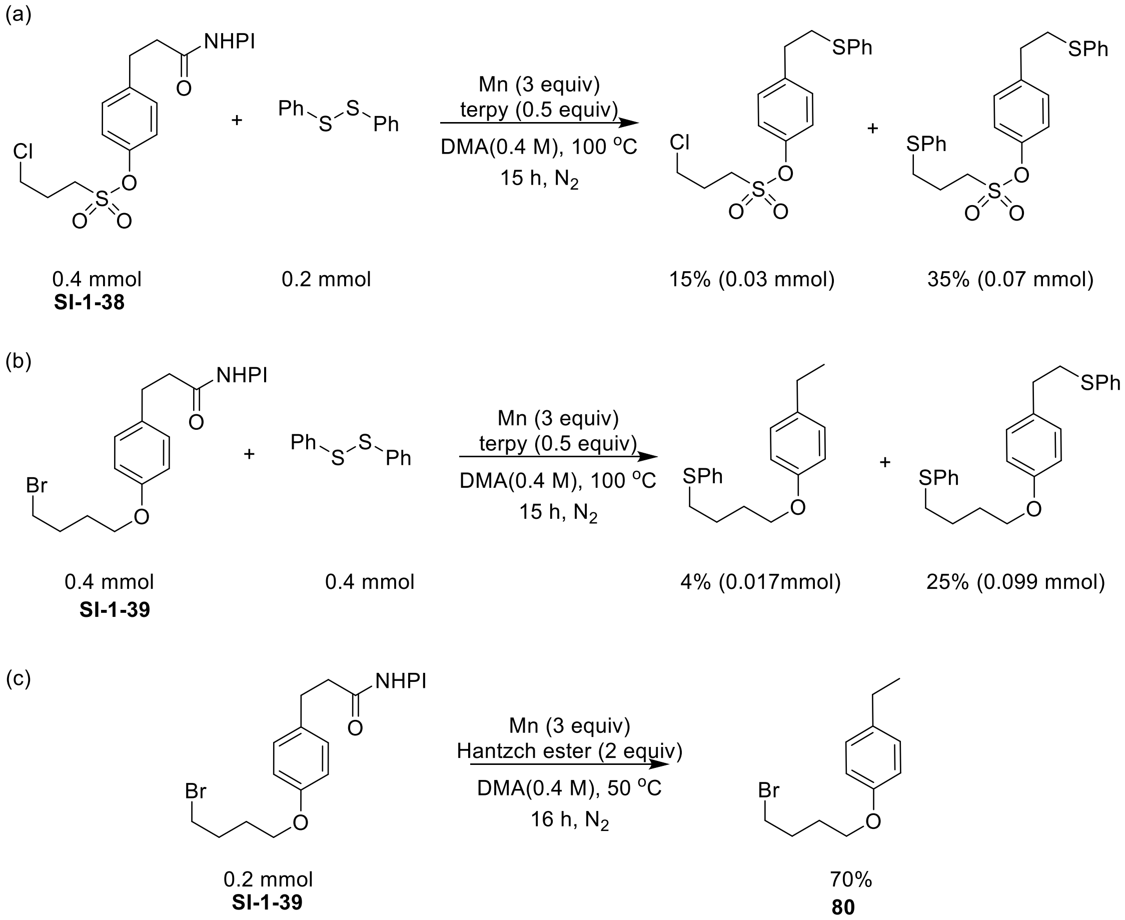

(a) Reactions were set up in a N_2 filled glove box. To a 10 mL reaction tube equipped with a stirring bar, were added NHPI ester **(SI-1-38)**(0.4 mmol, 2 equiv), disulfide (**2**) (0.2 mmol, 1.0 equiv), Mn (0.6 mmol, 3 equiv) and 2,2′:6′,2″-terpyridine (0.1 mmol, 0.5 equiv), DMA (1.0 mL, 0.2 M) under N_2 atmosphere. After that, the resulting mixture was sealed with a screw cap and carried out of the glove box, then the resulting mixture was stirred at 100 °C for 15 h. Then saturated aqueous NH_4Cl was added to quench the reaction. The aqueous layer was then extracted with diethyl ether, and the combined organic layers were washed with brine, dried over anhydrous Na_2SO_4, filtered and concentrated. The residue was purified by flash column chromatography on silica gel using Petroleum ether /EtOAc as eluant and afforded monothiolation (11 mg, 15% yield, as oil) and dithiolation (33 mg, 35% yield, as a white solid) product.

4-(2-(phenylthio)ethyl)phenyl 3-chloropropane-1-sulfonate

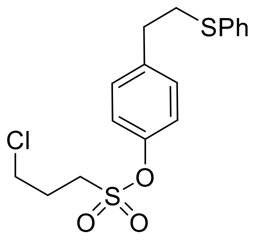

^{1}H NMR (400 MHz, Chloroform-*d*) δ 7.39 – 7.34 (m, 2H), 7.31 (t, *J* = 7.6 Hz, 2H), 7.26 – 7.22 (m, 1H), 7.22 – 7.18 (m, 2H), 7.14 – 7.08 (m, 2H), 3.44 – 3.35 (m, 2H), 3.09 (t, *J* = 6.8 Hz, 2H), 2.65 (q, *J* = 7.6 Hz, 2H), 2.27 (p, *J* = 6.9 Hz, 2H), 1.23 (t, *J* = 7.6 Hz, 1H).

^{13}C NMR (101 MHz, $CDCl_3$) δ 147.18, 143.62, 134.80, 130.39, 129.42, 129.34, 127.00, 121.87, 48.86, 32.48, 28.43, 23.36, 15.61.

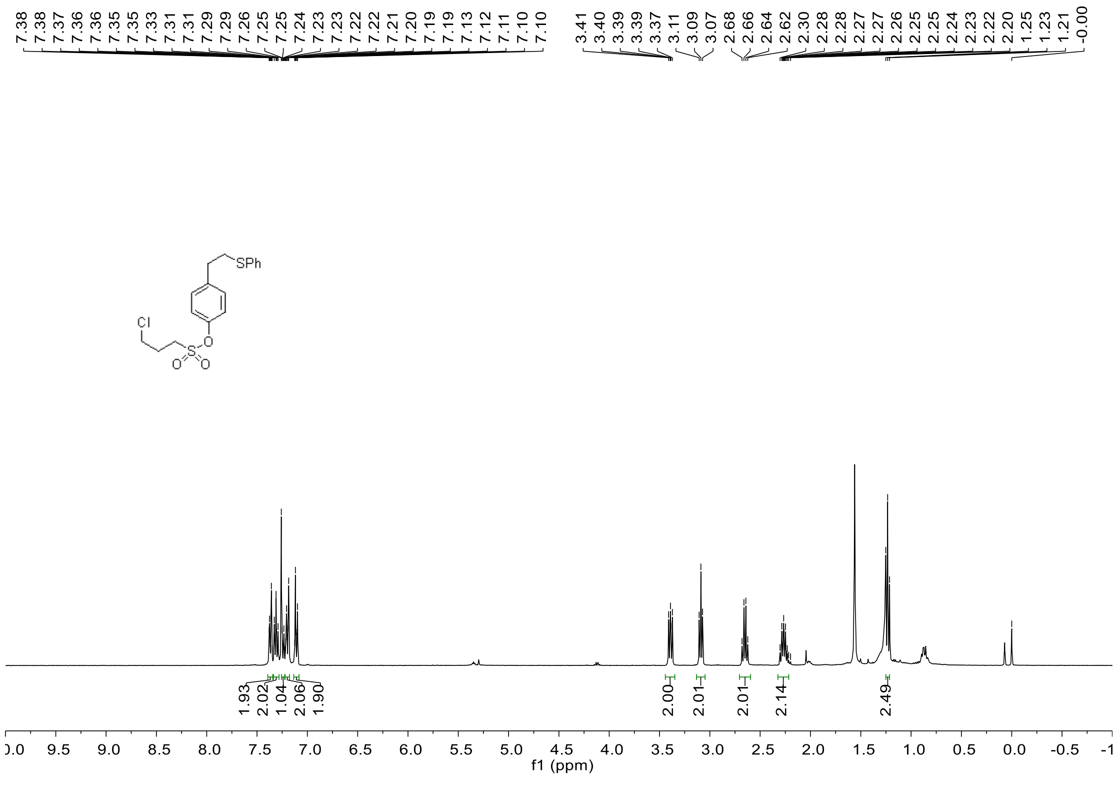

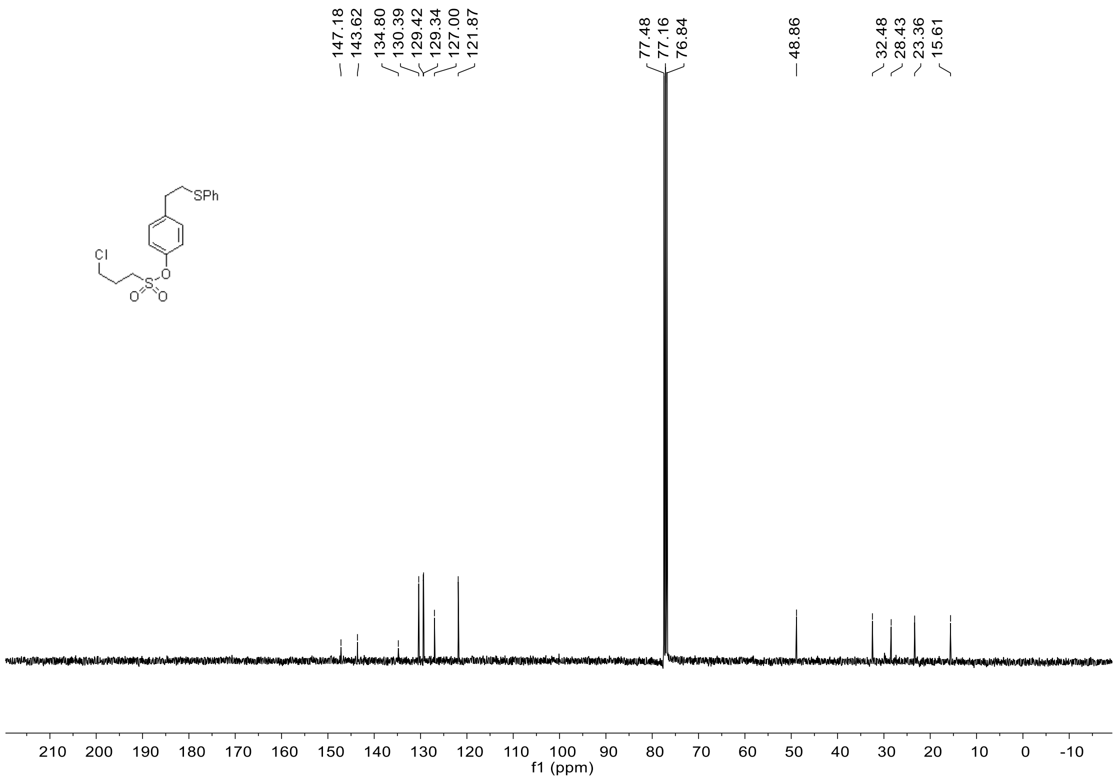

4-(2-(phenylthio)ethyl)phenyl 3-(phenylthio)propane-1-sulfonate

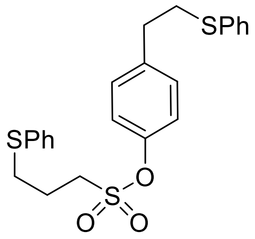

^{1}H NMR (400 MHz, Chloroform-*d*) δ 7.31 – 7.10 (m, 12H), 7.08 – 7.02 (m, 2H), 3.32 (dd, *J* = 8.6, 6.4 Hz, 2H), 3.07 (dd, *J* = 8.9, 6.5 Hz, 2H), 3.01 (t, *J* = 6.8 Hz, 2H), 2.85 (dd, *J* = 8.9, 6.5 Hz, 2H), 2.19 (p, *J* = 6.9 Hz, 2H).

^{13}C NMR (101 MHz, $CDCl_3$) δ 147.72, 139.56, 136.11, 134.76, 130.35, 130.18, 129.60, 129.33, 129.13, 126.99, 126.36, 122.10, 48.96, 35.18, 35.12, 32.42, 23.33.

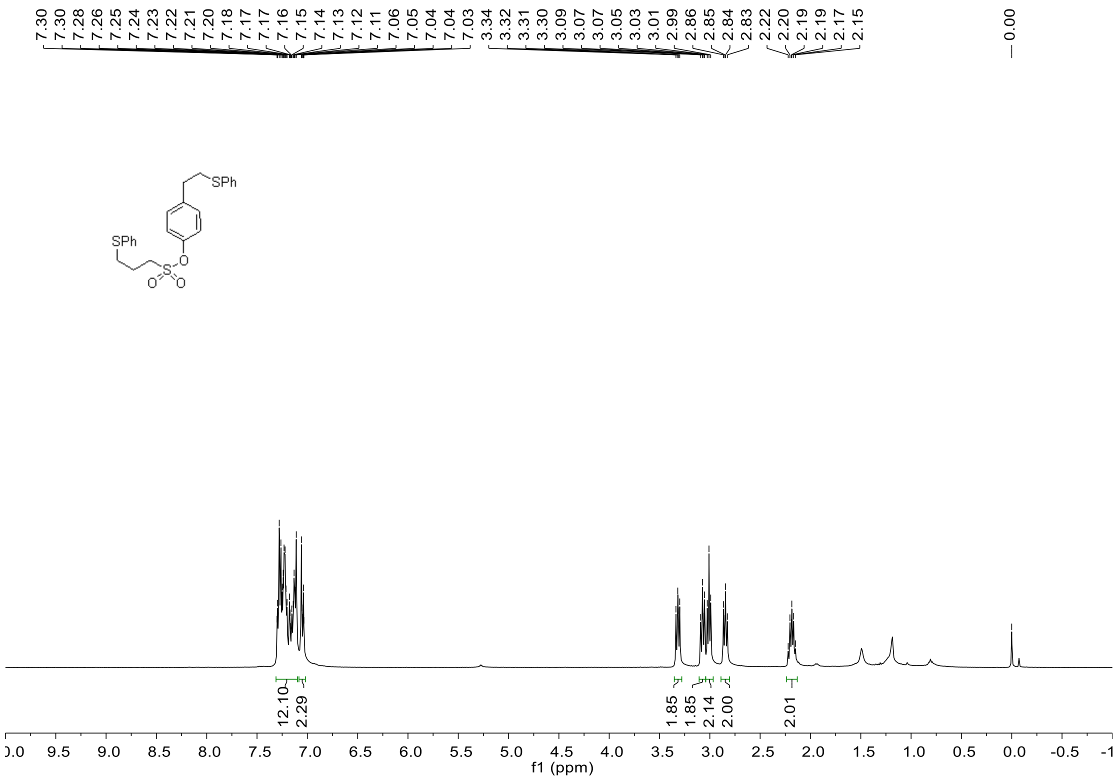

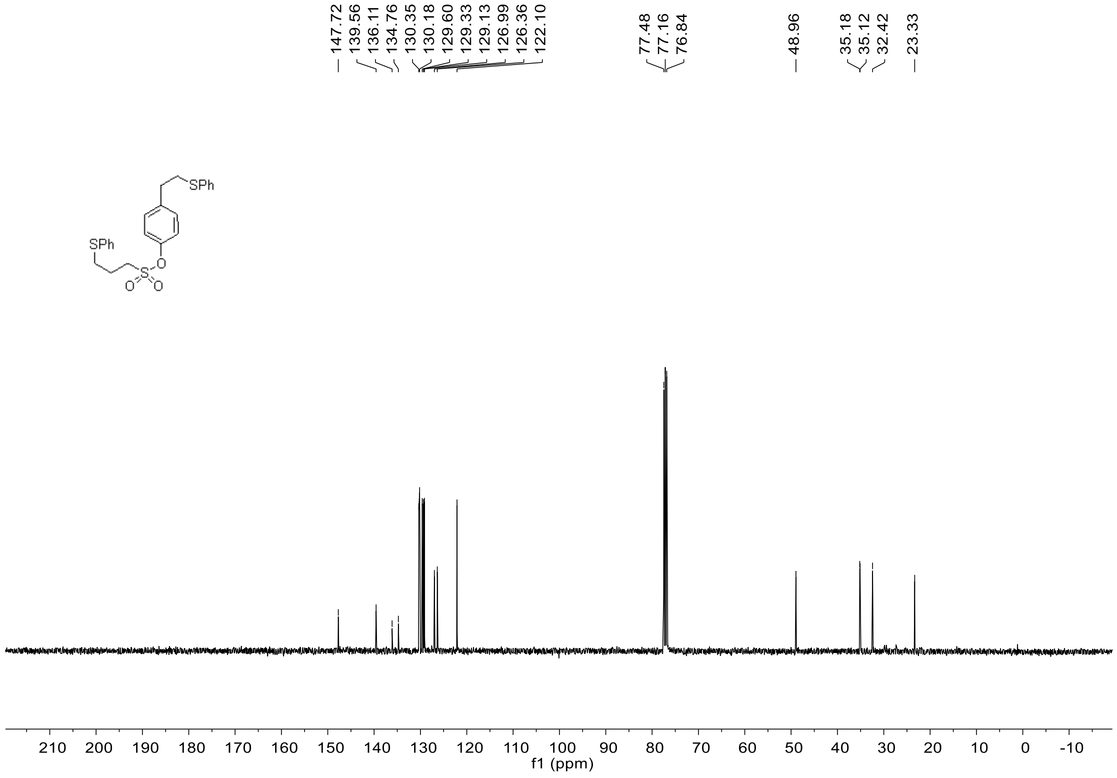

(b) Reactions were set up in a N_2 filled glove box. To a 10 mL reaction tube equipped with a stirring bar, were added NHPI ester **(SI-1-39)**(0.4 mmol, 1 equiv), disulfide (**2**) (0.4 mmol, 1.0 equiv), Mn (1.2 mmol, 3 equiv) and 2,2′:6′,2″-terpyridine (0.2 mmol, 0.5 equiv), DMA (1.0 mL, 0.2 M) under N_2 atmosphere. After that, the resulting

mixture was sealed with a screw cap and carried out of the glove box, then the resulting mixture was stirred at 100 ^{o}C for 15 h. Then saturated aqueous NH_4Cl was added to quench the reaction. The aqueous layer was then extracted with diethyl ether, and the combined organic layers were washed with brine, dried over anhydrous Na_2SO_4, filtered and concentrated. The residue was purified by flash column chromatography on silica gel using Petroleum ether /EtOAc as eluant and afforded 44 mg product as oil (monothiolation/dithiolation = 1/6).

^{1}H NMR (400 MHz, Chloroform-*d*) δ 7.36 – 7.24 (m, 8H), 7.17 (dddt, *J* = 7.2, 5.1, 3.7, 1.8 Hz, 2H), 7.12 – 7.05 (m, 2H), 6.80 (dd, *J* = 8.8, 2.5 Hz, 2H), 3.99 (t, *J* = 6.7 Hz, 0.15H), 3.94 (t, *J* = 6.0 Hz, 2H), 3.13 (dd, *J* = 8.9, 6.8 Hz, 2H), 2.98 (t, *J* = 7.1 Hz, 2H), 2.86 (dd, *J* = 9.1, 6.5 Hz, 2H), 2.58 (q, *J* = 7.6 Hz, 0.34H), 1.94 – 1.78 (m, 5H), 1.20 (t, *J* = 7.6 Hz, 0.48H).

^{13}C NMR (101 MHz, $CDCl_3$) δ 157.68, 136.64, 136.57, 132.41, 129.58, 129.28, 129.25, 129.03, 129.00, 128.81, 126.03, 126.00, 114.72, 114.62, 114.47, 77.48, 77.16, 76.84, 67.38, 35.47, 34.86, 33.50, 28.49, 28.44, 28.10, 25.92, 16.03.

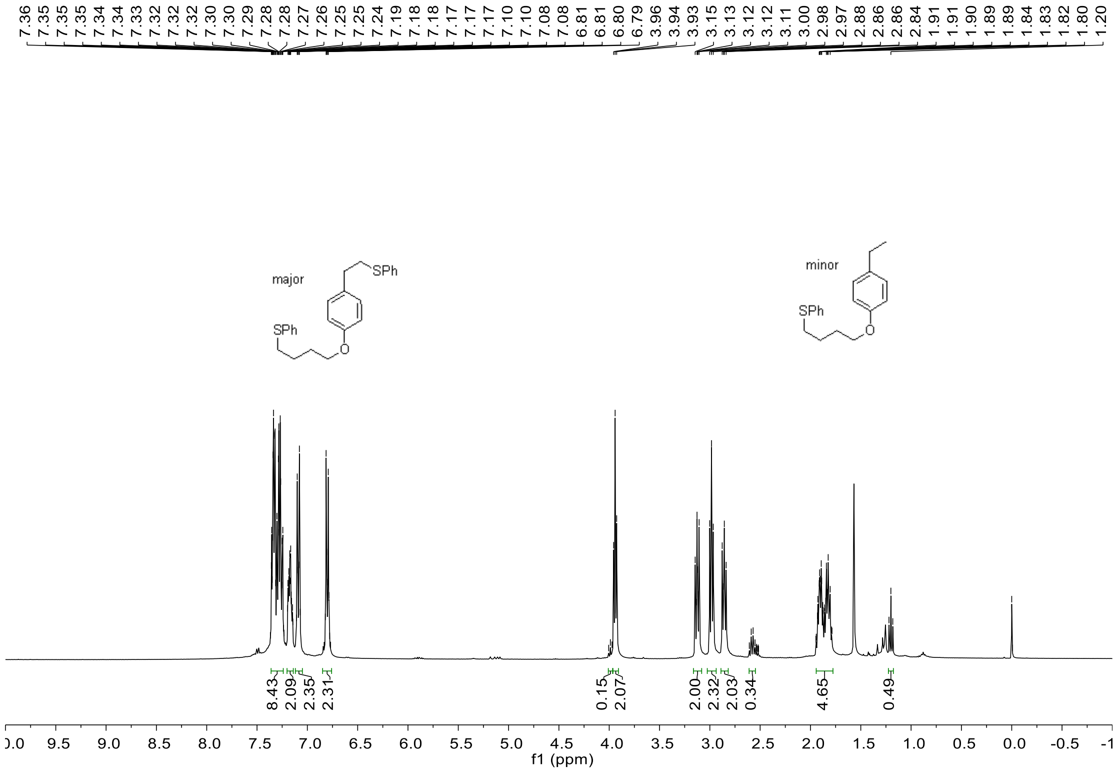

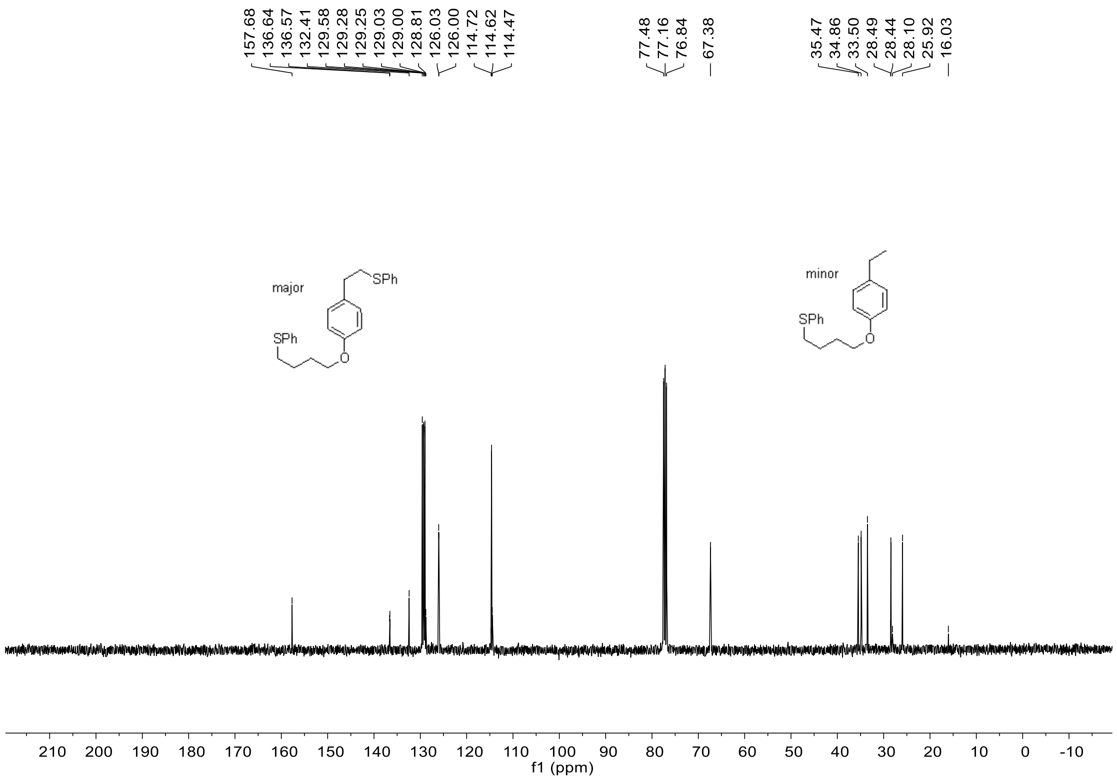

Kinetics

a Decarboxylative thiolation

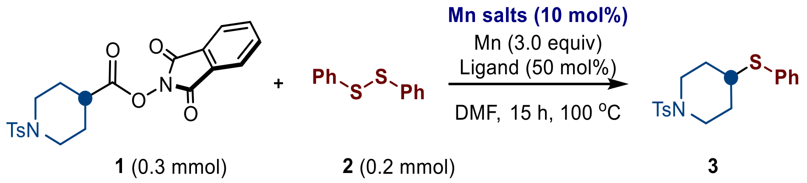

Reactions were set up in a N_2 filled glove box. To a 10 mL reaction tube equipped with a stirring bar, were added NHPI ester (**1**) (0.3 mmol, 1.5 equiv), disulfide (**2**) (0.2 mmol, 1.0 equiv), Mn (0.6 mmol, 3 equiv) and 2,2′:6′,2″-terpyridine (0.1 mmol, 0.5 equiv), Mn salts (0.02 mmol, 0.1 equiv), DMA (1.0 mL, 0.2 M) under N_2 atmosphere. After that, the resulting mixture was sealed with a screw cap and carried out of the glove box, then the resulting mixture was stirred at 100 ^{o}C. Then saturated aqueous NH_4Cl was added to quench the reaction, and then extracted with diethyl ether. The corresponding yield of each product was determined by ^{1}H NMR (1,3,5-trimethoxybenzene as standard).

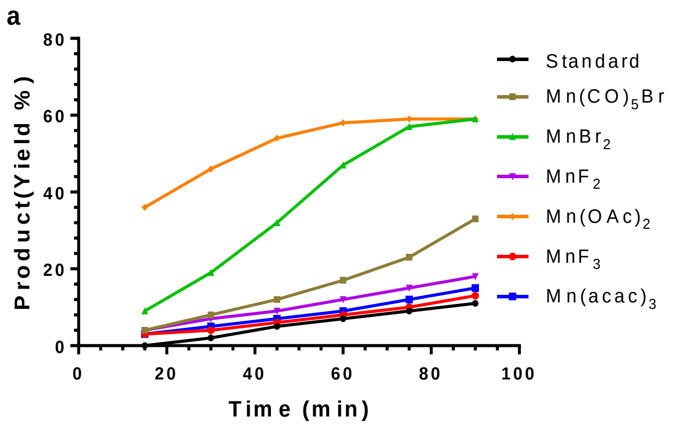

b Deaminative thiolation

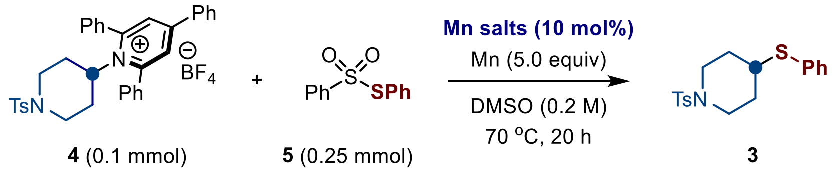

Reactions were set up in a N_2 filled glove box. To a 10 mL reaction tube equipped with a stirring bar, were added pyridinium salt (4) (0.1 mmol, 1.0 equiv), benzensulfonothioates (0.25 mmol, 2.5 equiv), Mn salts (0.01 mmol, 0.1 equiv), Mn (0.5 mmol, 5 equiv), DMSO (0.5 mL, 0.2 M) under N_2 atmosphere. After that, the resulting mixture was sealed with a screw cap and carried out of the glove box, then the resulting mixture was stirred at 70 ^{o}C. Then saturated aqueous NH_4Cl was added to quench the reaction, and then extracted with diethyl ether. The corresponding yield of each product was determined by ^{1}H NMR (1, 3, 5-trimethoxybenzene as standard).

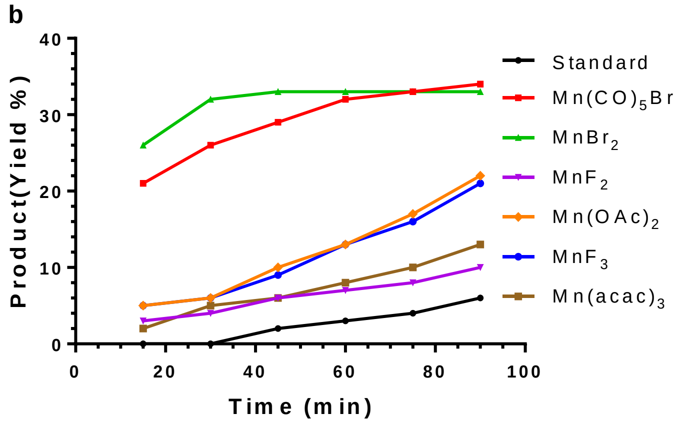

Cyclic Voltammetry

Cyclic voltammetry was conducted on an IGS 1230 electrochemical work station (Ingsens instruments, Guangzhou) using a 3-electrode cell configuration. A glassy carbon working electrode was employed alongside a platinum wire counter electrode and a Ag/AgCl (KCl 3 M.) reference electrode. The distance between the working and reference electrode was 1cm.

a

The RAEs (**1**) were prepared as 0.05 M solutions in DMA (dry) along with 0.1 M supporting electrolyte (tetrabutylammonium hexafluorophosphate). The 1,2-diphenyldisulfane (**2**) were prepared as 0.1 M solutions in DMSO (dry) along with 0.1 M supporting electrolyte (tetrabutylammonium hexafluorophosphate).

b

The pyridiniums (**4**) and S-phenyl benzenesulfonothioate (**5**) were prepared as 0.1 M solutions in DMSO (dry) along with 0.1 M supporting electrolyte (tetrabutylammonium hexafluorophosphate).

Argon was passed through the samples for 20 minutes before taking any measurements and an Ar atmosphere was maintained for the duration to avoid the deleterious influence of oxygen reduction. Samples were examined at scan rates of 100 mV s-1 depending on the substrate.

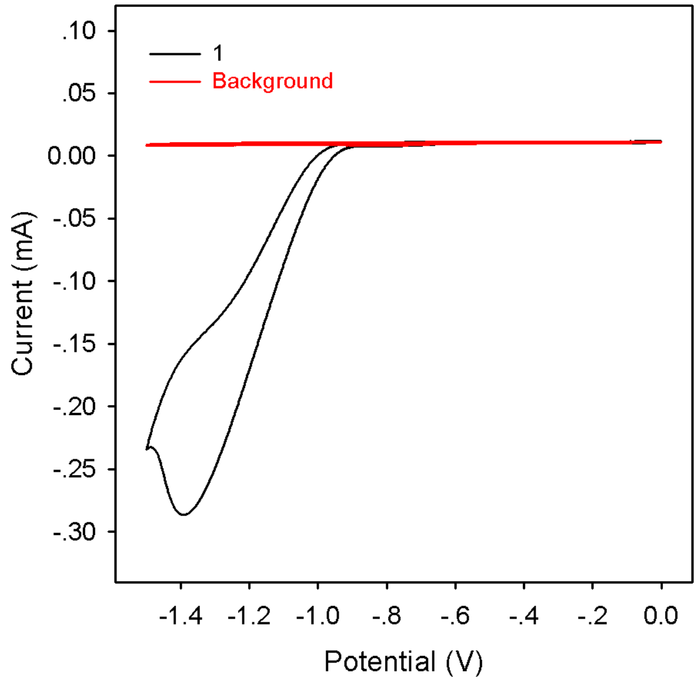

Supplementary Figure 1. The reduction Potential of 1,3-dioxoisoindolin-2-yl 1-tosylpiperidine-4-carboxylate (**1** $E_{1/2}$ = -1.15 V vs Ag/AgCl)

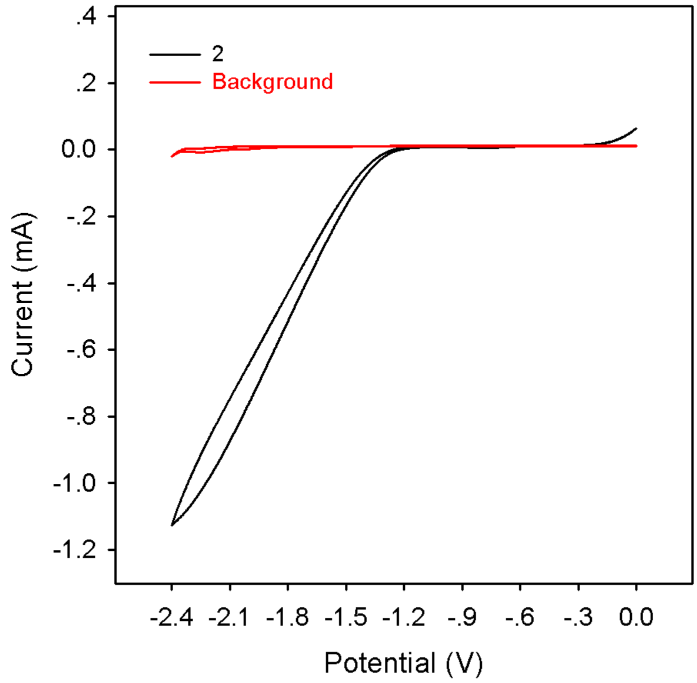

Supplementary Figure 2. The reduction Potential of 1,2-diphenyldisulfane (**2** $E_{1/2}$ = -1.85 V vs Ag/AgCl)

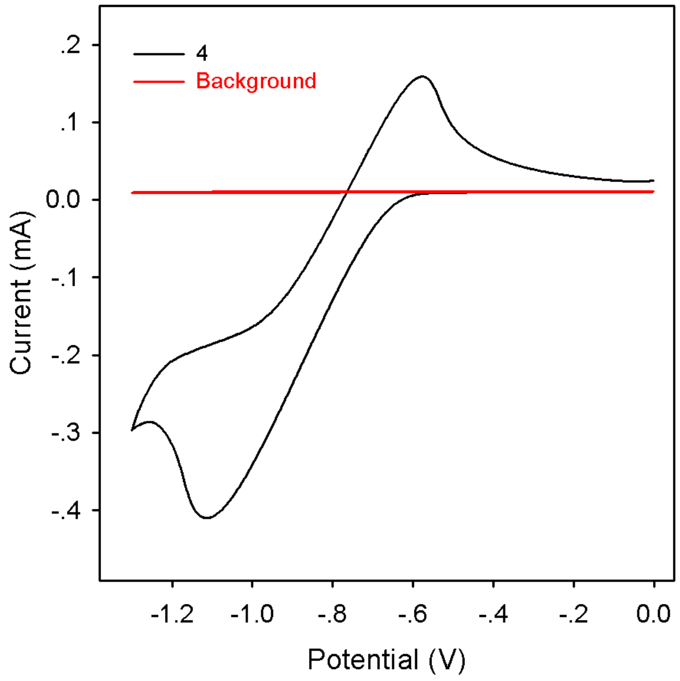

Supplementary Figure 3. The reduction Potential of Katritzky's salt (**4** $E_{1/2}$ = -0.85 V vs Ag/AgCl)

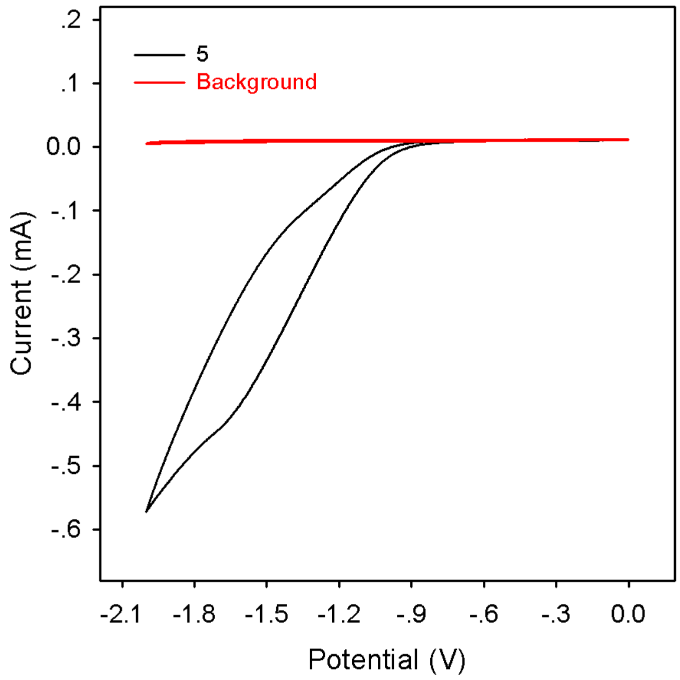

Supplementary Figure 4. The reduction Potential of S-phenyl benzenesulfonothioate (**5** $E_{1/2}$ = -1.35 V vs Ag/AgCl)

The role of ligand

a The use of chiral tridentate ligand

Three chiral tridentate ligands in lieu of terpyridine were used in the reaction. No apparent ee was found. These results indicate that the ligand or Mn-ligand complex is not involved in the C-S bond formation step.

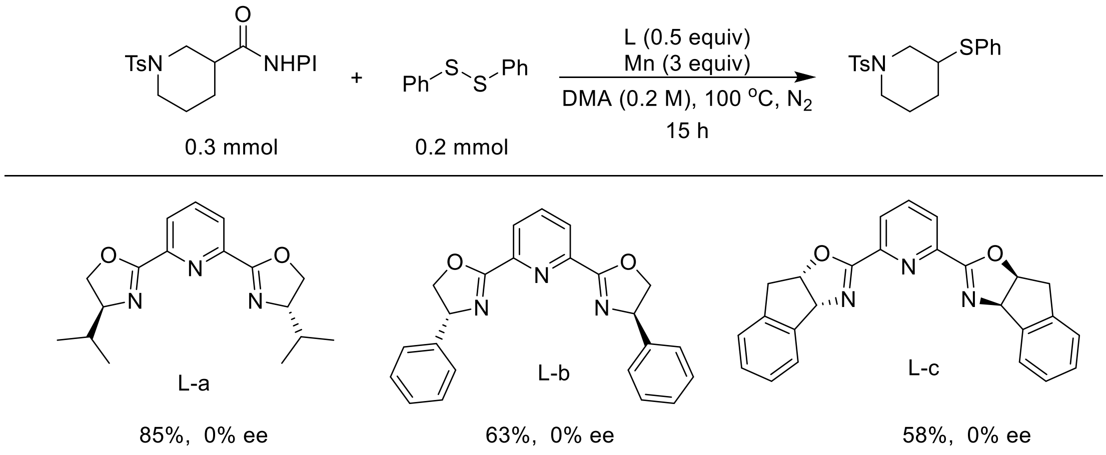

b The use of pre-formed Mn-terpyridine complex as additive

Mn(terpy)Cl_2 complex, a tentative mediator/catalyst for the reaction, was prepared according to a precedent literature (*Angew. Chem. Int. Ed.* **2016**, 55, 14369-14372). Its application (0.5 equiv or 0.1 equiv.) in the thiolation reaction give an inferior yield as compared to terpyridine.

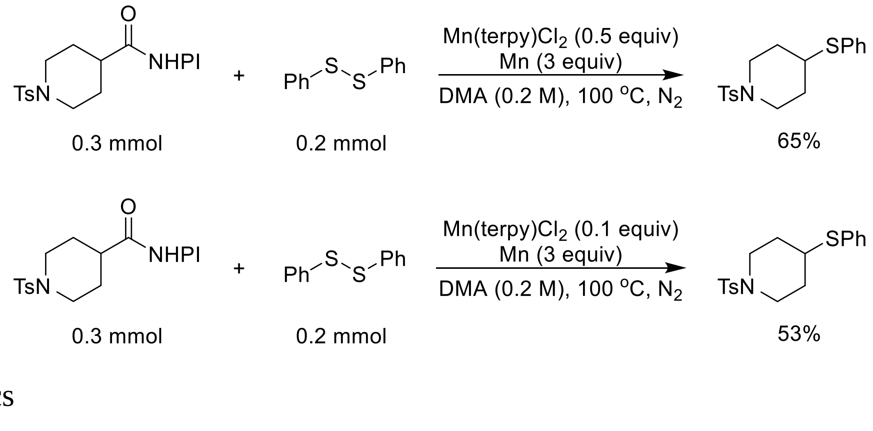

c Kinetics

Reactions were set up in a N_2 filled glove box. To a 10 mL reaction tube equipped with a stirring bar, were added NHPI ester (**1**) (0.3 mmol, 1.5 equiv), disulfide (**2**) (0.2 mmol, 1.0 equiv), Mn (0.6 mmol, 3 equiv) and with or without 2,2′:6′,2″-terpyridine (0.1 mmol, 0.5 equiv), DMA (1.0 mL, 0.2 M) under N_2 atmosphere. After that, the resulting mixture was sealed with a screw cap and carried out of the glove box, then the resulting mixture was stirred at 100 ^{o}C. Then saturated aqueous NH_4Cl was added to quench the reaction, and then extracted with diethyl ether. The corresponding yield of each product was determined by ^{1}H

NMR (1,3,5-trimethoxybenzene as standard).

The kinetics for both the reactions with terpyridine and without terpyridine were measured. Out of our expectation, a slow reaction rate was observed when terpyridine was used.

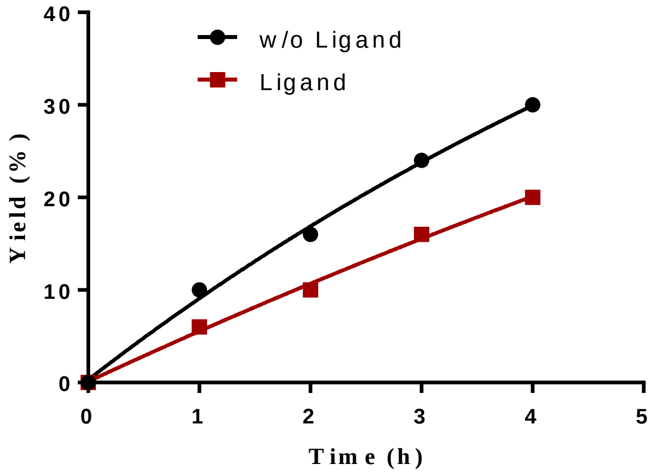

d Some results without ligand

We have tried more examples without terpyridine ligand. The results were shown below. We can see the role of terpyridine is not decisive. For compounds **8**, **22** and **45**, similar yields were observed.

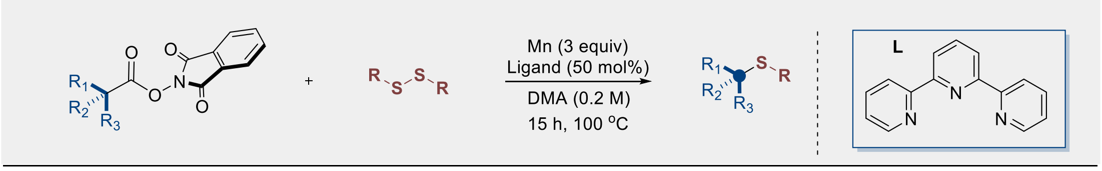

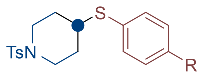

3, R = H, 81%
(without ligand, 60%)

8, R = Br, 97%
(without ligand, 90%)

10, R = Me, 72%
(without ligand, 60%)

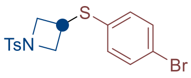

22, 47%
(without ligand, 45%)

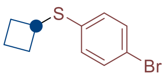

24, 81%
(without ligand, 63%)

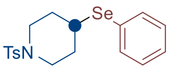

45, 80%[b]
(without ligand, 75%)

Reaction conditions.[a] NHPI ester (0.3 mmol), disulfide (0.2 mmol), Mn (0.6 mmol), in DMA (0.2 M), 100 °C, N_2, 15 h; yields are for isolated products. [b] NHPI ester (0.4 mmol).

In all, the use of terpyridine in our reaction is beneficial for the yield. However, its role is not decisive for both the reactivity and efficacy. Similar observation was actually found in Hu's manganese-mediated reductive transamidation reaction wherein bipyridine was used as ligand (*J. Am. Chem. Soc.* **2018**, 140, 6789−6792). The above results gave no clues on the tricky role of terpyridine in our reaction.

In situ Activation Protocol and Synthetic Applications

In situ activation protocol

a Decarboxylative thiolation

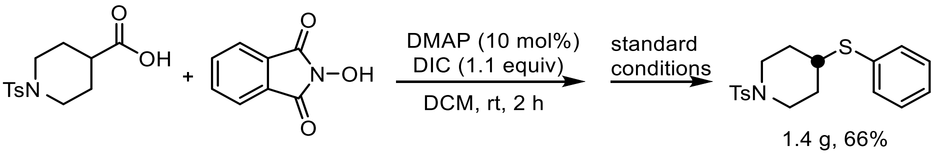

To a reaction tube equipped with a stirring bar, were added 1-tosylpiperidine-4-carboxylic acid (9 mmol, 1.5 equiv), 2-hydroxyisoindoline-1,3-dione (9 mmol, 1.5 equiv), *N*,*N'*-diisopropylcarbodiimide (9.9 mmol, 1.65 equiv), dimethylaminopyridine (0.9 mmol, 0.15 equiv) and DCM (36 mL, 0.25 M). The resulting mixture was sealed with a rubber stopper and protected with an Ar balloon. The resulting mixture was stirred at room temperature for 0.5 h and concentrated under reduced pressure. After, Mn (18 mmol, 3 equiv), 1,2-diphenyldisulfane (6 mmol, 1 equiv), 2,2′:6′,2″-terpyridine (3 mmol, 0.5 equiv), DMA (30 mL, 0.2 M) under N_2 atmosphere. After that, the resulting mixture was sealed with a screw cap and the resulting mixture was stirred at 100 ^{o}C for 15 h. Then saturated aqueous NH_4Cl was added to quench the reaction. The aqueous layer was then extracted with diethyl ether, and the combined organic layers were washed with brine, dried over anhydrous Na_2SO_4, filtered and concentrated. The residue was purified by flash column chromatography on silica gel (petroleum ether/ethyl acetate) to give the desired products **3** (white solid, 1.4 g, 66%).

b Decarboxylative hydrogenation

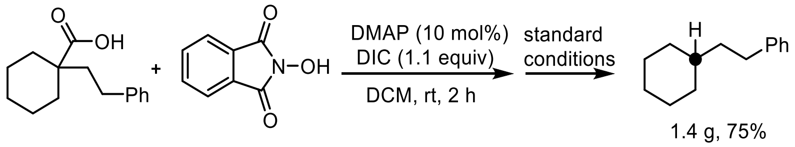

To a reaction tube equipped with a stirring bar, were added 1-phenethylcyclohexane-1-carboxylic acid (9.9 mmol, 1.5 equiv), 2-hydroxyisoindoline-1,3-dione (9.9 mmol, 1.5 equiv), *N*,*N'*-diisopropylcarbodiimide (16.3 mmol, 1.65 equiv), dimethylaminopyridine (0.99 mmol, 0.15 equiv) and DCM (40mL, 0.25 M). The resulting mixture was sealed with a rubber stopper and protected

with an Ar balloon. The resulting mixture was stirred at room temperature for 0.5 h and concentrated under reduced pressure. After, Mn (29.7 mmol, 3 equiv), Hantzsch ester (19.8 mmol, 2 equiv), DMF (25 mL, 0.4 M) under N_2 atmosphere. After that, the resulting mixture was sealed with a screw cap and the resulting mixture was stirred at 50 ^{o}C for 16 h. Then saturated aqueous NH_4Cl was added to quench the reaction. The aqueous layer was then extracted with diethyl ether, and the combined organic layers were washed with brine, dried over anhydrous Na_2SO_4, filtered and concentrated. The residue was purified by flash column chromatography on silica gel (petroleum ether/ethyl acetate) to give the desired products (white solid, 1.4 g, 75%).

Synthetic Applications

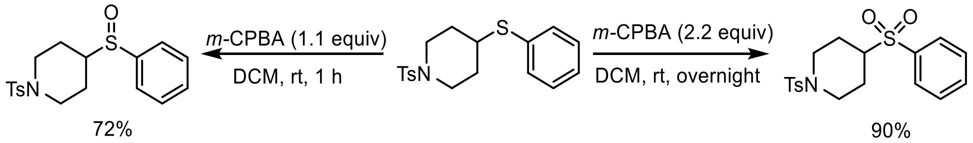

a Synthesis of sulfoxide

To a reaction tube equipped with a stirring bar, were added **3** (1.3 mmol, 1 equiv), *m*-CPBA (1.36 mmol, 1.05 equiv) and DCM (13 mL, 0.1 M). The resulting mixture was stirred at room temperature for 1 h. Then the reaction was quenched with 1.0 M NaOH (20 mL). The aqueous layer was extracted with DCM for 3 times. The combined organic layer was dried over anhydrous Na_2SO_4 and concentrated and subjected to flash column chromatography (10:1 Dichloromethane / Ethyl acetate) to afford the desired product (333 mg, 72% yield) as a white solid.

Rf = 0.80 (1:1 Dichloromethane / Ethyl acetate)

^{1}H NMR (400 MHz, $CDCl_3$) δ 7.57 (d, *J* = 8.0 Hz, 2H), 7.55 – 7.50 (m, 2H), 7.50 – 7.45 (m, 3H), 7.27 (d, *J* = 8.1 Hz, 2H), 3.80 (t, *J* = 12.8 Hz, 2H), 2.46 (ddt, *J* = 11.7, 8.0, 4.0 Hz, 1H), 2.39 (s, 3H), 2.28 (td, *J* = 10.2, 8.6, 3.3 Hz, 2H), 1.88 (d, *J* = 12.9 Hz, 1H), 1.84 - 1.74 (m, 2H), 1.74 – 1.60 (m, 1H).

^{13}C NMR (126 MHz, $CDCl_3$) δ 143.9, 141.0, 133.0, 131.6, 129.8, 129.2, 127.7, 125.0, 60.3, 45.4, 24.9, 23.8, 21.6.

HRMS (ESI): m/z calculated for $C_{18}H_{21}NO_3S_2$ $[M+Na]^+$, 386.0855; found, 386.0853.

b Synthesis of sulfone

To a reaction tube equipped with a stirring bar, were added **3** (0.5 mmol, 1 equiv), *m*-CPBA (1.1 mmol, 2.2 equiv) and DCM (5 mL, 0.1 M). The resulting mixture was stirred at room temperature for 12 h. Then the reaction was quenched with 1.0 M NaOH (10 mL). The aqueous layer was extracted with DCM for 3 times. The combined organic layer was dried over anhydrous Na_2SO_4 and concentrated and subjected to flash column chromatography (100:1 Dichloromethane / Ethyl acetate) to afford the desired product (169 mg, 90% yield) as a white solid.

Rf = 0.80 (20:1 Dichloromethane / Ethyl acetate)

^{1}H NMR (400 MHz, $CDCl_3$) δ 7.82 (d, *J* = 7.8 Hz, 2H), 7.68 (t, *J* = 7.4 Hz, 1H), 7.62 – 7.53 (m, 4H), 7.30 (d, *J* = 8.0 Hz, 2H), 3.91 – 3.82 (m, 2H), 2.81 (tt, *J* = 12.0, 3.6 Hz, 1H), 2.42 (s, 3H), 2.26 (td, *J* = 12.0, 2.1 Hz, 2H), 2.08 (d, *J* = 12.3 Hz, 2H), 1.74 (dd, *J* = 12.5, 4.0 Hz, 2H).

^{13}C NMR (126 MHz, $CDCl_3$) δ 144.1, 136.3, 134.3, 133.0, 129.9, 129.4, 129.3, 127.7, 60.9, 45.1, 25.1, 21.7.

HRMS (ESI): m/z calculated for $C_{18}H_{21}NO_4S_2$ $[M+ Na]^+$, 402.0804; found, 402.0799.

Spectra Data of substrates and products

Compound SI-1-2

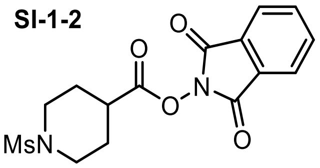

Prepared from 1-(methylsulfonyl)piperidine-4-carboxylic acid (3.1 g, 15 mmol, 1.0 equiv) according to the general procedure 1. Purification by flash chromatography on silica gel using PE/EA (2/1) afforded **SI-1-2** as a white solid (3.4 g, 64% yield).

Rf = 0.12 (Petroleum ether/Ethyl acetate = 2/1)

^{1}H NMR (500 MHz, $CDCl_3$) δ 7.90 – 7.88 (m, 2H), 7.81 – 7.79 (m, 2H), 3.63 – 3.57 (m, 2H), 3.12 (t, *J* = 10.2 Hz, 2H), 2.99 – 2.93 (m, 1H), 2.81 (s, 3H), 2.13 (dd, *J* = 21.4, 12.8 Hz, 4H).

^{13}C NMR (126 MHz, $CDCl_3$) δ 170.3, 162.0, 135.1, 129.0, 124.2, 44.4, 37.3, 35.2, 27.5.

HRMS (ESI): m/z calculated for $C_{15}H_{16}N_2O_6SNa^+$ $[M+Na]^+$, 375.0621; found, 375.0620.

Compound SI-1-5

SI-1-5

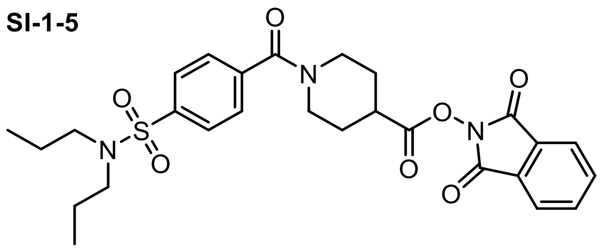

Prepared from 1-(4-(*N*,*N*-dipropylsulfamoyl)benzoyl)piperidine-4-carboxylic acid (2.0 g, 5.0 mmol, 1.0 equiv) according to the general procedure 1. Purification by flash chromatography on silica gel using PE/EA (2/1) afforded **SI-1-36** as a white solid (2.0 g, 74% yield).

Rf = 0.11 (Petroleum ether/Ethyl acetate = 2/1)

^{1}H NMR (500 MHz, $CDCl_3$) δ 7.89 (s, 2H), 7.86 (d, *J* = 7.8 Hz, 2H), 7.81 (s, 2H), 7.52 (d, *J* = 7.4 Hz, 2H), 4.44 (brs, 1H), 3.68 (brs, 1H), 3.28 (brs, 2H), 3.08 (t, *J* = 5.7 Hz, 5H), 2.06 (t, *J* = 78.8 Hz, 4H), 1.56 (q, *J* = 6.3 Hz, 4H), 0.88 (t, *J* = 6.5 Hz, 6H).

^{13}C NMR (126 MHz, $CDCl_3$) δ 170.3, 169.0, 162.0, 141.6, 139.6, 135.0, 129.0, 127.6, 127.6, 124.2, 50.3, 46.5, 41.1, 38.4, 28.5, 27.6, 22.2, 11.3.

HRMS (ESI): m/z calculated for $C_{27}H_{32}N_3O_7S^+$ $[M+H]^+$, 542.1955; found, 542.1951.

Compound SI-1-6

SI-1-6

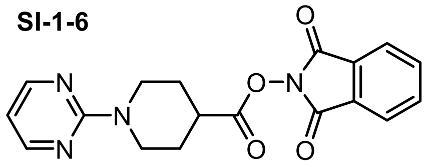

Prepared from 1-(pyrimidin-2-yl)piperidine-4-carboxylic acid (1.0 g, 5 mmol, 1.0 equiv) according to the general procedure 1. Purification by flash chromatography on silica gel using PE/EA (5/1) afforded SI-1-6 as a white solid (0.5 g, 28% yield).

Rf = 0.18 (Petroleum ether/Ethyl acetate = 5/1)

^{1}H NMR (500 MHz, $CDCl_3$) δ 8.31 (d, *J* = 4.7 Hz, 2H), 7.94 – 7.84 (m, 2H), 7.83 – 7.72 (m, 2H), 6.49 (t, *J* = 4.7 Hz, 1H), 4.66 (dt, *J* = 13.3, 3.5 Hz, 2H), 3.30 – 3.15 (m, 2H), 3.03 (ddd, *J* = 14.3, 10.3, 3.7 Hz, 1H), 2.21 – 2.11 (m, 2H), 2.02 – 1.83 (m, 2H).

^{13}C NMR (126 MHz, $CDCl_3$) δ 171.0, 162.1, 161.6, 157.9, 134.9, 129.1, 124.1, 110.1, 42.92, 39.12, 27.8.

HRMS (ESI): m/z calculated for $C_{18}H_{17}N_4O_4^+$ [M+H] $^+$, 353.1244; found, 353.1245.

Compound SI-1-9

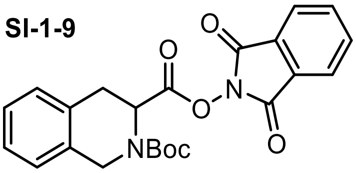

Prepared from 2-(*tert*-butoxycarbonyl)-1,2,3,4-tetrahydroisoquinoline-3-carboxylic acid (1.4 g, 5 mmol, 1.0 equiv) according to the general procedure 1. Purification by flash chromatography on silica gel using PE/EA (10/1) afforded SI-1-9 as a white solid (1.5 g, 70% yield).

Rf = 0.30 (Petroleum ether = 1/4)

^1H NMR (400 MHz, $CDCl_3$) δ 7.86 – 7.79 (m, 2H), 7.78 – 7.71 (m, 2H), 7.32 – 7.19 (m, 4H), 5.10 (t, *J* = 5.7 Hz, 1H), 4.83 – 4.49 (m, 2H), 3.41 – 3.32 (m, 2H), 1.55 (s, 9H).

HRMS (ESI): m/z calculated for $C_{23}H_{22}N_2O_6Na^+$ [M +Na]$^+$, 445.1370; found, 445.1362.

Compound SI-1-17

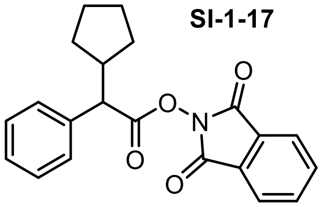

Prepared from 2-cyclopentyl-2-phenylacetic acid (1.0 g, 5 mmol, 1.0 equiv) according to the general procedure 1. Purification by flash chromatography on silica gel using PE/EA (10/1) afforded SI-1-17 as a white solid (1.2 g, 69% yield).

Rf = 0.48 (Petroleum ether/Ethyl acetate = 5/1)

^1H NMR (400 MHz, $CDCl_3$) δ 7.85 (dd, *J* = 5.2, 3.1 Hz, 2H), 7.76 (dd, *J* = 5.5, 3.1 Hz, 2H), 7.44 – 7.28 (m, 5H), 3.68 (d, *J* = 10.8 Hz, 1H), 2.62 (dq, *J* = 17.6, 8.5 Hz, 1H), 2.12 (td, *J* = 12.1, 7.3 Hz, 1H), 1.81 – 1.45 (m, 6H), 1.22 – 1.10 (m, 1H).

^{13}C NMR (101 MHz, $CDCl_3$) δ 170.2, 162.0, 136.9, 134.8, 129.1, 128.9, 128.5, 128.0, 124.0, 54.7, 44.0, 31.5, 31.0, 25.3, 24.9.

HRMS (ESI): m/z calculated for $C_{21}H_{19}NO_4Na^+$ [M +Na] $^+$, 372.1206; found, 372.1206.

Compound SI-1-18

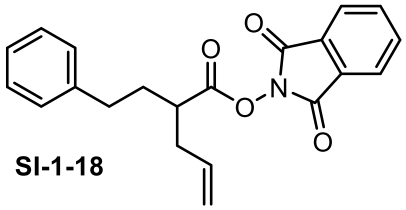

SI-1-18

Prepared from 2-phenethylpent-4-enoic acid (580 mg, 2 mmol, 1.0 equiv) according to the general procedure 1. Purification by flash chromatography on silica gel using PE/EA (5/1) afforded SI-1-18 as a white solid (705 mg, 71% yield).

Rf = 0.52 (Petroleum ether/Ethyl acetate = 5/1)

^1H NMR (500 MHz, Chloroform-*d*) δ 7.95 – 7.86 (m, 2H), 7.86 – 7.76 (m, 2H), 7.34 – 7.25 (m, 4H), 7.21 (t, *J* = 7.2 Hz, 1H), 5.94 – 5.76 (m, 1H), 5.25 – 5.12 (m, 2H), 2.93 – 2.80 (m, 2H), 2.74 (dt, *J* = 14.3, 8.1 Hz, 1H), 2.58 (dt, *J* = 14.5, 7.3 Hz, 1H), 2.43 (dt, *J* = 14.1, 6.8 Hz, 1H), 2.11 (tdd, *J* = 10.6, 7.6, 3.1 Hz, 1H), 2.03 – 1.93 (m, 1H).

^{13}C NMR (126 MHz, $CDCl_3$) δ 171.7, 162.1, 141.2, 134.9, 133.9, 129.1, 128.7, 128.6, 126.2, 124.1, 118.4, 42.4, 36.3, 33.6, 33.1.

HRMS (ESI): m/z calculated for $C_{21}H_{19}NO_4Na^+$ $[M+Na]^+$, 372.1206; found, 372.1205.

Compound SI-1-19

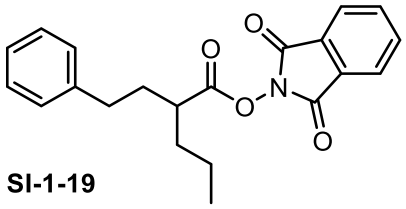

SI-1-19

Prepared from 2-phenethylpentanoic acid acid (420 mg, 2 mmol, 1.0 equiv) according to the general procedure 1. Purification by flash chromatography on silica gel using PE/EA (5/1) afforded SI-1-17 as a white solid (625 mg, 69% yield).

Rf = 0.55 (Petroleum ether/Ethyl acetate = 5/1)

^{1}H NMR (400 MHz, $CDCl_3$) δ 7.90 (dd, *J* = 5.4, 3.1 Hz, 2H), 7.80 (dd, *J* = 5.4, 3.1 Hz, 2H), 7.34 – 7.25 (m, 4H), 7.21 (t, *J* = 6.9 Hz, 1H), 2.87 (ddd, *J* = 14.6, 9.9, 5.2 Hz, 1H), 2.81 – 2.68 (m, 2H), 2.12 (dtd, *J* = 14.5, 9.5, 5.2 Hz, 1H), 1.95 (ddt, *J* = 14.1, 6.8, 3.8 Hz, 1H), 1.82 (dtd, *J* = 13.6, 9.4, 5.1 Hz, 1H), 1.67 – 1.59 (m, 2H), 1.52 – 1.40 (m, 1H), 0.96 (t, *J* = 7.2 Hz, 3H).

^{13}C NMR (101 MHz, $CDCl_3$) δ 172.5, 162.2, 141.4, 134.9, 129.1, 128.7, 128.6, 126.2, 124.1, 42.6, 34.7, 34.4, 33.3, 20.4, 14.0.

HRMS (ESI): m/z calculated for $C_{21}H_{21}NO_4Na^+$ $[M+Na]^+$, 374.1363; found, 374.1355.

Compound SI-1-20

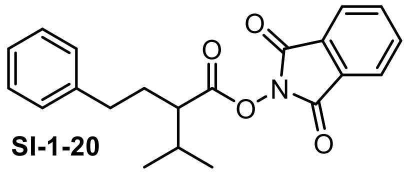

Prepared from 2-isopropyl-4-phenylbutanoic acid (551 mg, 2.7 mmol, 1.0 equiv) according to the general procedure 1. Purification by flash chromatography on silica gel using PE/EA (5/1) afforded SI-1-20 as a white solid (488 mg, 52% yield).

Rf = 0.54 (Petroleum ether/Ethyl acetate = 5/1)

^{1}H NMR (400 MHz, $CDCl_3$) δ 7.90 (dt, *J* = 7.3, 3.7 Hz, 2H), 7.80 (dd, *J* = 5.4, 3.1 Hz, 2H), 7.34 – 7.26 (m, 4H), 7.24 – 7.18 (m, 1H), 2.90 (td, *J* = 9.7, 4.9 Hz, 1H), 2.73 – 2.62 (m, 1H), 2.54 (ddd, *J* = 11.0, 7.2, 3.8 Hz, 1H), 2.12 – 2.03 (m, 2H), 2.02 – 1.92 (m, 1H), 1.08 (dd, *J* = 11.4, 6.8 Hz, 6H).

^{13}C NMR (101 MHz, $CDCl_3$) δ 171.8, 162.3, 141.5, 134.9, 129.2, 128.7, 128.6, 126.2, 124.1, 49.7, 33.6, 31.9, 31.0, 20.4, 20.2.

HRMS (ESI): m/z calculated for $C_{21}H_{21}NO_4Na^+$ $[M +Na]^+$, 374.1363; found, 374.1360.

Compound SI-1-24

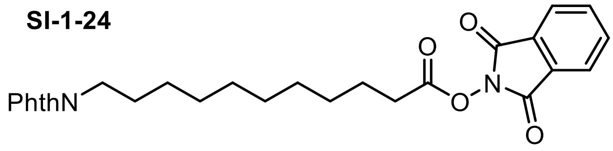

Prepared from 11-(1,3-dioxoisoindolin-2-yl)undecanoic acid (1.7 g, 5.0 mmol, 1.0 equiv) according to the general procedure 1. Purification by flash chromatography on silica gel using PE/EA (5/1) afforded SI-1-20 as a white solid (1.7 g, 71% yield).

Rf = 0.24 (Petroleum ether/Ethyl acetate = 5/1)

^{1}H NMR (500 MHz, $CDCl_3$) δ 7.88 (dt, *J* = 8.3, 4.2 Hz, 2H), 7.83 (dt, *J* = 8.3, 4.2 Hz, 2H), 7.80 – 7.76 (m, 2H), 7.73 – 7.65 (m, 2H), 3.72 – 3.63 (m, 2H), 2.70 – 2.60 (m, 2H), 1.82 – 1.62 (m, 4H), 1.46 – 1.26 (m, 12H).

^{13}C NMR (126 MHz, $CDCl_3$) δ 169.8, 168.6, 162.1, 134.9, 134.0, 132.3, 129.1, 124.1, 123.3, 38.21, 31.1, 29.5, 29.4, 29.3, 29.2, 28.9, 28.7, 27.0, 24.8.

HRMS (ESI): m/z calculated for $C_{27}H_{28}N_2O_6Na^+$ $[M+Na]^+$, 499.1840; found, 499.1840.

Compound SI-1-27

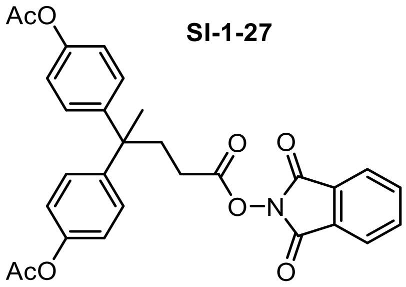

Prepared from 4,4-bis(4-acetoxyphenyl)pentanoic acid (1.8 g, 4.9 mmol, 1.0 equiv) according to the general procedure 1. Purification by flash chromatography on silica gel using PE/EA (2/1) afforded SI-1-27 as a white solid (1.5 g, 60% yield).

Rf = 0.11 (2:1 Petroleum ether/Ethyl acetate = 2/1)

^{1}H NMR (400 MHz, $CDCl_3$) δ 7.91 – 7.83 (m, 2H), 7.83 – 7.74 (m, 2H), 7.21 (d, *J* = 8.4 Hz, 4H), 7.03 (d, *J* = 8.3 Hz, 4H), 2.63 – 2.53 (m, 2H), 2.50 – 2.40 (m, 2H), 2.29 (s, 6H), 1.67 (s, 3H).

^{13}C NMR (126 MHz, $CDCl_3$) δ 169.9, 169.5, 162.0, 149.1, 145.3, 134.9, 129.0, 128.3, 124.1, 121.4, 45.4, 36.3, 27.8, 27.3, 21.3.

HRMS (ESI): m/z calculated for $C_{29}H_{25}NO_8Na^+$ $[M+Na]^+$, 538.1472; found, 538.1490.

Compound SI-1-32

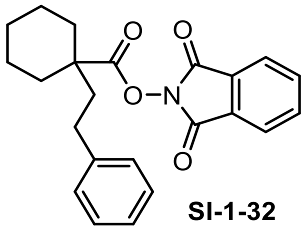

SI-1-32

Prepared from 1-phenethylcyclohexane-1-carboxylic acid (2.3 g, 10.0 mmol, 1.0 equiv) according to the general procedure 1. Purification by flash chromatography on silica gel using PE/EA (2/1) afforded SI-1-27 as a white solid (2.6 g, 70% yield).

1H NMR (400 MHz, $CDCl_3$) δ = 7.90 (dd, J=5.5, 3.2, 2H), 7.79 (dd, J=5.5, 3.1, 2H), 7.31 (d, J=4.4, 4H), 7.22 – 7.17 (m, 1H), 2.79 – 2.72 (m, 2H), 2.40 – 2.32 (m, 2H), 2.03 – 1.96 (m, 2H), 1.76 – 1.58 (m, 6H), 1.46 – 1.37 (m, 2H).

^{13}C NMR (126 MHz, $CDCl_3$) δ 172.8, 162.4, 142.2, 134.8, 129.3, 128.7, 128.6, 126.0, 124.0, 47.4, 43.4, 34.6, 30.6, 25.9, 23.2.

HRMS (ESI): m/z calculated for $C_{23}H_{23}NO_4Na^+$ $[M+Na]^+$, 400.1519; found, 400.1526.

Compound SI-1-35

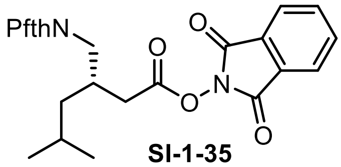

SI-1-35

Prepared from (S)-3-((1,3-dioxoisoindolin-2-yl)methyl)-5-methylhexanoic acid (1.4 g, 5.0 mmol, 1.0 equiv) according to the general procedure 1. Purification by flash chromatography on silica gel using PE/EA (5/1) afforded SI-1-35 as a white solid (0.74 g, 34% yield).

Rf = 0.23 (Petroleum ether/Ethyl acetate = 5/1)

^{1}H NMR (400 MHz, $CDCl_3$) δ 7.93 – 7.82 (m, 4H), 7.79- 7.70 (m, 4H), 3.84 – 3.66 (m, 2H), 2.80 – 2.57 (m, 2H), 2.58 – 2.50 (m, 1H), 1.83 (dq, *J* = 13.4, 6.7 Hz, 1H), 1.45 – 1.29 (m, 2H), 1.00 (d, *J* = 6.5 Hz, 3H), 0.95 (d, *J* = 6.5 Hz, 3H).

^{13}C NMR (101 MHz, $CDCl_3$) δ 168.8, 168.7, 162.0, 134.8, 134.2, 132.1, 129.1, 124.1, 123.6, 41.6, 41.3, 34.7, 33.3, 25.4, 23.1, 22.3.

HRMS (ESI): m/z calculated for $C_{24}H_{22}N_2O_6Na^+$ $[M+Na]^+$, 457.1370; found,

457.1373.

Compound SI-1-38

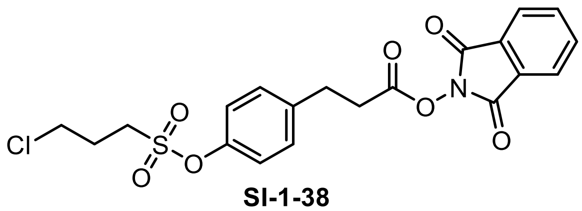

SI-1-38

Prepared from 3-(4-(((3-chloropropyl)sulfonyl)oxy)phenyl)propanoic acid (1.0 g, 3.3 mmol, 1.1 equiv) according to the general procedure 1. Purification by flash chromatography on silica gel using DCM afforded SI-1-38 as a white solid (1.2 g, 86% yield).

^{1}H NMR (400 MHz, $CDCl_3$) δ 7.89 (dd, *J* = 5.5, 3.1 Hz, 2H), 7.80 (dd, *J* = 5.5, 3.1 Hz, 2H), 7.35 – 7.30 (m, 2H), 7.28 – 7.23 (m, 2H), 3.73 (t, *J* = 6.1 Hz, 2H), 3.49 – 3.42 (m, 2H), 3.12 (t, *J* = 7.6 Hz, 2H), 2.99 (t, *J* = 7.4 Hz, 2H), 2.45 (dq, *J* = 7.2, 6.0 Hz, 2H).

^{13}C NMR (101 MHz, $CDCl_3$) δ 168.76, 161.97, 147.92, 138.67, 134.97, 130.15, 128.96, 124.16, 122.35, 47.71, 42.47, 32.68, 30.03, 26.89.

Compound SI-1-39

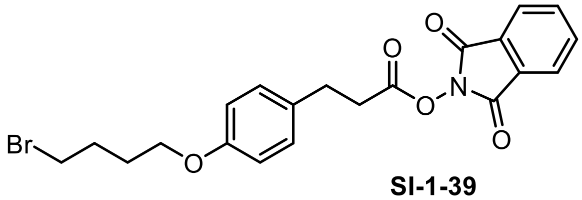

SI-1-39

Prepared from (S)-3-((1,3-dioxoisoindolin-2-yl)methyl)-5-methylhexanoic acid (0.94 g, 3.3 mmol, 1.1 equiv) according to the general procedure 1. Purification by flash chromatography on silica gel using DCM afforded SI-1-39 as a white solid (1.2 g, 90% yield).

^{1}H NMR (400 MHz, $CDCl_3$) δ 7.88 (td, *J* = 5.3, 3.3 Hz, 2H), 7.83 – 7.76 (m, 2H), 7.20 – 7.14 (m, 2H), 6.88 – 6.82 (m, 2H), 3.98 (td, *J* = 6.1, 1.5 Hz, 2H), 3.49 (td, *J* = 6.7, 1.5 Hz, 2H), 2.07 (dtd, *J* = 8.8, 6.9, 5.4 Hz, 2H), 1.93 (tt, *J* = 7.2, 5.9 Hz, 2H).

^{13}C NMR (101 MHz, $CDCl_3$) δ 169.05, 162.05, 157.78, 134.91, 131.48, 129.46, 129.03, 124.12, 114.80, 66.96, 33.66, 33.16, 29.89, 29.62, 28.05.

Compound SI-4-10

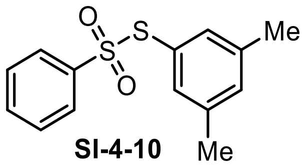

SI-4-10

Prepared from SI-3-11 (274 mg, 1.0 mmol, 1.0 equiv) and sodium benzensulfinate (1051 mg, 6.4 mmol, 3.2 equiv) according to the general procedure 3. Purification by flash chromatography on silica gel using PE/EA (20/1) afforded SI-4-10 as a white solid (489 mg, 88% yield).

Rf = 0.38 (Petroleum ether/Ethyl acetate = 10/1)

^{1}H NMR (400 MHz, $CDCl_3$) δ 7.65 – 7.54 (m, 3H), 7.49 – 7.38 (m, 2H), 7.08 (s, 1H), 6.93 (s, 2H), 2.24 (s, 6H).

^{13}C NMR (101 MHz, $CDCl_3$) δ 143.1, 139.3, 134.3, 133.6, 133.3, 128.8, 127.9, 127.2, 21.2.

HRMS (ESI): m/z calculated for $C_{14}H_{14}O_2S_2Na^+$ [M +Na]$^+$, 301.0327; found, 301.0326.

Compound SI-6-2

SI-6-2

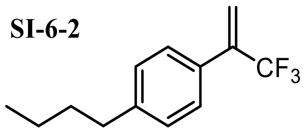

Prepared from (4-butylphenyl)boronic acid (890 mg, 5.0 mmol, 1.0 equiv) and 2-bromo-3,3,3-trifluoroprop-1-ene (1750 mg, 10.0 mmol, 2 equiv) according to the general procedure 4. Purification by flash chromatography on silica gel using PE afforded SI-6-2 as a colorless liquid (900 mg, 79% yield).

Rf = 0.90 (Petroleum)

^{1}H NMR (400 MHz, $CDCl_3$) δ 7.37 (d, *J* = 8.0 Hz, 2H), 7.23 – 7.17 (m, 2H), 5.91 (q, *J* = 1.4 Hz, 1H), 5.75 (q, *J* = 1.7 Hz, 1H), 2.68 – 2.59 (m, 2H), 1.67 – 1.56 (m, 2H), 1.43 – 1.32 (m, 2H), 0.94 (t, *J* = 7.3 Hz, 3H).

^{13}C NMR (126 MHz, $CDCl_3$) δ 144.10, 138.95 (q, *J* = 29.9 Hz), 131.03, 128.74, 127.33, 123.58 (q, *J* = 274.0 Hz), 119.72 (q, *J* = 5.8 Hz), 35.47, 33.59, 22.50, 14.08.

^{19}F NMR (471 MHz, $CDCl_3$) δ -64.73.

Compound SI-6-12

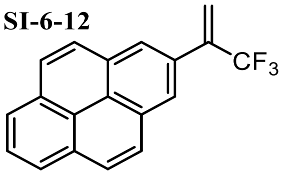

Prepared from (4-butylphenyl)boronic acid (960 mg, 3.9 mmol, 1.0 equiv) and 2-bromo-3,3,3-trifluoroprop-1-ene (1360 mg, 7.8 mmol, 2 equiv) according to the general procedure 4. Purification by flash chromatography on silica gel using PE afforded SI-6-12 as a solid (900 mg, 79% yield).

Rf = 0.70 (Petroleum)

^{1}H NMR (400 MHz, $CDCl_3$) δ 8.25 – 8.16 (m, 4H), 8.15 – 8.08 (m, 3H), 8.08 – 8.01 (m, 1H), 7.98 – 7.93 (m, 1H), 6.48 (q, *J* = 1.4 Hz, 1H), 5.79 (q, *J* = 1.4 Hz, 1H).

^{13}C NMR (101 MHz, $CDCl_3$) δ137.85 (q, *J* = 31.4 Hz), 131.79, 131.39, 130.90, 130.03, 128.78, 128.32, 127.35, 127.24, 126.36, 125.72, 125.52, 124.91, 124.75 (q, *J* = 5.1 Hz), 124.56, 124.37, 123.43 (q, *J* = 277.9 Hz).

^{19}F NMR (376 MHz, $CDCl_3$) δ -66.57.

Compound SI-6-14

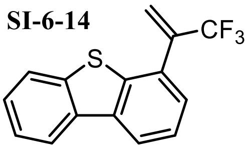

Prepared from dibenzo[*b,d*]thiophen-4-ylboronic acid (684 mg, 3.0 mmol, 1.0 equiv) and 2-bromo-3,3,3-trifluoroprop-1-ene (1044 mg, 6.0 mmol, 2 equiv) according to the general procedure 4. Purification by flash chromatography on silica gel using PE afforded SI-6-2 as a white solid (680 mg, 82% yield).

Rf = 0.90 (Petroleum)

^{1}H NMR (400 MHz, $CDCl_3$) δ 8.20 – 8.14 (m, 2H), 7.88 – 7.82 (m, 1H), 7.53 – 7.44 (m, 4H), 6.34 (q, *J* = 1.5 Hz, 1H), 6.07 (q, *J* = 1.5 Hz, 1H).

^{13}C NMR (126 MHz, $CDCl_3$) δ 140.18, 139.31, 137.87 (q, *J* = 31.4 Hz), 136.51, 135.79, 128.77, 127.25, 126.60, 124.75, 124.71, 123.77 (q, *J* = 5.4 Hz), 123.08 (q, *J* = 274.1 Hz)， 122.79, 122.12, 121.89.

^{19}F NMR (471 MHz, $CDCl_3$) δ -65.92.

4-(phenylthio)-1-tosylpiperidine (3)

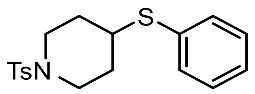

Prepared from **SI-1-1** (0.3 mmol, 1.5 equiv) and **SI-3-1**(0.2 mmol, 1.0 equiv) according to the general procedure 5. Purification by flash column (EtOAc/Petroleum Ether = 1/15) afforded **3** (56 mg, 81% yield) as a white solid.

Prepared from **SI-1-1** (0.3 mmol, 1.5 equiv) and **SI-3-1**(0.2 mmol, 1.0 equiv) according to the general procedure 5 (without 2,2':6',2''-terpyridine). Purification by flash column (EtOAc/Petroleum Ether = 1/15) afforded **3** (42 mg, 60% yield) as a white solid.

Prepared from 4-iodo-1-tosylpiperidine (**1-I**, 0.2 mmol, 1.0 equiv) and **SI-3-1** (0.2 mmol, 1.0 equiv) according to the general procedure 5. Purification by flash column (EtOAc/Petroleum Ether = 1/50) afforded **3** (44 mg, 63% yield) as a white solid.

Prepared from 4-bromo-1-tosylpiperidine (**1-Br,** 0.2 mmol, 1.0 equiv) and **SI-3-1**(0.2 mmol, 1.0 equiv) according to the general procedure 5. Purification by flash column (EtOAc/Petroleum Ether = 1/50) afforded **3** (49 mg, 71% yield) as a white solid.

Prepared from **SI-2-1** (0.1 mmol, 1.0 equiv) and **SI-4-1**(0.25 mmol, 2.5 equiv) according to the general procedure 6. Purification by flash column (EtOAc/Petroleum Ether = 1/15) afforded **3** (32 mg, 93% yield) as a white solid.

Rf = 0.30 (EtOAc/Petroleum Ether = 1/10)

^{1}H NMR (400 MHz, $CDCl_3$) δ 7.62 (d, *J* = 8.2 Hz, 2H), 7.38 – 7.33 (m, 2H), 7.31 (d, *J* = 8.1 Hz, 2H), 7.30 – 7.21 (m, 3H), 3.58 (dt, *J* = 9.8, 4.0 Hz, 2H), 3.00 (ddd, *J* = 13.7, 9.8, 3.8 Hz, 1H), 2.60 – 2.50 (m, 2H), 2.43 (s, 3H), 2.05 – 1.94 (m, 2H), 1.70 (dtd, *J* = 13.7, 10.1, 3.8 Hz, 2H).

^{13}C NMR (126 MHz, $CDCl_3$) δ 143.7, 133.4, 133.1, 132.8, 129.8, 129.1, 127.7, 127.6, 45.5, 43.5, 31.5, 21.6.

HRMS (ESI): m/z calculated for $C_{18}H_{22}NO_2S_2^+$ $[M+H]^+$, 348.1086; found, 348.1075.

4-((4-fluorophenyl)thio)-1-tosylpiperidine (6)

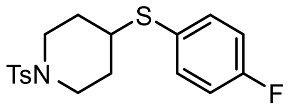

Prepared from **SI-1-1** (0.3 mmol, 1.5 equiv) and **SI-3-2** (0.2 mmol, 1.0 equiv) according to the general procedure 5. Purification by flash column (EtOAc/Petroleum Ether = 1/15) afforded **3** (49 mg, 67% yield) as a white solid.

Prepared from **SI-2-1** (0.1 mmol, 1.0 equiv) and **SI-4-2**(0.25 mmol, 2.5 equiv) according to the general procedure 6. Purification by flash column (EtOAc/Petroleum Ether = 1/15) afforded **6** (26 mg, 69% yield) as a white solid.

Rf = 0.28 (EtOAc/Petroleum Ether = 1/10)

^{1}H NMR (400 MHz, $CDCl_3$) δ = 7.61 (d, J=8.3, 2H), 7.35 (dd, J=8.8, 5.3, 2H), 7.31 (d, J=8.1, 2H), 6.97 (t, J=8.6, 2H), 3.63 – 3.56 (m, 2H), 2.91 – 2.84 (m, 1H), 2.54 – 2.47 (m, 2H), 2.43 (s, 3H), 1.99 – 1.92 (m, 2H), 1.71 – 1.61 (m, 2H).

^{13}C NMR (126 MHz, $CDCl_3$) δ 163.8, 161.8, 143.7, 136.0, 135.9, 133.5, 129.8, 128.4, 128.3, 127.8, 116.3, 116.2, 45.6, 44.5, 31.6, 21.6.

^{19}F NMR (471 MHz, CDCl3) δ -113.394.

HRMS (APCI): m/z calculated for $C_{18}H_{21}FNO_2S_2^+$ $[M+H]^+$, 366.0992; found, 366.0990.

4-((4-chlorophenyl)thio)-1-tosylpiperidine (7)

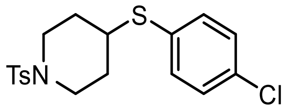

Prepared from **SI-1-1** (0.3 mmol, 1.5 equiv) and **SI-3-3** (0.2 mmol, 1.0 equiv) according to the general procedure 5. Purification by flash column (EtOAc/Petroleum Ether = 1/15) afforded **7** (64 mg, 84% yield) as a white solid.

Prepared from **SI-2-1** (0.1 mmol, 1.0 equiv) and **SI-4-3**(0.25 mmol, 2.5 equiv) according to the general procedure 6. Purification by flash column (EtOAc/Petroleum Ether = 1/15) afforded **7** (29 mg, 77% yield) as a white solid.

Rf = 0.29 (EtOAc/Petroleum Ether = 1/10)

^{1}H NMR (400 MHz, $CDCl_3$) δ = 7.62 (d, *J* =8.4, 2H), 7.31 (d, *J* =8.0, 2H), 7.28 (d, *J* =8.7, 2H), 7.24 (d, *J* =8.8, 2H), 3.66 – 3.54 (m, 2H), 2.99 – 2.92 (m, 1H), 2.57 – 2.49 (m, 2H), 2.43 (s, 3H), 1.98 (dd, *J* =13.6, 3.9, 2H), 1.73 – 1.63 (m, 2H).

^{13}C NMR (101 MHz, CDCl$_3$) δ 143.8, 134.3, 133.9, 133.2, 131.9, 129.8, 129.3, 127.8, 47.1, 45.6, 43.9, 31.5, 25.3, 21.7.

HRMS (ESI): m/z calculated for $C_{18}H_{21}ClNO_2S_2^+$ [M+H]$^+$, 382.0697; found, 382.0713.

4-((4-bromophenyl)thio)-1-tosylpiperidine (8)

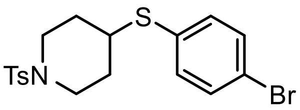

Prepared from **SI-1-1** (0.3 mmol, 1.5 equiv) and **SI-3-4** (0.2 mmol, 1.0 equiv) according to the general procedure 5. Purification by flash column (EtOAc/Petroleum Ether = 1/15) afforded **8** (83 mg, 97% yield) as a white solid.

Prepared from **SI-1-1** (0.3 mmol, 1.5 equiv) and **SI-3-4** (0.2 mmol, 1.0 equiv) according to the general procedure 5 (without 2,2':6',2"-terpyridine). Purification by flash column (EtOAc/Petroleum Ether = 1/15) afforded **8** (77 mg, 90% yield) as a white solid.

Prepared from **SI-2-1** (0.1 mmol, 1.0 equiv) and **SI-4-4** (0.25 mmol, 2.5 equiv) according to the general procedure 6. Purification by flash column (EtOAc/Petroleum Ether = 1/15) afforded **8** (36 mg, 84% yield) as a white solid.

Rf = 0.31 (EtOAc/Petroleum Ether = 1/10)

^{1}H NMR (500 MHz, CDCl$_3$) δ = 7.62 (d, *J* =7.4, 2H), 7.39 (d, *J* =7.3, 2H), 7.32 (d, *J* =7.6, 2H), 7.21 (d, *J* =7.4, 2H), 3.63 – 3.53 (m, 2H), 2.97 (t, *J* =8.5, 1H), 2.53 (t, *J* =11.1, 2H), 2.44 (s, 3H), 1.98 (d, *J* =14.0, 2H), 1.69 (q, *J* =10.5, 2H).

^{13}C NMR (126 MHz, CDCl$_3$) δ 143.8, 134.4, 133.2, 132.7, 132.2, 129.8, 127.8, 121.9, 47.1, 45.5, 43.8, 31.51, 25.3, 21.7.

HRMS (ESI): m/z calculated for $C_{18}H_{21}BrNO_2S_2^+$ [M+H]$^+$, 426.0192; found, 426.0205.

1-tosyl-4-((4-(trifluoromethyl)phenyl)thio)piperidine (9)

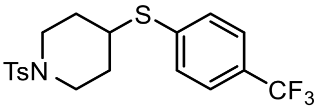

Prepared from **SI-1-1** (0.3 mmol, 1.5 equiv) and **SI-3-5** (0.2 mmol, 1.0 equiv)

according to the general procedure 5. Purification by flash column (EtOAc/Petroleum Ether = 1/15) afforded **9** (54 mg, 65% yield) as a white solid.

Prepared from **SI-2-1** (0.1 mmol, 1.0 equiv) and **SI-4-6**(0.25 mmol, 2.5 equiv) according to the general procedure 6. Purification by flash column (EtOAc/Petroleum Ether = 1/15) afforded **9** (27 mg, 66% yield) as a white solid.

Rf = 0.28 (EtOAc/Petroleum Ether = 1/10)

^{1}H NMR (400 MHz, $CDCl_3$) δ 7.64 (d, *J* = 8.3 Hz, 2H), 7.50 (d, *J* = 8.2 Hz, 2H), 7.39 (d, *J* = 8.1 Hz, 2H), 7.33 (d, *J* = 8.0 Hz, 2H), 3.58 (dt, *J* = 10.0, 4.0 Hz, 2H), 3.20 – 3.11 (m, 1H), 2.67 – 2.55 (m, 2H), 2.44 (s, 3H), 2.10 – 1.97 (m, 2H), 1.80 – 1.69 (m, 2H).

^{13}C NMR (101 MHz, $CDCl_3$) δ 143.8, 139.3, 133.1, 130.9, 129.9, 129.0 (q, *J* = 32.6 Hz), 127.82, 125.9 (q, *J* = 3.5 Hz), 124.1 (q, *J* = 271.7 Hz), 45.5, 42.8, 31.5, 21.7.

^{13}C NMR (101 MHz, CDCl3) δ 129.01 (d, *J* = 32.6 Hz), 125.94 (q, *J* = 3.5 Hz), 124.08 (d, *J* = 271.7 Hz).

^{19}F NMR (376 MHz, CDCl3) δ -62.65 .

HRMS (ESI): m/z calculated for $C_{19}H_{21}F_3NO_2S_2^+$ $[M+H]^+$, 416.0960; found, 416.0952.

4-(*p*-tolylthio)-1-tosylpiperidine (10)

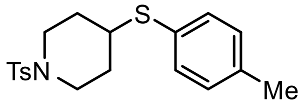

Prepared from **SI-1-1** (0.3 mmol, 1.5 equiv) and **SI-3-6** (0.2 mmol, 1.0 equiv) according to the general procedure 5. Purification by flash column (EtOAc/Petroleum Ether = 1/15) afforded **10** (52 mg, 72% yield) as a white solid.

Prepared from **SI-1-1** (0.3 mmol, 1.5 equiv) and **SI-3-6** (0.2 mmol, 1.0 equiv) according to the general procedure 5 (without ligand). Purification by flash column (EtOAc/Petroleum Ether = 1/15) afforded **10** (43 mg, 60% yield) as a white solid

Rf = 0.31 (EtOAc/Petroleum Ether = 1/10)

^{1}H NMR (400 MHz, $CDCl_3$) δ = 7.62 (d, *J* =8.4, 2H), 7.31 (d, *J* =7.9, 2H), 7.25 (s, 2H), 7.08 (d, *J* =7.8, 2H), 3.66 – 3.49 (m, 2H), 2.97 – 2.89 (m, 1H), 2.51 (t, *J* =9.6,

2H), 2.43 (s, 3H), 2.32 (s, 3H), 1.97 (dd, *J* =13.6, 3.9, 2H), 1.71 – 1.63 (m, 2H).

^{13}C NMR (101 MHz, $CDCl_3$) δ 143.7, 138.0, 133.7, 133.2, 129.9, 129.8, 129.5, 127.8, 45.6, 44.0, 31.6, 21.7, 21.2.

HRMS (ESI): m/z calculated for $C_{19}H_{24}NO_2S_2^+$ $[M+H]^+$, 362.1243; found, 362.1234.

4-((4-methoxyphenyl)thio)-1-tosylpiperidine (11)

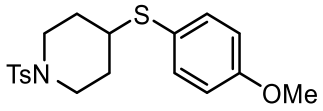

Prepared from **SI-1-1** (0.3 mmol, 1.5 equiv) and **SI-3-7** (0.2 mmol, 1.0 equiv) according to the general procedure 5. Purification by flash column (EtOAc/Petroleum Ether = 1/15) afforded **11** (52 mg, 69% yield) as a white solid.

Prepared from **SI-2-1** (0.1 mmol, 1.0 equiv) and **SI-4-5**(0.25 mmol, 2.5 equiv) according to the general procedure 6. Purification by flash column (EtOAc/Petroleum Ether = 1/15) afforded **11** (33 mg, 87% yield) as a white solid.

Rf = 0.13 (EtOAc/Petroleum Ether = 1/10)

1H NMR (400 MHz, CDCl3) δ = 7.61 (d, *J* =7.9, 2H), 7.31 (t, *J* =8.4, 4H), 6.81 (d, *J* =8.0, 2H), 3.79 (s, 3H), 3.63 – 3.54 (m, 2H), 2.79 (dt, *J* =10.1, 6.2, 1H), 2.48 (d, *J* =10.3, 2H), 2.42 (s, 3H), 1.98 – 1.89 (m, 2H), 1.70 – 1.62 (m, 2H).

^{13}C NMR (101 MHz, $CDCl_3$) δ 160.0, 143.7, 136.3, 133.2, 129.8, 127.8, 123.3, 114.7, 114.5, 55.5, 45.7, 44.7, 31.6, 21.7.

HRMS (ESI): m/z calculated for $C_{19}H_{24}NO_3S_2^+$ $[M+H]^+$, 378.1192; found, 378.1197.

4-((2-fluorophenyl)thio)-1-tosylpiperidine (12)

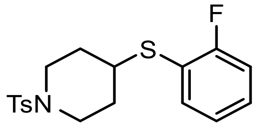

Prepared from **SI-1-1** (0.3 mmol, 1.5 equiv) and **SI-3-8** (0.2 mmol, 1.0 equiv) according to the general procedure 5. Purification by flash column (EtOAc/Petroleum Ether = 1/15) afforded **12** (47 mg, 64% yield) as a white solid.

Prepared from **SI-2-1** (0.1 mmol, 1.0 equiv) and **SI-4-7** (0.25 mmol, 2.5 equiv) according to the general procedure 6. Purification by flash column (EtOAc/Petroleum

Ether = 1/15) afforded **12** (30 mg, 81% yield) as a white solid.

Rf = 0.26 (EtOAc/Petroleum Ether = 1/10)

^{1}H NMR (400 MHz, $CDCl_3$) δ 7.62 (d, *J* = 8.2 Hz, 2H), 7.38 (td, *J* = 7.7, 1.8 Hz, 1H), 7.31 (d, *J* = 7.9 Hz, 2H), 7.27 (dd, *J* = 7.7, 5.4 Hz, 1H), 7.09 – 7.02 (m, 2H), 3.62 – 3.54 (m, 2H), 3.09 – 3.00 (m, 1H), 2.57 – 2.49 (m, 2H), 2.43 (s, 3H), 2.00 – 1.93 (m, 2H), 1.73 – 1.63 (m, 2H).

^{13}C NMR (101 MHz, $CDCl_3$) δ162.8 (d, *J* = 246.2 Hz), 143.7, 136.0, 133.2, 130.3 (d, *J* = 8.0 Hz), 129.8, 127.8, 124.6 (d, *J* = 3.9 Hz), 120.1 (d, *J* = 18.3 Hz), 116.1 (d, *J* = 23.2 Hz), 45.5, 43.0, 31.6, 21.7.

^{19}F NMR (376 MHz, $CDCl_3$) δ -107.25.

HRMS (ESI): m/z calculated for $C_{18}H_{21}FNO_2S_2^+$ $[M+H]^+$, 366.0992; found, 366.0988.

methyl 2-((1-tosylpiperidin-4-yl)thio)benzoate (13)

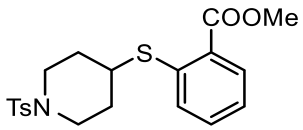

Prepared from **SI-1-1** (0.3 mmol, 1.5 equiv) and **SI-3-10** (0.2 mmol, 1.0 equiv) according to the general procedure 5. Purification by flash column (EtOAc/Petroleum Ether = 1/15) afforded **13** (45 mg, 56% yield) as a white solid.

Prepared from **SI-2-1** (0.1 mmol, 1.0 equiv) and **SI-4-9** (0.25 mmol, 2.5 equiv) according to the general procedure 6. Purification by flash column (EtOAc/Petroleum Ether = 1/15) afforded **13** (24 mg, 60% yield) as a white solid.

Rf = 0.10 (EtOAc/Petroleum Ether = 1/10)

^{1}H NMR (400 MHz, CDCl3) δ 7.83 (dd, *J* = 7.8, 1.3 Hz, 1H), 7.63 (d, *J* = 8.2 Hz, 2H), 7.40 – 7.35 (m, 1H), 7.32 (d, *J* = 8.2 Hz, 2H), 7.29 (d, *J* = 8.5 Hz, 1H), 7.20 (d, *J* = 7.7 Hz, 1H), 3.87 (s, 3H), 3.64 – 3.54 (m, 2H), 3.21 (t, *J* = 3.9 Hz, 1H), 2.68 – 2.58 (m, 2H), 2.43 (s, 3H), 2.13 – 2.04 (m, 2H), 1.82 – 1.71 (m, 2H).

^{13}C NMR (101 MHz, $CDCl_3$) δ 167.3, 143.7, 137.7, 133.3, 132.0, 131.1, 130.9, 129.8, 128.8, 127.8, 125.4, 52.3, 45.6, 41.4, 31.3, 21.6.

HRMS (ESI): m/z calculated for $C_{20}H_{24}NO_4S_2^+$ $[M+H]^+$, 406.1141; found, 406.1121.

4-((2-methoxyphenyl)thio)-1-tosylpiperidine (14)

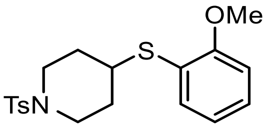

Prepared from **SI-1-1** (0.3 mmol, 1.5 equiv) and **SI-3-9** (0.2 mmol, 1.0 equiv) according to the general procedure 5. Purification by flash column (EtOAc/Petroleum Ether = 1/15) afforded 1**4** (59 mg, 78% yield) as a white solid.

Prepared from **SI-2-1** (0.1 mmol, 1.0 equiv) and **SI-4-8** (0.25 mmol, 2.5 equiv) according to the general procedure 6. Purification by flash column (EtOAc/Petroleum Ether = 1/15) afforded **14** (29 mg, 76% yield) as a white solid.

Rf = 0.11 (EtOAc/Petroleum Ether = 1/10)

^{1}H NMR (400 MHz, CDCl3) δ 7.62 (d, *J* = 8.2 Hz, 2H), 7.31 (d, *J* = 7.2 Hz, 2H), 7.29 – 7.22 (m, 2H), 6.90 – 6.82 (m, 2H), 3.84 (s, 3H), 3.59 – 3.50 (m, 2H), 3.13 (t, *J* = 4.0 Hz, 1H), 2.63 – 2.55 (m, 2H), 2.43 (s, 3H), 2.02 – 1.92 (m, 2H), 1.69 (dtd, *J* = 13.5, 9.8, 3.8 Hz, 2H).

^{1}H NMR (400 MHz, CDCl3) δ 7.84 (dd, *J* = 7.8, 1.6 Hz, 1H), 7.67 – 7.61 (m, 2H), 7.42 – 7.28 (m, 4H), 7.20 (td, *J* = 7.5, 1.2 Hz, 1H), 3.88 (s, 3H), 3.60 (dt, *J* = 10.6, 4.4 Hz, 2H), 3.22 (tt, *J* = 9.8, 3.9 Hz, 1H), 2.69 – 2.59 (m, 2H), 2.44 (s, 3H), 2.14 – 2.03 (m, 2H), 1.84 – 1.71 (m, 2H).

^{13}C NMR (101 MHz, $CDCl_3$) δ 159.0, 143.6, 134.2, 133.4, 129.8, 129.3, 127.8, 121.3, 121.1, 111.1, 55.9, 45.4, 41.4, 31.5, 21.7.

HRMS (ESI): m/z calculated for $C_{19}H_{24}NO_3S_2^+$ $[M+H]^+$, 378.1192; found, 378.1174.

4-((3,5-dimethylphenyl)thio)-1-tosylpiperidine (15)

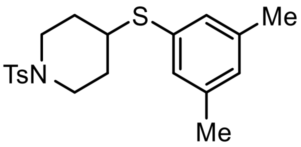

Prepared from **SI-1-1** (0.3 mmol, 1.5 equiv) and **SI-3-11** (0.2 mmol, 1.0 equiv) according to the general procedure 5. Purification by flash column (EtOAc/Petroleum Ether = 1/15) afforded **15** (48 mg, 64% yield) as a white solid.

Prepared from **SI-2-1** (0.1 mmol, 1.0 equiv) and **SI-4-10** (0.25 mmol, 2.5 equiv)

according to the general procedure 6. Purification by flash column (EtOAc/Petroleum Ether = 1/15) afforded **15** (34 mg, 91% yield) as a white solid.

Rf = 0.31 (EtOAc/Petroleum Ether = 1/10)

^{1}H NMR (400 MHz, $CDCl_3$) δ 7.63 (d, *J* = 8.3 Hz, 2H), 7.32 (d, *J* = 8.0 Hz, 2H), 6.97 (s, 2H), 6.87 (s, 1H), 3.57 (dt, *J* = 9.9, 4.1 Hz, 2H), 2.98 (tt, *J* = 9.8, 3.7 Hz, 2H), 2.55 (ddd, *J* = 12.3, 10.3, 3.0 Hz, 2H), 2.43 (s, 3H), 2.26 (s, 6H), 2.00 (dt, *J* = 13.6, 3.9 Hz, 2H), 1.76 – 1.60 (m, 3H).

^{13}C NMR (101 MHz, $CDCl_3$) δ 143.7, 138.7, 133.2, 132.9, 130.5, 129.8, 129.5, 127.8, 45.6, 43.5, 31.6, 21.7, 21.3.

HRMS (ESI): m/z calculated for $C_{20}H_{26}NO_2S_2^+$ [M+H]$^+$, 376.1399; found, 376.1387.

4-((4-bromophenyl)thio)-1-(methylsulfonyl)piperidine (16)

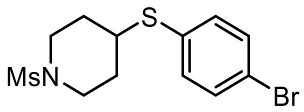

Prepared from **SI-1-2** (0.3 mmol, 1.5 equiv) and **SI-3-4** (0.2 mmol, 1.0 equiv) according to the general procedure 5. Purification by flash column (EtOAc/Petroleum Ether = 1/15) afforded **16** (44 mg, 63% yield) as a white solid.

Rf = 0.42 (EtOAc/Petroleum Ether = 1/10)

^{1}H NMR (500 MHz, $CDCl_3$) δ 7.45 (dd, *J* = 8.1, 2.6 Hz, 2H), 7.30 – 7.26 (m, 2H), 3.63 (dd, *J* = 8.4, 4.1 Hz, 2H), 3.20 (tt, *J* = 9.3, 3.9 Hz, 1H), 2.95 (td, *J* = 9.3, 3.4 Hz, 2H), 2.79 (d, *J* = 3.0 Hz, 3H), 2.09 – 2.02 (m, 2H), 1.73 (qd, *J* = 9.5, 5.8 Hz, 2H).

^{13}C NMR (126 MHz, $CDCl_3$) δ 134.5, 132.6, 132.3, 122.0, 44.9, 43.7, 35.1, 31.5.

HRMS (ESI): m/z calculated for $C_{12}H_{17}BrNO_2S_2^+$ [M+H]$^+$, 349.9879; found, 349.9878.

***tert*-butyl 4-((4-bromophenyl)thio)piperidine-1-carboxylate (17)**

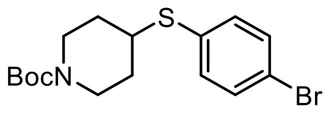

Prepared from **SI-1-3** (0.3 mmol, 1.5 equiv) and **SI-3-4** (0.2 mmol, 1.0 equiv) according to the general procedure 5. Purification by flash column (EtOAc/Petroleum Ether = 1/15) afforded **17** (61 mg, 82% yield) as a white solid.

Prepared from **SI-2-2** (0.1 mmol, 1.0 equiv) and **SI-4-4** (0.25 mmol, 2.5 equiv) according to the general procedure 6. Purification by flash column (EtOAc/Petroleum Ether = 1/15) afforded **17** (29 mg, 78% yield) as a white solid.

Rf = 0.42 (EtOAc/Petroleum Ether = 1/10)

^{1}H NMR (400 MHz, CDCl3) δ 7.42 (d, *J* = 8.3 Hz, 2H), 7.28 (d, *J* = 8.2 Hz, 2H), 3.96 (d, *J* = 10.9 Hz, 2H), 3.21 – 3.14 (m, 1H), 2.91 (t, *J* = 11.0 Hz, 2H), 1.94 – 1.86 (m, 2H), 1.57 – 1.45 (m, 2H), 1.45 (s, 9H).

^{13}C NMR (126 MHz, $CDCl_3$) δ 154.8, 134.3, 133.1, 132.2, 121.6, 79.8, 44.8, 43.5, 32.1, 28.5.

HRMS (EI): m/z calculated for $C_{16}H_{22}BrNO_2S^+$ $[M]^+$, 371.0549; found, 371.0543.

benzyl 4-((4-bromophenyl)thio)piperidine-1-carboxylate (18)

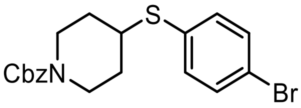

Prepared from **SI-1-4** (0.3 mmol, 1.5 equiv) and **SI-3-4** (0.2 mmol, 1.0 equiv) according to the general procedure 5. Purification by flash column (EtOAc/Petroleum Ether = 1/15) afforded **18** (68 mg, 84% yield) as a white solid.

Rf = 0.42 (EtOAc/Petroleum Ether = 1/10)

1H NMR (500 MHz, CDCl3) δ 7.42 (d, J = 8.5 Hz, 2H), 7.38 – 7.29 (m, 5H), 7.27 (d, J = 8.5 Hz, 2H), 5.12 (s, 2H), 4.04 (s, 2H), 3.25 – 3.14 (m, 1H), 3.01 (s, 2H), 1.97 – 1.87 (m, 2H), 1.54 (s, 2H).

^{13}C NMR (126 MHz, $CDCl_3$) δ 155.2, 136.8, 134.4, 132.9, 132.2, 128.6, 128.2, 128.0, 121.8, 67.3, 44.6, 43.4, 32.0.

HRMS (ESI): m/z calculated for $C_{19}H_{21}BrNO_2S^+$ $[M+H]^+$, 406.0471; found, 406.0466.

3-((4-bromophenyl)thio)-1-tosylpiperidine (19)

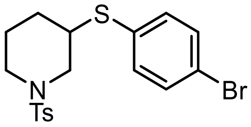

Prepared from **SI-1-7** (0.3 mmol, 1.5 equiv) and **SI-3-4** (0.2 mmol, 1.0 equiv)

according to the general procedure 5. Purification by flash column (EtOAc/Petroleum Ether = 1/15) afforded **19** (46 mg, 89% yield) as a white solid.

Rf = 0.28 (EtOAc/Petroleum Ether = 1/10)

1H NMR (400 MHz, $CDCl_3$) δ = 7.60 (d, J=8.3, 2H), 7.43 (d, J=8.5, 2H), 7.31 (d, J=8.0, 2H), 7.25 (d, J=8.5, 2H), 3.82 – 3.76 (m, 1H), 3.66 – 3.59 (m, 1H), 3.22 – 3.15 (m, 1H), 2.43 (s, 3H), 2.40 – 2.31 (m, 2H), 2.05 – 1.97 (m, 1H), 1.85 – 1.79 (m, 1H), 1.75 – 1.65 (m, 1H), 1.26 (t, J=10.3, 1H).

^{13}C NMR (126 MHz, $CDCl_3$) δ 143.7, 133.9, 133.6, 132.7, 132.4, 129.8, 127.7, 121.8, 51.6, 46.2, 44.0, 30.4, 25.0, 21.7.

HRMS (ESI): m/z calculated for $C_{18}H_{21}BrNO_2S_2^+$ $[M+H]^+$, 426.0192; found, 426.0193.

***tert*-butyl 3-((4-bromophenyl)thio)piperidine-1-carboxylate (20)**

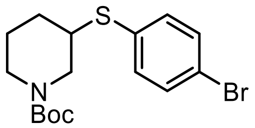

Prepared from **SI-1-8** (0.3 mmol, 1.5 equiv) and **SI-3-4** (0.2 mmol, 1.0 equiv) according to the general procedure 5. Purification by flash column (EtOAc/Petroleum Ether = 1/15) afforded **20** (42 mg, 56% yield) as a white solid.

Rf = 0.42 (EtOAc/Petroleum Ether = 1/10)

^{1}H NMR (400 MHz, CDCl3) δ 7.41 (d, *J* = 8.4 Hz, 2H), 7.27 (d, *J* = 8.5 Hz, 2H), 4.03 (s, 1H), 3.86 (d, *J* = 13.3 Hz, 1H), 3.14 – 3.03 (m, 1H), 2.92 – 2.81 (m, 2H), 2.11 – 2.04 (m, 1H), 1.81 – 1.71 (m, 1H), 1.52 (dt, *J* = 22.1, 11.0 Hz, 2H), 1.41 (s, 9H).

^{13}C NMR (126 MHz, $CDCl_3$) δ 154.6, 133.6, 133.0, 132.2, 121.0, 79.9, 50.0, 44.1, 30.9, 29.8, 28.5, 25.3.

HRMS (ESI): m/z calculated for $C_{16}H_{22}BrNO_2SNa^+$ $[M+Na]^+$, 394.0447; found, 394.0431.

2-(4-((4-bromophenyl)thio)piperidin-1-yl)pyrimidine (21)

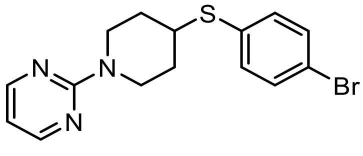

Prepared from **SI-1-6** (0.3 mmol, 1.5 equiv) and **SI-3-4** (0.2 mmol, 1.0 equiv) according to the general procedure 5. Purification by flash column (EtOAc/Petroleum Ether = 1/15) afforded **21** (40 mg, 57% yield) as a white solid.

Rf = 0.42 (EtOAc/Petroleum Ether = 1/10)

^{1}H NMR (500 MHz, $CDCl_3$) δ = 8.28 (s, 2H), 7.42 (d, *J* =7.5, 2H), 7.29 (d, *J* =7.5, 2H), 6.45 (s, 1H), 4.57 (d, *J* =13.1, 2H), 3.31 (t, *J* =11.4, 1H), 3.15 (t, *J* =12.1, 2H), 2.00 (d, *J* =13.2, 2H), 1.59 (q, *J* =10.8, 2H).

^{13}C NMR (126 MHz, $CDCl_3$) δ 161.6, 157.9, 134.3, 133.3, 132.2, 121.5, 109.8, 45.3, 43.5, 32.2.

HRMS (ESI): m/z calculated for $C_{15}H_{17}BrN_3S^+$ $[M+H]^+$, 350.0321; found, 350.0310.

3-((4-bromophenyl)thio)-1-tosylazetidine (22)

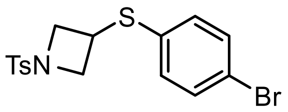

Prepared from **SI-1-10** (0.3 mmol, 1.5 equiv) and **SI-3-4** (0.2 mmol, 1.0 equiv) according to the general procedure 5. Purification by flash column (EtOAc/Petroleum Ether = 1/15) afforded **22** (37 mg, 47% yield) as a white solid.

Prepared from **SI-1-10** (0.3 mmol, 1.5 equiv) and **SI-3-4** (0.2 mmol, 1.0 equiv) according to the general procedure 5 (without 2,2':6',2"-terpyridine). Purification by flash column (EtOAc/Petroleum Ether = 1/15) afforded **22** (36 mg, 45% yield) as a white solid.

Rf = 0.24 (EtOAc/Petroleum Ether = 1/10)

^{1}H NMR (400 MHz, $CDCl_3$) δ 7.67 (d, *J* = 8.3 Hz, 2H), 7.42 – 7.30 (m, 4H), 7.03 (d, *J* = 8.5 Hz, 2H), 4.14 (t, *J* = 8.2 Hz, 2H), 3.91 – 3.78 (m, 1H), 3.64 (dd, *J* = 8.6, 6.4 Hz, 2H), 2.48 (s, 3H).

^{13}C NMR (126 MHz, $CDCl_3$) δ 144.5, 132.6, 132.4, 132.4, 131.5, 130.0, 128.5, 121.9, 57.2, 34.1, 21.9.

HRMS (ESI): m/z calculated for $C_{16}H_{17}BrNO_2S_2^+$ $[M+H]^+$, 397.9879; found, 397.9876.

(4-methoxyphenyl)(2,2,3,3-tetramethylcyclopropyl)sulfane (23)

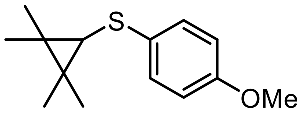

Prepared from **SI-1-11** (0.4 mmol, 2 equiv) and **SI-3-7** (0.2 mmol, 1.0 equiv) according to the general procedure 5. Purification by flash column (EtOAc/Petroleum Ether = 1/100) afforded **23** (18 mg, 38% yield) as a colorless oil.

Rf = 0.40 (Petroleum Ether)

1H NMR (500 MHz, CDCl3) δ 7.18 (dd, J = 8.5, 3.2 Hz, 2H), 6.84 (dd, J = 8.5, 3.1 Hz, 2H), 3.79 (d, J = 3.5 Hz, 3H), 1.79 (d, J = 3.2 Hz, 1H), 1.22 (d, J = 3.2 Hz, 6H), 1.11 (d, J = 3.3 Hz, 6H).

^{13}C NMR (126 MHz, $CDCl_3$) δ 157.6, 129.6, 128.3, 114.6, 55.5, 37.9, 29.9, 25.0, 23.3, 17.7.

HRMS (ESI): m/z calculated for $C_{14}H_{21}OS^+$ $[M+K]^+$, 275.0866; found, 275.0864.

(4-bromophenyl)(cyclobutyl)sulfane (24)

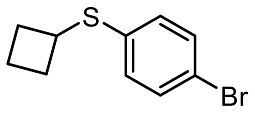

Prepared from **SI-1-12** (0.3 mmol, 1.5 equiv) and **SI-3-4** (0.2 mmol, 1.0 equiv) according to the general procedure 5. Purification by flash column (Petroleum Ether) afforded **24** (39 mg, 81% yield) as a white solid.

Prepared from **SI-1-12** (0.3 mmol, 1.5 equiv) and **SI-3-4** (0.2 mmol, 1.0 equiv) according to the general procedure 5 (without 2,2':6',2"-terpyridine). Purification by flash column (Petroleum Ether) afforded **24** (31 mg, 63% yield) as a white solid.

Rf = 0.72 (Petroleum Ether)

^{1}H NMR (500 MHz, $CDCl_3$) δ = 7.38 (d, *J* =8.3, 2H), 7.10 (d, *J* =8.3, 2H), 3.91 – 3.79 (m, 1H), 2.50 – 2.37 (m, 2H), 2.04 (ddt, *J* =29.8, 17.8, 8.4, 4H).

^{13}C NMR (126 MHz, $CDCl_3$) δ 136.3, 131.9, 130.6, 119.6, 40.3, 30.6, 18.9.

HRMS (EI): m/z calculated for $C_{10}H_{11}BrS^+$ $[M]^+$, 241.9759; found, 241.9758.

(4-bromophenyl)(cyclohexyl)sulfane (25)

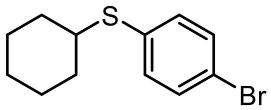

Prepared from **SI-1-13** (0.3 mmol, 1.5 equiv) and **SI-3-4** (0.2 mmol, 1.0 equiv) according to the general procedure 5. Purification by flash column (Petroleum Ether) afforded **25** (47 mg, 87% yield) as a white solid.

Prepared from **SI-2-4** (0.1 mmol, 1.0 equiv) and **SI-4-4** (0.25 mmol, 2.5 equiv) according to the general procedure 6. Purification by flash column (Petroleum Ether) afforded **25** (22 mg, 81% yield) as a white solid.

Rf = 0.77 (Petroleum Ether)

^{1}H NMR (400 MHz, $CDCl_3$) δ = 7.40 (d, *J* =8.5, 2H), 7.25 (d, *J* =8.6, 2H), 3.11 – 3.03 (m, 1H), 2.00 – 1.92 (m, 2H), 1.77 (dd, *J* =9.2, 3.7, 2H), 1.66 – 1.59 (m, 1H), 1.41 – 1.20 (m, 5H).

^{13}C NMR (101 MHz, $CDCl_3$) δ 134.5, 133.5, 131.9, 120.7, 46.9, 33.4, 26.2, 25.8.

HRMS (EI): m/z calculated for $C_{12}H_{15}BrS^+$ $[M]^+$, 270.0072; found, 270.0070.

cycloheptyl(4-methoxyphenyl)sulfane (26)

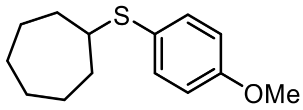

Prepared from **SI-1-15** (0.3 mmol, 1.5 equiv) and **SI-3-7** (0.2 mmol, 1.0 equiv) according to the general procedure 5. Purification by flash column (EtOAc/Petroleum Ether = 1/100) afforded **26** (30 mg, 64% yield) as a white solid.

Rf = 0.22 (Petroleum Ether)

^{1}H NMR (500 MHz, $CDCl_3$) δ 7.37 (d, *J* = 8.8 Hz, 2H), 6.84 (d, *J* = 8.8 Hz, 2H), 3.80 (s, 3H), 3.14 (dt, *J* = 9.3, 4.9 Hz, 1H), 2.01 - 1.93 (m, 2H), 1.74 – 1.66 (m, 2H), 1.58 – 1.50 (m, 6H), 1.46 – 1.37 (m, 2H).

^{13}C NMR (126 MHz, $CDCl_3$) δ 159.6, 135.1, 126.3, 114.5, 55.4, 49.8, 34.8, 28.3, 26.0.

HRMS (ESI): m/z calculated for $C_{14}H_{21}OS^+$ $[M+H]^+$, 237.1308; found, 237.1318.

(4-methoxyphenyl)(1-phenylhexan-3-yl)sulfane (27)

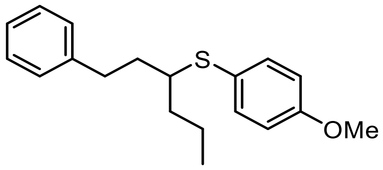

Prepared from **SI-1-19** (0.3 mmol, 1.5 equiv) and **SI-3-7** (0.2 mmol, 1.0 equiv) according to the general procedure 5. Purification by flash column (EtOAc/Petroleum Ether = 1/100) afforded **27** (34 mg, 56% yield) as a white solid.

Rf = 0.12 (Petroleum Ether)

1H NMR (500 MHz, $CDCl_3$) δ 7.47 (d, J = 8.7 Hz, 2H), 7.40 – 7.35 (m, 2H), 7.31 – 7.27 (m, 2H), 6.94 (d, J = 8.7 Hz, 2H), 3.91 (s, 3H), 3.02 - 2.97 (m, 1H), 2.96 – 2.85 (m, 2H), 1.98 – 1.86 (m, 2H), 1.66 – 1.56 (m, 4H), 0.99 (t, J = 6.8 Hz, 3H).

^{13}C NMR (126 MHz, $CDCl_3$) δ 159.4, 142.2, 135.8, 128.6, 128.5, 125.9, 125.0, 114.5, 55.4, 49.6, 36.8, 36.1, 33.1, 20.1, 14.1.

HRMS (ESI): m/z calculated for $C_{19}H_{25}OS^+$ $[M+H]^+$, 301.1621; found, 301.1593.

(4-methoxyphenyl)(1-phenylhex-5-en-3-yl)sulfane (28)

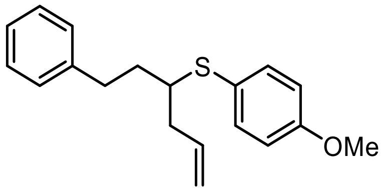

Prepared from **SI-1-18** (0.3 mmol, 1.5 equiv) and **SI-3-7** (0.2 mmol, 1.0 equiv) according to the general procedure 5. Purification by flash column (EtOAc/Petroleum Ether = 1/100) afforded **28** (40 mg, 67% yield) as a white solid.

Rf = 0.14 (Petroleum Ether)

^1H NMR (400 MHz, $CDCl_3$) δ 7.39 (d, *J* = 8.8 Hz, 2H), 7.31 – 7.23 (m, 2H), 7.23 – 7.12 (m, 3H), 6.85 (d, *J* = 8.8 Hz, 2H), 5.95 – 5.79 (m, 1H), 5.11 – 5.03 (m, 2H), 3.81 (s, 3H), 2.96 (dt, *J* = 13.1, 6.4 Hz, 1H), 2.89 -2.77 (m, 2H), 2.41 – 2.25 (m, 2H), 1.95 – 1.73 (m, 2H).

^{13}C NMR (101 MHz, $CDCl_3$) δ 159.6, 142.0, 136.0, 135.7, 128.6, 128.5, 126.0, 124.7, 117.2, 114.6, 55.4, 49.0, 39.0, 35.3, 33.0.

HRMS (ESI): m/z calculated for $C_{19}H_{23}OS^+$ $[M+H]^+$, 299.1464; found, 299.1458.

(cyclopentyl(phenyl)methyl)(4-methoxyphenyl)sulfane (29)

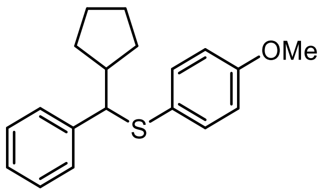

Prepared from **SI-1-17** (0.3 mmol, 1.5 equiv) and **SI-3-7** (0.2 mmol, 1.0 equiv) according to the general procedure 5. Purification by flash column (EtOAc/Petroleum Ether = 1/100) afforded **29** (28 mg, 47% yield) as a white solid.

Rf = 0.10 (Petroleum Ether)

1H NMR (400 MHz, $CDCl_3$) δ 7.21 – 7.09 (m, 5H), 7.07 (d, J = 8.7 Hz, 2H), 6.67 (d, J = 8.7 Hz, 2H), 3.74 (s, 3H), 3.73 (d, J = 10.2 Hz, 1H), 2.37 (d, J = 8.0 Hz, 1H), 2.17 – 2.09 (m, 1H), 1.74 – 1.43 (m, 6H), 1.16 – 1.03 (m, 1H).

^{13}C NMR (126 MHz, $CDCl_3$) δ 159.4, 143.2, 136.0, 128.3, 128.0, 126.7, 125.3, 114.1, 61.8, 55.3, 45.4, 32.4, 32.1, 25.6, 25.3.

HRMS (ESI): m/z calculated for $C_{19}H_{22}OSK^+$ $[M+K]^+$, 337.1023; found, 337.1022.

4-((2-methylfuran-3-yl)thio)-1-tosylpiperidine (30)

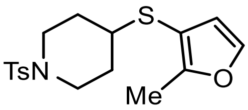

Prepared from **SI-1-1** (0.3 mmol, 1.5 equiv) and **SI-3-19** (0.2 mmol, 1.0 equiv) according to the general procedure 5. Purification by flash column (EtOAc/Petroleum Ether = 1/15) afforded **30** (41 mg, 58% yield) as a white solid.

Prepared from **SI-2-1** (0.1 mmol, 1.0 equiv) and **SI-4-11** (0.25 mmol, 2.5 equiv) according to the general procedure 5. Purification by flash column (EtOAc/Petroleum Ether = 1/15) afforded **30** (32 mg, 90% yield) as a white solid.

Rf = 0.18 (EtOAc/Petroleum Ether = 1/10)

^{1}H NMR (400 MHz, $CDCl_3$) δ 7.61 (d, *J* = 8.3 Hz, 2H), 7.30 (d, *J* = 8.0 Hz, 2H), 7.25 (d, *J* = 1.9 Hz, 1H), 6.23 (d, *J* = 1.8 Hz, 1H), 3.63 (dt, *J* = 11.8, 3.4 Hz, 2H), 2.66 -2.57 (m, 1H), 2.48 – 2.40 (m, 2H), 2.42 (s, 3H), 2.28 (s, 3H), 1.95 – 1.88 (m, 2H), 1.68 – 1.57 (m, 2H).

^{13}C NMR (101 MHz, $CDCl_3$) δ 156.5, 143.7, 140.7, 133.3, 129.8, 127.7, 116.0, 107.7, 45.8, 43.9, 31.6, 21.6, 12.0.

HRMS (ESI): m/z calculated for $C_{17}H_{22}NO_3S_2^+$ $[M+H]^+$, 352.1036; found, 352.1022.

4-(methylthio)-1-tosylpiperidine (31)

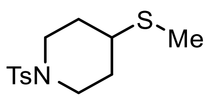

Prepared from **SI-1-1** (0.4 mmol, 2 equiv) and **SI-3-12** (0.2 mmol, 1.0 equiv) according to the general procedure 5. Purification by flash column (EtOAc/Petroleum Ether = 1/15) afforded **31** (20 mg, 36% yield) as a white solid.

Prepared from **SI-2-1** (0.1 mmol, 1.0 equiv) and **SI-4-16** (0.25 mmol, 2.5 equiv) according to the general procedure 6. Purification by flash column (EtOAc/Petroleum Ether = 1/15) afforded **31** (24 mg, 88% yield) as a white solid.

Rf = 0.25 (EtOAc/Petroleum Ether = 1/10)

^{1}H NMR (400 MHz, $CDCl_3$) δ 7.63 (d, *J* = 8.3 Hz, 2H), 7.31 (d, *J* = 8.0 Hz, 2H), 3.63 – 3.53 (m, 2H), 2.59 – 2.51 (m, 2H), 2.51 – 2.47 (m, 1H), 2.43 (s, 3H), 2.03 (s, 3H), 2.02 - 1.95 (m, 2H), 1.73 - 1.63 (m, 2H).

^{13}C NMR (101 MHz, $CDCl_3$) δ 143.7, 133.4, 129.8, 127.8, 45.6, 41.5, 31.3, 21.7, 13.3.

HRMS (ESI): m/z calculated for $C_{13}H_{20}NO_2S_2^+$ $[M+H]^+$, 286.0930; found, 286.0916.

4-(propylthio)-1-tosylpiperidine (32)

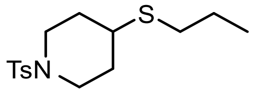

Prepared from **SI-1-1** (0.4 mmol, 2 equiv) and **SI-3-13** (0.2 mmol, 1.0 equiv) according to the general procedure 5. Purification by flash column (EtOAc/Petroleum Ether = 1/15) afforded **32** (26 mg, 41% yield) as a white solid.

Prepared from **SI-2-1** (0.1 mmol, 1.0 equiv) and **SI-4-12** (0.25 mmol, 2.5 equiv) according to the general procedure 6. Purification by flash column (EtOAc/Petroleum Ether = 1/15) afforded **32** (22 mg, 70% yield) as a white solid.

Rf = 0.26 (EtOAc/Petroleum Ether = 1/10)

^{1}H NMR (400 MHz, $CDCl_3$) δ 7.63 (d, *J* = 8.2 Hz, 2H), 7.32 (d, *J* = 8.1 Hz, 2H), 3.55 (dt, *J* = 9.8, 4.0 Hz, 2H), 2.63 – 2.52 (m, 3H), 2.46 (d, *J* = 7.3 Hz, 2H), 2.43 (s, 3H), 2.03 - 1.94 (m, 2H), 1.73 - 1.62 (m, 2H), 1.58 – 1.50 (m, 2H), 0.94 (t, *J* = 7.3 Hz, 3H).

^{13}C NMR (101 MHz, $CDCl_3$) δ 143.7, 133.4, 129.8, 127.8, 45.6, 40.1, 32.4, 32.0, 23.3, 21.7, 13.7.

HRMS (APCI): m/z calculated for $C_{15}H_{24}NO_2S_2^+$ $[M+H]^+$, 314.1243; found, 314.1238.

4-(allylthio)-1-tosylpiperidine (33)

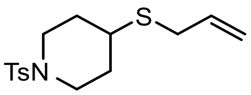

Prepared from **SI-1-1** (0.4 mmol, 2 equiv) and **SI-3-14** (0.2 mmol, 1.0 equiv) according to the general procedure 5. Purification by flash column (EtOAc/Petroleum Ether = 1/15) afforded **33** (13 mg, 21% yield) as a white solid.

Prepared from **SI-2-1** (0.1 mmol, 1.0 equiv) and **SI-4-15** (0.25 mmol, 2.5 equiv) according to the general procedure 6. Purification by flash column (EtOAc/Petroleum Ether = 1/15) afforded **33** (22 mg, 71% yield) as a white solid.

Rf = 0.25 (EtOAc/Petroleum Ether = 1/10)

^{1}H NMR (500 MHz, $CDCl_3$) δ 7.63 (d, *J* = 8.2 Hz, 2H), 7.32 (d, *J* = 8.1 Hz, 2H), 5.80 – 5.69 (m, 1H), 5.13 – 4.98 (m, 2H), 3.58 – 3.48 (m, 2H), 3.11 (d, *J* = 7.1 Hz, 2H), 2.66 – 2.51 (m, 3H), 2.43 (s, 3H), 2.01 - 1.94 (m, 2H), 1.71 - 1.63 (m, 2H).

^{13}C NMR (126 MHz, $CDCl_3$) δ 143.7, 134.6, 133.3, 129.8, 127.8, 117.1, 45.5, 38.8, 33.4, 31.7, 21.7.

HRMS (ESI): m/z calculated for $C_{15}H_{22}NO_2S_2^+$ $[M+H]^+$, 312.1086; found, 312.1075.

4-(benzylthio)-1-tosylpiperidine (34)

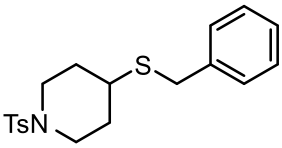

Prepared from **SI-1-1** (0.3 mmol, 1.5 equiv) and **SI-3-15** (0.2 mmol, 1.0 equiv) according to the general procedure 5. Purification by flash column (EtOAc/Petroleum Ether = 1/15) afforded **34** (26 mg, 36% yield) as a white solid.

Prepared from **SI-2-1** (0.1 mmol, 1.0 equiv) and **SI-4-14** (0.25 mmol, 2.5 equiv) according to the general procedure 6. Purification by flash column (EtOAc/Petroleum Ether = 1/15) afforded **34** (34 mg, 95% yield) as a white solid.

Rf = 0.24 (EtOAc/Petroleum Ether = 1/10)

^{1}H NMR (400 MHz, $CDCl_3$) δ = 7.62 (d, *J* =8.3, 2H), 7.31 (d, *J*=8.0, 2H), 7.29 – 7.19 (m, 5H), 3.69 (s, 2H), 3.54 – 3.46 (m, 2H), 2.58 – 2.46 (m, 3H), 2.43 (s, 3H), 1.98 – 1.90 (m, 2H), 1.73 - 1.62 (m, 2H).

^{13}C NMR (126 MHz, $CDCl_3$) δ 143.7, 138.2, 133.3, 129.8, 128.8, 128.7, 127.8, 127.2, 45.4, 39.4, 34.8, 31.7, 21.7.

4-(cyclohexylthio)-1-tosylpiperidine (35)

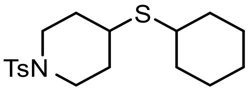

Prepared from **SI-1-1** (0.3 mmol, 1.5 equiv) and **SI-3-16** (0.2 mmol, 1.0 equiv) according to the general procedure 5. Purification by flash column (EtOAc/Petroleum Ether = 1/15) afforded **35** (27 mg, 38% yield) as a white solid.

Prepared from **SI-2-1** (0.1 mmol, 1.0 equiv) and **SI-4-13** (0.25 mmol, 2.5 equiv) according to the general procedure 6. Purification by flash column (EtOAc/Petroleum Ether = 1/15) afforded **35** (25 mg, 69% yield) as a white solid.

Rf = 0.28 (EtOAc/Petroleum Ether = 1/10)

^{1}H NMR (400 MHz, $CDCl_3$) δ 7.62 (d, *J* = 8.2 Hz, 2H), 7.31 (d, *J* = 8.0 Hz, 2H), 3.52 (dt, *J* = 9.7, 3.8 Hz, 2H), 2.71 - 2.62 (m, 2H), 2.62 – 2.52 (m, 2H), 2.42 (s, 3H), 2.02 – 1.93 (m, 2H), 1.91 – 1.83 (m, 2H), 1.77 – 1.60 (m, 4H), 1.29 – 1.18 (m, 6H).

^{13}C NMR (101 MHz, $CDCl_3$) δ 143.6, 133.3, 129.8, 127.8, 45.6, 42.4, 38.5, 34.2, 32.6, 26.2, 25.8, 21.7.

HRMS (ESI): m/z calculated for $C_{18}H_{28}NO_2S_2^+$ $[M+H]^+$,354.1556; found, 354.1542.

(4-methoxyphenyl)(nonyl)sulfane (36)

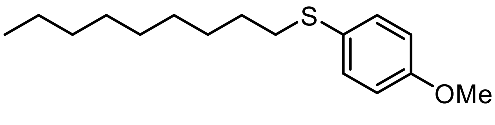

Prepared from **SI-1-22** (0.4 mmol, 2 equiv) and **SI-3-7** (0.2 mmol, 1.0 equiv)

according to the general procedure 5. Purification by flash column (EtOAc/Petroleum Ether = 1/15) afforded **36** (38 mg, 72% yield) as a white solid.

Rf = 0.32 (EtOAc/Petroleum Ether = 1/50)

1H NMR (400 MHz, $CDCl_3$) δ 7.33 (d, J = 8.8 Hz, 2H), 6.84 (d, J = 8.8 Hz, 2H), 3.79 (s, 3H), 2.86 – 2.73 (m, 2H), 1.61 - 1.54 (m, 2H), 1.42 – 1.34 (m, 2H), 1.31 - 1.21 (m, 10H), 0.88 (t, J = 6.9 Hz, 3H)

^{13}C NMR (126 MHz, $CDCl_3$) δ 158.8, 133.0, 127.1, 114.6, 55.5, 36.0, 32.0, 29.6, 29.5, 29.4, 29.3, 28.9, 22.8, 14.3.

HRMS (ESI): m/z calculated for $C_{16}H_{27}OS^+$ $[M+H]^+$, 267.1777; found, 267.1768.

2-(10-((4-methoxyphenyl)thio)decyl)isoindoline-1,3-dione (37)

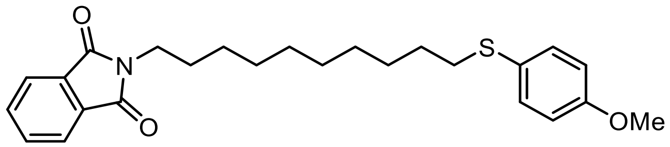

Prepared from **SI-1-24** (0.4 mmol, 2 equiv) and **SI-3-7** (0.2 mmol, 1.0 equiv) according to the general procedure 5. Purification by flash column (EtOAc/Petroleum Ether = 1/20) afforded **37** (55 mg, 65% yield) as a white solid.

Rf = 0.45 (EtOAc/Petroleum Ether = 1/10)

1H NMR (400 MHz, $CDCl_3$) δ 7.83 (dd, J = 5.4, 3.1 Hz, 2H), 7.69 (dd, J = 5.4, 3.0 Hz, 2H), 7.32 (d, J = 8.6 Hz, 2H), 6.83 (d, J = 8.7 Hz, 2H), 3.78 (s, 3H), 3.66 (t, J = 7.3 Hz, 2H), 2.82 – 2.76 (m, 2H), 1.72 – 1.58 (m, 2H), 1.60 – 1.50 (m, 2H), 1.40 – 1.20 (m, 1H).

^{13}C NMR (126 MHz, $CDCl_3$) δ 168.6, 158.8, 134.0, 133.0, 132.3, 127.1, 123.3, 114.6, 55.4, 38.2, 35.9, 29.5, 29.5, 29.5, 29.3, 29.2, 28.8, 28.7, 27.0.

HRMS (ESI): m/z calculated for $C_{25}H_{32}NO_3S^+$ $[M+H]^+$, 426.2097; found, 426.2100.

(4-methoxyphenyl)(3-phenylpropyl)sulfane (38)

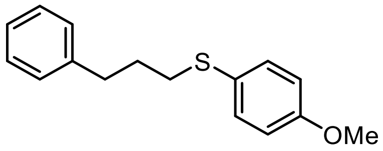

Prepared from **SI-1-23** (0.4 mmol, 2 equiv) and **SI-3-7** (0.2 mmol, 1.0 equiv)

according to the general procedure 5. Purification by flash column (EtOAc/Petroleum Ether = 1/100) afforded **38** (37 mg, 71% yield) as a white solid.

Rf = 0.33 (EtOAc/Petroleum Ether = 1/50)

^{1}H NMR (400 MHz, $CDCl_3$) δ 7.35 (d, *J* = 8.7 Hz, 2H), 7.29 (t, *J* = 7.4 Hz, 2H), 7.22 – 7.15 (m, 3H), 6.85 (d, *J* = 8.7 Hz, 2H), 3.80 (s, 3H), 2.84 (t, *J* = 7.2 Hz, 2H), 2.74 (t, *J* = 7.6 Hz, 2H), 1.96 – 1.87 (m, 2H).

^{13}C NMR (126 MHz, $CDCl_3$) δ 159.0, 141.6, 133.3, 128.6, 128.5, 126.6, 126.0, 114.7, 55.5, 35.3, 34.7, 30.9.

HRMS (ESI): m/z calculated for $C_{16}H_{18}OSK^+$ $[M+K]^+$, 297.0710; found, 297.0724.

4-((4-bromophenyl)thio)-1-(4-fluorophenyl)butan-1-one (39)

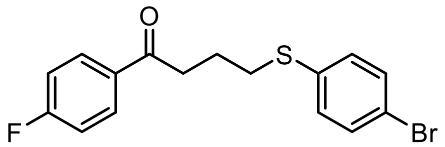

Prepared from **SI-1-26** (0.4 mmol, 2 equiv) and **SI-3-4** (0.2 mmol, 1.0 equiv) according to the general procedure 5. Purification by flash column (EtOAc/Petroleum Ether = 1/20) afforded **39** (25 mg, 36% yield) as a white solid.

Rf = 0.50 (EtOAc/Petroleum Ether = 1/10)

^{1}H NMR (400 MHz, $CDCl_3$) δ 8.02 – 7.92 (m, 2H), 7.44 – 7.35 (m, 2H), 7.24 – 7.18 (m, 2H), 7.18 – 7.04 (m, 2H), 3.10 (t, *J* = 6.9 Hz, 2H), 3.02 (t, *J* = 7.0 Hz, 2H), 2.12 – 2.04 (m, 2H).

^{13}C NMR (101 MHz, $CDCl_3$) δ 197.7, 165.9 (d, *J* = 254.8 Hz), 135.5, 133.3 (d, *J* = 2.8 Hz), 132.1, 130.8, 130.8 (d, *J* = 9.4 Hz), 120.0, 115.9 (d, *J* = 21.9 Hz), 36.8, 33.2, 23.4.

HRMS (EI): m/z calculated for $C_{16}H_{14}BrFOS^+$ $[M]^+$, 351.9927; found, 351.9922.

methyl 4-((4-bromophenyl)thio)butanoate (40)

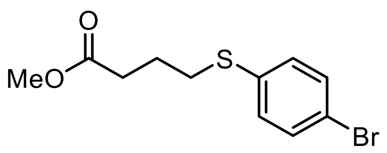

Prepared from **SI-1-25** (0.4 mmol, 2 equiv) and **SI-3-4** (0.2 mmol, 1.0 equiv)

according to the general procedure 5. Purification by flash column (EtOAc/Petroleum Ether = 1/20) afforded **40** (29 mg, 51% yield) as a white solid.

Rf = 0.42 (EtOAc/Petroleum Ether = 1/10)

^{1}H NMR (400 MHz, $CDCl_3$) δ 7.40 (d, *J* = 8.6 Hz, 2H), 7.20 (d, *J* = 8.6 Hz, 2H), 3.67 (s, 3H), 2.94 (t, *J* = 7.2 Hz, 2H), 2.46 (t, *J* = 7.2 Hz, 2H), 2.00 - 1.88 (m, 2H).

^{13}C NMR (101 MHz, $CDCl_3$) δ 173.4, 135.5, 132.1, 131.0, 120.1, 51.8, 33.2, 32.7, 24.4.

HRMS (ESI): m/z calculated for $C_{11}H_{13}BrO_2SK^+$ $[M+K]^+$, 326.9451; found, 326.9467.

(4-bromophenyl)((2,4-dichlorophenoxy)methyl)sulfane (41)

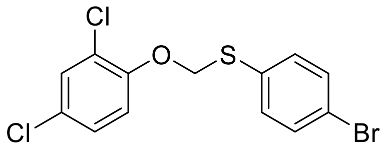

Prepared from **SI-1-28** (0.4 mmol, 2 equiv) and **SI-3-4** (0.2 mmol, 1.0 equiv) according to the general procedure 5. Purification by flash column (EtOAc/Petroleum Ether = 1/100) afforded **41** (22 mg, 34% yield) as a white solid.

Rf = 0.52 (EtOAc/Petroleum Ether = 1/50)

Following the GP V with S1 (0.3 mmol) and B1 (0.2 mmol, 1.0 equiv), purification by flash column (100:1 Petroleum ether/Ethyl acetate) afforded (22 mg, 34% yield) as a white solid.

Rf = 0.52 (50:1 Petroleum ether / Ethyl acetate)

^{1}H NMR (400 MHz, $CDCl_3$) δ 7.48 – 7.43 (m, 2H), 7.42 – 7.36 (m, 3H), 7.20 (dd, *J* = 8.8, 2.5 Hz, 1H), 6.92 (d, *J* = 8.8 Hz, 1H), 5.48 (s, 2H).

^{13}C NMR (126 MHz, $CDCl_3$) δ 151.0, 133.6, 132.7, 132.4, 130.6, 127.9, 127.6, 125.5, 122.0, 117.2, 74.5.

HRMS (EI): m/z calculated for $C_{13}H_9BrCl_2OS^+$ $[M]^+$, 361.8929; found, 361.8927.

***tert*-butyl(4-methoxyphenyl)sulfane (42)**

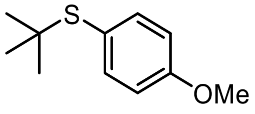

Prepared from **SI-1-31** (0.4 mmol, 2 equiv) and **SI-3-7** (0.2 mmol, 1.0 equiv) according to the general procedure 5. Purification by flash column (EtOAc/Petroleum Ether = 1/100) afforded **42** (30 mg, 77% yield) as oil.

Rf = 0.48 (EtOAc/Petroleum Ether = 1/50)

1H NMR (400 MHz, $CDCl_3$) δ 7.44 (d, J = 8.7 Hz, 2H), 6.86 (d, J = 8.7 Hz, 2H), 3.81 (s, 3H), 1.26 (s, 9H).

^{13}C NMR (101 MHz, $CDCl_3$) δ 160.3, 139.0, 123.7, 114.1, 55.4, 45.6, 30.9.

HRMS (EI): m/z calculated for $C_{11}H_{16}OS^+$ $[M]^+$, 196.0916; found, 196.0915.

methyl 4-((4-bromophenyl)thio)bicyclo[2.2.2]octane-1-carboxylate (43)

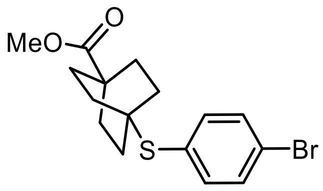

Prepared from **SI-1-33** (0.4 mmol, 2 equiv) and **SI-3-4** (0.2 mmol, 1.0 equiv) according to the general procedure 5. Purification by flash column (EtOAc/Petroleum Ether = 1/15) afforded **43** (34 mg, 48% yield) as a white solid.

Rf = 0.34 (EtOAc/Petroleum Ether = 1/10)

1H NMR (400 MHz, $CDCl_3$) δ 7.44 (d, J = 8.3 Hz, 2H), 7.33 (d, J = 8.3 Hz, 2H), 3.61 (s, 3H), 1.88 – 1.77 (m, 6H), 1.78 – 1.67 (m, 6H).

^{13}C NMR (126 MHz, $CDCl_3$) δ 177.7, 139.1, 131.9, 130.1, 123.7, 51.9, 46.3, 38.5, 32.0, 29.4.

HRMS (APCI): m/z calculated for $C_{16}H_{20}BrO_2S^+$ $[M+H]^+$, 355.0362; Found, 355.0368.

(adamantan-1-yl)(4-methoxyphenyl)sulfane (44)

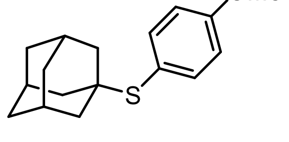

Prepared from **SI-1-34** (0.4 mmol, 2 equiv) and **SI-3-7** (0.2 mmol, 1.0 equiv) according to the general procedure 5. Purification by flash column (EtOAc/Petroleum Ether = 1/100) afforded **44** (37 mg, 67% yield) as a white solid.

Rf = 0.52 (EtOAc/Petroleum Ether = 1/50)

1H NMR (500 MHz, $CDCl_3$) δ 7.40 (d, J = 8.5 Hz, 2H), 6.84 (d, J = 8.6 Hz, 2H), 3.81 (s, 3H), 2.00 (brs, 3H), 1.78 (d, J = 2.3 Hz, 6H), 1.66 – 1.56 (m, 6H).

^{13}C NMR (101 MHz, $CDCl_3$) δ 160.3, 139.1, 121.5, 113.9, 55.4, 47.5, 43.6, 36.3, 30.1.

HRMS (EI): m/z calculated for $C_{17}H_{22}OS^+$ $[M]^+$, 274.1386; found, 274.1385.

4-(phenylselanyl)-1-tosylpiperidine (45)

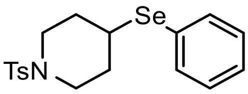

Prepared from **SI-1-1** (0.4 mmol, 2 equiv) and **SI-3-17** (0.2 mmol, 1.0 equiv) according to the general procedure 5. Purification by flash column (EtOAc/Petroleum Ether = 1/15) afforded **45** (63 mg, 80% yield) as a white solid.

Prepared from **SI-1-1** (0.4 mmol, 2 equiv) and **SI-3-17** (0.2 mmol, 1.0 equiv) according to the general procedure 5 (without 2,2':6',2"-terpyridine). Purification by flash column (EtOAc/Petroleum Ether = 1/15) afforded **45** (59 mg, 75% yield) as a white solid.

Rf = 0.30 (EtOAc/Petroleum Ether = 1/10)

^{1}H NMR (400 MHz, $CDCl_3$) δ 7.61 (d, *J* = 8.2 Hz, 2H), 7.49 (d, *J* = 6.6 Hz, 2H), 7.31 (d, *J* = 8.1 Hz, 2H), 7.28 – 7.22 (m, 3H), 3.54 (dt, *J* = 9.5, 3.8 Hz, 2H), 3.12 – 3.03 (m, 1H), 2.57 – 2.48 (m, 2H), 2.43 (s, 3H), 2.04 (dd, *J* = 13.6, 3.4 Hz, 2H), 1.86 – 1.75 (m, 2H).

^{13}C NMR (101 MHz, $CDCl_3$) δ 143.7, 135.5, 133.3, 129.8, 129.2, 128.1, 127.8, 127.8, 46.4, 38.9, 32.5, 21.7.

HRMS (ESI): m/z calculated for $C_{18}H_{22}NO_2SSe^+$ $[M+H]^+$, 396.0531; found, 396.0520.

2-(10-(phenylselanyl)decyl)isoindoline-1,3-dione (46)

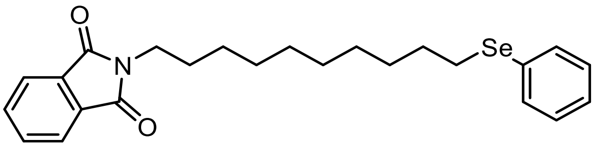

Prepared from **SI-1-24** (0.4 mmol, 2 equiv) and **SI-3-17** (0.2 mmol, 1.0 equiv) according to the general procedure 5. Purification by flash column (EtOAc/Petroleum Ether = 1/15) afforded **46** (56 mg, 63% yield) as a white solid.

Rf = 0.53 (EtOAc/Petroleum Ether = 1/10)

^{1}H NMR (500 MHz, $CDCl_3$) δ 7.85 (dd, *J* = 5.3, 3.1 Hz, 2H), 7.71 (dd, *J* = 5.4, 3.0 Hz, 2H), 7.49 (d, *J* = 6.8 Hz, 2H), 7.31 – 7.16 (m, 3H), 3.69 (t, *J* = 7.3 Hz, 2H), 2.91 (t, *J* = 7.5 Hz, 2H), 1.69 (dd, *J* = 12.8, 5.1 Hz, 4H), 1.44 – 1.18 (m, 12H).

^{13}C NMR (126 MHz, $CDCl_3$) δ 168.6, 133.9, 132.5, 132.3, 130.8, 129.1, 126.7, 123.3, 38.2, 30.2, 29.9, 29.5, 29.2, 29.1, 28.7, 28.0, 27.0.

HRMS (ESI): m/z calculated for $C_{24}H_{29}NO_2SeNa^+$ $[M+Na]^+$, 466.1257; found, 466.1273.

4-(phenyltellanyl)-1-tosylpiperidine (47)

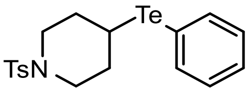

Prepared from **SI-1-1** (0.4 mmol, 2 equiv) and **SI-3-18** (0.2 mmol, 1.0 equiv) according to the general procedure 5. Purification by flash column (EtOAc/Petroleum Ether = 1/15) afforded **47** (85 mg, 96% yield) as a white solid.

Rf = 0.31 (EtOAc/Petroleum Ether = 1/10)

^{1}H NMR (400 MHz, $CDCl_3$) δ 7.75 (d, *J* = 7.0 Hz, 2H), 7.60 (d, *J* = 8.2 Hz, 2H), 7.31 (t, *J* = 7.0 Hz, 3H), 7.20 (t, *J* = 7.5 Hz, 2H), 3.47 (dt, *J* = 11.5, 3.5 Hz, 2H), 3.26 – 3.11 (m, 1H), 2.51 – 2.44 (m, 2H), 2.42 (s, 3H), 2.12 (dd, *J* = 13.6, 3.5 Hz, 2H), 2.00 - 1.88 (m, 2H).

^{13}C NMR (126 MHz, $CDCl_3$) δ 143.6, 140.6, 133.1, 129.7, 129.4, 128.5, 127.7, 110.4, 47.9, 34.5, 21.6, 21.4.

HRMS (ESI): m/z calculated for $C_{18}H_{22}NO_2STe^+$ $[M+H]^+$, 446.0427; found, 446.0424.

2-(10-(phenyltellanyl)decyl)isoindoline-1,3-dione (48)

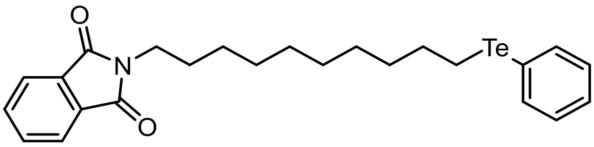

Prepared from **SI-1-24** (0.4 mmol, 2 equiv) and **SI-3-18** (0.2 mmol, 1.0 equiv) according to the general procedure 5. Purification by flash column (EtOAc/Petroleum Ether = 1/100) afforded **48** (89 mg, 90% yield) as a white solid.

Rf = 0.50 (EtOAc/Petroleum Ether = 1/10)

^{1}H NMR (500 MHz, $CDCl_3$) δ 7.83 (tt, J = 5.1, 2.5 Hz, 2H), 7.74 – 7.66 (m, 4H), 7.28 – 7.16 (m, 3H), 3.69 – 3.65 (m, 2H), 2.89 (t, J = 7.6 Hz, 2H), 1.82 - 1.74 (m, 2H), 1.70 - 1.63 (m, 2H), 1.37 – 1.21 (m, 12H).

^{13}C NMR (126 MHz, $CDCl_3$) δ 168.6, 138.4, 134.0, 132.3, 129.2, 127.5, 123.3, 112.0, 38.2, 32.0, 31.9, 29.5, 29.3, 29.0, 28.7, 27.0, 8.9.

(adamantan-1-yl)(phenyl)tellane (49)

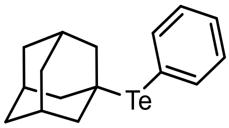

Prepared from **SI-1-34** (0.4 mmol, 2 equiv) and **SI-3-18** (0.2 mmol, 1.0 equiv) according to the general procedure 5. Purification by flash column (EtOAc/Petroleum Ether = 1/15) afforded **49** (68 mg, 99% yield) as a white solid.

Rf = 0.52 (EtOAc/Petroleum Ether = 1/10)

^{1}H NMR (400 MHz, $CDCl_3$) δ 7.86 (d, *J* = 7.2 Hz, 2H), 7.36 (t, *J* = 7.4 Hz, 1H), 7.23 (t, *J* = 6.9 Hz, 2H), 2.22 (d, *J* = 2.2 Hz, 6H), 1.90 (brs, 3H), 1.72 (brs, 6H).

^{13}C NMR (126 MHz, $CDCl_3$) δ 142.4, 129.0, 128.4, 111.5, 47.5, 36.4, 35.7, 31.5.

HRMS (EI): m/z calculated for $C16H20Te^+$ $[M]^+$, 342.0622; found, 342.0616.

(R)-2-(2-(((4-methoxyphenyl)thio)methyl)-4-methylpentyl)isoindoline-1,3-dione (50)

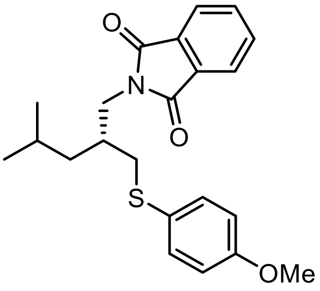

Prepared from **SI-1-35** (0.4 mmol, 2 equiv) and **SI-3-7** (0.2 mmol, 1.0 equiv) according to the general procedure 5. Purification by flash column (EtOAc/Petroleum

Ether = 1/15) afforded **50** (54 mg, 71% yield) as a white solid.

Rf = 0.20 (EtOAc/Petroleum Ether = 1/10)

^{1}H NMR (500 MHz, $CDCl_3$) δ 7.83 (dd, J = 5.4, 3.1 Hz, 2H), 7.71 (dd, J = 5.4, 3.0 Hz, 2H), 7.30 (d, J = 8.7 Hz, 2H), 6.74 (d, J = 8.7 Hz, 2H), 3.78 – 3.72 (m, 1H), 3.75 (s, 3H), 3.64 (dd, J = 13.7, 6.0 Hz, 1H), 2.84 - 2.74 (m, 2H), 2.14 (dt, J = 13.4, 6.7 Hz, 1H), 1.71 – 1.56 (m, 1H), 1.38 (dt, J = 14.0, 7.0 Hz, 1H), 1.14 (dt, J = 14.0, 7.1 Hz, 1H), 0.87 (d, J = 6.6 Hz, 3H), 0.77 (d, J = 6.5 Hz, 3H).

^{13}C NMR (126 MHz, $CDCl_3$) δ 168.7, 159.0, 134.0, 133.8, 132.2, 126.9, 123.3, 114.6, 55.4, 42.1, 41.2, 40.0, 35.6, 25.4, 22.9, 22.6.

HRMS (ESI): m/z calculated for $C_{22}H_{25}NO_3SNa^+$ $[M+Na]^+$, 406.1447; found, 406.1450.

4-(4-((4-bromophenyl)thio)piperidine-1-carbonyl)-*N,N*-dipropylbenzenesulfonamide (51)

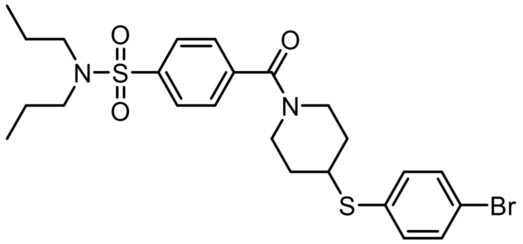

Prepared from **SI-1-5** (0.3 mmol, 1.5 equiv) and **SI-3-4** (0.2 mmol, 1.0 equiv) according to the general procedure 5. Purification by flash column (EtOAc/Petroleum Ether = 1/5) afforded **51** (79 mg, 73% yield) as a white solid.

Rf = 0.22 (EtOAc/Petroleum Ether = 1/5)

^{1}H NMR (500 MHz, $CDCl_3$) δ 7.83 (d, *J* = 8.2 Hz, 2H), 7.48 (d, *J* = 8.2 Hz, 2H), 7.42 (d, *J* = 8.4 Hz, 2H), 7.28 (d, *J* = 8.4 Hz, 2H), 4.41 (s, 1H), 3.61 (s, 1H), 3.29 (tt, *J* = 9.6, 3.8 Hz, 1H), 3.13 (s, 2H), 3.09 – 3.00 (m, 4H), 2.05 (s, 1H), 1.89 (s, 1H), 1.67 (s, 2H), 1.60 – 1.47 (m, 4H), 0.86 (t, *J* = 7.4 Hz, 6H).

^{13}C NMR (126 MHz, $CDCl_3$) δ 168.8, 141.4, 139.7, 134.5, 132.6, 132.3, 127.5, 122.0, 50.2, 46.9, 44.5, 41.5, 32.7, 31.8, 22.2, 11.3.

HRMS (ESI): m/z calculated for $C_{24}H_{32}BrN_2O_3S_2^+$ $[M+H]^+$, 539.1032; found, 539.1022.

(8*R*, 9*S*, 10*R*, 13*S*, 14*S*, 17*R*)-17-((4-bromophenyl)thio)-10,13-dimethyl-1, 2, 6, 7, 8, 9, 10, 11, 12, 13, 14, 15, 16, 17-tetradecahydro-3*H*-cyclopenta[*a*]phenanthren-3-one (52)

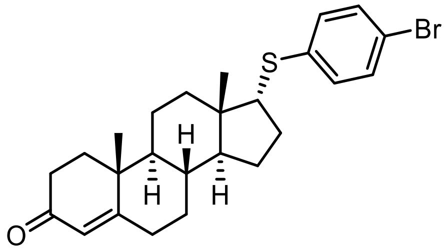

Prepared from **SI-1-36** (0.3 mmol, 1.5 equiv) and **SI-3-4** (0.2 mmol, 1.0 equiv) according to the general procedure 5. Purification by flash column (EtOAc/Petroleum Ether = 1/5) afforded **52** (41 mg, 45% yield) as a white solid.

Rf = 0.18 (EtOAc/Petroleum Ether = 1/5)

^{1}H NMR (400 MHz, $CDCl_3$) δ 7.38 – 7.34 (m, 2H), 7.18 – 7.14 (m, 2H), 5.73 (s, 1H), 3.47 (dd, *J* = 8.1, 2.0 Hz, 1H), 2.47 – 2.34 (m, 4H), 2.33 – 2.23 (m, 1H), 2.01 (ddd, *J* = 13.4, 4.9, 3.3 Hz, 1H), 1.87 (ddt, *J* = 11.0, 5.5, 2.5 Hz, 1H), 1.81 (ddd, *J* = 9.5, 6.8, 3.5 Hz, 1H), 1.75 – 1.69 (m, 2H), 1.69 – 1.64 (m, 1H), 1.56 (dd, *J* = 8.6, 3.3 Hz, 1H), 1.50 (dd, *J* = 12.1, 5.0 Hz, 2H), 1.46 – 1.37 (m, 1H), 1.36 – 1.24 (m, 2H), 1.17 (s, 3H), 1.15 – 1.06 (m, 1H), 1.00 (d, *J* = 1.8 Hz, 1H), 0.93 (s, 3H).

^{13}C NMR (101 MHz, $CDCl_3$) δ 199.7, 171.2, 137.2, 131.9, 130.5, 124.1, 119.2, 56.2, 53.4, 50.0, 45.8, 38.7, 36.2, 35.8, 34.6, 34.1, 33.0, 32.3, 30.8, 25.2, 21.1, 19.8, 17.6.

HRMS (ESI): m/z calculated for $C_{25}H_{32}BrOS^{+}$ $[M+H]^{+}$, 459.1352; found, 459.1352.

(4-bromophenyl)(5-(2,5-dimethylphenoxy)-2-methylpentan-2-yl)sulfane (53)

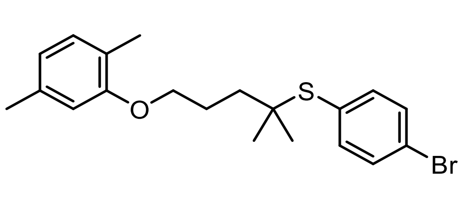

Prepared from **SI-1-37** (0.4 mmol, 2.0 equiv) and **SI-3-4** (0.2 mmol, 1.0 equiv) according to the general procedure 5. Purification by flash column (EtOAc/Petroleum Ether = 1/20) afforded **53** (31 mg, 40% yield) as a white solid.

Rf = 0.52 (EtOAc/Petroleum Ether = 1/10)

^{1}H NMR (400 MHz, $CDCl_3$) δ 7.45 (d, *J* = 8.4 Hz, 2H), 7.38 (d, *J* = 8.4 Hz, 2H), 7.03 (d, *J* = 7.5 Hz, 1H), 6.68 (d, *J* = 7.5 Hz, 1H), 6.64 (s, 1H), 3.96 (t, *J* = 6.2 Hz, 2H), 2.33 (s, 3H), 2.18 (s, 3H), 2.04 – 1.96 (m, 2H), 1.68 – 1.63 (m, 2H), 1.28 (s, 6H).

^{13}C NMR (101 MHz, $CDCl_3$) δ 157.0, 139.0, 136.6, 131.8, 131.5, 130.5, 123.7, 123.6, 120.9, 112.0, 68.0, 49.4, 38.9, 28.9, 25.2, 21.6, 16.0.

HRMS (ESI): m/z calculated for $C_{20}H_{25}BrOSNa^+$ $[M+Na]^+$, 415.0702; found, 415.0701.

4-(phenylthio)tetrahydro-2H-pyran (54)

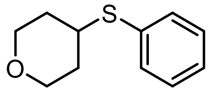

Prepared from **SI-2-3** (0.1 mmol, 1.0 equiv) and **SI-4-1** (0.25 mmol, 2.5 equiv) according to the general procedure 6. Purification by flash column (EtOAc/Petroleum Ether = 1/50) afforded **54** (15 mg, 78% yield) as a white solid.

Rf = 0.40 (EtOAc/Petroleum Ether = 1/20)

^{1}H NMR (400 MHz, $CDCl_3$) δ 7.47 – 7.39 (m, 2H), 7.34 – 7.23 (m, 3H), 3.97 (dt, *J* = 11.7, 3.8 Hz, 2H), 3.43 (ddd, *J* = 11.6, 10.7, 2.4 Hz, 2H), 3.27 (tt, *J* = 10.6, 4.0 Hz, 1H), 1.90 (dtd, *J* = 11.8, 4.0, 2.2 Hz, 2H), 1.67 (dtd, *J* = 13.5, 10.7, 4.2 Hz, 2H).

^{13}C NMR (101 MHz, $CDCl_3$) δ 133.9, 132.9, 129.1, 127.4, 67.5, 43.6, 33.3.

cyclooctyl(phenyl)sulfane (55)

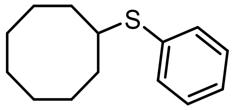

Prepared from **SI-2-5** (0.1 mmol, 1.0 equiv) and **SI-4-1** (0.25 mmol, 2.5 equiv) according to the general procedure 6. Purification by flash column (Petroleum Ether) afforded **55** (18 mg, 81% yield) as oil.

Rf = 0.68 (Petroleum Ether)

^{1}H NMR (400 MHz, $CDCl_3$) δ 7.38 (d, *J* = 7.8 Hz, 2H), 7.30 – 7.25 (m, 2H), 7.19 (t, *J* = 7.3 Hz, 1H), 3.39 (tt, *J* = 8.4, 3.8 Hz, 1H), 2.01 – 1.91 (m, 2H), 1.81 – 1.72 (m, 2H), 1.71 – 1.64 (m, 2H), 1.61 – 1.48 (m, 8H).

^{13}C NMR (101 MHz, $CDCl_3$) δ 136.3, 131.6, 128.9, 126.5, 47.8, 32.2, 27.3, 26.0, 25.3.

HRMS (EI): m/z calculated for $C_{14}H_{20}S^+$ $[M]^+$, 220.1280; found, 220.1278.

(4-methoxyphenyl)(4-phenylbutan-2-yl)sulfane (56)

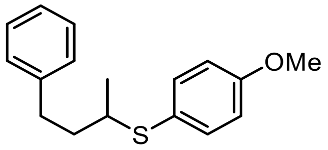

Prepared from **SI-2-7** (0.1 mmol, 1.0 equiv) and **SI-4-5** (0.25 mmol, 2.5 equiv) according to the general procedure 6. Purification by flash column (Petroleum Ether) afforded **56** (20 mg, 74% yield) as a white solid.

Rf = 0.1 (Petroleum Ether)

^{1}H NMR (400 MHz, CDCl3) δ 7.44 – 7.36 (m, 2H), 7.33 – 7.27 (m, 2H), 7.24 – 7.17 (m, 3H), 6.93 – 6.81 (m, 2H), 3.83 (s, 3H), 3.04 (p, *J* = 6.7 Hz, 1H), 2.87 – 2.74 (m, 2H), 1.96 – 1.72 (m, 2H), 1.29 (d, *J* = 6.7 Hz, 3H).

^{13}C NMR (101 MHz, $CDCl_3$) δ 159.6, 142.0, 135.9, 128.6, 128.5, 126.0, 124.9, 114.5, 55.5, 44.0, 38.2, 33.4, 21.4.

HRMS (ESI): m/z calculated for $C_{17}H_{21}OS^+$ $[M+H]^+$, 273.1308; found, 273.1318.

benzyl(4-methoxyphenyl)sulfane (57)

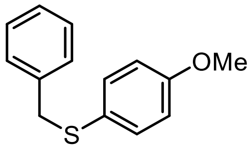

Prepared from **SI-2-10** (0.1 mmol, 1.0 equiv) and **SI-4-5** (0.25 mmol, 2.5 equiv) according to the general procedure 6. Purification by flash column (EtOAc/Petroleum Ether = 1/100) afforded **57** (13 mg, 56% yield) as a white solid.

Rf = 0.11 (Petroleum Ether)

^{1}H NMR (400 MHz, $CDCl_3$) δ 7.27 – 7.17 (m, 7H), 6.83 – 6.75 (m, 2H), 3.98 (s, 2H), 3.78 (s, 3H).

^{13}C NMR (101 MHz, $CDCl_3$) δ 159.4, 138.3, 134.2, 129.0, 128.5, 127.1, 126.2, 114.6, 55.4, 41.4.

HRMS (EI): m/z calculated for $C_{14}H_{14}OS^+$ $[M]^+$, 230.0760; found, 230.0759.

(2*R*,3*R*,4*S*,5*R*,6*S*)-2-(acetoxymethyl)-6-((tetrahydro-2*H*-pyran-4-yl)thio)tetrahydro-2*H*-pyran-3,4,5-triyl triacetate (58)

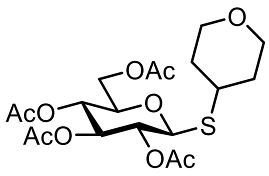

Prepared from **SI-2-3** (0.1 mmol, 1.0 equiv) and **SI-4-19** (0.25 mmol, 2.5 equiv) according to the general procedure 6. Purification by flash column (Petroleum Ether/Ethyl acetate = 1/5) afforded **58** (31 mg, 25% yield) as a white solid.

Rf = 0.65 (Petroleum Ether/Ethyl acetate = 1/1)

^{1}H NMR (400 MHz, CDCl3) δ 5.43 (dd, *J* = 3.3, 1.2 Hz, 1H), 5.22 (t, *J* = 10.0 Hz, 1H), 5.04 (dd, *J* = 10.0, 3.4 Hz, 1H), 4.60 (d, *J* = 9.9 Hz, 1H), 4.16 (dd, *J* = 11.3, 6.9 Hz, 1H), 4.08 (dd, *J* = 11.4, 6.4 Hz, 1H), 4.00 – 3.88 (m, 3H), 3.50 – 3.40 (m, 2H), 3.17 – 3.08 (m, 1H), 2.15 (s, 3H), 2.05 (s, 3H), 2.03 (s, 3H), 1.98 (s, 3H), 1.76 – 1.60 (m, 4H).

^{13}C NMR (126 MHz, $CDCl_3$) δ 170.5, 170.4, 170.2, 169.7, 83.5, 74.5, 72.0, 67.5, 67.4, 67.3, 61.7, 40.5, 34.2, 33.9, 21.0, 20.8 (2C), 20.7.

HRMS (ESI): m/z calculated for $C_{19}H_{28}O_{10}SNa^+$ $[M+Na]^+$, 471.1295; found, 471.1313.

(2*R*,3*R*,4*S*,5*R*,6*S*)-2-(acetoxymethyl)-6-((1-tosylpiperidin-4-yl)thio)tetrahydro-2*H*-pyran-3,4,5-triyl triacetate (59)

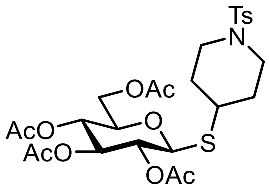

Following the General Procedure 6 (80 ^{o}C) with **SI-2-1** (0.1 mmol, 1.0 equiv) and **SI-4-19** (0.3 mmol, 3.0 equiv) according to the general procedure 6. Purification by flash column (Petroleum Ether/Ethyl acetate = 1/5) afforded **59** (24 mg, 43% yield) as a white solid.

Rf = 0.65 (Petroleum Ether/Ethyl acetate = 1/1)

^{1}H NMR (400 MHz, $CDCl_3$) δ 7.63 (d, *J* = 8.3 Hz, 2H), 7.32 (d, *J* = 8.0 Hz, 2H), 5.40 (dd, *J* = 3.4, 1.1 Hz, 1H), 5.16 (t, *J* = 10.0 Hz, 1H), 5.01 (dd, *J* = 10.0, 3.4 Hz, 1H), 4.51 (d, *J* = 9.9 Hz, 1H), 4.15 – 4.01 (m, 2H), 3.86 (td, *J* = 6.6, 1.2 Hz, 1H), 3.57 – 3.44 (m, 2H), 2.89 (dq, *J* = 9.6, 4.7, 3.9 Hz, 1H), 2.62 (d, *J* = 10.2 Hz, 2H), 2.44 (s, 3H), 2.12 (s, 3H), 2.06 – 1.95 (m, 11H), 1.76 (dddd, *J* = 12.3, 9.9, 7.2, 4.9 Hz, 2H).

^{13}C NMR (126 MHz, $CDCl_3$) δ 170.5, 170.3, 170.2, 169.7, 143.8, 133.1, 129.9, 127.8, 83.7, 74.6, 71.9, 67.3 (2C), 61.5, 45.5, 45.4, 40.4, 32.7, 32.2, 21.7, 20.9, 20.8, 20.7.

HRMS (ESI): m/z calculated for $C_{26}H_{35}NO_{11}S_2Na^+$ $[M+Na]^+$, 624.1544; found, 624.1575.

(2*R*,3*R*,4*S*,5*R*,6*S*)-2-(acetoxymethyl)-6-((2,3-dihydro-1*H*-inden-2-yl)thio)tetrahydro-2*H*-pyran-3,4,5-triyl triacetate (60)

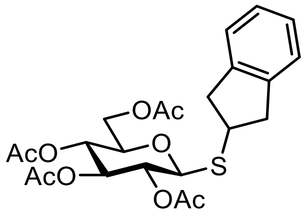

Prepared from **SI-2-6** (0.1 mmol, 1.0 equiv) and **SI-4-19** (0.25 mmol, 2.5 equiv) according to the general procedure 6. Purification by flash column (Petroleum Ether/Ethyl acetate = 1/5) afforded **60** (20 mg, 42% yield) as a white solid.

Rf = 0.62 (Petroleum Ether/Ethyl acetate = 1/2)

^{1}H NMR (400 MHz, $CDCl_3$) δ 7.22 – 7.12 (m, 4H), 5.44 (dd, *J* = 3.4, 1.2 Hz, 1H), 5.25 (t, *J* = 10.0 Hz, 1H), 5.06 (dd, *J* = 10.0, 3.4 Hz, 1H), 4.61 (d, *J* = 10.0 Hz, 1H), 4.15 (qd, *J* = 11.3, 6.6 Hz, 2H), 3.92 – 3.79 (m, 2H), 3.35 (td, *J* = 15.6, 7.7 Hz, 2H), 3.01 (ddd, *J* = 16.1, 11.9, 7.4 Hz, 2H), 2.15 (s, 3H), 2.05 (s, 6H).

^{13}C NMR (101 MHz, $CDCl_3$) δ 170.5, 170.4, 170.2, 169.7, 141.7, 141.5, 126.9, 126.8, 124.4, 84.3, 74.6, 72.1, 67.5 (2C), 61.7, 43.0, 41.5, 40.9, 21.0, 20.8 (2C), 20.7.

HRMS (APCI): m/z calculated for $C_{23}H_{32}NO_9S^+$ $[M+NH_4]^+$, 498.1792; found, 498.1807.

(2*R*,3*R*,4*S*,5*R*,6*S*)-2-(acetoxymethyl)-6-((4-phenylbutan-2-yl)thio)tetrahydro-2*H*-pyran-3,4,5-triyl triacetate (61)

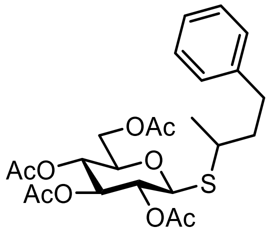

Following the General Procedure 6 (80 ^{o}C) with **SI-2-7** (0.1 mmol, 1.0 equiv) and **SI-4-19** (0.3 mmol, 3 equiv) according to the general procedure 6. Purification by flash column (Petroleum Ether/Ethyl acetate = 1/5) afforded **61** (19 mg, 39% yield, dr = 1/1) as a white solid.

Rf = 0.48 (Petroleum Ether/Ethyl acetate = 1/2)

^{1}H NMR (400 MHz, CDCl3) δ 7.32 – 7.26 (m, 2H), 7.22 – 7.16 (m, 3H), 5.42 (ddd, *J* = 5.8, 3.4, 1.2 Hz, 1H), 5.22 (td, *J* = 10.0, 5.6 Hz, 1H), 5.05 (ddd, *J* = 9.9, 4.9, 3.4 Hz, 1H), 4.55 (dd, *J* = 14.4, 10.0 Hz, 1H), 4.19 – 4.03 (m, 2H), 3.87 (dtd, *J* = 18.7, 6.7, 1.2 Hz, 1H), 2.99 (dh, *J* = 20.4, 6.7 Hz, 1H), 2.75 (q, *J* = 7.5, 6.8 Hz, 2H), 2.16 (s, 3H), 2.06 (d, *J* = 2.4 Hz, 3H), 2.01 – 1.98 (m, 5H), 1.96 – 1.81 (m, 2H), 1.37 (dd, *J* = 6.8, 4.9 Hz, 3H).

^{13}C NMR (126 MHz, $CDCl_3$) δ 170.54, 170.50, 170.43, 170.24, 169.66, 141.67, 128.60, 128.56, 128.54, 128.51, 126.14, 126.10, 84.43, 83.80, 74.47, 74.42, 72.11, 72.08, 67.66, 67.62, 67.40, 67.36, 61.72, 40.83, 40.29, 39.13, 38.77, 33.13, 33.00, 22.33, 22.31, 20.99, 20.85, 20.83, 20.75.

HRMS (ESI): m/z calculated for $C_{24}H_{32}O_9SNa^+$ $[M+Na]^+$, 519.1659; found, 519.1672.

4-(*tert*-butyldisulfanyl)-1-tosylpiperidine (62)

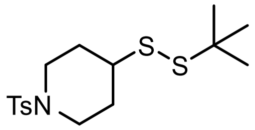

Prepared from **SI-2-1** (0.1 mmol, 1.0 equiv) and **SI-4-17** (0.25 mmol, 2.5 equiv) according to the general procedure 6. Purification by flash column (EtOAc/Petroleum Ether = 1/15) afforded **62** (25 mg, 72% yield) as a white solid.

Rf = 0.17 (EtOAc/Petroleum Ether = 1/50)

^{1}H NMR (400 MHz, $CDCl_3$) δ 7.67 – 7.60 (m, 2H), 7.35 – 7.29 (m, 2H), 3.54 (dt, *J* = 12.1, 4.5 Hz, 2H), 2.71 – 2.62 (m, 1H), 2.57 (ddd, *J* = 12.4, 9.9, 3.1 Hz, 2H), 2.42 (s, 3H), 2.09 (ddt, *J* = 12.8, 4.5, 2.7 Hz, 2H), 1.68 (dtd, *J* = 13.6, 9.8, 3.8 Hz, 2H), 1.27 (s, 9H).

^{13}C NMR (101 MHz, $CDCl_3$) δ 143.7, 133.4, 129.8, 127.8, 47.8, 46.6, 45.3, 31.1, 30.1, 21.7.

HRMS (ESI): m/z calculated for $C_{16}H_{25}NO_2S_3Na^+$ $[M+Na]^+$, 382.0940; found, 382.0946.

1-(*tert*-butyl)-2-(2,3-dihydro-1H-inden-2-yl)disulfane (63)

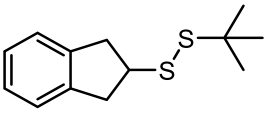

Prepared from **SI-2-6** (0.1 mmol, 1.0 equiv) and **SI-4-17** (0.25 mmol, 2.5 equiv) according to the general procedure 6. Purification by flash column (Petroleum Ether) afforded **63** (9 mg, 40% yield) as a colorless oli.

Rf = 0.57 (Petroleum Ether)

^{1}H NMR (400 MHz, CDCl3) δ 7.24 – 7.13 (m, 4H), 3.78 (tt, *J* = 7.3, 5.1 Hz, 1H), 3.33 (dd, *J* = 16.5, 7.4 Hz, 2H), 3.08 (dd, *J* = 16.5, 5.1 Hz, 2H), 1.37 (s, 9H).

^{13}C NMR (101 MHz, $CDCl_3$) δ 141.6, 126.8, 124.8, 49.8, 47.9, 39.8, 30.3.

HRMS (ESI): m/z calculated for $C_{13}H_{18}S_2Na^+$ $[M+Na]^+$, 261.0742; found, 261.0736.

1-(tert-butyl)-2-(4-phenylbutan-2-yl)disulfane (64)

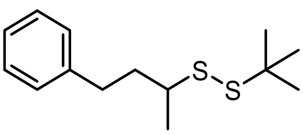

Prepared from **SI-2-7** (0.1 mmol, 1.0 equiv) and **SI-4-17** (0.25 mmol, 2.5 equiv) according to the general procedure 6. Purification by flash column (Petroleum Ether) afforded **64** (14 mg, 56% yield) as a colorless oli.

Rf = 0.48 (Petroleum Ether)

^{1}H NMR (400 MHz, CDCl3) δ 7.33 – 7.15 (m, 5H), 2.89 – 2.67 (m, 3H), 2.02 (ddt, *J* = 13.9, 9.5, 6.4 Hz, 1H), 1.86 – 1.74 (m, 1H), 1.33 (d, *J* = 6.8 Hz, 3H), 1.29 (s, 9H).

^{13}C NMR (101 MHz, $CDCl_3$) δ 141.8, 128.6, 128.5, 126.0, 47.6, 46.5, 38.2, 33.3, 30.2, 20.6.

HRMS (EI): m/z calculated for $C_{14}H_{22}S_2^+$ $[M]^+$, 254.1157; found, 254.1156.

methyl 2-(*tert*-butyldisulfanyl)-3-phenylpropanoate (65)

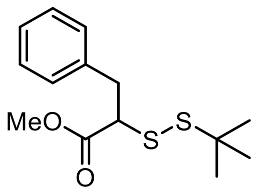

Prepared from **SI-2-8** (0.1 mmol, 1.0 equiv) and **SI-4-17** (0.25 mmol, 2.5 equiv) according to the general procedure 6. Purification by flash column (Petroleum Ether/Ethyl acetate = 1/20) afforded **65** (7 mg, 25% yield) as a white solid.

Rf = 0.76 (Petroleum Ether/Ethyl acetate = 1/10)

^{1}H NMR (400 MHz, $CDCl_3$) δ 7.32 – 7.25 (m, 2H), 7.25 – 7.16 (m, 3H), 3.67 (s, 3H), 3.66 – 3.63 (m, 1H), 3.19 (dd, *J* = 13.9, 9.4 Hz, 1H), 3.09 (dd, *J* = 13.9, 6.3 Hz, 1H), 1.30 (s, 9H).

^{13}C NMR (101 MHz, $CDCl_3$) δ 171.9, 137.8, 129.2, 128.7, 127.0, 57.0, 52.3, 48.5, 38.3, 30.0.

HRMS (ESI): m/z calculated for $C_{14}H_{20}O_2S_2Na^+$ $[M+Na]^+$, 307.0797; found, 307.0810.

1-tosylpiperidine (66)

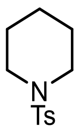

Prepared from **SI-1-1** (0.2 mmol, 1.0 equiv) according to the general procedure 7. Purification by flash column (Petroleum Ether/Ethyl acetate = 1/30) afforded **66** (41 mg, 85% yield) as a white solid.

Prepared from **SI-1-7** (0.2 mmol, 1.0 equiv) according to the general procedure 7. Purification by flash column (Petroleum Ether/Ethyl acetate = 1/30) afforded **69** (35 mg, 73% yield) as a white solid.

Rf = 0.30 (Petroleum Ether/Ethyl acetate = 1/30)

1H NMR (400 MHz, $CDCl_3$) δ = 7.64 (d, J=8.3, 2H), 7.32 (s, 2H), 3.01 – 2.93 (m, 4H), 2.43 (s, 3H), 1.70 – 1.60 (m, 4H), 1.46 – 1.37 (m, 2H).

^{13}C NMR (126 MHz, $CDCl_3$) δ 143.4, 133.4, 129.7, 127.9, 47.1, 25.3, 23.7, 21.7.

benzyl piperidine-1-carboxylate (67)

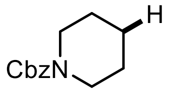

Prepared from **SI-1-4** (0.2 mmol, 1.0 equiv) according to the general procedure 7. Purification by flash column (Petroleum Ether/Ethyl acetate = 1/30) afforded **67** (37 mg, 84% yield) as a white solid.

Rf = 0.25 (Petroleum Ether/Ethyl acetate = 1/30)

1H NMR (500 MHz, $CDCl_3$) δ = 7.39 – 7.28 (m, 5H), 5.13 (s, 2H), 3.49 – 3.40 (m, 4H), 1.63 – 1.47 (m, 6H).

^{13}C NMR (126 MHz, $CDCl_3$) δ 155.5, 137.2, 128.6, 128.0, 127.9, 67.0, 45.0, 25.8, 24.5.

***tert*-butyl piperidine-1-carboxylate (68)**

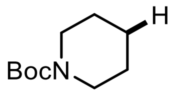

Prepared from **SI-1-3** (0.2 mmol, 1.0 equiv) according to the general procedure 7. Purification by flash column (Petroleum Ether/Ethyl acetate = 1/30) afforded **68** (22 mg, 60% yield) as a white solid.

Rf = 0.20 (Petroleum Ether/Ethyl acetate = 1/30)

^{1}H NMR (400 MHz, $CDCl_3$) δ = 3.37 – 3.32 (m, 4H), 1.55 (q, *J*=5.0, 4.2, 2H), 1.53 – 1.47 (m, 4H), 1.45 (s, 9H).

^{13}C NMR (126 MHz, $CDCl_3$) δ 155.1, 79.3, 45.0, 28.6, 25.9, 24.6.

***tert*-butyl 3,4-dihydroisoquinoline-2(1*H*)-carboxylate (70)**

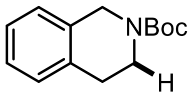

Prepared from **SI-1-9** (0.2 mmol, 1.0 equiv) according to the general procedure 7. Purification by flash column (Petroleum Ether/Ethyl acetate = 1/30) afforded **70** (24

mg, 51% yield) as yellow oil.

Rf = 0.30 (Petroleum Ether/Ethyl acetate = 1/30)

1H NMR (400 MHz, CDCl3) δ = 7.22 – 7.07 (m, 4H), 4.57 (s, 2H), 3.64 (d, J=5.5, 2H), 2.84 (t, J=6.0, 2H), 1.49 (s, 9H).

^{13}C NMR (101 MHz, $CDCl_3$) δ 155.1, 134.9, 128.8, 126.5, 126.32, 79.9, 45.7, 41.4, 29.1, 28.6.

4-(piperidine-1-carbonyl)-*N,N*-dipropylbenzenesulfonamide (71)

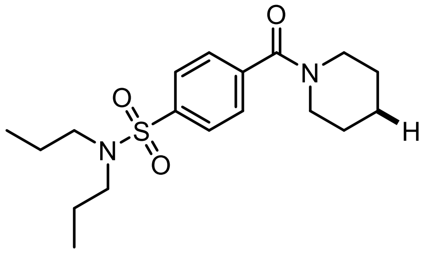

Prepared from **SI-1-5** (0.2 mmol, 1.0 equiv) according to the general procedure 7. Purification by flash column (Petroleum Ether/ Ethyl Acetate = 1/30) afforded **71** (60 mg, 85% yield) as a white solid.

Rf = 0.20 (Petroleum Ether/ Ethyl Acetate = 1/30)

^{1}H NMR (400 MHz, $CDCl_3$) δ 7.83 (d, *J* = 8.0 Hz, 2H), 7.49 (d, *J* = 8.0 Hz, 2H), 3.71 (brs, 2H), 3.26 (brs, 2H), 3.06 (dd, *J* = 8.9, 6.5 Hz, 4H), 1.68 (brs, 4H), 1.61 – 1.47 (m, 6H), 0.87 (t, *J* = 7.4 Hz, 6H).

^{13}C NMR (126 MHz, $CDCl_3$) δ 168.8, 141.1, 140.4, 127.4 (2C), 50.3, 48.8, 43.3, 26.6, 25.7, 24.6, 22.2, 11.3.

HRMS (ESI): *m/z* calculated for $C_{18}H_{28}N_2O_3SNa^+$ $[M+Na]^+$, 375.1713; found, 375.1709.

2-(piperidin-1-yl)pyrimidine (72)

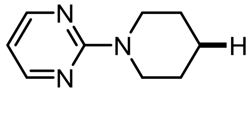

Prepared from **SI-1-6** (0.2 mmol, 1.0 equiv) according to the general procedure 7. Purification by flash column (Petroleum Ether/ Ethyl Acetate = 1/10) afforded **72** (26 mg, 80% yield) as a white solid.

Rf = 0.30 (Petroleum Ether/ Ethyl Acetate = 1/10)

^{1}H NMR (400 MHz, CDCl3) δ = 8.27 (d, *J*=4.8, 2H), 6.40 (t, *J*=4.7, 1H), 3.84 – 3.71 (m, 4H), 1.69 – 1.56 (m, 6H).

^{13}C NMR (126 MHz, $CDCl_3$) δ 161.8, 157.8, 109.2, 44.9, 25.9, 25.0.

1-chloro-4-cyclohexylbenzene (73)

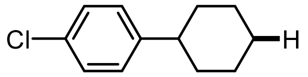

Prepared from **SI-1-14** (0.2 mmol, 1.0 equiv) according to the general procedure 7. Purification by flash column (Petroleum Ether) afforded **73** (32 mg, 82% yield) as yellow oil.

Rf = 0.90 (Petroleum Ether)

^{1}H NMR (500 MHz, CDCl3) δ = 7.24 (s, 2H), 7.18 – 7.09 (m, 2H), 2.48 (ddq, *J*=11.6, 8.7, 3.1, 1H), 1.84 (tq, *J*=11.1, 4.3, 4H), 1.78 – 1.71 (m, 1H), 1.39 (ddd, *J*=12.1, 7.5, 3.6, 4H), 1.30 – 1.18 (m, 1H).

^{13}C NMR (126 MHz, $CDCl_3$) δ 146.6, 131.4, 128.6, 128.5, 128.4, 128.3, 44.1 (2C), 44.1, 34.6, 26.9, 26.2.

9*H*-fluorene (74)

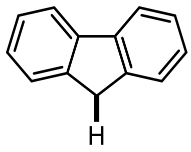

Prepared from **SI-1-16** (0.2 mmol, 1.0 equiv) according to the general procedure 7. Purification by flash column (Petroleum Ether) afforded **74** (20 mg, 60% yield) as a white solid.

Rf = 0.90 (Petroleum Ether)

^{1}H NMR (400 MHz, $CDCl_3$) δ = 7.81 (d, *J*=7.6, 2H), 7.56 (dt, *J*=7.5, 1.0, 2H), 7.39 (td, *J*=7.5, 1.0, 2H), 7.35 – 7.29 (m, 2H), 3.92 (s, 2H).

^{13}C NMR (126 MHz, $CDCl_3$) δ 143.3, 141.8, 127.1, 126.9, 126.8, 126.6, 125.2, 120.0, 37.1.

Hexylbenzene (75)

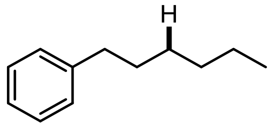

Prepared from **SI-1-19** (0.2 mmol, 1.0 equiv) according to the general procedure 7. Purification by flash column (Petroleum Ether) afforded **75** (20 mg, 62% yield) as oil.

Rf = 0.95 (Petroleum Ether)

^{1}H NMR (400 MHz, $CDCl_3$) δ = 7.33 – 7.24 (m, 2H), 7.19 (d, *J*=7.0, 3H), 2.65 – 2.57 (m, 2H), 1.66 – 1.58 (m, 2H), 1.38 – 1.28 (m, 6H), 0.93 – 0.85 (m, 3H).

^{13}C NMR (126 MHz, $CDCl_3$) δ 143.1, 128.5, 128.4, 125.7, 36.2, 31.9, 31.7, 29.2, 22.8, 14.3.

(4-methylpentyl)benzene (76)

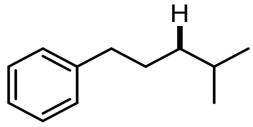

Prepared from **SI-1-20** (0.2 mmol, 1.0 equiv) according to the general procedure 7. Purification by flash column (Petroleum Ether) afforded **76** (21 mg, 65% yield) as colorless oil.

Rf = 0.95 (Petroleum Ether)

^{1}H NMR (400 MHz, $CDCl_3$) δ = 7.33 – 7.27 (m, 2H), 7.21 (d, *J*=7.3, 3H), 2.61 (t, *J*=7.8, 2H), 1.70 – 1.57 (m, 3H), 1.30 – 1.21 (m, 2H), 0.91 (dd, *J*=6.6, 0.7, 6H).

^{13}C NMR (126 MHz, $CDCl_3$) δ 143.1, 128.5, 128.4, 125.7, 38.8, 36.4, 29.5, 28.1, 22.8.

hex-5-en-1-ylbenzene (77)

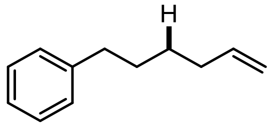

Prepared from **SI-1-18** (0.2 mmol, 1.0 equiv) according to the general procedure 7. Purification by flash column (Petroleum Ether) afforded **77** (18 mg, 56% yield) as colorless oil.

Rf = 0.95 (Petroleum Ether)

^{1}H NMR (400 MHz, $CDCl_3$) δ = 7.30 (d, *J*=7.6, 2H), 7.19 (dt, *J*=7.1, 2.8, 3H), 5.83 (ddt, *J*=16.9, 10.1, 6.7, 1H), 5.09 – 4.90 (m, 2H), 2.64 (t, *J*=7.8, 2H), 2.10 (q, *J*=7.0, 2H), 1.72 – 1.60 (m, 2H), 1.52 – 1.41 (m, 2H).

^{13}C NMR (126 MHz, $CDCl_3$) δ 142.8, 139.0, 128.5, 128.4, 125.8, 114.5, 36.0, 33.8, 31.1, 28.7

2-phenethylisoindoline-1,3-dione (78)

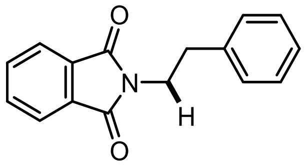

Prepared from **SI-1-21** (0.2 mmol, 1.0 equiv) according to the general procedure 7. Purification by flash column (Petroleum Ether/ Ethyl Acetate = 1/30) afforded **78** (35 mg, 70% yield) as a white solid.

Rf = 0.30 (Petroleum Ether/ Ethyl Acetate = 1/30)

^{1}H NMR (400 MHz, $CDCl_3$) δ 7.83 (dd, *J* = 5.4, 3.1 Hz, 2H), 7.70 (dd, *J* = 5.5, 3.1 Hz, 2H), 7.31 – 7.20 (m, 5H), 3.98 – 3.87 (m, 2H), 3.05 – 2.90 (m, 2H).

^{13}C NMR (126 MHz, $CDCl_3$) δ 168.3, 138.1, 134.0, 132.2, 129.0, 128.7, 126.8, 123.4, 39.4, 34.8.

2-decylisoindoline-1,3-dione (79)

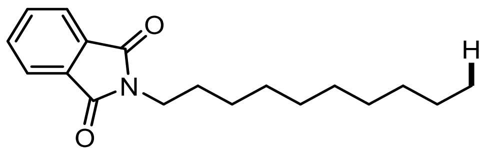

Prepared from **SI-1-24** (0.2 mmol, 1.0 equiv) according to the general procedure 7. Purification by flash column (Petroleum Ether/ Ethyl Acetate = 1/30) afforded **79** (35 mg, 61% yield) as a white solid.

Rf = 0.40 (Petroleum Ether/ Ethyl Acetate = 1/30)

^{1}H NMR (400 MHz, $CDCl_3$) δ = 7.82 (dt, *J*=7.3, 3.7, 2H), 7.69 (dd, *J*=5.5, 3.1, 2H), 3.66 (t, *J*=7.3, 2H), 1.28 (d, *J*=30.8, 16H), 0.86 (t, *J*=6.8, 3H).

^{13}C NMR (126 MHz, $CDCl_3$) δ 167.6, 132.9, 131.3, 122.3, 37.2, 31.0, 28.6 (2C), 28.4, 28.3, 27.7, 26.0, 21.8, 13.2.

1-(4-bromobutoxy)-4-ethylbenzene (80)

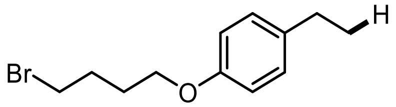

Prepared from **SI-1-39** (0.2 mmol, 1.0 equiv) according to the general procedure 7. Purification by flash column (Petroleum Ether) afforded **80** (36 mg, 70% yield) as oil.

Rf = 0.33 (Petroleum Ether)

^{1}H NMR (500 MHz, Chloroform-*d*) δ 7.11 (d, *J* = 8.5 Hz, 2H), 6.82 (d, *J* = 8.5 Hz, 2H), 3.98 (t, *J* = 6.1 Hz, 2H), 3.49 (t, *J* = 6.6 Hz, 2H), 2.59 (q, *J* = 7.6 Hz, 2H), 2.11 – 2.03 (m, 2H), 1.94 (dt, *J* = 8.9, 6.1 Hz, 2H), 1.21 (t, *J* = 7.6 Hz, 3H).

^{13}C NMR (126 MHz, $CDCl_3$) δ 157.01, 136.65, 128.85, 114.46, 66.97, 33.68, 29.66, 28.11, 16.04.

butane-2, 2-diylbis(4,1-phenylene) diacetate (81)

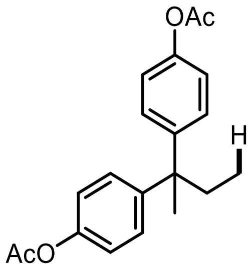

Prepared from **SI-1-27** (0.2 mmol, 1.0 equiv) according to the general procedure 7. Purification by flash column (Petroleum Ether/ Ethyl Acetate = 1/10) afforded **80** (40 mg, 62% yield) as a white solid.

Rf = 0.30 (Petroleum Ether/ Ethyl Acetate = 1/10)

^{1}H NMR (400 MHz, $CDCl_3$) δ = 7.23 – 7.12 (m, 4H), 7.03 – 6.94 (m, 4H), 2.11 (q, *J*=7.3, 2H), 1.59 (s, 3H), 0.74 (t, *J*=7.3, 3H).

^{13}C NMR (101 MHz, $CDCl_3$) δ 169.6, 148.6, 146.9, 128.5, 120.9, 46.2, 34.4, 27.3, 21.3, 9.3.

(*S*)-2-(2,4-dimethylpentyl)isoindoline-1,3-dione (82)

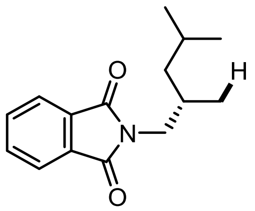

Prepared from **SI-1-35** (0.2 mmol, 1.0 equiv) according to the general procedure 7. Purification by flash column (Petroleum Ether/ Ethyl Acetate = 1/30) afforded **81** (30 mg, 61% yield) as a white solid.

Rf = 0.30 (Petroleum Ether/ Ethyl Acetate = 1/30)

^{1}H NMR (400 MHz, $CDCl_3$) δ 7.84 (dd, *J* = 5.5, 3.1 Hz, 2H), 7.70 (dd, *J* = 5.4, 3.0 Hz, 2H), 3.56 (dd, *J* = 13.4, 6.3 Hz, 1H), 3.46 (dd, *J* = 13.4, 8.3 Hz, 1H), 2.11 – 1.99 (m, 1H), 1.75 – 1.64 (m, 1H), 1.21 – 1.05 (m, 2H), 0.91 (d, *J* = 6.6 Hz, 3H), 0.87 (d, *J* = 6.7 Hz, 3H), 0.83 (d, *J* = 6.5 Hz, 3H).

^{13}C NMR (126 MHz, $CDCl_3$) δ 168.9, 134.0, 132.2, 123.3, 44.6, 44.0, 30.5, 25.3, 23.6, 22.1, 17.8.

HRMS (ESI): *m/z* calculated for $C_{20}H_{22}O_4Na^+$ $[M+Na]^+$, 349.1410; found, 349.1396.

2,4-dichloro-1-methoxybenzene (83)

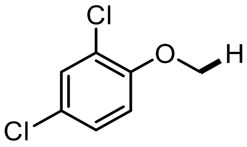

Prepared from **SI-1-28** (0.2 mmol, 1.0 equiv) according to the general procedure 7. Purification by flash column (Petroleum Ether) afforded **82** (16 mg, 45% yield) as yellow oil.

Rf = 0.70 (Petroleum Ether)

^{1}H NMR (400 MHz, $CDCl_3$) δ = 7.36 (d, *J*=2.6, 1H), 7.21 – 7.17 (m, 1H), 6.84 (d, *J*=8.8, 1H), 3.88 (s, 3H).

^{13}C NMR (101 MHz, $CDCl_3$) δ 154.0, 130.1, 127.7, 125.8, 123.4, 112.9, 56.5.

1,4-dimethyl-2-((4-methylpentyl)oxy)benzene (84)

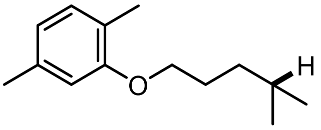

Prepared from **SI-1-37** (0.2 mmol, 1.0 equiv) according to the general procedure 7. Purification by flash column (Petroleum Ether) afforded **83** (33 mg, 80% yield) as a white solid.

Rf = 0.70 (Petroleum Ether)

^{1}H NMR (400 MHz, $CDCl_3$) δ = 7.02 (d, *J*=7.5, 1H), 6.72 – 6.62 (m, 2H), 3.95 (t, *J*=6.5, 2H), 2.33 (s, 3H), 2.20 (s, 3H), 1.82 (ddt, *J*=10.3, 8.2, 6.4, 2H), 1.65 (dq, *J*=13.3, 6.7, 1H), 1.43 – 1.33 (m, 2H), 0.95 (d, *J*=6.6, 6H).

^{13}C NMR (126 MHz, $CDCl_3$) δ 157.3, 136.6, 130.4, 123.8, 120.7, 112.1 (2C), 68.3, 35.5, 27.9, 27.4, 22.7, 21.6, 15.9.

(2-cyclohexylethyl)benzene (85)

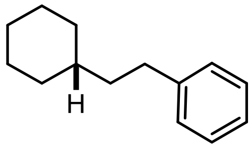

Prepared from **SI-1-32** (0.2 mmol, 1.0 equiv) according to the general procedure 7. Purification by flash column (Petroleum Ether) afforded **84** (35 mg, 93% yield) as colorless oil.

Rf = 0.98 (Petroleum Ether)

^{1}H NMR (400 MHz, $CDCl_3$) δ 7.28 (td, *J* = 7.2, 1.8 Hz, 2H), 7.22 – 7.13 (m, 3H), 2.65 – 2.58 (m, 2H), 1.81 – 1.62 (m, 5H), 1.53 – 1.47 (m, 2H), 1.34 – 1.11 (m, 4H), 1.01 – 0.88 (m, 2H).

^{13}C NMR (126 MHz, $CDCl_3$) δ 143.4, 128.5, 128.4, 125.6, 39.6, 37.5, 33.5, 33.4, 26.9, 26.5.

(*E*)-4-styryl-1-tosylpiperidine (85)

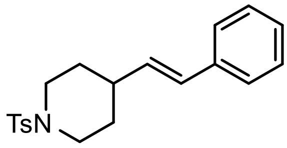

Prepared from **SI-1-1** (0.2 mmol, 1.0 equiv) and **SI-5-1** (0.6 mmol, 3 equiv) according to the general procedure 8. Purification by flash column (Petroleum Ether/ Ethyl Acetate = 1/10) afforded **85** (39 mg, 59% yield) as a white solid.

Rf = 0.40 (Petroleum Ether/ Ethyl Acetate = 1/10)

^{1}H NMR (400 MHz, $CDCl_3$) δ 7.65 (t, *J* = 8.9 Hz, 2H), 7.35 – 7.27 (m, 6H), 7.22 – 7.18 (m, 1H), 6.34 (d, *J* = 16.0 Hz, 1H), 6.08 (dd, *J* = 16.0, 6.9 Hz, 1H), 3.81 (dt, *J* = 12.7, 4.3 Hz, 2H), 2.97 (t, *J* = 5.6 Hz, 1H), 2.44 (d, *J* = 4.6 Hz, 3H), 2.34 (td, *J* = 11.9, 2.7 Hz, 2H), 1.86 – 1.79 (m, 2H), 1.67 – 1.59 (m, 2H).

^{13}C NMR (126 MHz, $CDCl_3$) δ 143.6, 137.3, 133.6, 133.3, 129.8, 129.1, 128.7, 127.9, 127.4, 126.2, 46.3, 38.7, 31.4, 21.7.

(*E*)-4-(4-methylstyryl)-1-tosylpiperidine (86)

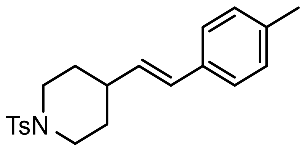

Prepared from **SI-1-1** (0.2 mmol, 1.0 equiv) and **SI-5-2** (0.6 mmol, 3.0 equiv) according to the general procedure 8. Purification by flash column (Petroleum Ether/ Ethyl Acetate = 1/10) afforded **86** (36 mg, 52% yield) as a white solid.

Rf = 0.40 (Petroleum Ether/ Ethyl Acetate = 1/10)

^{1}H NMR (400 MHz, CDCl3) δ = 7.68 – 7.63 (m, 2H), 7.33 (d, *J*=8.3, 2H), 7.21 (d, *J*=8.1, 2H), 7.09 (d, *J*=8.2, 2H), 6.30 (d, *J*=16.0, 1H), 6.02 (dd, *J*=16.0, 7.0, 1H), 3.86 – 3.74 (m, 2H), 2.44 (s, 3H), 2.35 – 2.27 (m, 5H), 2.03 (dt, *J*=11.5, 6.4, 1H), 1.86 – 1.77 (m, 2H), 1.65 – 1.58 (m, 2H).

^{13}C NMR (126 MHz, $CDCl_3$) δ 143.6, 137.2, 134.5, 133.3, 132.5, 129.7, 129.4, 128.9, 127.9, 126.1, 46.3, 38.7, 31.5, 21.7, 21.3.

(*E*)-4-(4-methoxystyryl)-1-tosylpiperidine (87)

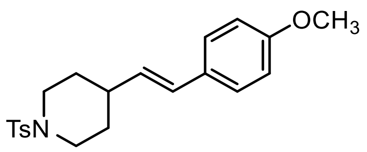

Prepared from **SI-1-1** (0.2 mmol, 1.0 equiv) and **SI-5-3** (0.6 mmol, 3.0 equiv) according to the general procedure 8. Purification by flash column (Petroleum Ether/ Ethyl Acetate = 1/10) afforded **87** (31 mg, 42% yield) as a white solid.

Rf = 0.40 (Petroleum Ether/ Ethyl Acetate = 1/10)

^{1}H NMR (400 MHz, CDCl3) δ 7.66 (d, *J* = 8.3 Hz, 2H), 7.33 (d, *J* = 8.2 Hz, 2H), 7.29 – 7.21 (m, 2H), 6.87 – 6.78 (m, 2H), 6.27 (d, *J* = 16.0 Hz, 1H), 5.94 (dd, *J* = 16.0, 6.9

Hz, 1H), 3.83 – 3.75 (m, 5H), 2.44 (s, 3H), 2.32 (td, *J* = 11.9, 2.8 Hz, 2H), 2.07 – 1.97 (m, 1H), 1.84 – 1.76 (m, 2H), 1.60 – 1.50 (m, 2H).

^{13}C NMR (126 MHz, $CDCl_3$) δ 159.0, 143.5, 133.3, 131.4, 130.1, 129.7, 128.4, 127.8, 127.2, 114.1, 55.4, 46.3, 38.6, 31.5, 21.6.

HRMS (ESI): *m/z* calculated for $C_{21}H_{25}NO_3SNa^+$ $[M+Na]^+$, 394.1447; found, 394.1445.

methyl (*E*)-4-(2-(1-tosylpiperidin-4-yl)vinyl)benzoate (88)

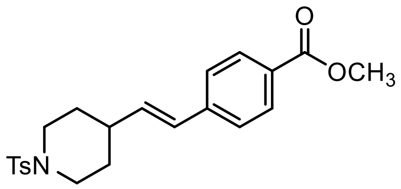

Prepared from **SI-1-1** (0.2 mmol, 1.0 equiv) and **SI-5-4** (0.6 mmol, 3.0 equiv) according to the general procedure 8. Purification by flash column (Petroleum Ether/ Ethyl Acetate = 1/10) afforded **88** (48 mg, 60% yield) as a white solid.

Rf = 0.20 (Petroleum Ether/ Ethyl Acetate = 1/10)

^{1}H NMR (500 MHz, CDCl3) δ = 7.95 (d, *J*=8.2, 2H), 7.66 (d, *J*=8.1, 2H), 7.35 (dd, *J*=15.3, 8.2, 4H), 6.36 (d, *J*=15.9, 1H), 6.21 (dd, *J*=15.9, 6.9, 1H), 3.90 (s, 3H), 3.83 (dd, *J*=9.6, 6.2, 2H), 2.44 (s, 3H), 2.32 (td, *J*=11.9, 2.6, 2H), 2.09 (tdd, *J*=11.6, 7.2, 3.5, 1H), 1.83 (dd, *J*=13.7, 3.4, 2H).

^{13}C NMR (126 MHz, $CDCl_3$) δ 167.0, 143.6, 141.8, 136.4, 133.2, 130.1, 129.8, 128.9, 128.4, 127.9, 126.1, 52.2, 46.2, 38.8, 31.3, 21.7.

HRMS (ESI): *m/z* calculated for $C_{22}H_{25}NO_4SNa^+$ $[M+Na]^+$, 422.1397; found, 422.1399.

(*E*)-1-tosyl-4-(4-(trifluoromethyl)styryl)piperidine (89)

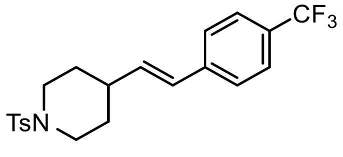

Prepared from **SI-1-1** (0.2 mmol, 1.0 equiv) and **SI-5-5** (0.6 mmol, 3.0 equiv) according to the general procedure 8. Purification by flash column (Petroleum Ether/ Ethyl Acetate = 1/10) afforded **89** (33 mg, 41% yield) as a white solid.

Rf = 0.21 (Petroleum Ether/ Ethyl Acetate = 1/10)

^{1}H NMR (400 MHz, $CDCl_3$) δ = 7.70 – 7.64 (m, 2H), 7.53 (d, *J*=8.4, 2H), 7.40 (d, *J*=8.3, 2H), 7.33 (d, *J*=8.3, 2H), 6.36 (d, *J*=16.0, 1H), 6.19 (dd, *J*=16.0, 6.8, 1H), 3.83 (dt, *J*=13.1, 4.3, 2H), 2.44 (s, 3H), 2.33 (td, *J*=11.9, 2.6, 2H), 2.14 - 2.03 (m, 1H), 1.87 – 1.80 (m, 2H), 1.63 (dd, *J*=11.9, 4.2, 2H).

^{13}C NMR (101 MHz, $CDCl_3$) δ 143.64, 140.84, 136.31, 133.36, 129.77, 128.00, 127.87, 126.35, 125.68, 125.63, 125.60, 125.56, 46.20, 38.75, 31.27, 21.67.

^{19}F NMR (376 MHz, $CDCl_3$) δ -62.46.

(*E*)-4-(2-(1-tosylpiperidin-4-yl)vinyl)benzonitrile (90)

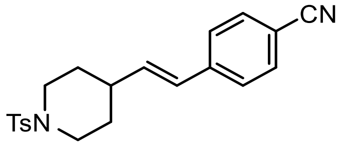

Prepared from **SI-1-1** (0.2 mmol, 1.0 equiv) and **SI-5-6** (0.6 mmol, 3.0 equiv) according to the general procedure 8. Purification by flash column (Petroleum Ether/ Ethyl Acetate = 1/10) afforded **90** (36 mg, 49% yield) as a white solid.

Rf = 0.20 (Petroleum Ether/ Ethyl Acetate = 1/10)

^{1}H NMR (500 MHz, $CDCl_3$) δ = 7.65 (d, *J*=8.1, 2H), 7.56 (d, *J*=8.2, 2H), 7.39 (d, *J*=8.2, 2H), 7.33 (d, *J*=8.1, 2H), 6.34 (d, *J*=16.2, 1H), 6.23 (dd, *J*=16.0, 6.9, 1H), 3.84 (d, *J*=11.7, 2H), 2.44 (s, 3H), 2.31 (t, *J*=11.6, 2H), 2.09 (h, *J*=7.2, 1H), 1.83 (d, *J*=13.0, 2H), 1.62 (d, *J*=8.9, 2H).

^{13}C NMR (126 MHz, $CDCl_3$) δ 143.7, 141.8, 137.7, 133.2, 132.5, 129.8, 127.9, 127.8, 126.7, 119.1, 110.6, 46.2, 38.8, 31.2, 21.7.

(*E*)-3-styryl-1-tosylazetidine (91)

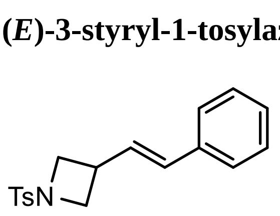

Prepared from **SI-1-10** (0.2 mmol, 1.0 equiv) and **SI-5-1** (0.6 mmol, 3.0 equiv) according to the general procedure 8. Purification by flash column (Petroleum Ether/ Ethyl Acetate = 1/10) afforded **91** (20 mg, 32% yield) as a white solid.

Rf = 0.40 (Petroleum Ether/ Ethyl Acetate = 1/10)

^{1}H NMR (400 MHz, CDCl3) δ 7.79 – 7.75 (m, 2H), 7.40 (d, *J* = 8.3 Hz, 2H), 7.30 – 7.24 (m, 4H), 7.23 – 7.21 (m, 1H), 6.27 (d, *J* = 15.8 Hz, 1H), 6.01 (dd, *J* = 15.8, 8.4 Hz, 1H), 4.01 (t, *J* = 8.2 Hz, 2H), 3.64 (dd, *J* = 8.2, 6.5 Hz, 2H), 3.29 – 3.18 (m, 1H), 2.47 (s, 3H).

^{13}C NMR (126 MHz, $CDCl_3$) δ 144.3, 136.5, 132.0, 131.7, 129.9, 128.8, 128.7, 128.6, 127.9, 126.3, 56.4, 31.7, 21.8.

HRMS (ESI): *m/z* calculated for $C_{18}H_{19}NO_2SNa^+$ $[M+Na]^+$, 336.1029; found, 336.1033.

(*E*)-2-(4-styrylpiperidin-1-yl)pyrimidine (92)

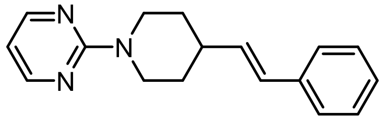

Prepared from **SI-1-6** (0.2 mmol, 1.0 equiv) and **SI-5-1** (0.6 mmol, 3.0 equiv) according to the general procedure 8. Purification by flash column (Petroleum Ether/ Ethyl Acetate = 1/20) afforded **92** (22 mg, 42% yield) as a white solid.

Rf = 0.30 (Petroleum Ether/ Ethyl Acetate = 1/20)

^{1}H NMR (400 MHz, CDCl3) δ 8.30 (d, *J* = 4.8 Hz, 2H), 7.36 – 7.26 (m, 4H), 7.22 – 7.17 (m, 1H), 6.46 – 6.38 (m, 2H), 6.18 (dd, *J* = 15.9, 6.8 Hz, 1H), 4.81 – 4.73 (m, 2H), 3.02 – 2.92 (m, 2H), 2.48 – 2.36 (m, 1H), 1.91 – 1.83 (m, 2H), 1.53 – 1.40 (m, 2H).

^{13}C NMR (126 MHz, $CDCl_3$) δ 161.8, 157.9, 137.7, 134.7, 128.6, 128.5, 127.2, 126.2, 109.5, 44.0, 39.9, 31.9.

HRMS (ESI): *m/z* calculated for $C_{17}H_{20}N_3^+$ $[M+H]^+$, 266.1652; found, 266.1649.

(*E*)-2-(1,4-diphenylbut-3-en-2-yl)isoindoline-1,3-dione (93)

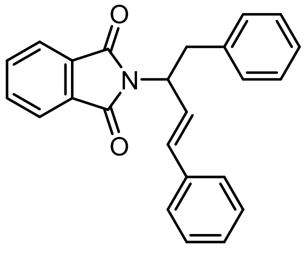

Prepared from **SI-1-21** (0.2 mmol, 1.0 equiv) and **SI-5-1** (0.6 mmol, 3.0 equiv)

according to the general procedure 8. Purification by flash column (Petroleum Ether/ Ethyl Acetate = 1/20) afforded **93** (15 mg, 17% yield) as a white solid.

Rf = 0.30 (Petroleum Ether/ Ethyl Acetate = 1/20)

^{1}H NMR (400 MHz, $CDCl_3$) δ 7.76 (dd, *J* = 5.5, 3.1 Hz, 2H), 7.68 – 7.63 (m, 2H), 7.38 – 7.34 (m, 2H), 7.31 – 7.24 (m, 3H), 7.24 – 7.19 (m, 5H), 6.70 (dd, *J* = 15.9, 7.9 Hz, 1H), 6.58 (d, *J* = 15.9 Hz, 1H), 5.23 – 5.16 (m, 1H), 3.52 (dd, *J* = 13.8, 9.8 Hz, 1H), 3.29 (dd, *J* = 13.8, 6.5 Hz, 1H).

^{13}C NMR (126 MHz, $CDCl_3$) δ 168.1, 137.6, 136.4, 134.0, 133.9, 133.3, 131.9, 129.2, 128.7, 128.6, 128.1, 126.7, 126.6, 123.3, 55.1, 39.0.

HRMS (ESI): *m/z* calculated for $C_{24}H_{19}NO_2Na^+$ $[M+Na]^+$, 376.1308; found, 376.1307

(*E*)-2-(12-phenyldodec-11-en-1-yl)isoindoline-1,3-dione (94)

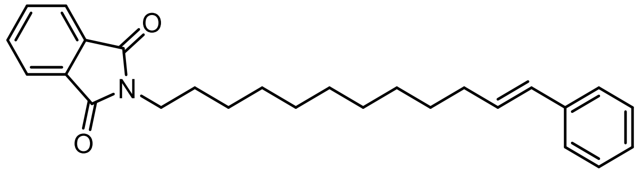

Prepared from **SI-1-24** (0.2 mmol, 1.0 equiv) and **SI-5-1** (0.6 mmol, 3.0 equiv) according to the general procedure 8. Purification by flash column (Petroleum Ether/ Ethyl Acetate = 1/10) afforded **94** (26 mg, 33% yield) as a white solid.

Rf = 0.40 (Petroleum Ether/ Ethyl Acetate = 1/10)

^{1}H NMR (400 MHz, CDCl3) δ = 7.84 (dd, *J*=5.4, 3.1, 2H), 7.70 (dd, *J*=5.5, 3.1, 2H), 7.34 (d, *J*=7.0, 2H), 7.29 (d, *J*=7.3, 2H), 7.20 – 7.15 (m, 1H), 6.37 (d, *J*=15.9, 1H), 3.67 (t, *J*=7.3, 2H), 2.24 – 2.15 (m, 2H), 1.72 - 1.62 (m, 2H), 1.49 – 1.42 (m, 2H), 1.34 – 1.27 (m, 10H).

^{13}C NMR (101 MHz, $CDCl_3$) δ 168.6, 138.1, 134.0, 132.3, 131.4, 129.8, 128.6, 126.8, 126.0, 123.3, 38.2, 33.2, 29.6, 29.6, 29.5, 29.4, 29.3, 28.7, 27.0.

HRMS (ESI): *m/z* calculated for $C_{26}H_{31}NO_2Na^+$ $[M+Na]^+$, 412.2247; found, 412.2249.

(*S*,*E*)-2-(2-isobutyl-5-phenylpent-4-en-1-yl)isoindoline-1,3-dione (95)

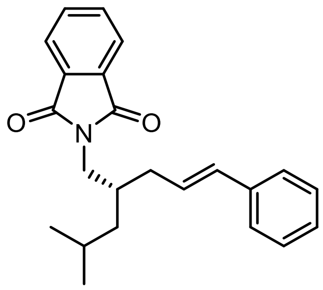

Prepared from **SI-1-35** (0.2 mmol, 1.0 equiv) and **SI-5-1** (0.6 mmol, 3.0 equiv) according to the general procedure 8. Purification by flash column (Petroleum Ether/ Ethyl Acetate = 1/10) afforded **95** (17 mg, 24% yield) as a white solid.

Rf = 0.30 (Petroleum Ether/ Ethyl Acetate = 1/10)

^{1}H NMR (400 MHz, $CDCl_3$) δ = 7.78 (dd, *J*=5.5, 3.1, 2H), 7.63 (dd, *J*=5.4, 3.1, 2H), 7.25 – 7.19 (m, 4H), 7.16 – 7.11 (m, 1H), 6.38 – 6.32 (m, 1H), 6.16 (dt, *J*=15.8, 6.9, 1H), 3.62 (t, *J*=7.2, 2H), 2.50 - 2.20 (m, 2H), 1.76 (dt, *J*=13.4, 6.7, 1H), 1.32 – 1.24 (m, 2H), 1.15 (dt, *J*=13.9, 6.9, 1H), 0.94 (d, *J*=6.5, 3H), 0.89 (d, *J*=6.6, 3H).

^{13}C NMR (126 MHz, $CDCl_3$) δ 168.9, 137.6, 133.9, 132.2, 131.7, 128.5, 128.2, 126.9, 126.0, 123.3, 42.6, 41.8, 36.2, 35.0, 25.5, 23.0, 22.8.

HRMS (ESI): *m/z* calculated for $C_{23}H_{25}NO_2Na^+$ $[M+Na]^+$, 370.1778; found, 370.1774.

methyl (*E*)-4-styrylbicyclo[2.2.2]octane-1-carboxylate (96)

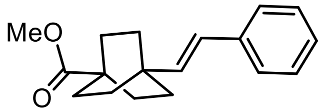

Prepared from **SI-1-33** (0.2 mmol, 1.0 equiv) and **SI-5-1** (0.6 mmol, 3.0 equiv) according to the general procedure 8. Purification by flash column (Petroleum Ether/ Ethyl Acetate = 1/50) afforded **96** (21 mg, 39% yield) as a white solid.

Rf = 0.40 (Petroleum Ether/ Ethyl Acetate = 1/50)

^{1}H NMR (400 MHz, $CDCl_3$) δ 7.35 – 7.25 (m, 5H), 7.20 – 7.15 (m, 1H), 6.24 (d, *J* = 16.4 Hz, 1H), 6.12 (d, *J* = 16.4 Hz, 1H), 3.66 (s, 3H), 1.89 – 1.81 (m, 6H), 1.61 (s, 6H).

^{13}C NMR (126 MHz, $CDCl_3$) δ 178.6, 139.3, 138.0, 128.6, 127.0, 126.1, 126.0, 51.8, 39.3, 33.4, 30.9, 28.5.

4-(2-([1,1'-biphenyl]-4-yl)-3,3-difluoroallyl)-1-tosylpiperidine (97)

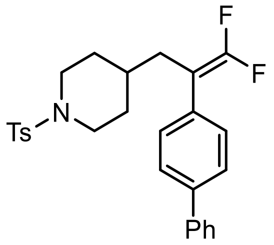

Prepared from **SI-2-1** (0.26 mmol, 1.3 equiv) and **SI-6-1** (0.2 mmol, 1.0 equiv) according to the general procedure 9. Purification by flash column (Petroleum Ether/ Ethyl Acetate = 1/20) afforded **97** (89 mg, 95% yield) as a white solid.

Rf = 0.27 (Petroleum Ether/ Ethyl Acetate = 1/10)

^{1}H NMR (400 MHz, CDCl3) δ 7.62 – 7.51 (m, 6H), 7.43 (dd, *J* = 8.4, 6.8 Hz, 2H), 7.38 – 7.24 (m, 5H), 3.74 (dt, *J* = 12.1, 3.4 Hz, 2H), 2.39 (s, 3H), 2.35 (dt, *J* = 7.2, 2.4 Hz, 2H), 2.10 (td, *J* = 11.8, 2.5 Hz, 2H), 1.70 (dd, *J* = 13.3, 3.5 Hz, 2H), 1.43 – 1.17 (m, 4H), 0.92 – 0.81 (m, 1H).

^{13}C NMR (126 MHz, $CDCl_3$) δ154.25 (dd, *J* = 291.6, 287.2 Hz), 143.51, 140.46, 140.31, 133.17, 132.37 (t, *J* = 3.8 Hz), 129.67, 128.96, 128.57 (t, *J* = 3.3 Hz), 127.83, 127.63, 127.32, 127.08, 90.12 (dd, *J* = 21.9, 13.2 Hz), 46.38, 33.96, 33.58 (t, *J* = 2.5 Hz), 31.21, 21.62.

^{19}F NMR (376 MHz, CDCl3) δ -89.88 (d, *J* = 41.3 Hz), -90.28 (d, *J* = 41.3 Hz).

HRMS (ESI) m/z calculated for $C_{27}H_{28}F_2NO_2S^+$ $[M+H]^+$, 468.1803; found, 468.1859.

4-(3,3-difluoro-2-(3-methoxyphenyl)allyl)-1-tosylpiperidine (98)

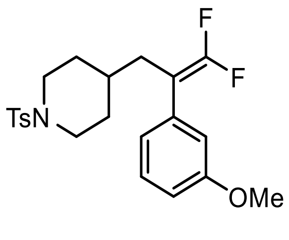

Prepared from **SI-2-1** (0.26 mmol, 1.3 equiv) and **SI-6-6** (0.2 mmol, 1.0 equiv) according to the general procedure 9. Purification by flash column (Petroleum Ether/ Ethyl Acetate = 1/20) afforded **98** (57 mg, 89% yield) as a white solid.

Rf = 0.4 (Petroleum Ether/ Ethyl Acetate = 1/5)

^{1}H NMR (400 MHz, CDCl3) δ 7.59 (d, *J* = 8.0 Hz, 2H), 7.28 (d, *J* = 8.0 Hz, 2H), 7.23 (t, *J* = 7.8 Hz, 1H), 6.85 – 6.76 (m, 3H), 3.78 (s, 3H), 3.72 (dd, *J* = 11.8, 3.3 Hz, 2H), 2.40 (s, 3H), 2.29 (dt, *J* = 7.0, 2.3 Hz, 2H), 2.09 (td, *J* = 11.8, 2.5 Hz, 2H), 1.67 (d, *J* = 12.9 Hz, 2H), 1.38 – 1.25 (m, 2H), 1.23 – 1.11 (m, 1H).

^{13}C NMR (101 MHz, $CDCl_3$) δ 159.68, 154.15 (dd, *J* = 291.1, 287.4 Hz), 143.52, 134.85 (t, *J* = 3.3 Hz), 133.10, 129.66, 129.62, 127.81, 120.64 (t, *J* = 3.0 Hz), 114.50 (t, *J* = 3.3 Hz), 112.45, 90.33 (dd, *J* = 21.2, 14.0 Hz), 55.35, 46.36, 34.08, 33.51 (dd, *J* = 2.7, 2.5 Hz), 31.16, 21.60.

^{19}F NMR (376 MHz, CDCl3) δ -90.12 (d, *J* = 41.5 Hz), -90.31 (d, *J* = 41.5 Hz).

HRMS (ESI) m/z calculated for $C_{22}H_{26}F_2NO_3S^+$ $[M+H]^+$, 422.1596; found, 422.1592.

4-(2-(3,4-dichlorophenyl)-3,3-difluoroallyl)-1-tosylpiperidine (99)

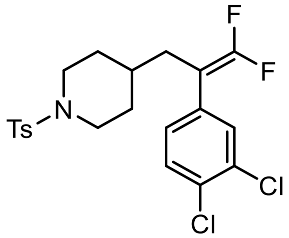

Prepared from **SI-2-1** (0.26 mmol, 1.3 equiv) and **SI-6-9** (0.2 mmol, 1.0 equiv) according to the general procedure 9. Purification by flash column (Petroleum Ether/ Ethyl Acetate = 1/20) afforded **99** (70 mg, 76% yield) as a white solid.

Rf = 0.30 (Petroleum Ether/ Ethyl Acetate = 1/10)

^{1}H NMR (400 MHz, $CDCl_3$) δ 7.59 (d, *J* = 8.1 Hz, 2H), 7.38 (d, *J* = 8.3 Hz, 1H), 7.32 (d, *J* = 2.2 Hz, 1H), 7.28 (d, *J* = 8.0 Hz, 2H), 7.08 (dt, *J* = 8.5, 1.6 Hz, 1H), 3.73 (dt, *J* = 11.8, 3.4 Hz, 2H), 2.40 (s, 3H), 2.28 (dt, *J* = 7.2, 2.4 Hz, 2H), 2.10 (td, *J* = 11.9, 2.5 Hz, 2H), 1.71 – 1.60 (m, 2H), 1.42 – 1.22 (m, 2H), 1.21 – 1.08 (m, 1H).

^{13}C NMR (126 MHz, $CDCl_3$) δ154.34 (dd, *J* = 293.2, 288.7 Hz), 143.60, 133.53 (dd, *J* = 4.4, 3.1 Hz), 133.09, 132.91, 131.66, 130.66, 130.03 (t, *J* = 3.5 Hz), 129.72, 127.82, 127.54 (t, *J* = 3.2 Hz), 89.08 (dd, *J* = 23.3, 13.0 Hz), 46.30, 33.79, 33.52 (dd, *J* = 2.8, 1.6 Hz), 31.14, 21.64.

^{19}F NMR (471 MHz, $CDCl_3$) δ -88.27 (d, *J* = 37.4 Hz), -88.65 (d, *J* = 37.5 Hz).

HRMS (ESI) m/z calculated for $C_{21}H_{22}Cl_2F_2NO_2S$ $[M+H]^+$, 460.0711; found,

460.0698.

4-(2-(3-(benzyloxy)phenyl)-3,3-difluoroallyl)-1-tosylpiperidine (100)

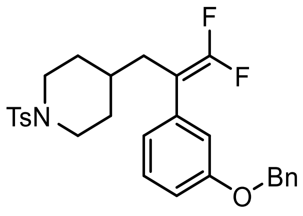

Prepared from **SI-1-x** (0.26 mmol, 1.3 equiv) and SI-(0.2 mmol, 1.0 equiv) according to the general procedure 9. Purification by flash column (Petroleum Ether/ Ethyl Acetate = 1/20) afforded **100** (65 mg, 65% yield) as a white solid.

Rf = 0.30 (Petroleum Ether/ Ethyl Acetate = 1/10)

^{1}H NMR (400 MHz, CDCl3) δ 7.59 (d, *J* = 8.1 Hz, 2H), 7.43 – 7.20 (m, 8H), 6.89 – 6.81 (m, 3H), 5.04 (s, 2H), 3.70 (dt, *J* = 12.2, 3.4 Hz, 2H), 2.40 (s, 3H), 2.27 (dt, *J* = 7.3, 2.4 Hz, 2H), 2.07 (td, *J* = 11.9, 2.5 Hz, 2H), 1.67 – 1.59 (m, 2H), 1.33 – 1.25 (m, 2H), 1.20 – 1.05 (m, 1H).

^{13}C NMR (126 MHz, $CDCl_3$) δ 158.86, 154.14 (dd, *J* = 290.9, 287.2 Hz), 143.51, 136.85, 134.83 (t, *J* = 3.2 Hz), 133.12, 129.66, 128.71, 128.16, 127.81, 127.59, 120.93 (t, *J* = 3.1 Hz), 115.26 (t, *J* = 3.2 Hz), 113.61, 90.29 (dd, *J* = 21.0, 13.8 Hz), 70.14, 46.34, 34.02, 33.46, 31.11, 21.60.

^{19}F NMR (471 MHz, CDCl3) δ -90.04 (d, *J* = 41.1 Hz), -90.20 (d, *J* = 41.1 Hz).

HRMS (ESI) m/z calculated for $C_{28}H_{30}F_2NO_3S^+$ $[M+H]^+$, 498.1909; found, 498.1901.

4-(3,3-difluoro-2-(4-methoxyphenyl)allyl)-1-tosylpiperidine (101)

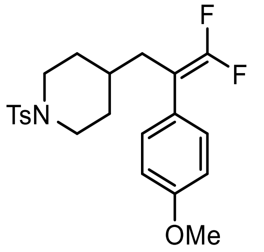

Prepared from **SI-2-1** (0.26 mmol, 1.3 equiv) and **SI-6-3** (0.2 mmol, 1.0 equiv) according to the general procedure 9. Purification by flash column (Petroleum Ether/ Ethyl Acetate = 1/20) afforded **101** (51 mg, 60% yield) as a white solid.

Rf = 0.20 (Petroleum Ether/ Ethyl Acetate = 1/10)

^{1}H NMR (400 MHz, CDCl3) δ 7.59 (d, *J* = 8.0 Hz, 2H), 7.31 – 7.24 (m, 2H), 7.19 – 7.12 (m, 2H), 6.88 – 6.81 (m, 2H), 3.78 (d, *J* = 0.7 Hz, 3H), 3.72 (dt, *J* = 12.1, 3.4 Hz, 2H), 2.40 (s, 3H), 2.28 (dt, *J* = 7.2, 2.4 Hz, 2H), 2.09 (td, *J* = 11.9, 2.6 Hz, 2H), 1.66 (dd, *J* = 13.5, 3.4 Hz, 2H), 1.36 – 1.26 (m, 2H), 1.22 – 1.10 (m, 1H).

^{13}C NMR (126 MHz, $CDCl_3$) δ 158.85, 154.02 (dd, *J* = 289.9, 286.4 Hz), 143.51, 133.17, 129.66, 129.34 (t, *J* = 3.2 Hz), 127.82, 125.54 (t, *J* = 3.5 Hz), 114.09, 89.81 (dd, *J* = 21.6, 14.0 Hz), 55.36, 46.38, 34.13, 33.52 (d, *J* = 2.6 Hz), 33.49, 31.16, 21.61.

^{19}F NMR (471 MHz, CDCl3) δ -90.04 (d, *J* = 41.3 Hz), -90.20 (d, *J* = 41.2 Hz).

HRMS (ESI) m/z calculated for $C_{22}H_{26}F_2NO_3S^+$ $[M+H]^+$, 422.1596; found, 422.1600.

4-(1, 1-difluoro-3-(1-tosylpiperidin-4-yl)prop-1-en-2-yl)benzaldehyde (102)

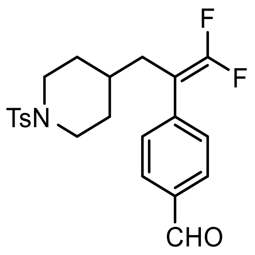

Prepared from **SI-2-1** (0.26 mmol, 1.3 equiv) and **SI-6-4** (0.2 mmol, 1.0 equiv) according to the general procedure 9. Purification by flash column (Petroleum Ether/ Ethyl Acetate = 1/20) afforded **102** (80 mg, 95% yield) as a white solid.

Rf = 0.20 (Petroleum Ether/ Ethyl Acetate = 1/5)

^{1}H NMR (400 MHz, CDCl3) δ 9.98 (s, 1H), 7.84 (dd, *J* = 8.1, 6.3 Hz, 2H), 7.61 – 7.55 (m, 2H), 7.46 – 7.39 (m, 2H), 7.30 – 7.25 (m, 2H), 3.73 (dt, *J* = 11.9, 3.3 Hz, 2H), 2.39 (s, 3H), 2.37 (d, *J* = 2.6 Hz, 2H), 2.09 (td, *J* = 11.9, 2.6 Hz, 2H), 1.72 – 1.63 (m, 2H), 1.34 (qd, *J* = 12.2, 4.0 Hz, 2H), 1.23 – 1.12 (m, 1H).

^{13}C NMR (101 MHz, $CDCl_3$) δ 191.58, 154.49 (dd, *J* = 294.0, 289.2 Hz), 143.57, 139.96 (t, *J* = 4.4 Hz), 135.34, 133.06, 129.98, 129.67, 128.75 (t, *J* = 3.6 Hz), 127.76, 90.24 (dd, *J* = 22.6, 12.4 Hz), 46.28, 33.69, 31.13, 21.59.

^{19}F NMR (471 MHz, CDCl3) δ -91.53 (d, *J* = 44.6 Hz), -91.80 (d, *J* = 44.7 Hz).

HRMS (ESI) m/z calculated for $C_{22}H_{24}F_2NO_3S^+$ $[M+H]^+$, 420.1439; found, 420.1434.

methyl 4-(1,1-difluoro-3-(1-tosylpiperidin-4-yl)prop-1-en-2-yl)benzoate (103)

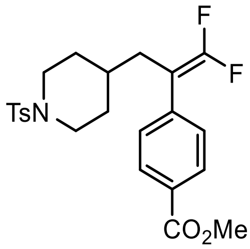

Prepared from **SI-2-1** (0.26 mmol, 1.3 equiv) and **SI-6-5** (0.2 mmol, 1.0 equiv) according to the general procedure 9. Purification by flash column (Petroleum Ether/ Ethyl Acetate = 1/10) afforded **103** (65 mg, 72% yield) as a white solid.

Rf = 0.15 (Petroleum Ether/ Ethyl Acetate = 1/10)

^{1}H NMR (500 MHz, CDCl3) δ 8.04 – 7.94 (m, 2H), 7.64 – 7.54 (m, 2H), 7.34 – 7.26 (m, 4H), 3.90 (s, 3H), 3.77 – 3.68 (m, 2H), 2.40 (s, 3H), 2.35 (dt, *J* = 7.3, 2.3 Hz, 2H), 2.06 (td, *J* = 12.0, 2.6 Hz, 2H), 1.70 – 1.62 (m, 2H), 1.38 – 1.27 (m, 2H), 1.19 - 1.09 (m, 1H).

^{13}C NMR (126 MHz, $CDCl_3$) δ 166.72, 154.39 (dd, *J* = 293.6, 288.5 Hz), 143.56, 138.33 (t, *J* = 4.1 Hz), 133.09, 130.16, 129.92, 129.69, 129.22, 128.18 (t, *J* = 3.2 Hz), 127.81, 90.17 (dd, *J* = 22.1, 12.6 Hz), 52.34, 46.33, 33.75, 33.67, 31.16, 21.63.

^{19}F NMR (376 MHz, $CDCl_3$) δ -88.14 (d, *J* = 37.1 Hz), -88.61 (d, *J* = 37.1 Hz).

HRMS (ESI) m/z calculated for $C_{23}H_{26}F_2NO_4S^+$ $[M+H]^+$, 450.1545; found, 450.1544.

4-(2-(4-butylphenyl)-3, 3-difluoroallyl)-1-tosylpiperidine (104)

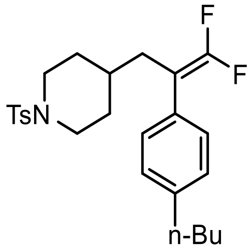

Prepared from **SI-2-1** (0.26 mmol, 1.3 equiv) and **SI-6-2** (0.2 mmol, 1.0 equiv) according to the general procedure 9. Purification by flash column (Petroleum Ether/ Ethyl Acetate = 1/20) afforded **104** (62 mg, 69% yield) as a white solid.

Rf = 0.40 (Petroleum Ether/ Ethyl Acetate = 1/10)

^{1}H NMR (400 MHz, CDCl3) δ 7.59 (d, *J* = 8.0 Hz, 2H), 7.27 (d, *J* = 8.0 Hz, 2H), 7.13 (d, *J* = 1.7 Hz, 4H), 3.72 (dt, *J* = 11.9, 3.4 Hz, 2H), 2.61 – 2.52 (m, 2H), 2.40 (s, 3H),

2.29 (dt, *J* = 7.2, 2.4 Hz, 2H), 2.10 (td, *J* = 11.9, 2.5 Hz, 2H), 1.71 – 1.63 (m, 2H), 1.61 – 1.53 (m, 2H), 1.40 – 1.28 (m, 4H), 1.24 – 1.12 (m, 1H), 0.91 (t, *J* = 7.3 Hz, 3H).

^{13}C NMR (126 MHz, $CDCl_3$) δ154.09 (dd, *J* = 290.8, 286.6 Hz), 143.50, 142.28, 133.17, 130.55 (t, *J* = 3.6 Hz), 129.65, 128.65, 128.00 (t, *J* = 3.2 Hz), 127.81, 90.18 (dd, *J* = 21.4, 13.6 Hz), 46.36, 35.39, 34.04, 33.57, 33.49 (t, *J* = 2.6 Hz), 31.17, 22.52, 21.59, 14.06.

^{19}F NMR (376 MHz, $CDCl_3$) δ -90.84, -90.95, -91.11, -91.23.

HRMS (ESI) m/z calculated for $C_{25}H_{32}F_2NO_2S^+$ $[M+H]^+$, 448.2116; found, 448.2124.

4-(3, 3-difluoro-2-(naphthalen-1-yl)allyl)-1-tosylpiperidine (105)

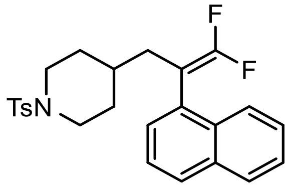

Prepared from **SI-2-1** (0.26 mmol, 1.3 equiv) and **SI-6-10** (0.2 mmol, 1.0 equiv) according to the general procedure 9. Purification by flash column (Petroleum Ether/ Ethyl Acetate = 1/20) afforded **105** (47 mg, 53% yield) as a white solid.

Rf = 0.30 (Petroleum Ether/ Ethyl Acetate = 1/10)

^{1}H NMR (500 MHz, CDCl3) δ 7.88 – 7.84 (m, 1H), 7.81 (dd, *J* = 7.7, 3.2 Hz, 2H), 7.62 – 7.57 (m, 2H), 7.54 – 7.46 (m, 2H), 7.41 (dd, *J* = 8.3, 7.1 Hz, 1H), 7.29 – 7.26 (m, 3H), 3.72 (d, *J* = 11.3 Hz, 2H), 2.40 (bs, 5H), 2.08 (bs, 2H), 1.42 - 1.32 (m, 2H), 1.18 – 1.08 (m, 1H), 0.94 – 0.77 (m, 2H).

^{13}C NMR (126 MHz, $CDCl_3$) δ153.93 (t, *J* = 288.1 Hz), 143.50, 133.96, 133.23, 131.50, 131.21 (d, *J* = 4.7 Hz), 129.68, 128.80, 128.58, 127.79, 127.35 (d, *J* = 3.3 Hz), 126.58, 126.14, 125.34, 124.78, 88.40 (dd, *J* = 22.4, 17.4 Hz), 46.29, 36.05, 33.55, 31.42 (bs), 21.61.

^{19}F NMR (471 MHz, CDCl3) δ -87.21 (d, *J* = 41.5 Hz), -91.86 (d, *J* = 41.4 Hz).

HRMS (ESI) m/z calculated for $C_{25}H_{26}F_2NO_2S^+$ $[M+H]^+$, 442.1647; found, 442.1650.

4-(3,3-difluoro-2-(6-methoxynaphthalen-2-yl)allyl)-1-tosylpiperidine (106)

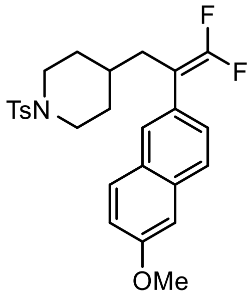

Prepared from **SI-2-1** (0.26 mmol, 1.3 equiv) and **SI-6-11** (0.2 mmol, 1.0 equiv) according to the general procedure 9. Purification by flash column (Petroleum Ether/ Ethyl Acetate = 1/15) afforded **106** (85 mg, 90% yield) as a white solid.

Rf = 0.40 (Petroleum Ether/ Ethyl Acetate = 1/6)

^{1}H NMR (400 MHz, CDCl3) δ 7.67 (dd, *J* = 8.7, 3.4 Hz, 2H), 7.60 (s, 1H), 7.59 – 7.54 (m, 2H), 7.31 (dt, *J* = 8.7, 1.7 Hz, 1H), 7.26 (d, *J* = 1.5 Hz, 2H), 7.14 (dd, *J* = 8.9, 2.6 Hz, 1H), 7.09 (d, *J* = 2.5 Hz, 1H), 3.91 (s, 3H), 3.71 (d, *J* = 11.6 Hz, 2H), 2.40 (dt, *J* = 7.1, 2.3 Hz, 2H), 2.37 (s, 3H), 2.04 (td, *J* = 11.9, 2.6 Hz, 2H), 1.70 (d, *J* = 12.6 Hz, 2H), 1.41 – 1.27 (m, 2H), 1.24 – 1.12 (m, 1H).

^{13}C NMR (126 MHz, $CDCl_3$) δ 158.13, 154.29 (dd, *J* = 290.7, 287.1 Hz), 143.49, 133.76, 133.05, 129.65, 129.48, 128.79, 128.50 (t, *J* = 3.7 Hz), 127.80, 127.15, 127.13, 126.53 (t, *J* = 3.1 Hz), 119.41, 105.66, 90.44 (dd, *J* = 21.8, 13.6 Hz), 55.50, 46.40, 34.12, 33.56 (t, *J* = 2.5 Hz), 31.19, 21.60.

^{19}F NMR (471 MHz, CDCl3) δ -90.55 (d, *J* = 42.6 Hz), -91.04 (d, *J* = 42.5 Hz).

HRMS (ESI) m/z calculated for $C_{26}H_{28}F_2NO_3S^+$ $[M+H]^+$, 472.1752; found, 472.1756.

4-(2-(dibenzo[b,d]thiophen-4-yl)-3,3-difluoroallyl)-1-tosylpiperidine (107)

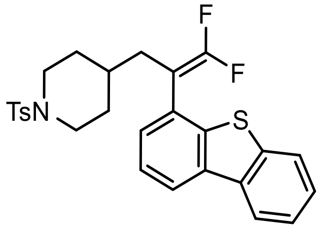

Prepared from **SI-2-1** (0.26 mmol, 1.3 equiv) and **SI-6-14** (0.2 mmol, 1.0 equiv) according to the general procedure 9. Purification by flash column (Petroleum Ether/ Ethyl Acetate = 1/20) afforded **107** (82 mg, 82% yield) as a white solid.

Rf = 0.20 (Petroleum Ether/ Ethyl Acetate = 1/10)

^{1}H NMR (400 MHz, CDCl3) δ 8.15 – 8.10 (m, 1H), 8.08 (dd, *J* = 7.9, 1.1 Hz, 1H), 7.86 – 7.81 (m, 1H), 7.58 – 7.54 (m, 2H), 7.49 – 7.41 (m, 3H), 7.27 – 7.21 (m, 3H), 3.71 (dt, *J* = 12.2, 3.5 Hz, 2H), 2.44 (dt, *J* = 7.2, 2.3 Hz, 2H), 2.35 (s, 3H), 2.05 (td, *J* = 12.0, 2.5 Hz, 2H), 1.77 (dd, *J* = 13.8, 3.5 Hz, 2H), 1.44 – 1.29 (qd, *J* = 12.3, 4.1 Hz, 2H), 1.15 – 1.105 (m, 1H).

^{13}C NMR (126 MHz, $CDCl_3$) δ153.70 (dd, *J* = 291.1, 290.1 Hz), 143.44, 139.71, 139.08, 136.18, 135.71, 133.15, 129.64, 128.63 (dd, *J* = 5.1, 1.6 Hz), 127.74, 127.37 (t, *J* = 1.8 Hz), 127.19, 124.87, 124.69, 122.83, 121.87, 121.20, 89.60 (dd, *J* = 23.5, 15.9 Hz), 46.29, 34.76, 33.65 (t, *J* = 2.6 Hz), 31.39, 21.56.

^{19}F NMR (471 MHz, CDCl3) δ -85.37 (d, *J* = 36.6 Hz), -90.41 (d, *J* = 36.6 Hz).

HRMS (ESI) m/z calculated for $C_{27}H_{26}F_2NO_2S_2^+$ $[M+H]^+$, 498.1368; found, 498.1367.

2-(1,1-difluoro-3-(1-tosylpiperidin-4-yl)prop-1-en-2-yl)aniline (108)

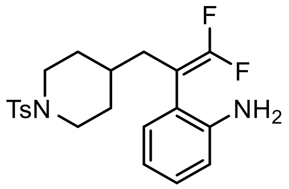

Prepared from **SI-2-1** (0.26 mmol, 1.3 equiv) and **SI-6-8** (0.2 mmol, 1.0 equiv) according to the general procedure 9. Purification by flash column (Petroleum Ether/ Ethyl Acetate = 1/20) afforded **108** (73 mg, 90% yield) as a white solid.

Rf = 0.30 (Petroleum Ether/ Ethyl Acetate = 1/5)

^{1}H NMR (400 MHz, $CDCl_3$) δ 7.60 (d, *J* = 7.9 Hz, 2H), 7.31 – 7.25 (m, 2H), 7.09 (td, *J* = 7.7, 1.5 Hz, 1H), 6.90 (dd, *J* = 7.5, 1.5 Hz, 1H), 6.73 – 6.66 (m, 2H), 3.73 (dd, *J* = 11.7, 3.4 Hz, 2H), 3.16 (brs, 2H), 2.41 (s, 3H), 2.23 (dt, *J* = 7.1, 2.1 Hz, 2H), 2.13 (td, *J* = 11.9, 2.5 Hz, 2H), 1.76 – 1.66 (m, 2H), 1.32 (qt, *J* = 14.1, 7.0 Hz, 2H), 1.23 – 1.10 (m, 1H).

^{13}C NMR (101 MHz, $CDCl_3$) δ153.53 (dd, *J* = 290.3, 288.1 Hz), 144.32, 143.52, 133.15, 130.30 (t, *J* = 1.71 Hz), 129.68, 129.17, 127.79, 118.56, 118.51, 115.98, 87.18 (dd, *J* = 21.5, 17.2 Hz), 46.34, 34.16, 33.44 (t, *J* = 2.5 Hz), 31.38, 21.61.

^{19}F NMR (376 MHz, $CDCl_3$) δ -87.52 (d, *J* = 40.8 Hz), -91.57 (d, *J* = 40.8 Hz).

HRMS (ESI) m/z calculated for $C_{21}H_{25}F_2N_2O_2S^+$ $[M+H]^+$, 407.1599; found, 407.1603.

4-(2-(1,9-dihydropyren-2-yl)-3,3-difluoroallyl)-1-tosylpiperidine (109)

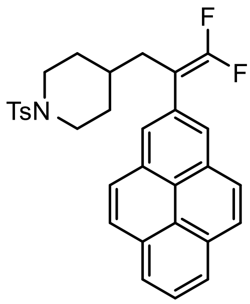

Prepared from **SI-2-1** (0.26 mmol, 1.3 equiv) and **SI-6-12** (0.2 mmol, 1.0 equiv) according to the general procedure 9. Purification by flash column (Petroleum Ether/ Ethyl Acetate = 1/20) afforded **109** (58 mg, 56% yield) as a white solid.

Rf = 0.30 (Petroleum Ether/ Ethyl Acetate = 1/10)

^{1}H NMR (400 MHz, $CDCl_3$) δ 8.20 (d, *J* = 7.6 Hz, 2H), 8.14 – 8.00 (m, 6H), 7.78 (d, *J* = 7.9 Hz, 1H), 7.57 – 7.53 (m, 2H), 7.22 (d, *J* = 8.0 Hz, 2H), 3.71 (d, *J* = 11.5 Hz, 2H), 2.54 (brs, 2H), 2.35 (s, 3H), 2.03 (brs, 2H), 1.76 (d, *J* = 13.2 Hz, 2H), 1.41 (qd, *J* = 12.2, 4.2 Hz, 2H), 1.19-1.06 (m, 1H).

^{13}C NMR (126 MHz, $CDCl_3$) δ 153.90 (dd, *J* = 288.5, 288.5 Hz), 143.45, 133.11, 131.36, 131.14, 130.87, 129.64, 129.15, 128.45 (d, *J* = 4.6 Hz), 128.24, 127.96, 127.74, 127.31, 127.16 (d, *J* = 3.3 Hz), 126.35, 125.65, 125.46, 125.10, 124.79, 124.09, 88.90 (dd, *J* = 21.9, 17.2 Hz), 46.28, 36.63, 33.63, 31.52, 21.57.

^{19}F NMR (376 MHz, $CDCl_3$) δ -86.95 (d, *J* = 40.4 Hz), -91.42 (d, *J* = 40.4 Hz).

HRMS (ESI) m/z calculated for $C_{31}H_{28}F_2NO_2S^+$ $[M+H]^+$, 516.1803; found, 516.1804.

4-(2-(benzofuran-2-yl)-3,3-difluoroallyl)-1-tosylpiperidine (110)

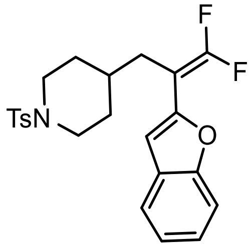

Prepared from **SI-2-1** (0.26 mmol, 1.3 equiv) and **SI-6-13** (0.2 mmol, 1.0 equiv) according to the general procedure 9. Purification by flash column (Petroleum Ether/

Ethyl Acetate = 1/20) afforded **110** (36 mg, 42% yield) as a white solid.

Rf = 0.35 (Petroleum Ether/ Ethyl Acetate = 1/10)

^{1}H NMR (500 MHz, CDCl3) δ 7.60 (dd, *J* = 8.4, 2.0 Hz, 2H), 7.51 (dd, *J* = 7.4, 1.5 Hz, 1H), 7.43 – 7.39 (m, 1H), 7.28 (d, *J* = 8.0 Hz, 2H), 7.22 (dtd, *J* = 14.7, 7.6, 1.3 Hz, 2H), 6.65 (s, 1H), 3.76 (dt, *J* = 11.3, 2.5 Hz, 2H), 2.43 - 2.38 (m, 5H), 2.15 (td, *J* = 11.6, 2.4 Hz, 2H), 1.76 (dt, *J* = 13.4, 2.7 Hz, 2H), 1.48 - 1.35 (m, 3H).

^{13}C NMR (126 MHz, $CDCl_3$) δ 155.20 (dd, *J* = 288.1, 288.4 Hz), 154.32, 149.64, 143.55, 133.09, 129.69, 128.68, 127.83, 124.32, 123.19, 120.83, 111.10, 104.72 (dd, *J* = 8.9, 4.9 Hz), 84.51 (dd, *J* = 28.5, 11.3 Hz), 46.45, 34.68 (d, *J* = 2.7 Hz), 31.19, 21.63.

^{19}F NMR (471 MHz, CDCl3) δ -80.06 (dd, *J* = 24.3, 1.6 Hz), -86.14 (dd, *J* = 24.4, 2.8 Hz).

HRMS (ESI) m/z calculated for $C_{23}H_{23}F_2NO_3SNa^+$ $[M+Na]^+$, 454.1259; found, 454.1262.

***tert*-butyl 4-(2-([1,1'-biphenyl]-4-yl)-3,3-difluoroallyl)piperidine-1-carboxylate (111)**

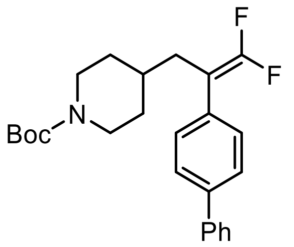

Prepared from **SI-2-2** (0.26 mmol, 1.3 equiv) and **SI-6-1** (0.2 mmol, 1.0 equiv) according to the general procedure 9. Purification by flash column (Petroleum Ether/ Ethyl Acetate = 1/20) afforded **111** (63 mg, 76% yield) as a white solid.

Rf = 0.35 (Petroleum Ether/ Ethyl Acetate = 1/10)

^{1}H NMR (500 MHz, $CDCl_3$) δ 7.60 (p, *J* = 3.4 Hz, 4H), 7.45 (td, *J* = 8.1, 7.5, 2.4 Hz, 2H), 7.38 (t, *J* = 9.5 Hz, 3H), 4.06 (brs, 2H), 2.60 (t, *J* = 12.9 Hz, 2H), 2.45 – 2.34 (m, 2H), 1.65 (d, *J* = 13.3 Hz, 3H), 1.45 (s, 9H), 1.15 (q, *J* = 12.6 Hz, 2H).

^{13}C NMR (126 MHz, $CDCl_3$) δ 154.90, 154.27 (dd, *J* = 291.4, 287.0 Hz), 140.56, 140.25, 132.68 (t, *J* = 3.9 Hz), 128.95, 128.66 (t, *J* = 3.3 Hz), 127.58, 127.32, 127.11, 90.36 (dd, *J* = 22.0, 12.9 Hz), 79.41, 43.71 (m), 34.42 (d, *J* = 2.5 Hz), 31.87, 28.59.

^{19}F NMR (376 MHz, $CDCl_3$) δ -90.07 (d, *J* = 42.0 Hz), -90.53 (d, *J* = 42.0 Hz).

HRMS (ESI) m/z calculated for $C_{25}H_{29}F_2NO_2Na^+$ $[M+Na]^+$, 436.2059; found, 436.2060.

4-(2-([1, 1'-biphenyl]-4-yl)-3,3-difluoroallyl)tetrahydro-2*H*-pyran (112)

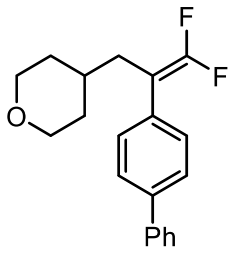

Prepared from **SI-2-3** (0.26 mmol, 1.3 equiv) and **SI-6-1** (0.2 mmol, 1.0 equiv) according to the general procedure 9. Purification by flash column (Petroleum Ether/ Ethyl Acetate = 1/50) afforded **112** (55 mg, 88% yield) as a white solid.

Rf = 0.50 (Petroleum Ether/ Ethyl Acetate = 1/10)

^1H NMR (400 MHz, $CDCl_3$) δ 7.68 – 7.56 (m, 4H), 7.45 (dd, *J* = 8.4, 6.9 Hz, 2H), 7.41 – 7.33 (m, 3H), 3.98 – 3.88 (m, 2H), 3.28 (td, *J* = 11.8, 1.9 Hz, 2H), 2.39 (dt, *J* = 6.9, 2.5 Hz, 2H), 1.60 – 1.53 (m, 3H), 1.40 - 1.25 (m, 2H).

^{13}C NMR (126 MHz, $CDCl_3$) δ154.31 (dd, *J* = 291.1, 287.0 Hz), 140.59, 140.25, 132.74 (dd, *J* = 3.34, 3.59 Hz), 128.96, 128.67 (t, *J* = 3.3 Hz), 127.59, 127.32, 127.13, 90.22 (dd, *J* = 22.0, 12.9 Hz), 67.93, 34.77, 33.41 (t, *J* = 2.5 Hz), 32.79.

^{19}F NMR (471 MHz, $CDCl_3$) δ -90.16 (d, *J* = 42.2 Hz), -90.57 (d, *J* = 42.0 Hz).

HRMS (APCI) m/z calculated for $C_{20}H_{21}F_2O^+$ $[M+H]^+$, 315.1555; found, 315.1555.

2-(2-([1,1'-biphenyl]-4-yl)-3,3-difluoroallyl)-2,3-dihydro-1*H*-indene (113)

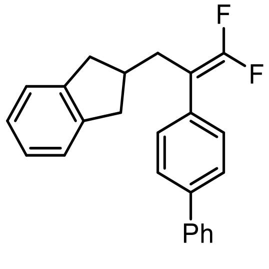

Prepared from **SI-2-6** (0.26 mmol, 1.3 equiv) and **SI-1-6** (0.2 mmol, 1.0 equiv) according to the general procedure 9. Purification by flash column (Petroleum Ether) afforded **113** (42 mg, 61% yield) as a white solid.

Rf = 0.50 (Petroleum Ether)

^{1}H NMR (400 MHz, $CDCl_3$) δ 7.64 – 7.57 (m, 4H), 7.44 (q, *J* = 7.7 Hz, 4H), 7.36 (t, *J* = 7.4 Hz, 1H), 7.20 – 7.09 (m, 4H), 2.99 (dd, *J* = 15.4, 7.6 Hz, 2H), 2.70 – 2.59 (m, 4H), 2.58 – 2.46 (m, 1H).

^{13}C NMR (126 MHz, $CDCl_3$) δ 154.26 (dd, *J* = 287.88, 290.87), 143.04, 140.69, 140.31, 132.72, 128.95, 128.85 (t, *J* = 3.1 Hz), 127.55, 127.31, 127.17, 126.32, 124.58, 91.68 (dd, *J* = 21.6, 13.2 Hz), 38.79, 38.28 (t, *J* = 2.4 Hz)., 33.27.

^{19}F NMR (471 MHz, $CDCl_3$) δ -90.86 (d, *J* = 43.0 Hz), -91.12 (d, *J* = 42.9 Hz).

HRMS (APCI) m/z calculated for $C_{24}H_{21}F_2^+$ $[M+H]^+$, 347.1606; found, 347.1593.

4-(3-cyclohexyl-1,1-difluoroprop-1-en-2-yl)-1,1'-biphenyl (114)

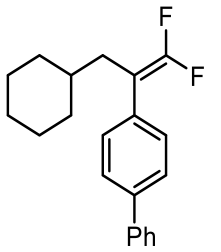

Prepared from **SI-2-4** (0.26 mmol, 1.3 equiv) and **SI-6-1** (0.2 mmol, 1.0 equiv) according to the general procedure 9. Purification by flash column (Petroleum Ether) afforded **114** (45 mg, 72% yield) as a white solid.

Rf = 0.80 (Petroleum Ether)

^{1}H NMR (400 MHz, CDCl3) δ 7.64 – 7.57 (m, 4H), 7.45 (dd, *J* = 8.4, 6.8 Hz, 2H), 7.42 – 7.33 (m, 3H), 2.32 (dt, *J* = 7.4, 2.5 Hz, 2H), 1.76 – 1.60 (m, 5H), 1.37 – 1.27 (m, 1H), 1.20 – 1.10 (m, 3H), 1.02 - 0.90 (m, 2H).

^{13}C NMR (101 MHz, $CDCl_3$) δ154.23 (dd, *J* = 290.7, 286.5 Hz), 140.75, 140.01, 133.24 (dd, *J* = 3.9 Hz), 128.93, 128.76 (t, *J* = 3.3 Hz), 127.49, 127.19, 127.14, 90.95 (dd, *J* = 22.1, 12.3 Hz), 35.91 (dd, *J* = 2.8, 2.8 Hz)., 35.28, 33.05, 26.57, 26.22.

^{19}F NMR (376 MHz, CDCl3) δ -90.70 (d, *J* = 43.4 Hz), -91.24 (d, *J* = 43.4 Hz).

methyl 4-([1,1'-biphenyl]-4-yl)-2-benzyl-5,5-difluoropent-4-enoate (115)

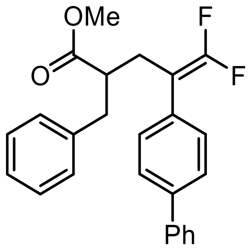

Prepared from **SI-2-8** (0.26 mmol, 1.3 equiv) and **SI-6-1** (0.2 mmol, 1.0 equiv) according to the general procedure 9. Purification by flash column (Petroleum Ether) afforded **115** (31 mg, 40% yield) as a white solid.

Rf = 0.50 (Petroleum Ether/ Ethyl Acetate = 1/10)

^{1}H NMR (400 MHz, $CDCl_3$) δ 7.62 – 7.54 (m, 4H), 7.47 – 7.42 (m, 2H), 7.38 – 7.32 (m, 1H), 7.28 – 7.18 (m, 5H), 7.09 – 7.04 (m, 2H), 3.50 (s, 3H), 2.96 (dd, *J* = 13.2, 7.8 Hz, 1H), 2.86 – 2.69 (m, 3H), 2.63 (ddt, *J* = 13.7, 5.3, 2.4 Hz, 1H).

^{13}C NMR (101 MHz, $CDCl_3$) δ 174.95, 154.23 (dd, *J* = 291.0, 289.1 Hz), 140.61, 140.49, 138.78, 131.72 (t, *J* = 3.2 Hz), 128.96, 128.85 (t, *J* = 3.2 Hz), 128.57, 127.61, 127.52, 127.31, 127.16, 126.65, 90.22 (dd, *J* = 20.9, 15.1 Hz), 51.68, 45.89 (t, *J* = 2.2 Hz), 38.00, 30.20.

^{19}F NMR (376 MHz, $CDCl_3$) δ -89.45 (d, *J* = 38.8 Hz), -89.68 (d, *J* = 38.8 Hz).

HRMS (ESI) m/z calculated for $C_{25}H_{22}F_2O_2Na^+$ $[M+Na]^+$, 415.1480; found, 415.1474.

2-(1,1-difluoro-4-methyl-6-phenylhex-1-en-2-yl)-6-methoxynaphthalene (116)

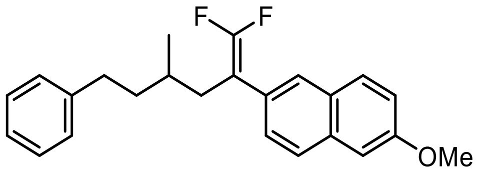

Prepared from **SI-2-7** (0.26 mmol, 1.3 equiv) and **SI-6-11** (0.2 mmol, 1.0 equiv) according to the general procedure 9. Purification by flash column (Petroleum Ether/ Ethyl Acetate = 1/400) afforded **116** (50 mg, 68% yield) as a white solid.

Rf = 0.40 (Petroleum Ether)

^{1}H NMR (400 MHz, $CDCl_3$) δ 7.71 (dd, *J* = 8.8, 2.0 Hz, 2H), 7.65 (d, *J* = 1.6 Hz, 1H), 7.36 (dt, *J* = 8.6, 1.6 Hz, 1H), 7.24 – 7.07 (m, 7H), 3.93 (s, 3H), 2.68 – 2.58 (m, 1H),

2.57 – 2.46 (m, 2H), 2.38 – 2.30 (m, 1H), 1.74 – 1.64 (m, 1H), 1.55 – 1.42 (m, 2H), 0.94 (d, *J* = 6.4 Hz, 3H).

^{13}C NMR (126 MHz, $CDCl_3$) δ 157.99, 154.27 (dd, *J* = 289.7, 286.2 Hz), 142.72, 133.71, 129.56, 129.05 (dd, *J* = 3.3, 2.4 Hz), 128.87, 128.40, 128.39, 127.32 (t, *J* = 3.2 Hz), 126.98, 126.85 (t, *J* = 3.1 Hz), 125.75, 119.22, 105.67, 91.56 (dd, *J* = 21.8, 13.2 Hz), 55.49, 38.37, 35.02, 33.35, 30.97 (t, *J* = 2.3 Hz), 19.35.

^{19}F NMR (376 MHz, CDCl3) δ -91.48 (d, *J* = 44.5 Hz), -91.83 (d, *J* = 44.7 Hz).

HRMS (APCI) m/z calculated for $C_{24}H_{25}F_2O^+$ $[M+H]^+$, 367.1868; found, 367.1875.

4-(1,1-difluoro-4-phenylbut-1-en-2-yl)-1,1'-biphenyl (117)

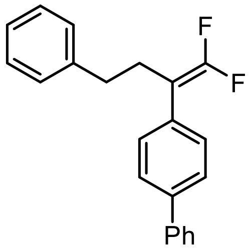

Prepared from **SI-2-10** (0.26 mmol, 1.3 equiv) and **SI-6-1** (0.2 mmol, 1.0 equiv) according to the general procedure 9. Purification by flash column (Petroleum Ether) afforded **117** (20 mg, 31% yield) as a white solid.

Rf = 0.80 (Petroleum Ether)

^{1}H NMR (400 MHz, CDCl3) δ 7.65 – 7.59 (m, 4H), 7.48 – 7.35 (m, 5H), 7.29 (dd, *J* = 8.1, 6.7 Hz, 2H), 7.24 – 7.15 (m, 3H), 2.78 - 2.69 (m, 4H).

^{13}C NMR (126 MHz, $CDCl_3$) δ153.98 (dd, *J* = 291.3, 287.2 Hz), 141.15, 140.69, 140.25, 132.54 (d, *J* = 4.3 Hz), 128.97, 128.76 (t, *J* = 3.4 Hz), 128.56, 128.53, 127.57, 127.33, 127.17, 126.26, 91.70 (dd), 34.24 (t, *J* = 2.6 Hz), 29.72.

^{19}F NMR (376 MHz, CDCl3) δ -90.48 (d, *J* = 41.7 Hz), -90.92 (d, *J* = 41.7 Hz).

HRMS (APCI) m/z calculated for $C_{22}H_{18}F_2^+$ $[M]^+$, 320.1371; found, 320.1376.

4-(1,1-difluorohept-1-en-2-yl)-1,1'-biphenyl (118)

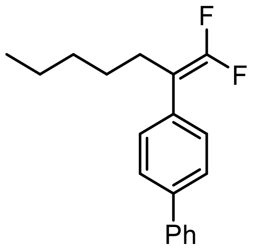

Prepared from **SI-2-9** (0.26 mmol, 1.3 equiv) and **SI-6-1** (0.2 mmol, 1.0 equiv) according to the general procedure 9. Purification by flash column (Petroleum Ether) afforded **118** (14 mg, 25% yield) as a white solid.

Rf = 0.90 (Petroleum Ether)

^{1}H NMR (400 MHz, CDCl3) δ 7.60 (t, *J* = 7.4 Hz, 4H), 7.40 (tt, *J* = 19.5, 7.4 Hz, 5H), 2.43 (tt, *J* = 8.0, 2.5 Hz, 2H), 1.41 (q, *J* = 7.1, 6.6 Hz, 2H), 1.30 (dp, *J* = 10.3, 6.2, 5.0 Hz, 4H), 0.92 – 0.82 (m, 3H).

^{13}C NMR (101 MHz, $CDCl_3$) δ 153.83 (dd, *J* = 287.63, 288.35 Hz), 140.78, 140.08, 133.01, 128.94, 128.72 (t, *J* = 3.3 Hz), 127.50, 127.21, 127.16, 92.35 (dd, *J* = 19.7, 14.7 Hz), 31.40, 27.67 (dd, *J* = 2.5, 2.5 Hz), 27.64, 22.50, 14.15.

^{19}F NMR (376 MHz, $CDCl_3$) δ -91.37 (td, *J* = 44.1, 2.7 Hz), -90.51 (d, *J* = 43.61 Hz).

HRMS (APCI) m/z calculated for $C_{19}H_{20}F_2^+$ $[M]^+$, 286.1528; found, 286.1532.

4-(2-phenylallyl)-1-tosylpiperidine (120)

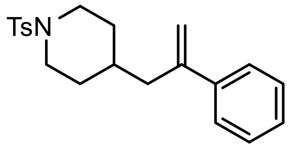

Reactions were set up in a N_2 filled glove box. To a 10 mL reaction tube equipped with a stirring bar, were added NHPI ester **1** (0.2 mmol, 1 equiv), ((2-phenylallyl)sulfonyl)benzene **119** (0.6 mmol, 3 equiv), Mn (1 mmol, 5 equiv), 2,2':6',2''-terpyridine (0.1 mmol, 0.5 equiv), DMA (0.5 mL, 0.4 M) under N_2 atmosphere. After that, the resulting mixture was sealed with a screw cap and the resulting mixture was stirred at 50 ^{o}C for 36 h. Then saturated aqueous NH_4Cl was added to quench the reaction. The aqueous layer was then extracted with diethyl ether, and the combined organic layers were washed with brine, dried over anhydrous Na_2SO_4, filtered and concentrated. The residue was purified by flash column

chromatography on silica gel (Petroleum ether/Ethyl acetate = 30/1) to afforded **120** (30 mg, 42% yield) as a white solid.

Rf = 0.4 (Petroleum ether/Ethyl acetate = 50/1).

^{1}H NMR (400 MHz, CDCl3) δ 7.58 (d, *J* = 8.2 Hz, 2H), 7.38 – 7.24 (m, 7H), 5.26 (d, *J* = 1.8 Hz, 1H), 5.05 – 4.98 (m, 1H), 3.71 (dt, *J* = 12.3, 3.9 Hz, 2H), 2.46 – 2.38 (m, 5H), 2.08 (td, *J* = 11.7, 2.6 Hz, 2H), 1.73 – 1.65 (m, 2H), 1.36 – 1.23 (m, 3H).

^{13}C NMR (101 MHz, $CDCl_3$) δ 146.1, 143.5, 140.8, 133.1, 129.6, 128.5, 127.8, 127.6, 126.2, 114.5, 46.5, 42.4, 33.4, 31.5, 21.6.

***tert*-butyl (*Z*)-4-(2-(4-cyanophenyl)-1-fluorovinyl)piperidine-1-carboxylate (123)**

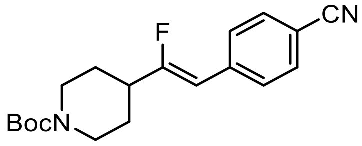

Reactions were set up in a N_2 filled glove box. To a 10 mL reaction tube equipped with a stirring bar, were added **SI-2-2** (0.2 mmol, 1 equiv), **122** (0.3 mmol, 1.5 equiv), Mn (1 mmol, 5 equiv), DMF (0.5 mL, 0.4 M) under N_2 atmosphere. After that, the resulting mixture was sealed with a screw cap and the resulting mixture was stirred at 50 ^{o}C for 20 h. Then saturated aqueous NH_4Cl was added to quench the reaction. The aqueous layer was then extracted with diethyl ether, and the combined organic layers were washed with brine, dried over anhydrous Na_2SO_4, filtered and concentrated. The residue was purified by flash column chromatography on silica gel (Petroleum ether/Ethyl acetate = 10/1) to afforded **123** (20 mg, 30% yield) as a white solid.

Rf = 0.2 (Petroleum ether/Ethyl acetate = 10/1).

^{1}H NMR (500 MHz, CDCl3) δ 7.64 – 7.50 (m, 4H), 5.50 (dd, *J* = 39.0, 2.9 Hz, 1H), 4.21 (brs, 2H), 2.76 (brs, 2H), 2.43 (q, *J* = 13.1 Hz, 1H), 1.90 (d, *J* = 13.1 Hz, 2H), 1.59 – 1.40 (m, 11H).

^{13}C NMR (126 MHz, $CDCl_3$) δ165.82 (d, *J* = 272.6 Hz), 154.80, 138.29 (d, *J* = 2.3 Hz), 132.35, 129.00 (d, *J* = 8.1 Hz), 119.11, 110.25 (d, *J* = 2.9 Hz), 103.80 (d, *J* = 8.0 Hz), 79.91, 43.56, 40.09 (d, *J* = 25.1 Hz), 29.85, 29.00, 28.58.

^{19}F NMR (376 MHz, $CDCl_3$) δ -100.64.

cyclohexyldiphenylphosphane (126)

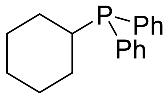

Reactions were set up in a N_2 filled glove box. To a 10 mL reaction tube equipped with a stirring bar, were added NHPI ester **1** (0.15 mmol, 1.5 equiv), diphenyl-phosphinouschlorid (0.1 mmol, 1 equiv), Mn (0.3 mmol, 3 equiv) DMA (0.2 mL, 0.5 M) under N_2 atmosphere. After that, the resulting mixture was sealed with a screw cap and the resulting mixture was stirred at 100 ^oC for 15 h. Then saturated aqueous NH_4Cl was added to quench the reaction. The aqueous layer was then extracted with diethyl ether, and the combined organic layers were washed with brine, dried over anhydrous Na_2SO_4, filtered and concentrated. The residue was purified by flash column chromatography on silica gel (Petroleum ether/Ethyl acetate = 10/1) to afforded **126** (10 mg, 36% yield) as a white solid.

Reactions were set up in a N_2 filled glove box. To a 10 mL reaction tube equipped with a stirring bar, were added pyridinium salt **SI-2-4** (0.15 mmol, 1.5 equiv), diphenyl-phosphinouschlorid (0.1 mmol, 1 equiv), Mn (0.5 mmol, 5 equiv), DMSO (0.2 mL, 0.5 M) under N_2 atmosphere. After that, the resulting mixture was sealed with a screw cap and the resulting mixture was stirred at 70 ^oC for 12 h. Then saturated aqueous NH_4Cl was added to quench the reaction. The aqueous layer was then extracted with diethyl ether, and the combined organic layers were washed with brine, dried over anhydrous Na_2SO_4, filtered and concentrated. The residue was purified by flash column chromatography on silica gel (Petroleum ether/Ethyl acetate = 10/1) to afforded **126** (2 mg, 7% yield) as a white solid.

^{1}H NMR (500 MHz, $CDCl_3$) δ 7.50 (td, *J* = 7.4, 2.0 Hz, 4H), 7.37 – 7.29 (m, 6H), 2.26 – 2.17 (m, 1H), 1.76 (d, *J* = 11.7 Hz, 2H), 1.70 (d, *J* = 9.3 Hz, 3H), 1.36 – 1.19 (m, 5H).

^{13}C NMR (126 MHz, $CDCl_3$) δ 137.3 (d, *J* = 13.8 Hz), 133.8 (d, *J* = 19.1 Hz), 128.7, 128.4 (d, *J* = 6.9 Hz), 35.6 (d, *J* = 8.7 Hz), 29.7 (d, *J* = 15.3 Hz), 26.9 (d, *J* = 11.3 Hz), 26.50.

^{31}P NMR (202 MHz, $CDCl_3$) δ -3.61.

NMR Spectra

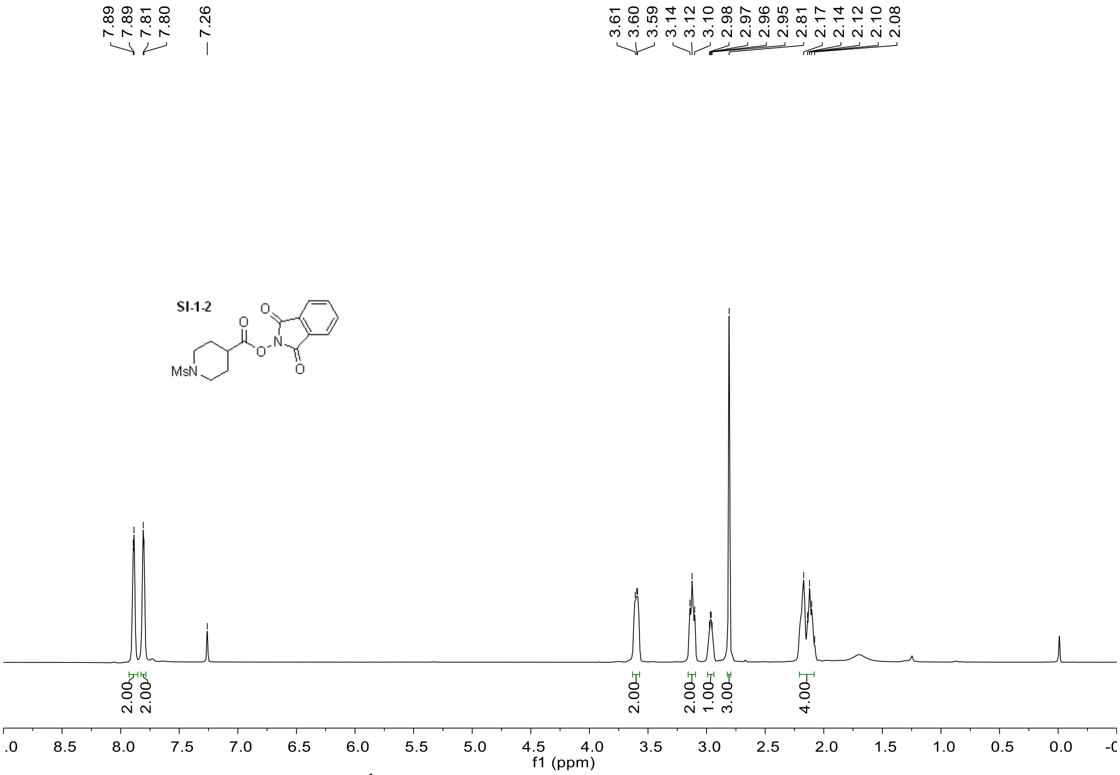

Supplementary Figure 5. ^{1}H NMR Spectra of compound **SI-1-2**.

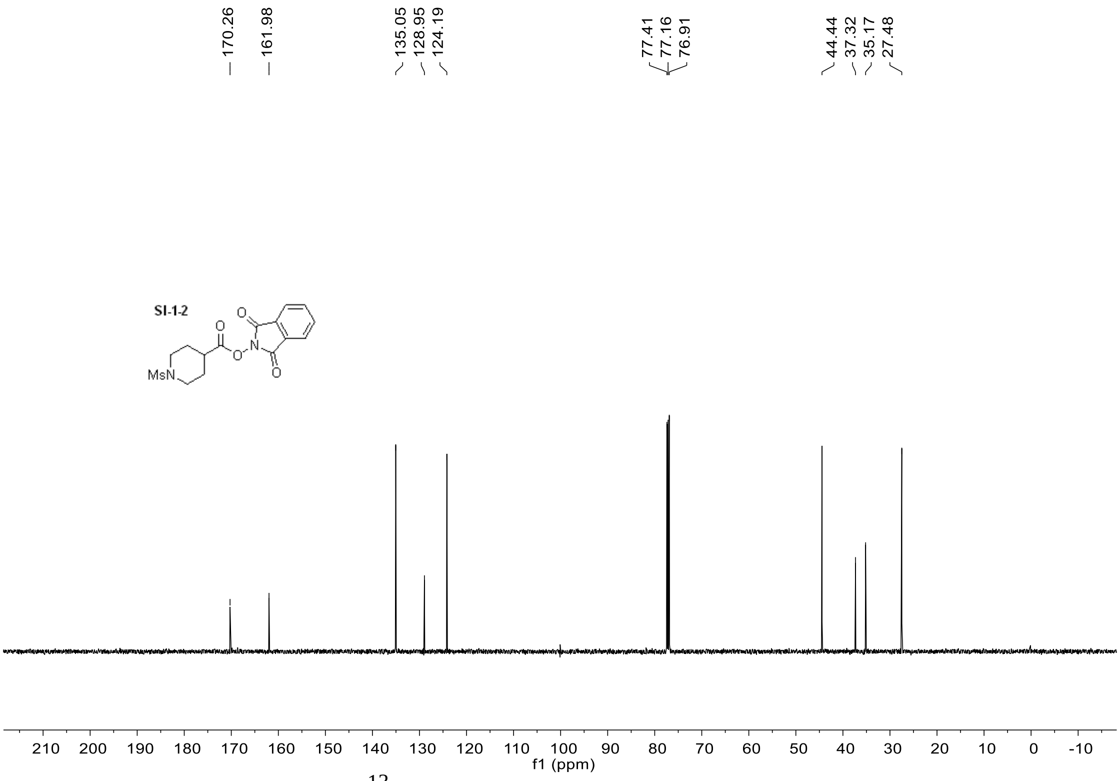

Supplementary Figure 6. ^{13}C NMR Spectra of compound **SI-1-2**.

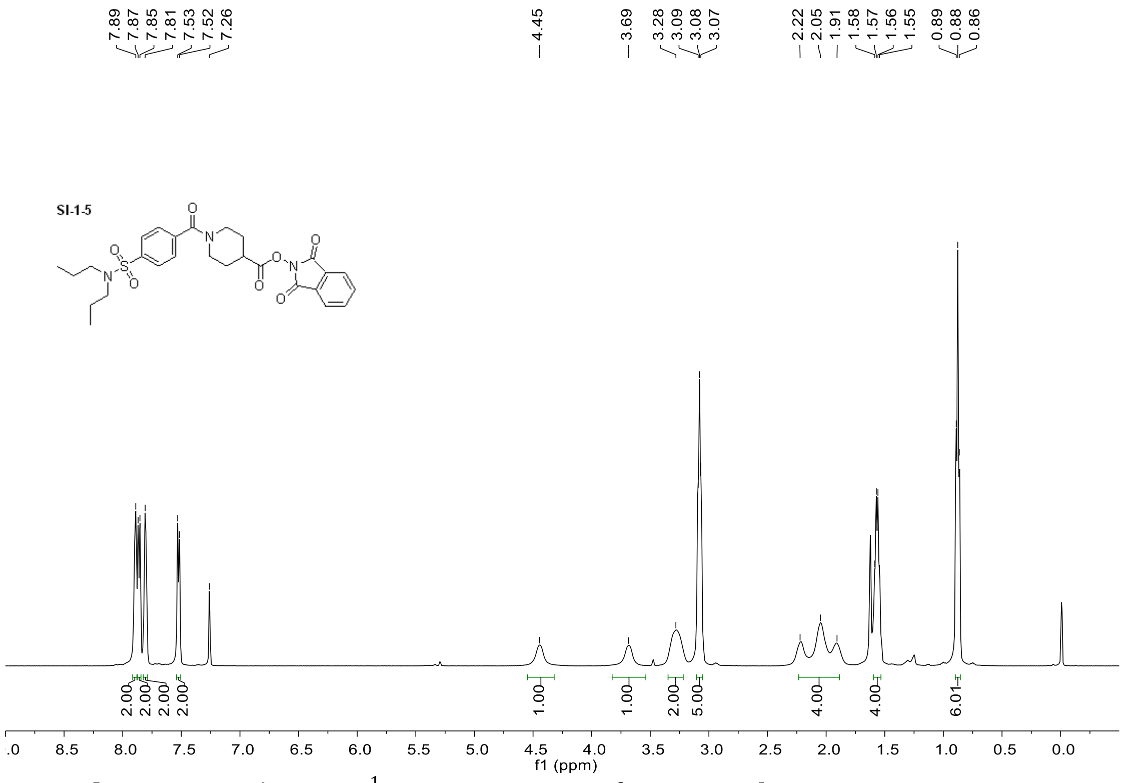

Supplementary Figure 7. ^{1}H NMR Spectra of compound **SI-1-5**.

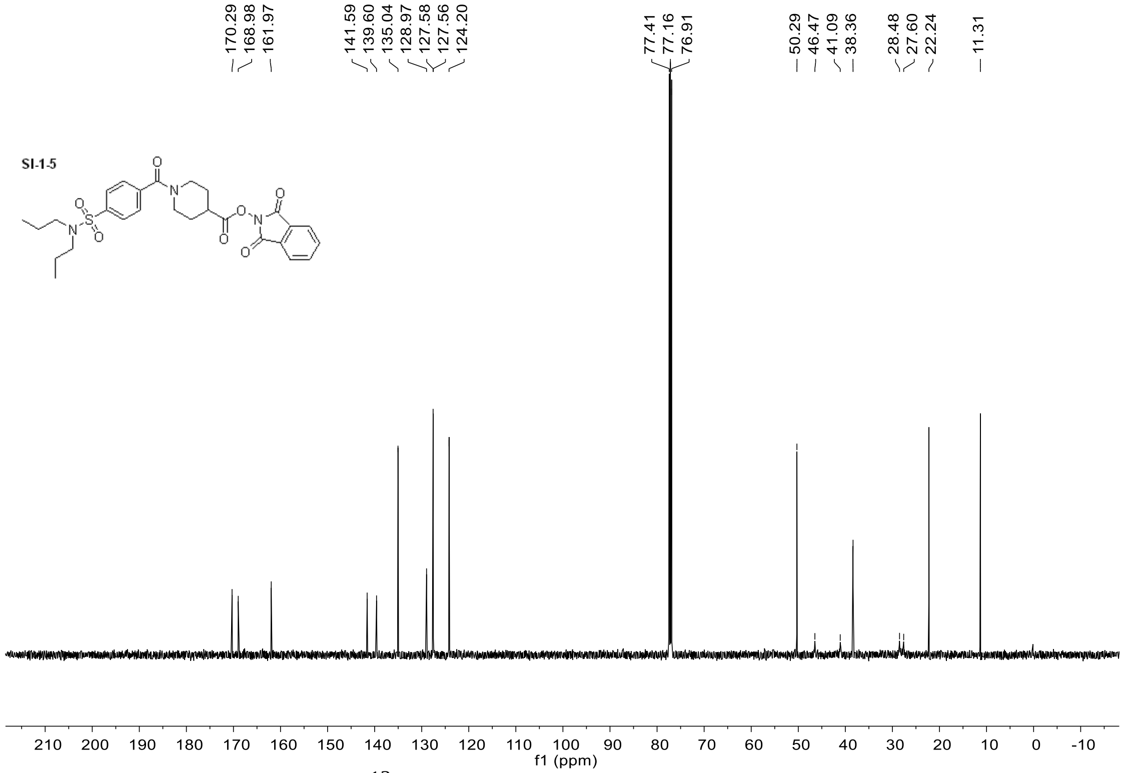

Supplementary Figure 8. ^{13}C NMR Spectra of compound **SI-1-5**.

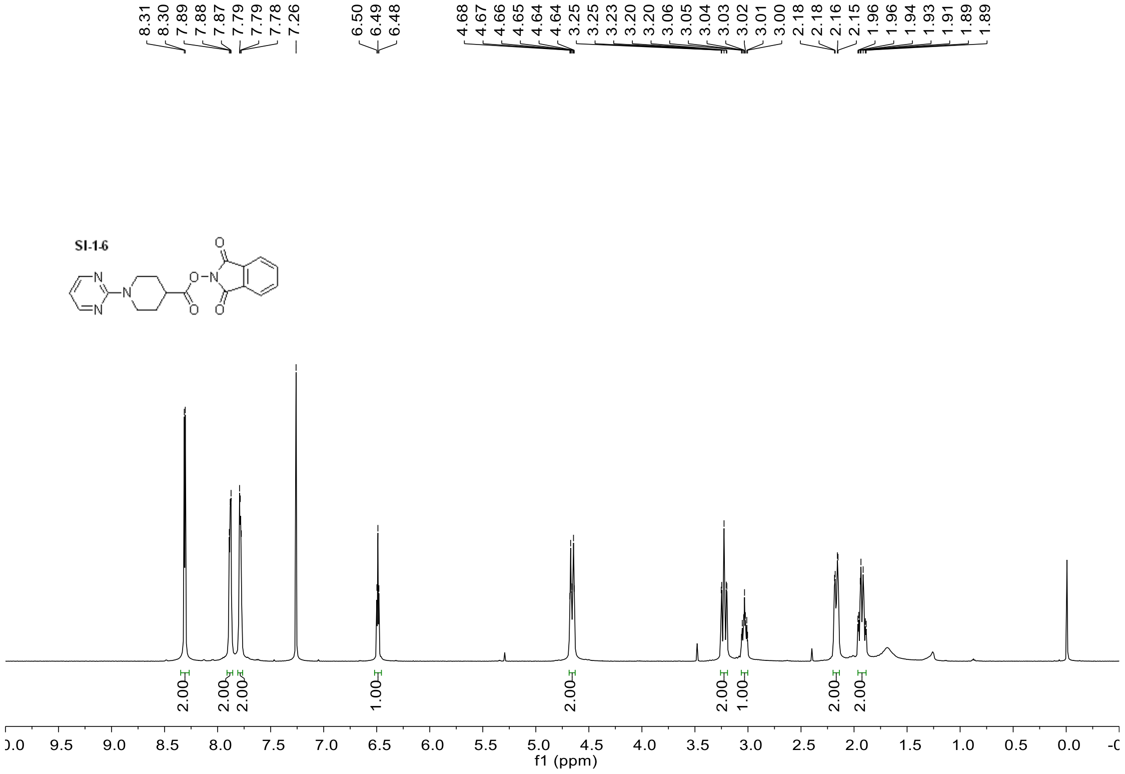

Supplementary Figure 9. ^{1}H NMR Spectra of compound **SI-1-6**.

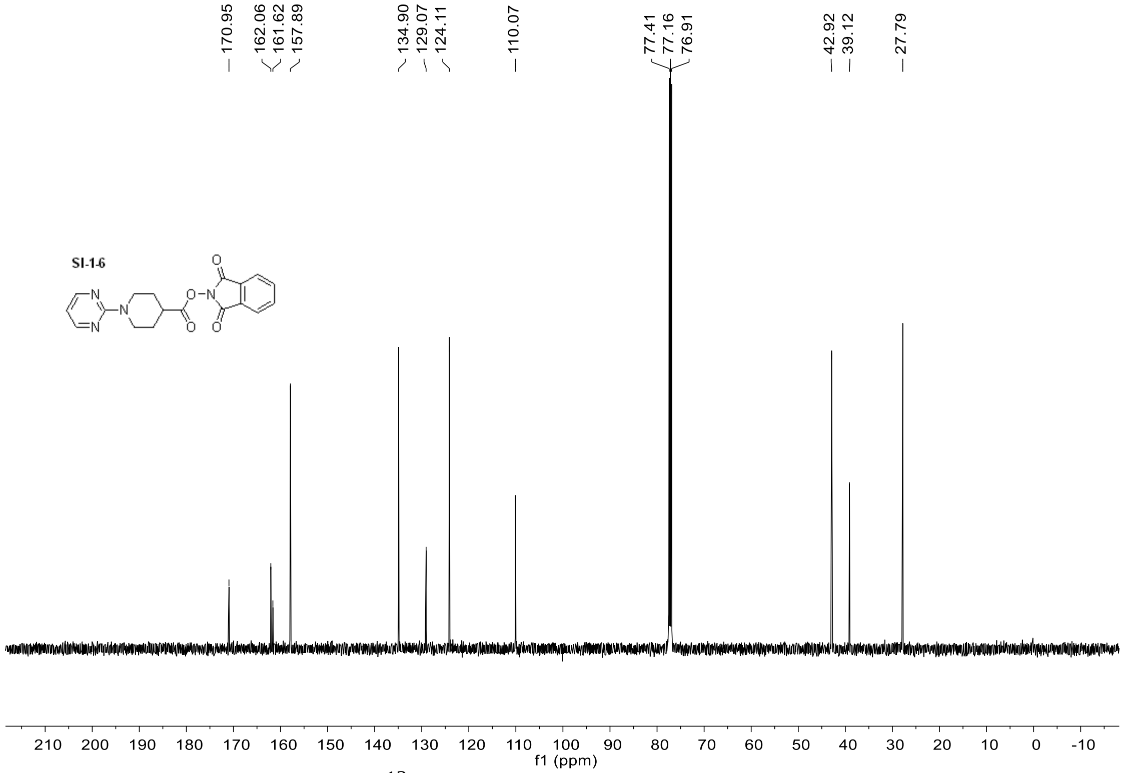

Supplementary Figure 10. ^{13}C NMR Spectra of compound **SI-1-6**.

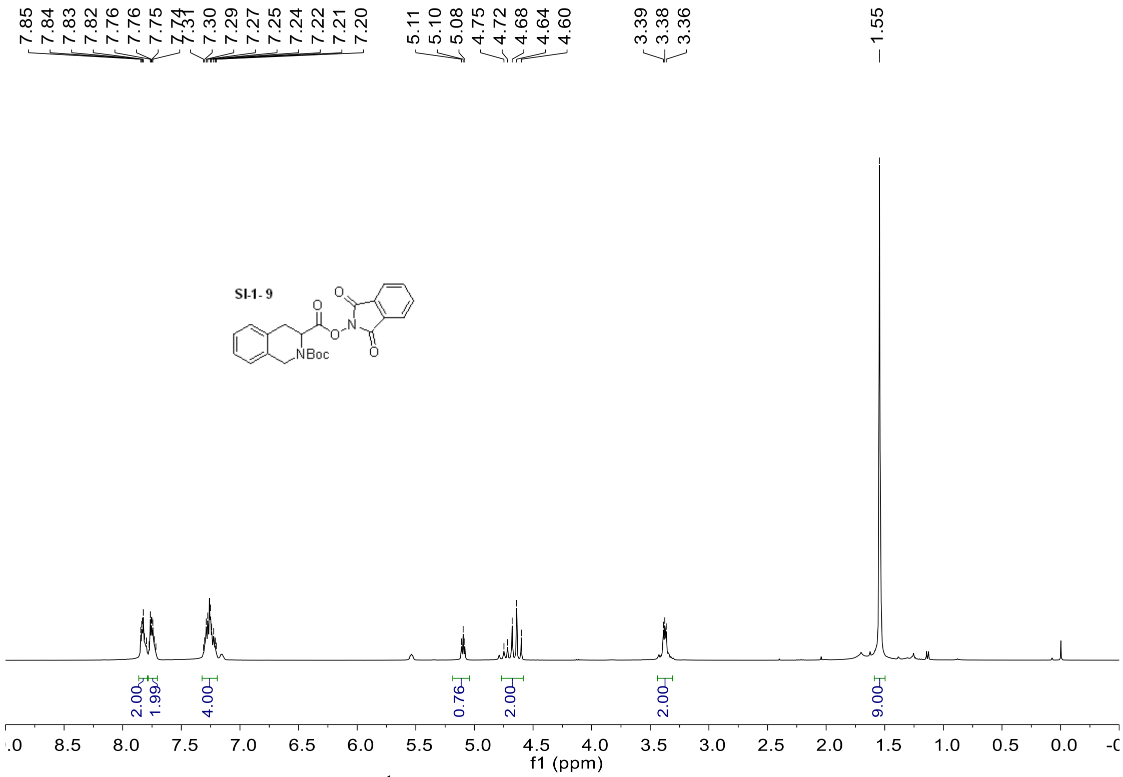

Supplementary Figure 11. ^{1}H NMR Spectra of compound **SI-1-9**.

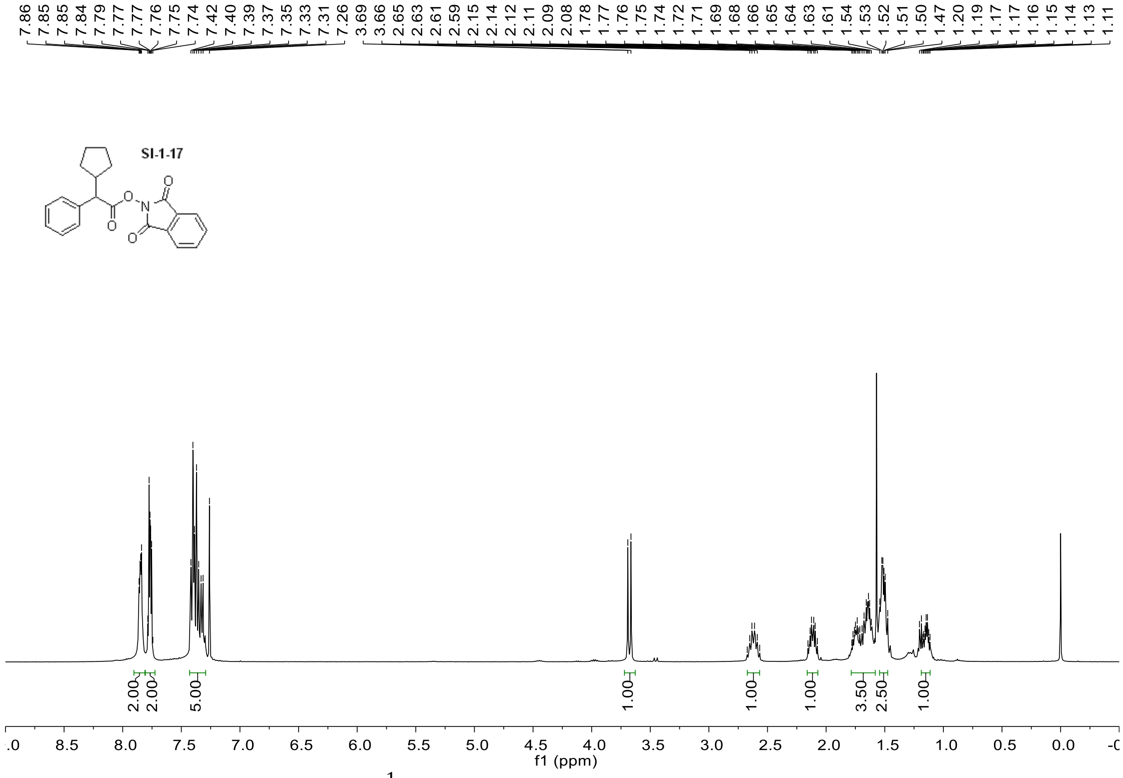

Supplementary Figure 12. ^{1}H NMR Spectra of compound **SI-1-17**.

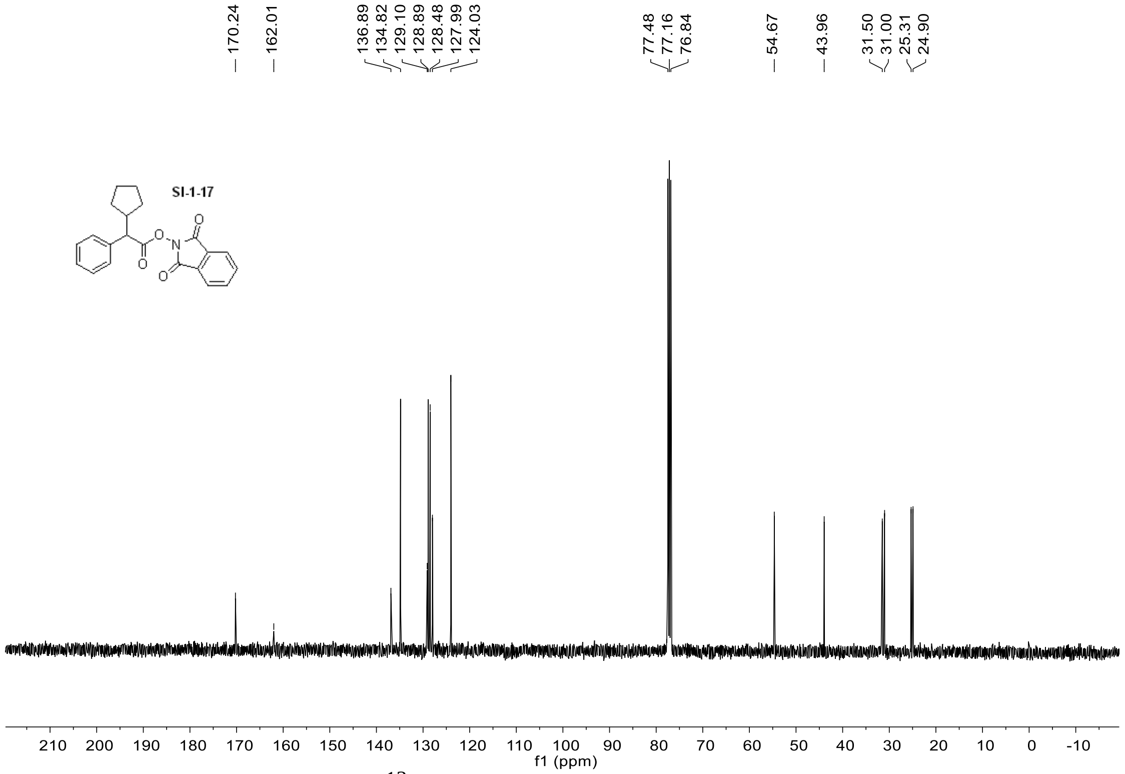

Supplementary Figure 13. ^{13}C NMR Spectra of compound **SI-1-17**.

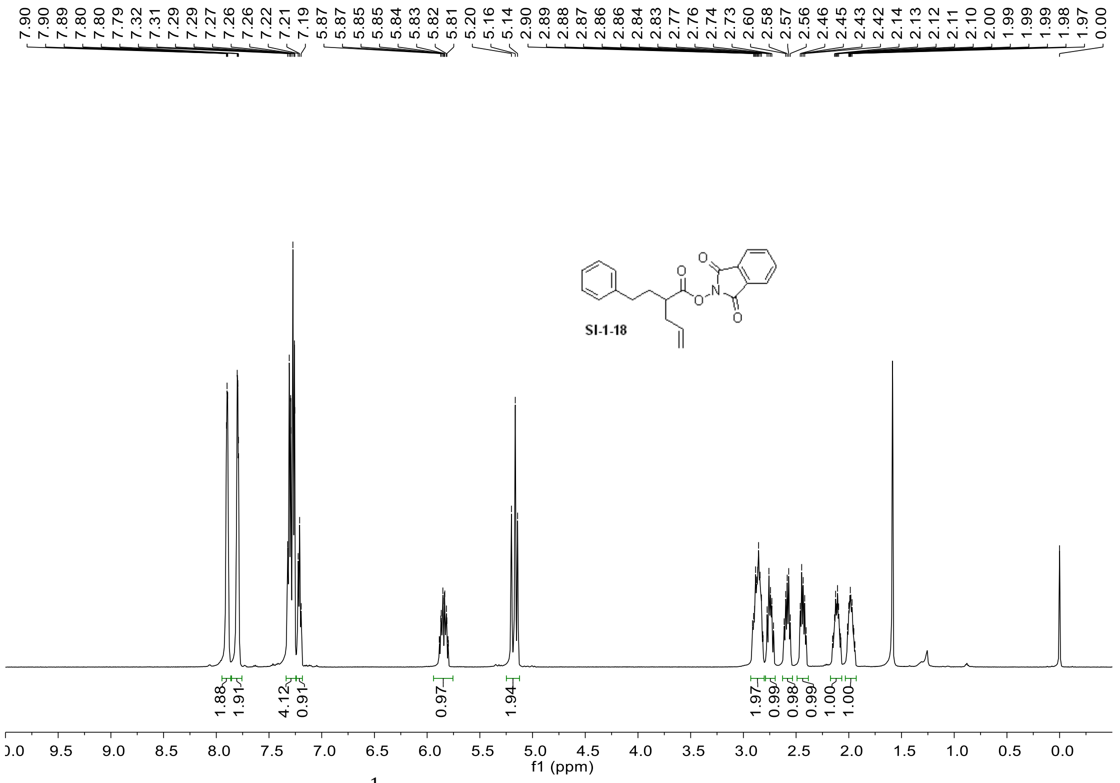

Supplementary Figure 14. ^{1}H NMR Spectra of compound **SI-1-18**.

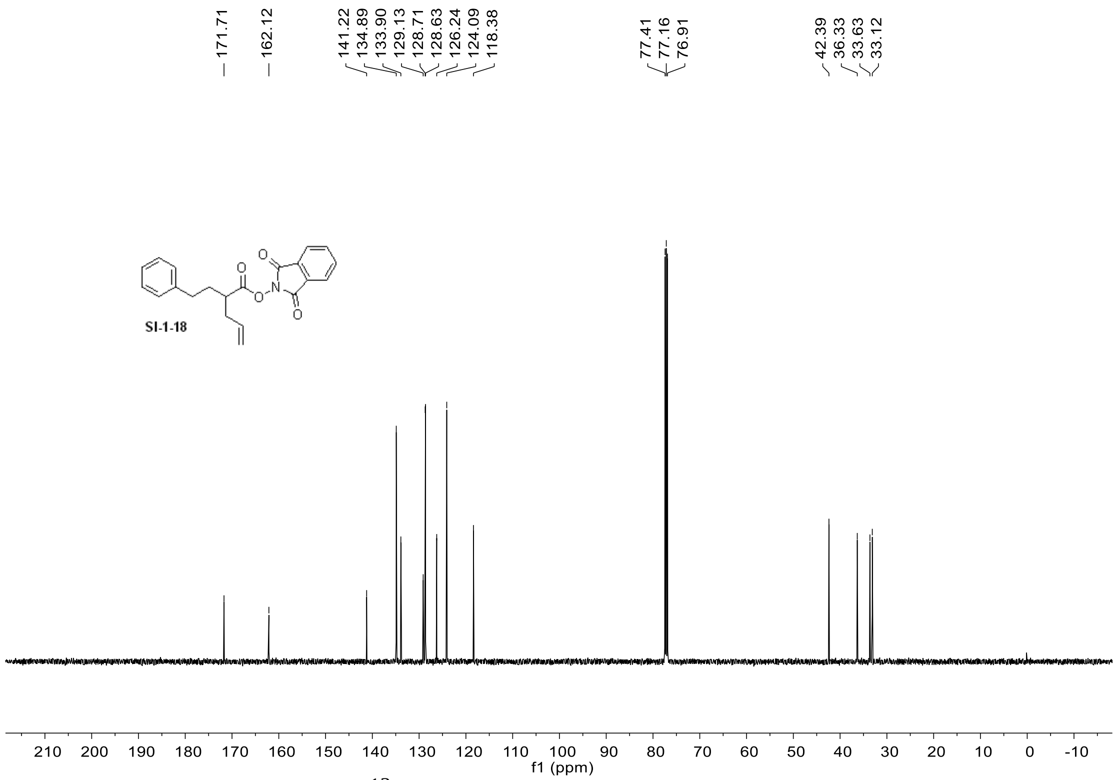

Supplementary Figure 15. ^{13}C NMR Spectra of compound **SI-1-18**.

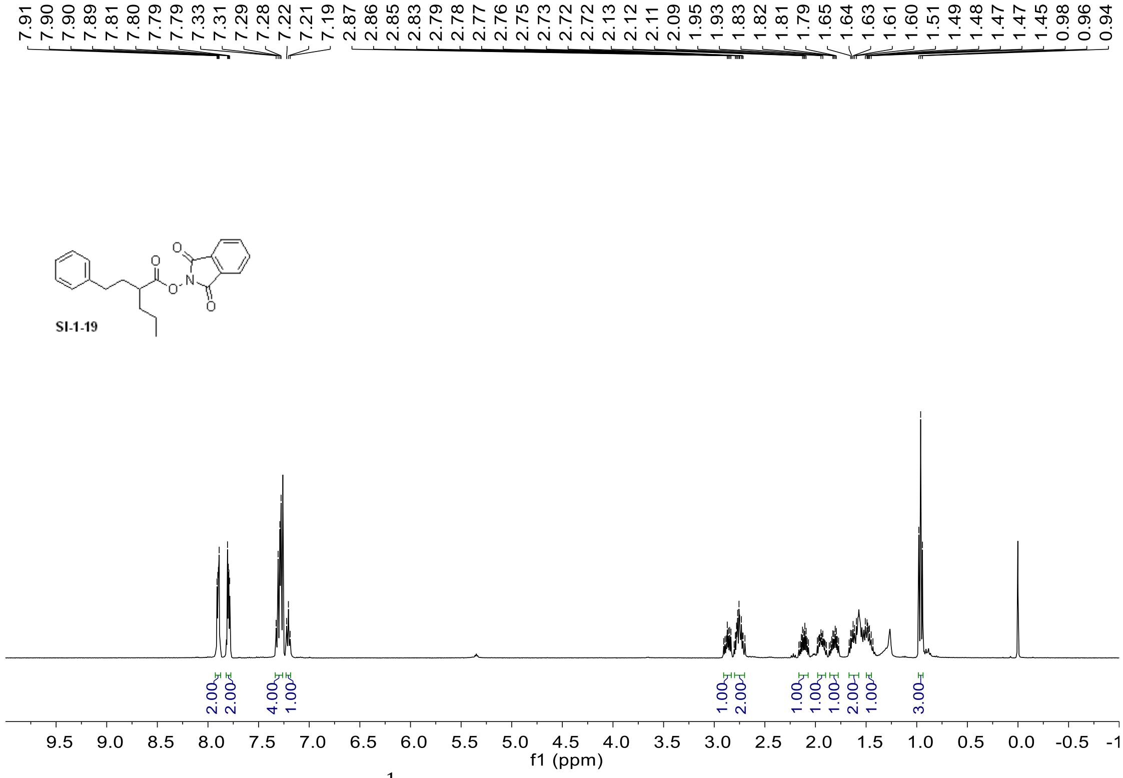

Supplementary Figure 16. ^{1}H NMR Spectra of compound **SI-1-19**.

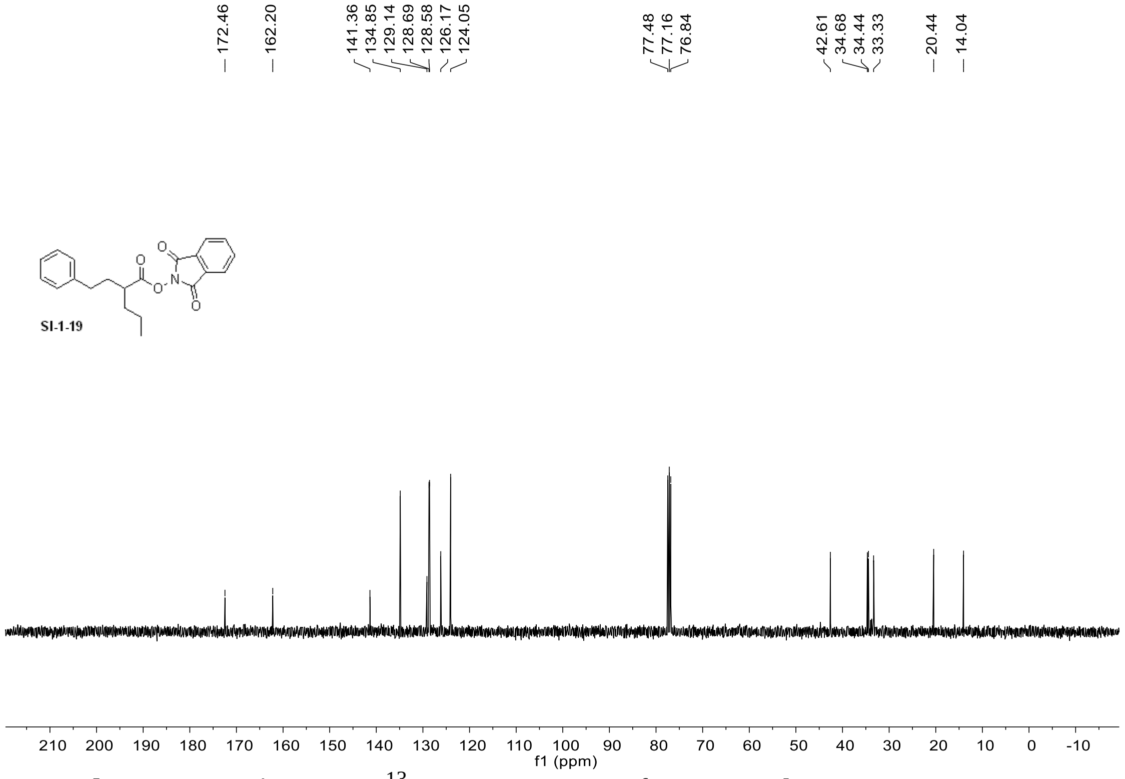

Supplementary Figure 17. ^{13}C NMR Spectra of compound **SI-1-19**.

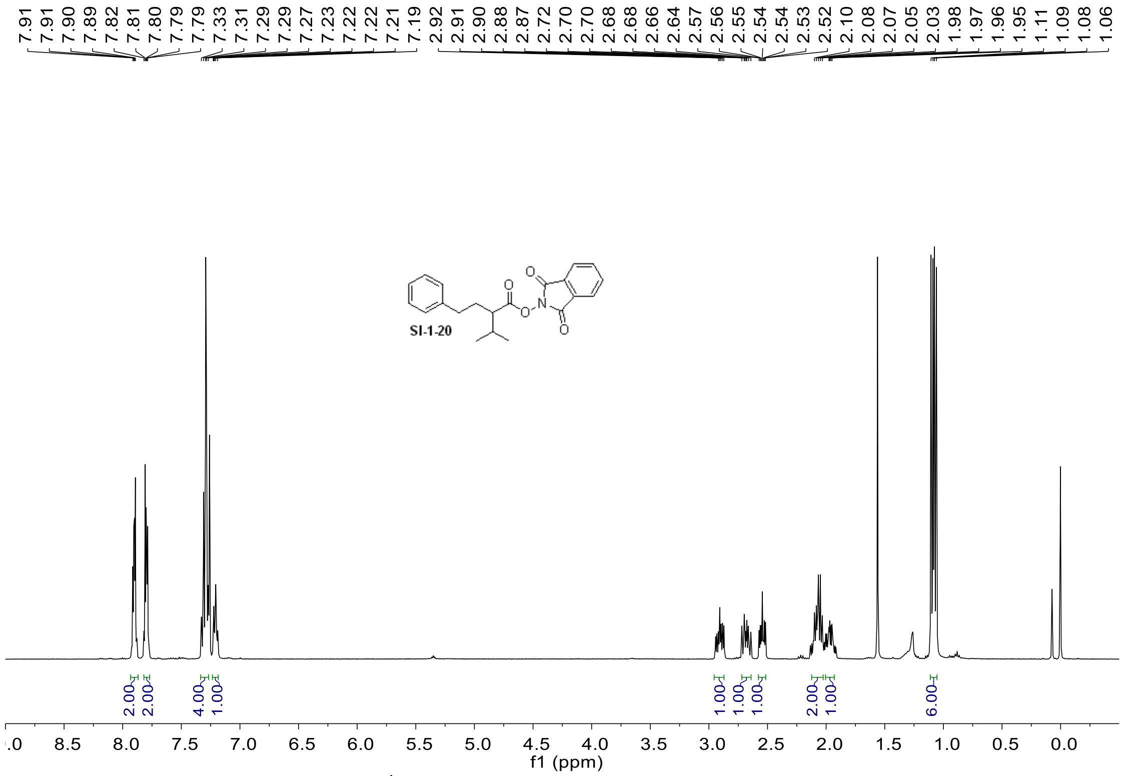

Supplementary Figure 18. ^{1}H NMR Spectra of compound **SI-1-20**.

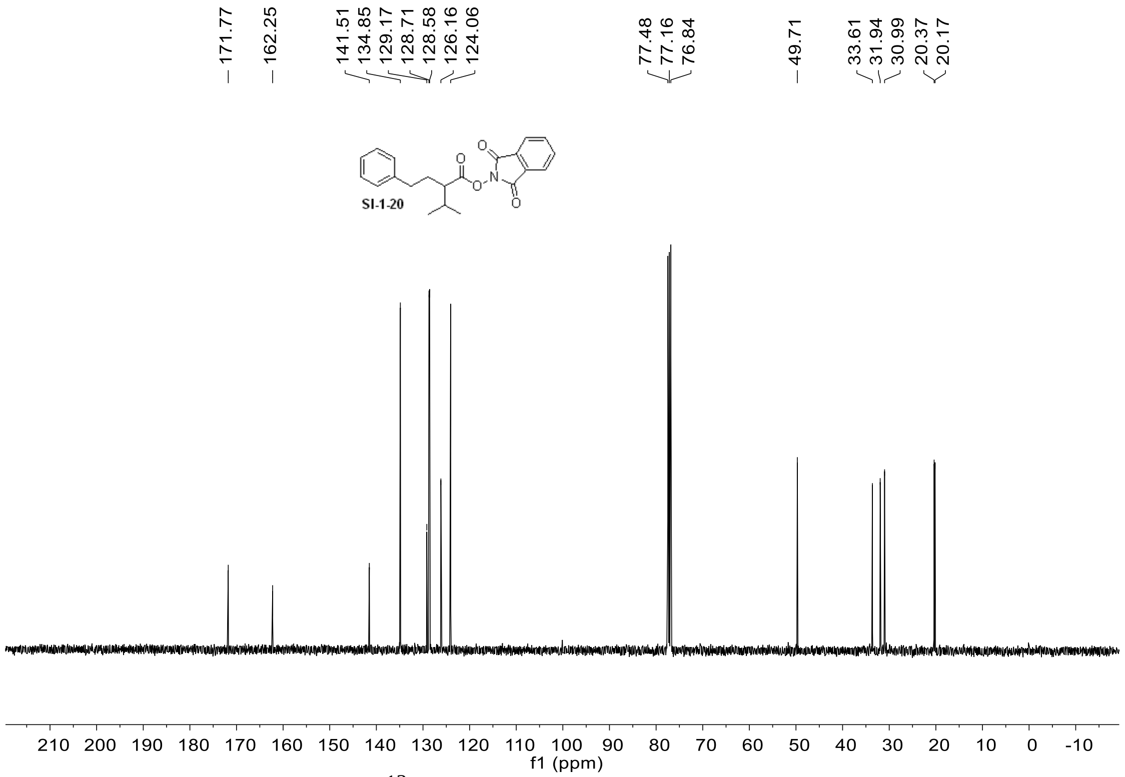

Supplementary Figure 19. ^{13}C NMR Spectra of compound **SI-1-20**.

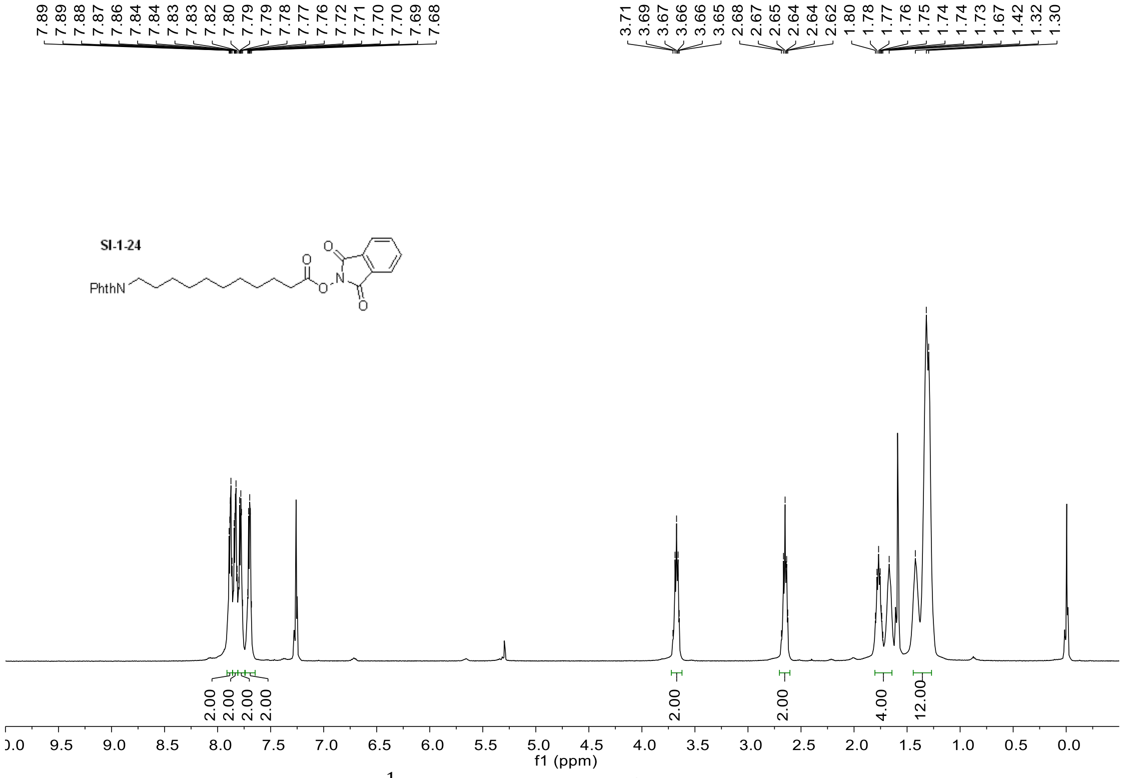

Supplementary Figure 20. ^{1}H NMR Spectra of compound **SI-1-24**.

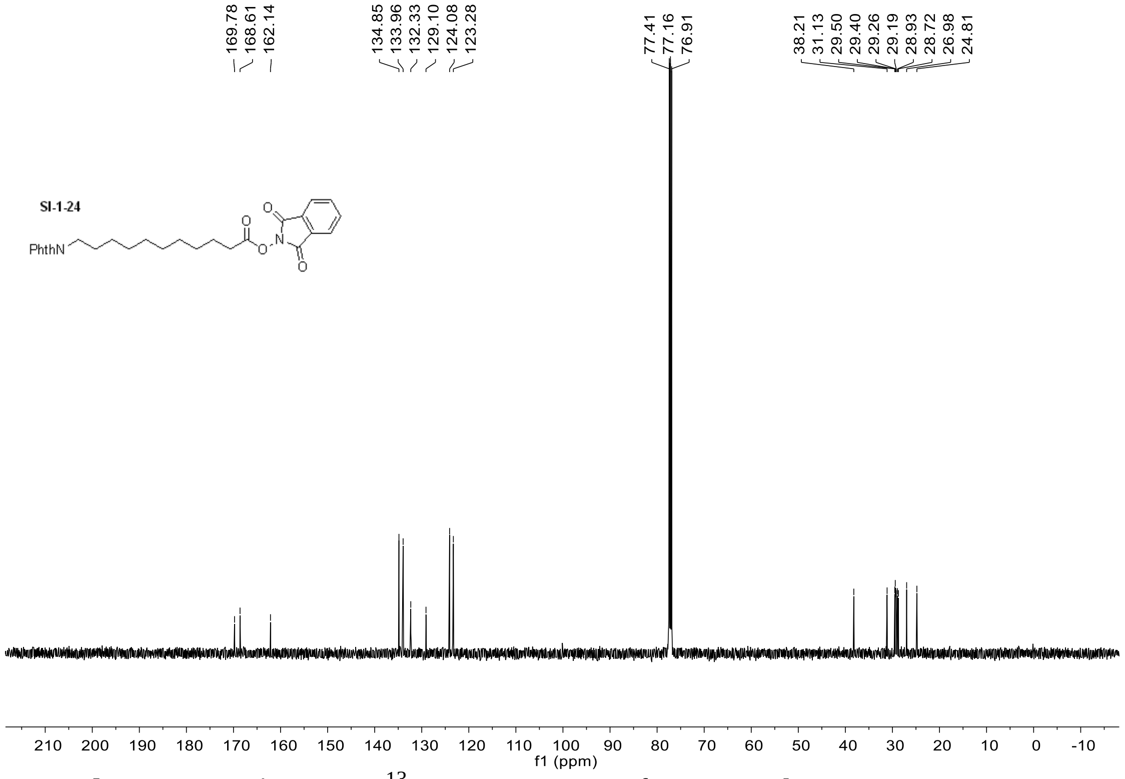

Supplementary Figure 21. ^{13}C NMR Spectra of compound **SI-1-24**.

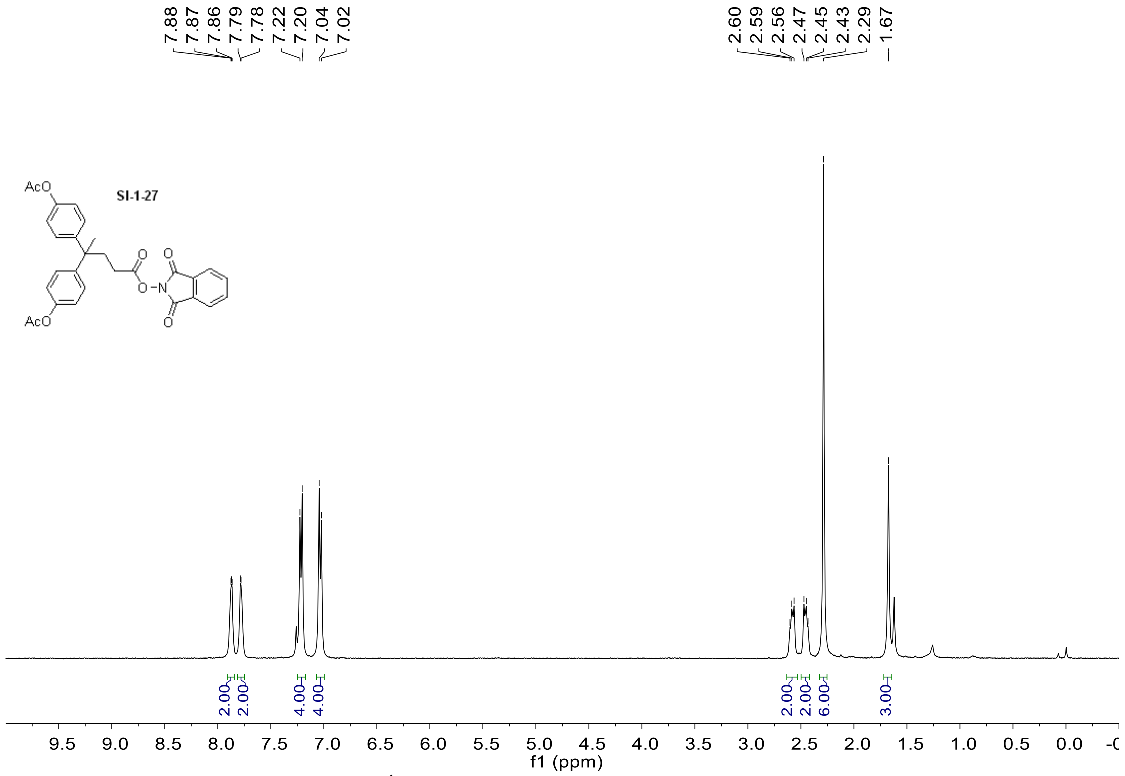

Supplementary Figure 22. ^{1}H NMR Spectra of compound **SI-1-27**.

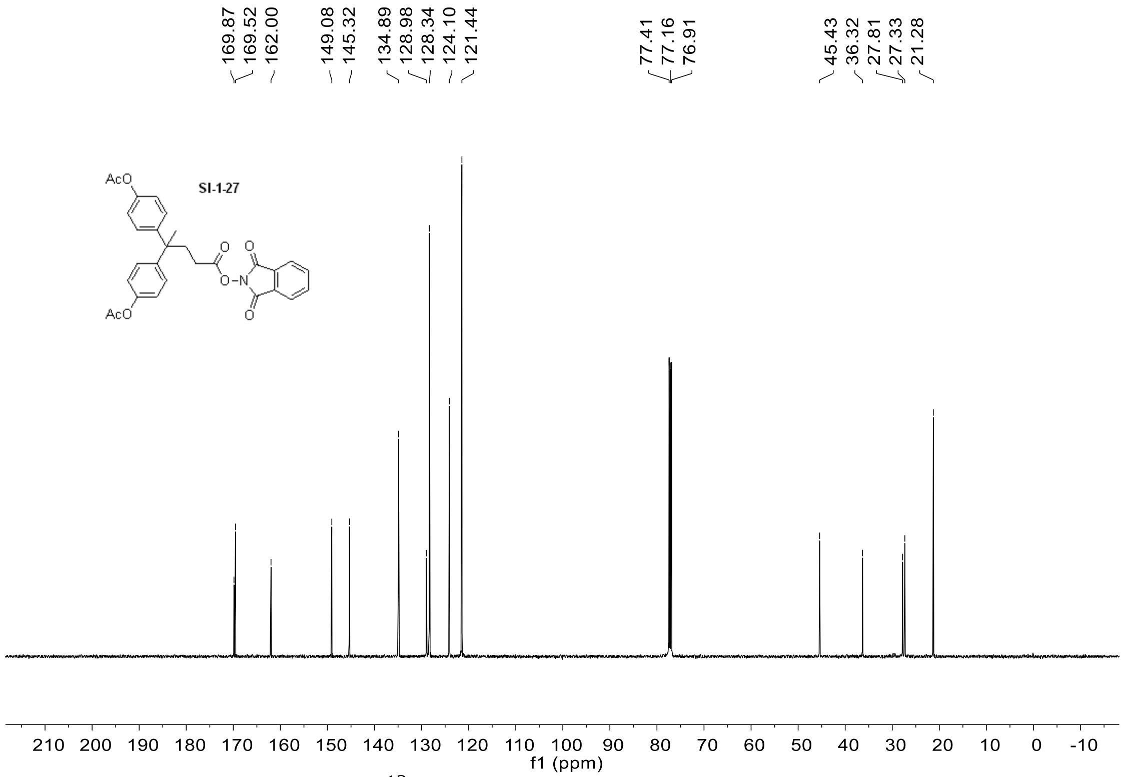

Supplementary Figure 23. ^{13}C NMR Spectra of compound **SI-1-27**.

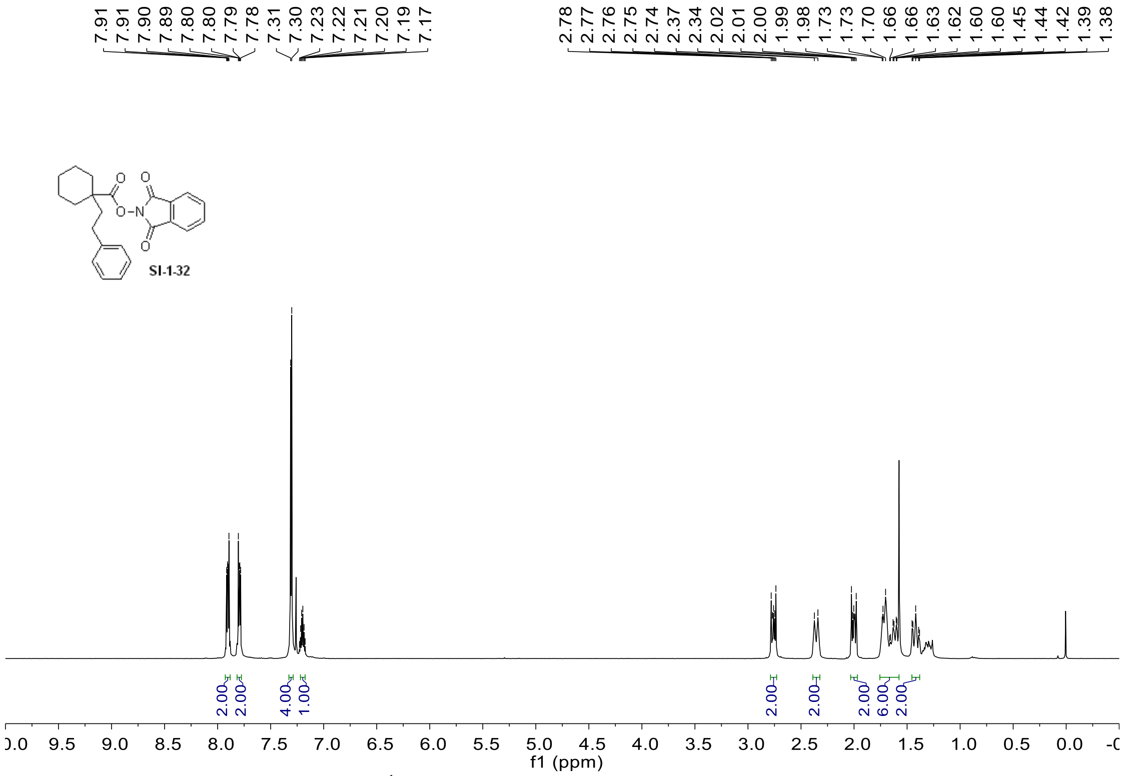

Supplementary Figure 24. ^{1}H NMR Spectra of compound **SI-1-32**.

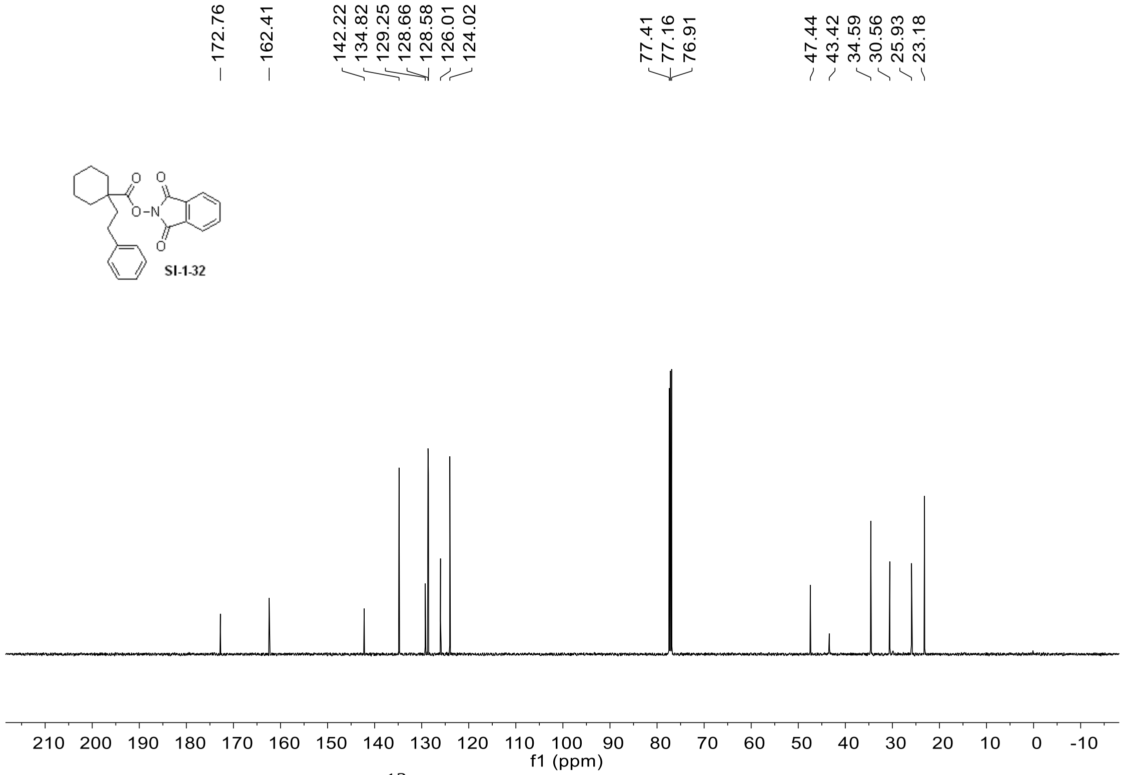

Supplementary Figure 25. ^{13}C NMR Spectra of compound **SI-1-32**.

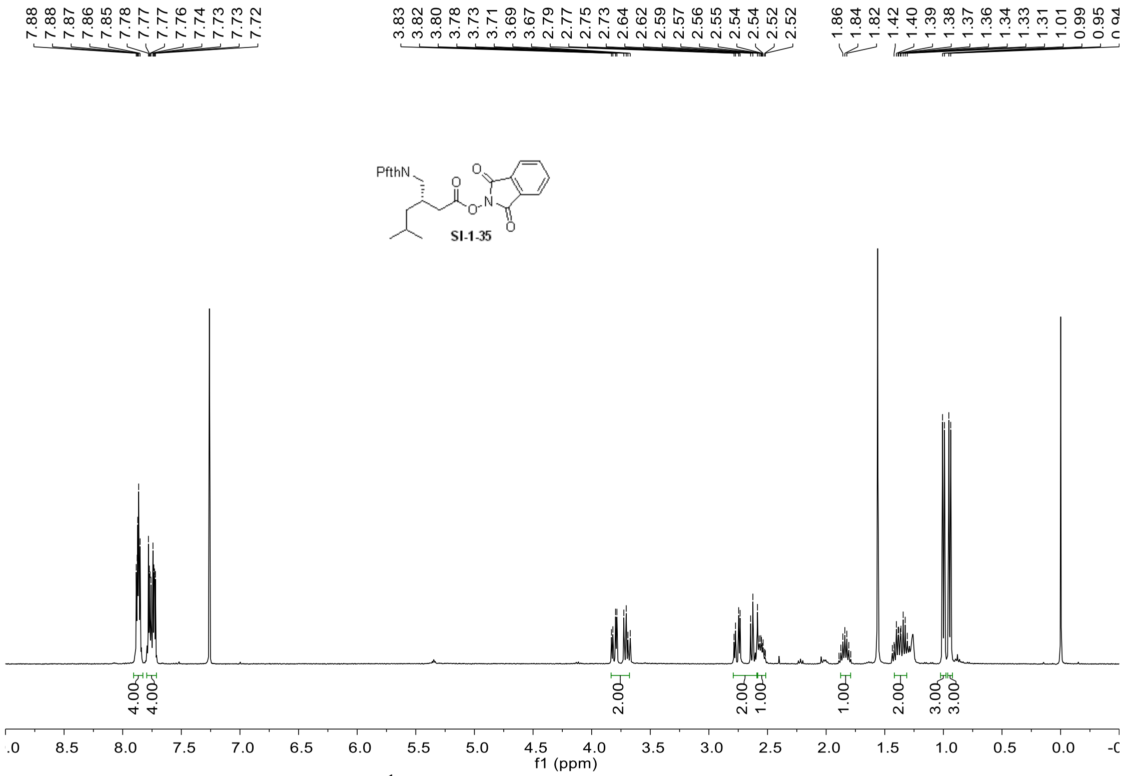

Supplementary Figure 26. ^{1}H NMR Spectra of compound **SI-1-35**.

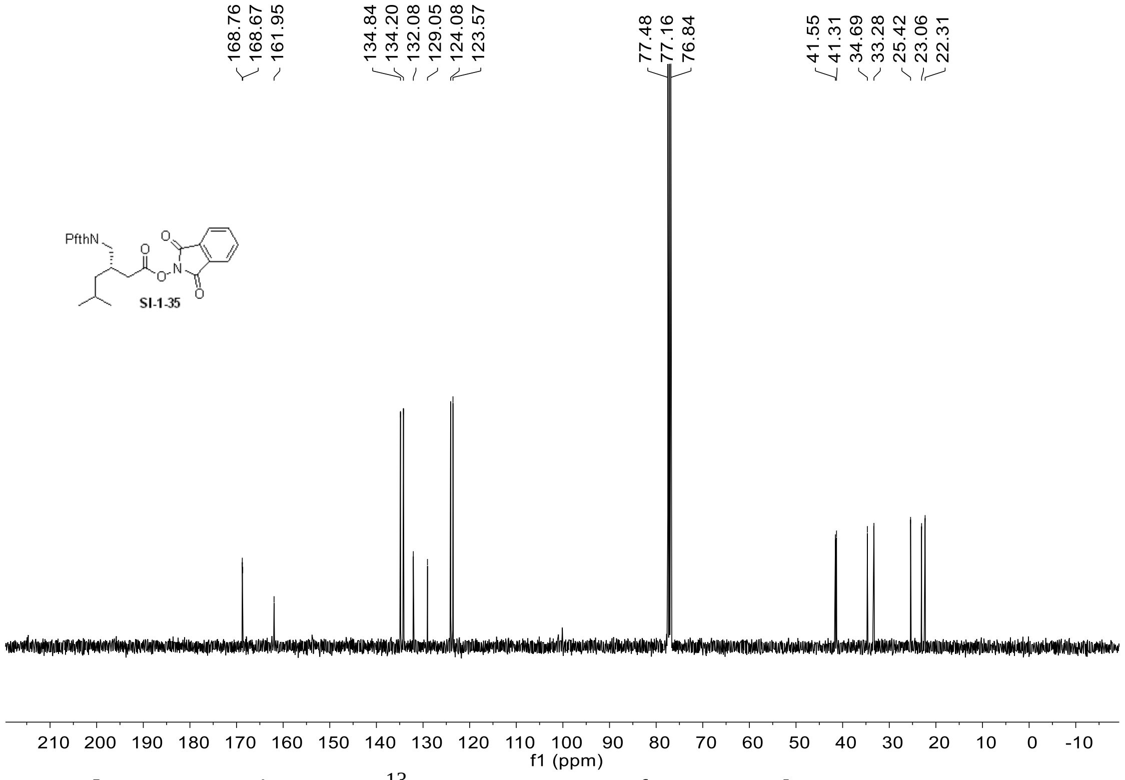

Supplementary Figure 27. ^{13}C NMR Spectra of compound **SI-1-35**.

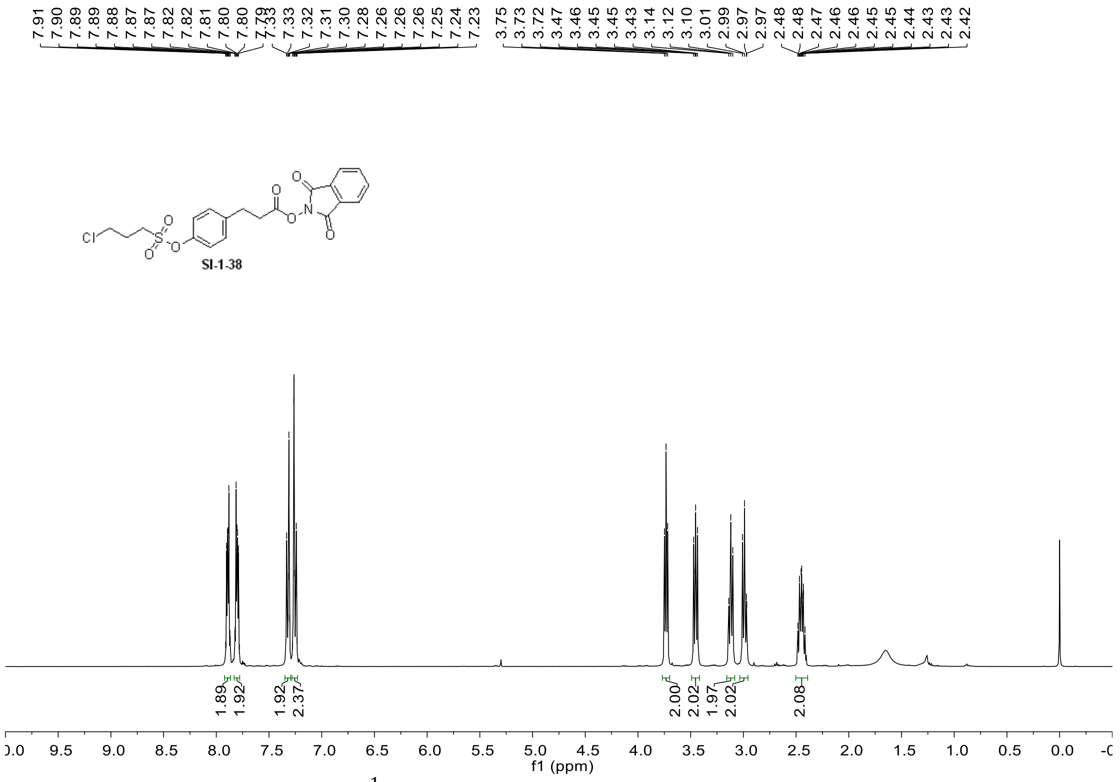

Supplementary Figure 28. ^{1}H NMR Spectra of compound **SI-1-38**.

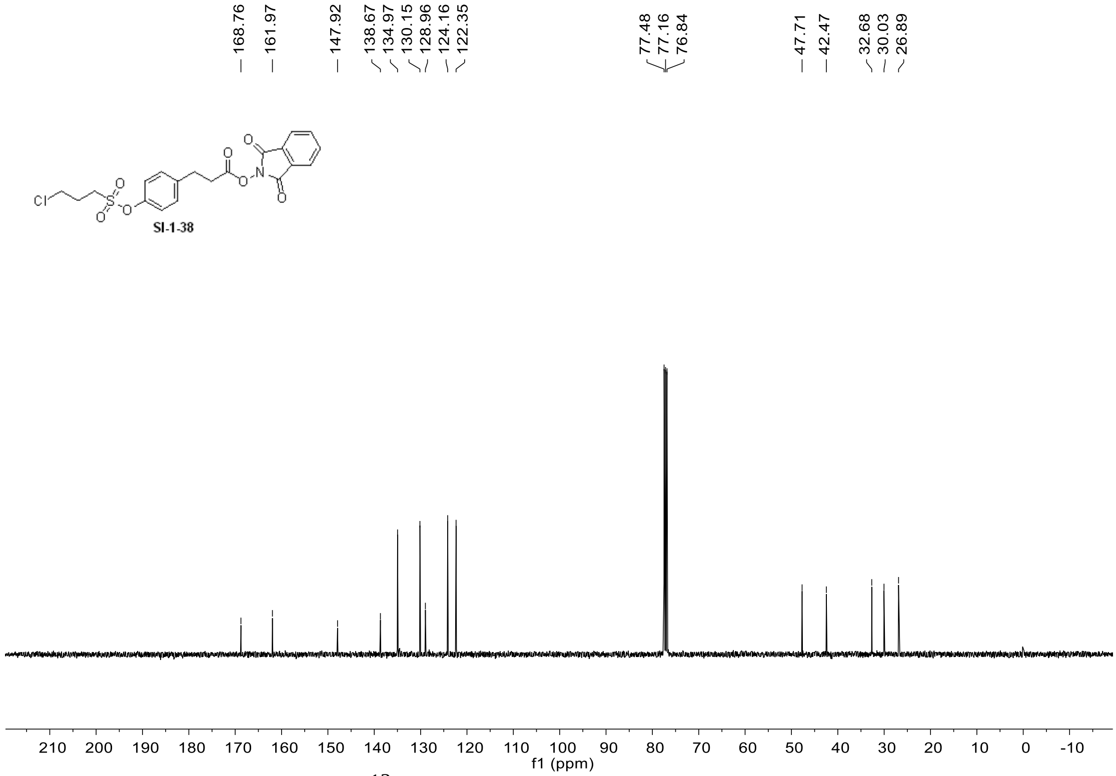

Supplementary Figure 29. ^{13}C NMR Spectra of compound **SI-1-28**.

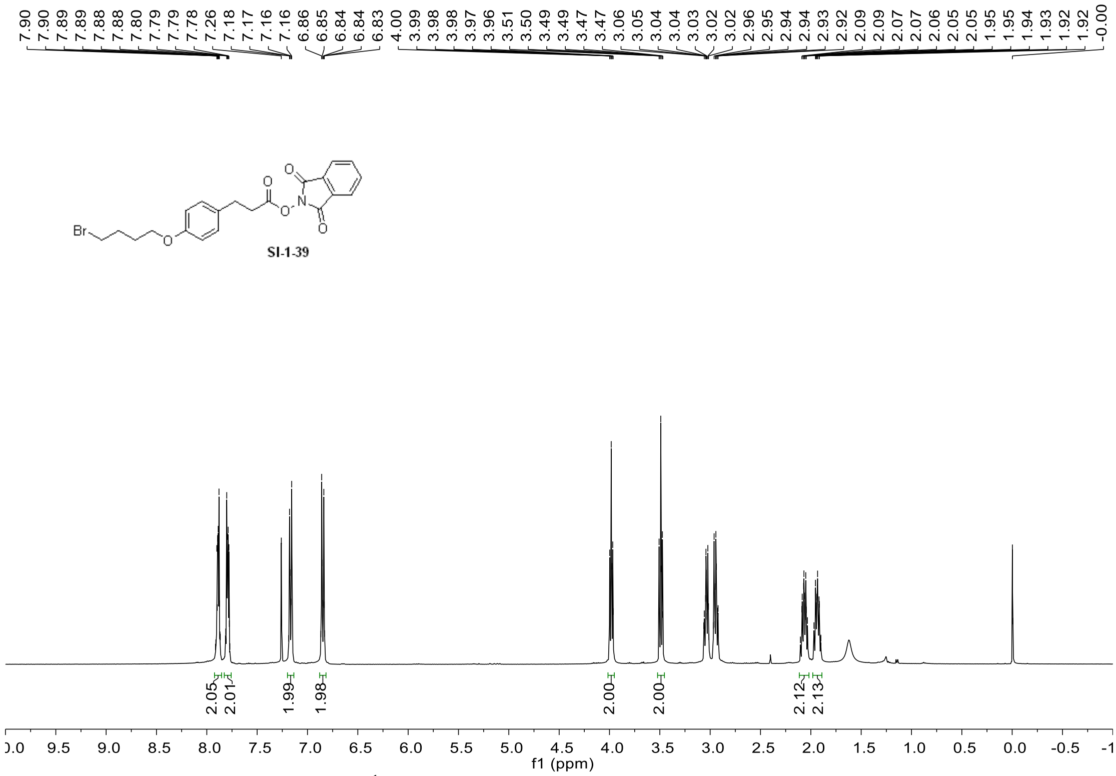

Supplementary Figure 30. ^{1}H NMR Spectra of compound **SI-1-39**.

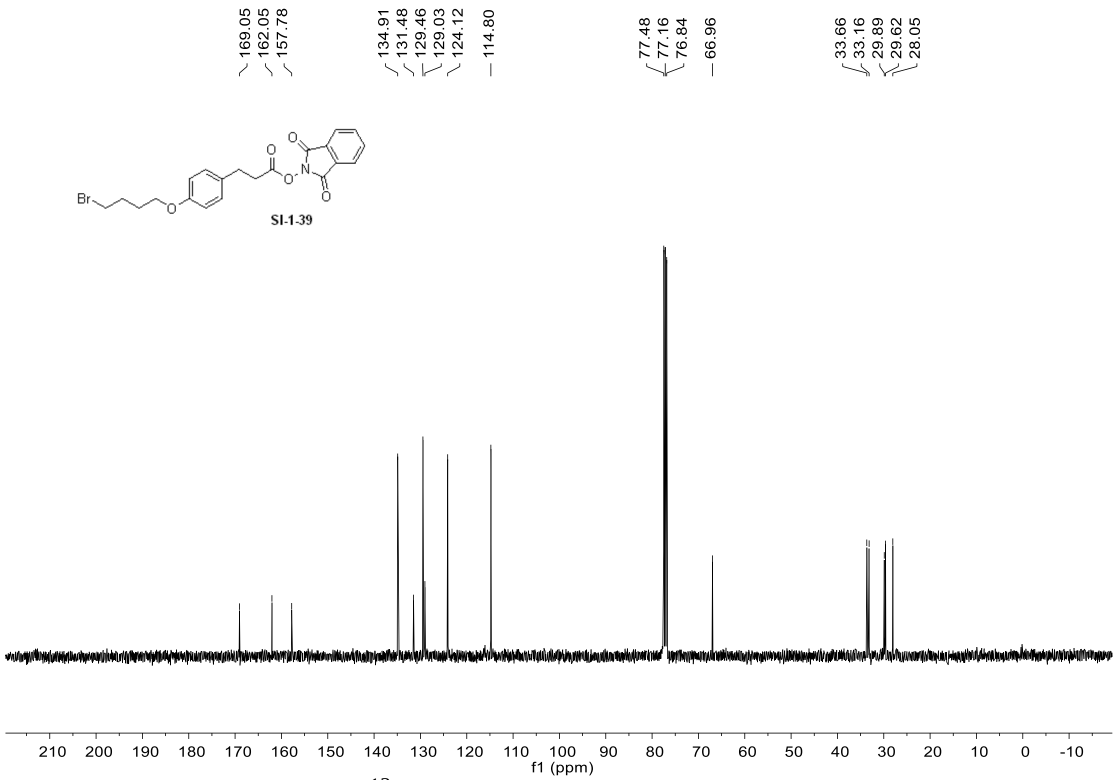

Supplementary Figure 31. ^{13}C NMR Spectra of compound **SI-1-39**.

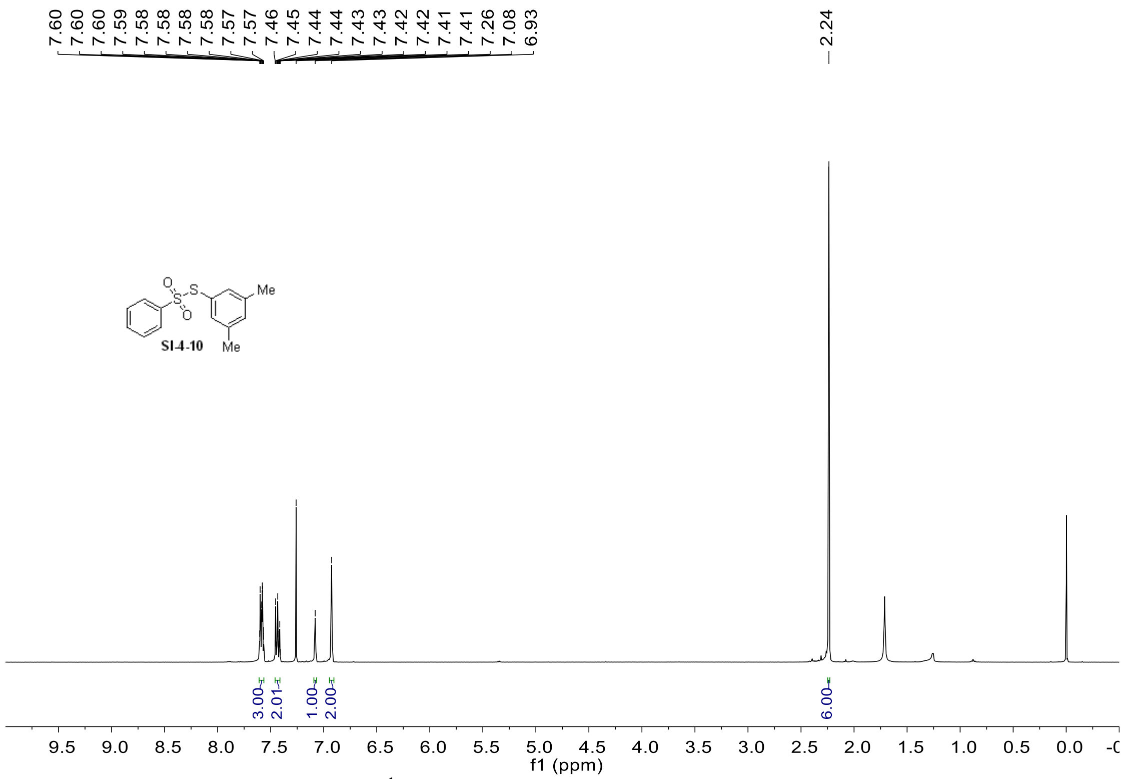

Supplementary Figure 32. ^{1}H NMR Spectra of compound **SI-4-10**.

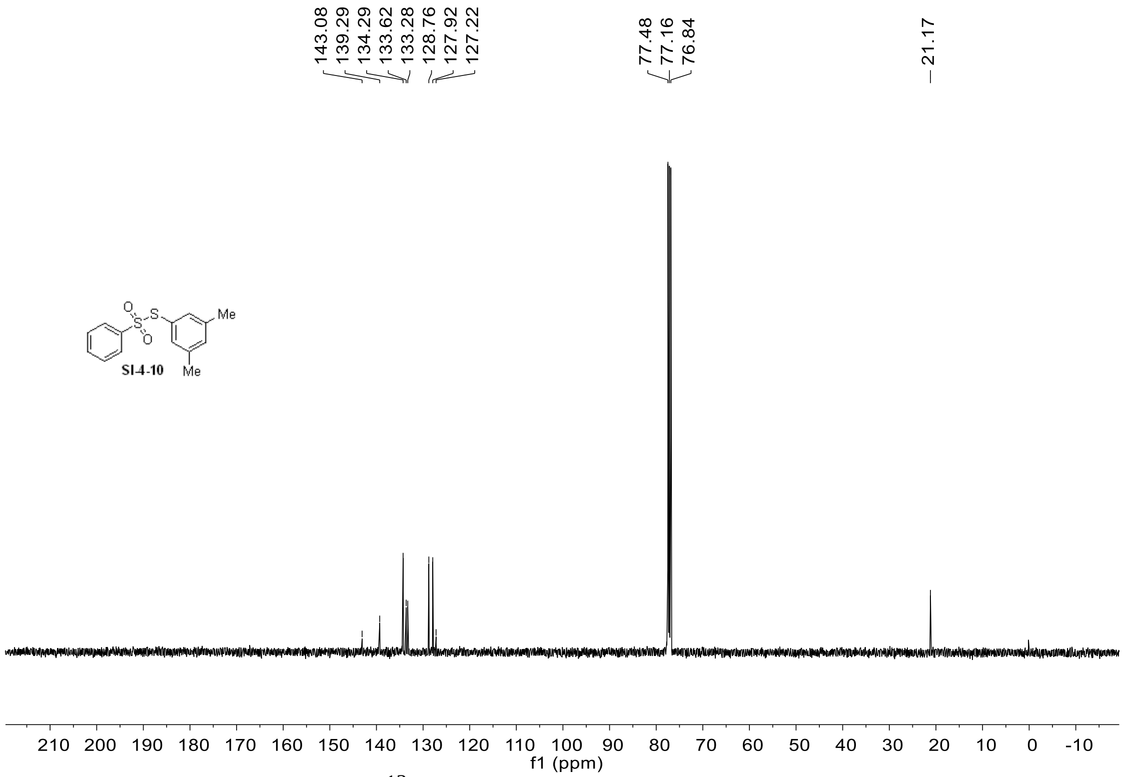

Supplementary Figure 33. ^{13}C NMR Spectra of compound **SI-4-10**.

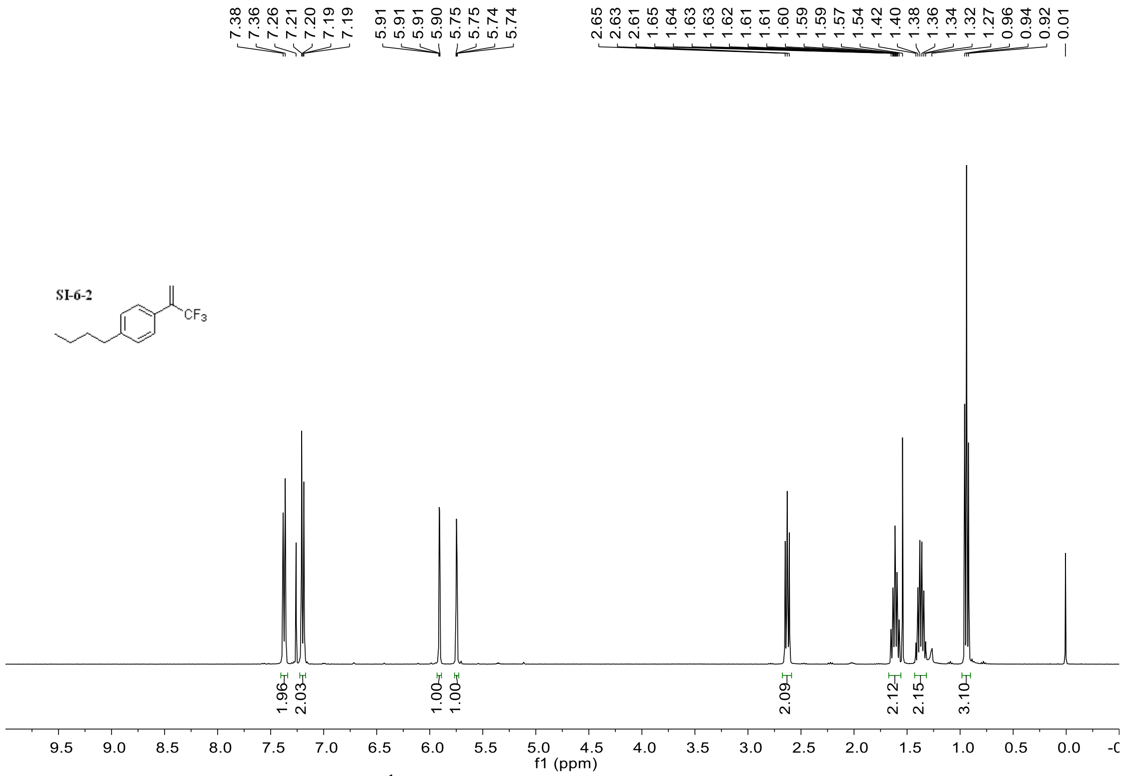

Supplementary Figure 34. ^{1}H NMR Spectra of compound **SI-6-2**.

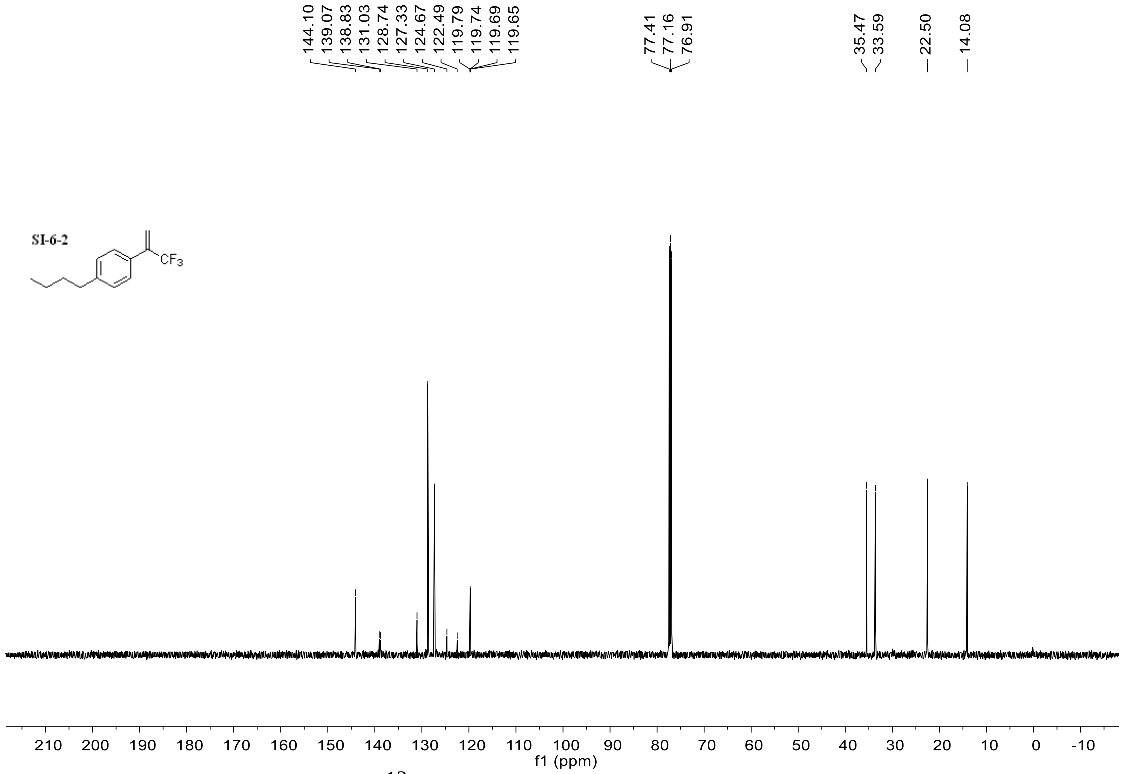

Supplementary Figure 35. ^{13}C NMR Spectra of compound **SI-6-2**.

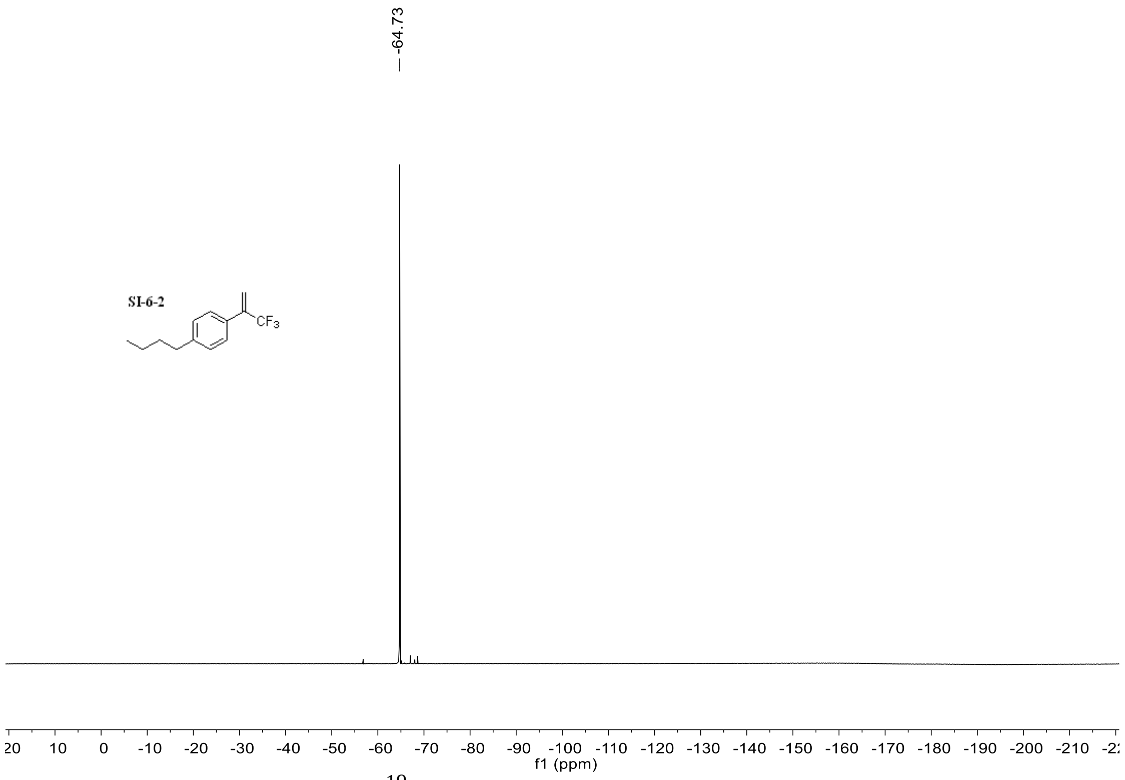

Supplementary Figure 36. ^{19}F NMR Spectra of compound **SI-6-2**.

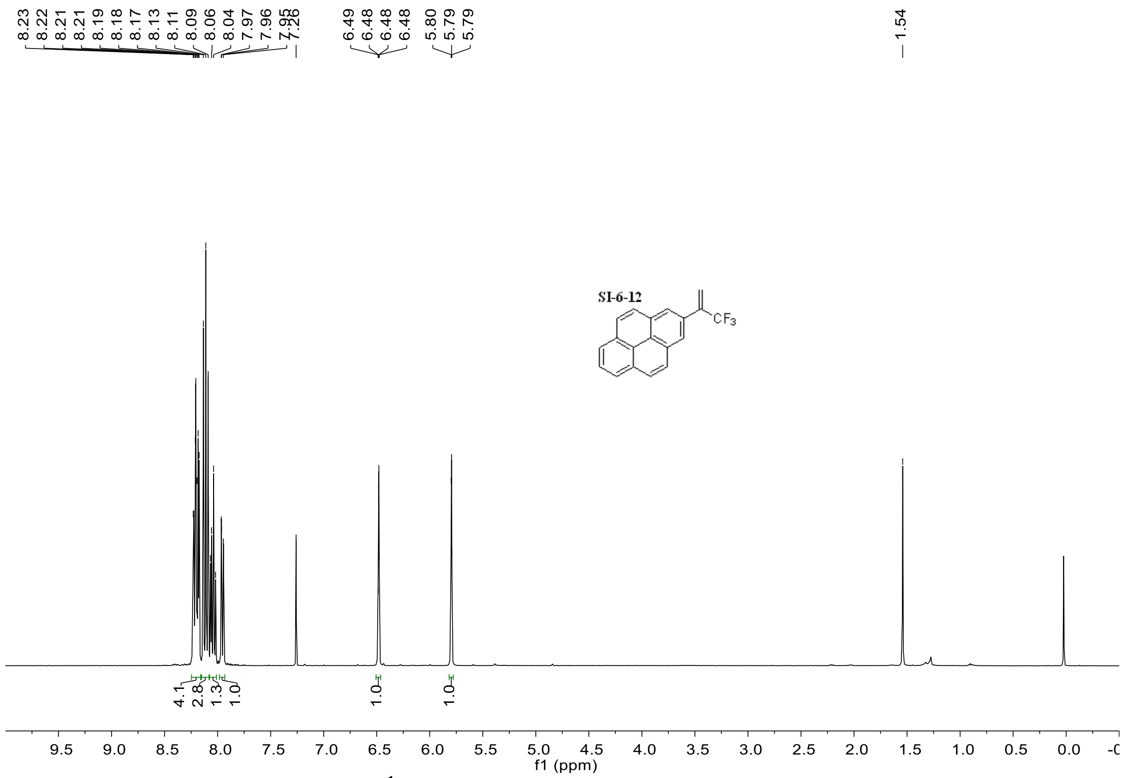

Supplementary Figure 37. ^{1}H NMR Spectra of compound **SI-6-12**.

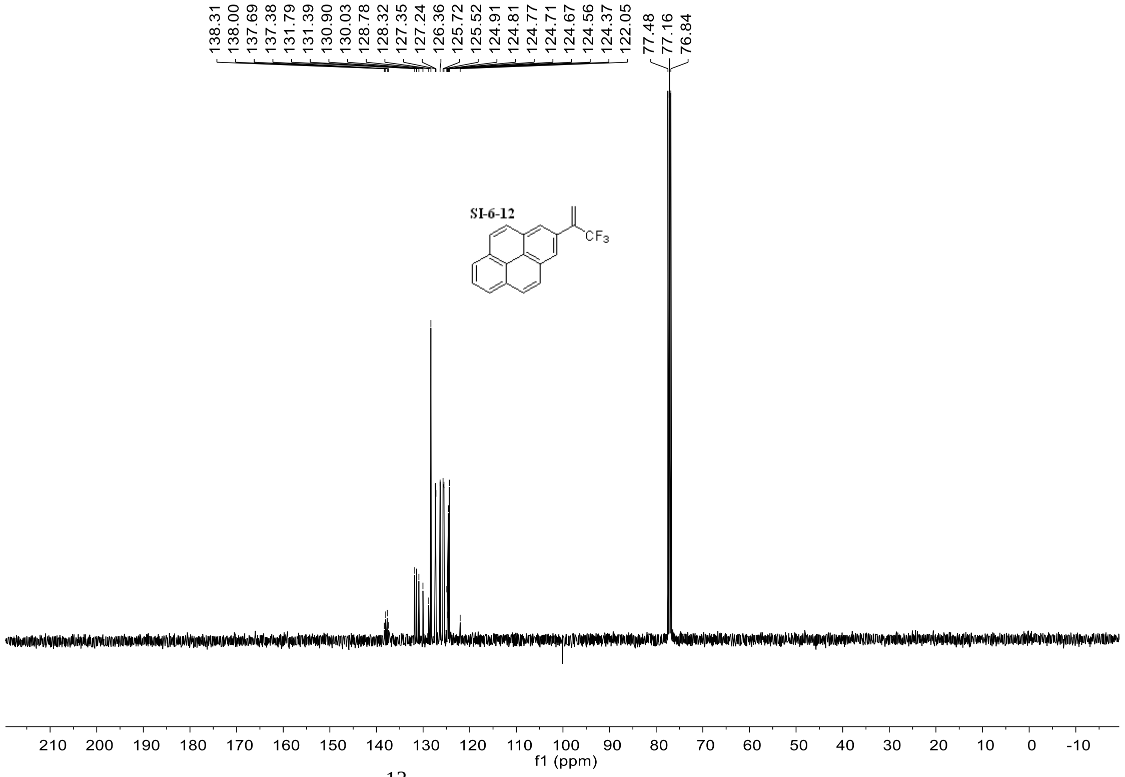

Supplementary Figure 38. ^{13}C NMR Spectra of compound **SI-6-12**.

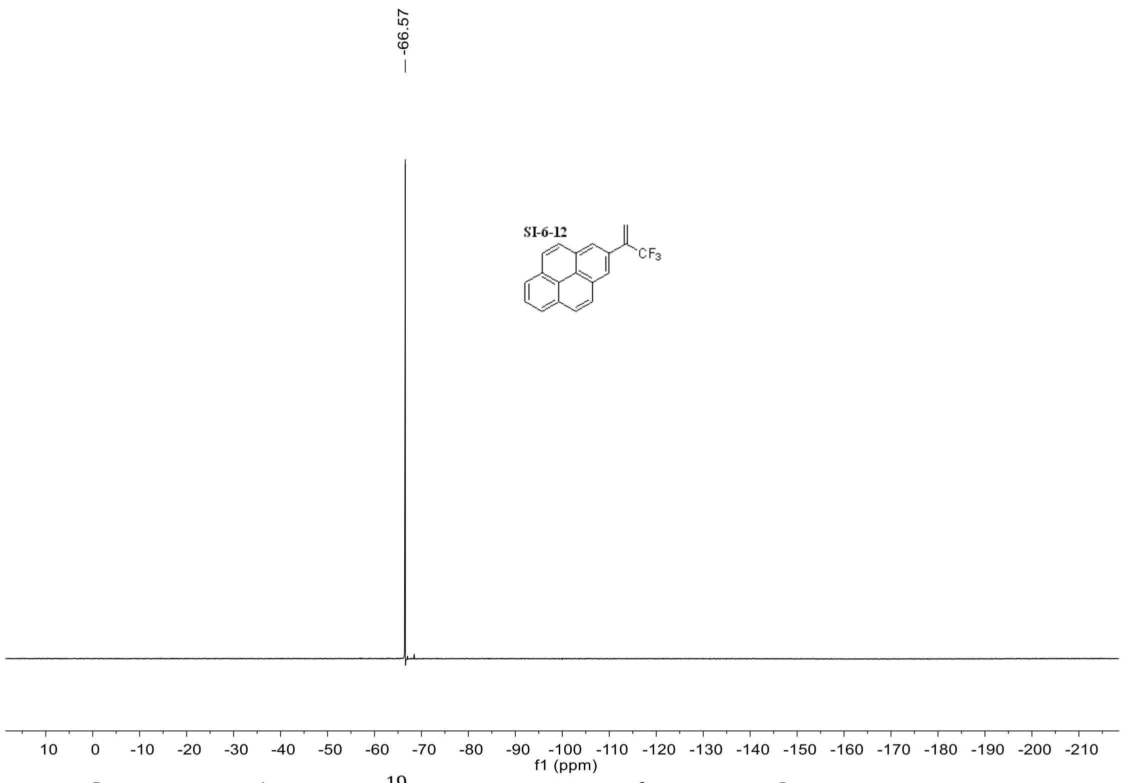

Supplementary Figure 39. ^{19}F NMR Spectra of compound **SI-6-12**.

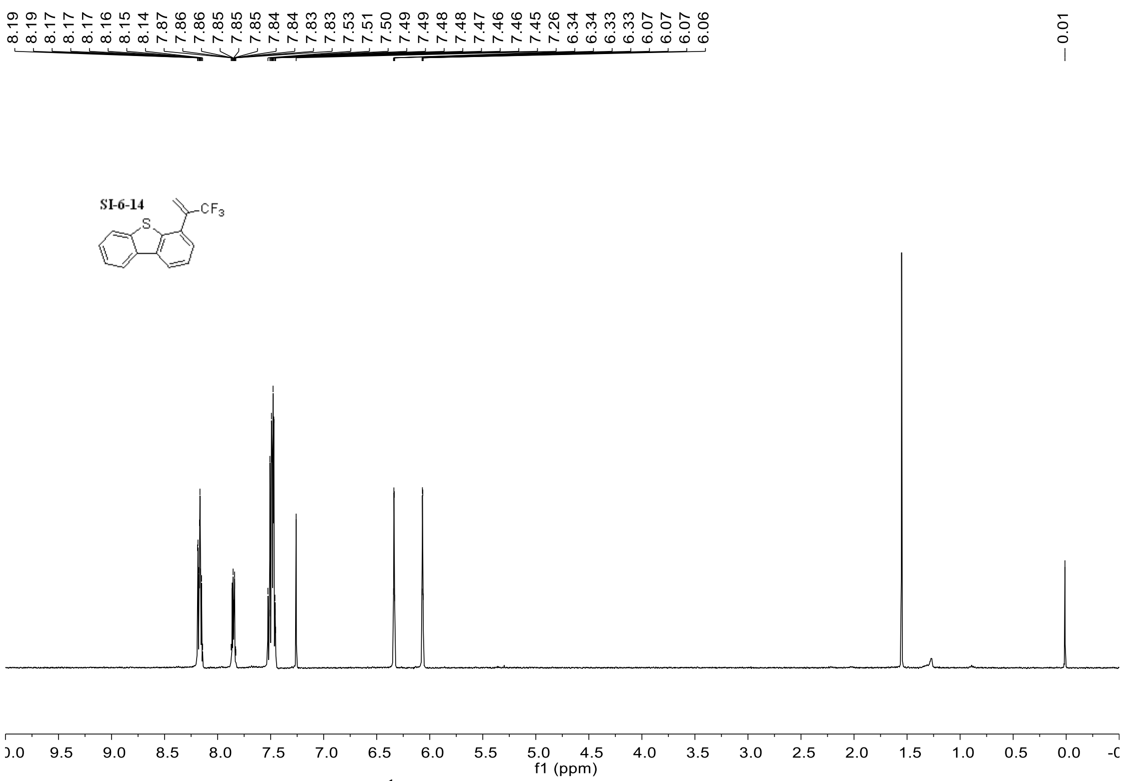

Supplementary Figure 40. ^{1}H NMR Spectra of compound **SI-6-14**.

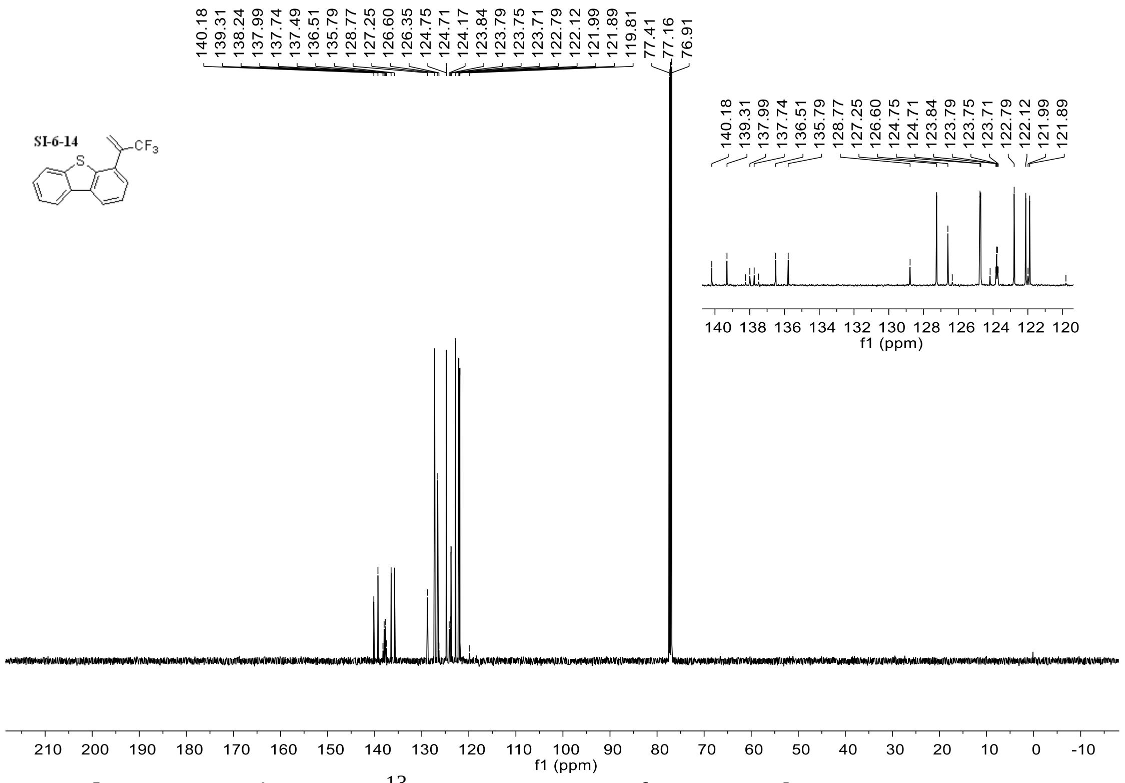

Supplementary Figure 41. ^{13}C NMR Spectra of compound **SI-6-14**.

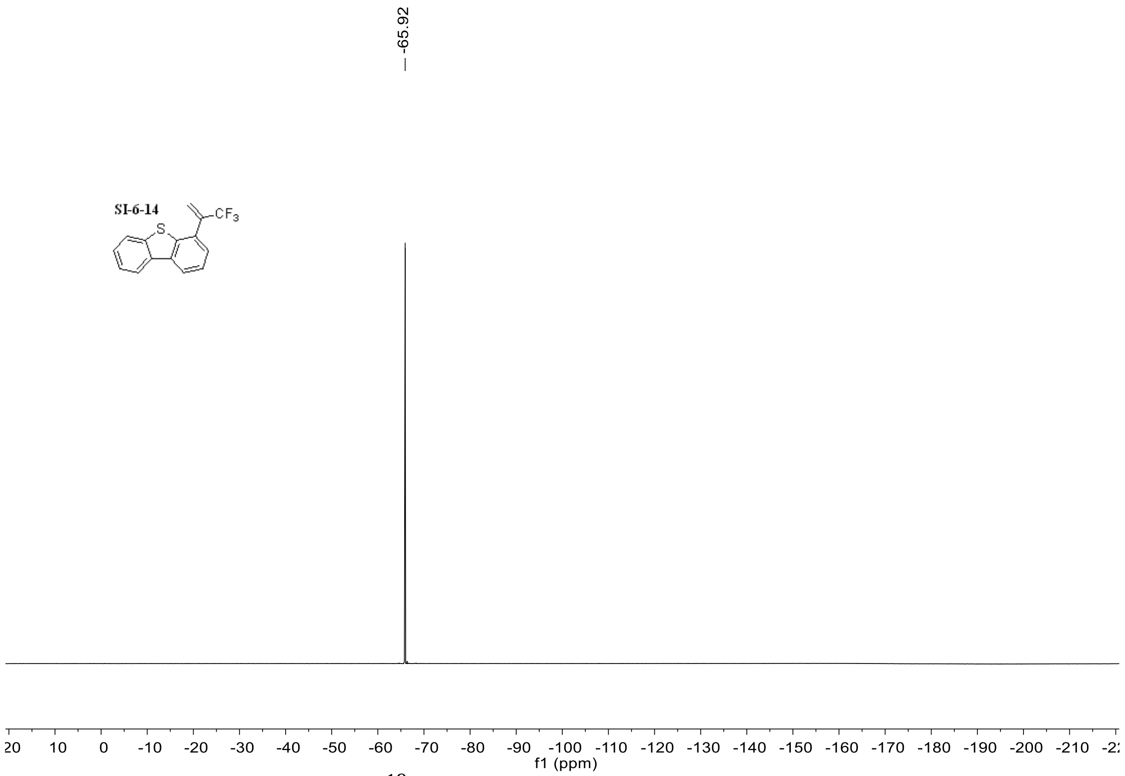

Supplementary Figure 42. ^{19}F NMR Spectra of compound **SI-6-14**.

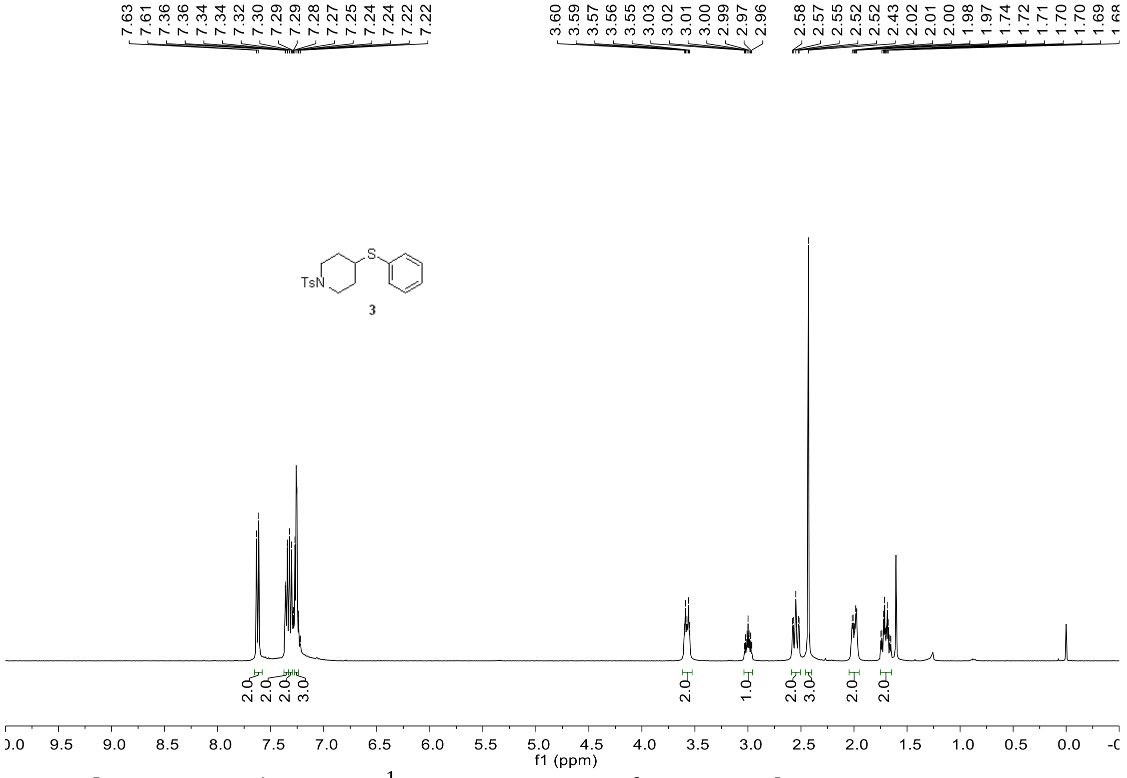

Supplementary Figure 43. ^{1}H NMR Spectra of compound **3**.

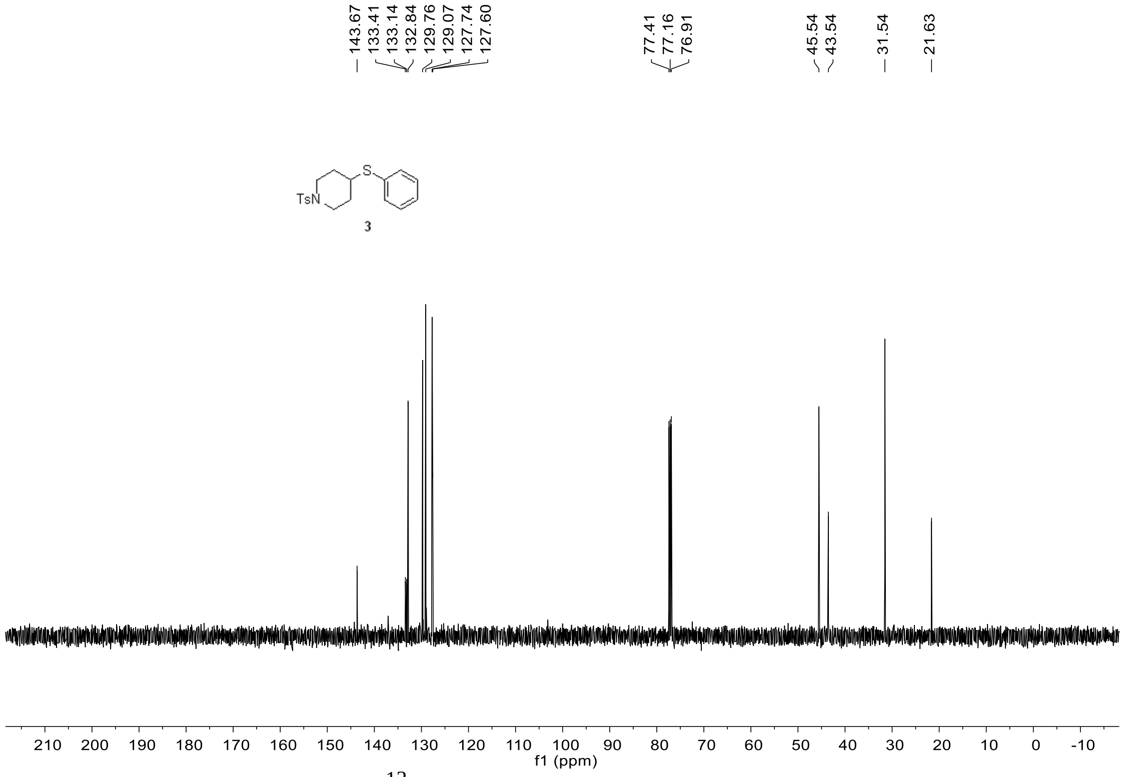

Supplementary Figure 44. ^{13}C NMR Spectra of compound **3**.

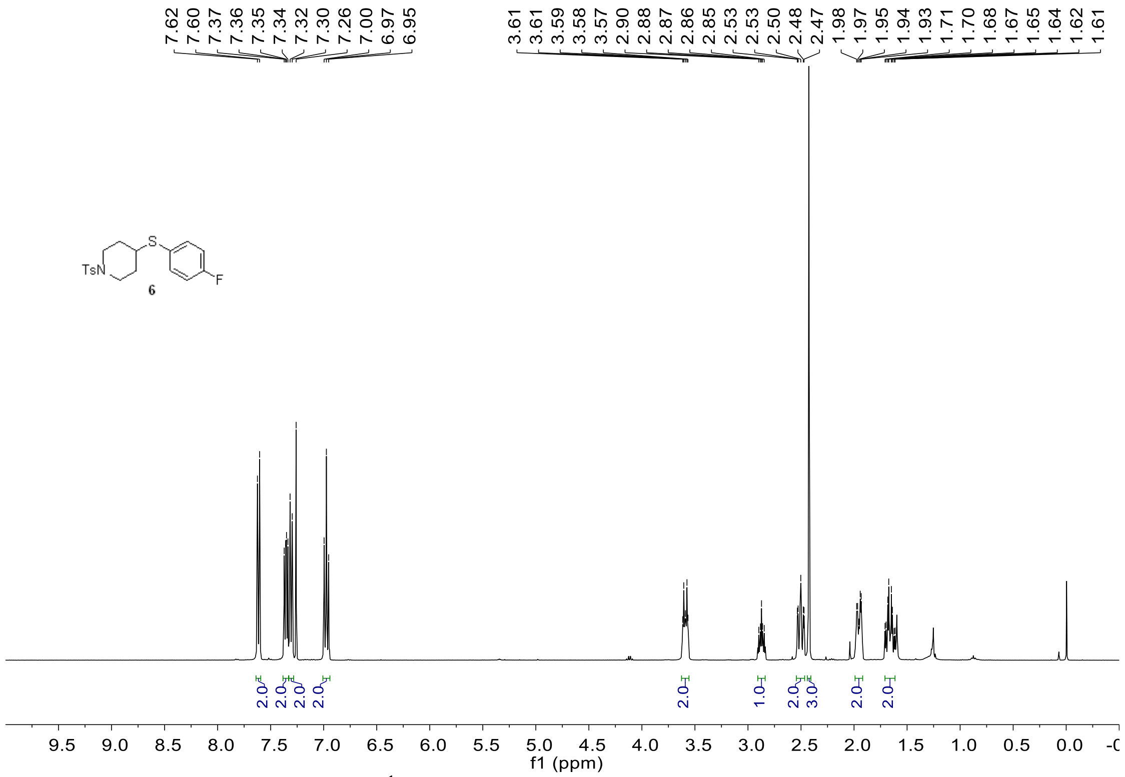

Supplementary Figure 45. ^{1}H NMR Spectra of compound **6**.

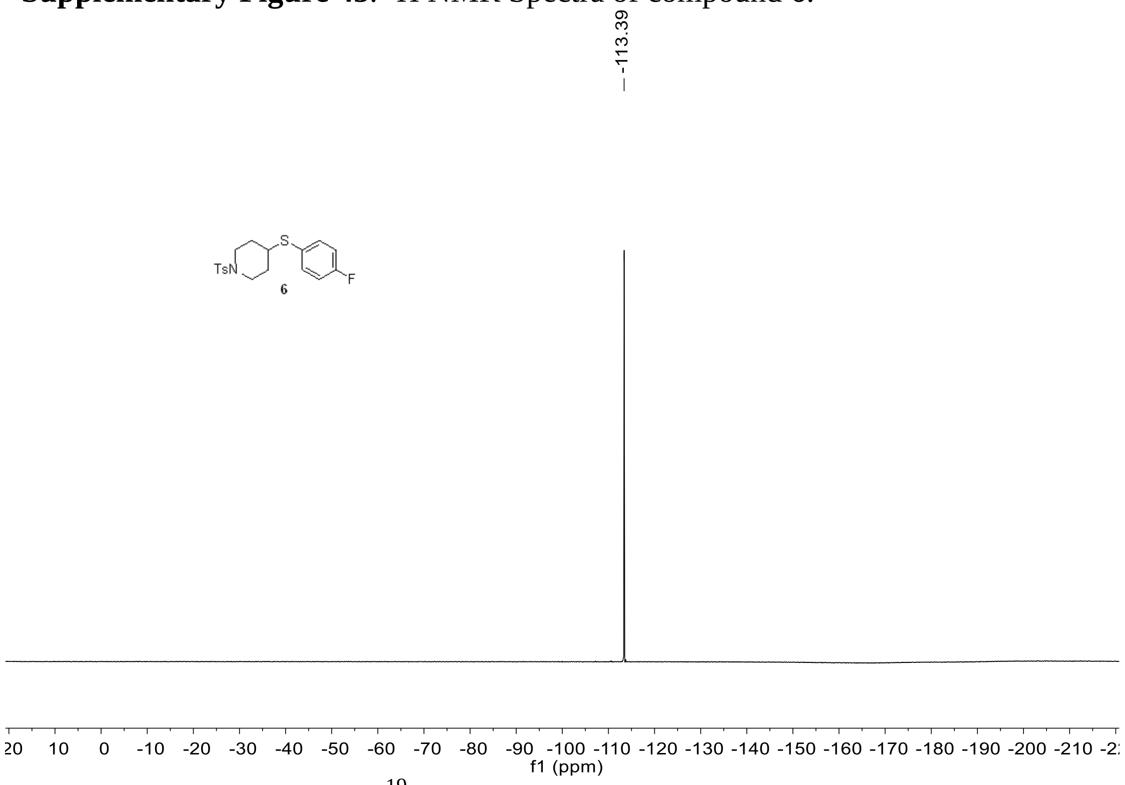

Supplementary Figure 46. ^{19}F NMR Spectra of compound **6**.

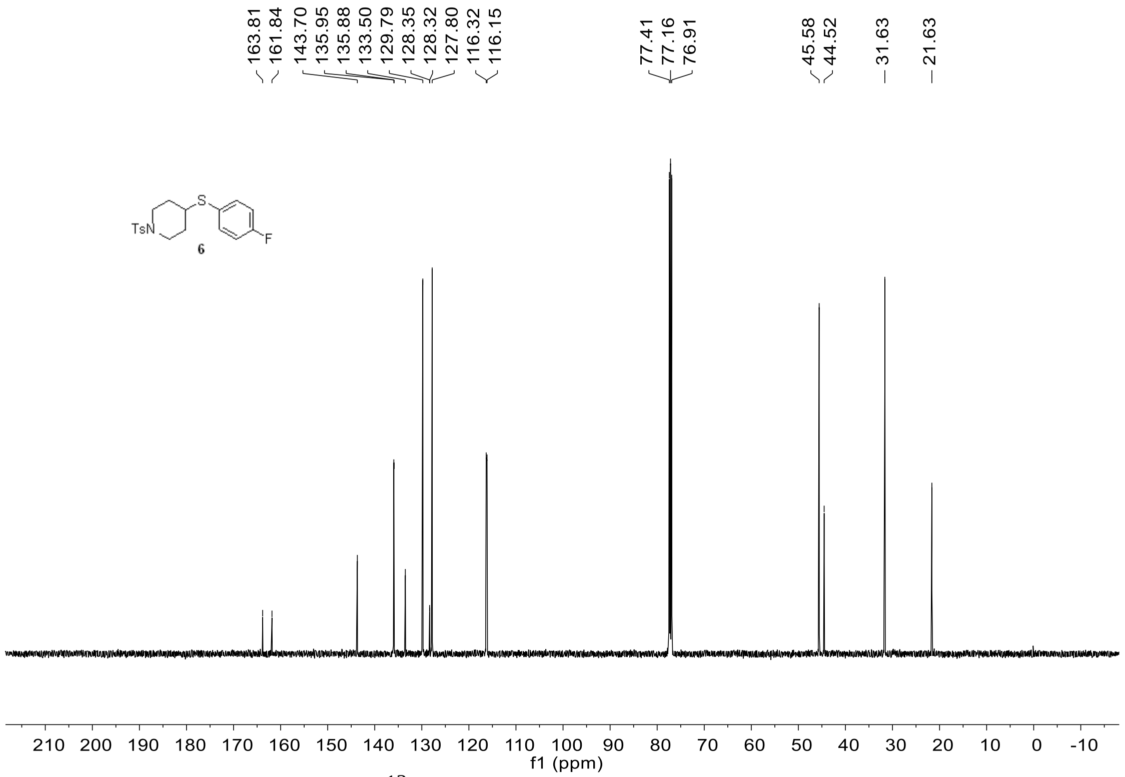

Supplementary Figure 47. ^{13}C NMR Spectra of compound **6**.

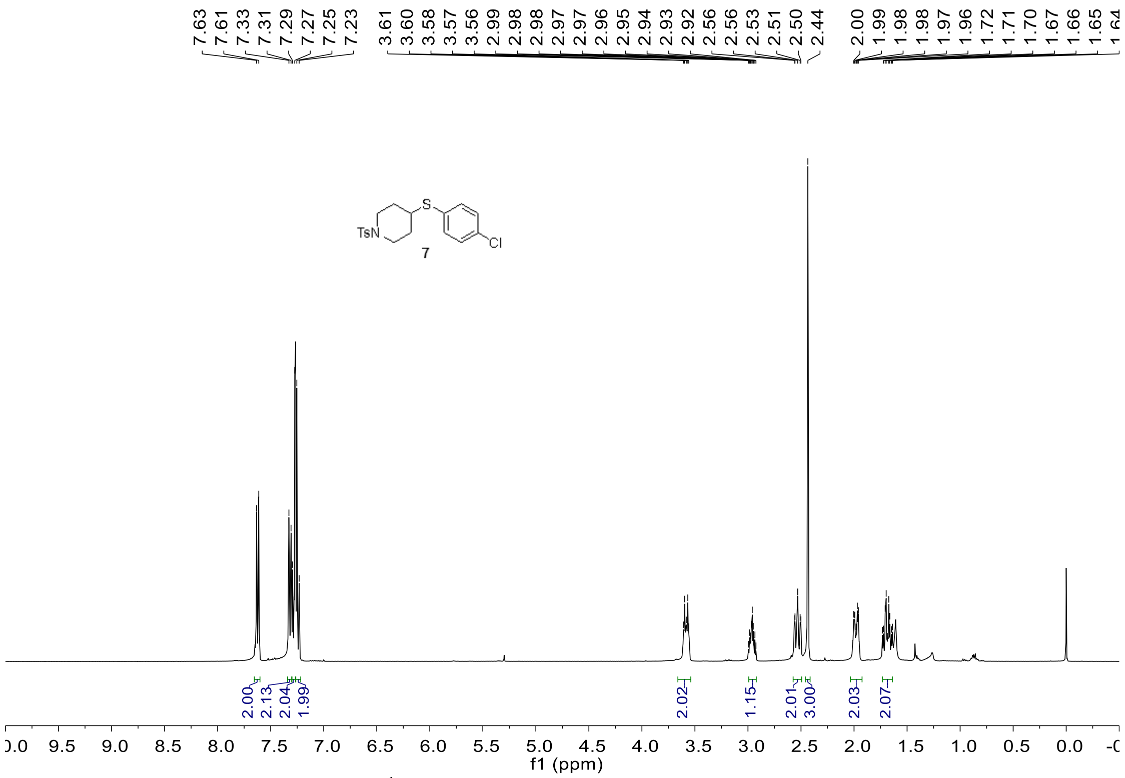

Supplementary Figure 48. ^{1}H NMR Spectra of compound **7**.

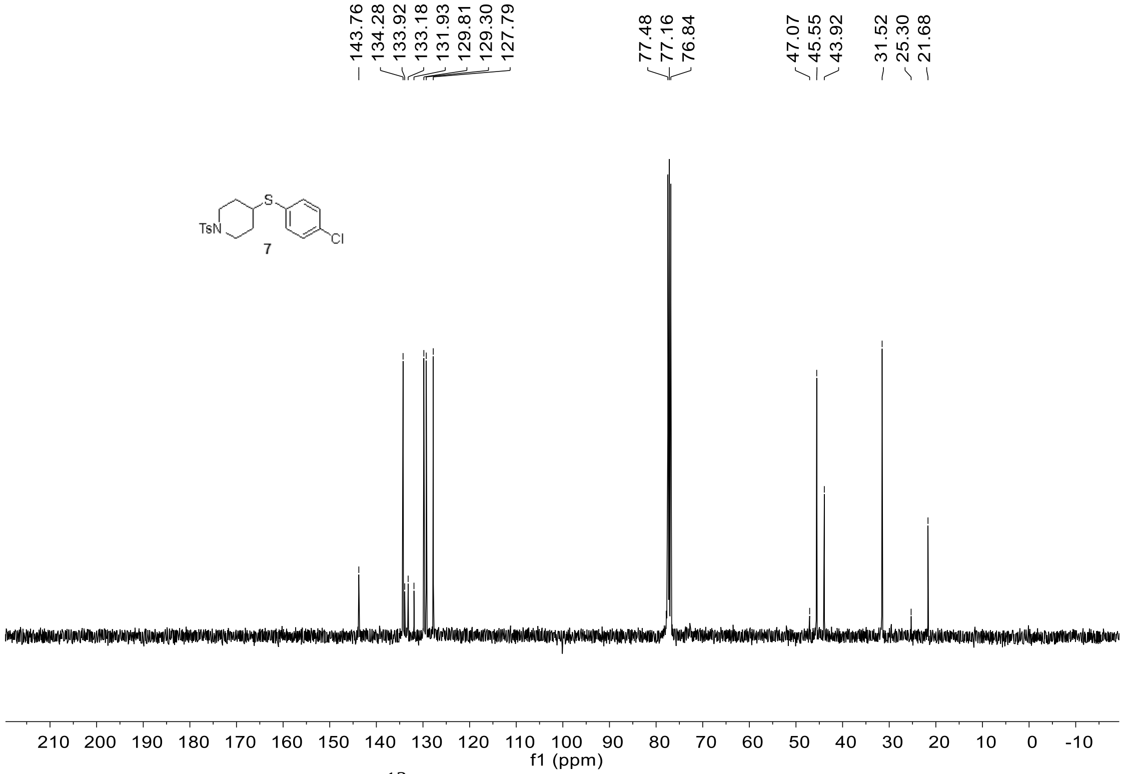

Supplementary Figure 49. ^{13}C NMR Spectra of compound **7**.

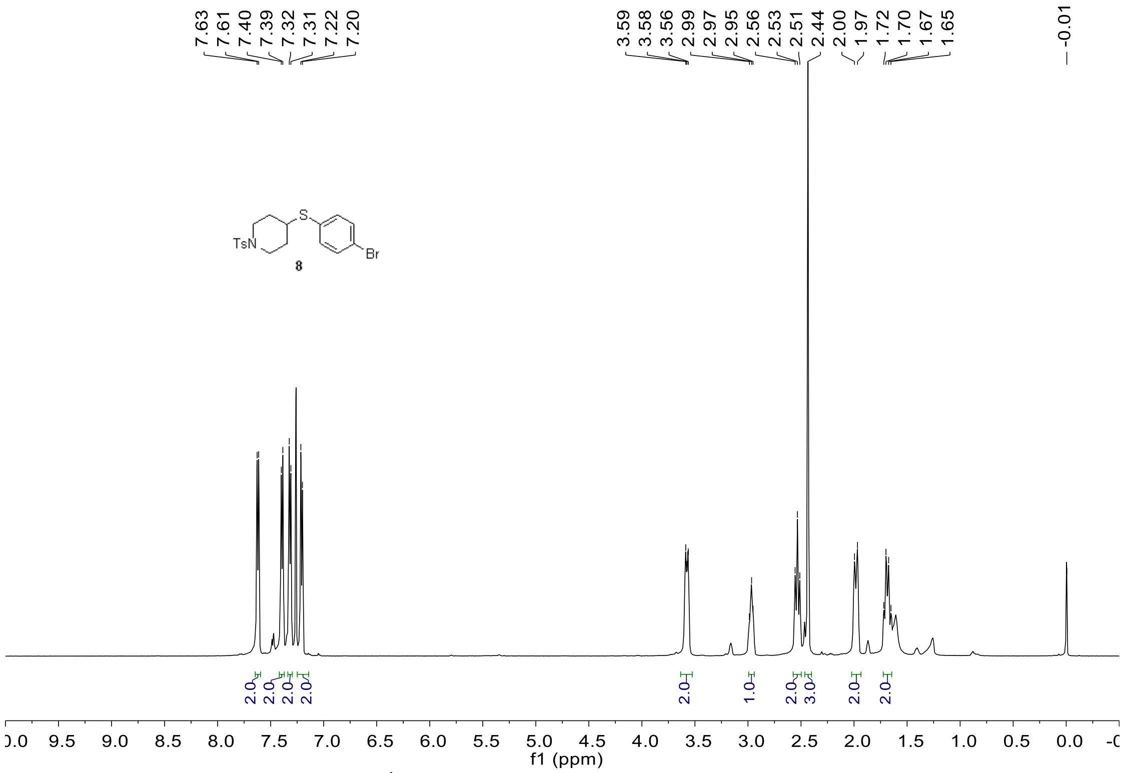

Supplementary Figure 50. ^{1}H NMR Spectra of compound **8**.

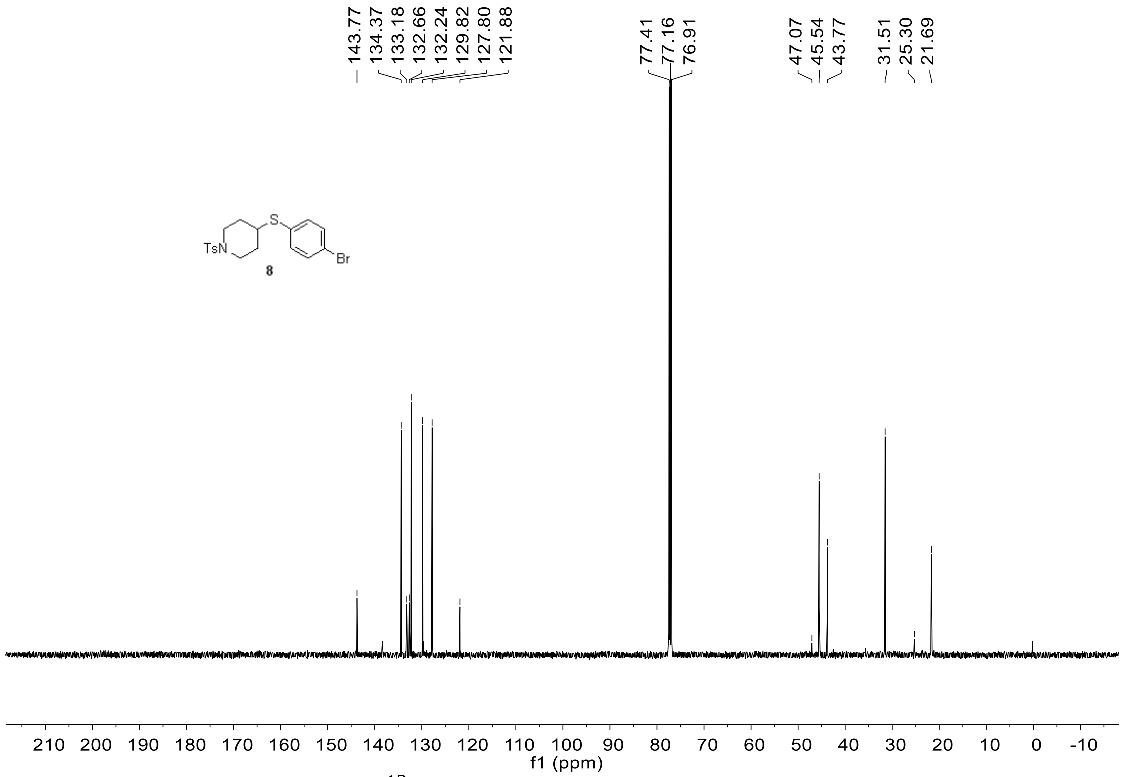

Supplementary Figure 51. ^{13}C NMR Spectra of compound **8**.

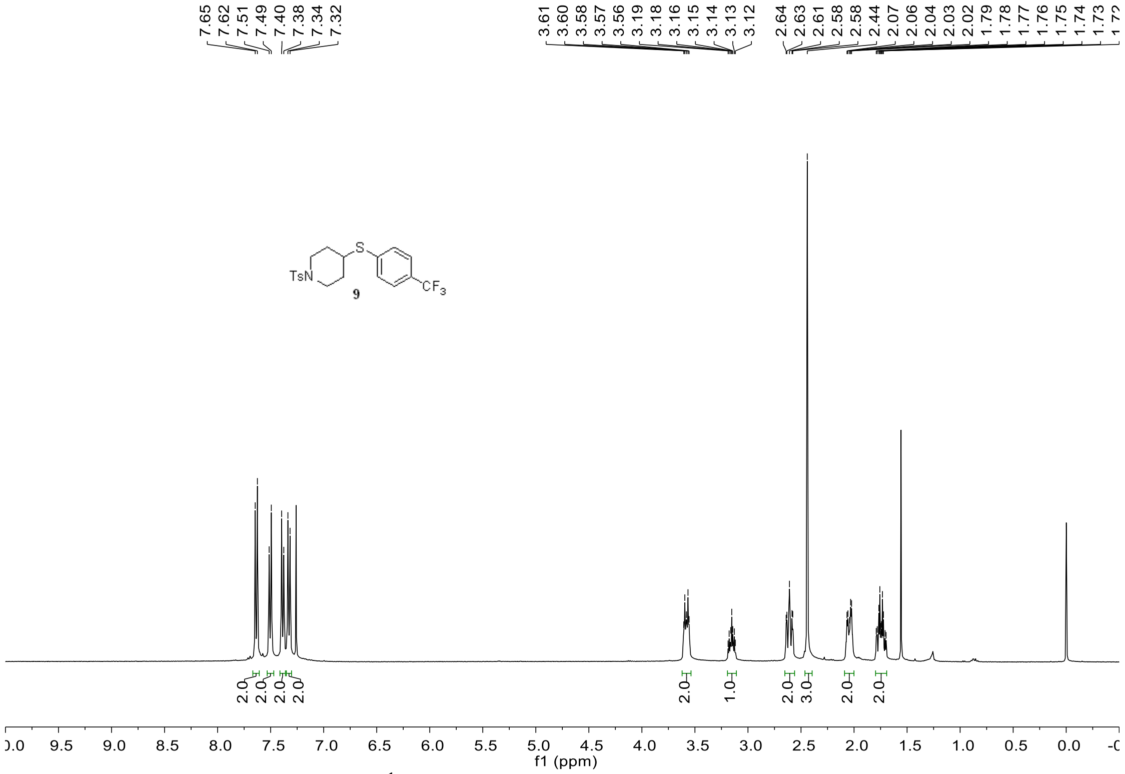

Supplementary Figure 52. ^{1}H NMR Spectra of compound **9**.

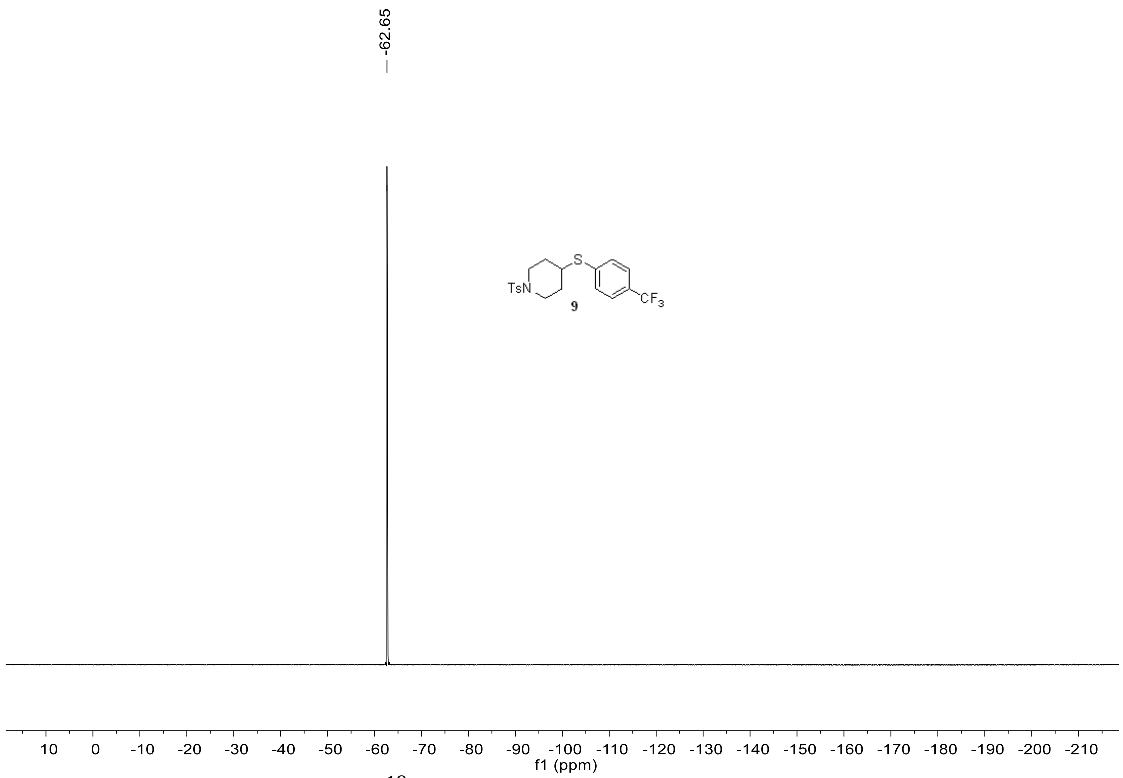

Supplementary Figure 53. ^{19}F NMR Spectra of compound **9**.

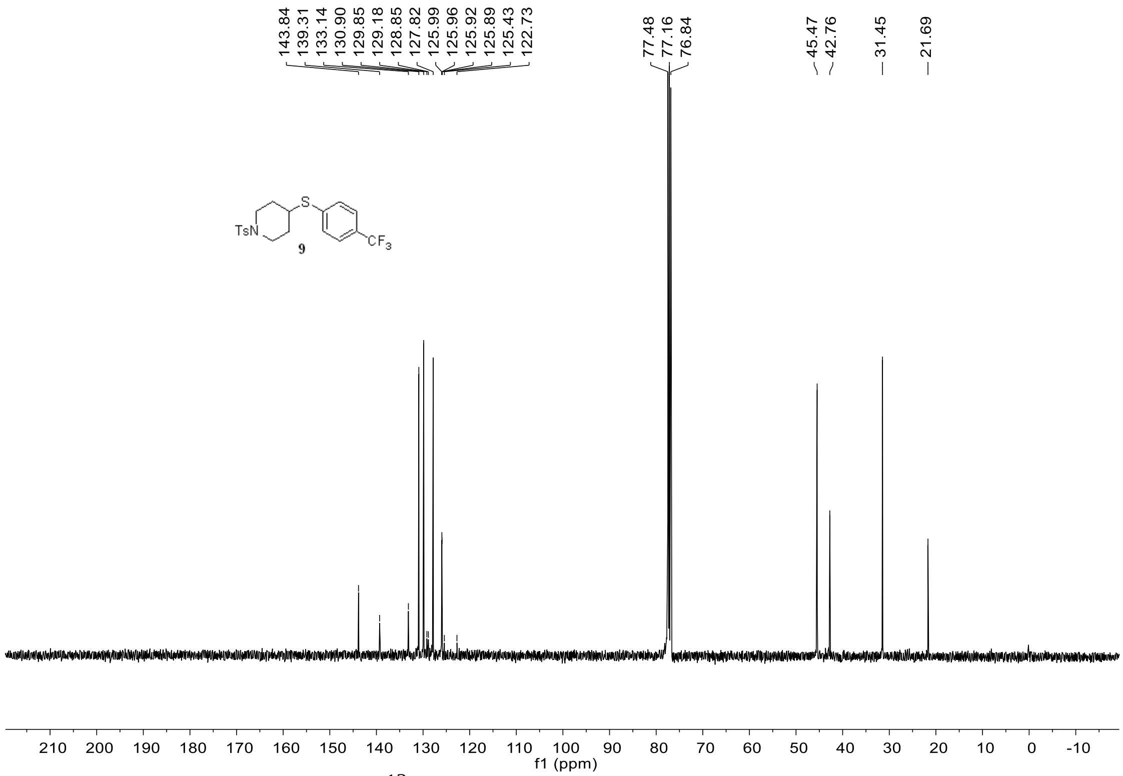

Supplementary Figure 54. ^{13}C NMR Spectra of compound **9**.

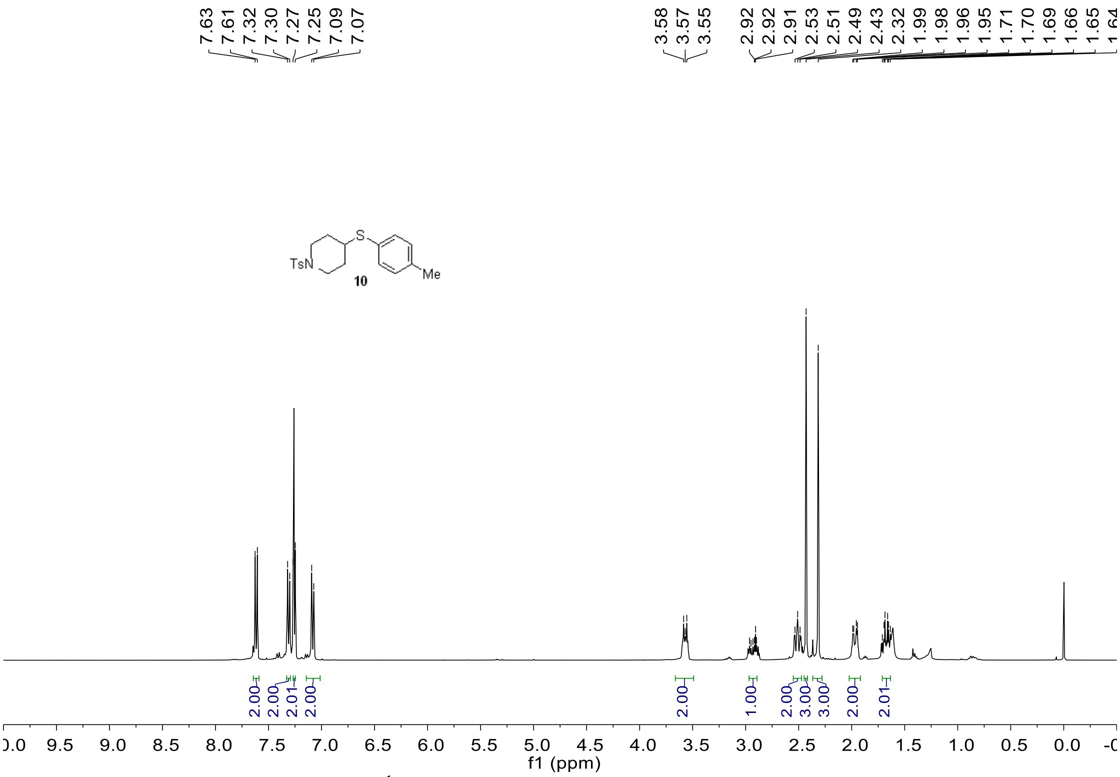

Supplementary Figure 55. ^{1}H NMR Spectra of compound **10**.

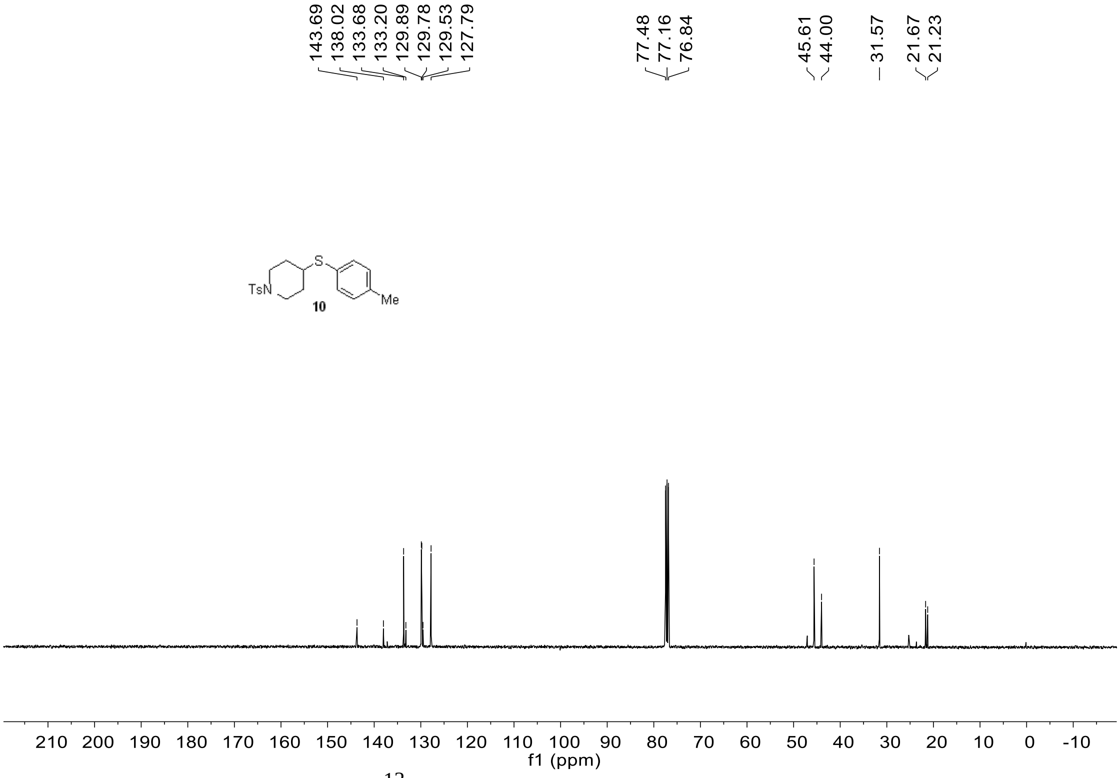

Supplementary Figure 56. ^{13}C NMR Spectra of compound **10**.

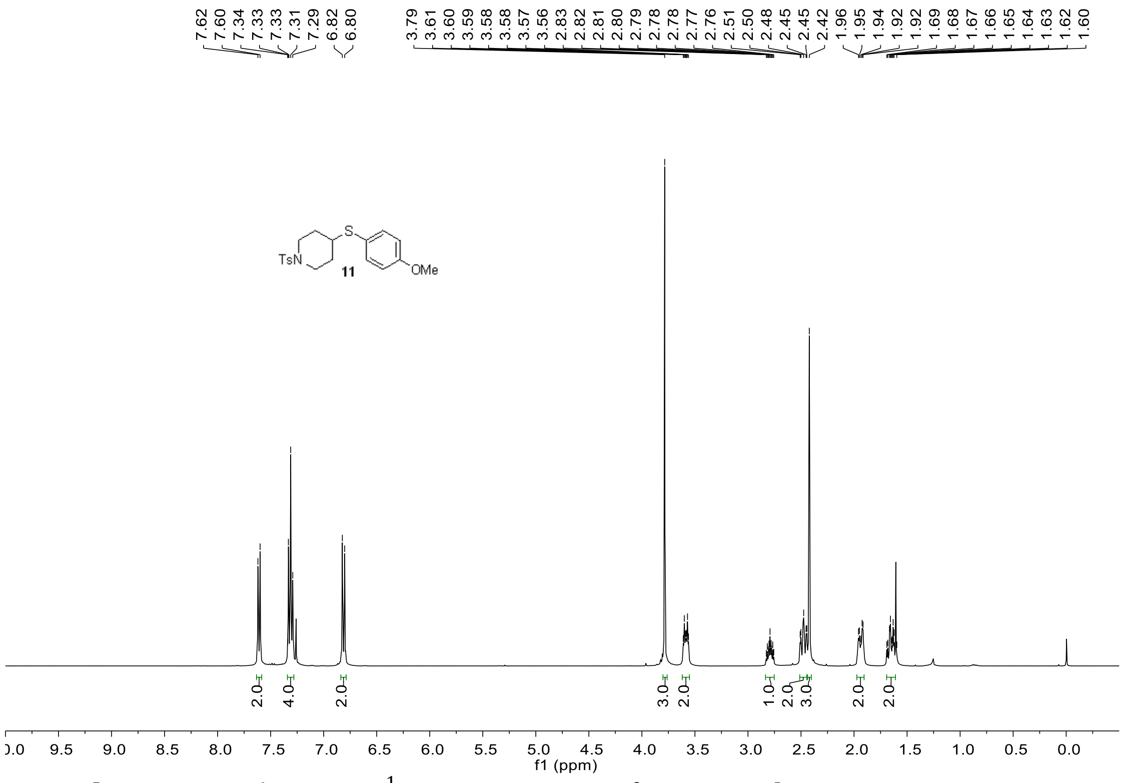

Supplementary Figure 57. ^{1}H NMR Spectra of compound **11**.

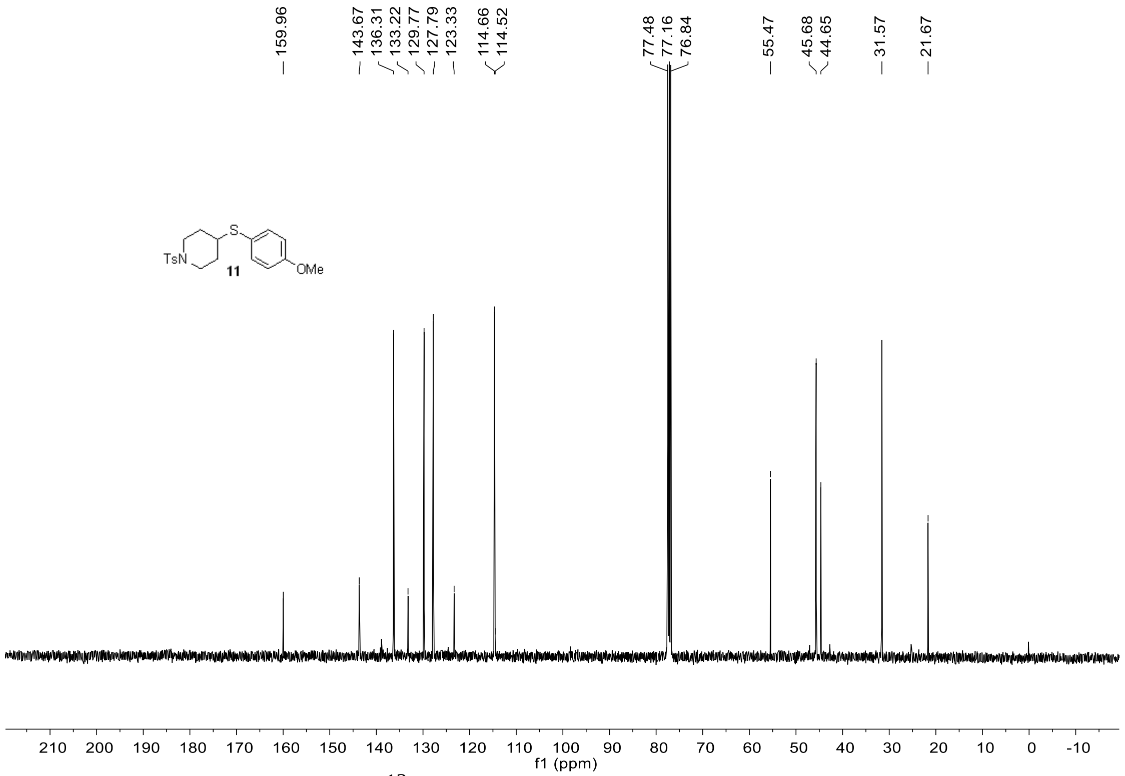

Supplementary Figure 58. ^{13}C NMR Spectra of compound **11**.

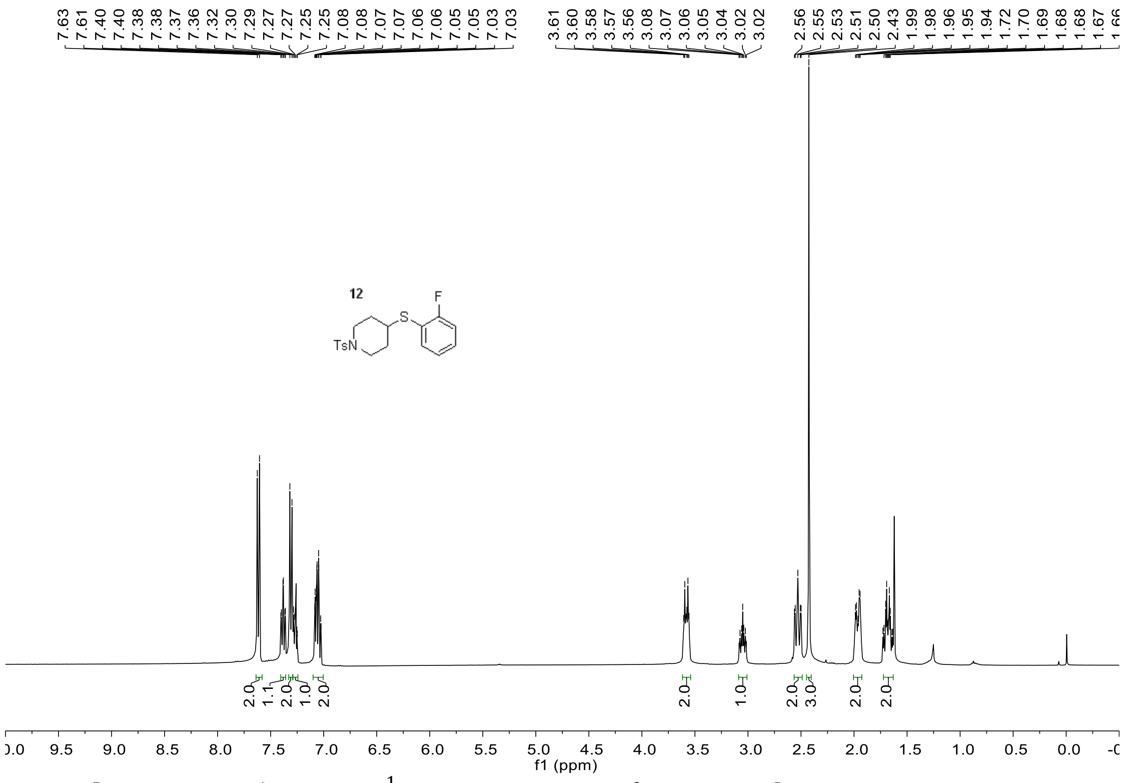

Supplementary Figure 59. ^{1}H NMR Spectra of compound **12**.

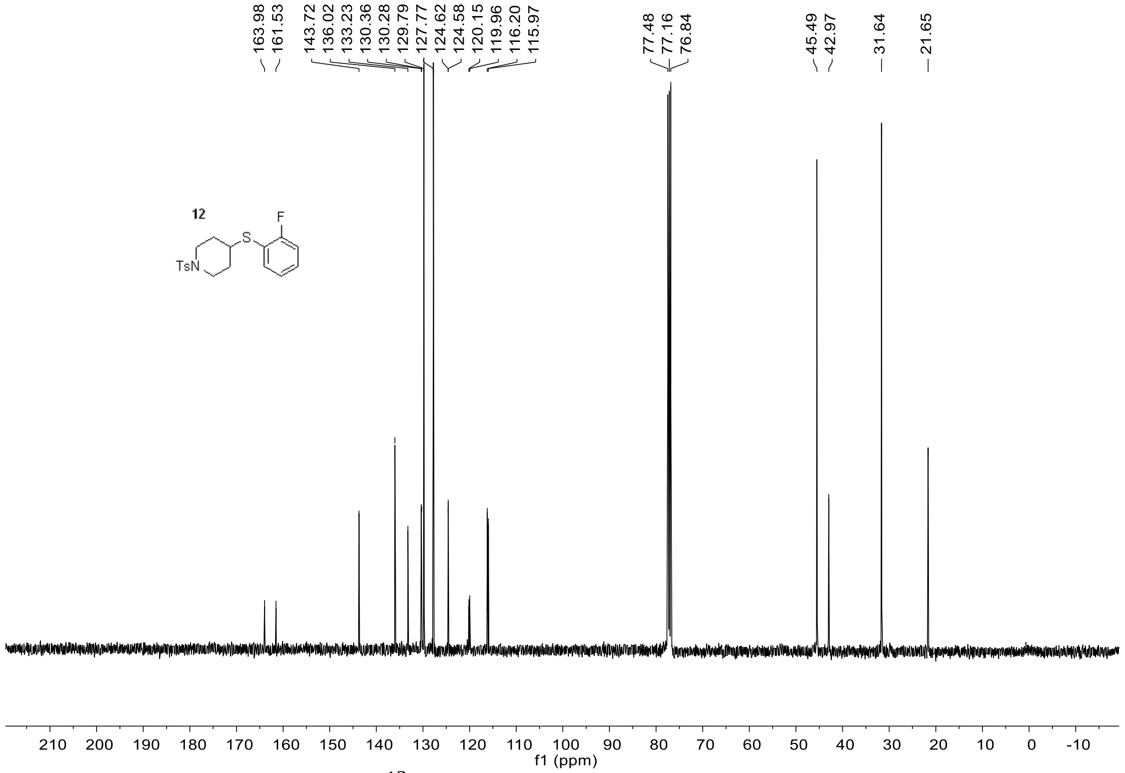

Supplementary Figure 60. ^{13}C NMR Spectra of compound **12**.

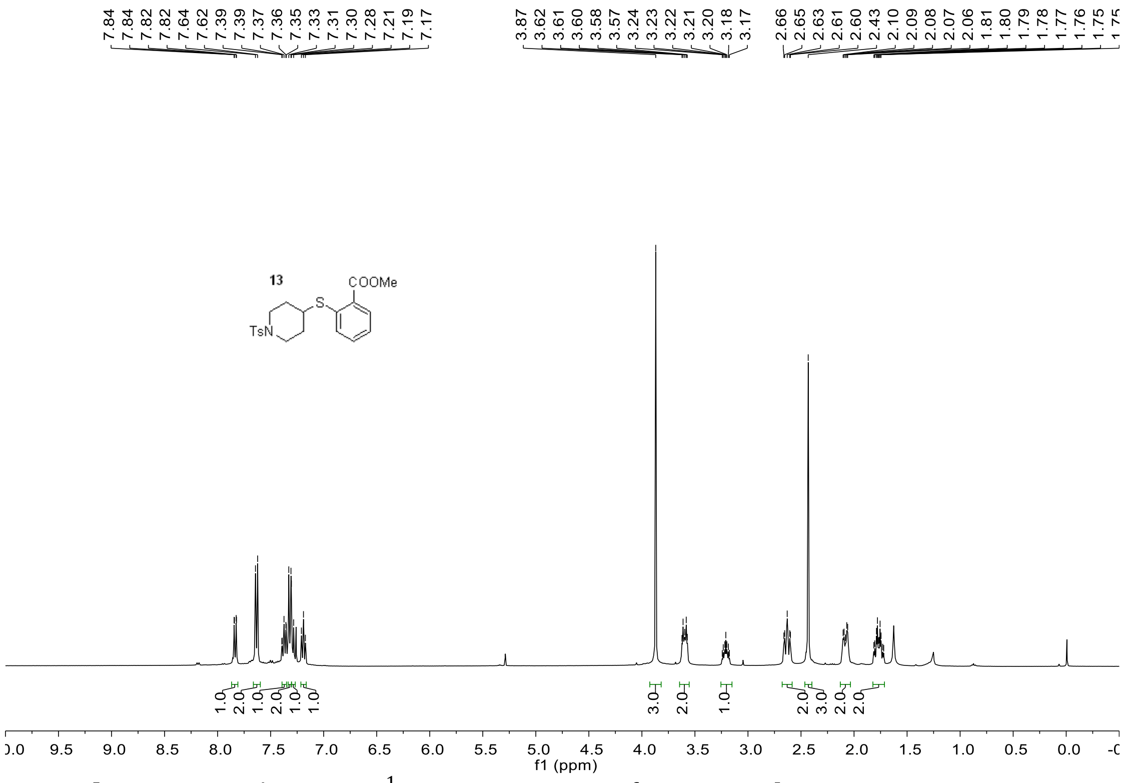

Supplementary Figure 61. ^{1}H NMR Spectra of compound **13**.

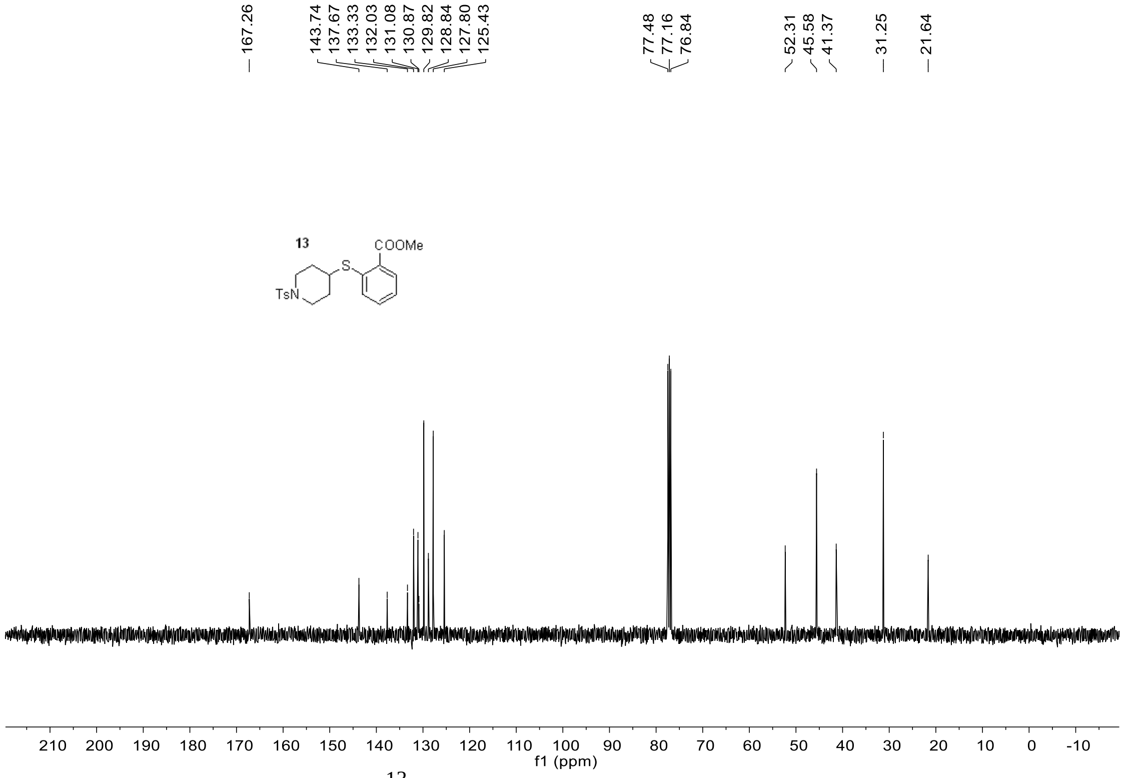

Supplementary Figure 62. ^{13}C NMR Spectra of compound **13**.

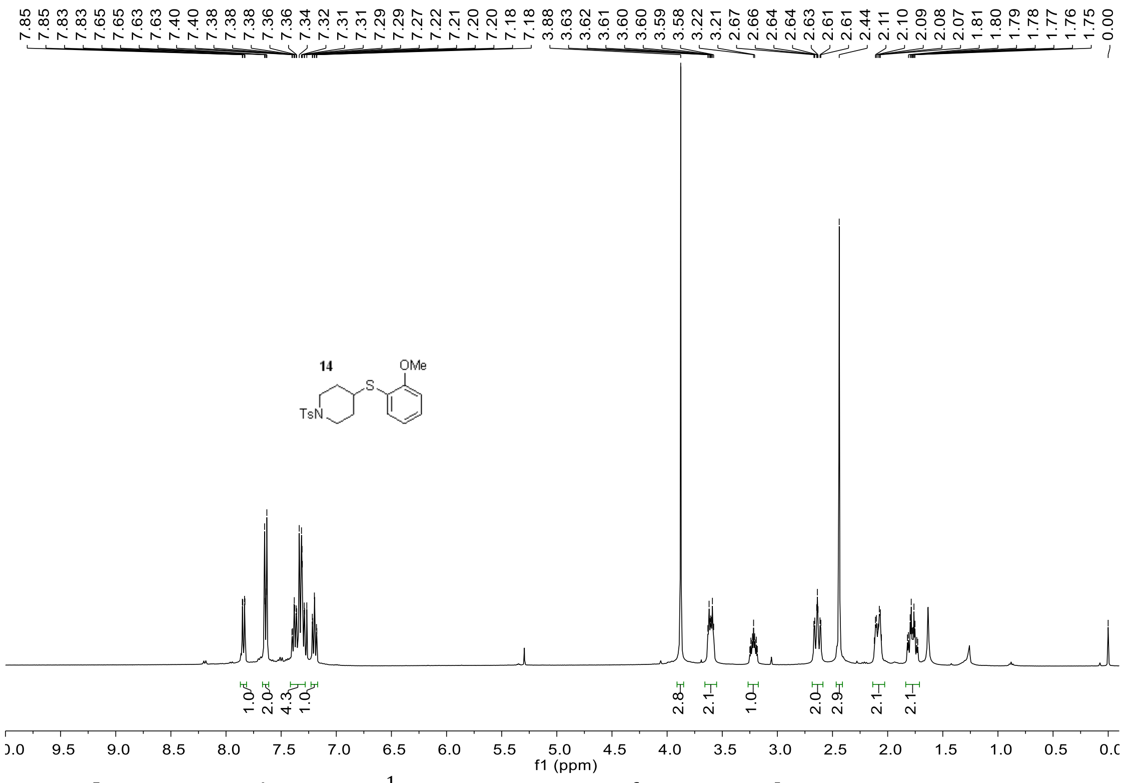

Supplementary Figure 63. ^{1}H NMR Spectra of compound **14**.

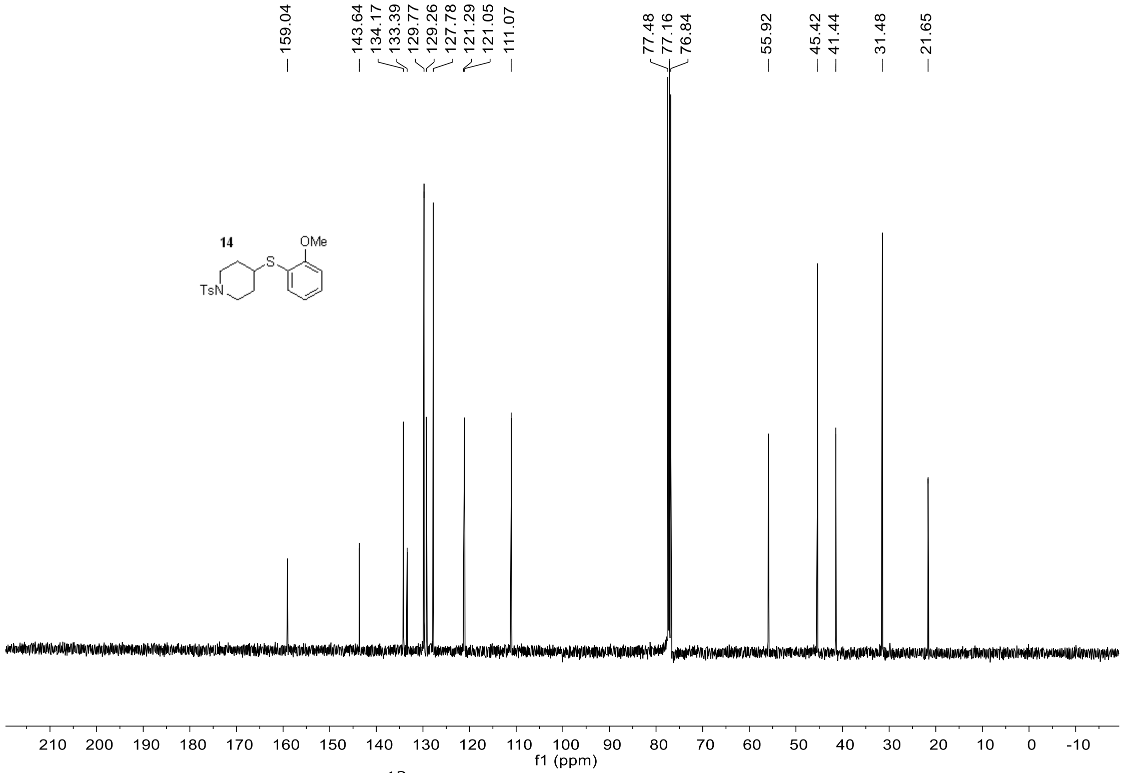

Supplementary Figure 64. ^{13}C NMR Spectra of compound **14**.

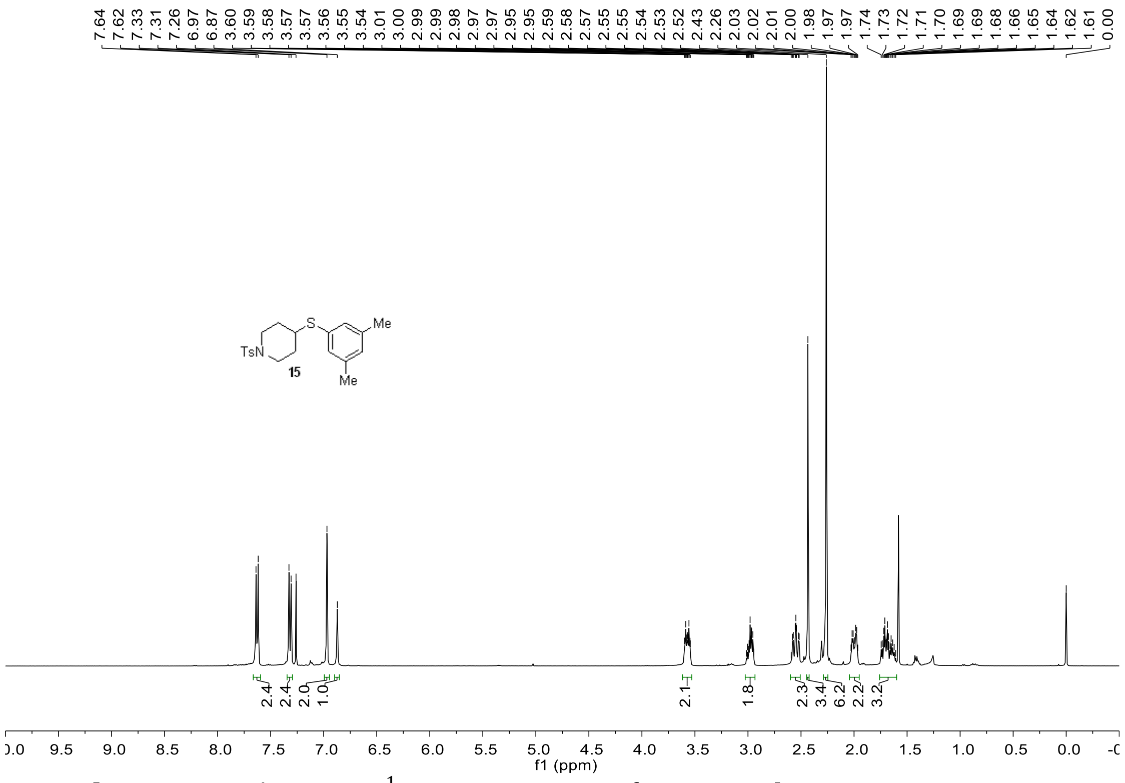

Supplementary Figure 65. ^{1}H NMR Spectra of compound **15**.

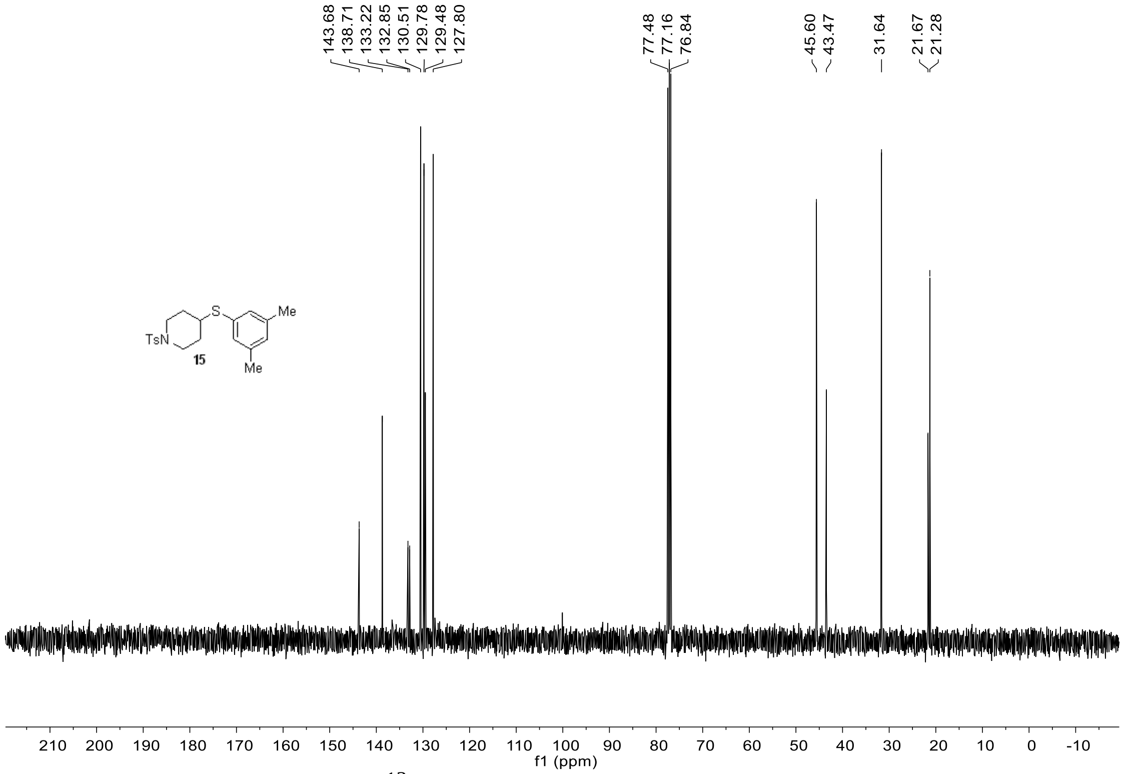

Supplementary Figure 66. ^{13}C NMR Spectra of compound **15**.

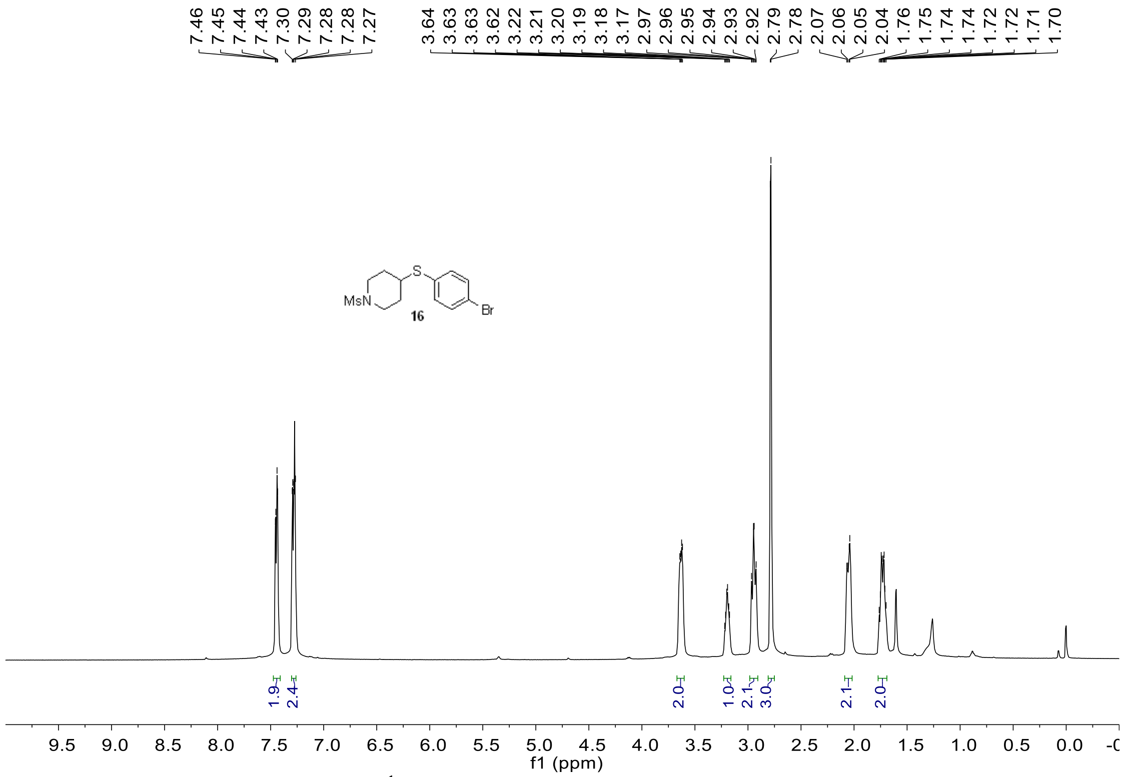

Supplementary Figure 67. ^{1}H NMR Spectra of compound **16**.

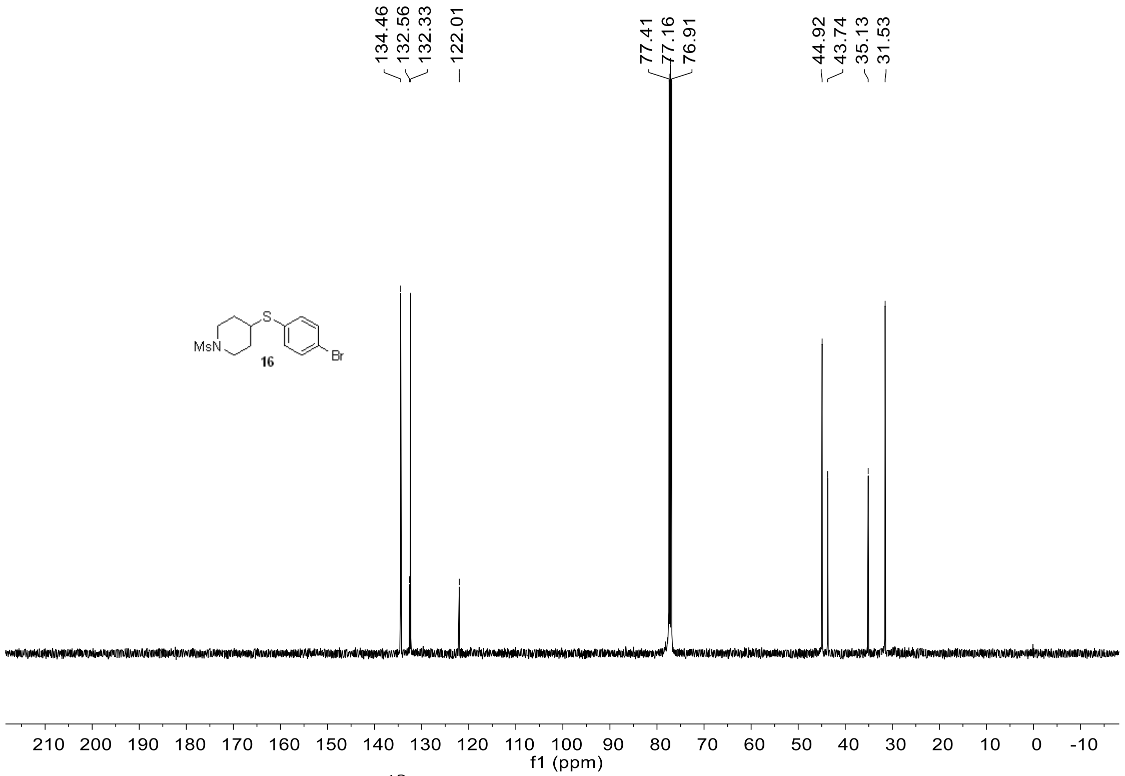

Supplementary Figure 68. ^{13}C NMR Spectra of compound **16**.

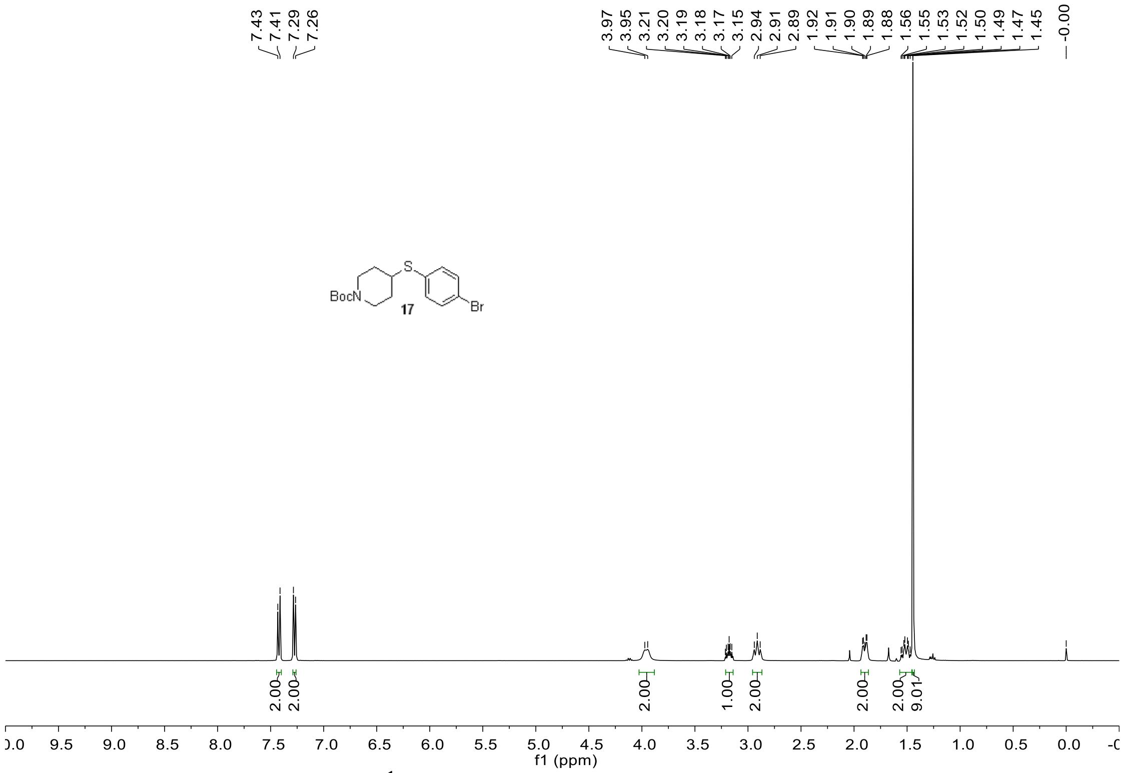

Supplementary Figure 69. ^{1}H NMR Spectra of compound **17**.

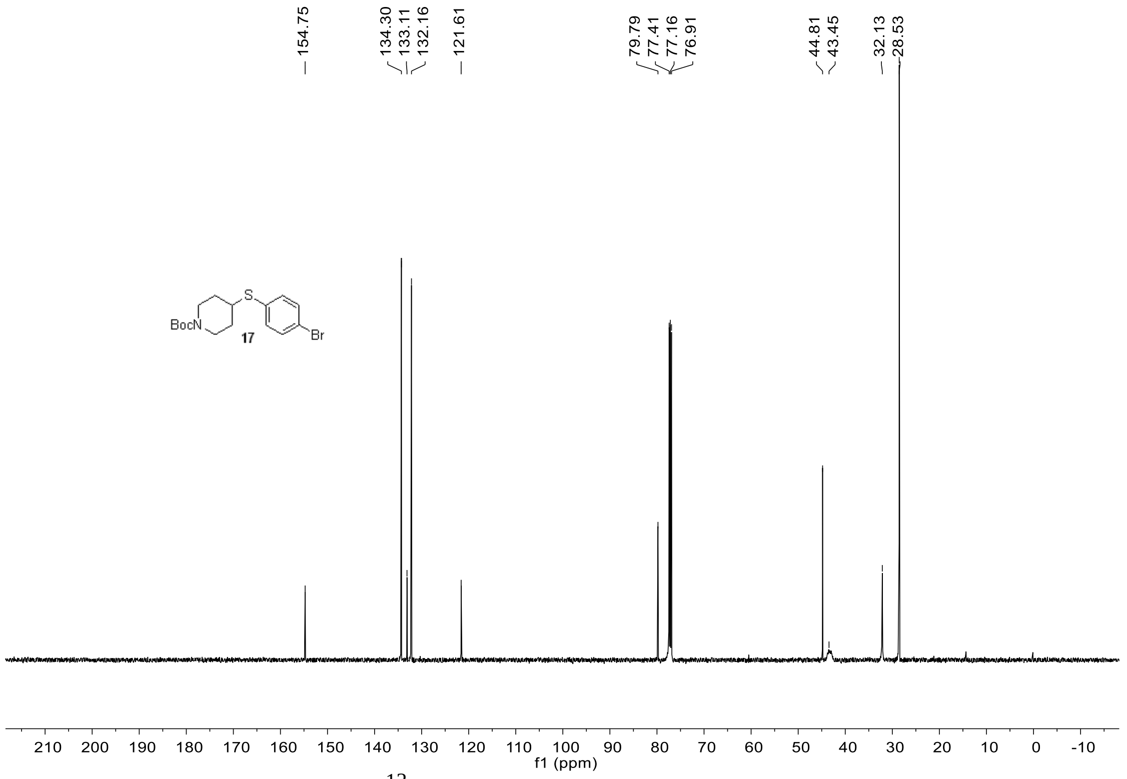

Supplementary Figure 70. ^{13}C NMR Spectra of compound **17**.

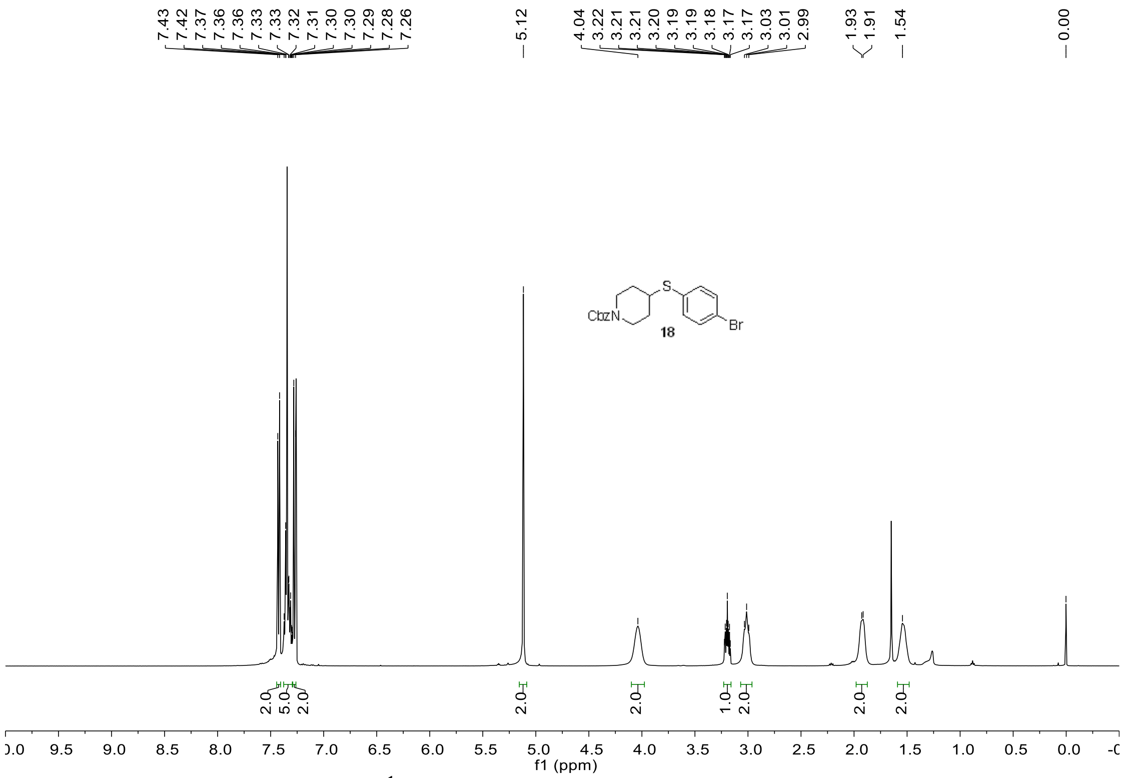

Supplementary Figure 71. ^{1}H NMR Spectra of compound **18**.

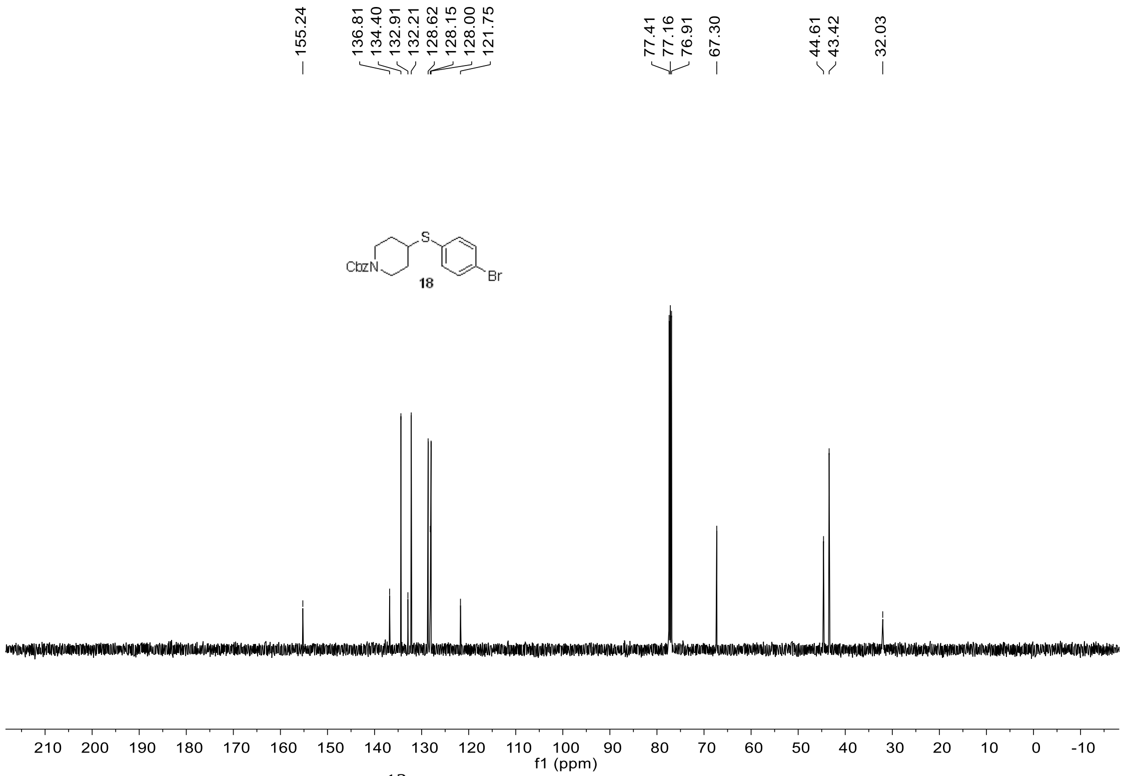

Supplementary Figure 72. ^{13}C NMR Spectra of compound **18**.

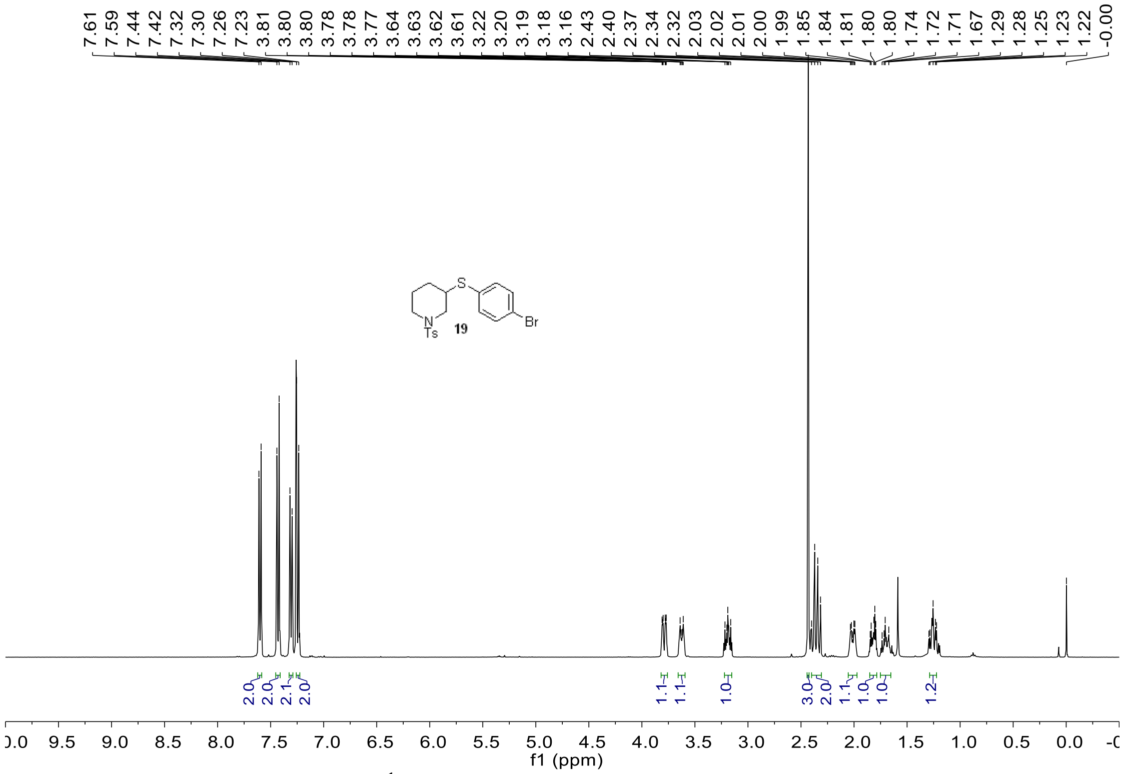

Supplementary Figure 73. ^{1}H NMR Spectra of compound **19**.

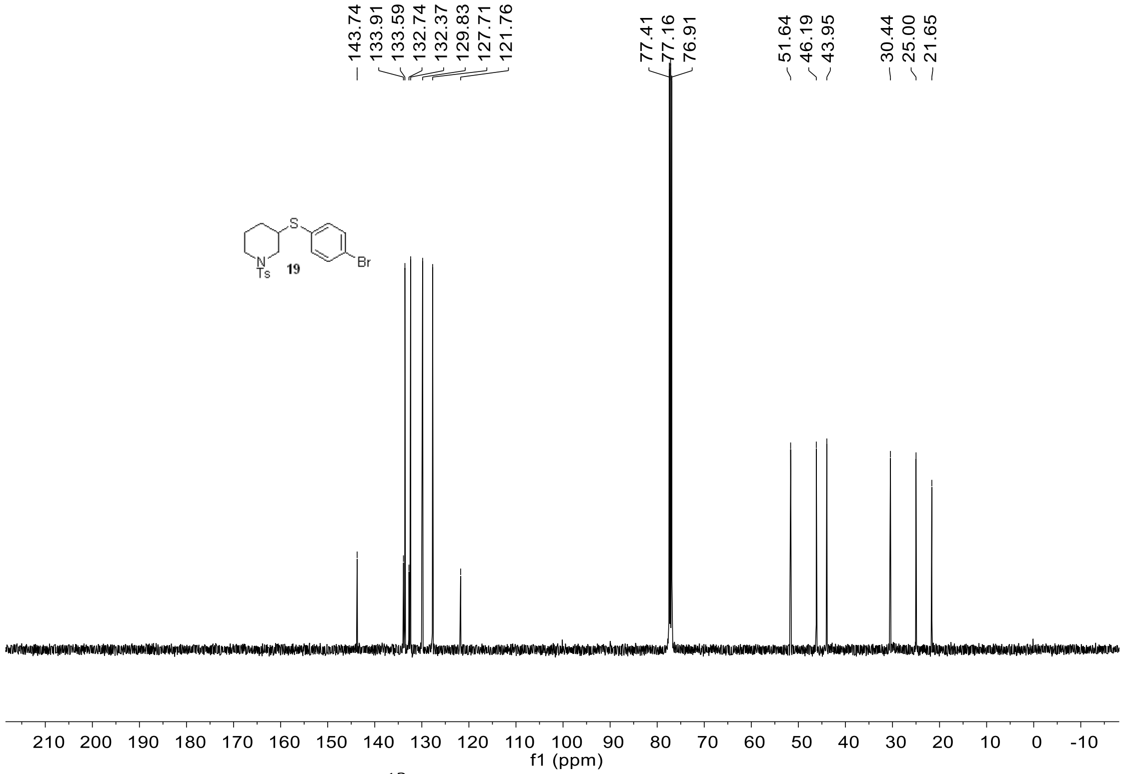

Supplementary Figure 74. ^{13}C NMR Spectra of compound **19**.

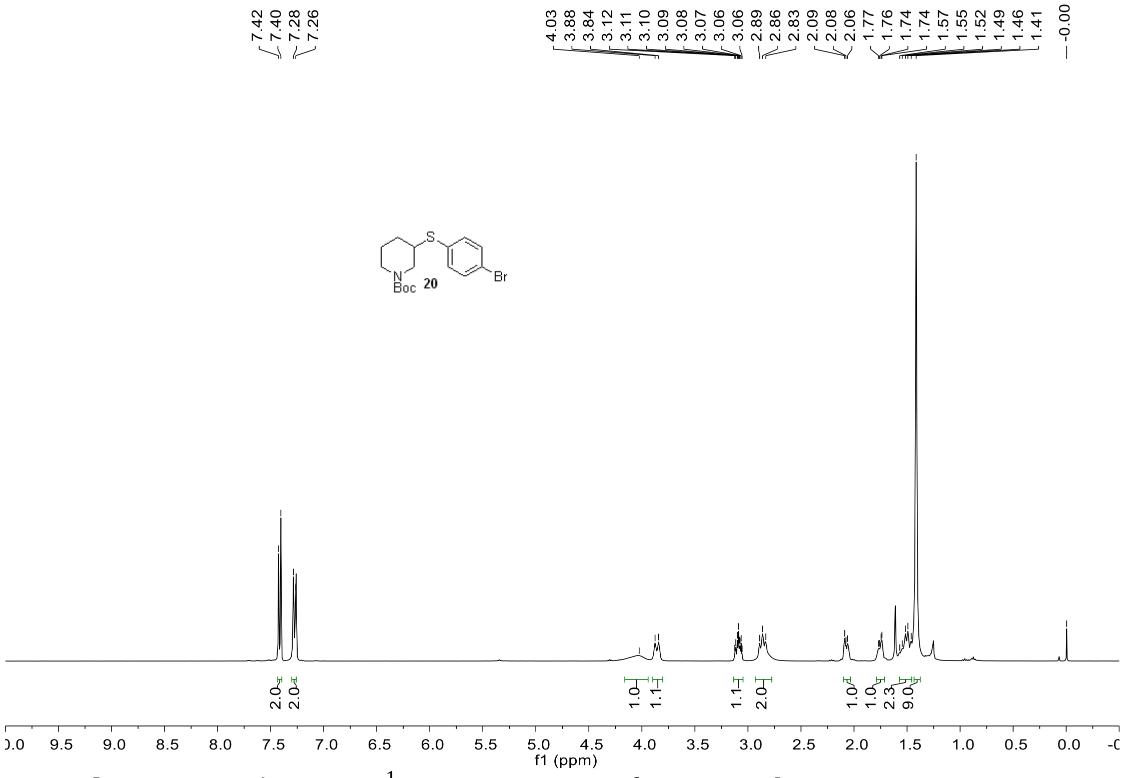

Supplementary Figure 75. ^{1}H NMR Spectra of compound **20**.

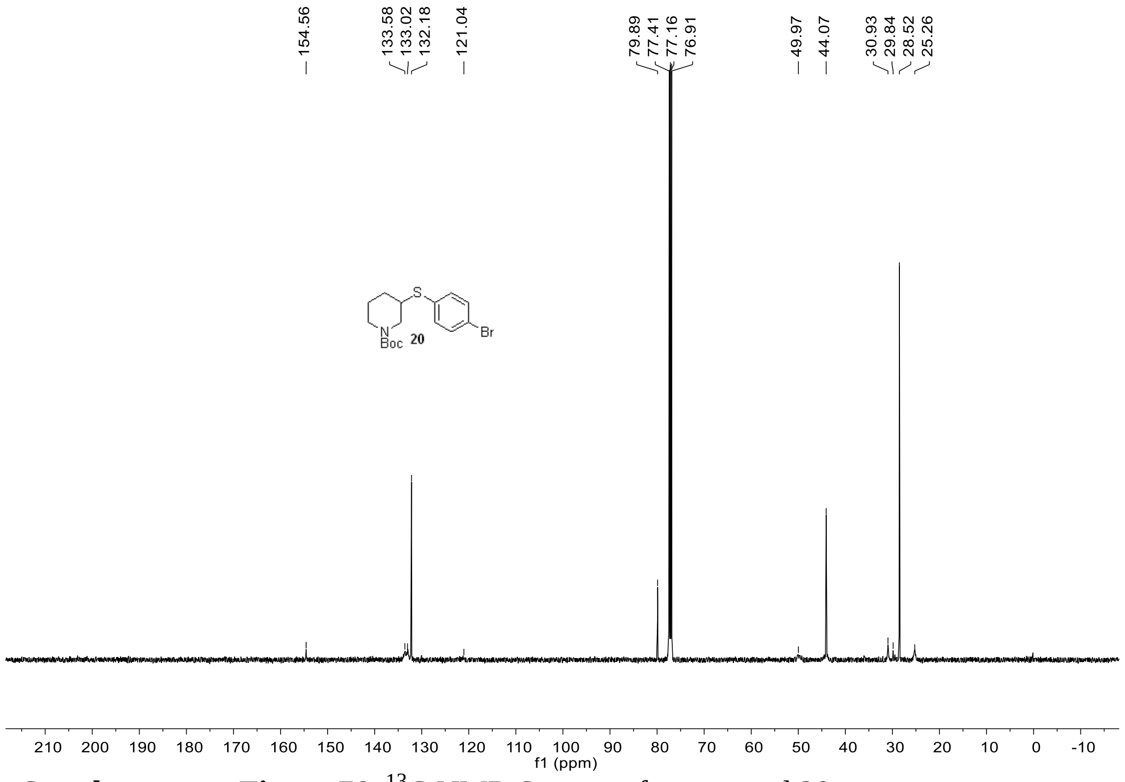

Supplementary Figure 76. ^{13}C NMR Spectra of compound **20**.

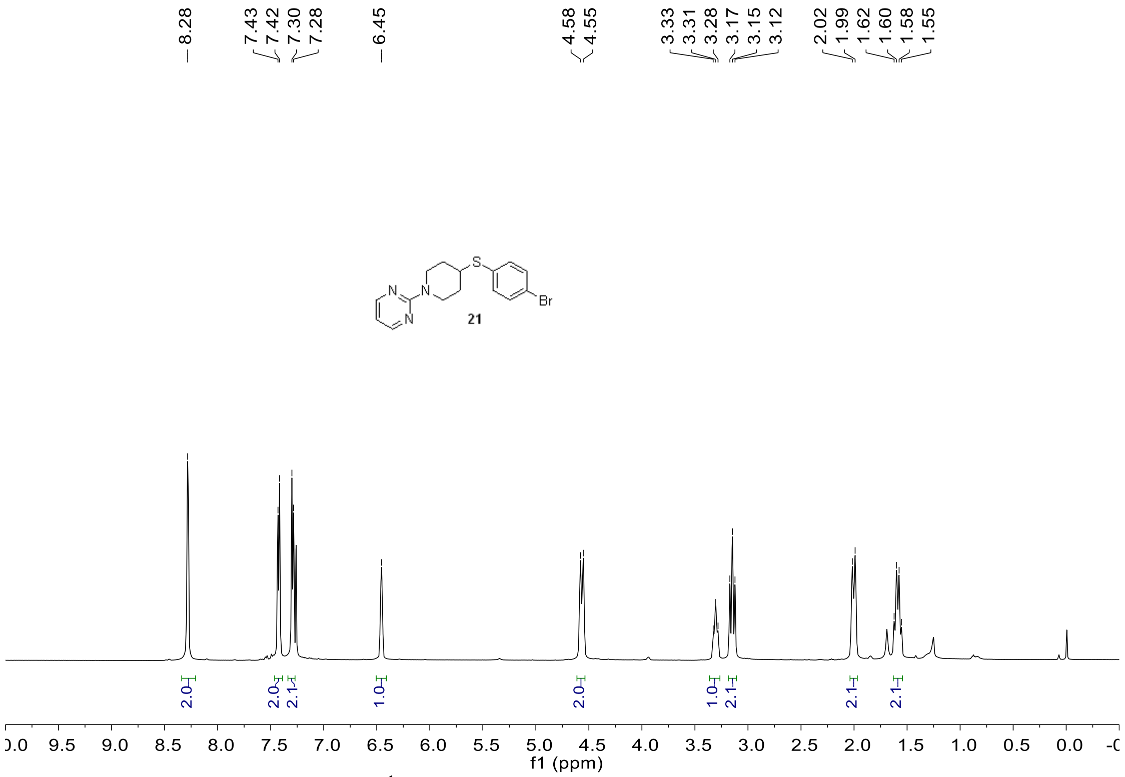

Supplementary Figure 77. ^{1}H NMR Spectra of compound **21**.

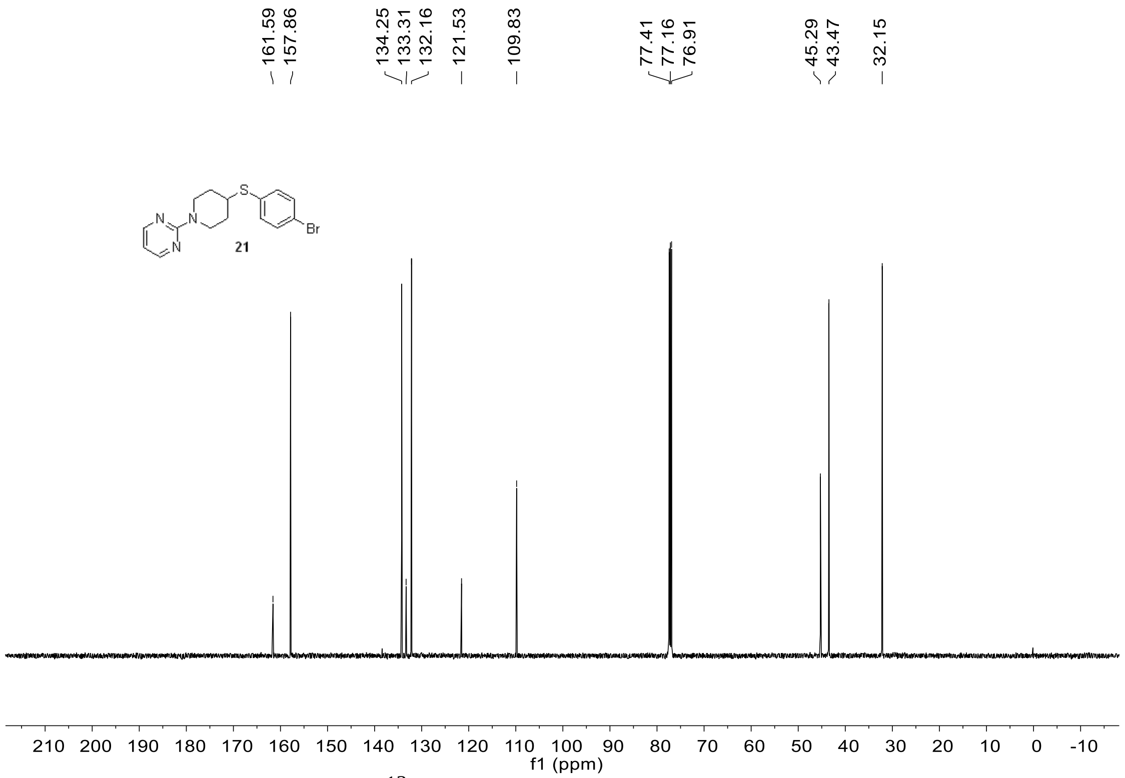

Supplementary Figure 78. ^{13}C NMR Spectra of compound **21**.

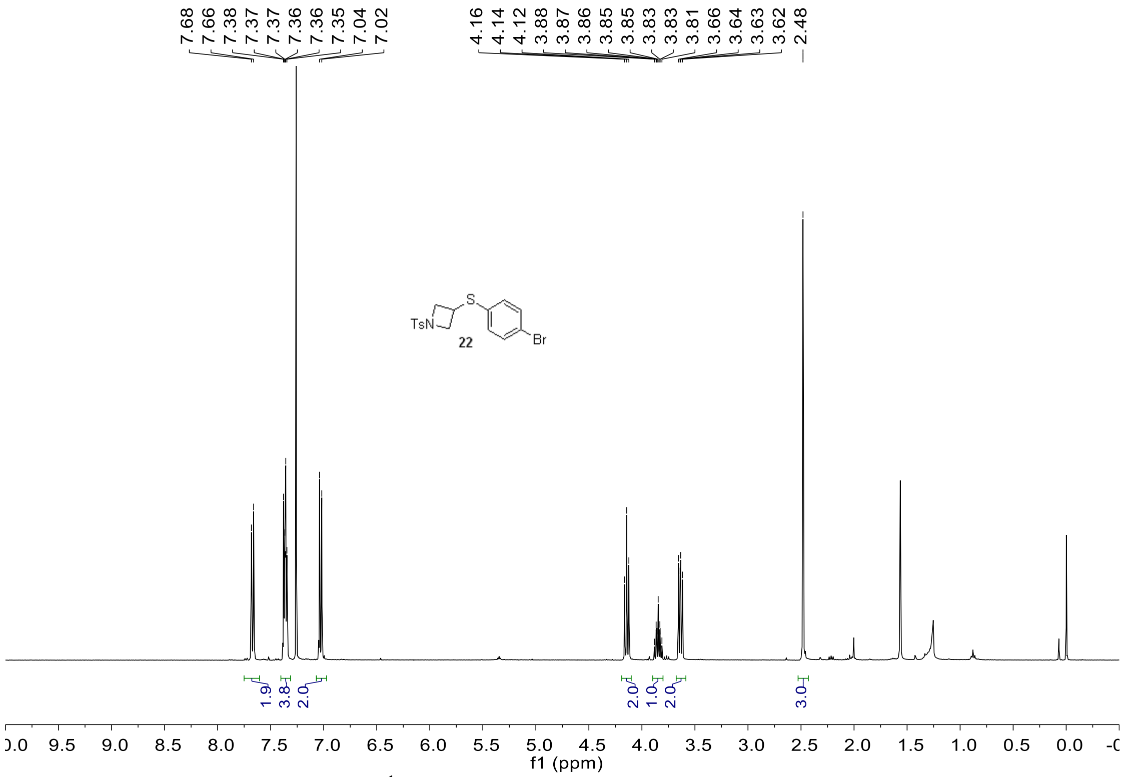

Supplementary Figure 79. ^{1}H NMR Spectra of compound **22**.

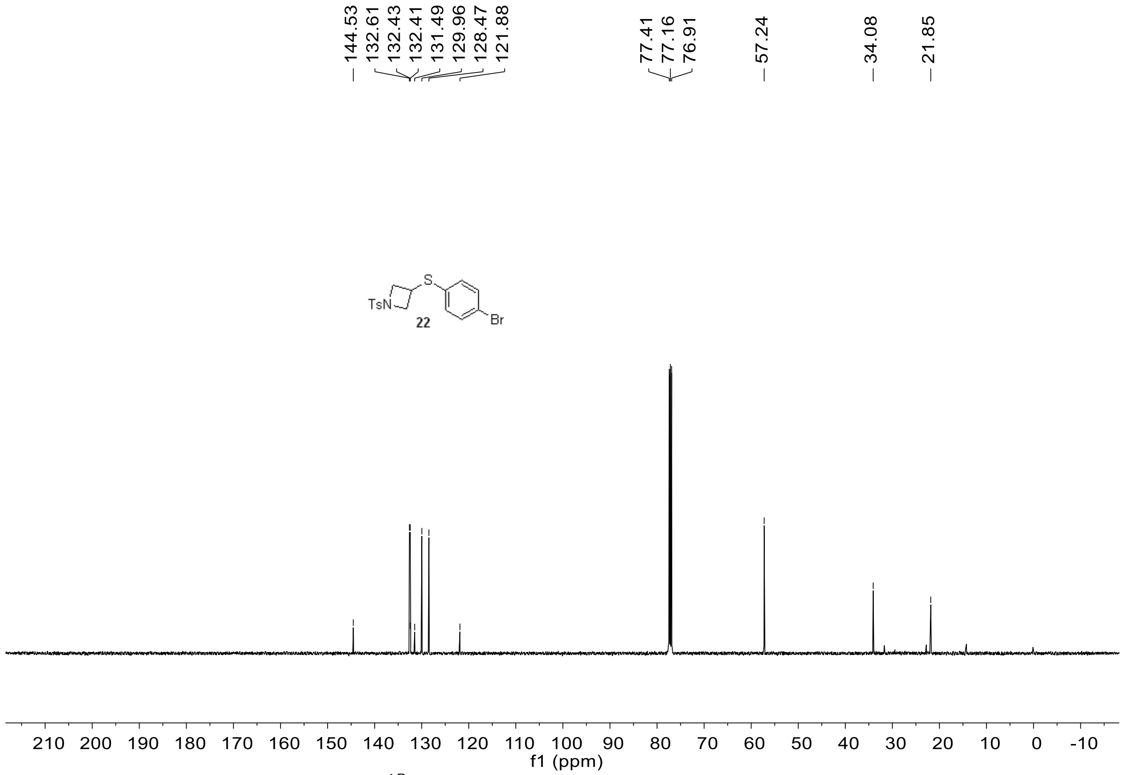

Supplementary Figure 80. ^{13}C NMR Spectra of compound **22**.

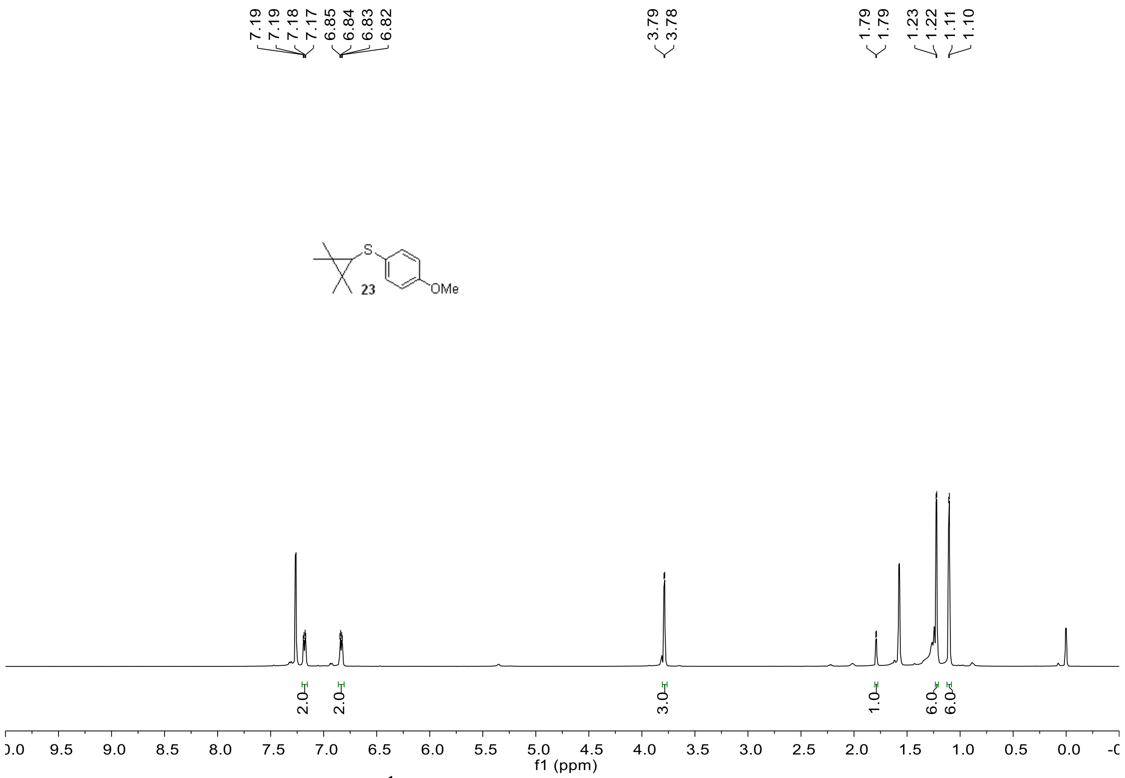

Supplementary Figure 81. ^{1}H NMR Spectra of compound **23**.

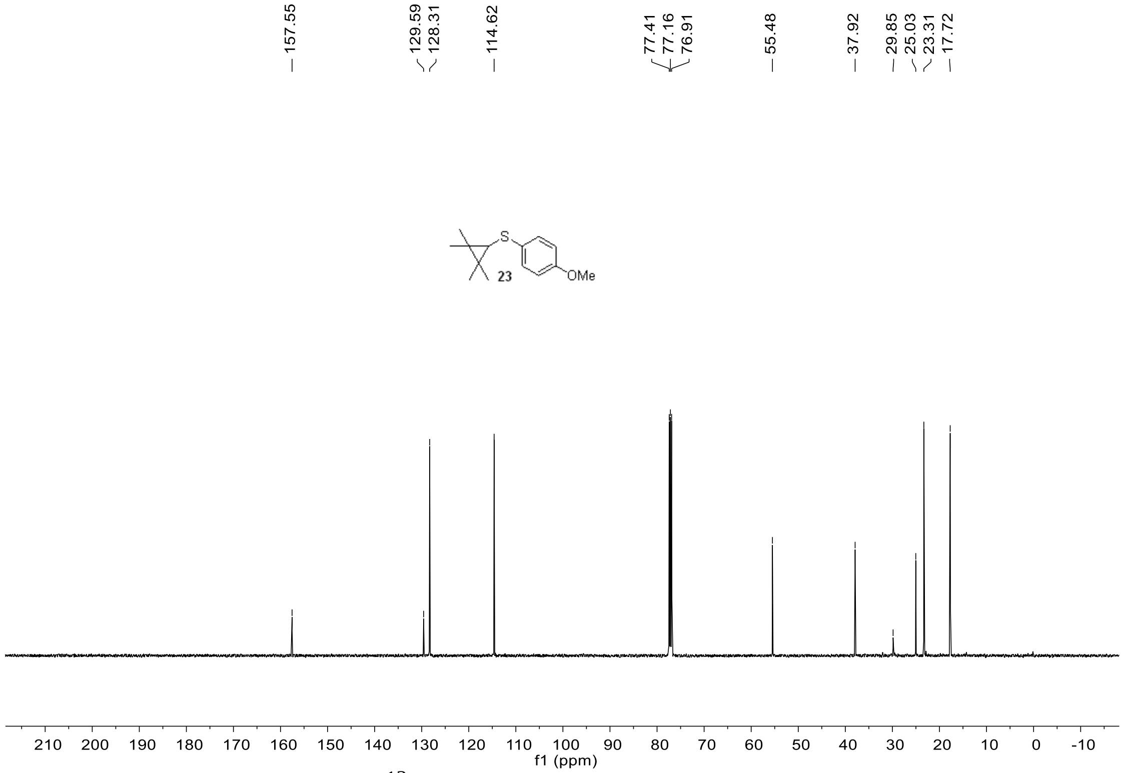

Supplementary Figure 82. ^{13}C NMR Spectra of compound **23**.

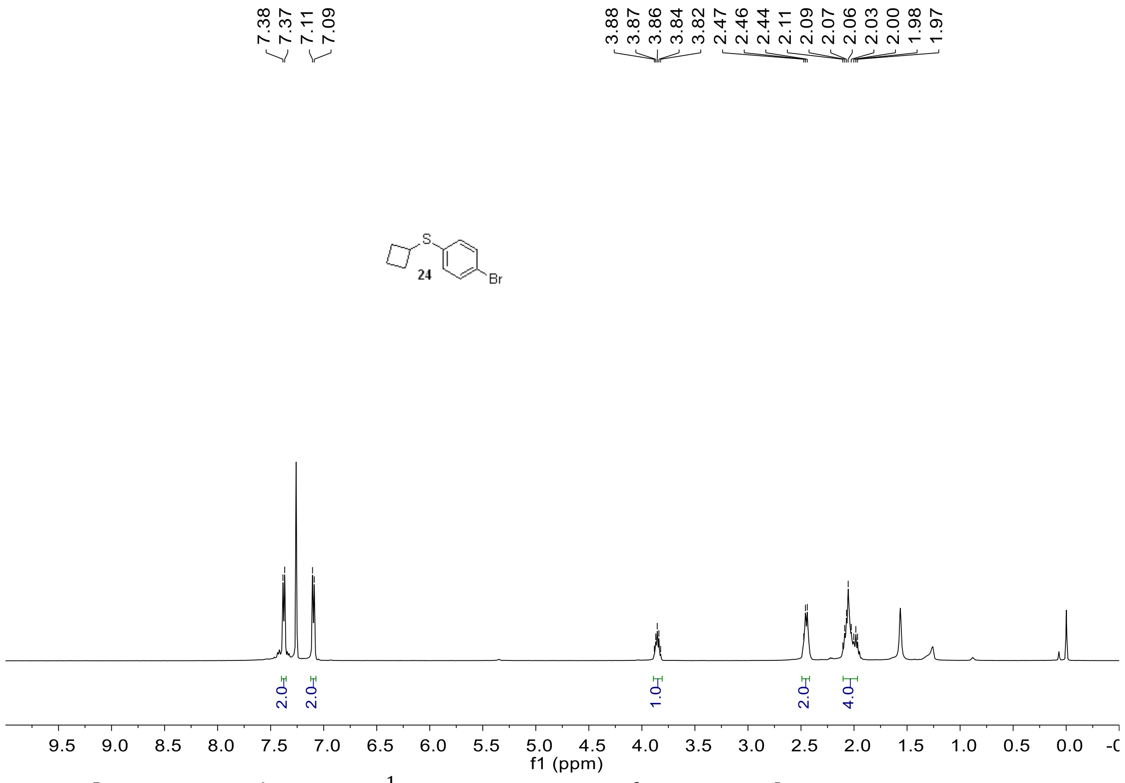

Supplementary Figure 83. ^{1}H NMR Spectra of compound **24**.

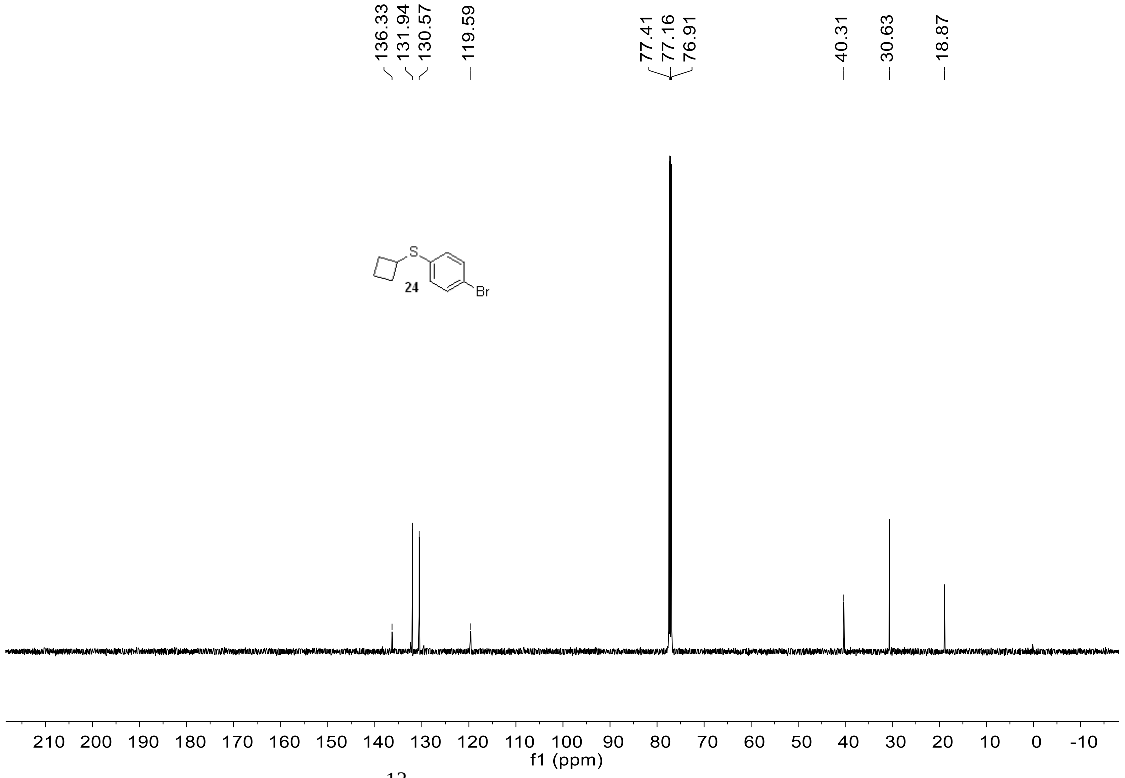

Supplementary Figure 84. ^{13}C NMR Spectra of compound **24**.

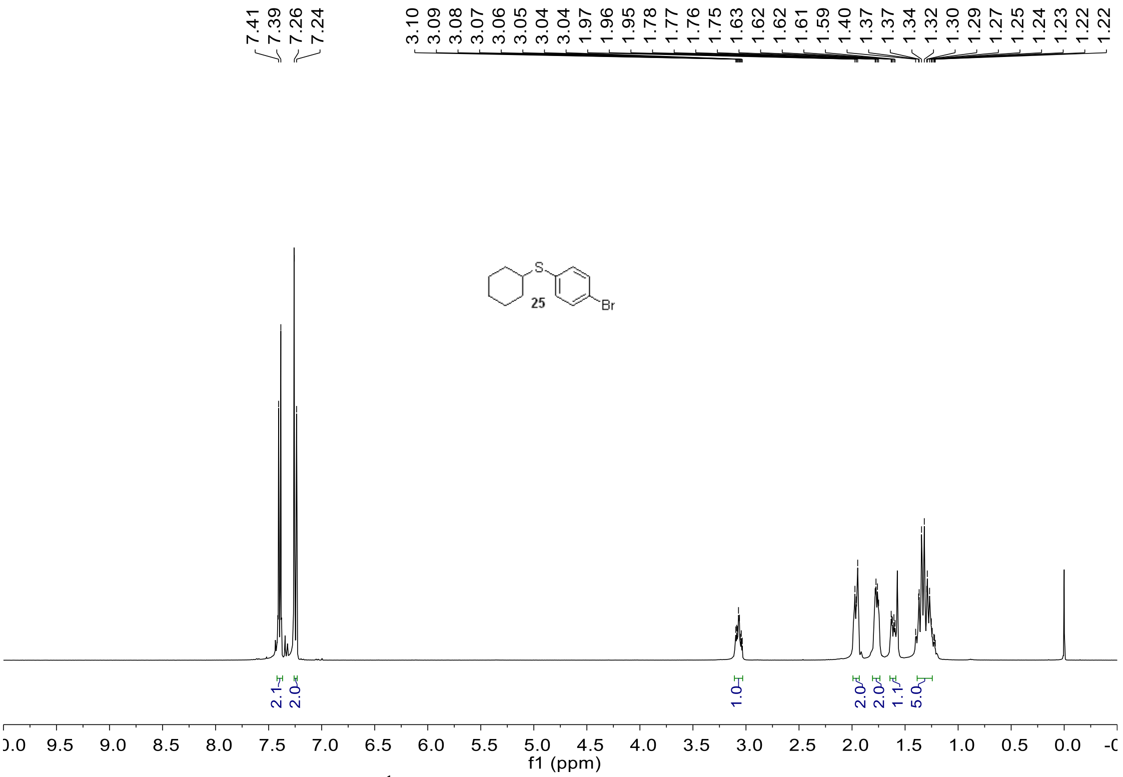

Supplementary Figure 85. ^{1}H NMR Spectra of compound **25**.

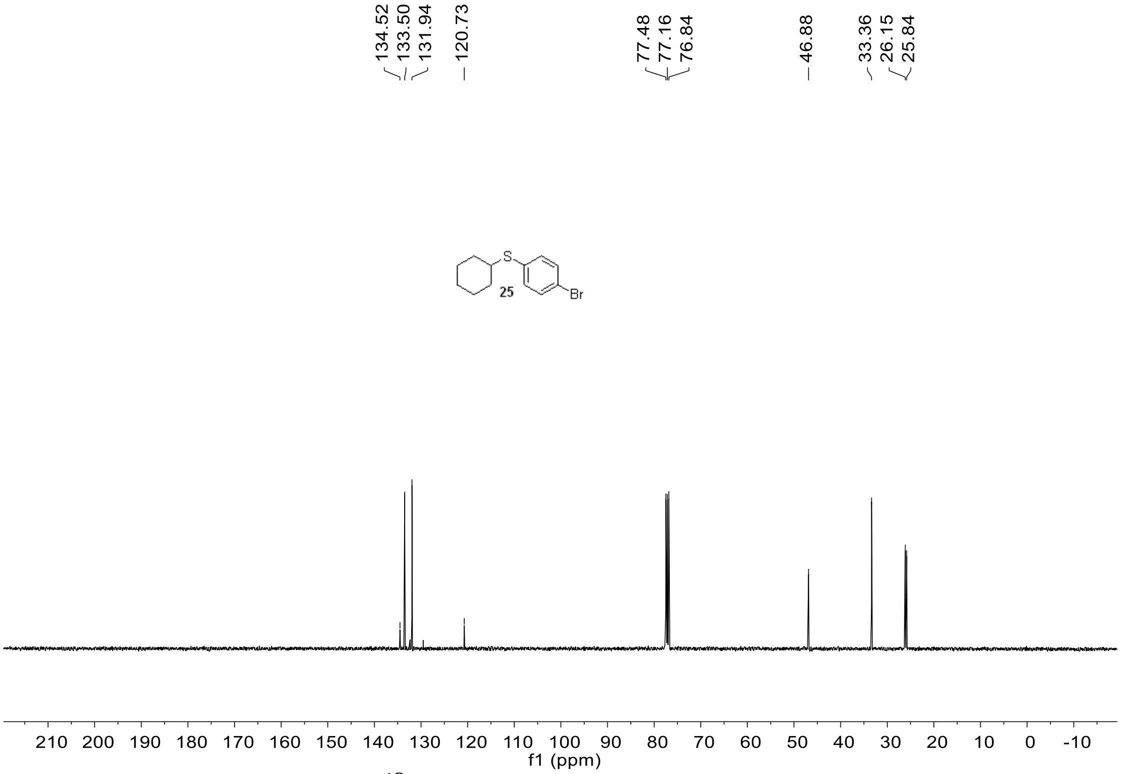

Supplementary Figure 86. ^{13}C NMR Spectra of compound **25**.

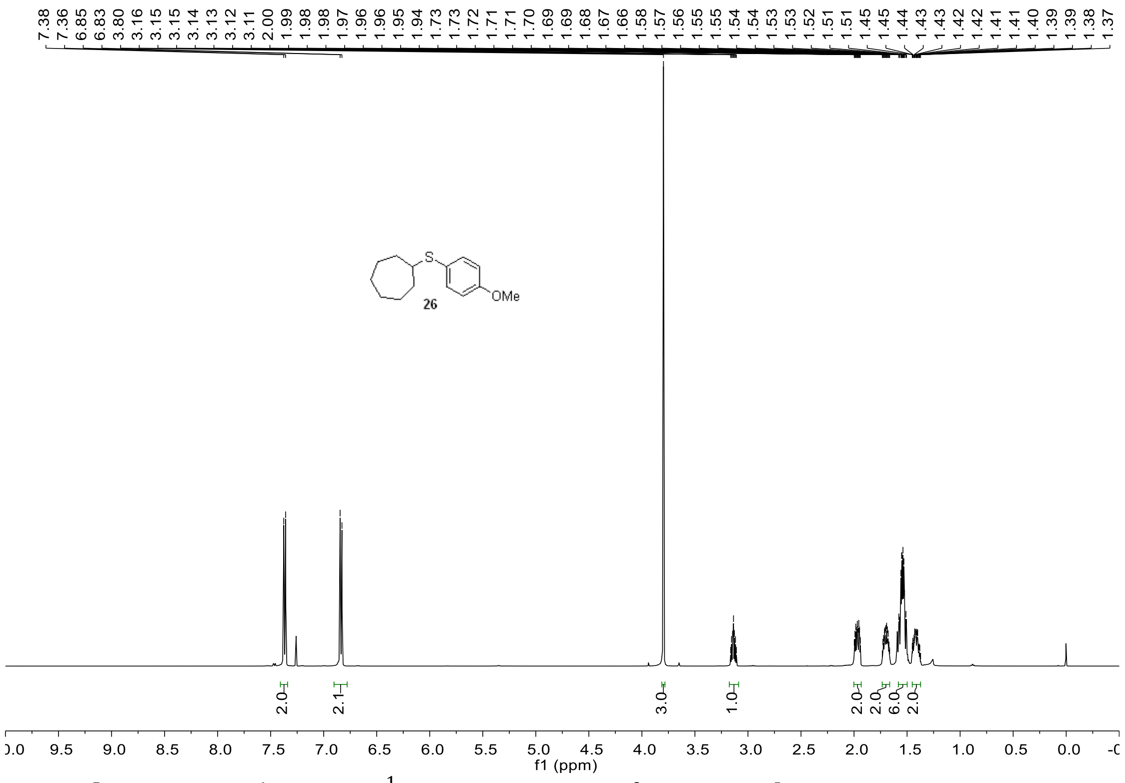

Supplementary Figure 87. ^{1}H NMR Spectra of compound **26**.

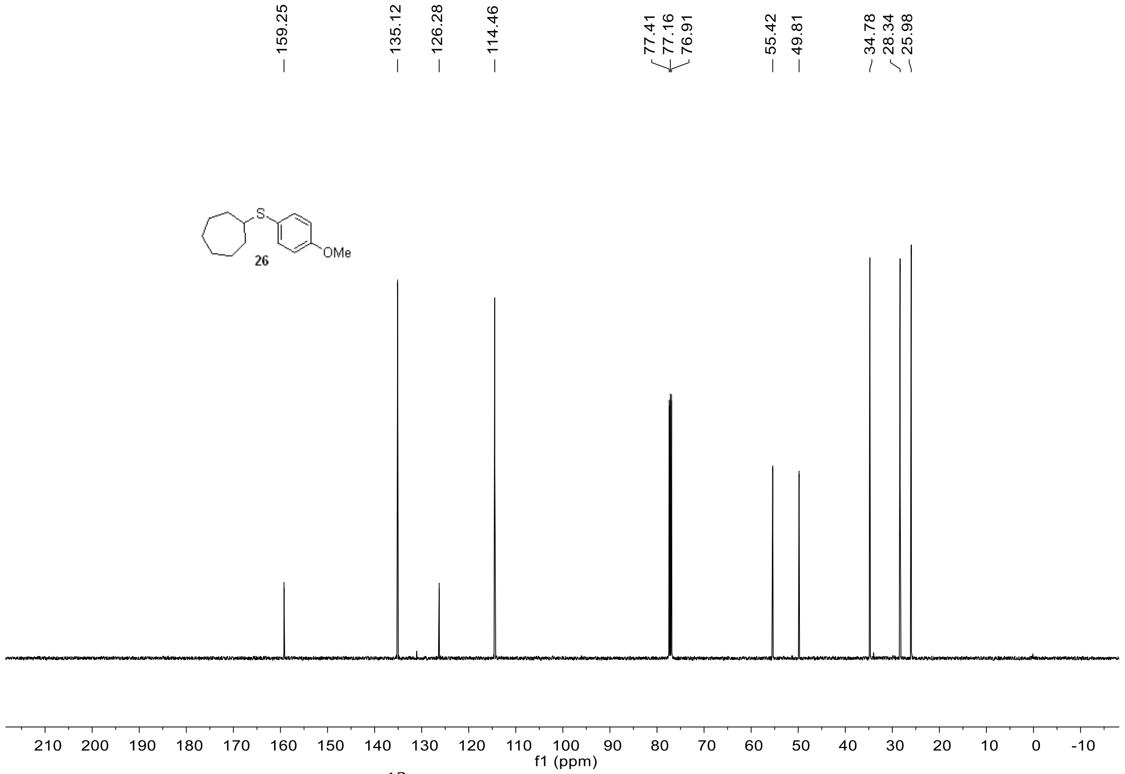

Supplementary Figure 88. ^{13}C NMR Spectra of compound **26**.

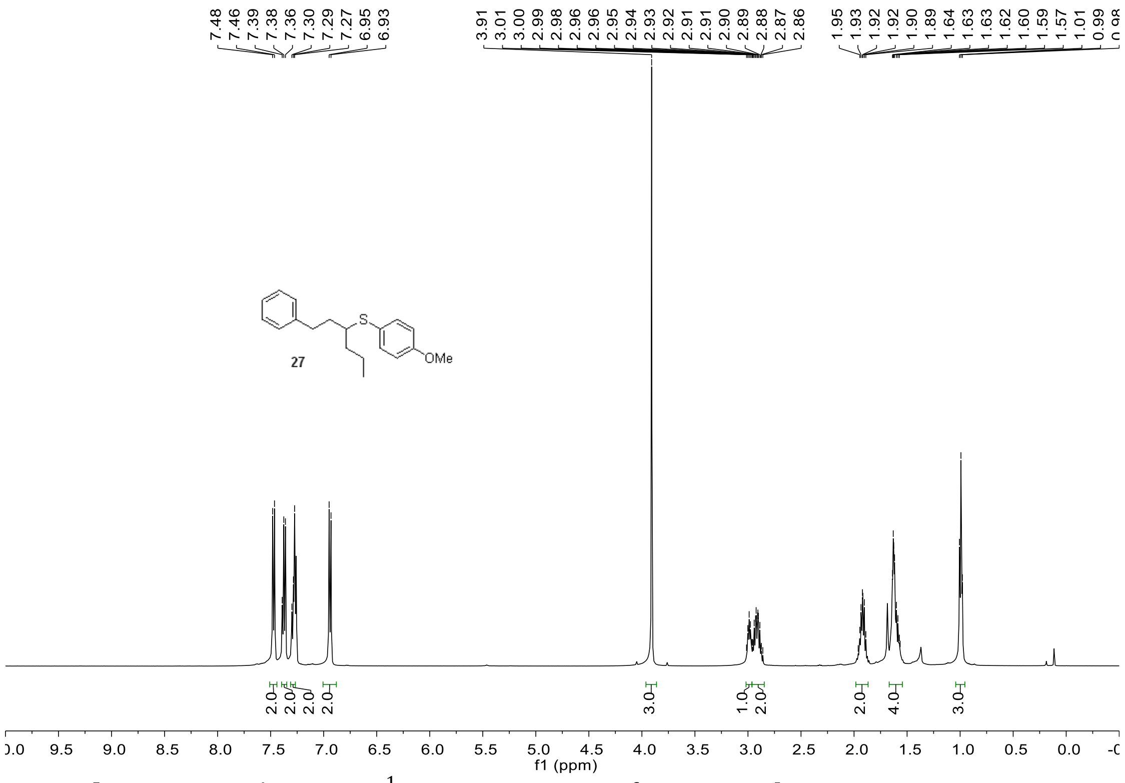

Supplementary Figure 89. ^{1}H NMR Spectra of compound **27**.

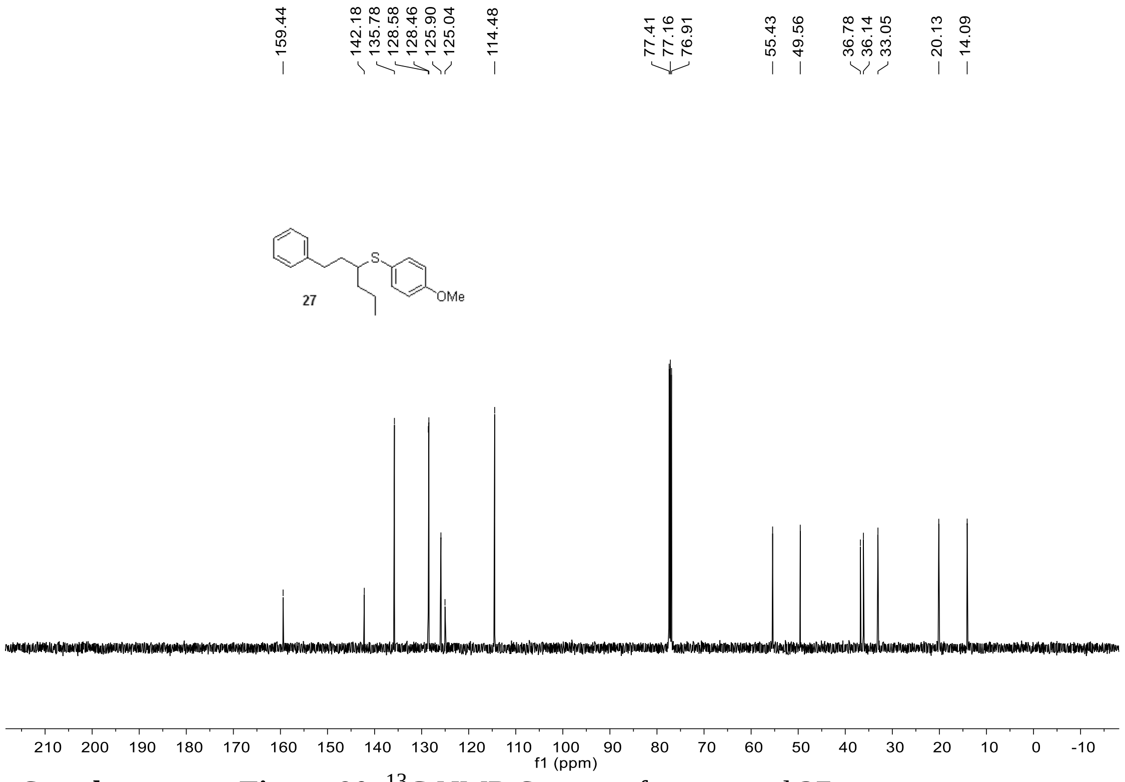

Supplementary Figure 90. ^{13}C NMR Spectra of compound **27**.

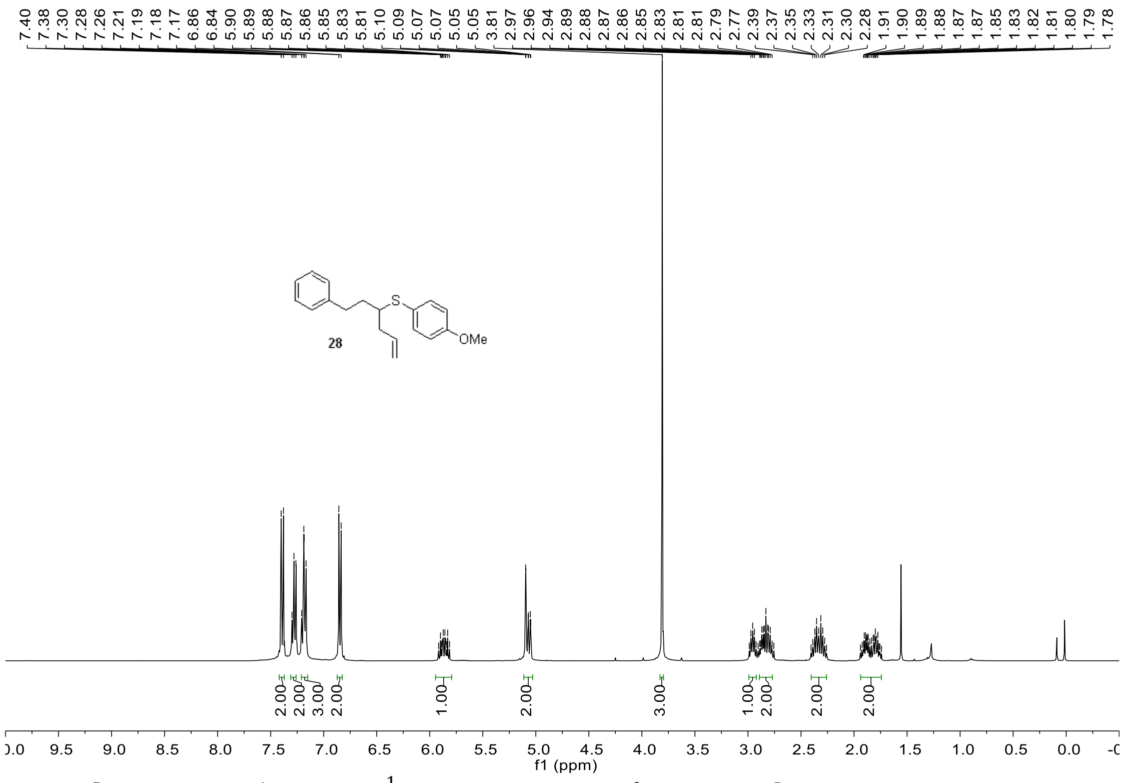

Supplementary Figure 91. ^{1}H NMR Spectra of compound **28**.

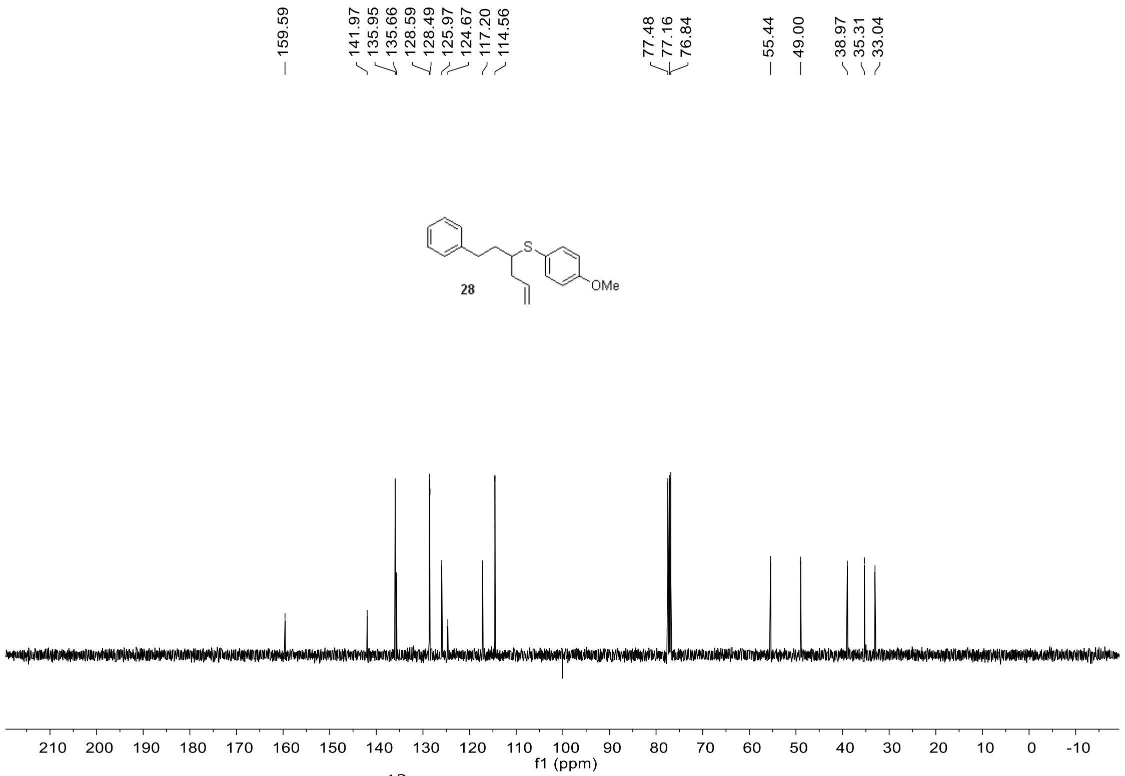

Supplementary Figure 92. ^{13}C NMR Spectra of compound **28**.

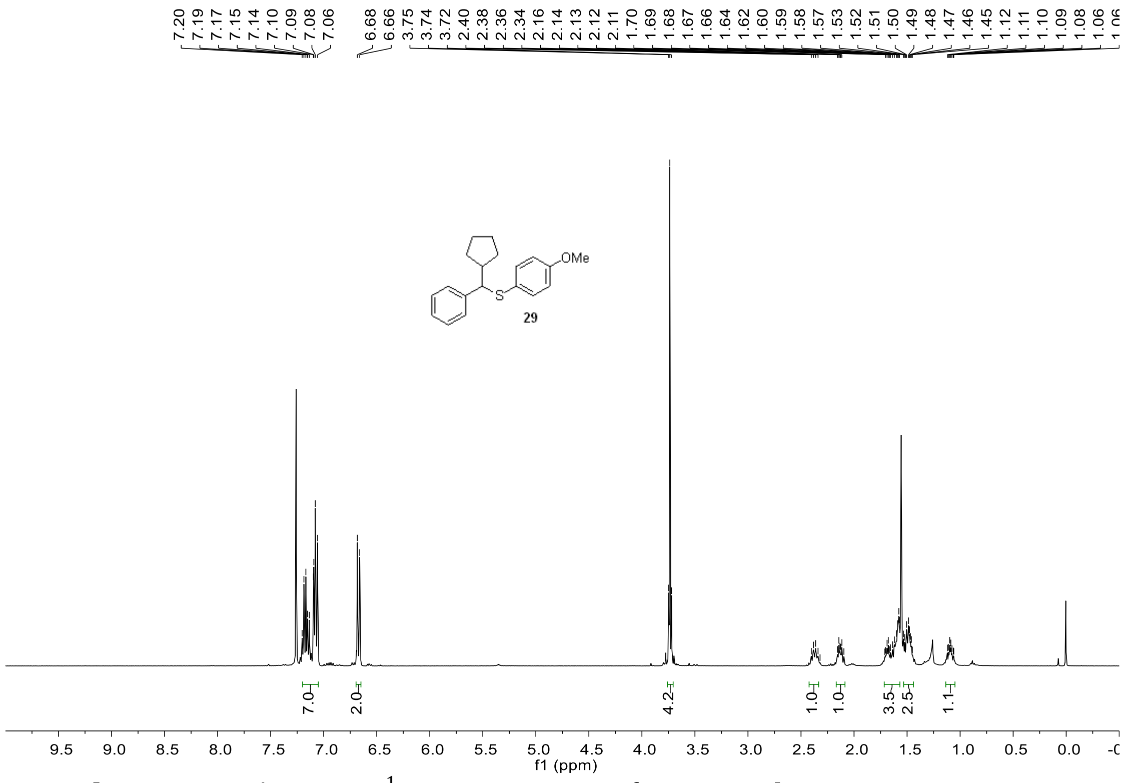

Supplementary Figure 93. ^{1}H NMR Spectra of compound **29**.

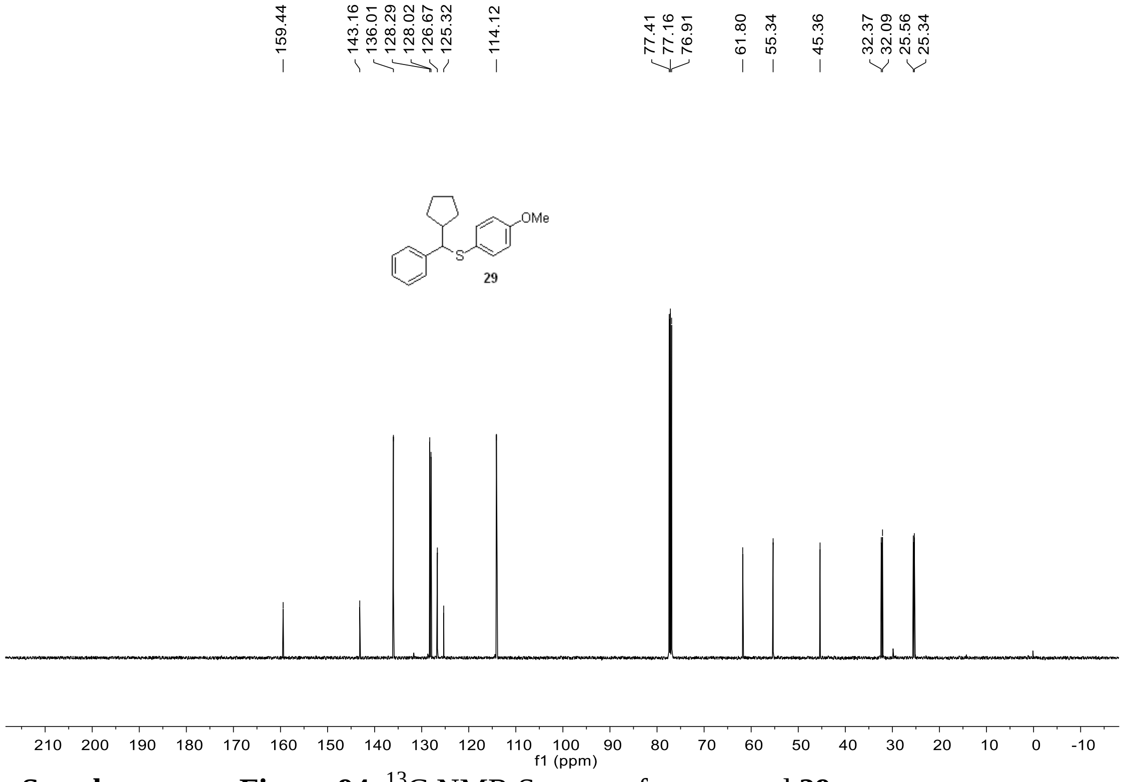

Supplementary Figure 94. ^{13}C NMR Spectra of compound **29**.

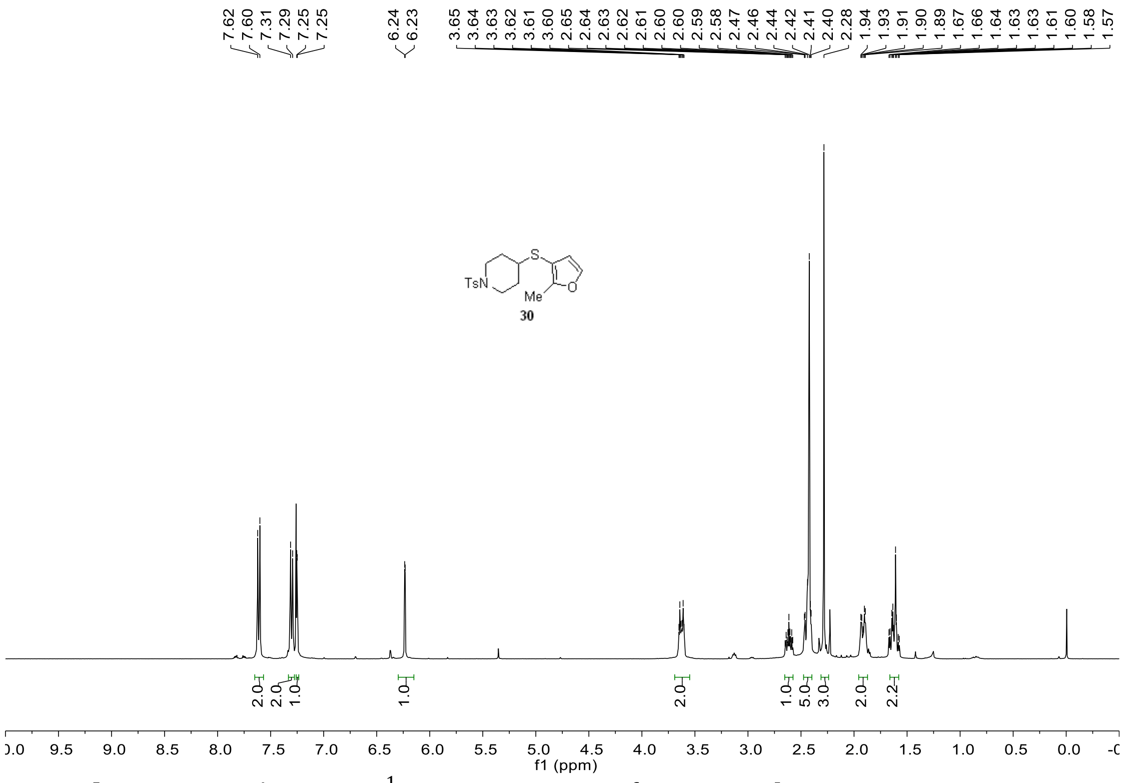

Supplementary Figure 95. ^{1}H NMR Spectra of compound **30**.

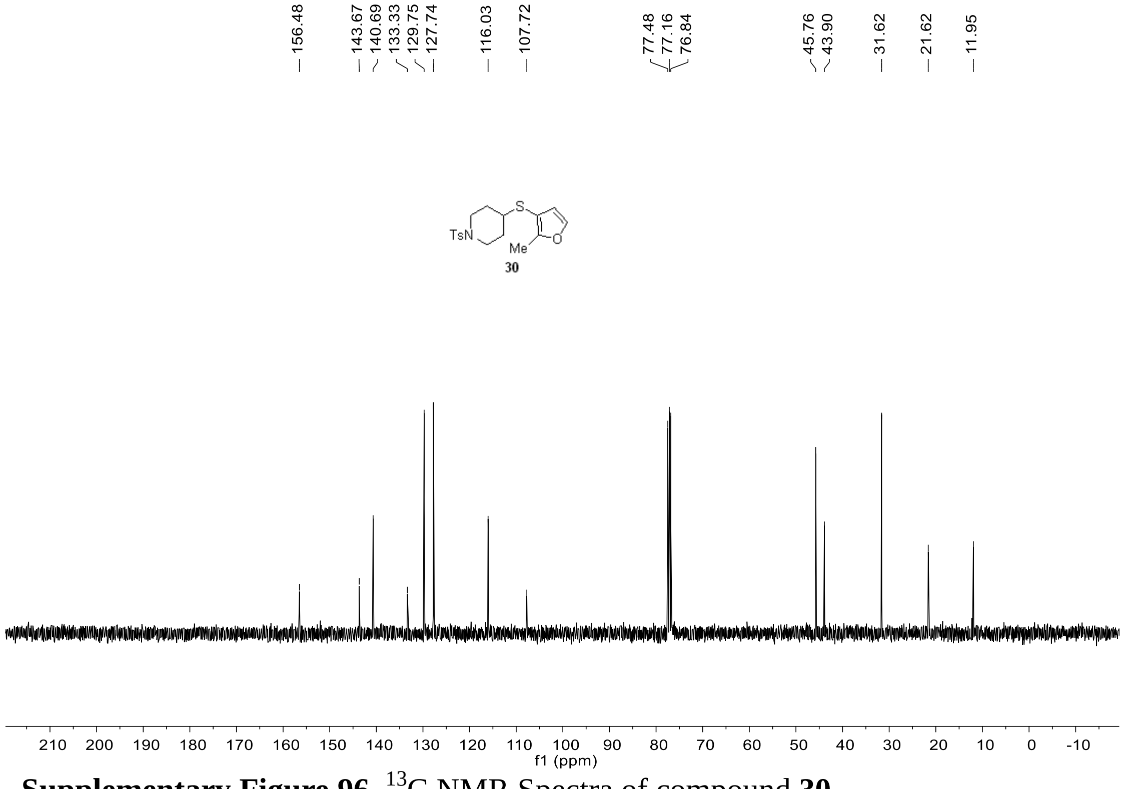

Supplementary Figure 96. ^{13}C NMR Spectra of compound **30**.

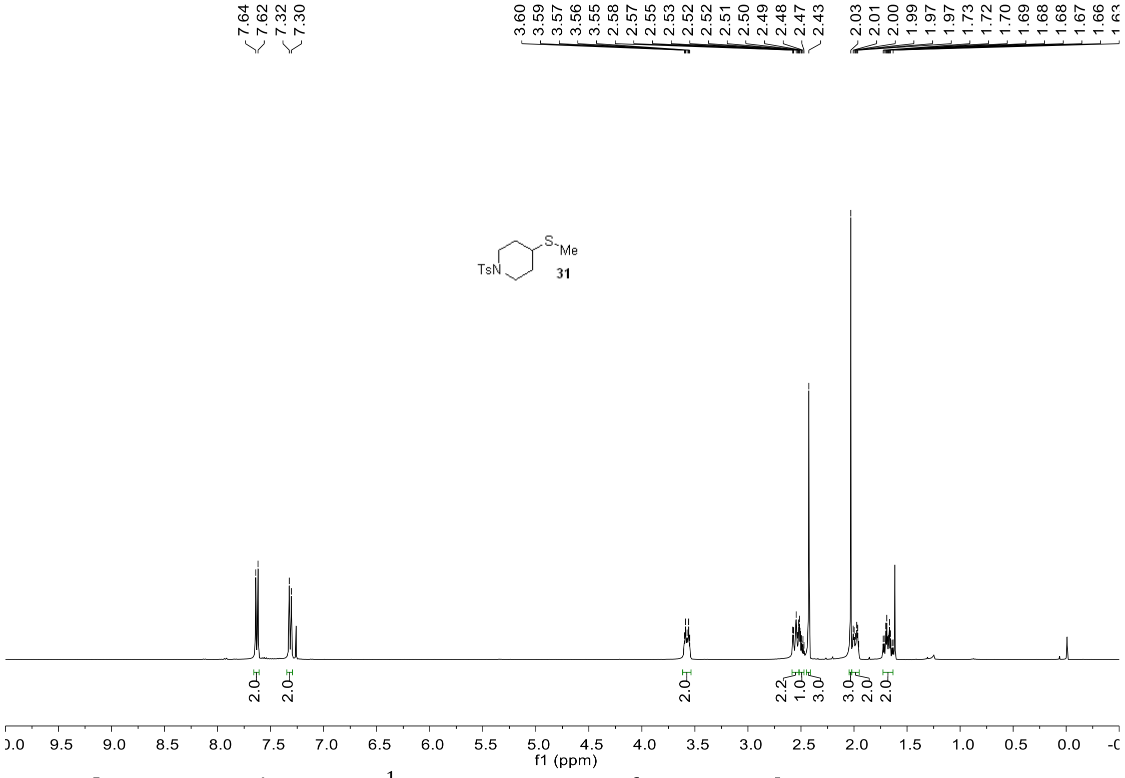

Supplementary Figure 97. ^{1}H NMR Spectra of compound **31**.

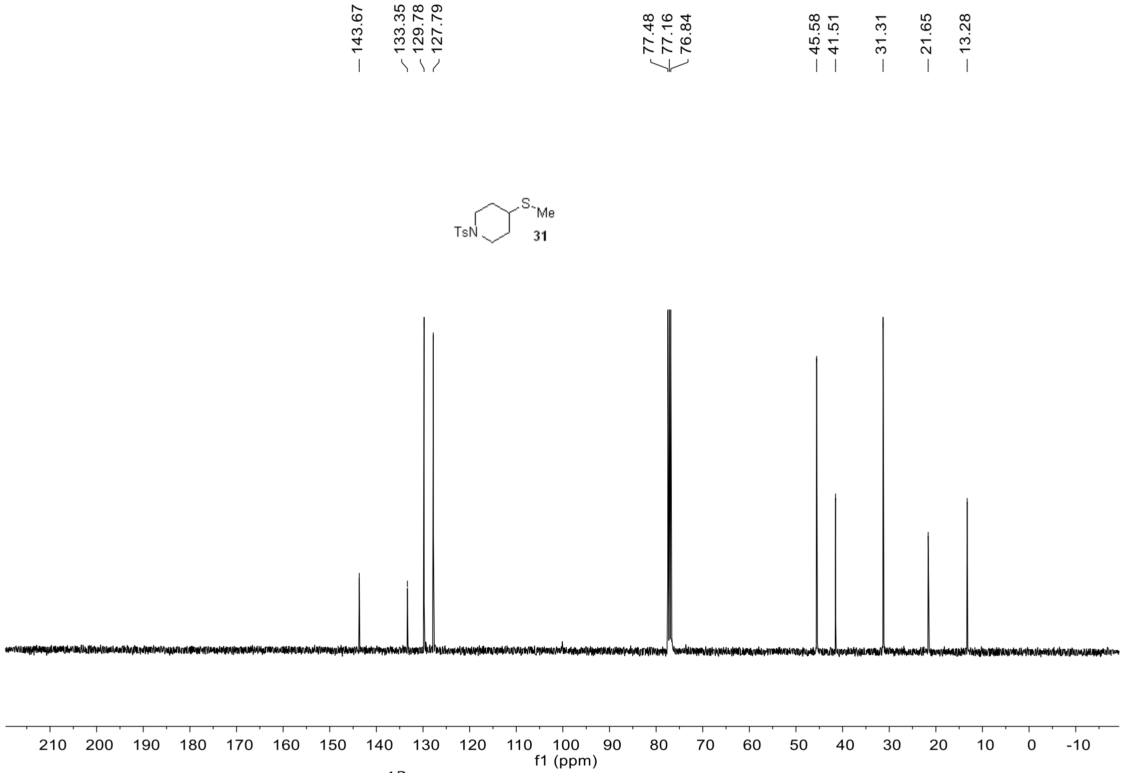

Supplementary Figure 98. ^{13}C NMR Spectra of compound **31**.

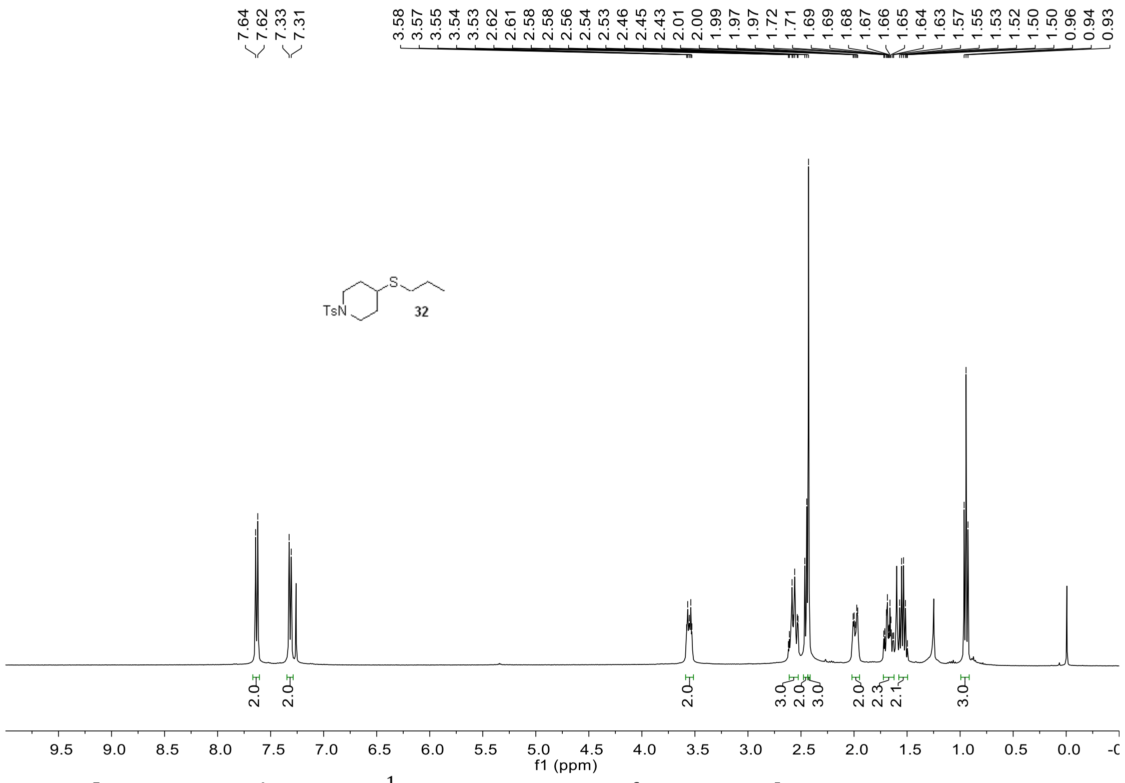

Supplementary Figure 99. ^{1}H NMR Spectra of compound **32**.

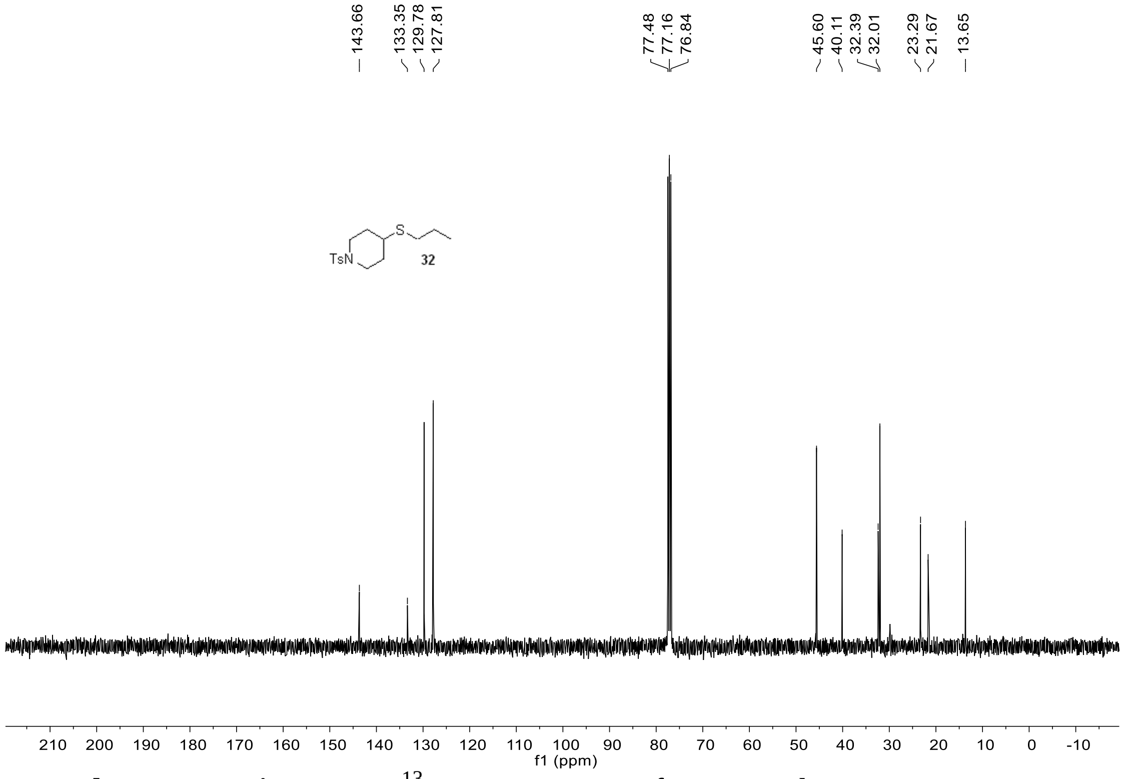

Supplementary Figure 100. ^{13}C NMR Spectra of compound **32**.

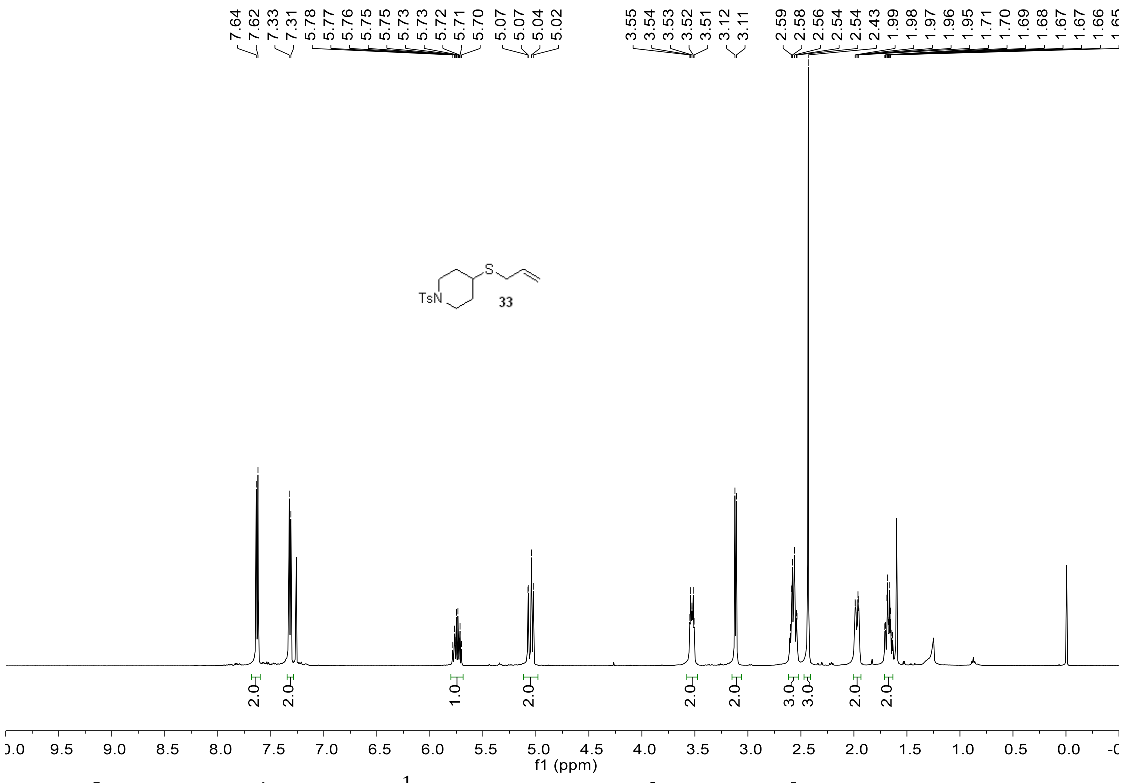

Supplementary Figure 101. ^{1}H NMR Spectra of compound **33**.

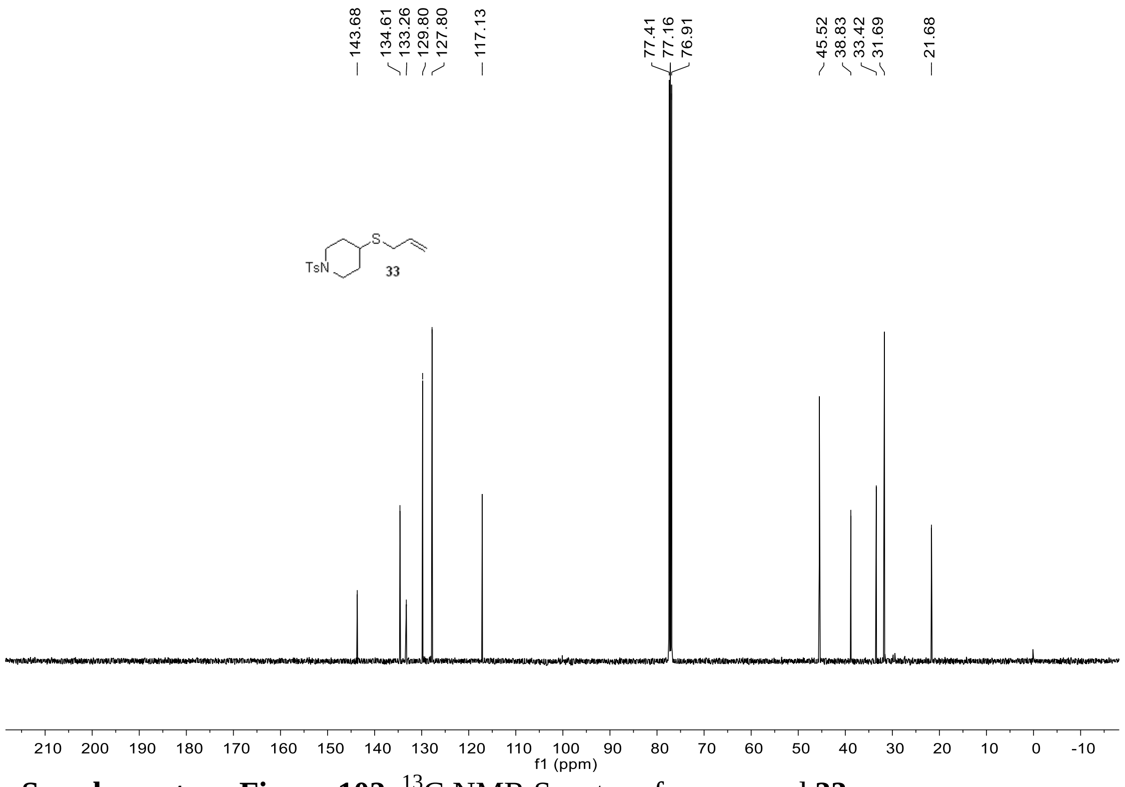

Supplementary Figure 102. ^{13}C NMR Spectra of compound **33**.

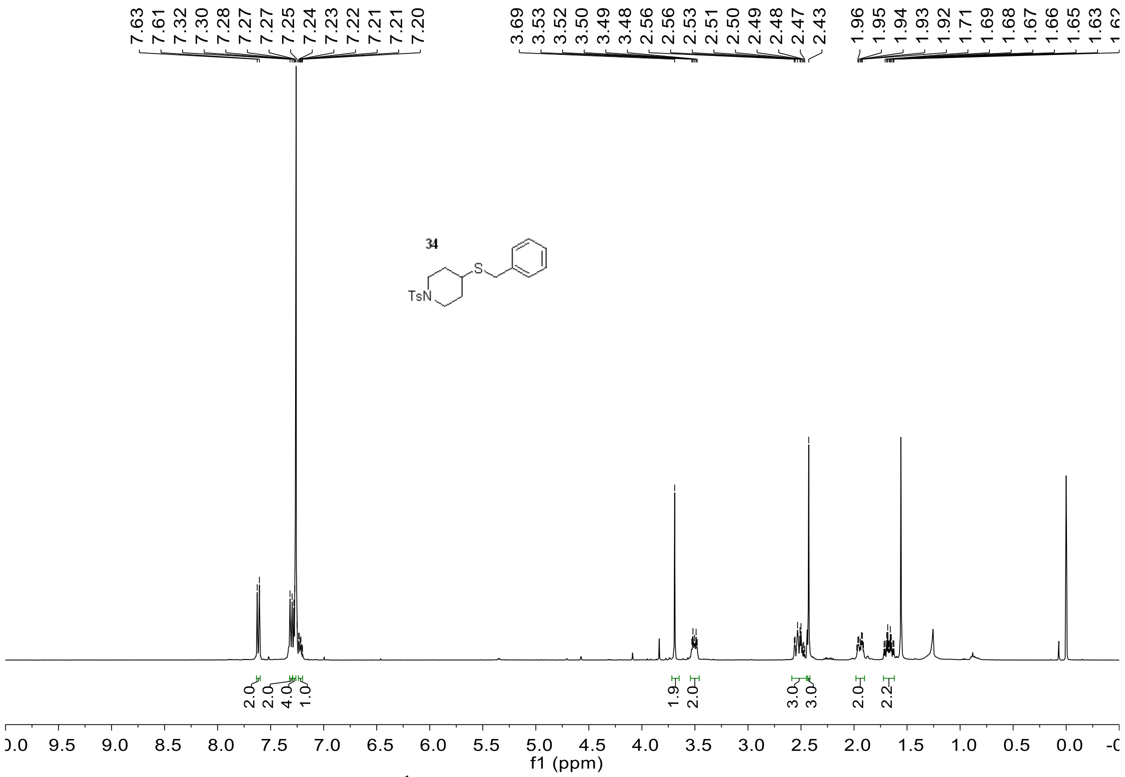

Supplementary Figure 103. ^{1}H NMR Spectra of compound **34**.

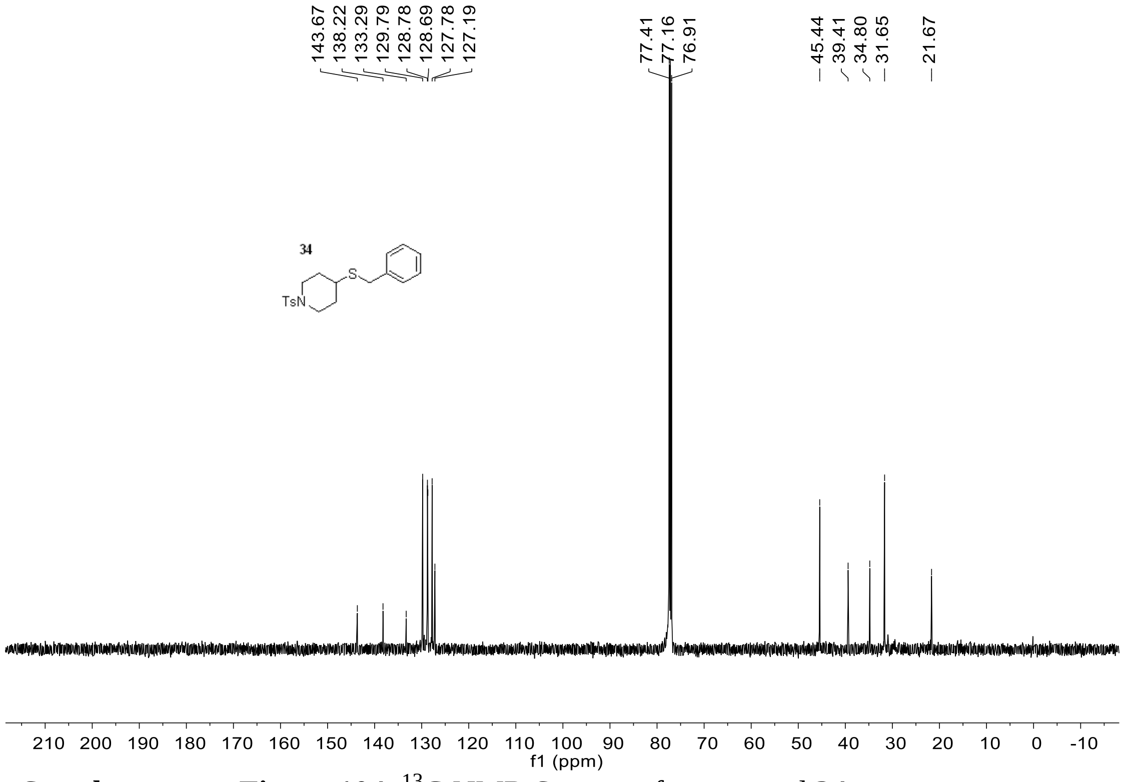

Supplementary Figure 104. ^{13}C NMR Spectra of compound **34**.

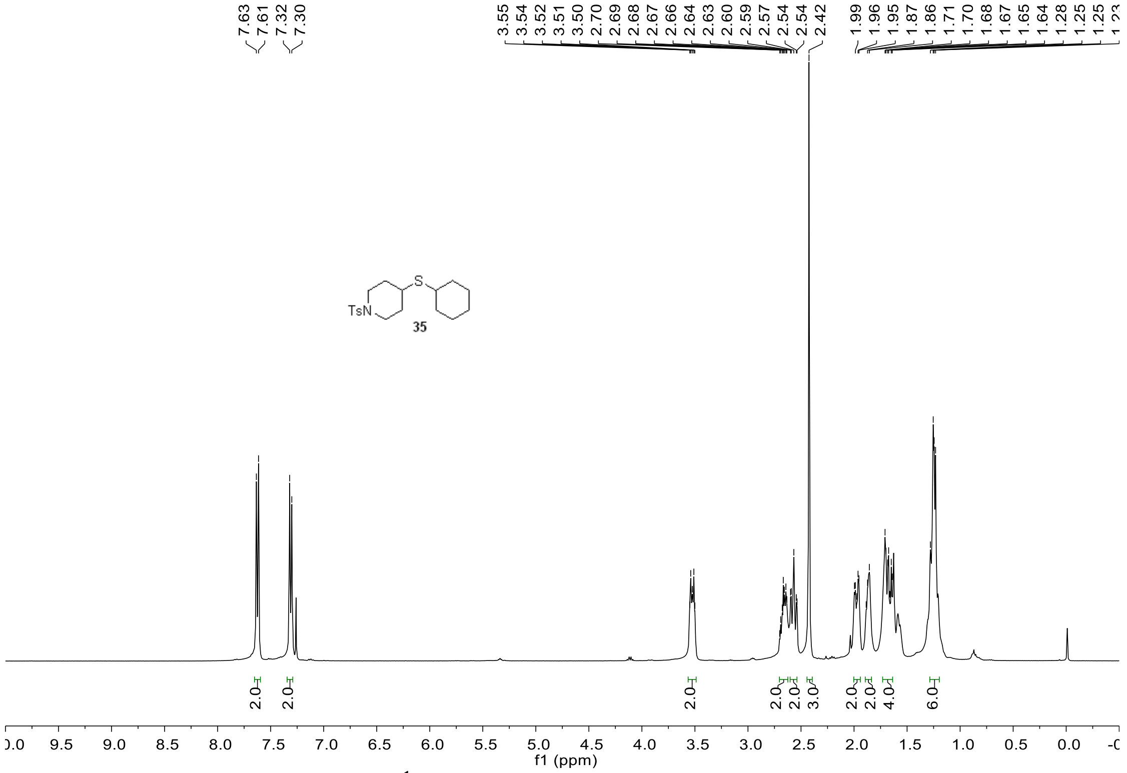

Supplementary Figure 105. ^{1}H NMR Spectra of compound **35**.

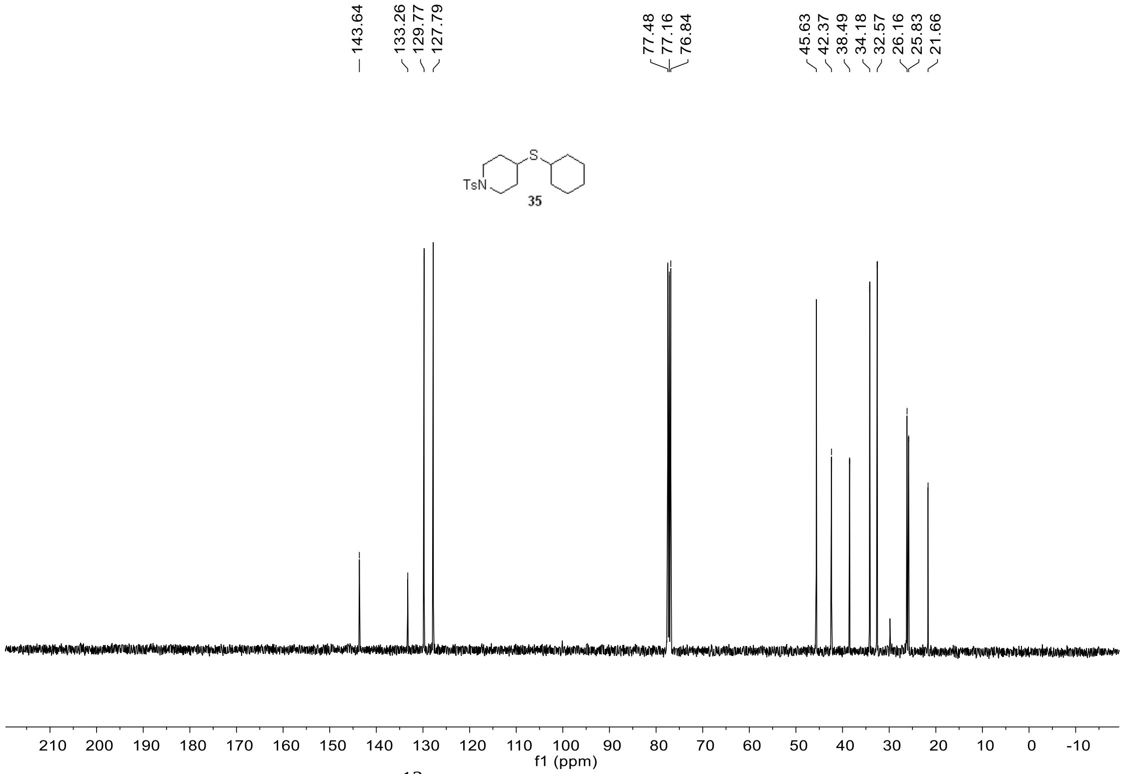

Supplementary Figure 106. ^{13}C NMR Spectra of compound **35**.

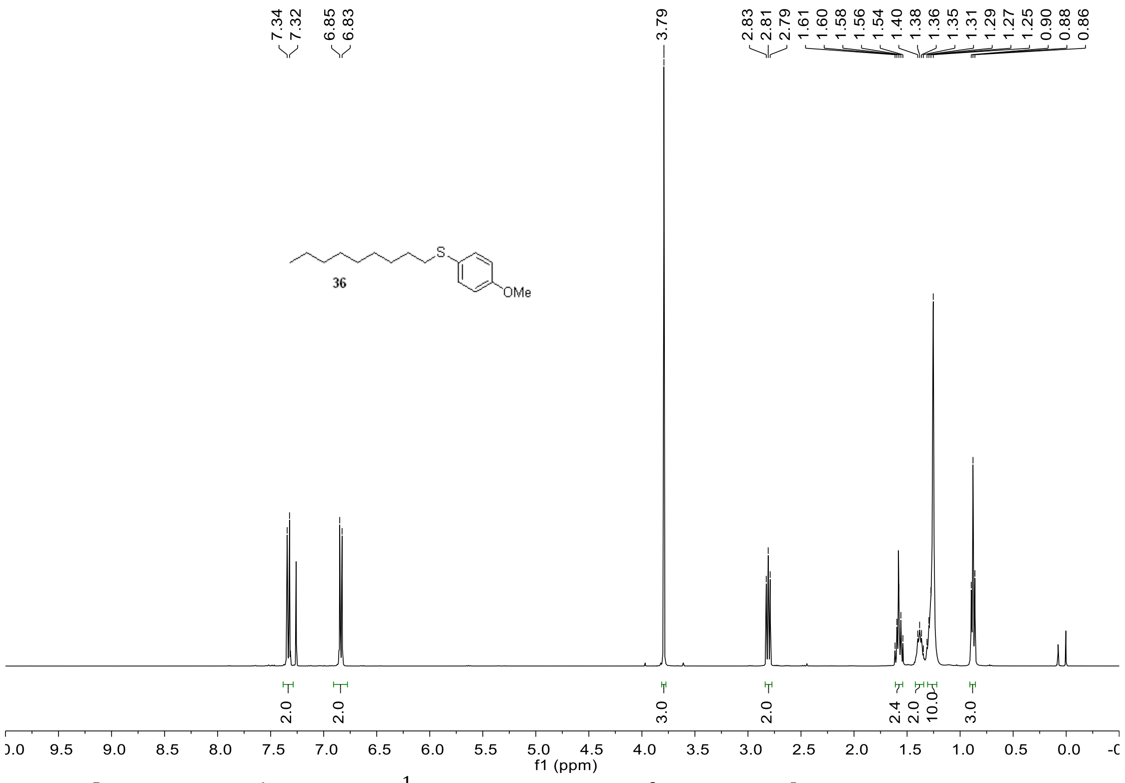

Supplementary Figure 107. ^{1}H NMR Spectra of compound **36**.

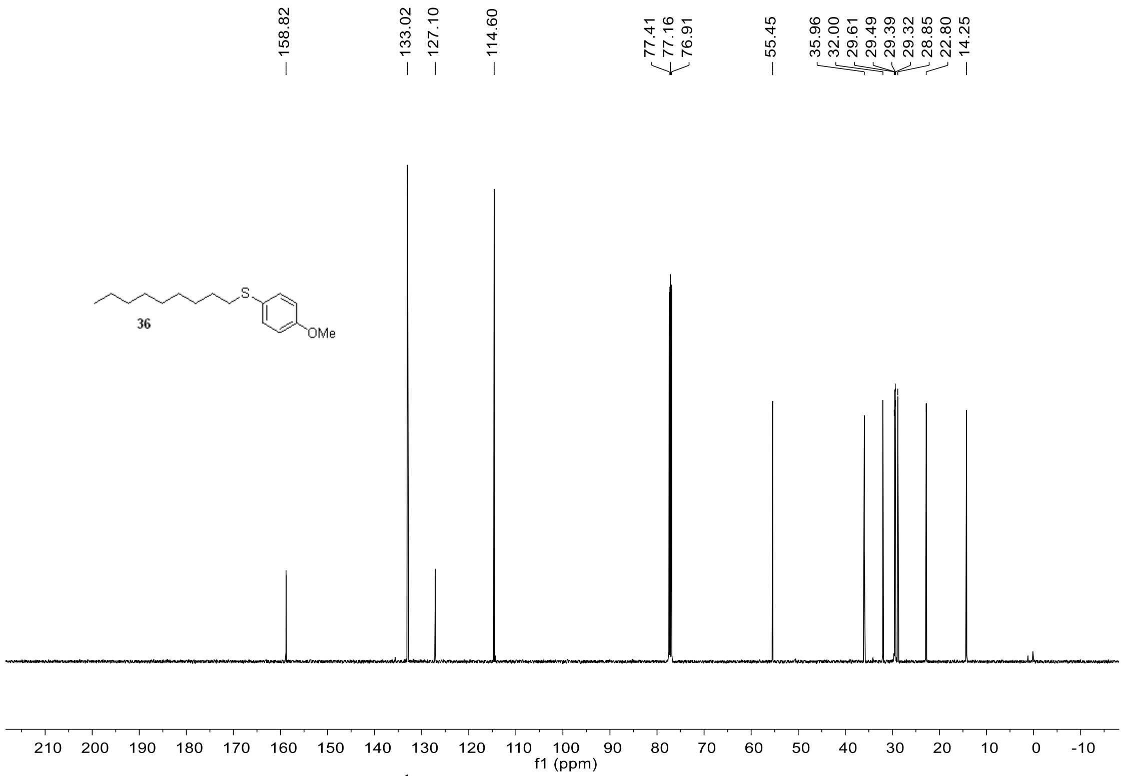

Supplementary Figure 108. ^{1}H NMR Spectra of compound **36**.

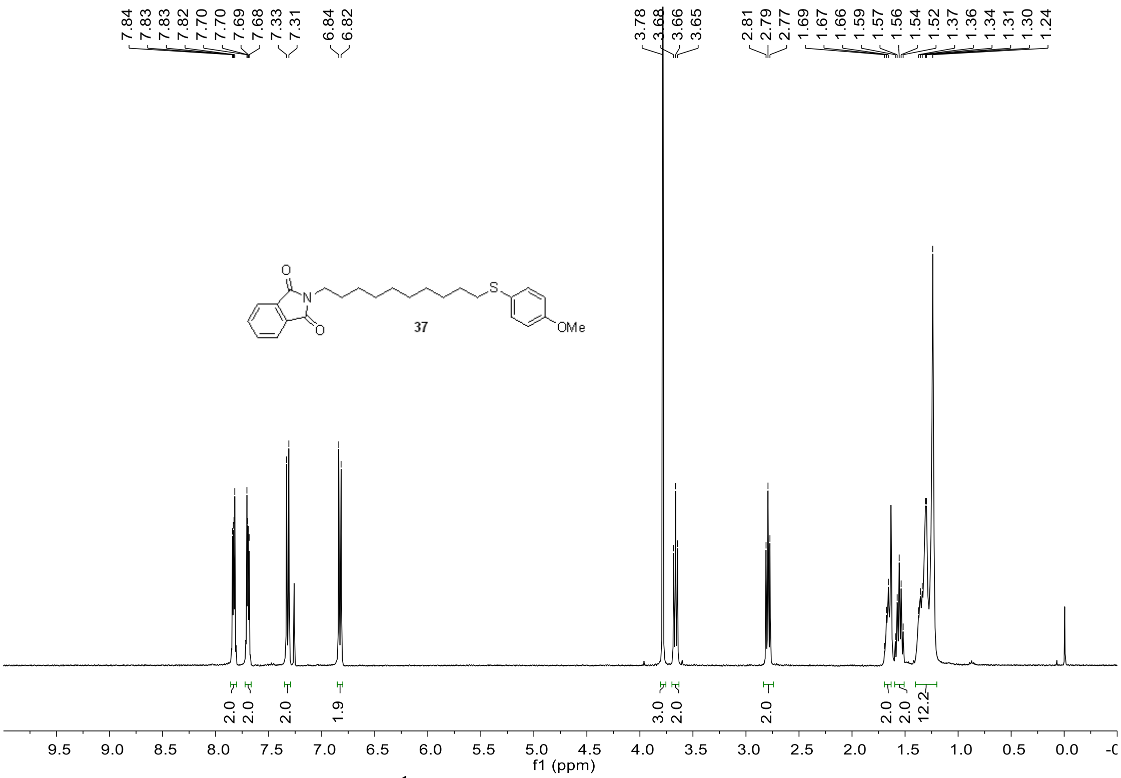

Supplementary Figure 109. ^{1}H NMR Spectra of compound **37**.

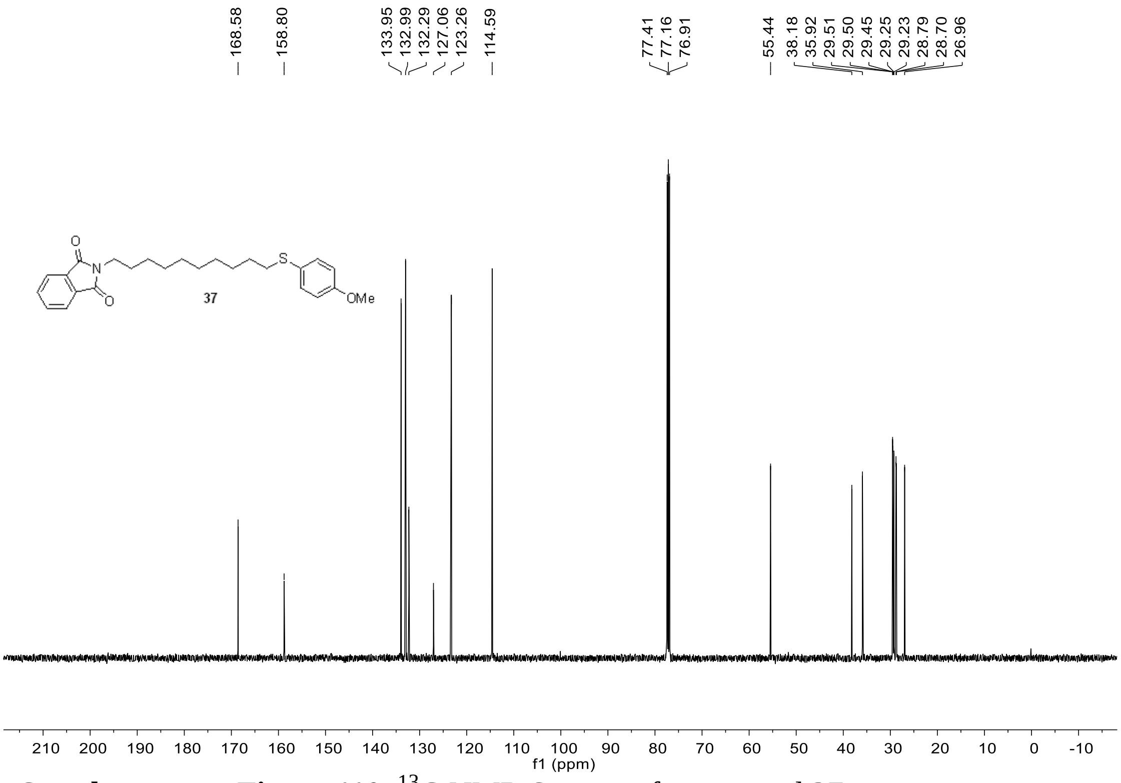

Supplementary Figure 110. ^{13}C NMR Spectra of compound **37**.

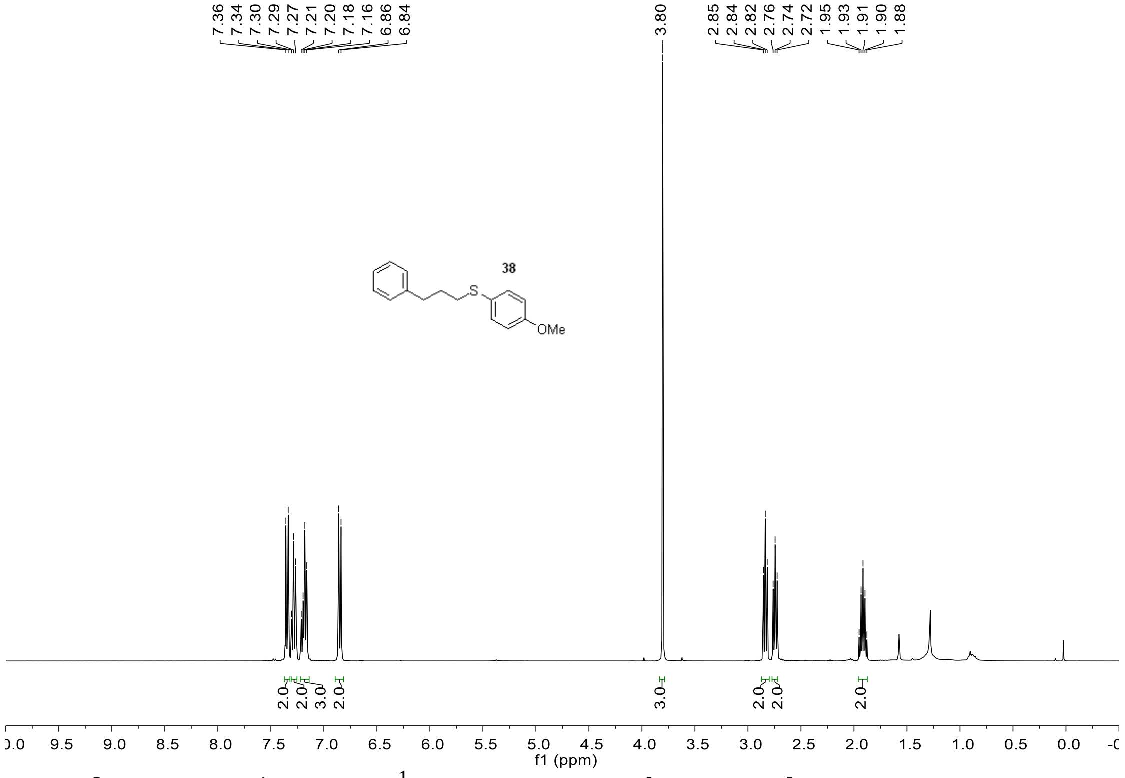

Supplementary Figure 111. ^{1}H NMR Spectra of compound **38**.

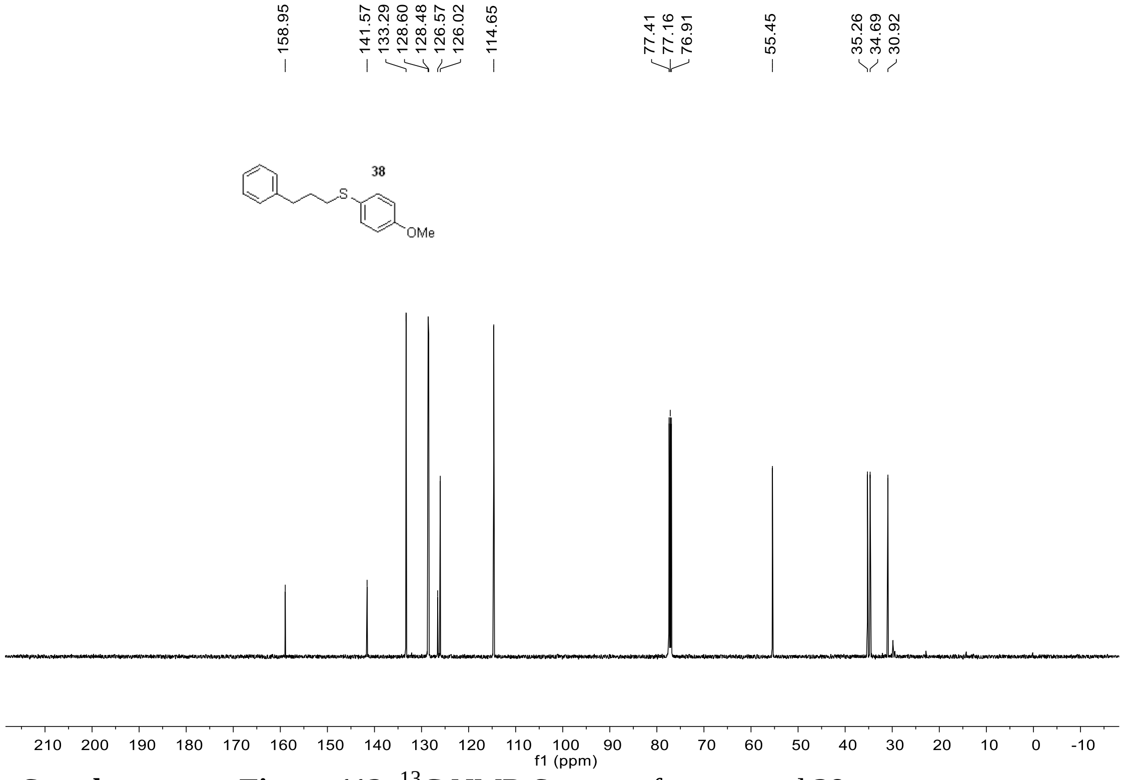

Supplementary Figure 112. ^{13}C NMR Spectra of compound **38**.

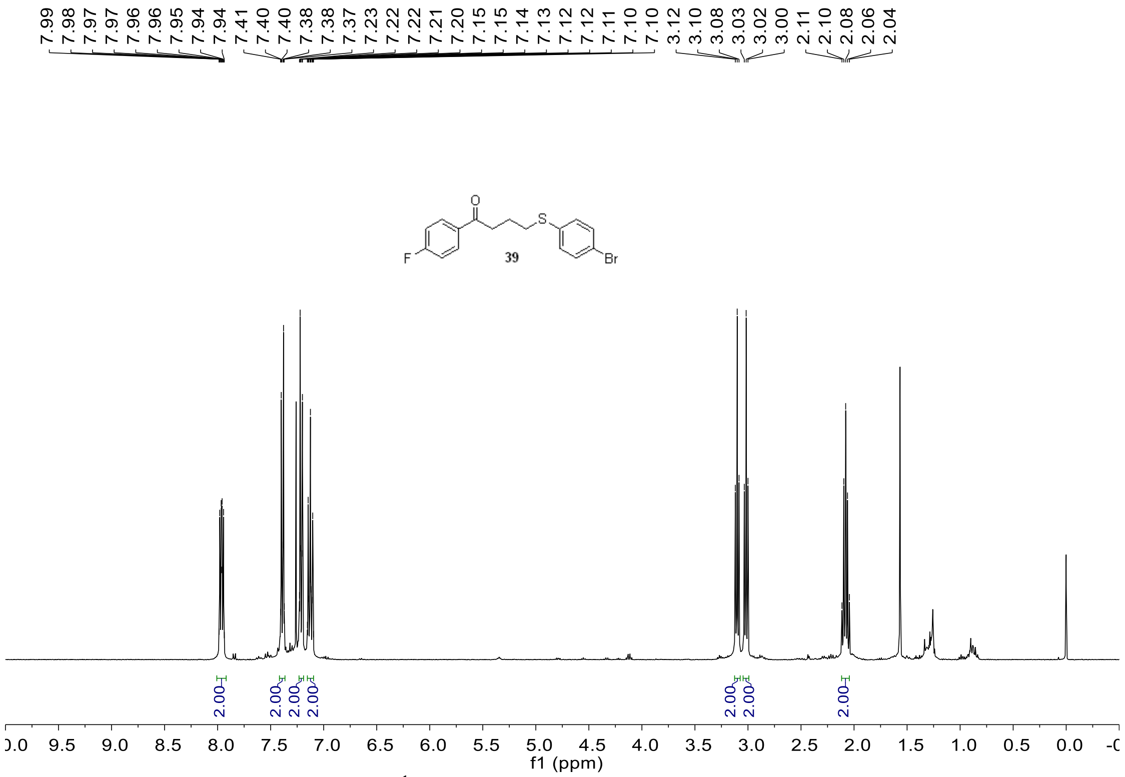

Supplementary Figure 113. ^{1}H NMR Spectra of compound **39**.

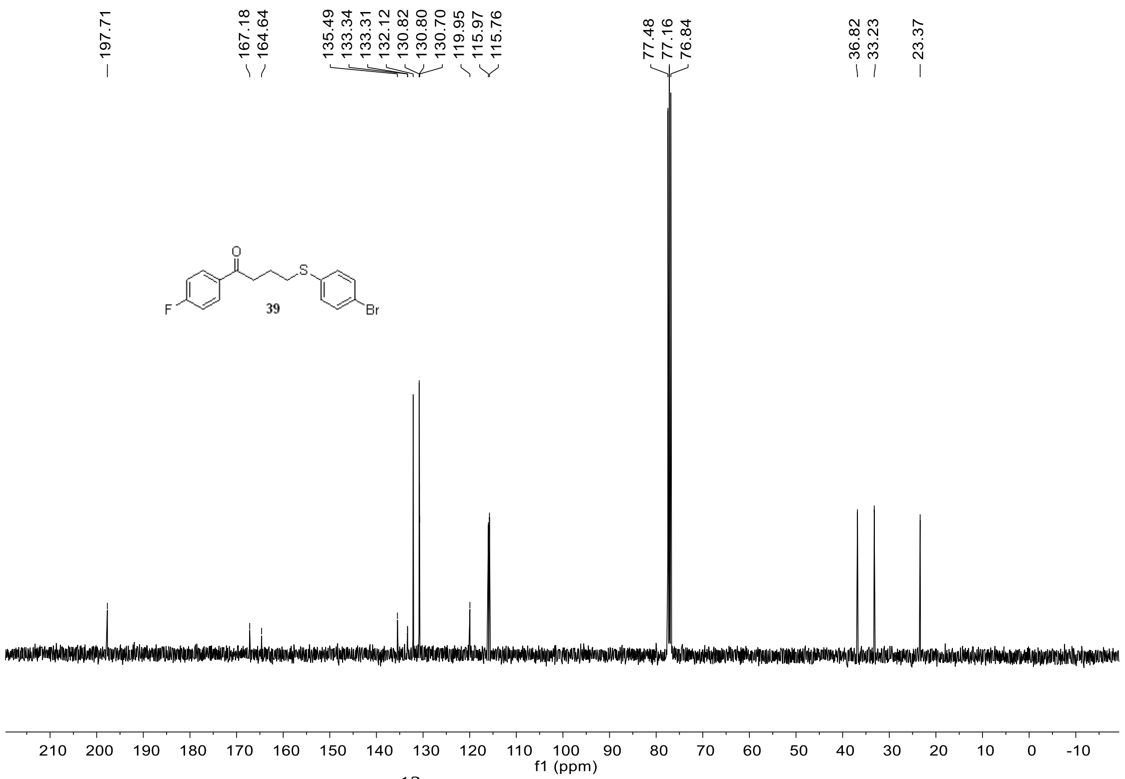

Supplementary Figure 114. ^{13}C NMR Spectra of compound **39**.

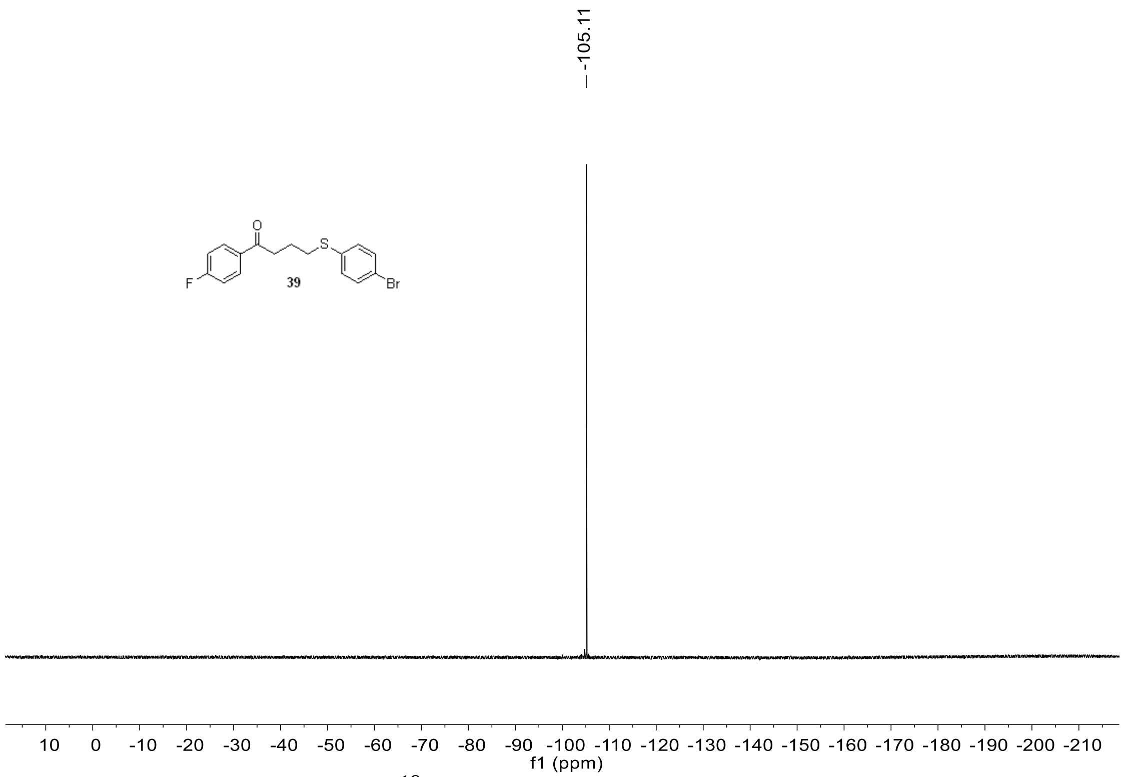

Supplementary Figure 115. ^{19}F NMR Spectra of compound **39**.

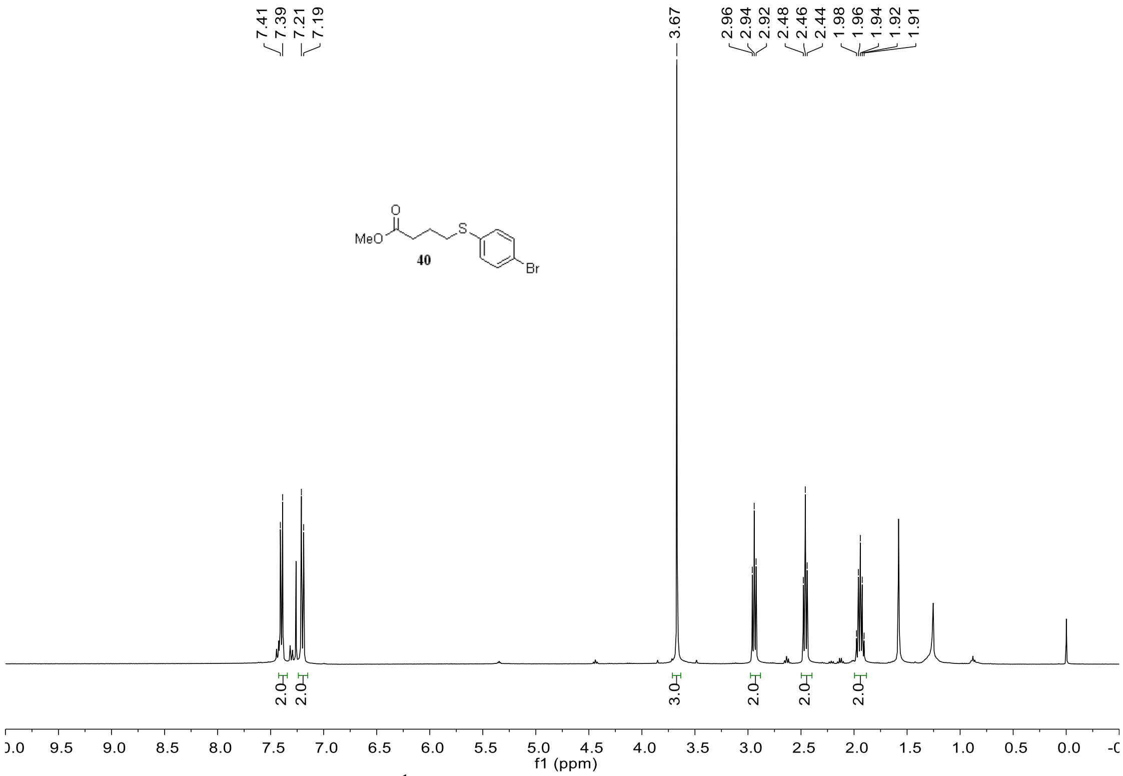

Supplementary Figure 116. ^{1}H NMR Spectra of compound **40**.

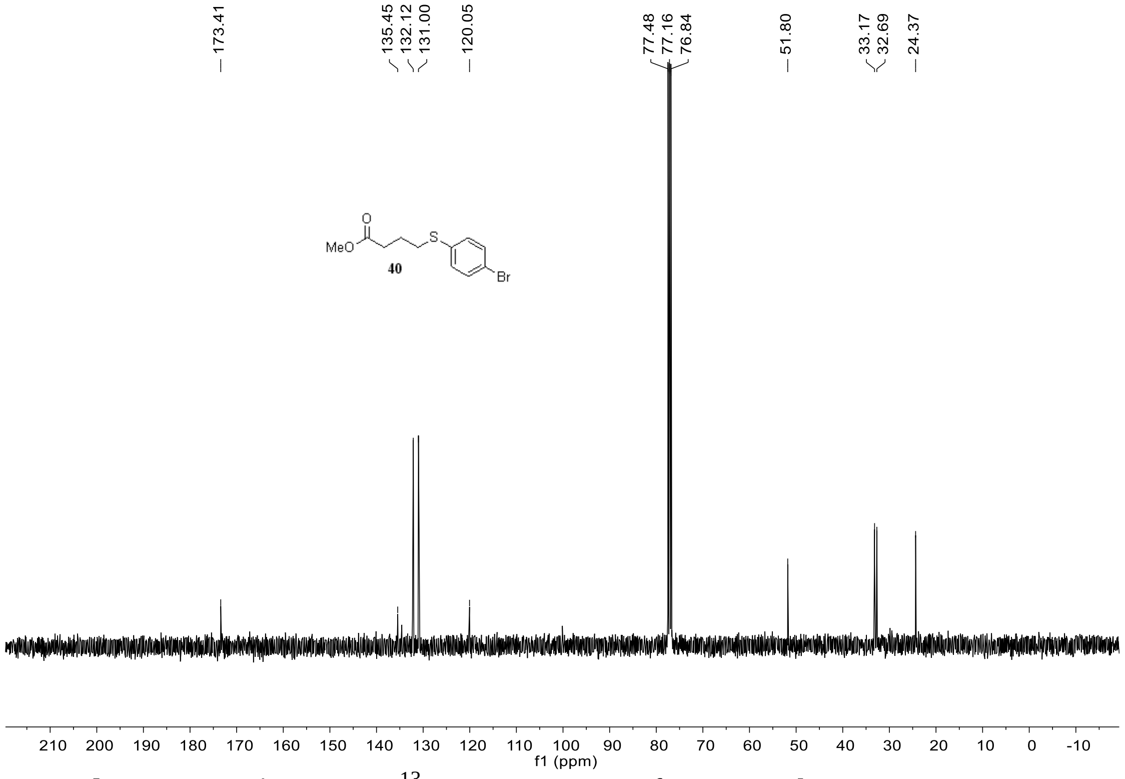

Supplementary Figure 117. ^{13}C NMR Spectra of compound **40**.

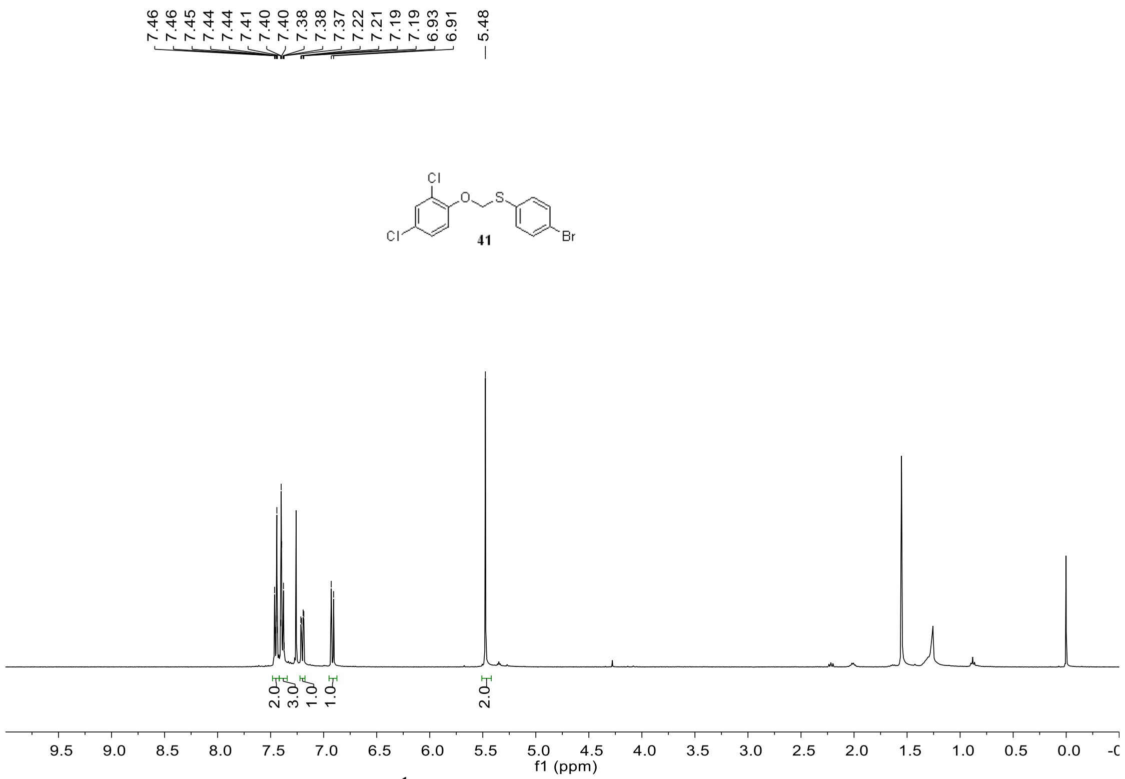

Supplementary Figure 118. ^{1}H NMR Spectra of compound **41**.

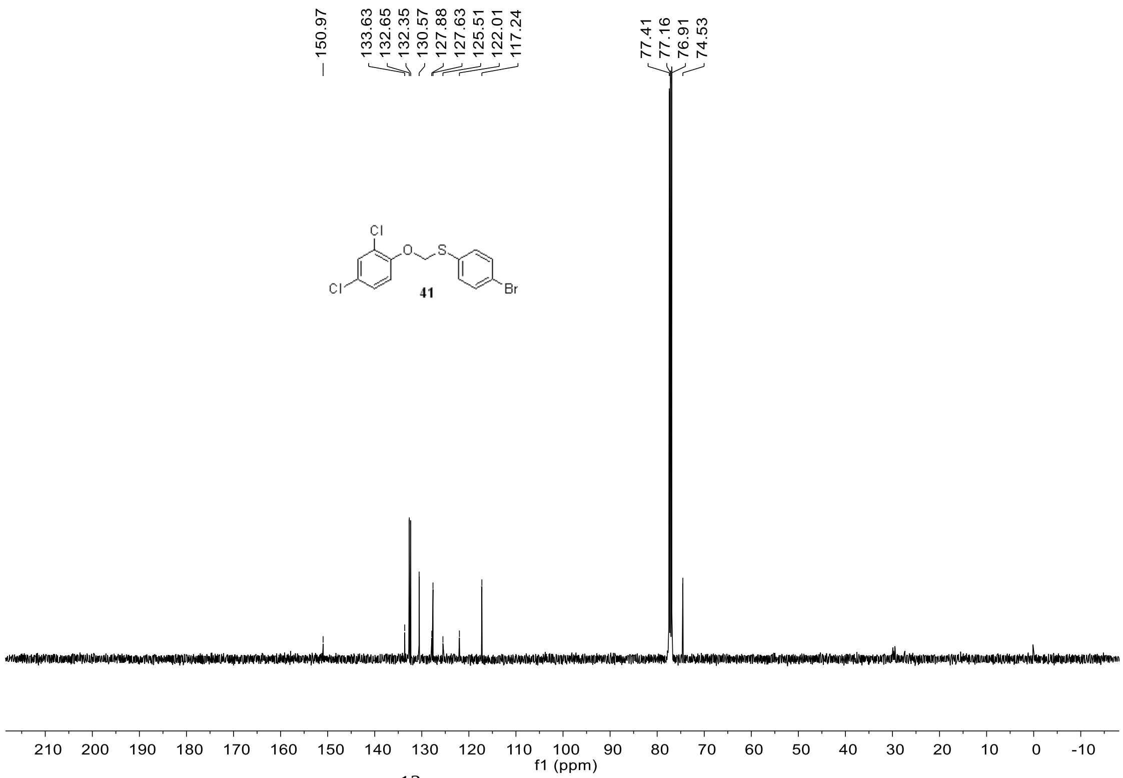

Supplementary Figure 119. ^{13}C NMR Spectra of compound **41**.

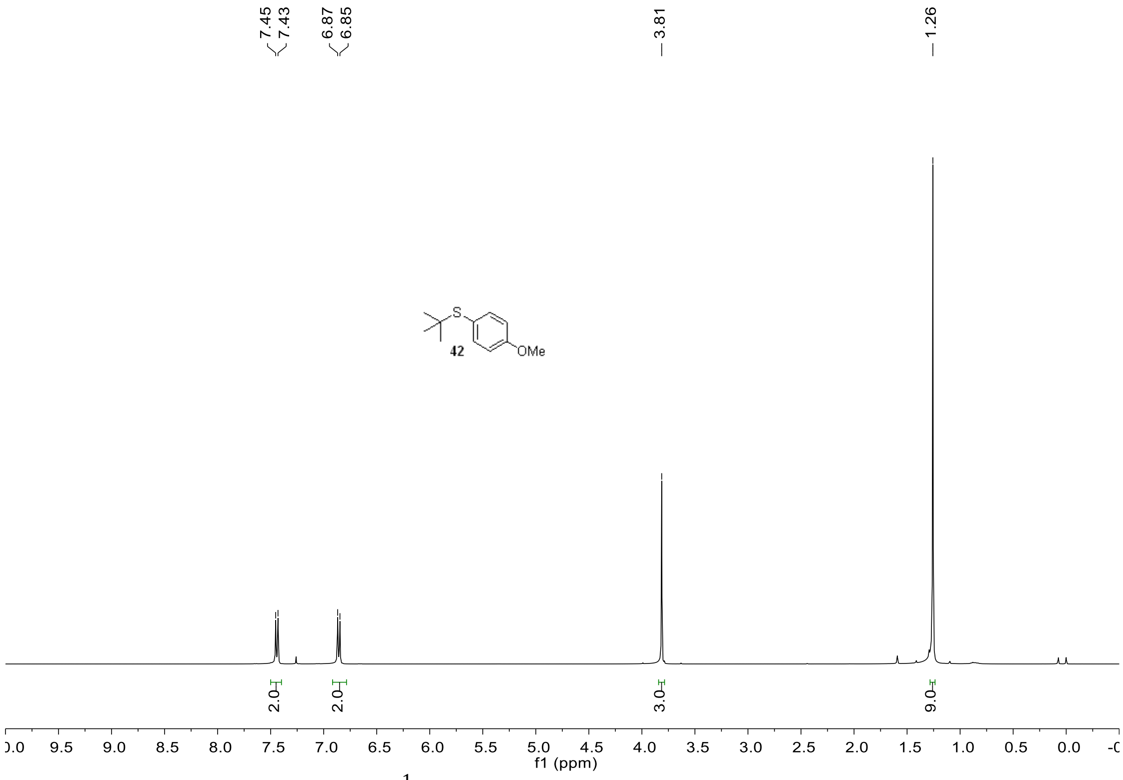

Supplementary Figure 120. ^{1}H NMR Spectra of compound **42**.

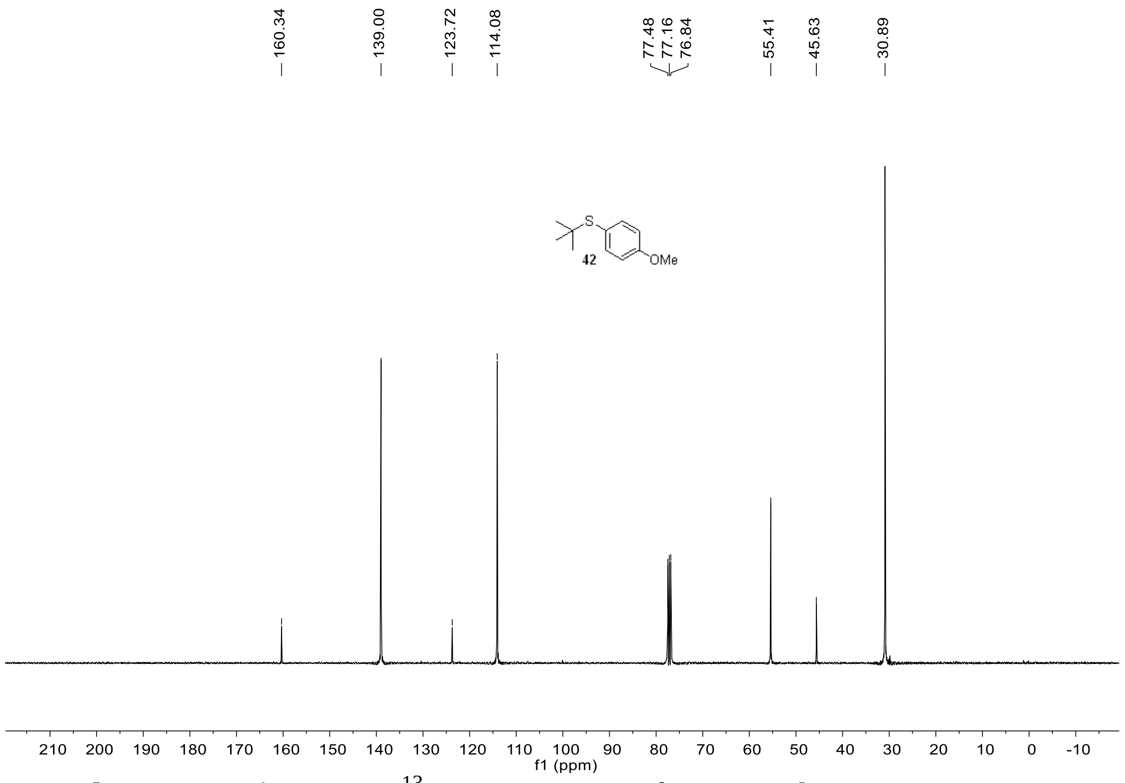

Supplementary Figure 121. ^{13}C NMR Spectra of compound **42**.

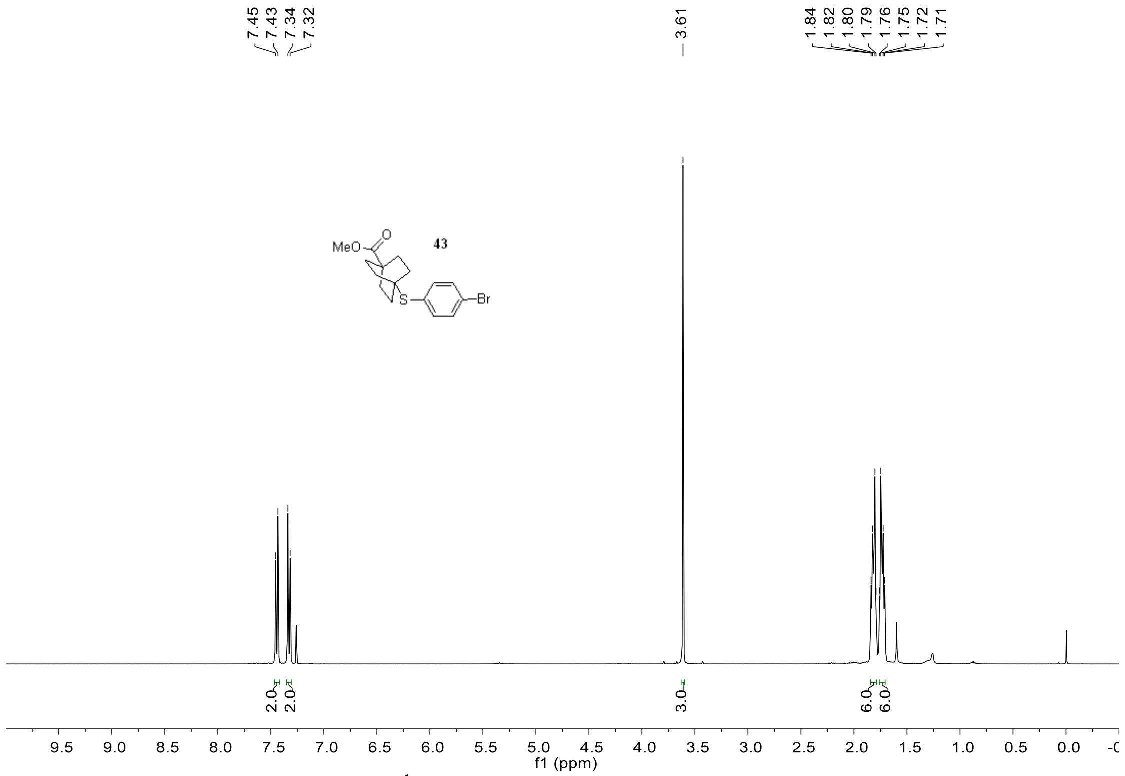

Supplementary Figure 122. ^{1}H NMR Spectra of compound **43**.

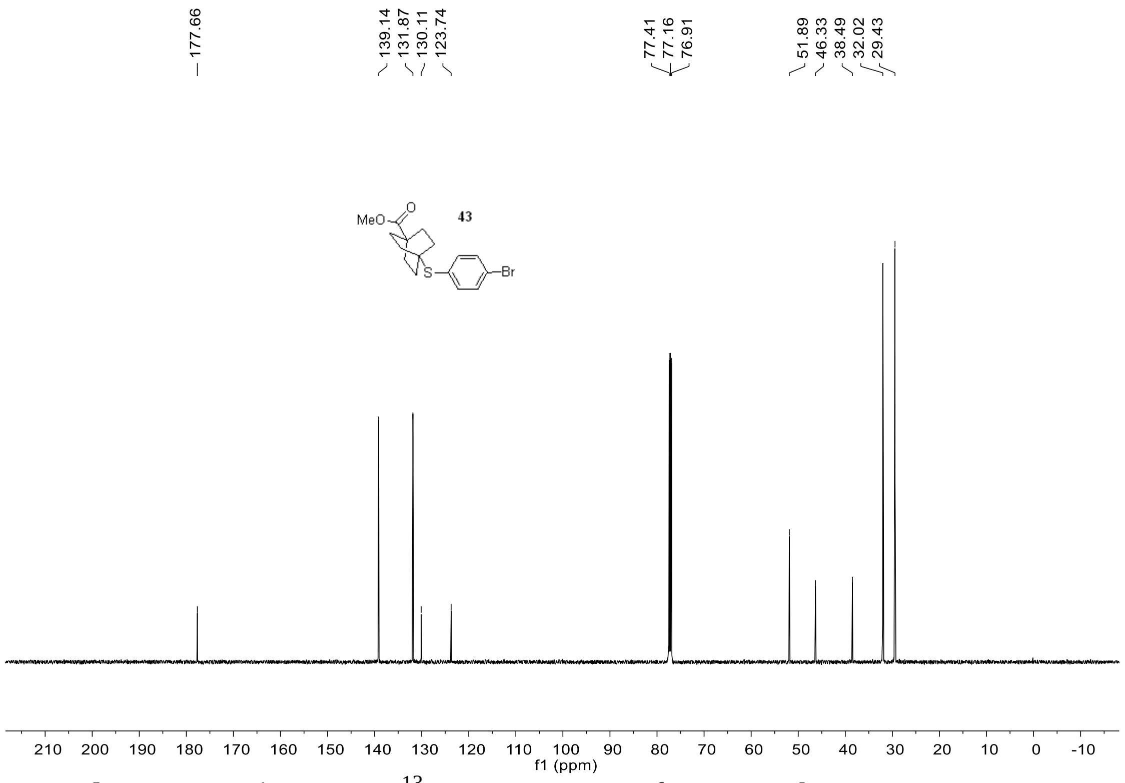

Supplementary Figure 123. ^{13}C NMR Spectra of compound **43**.

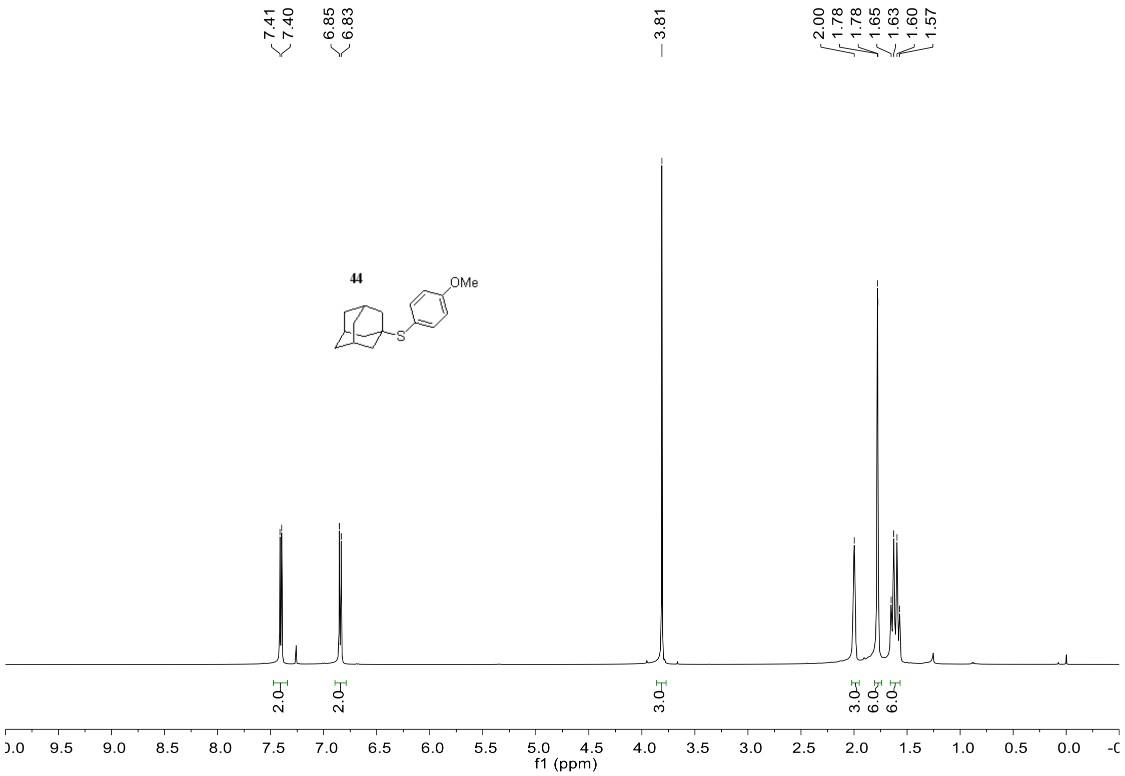

Supplementary Figure 124. ^{1}H NMR Spectra of compound **44**.

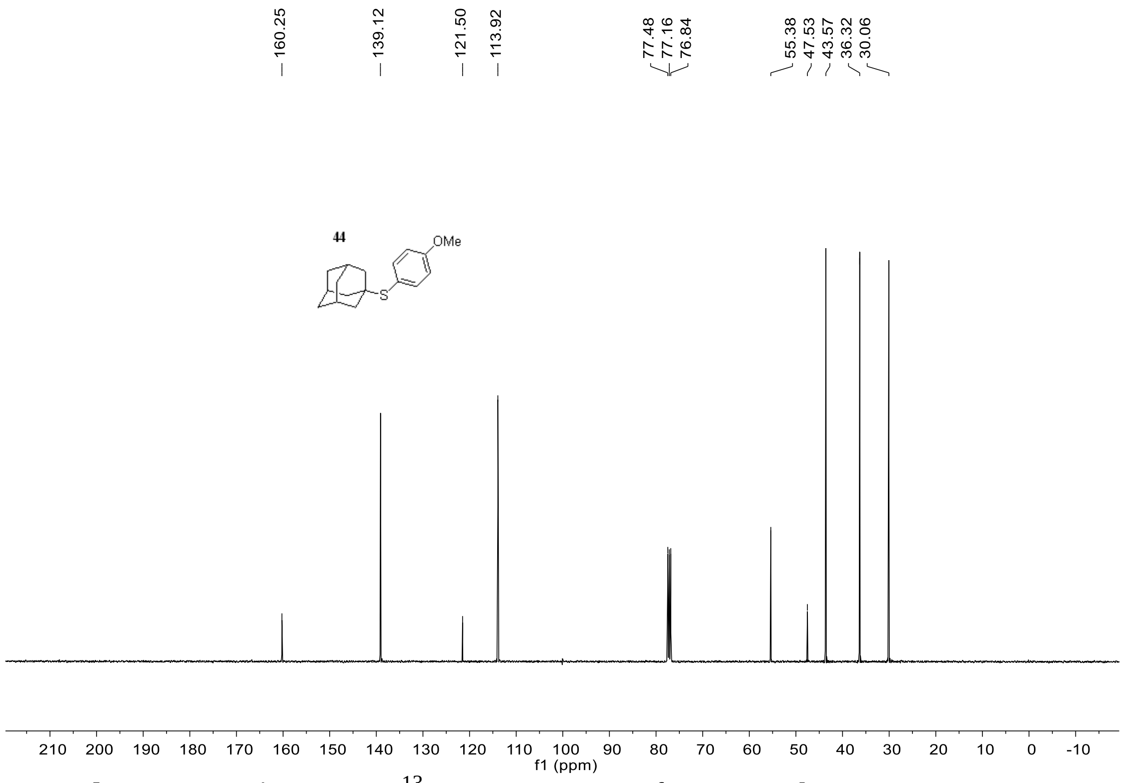

Supplementary Figure 125. ^{13}C NMR Spectra of compound **44**.

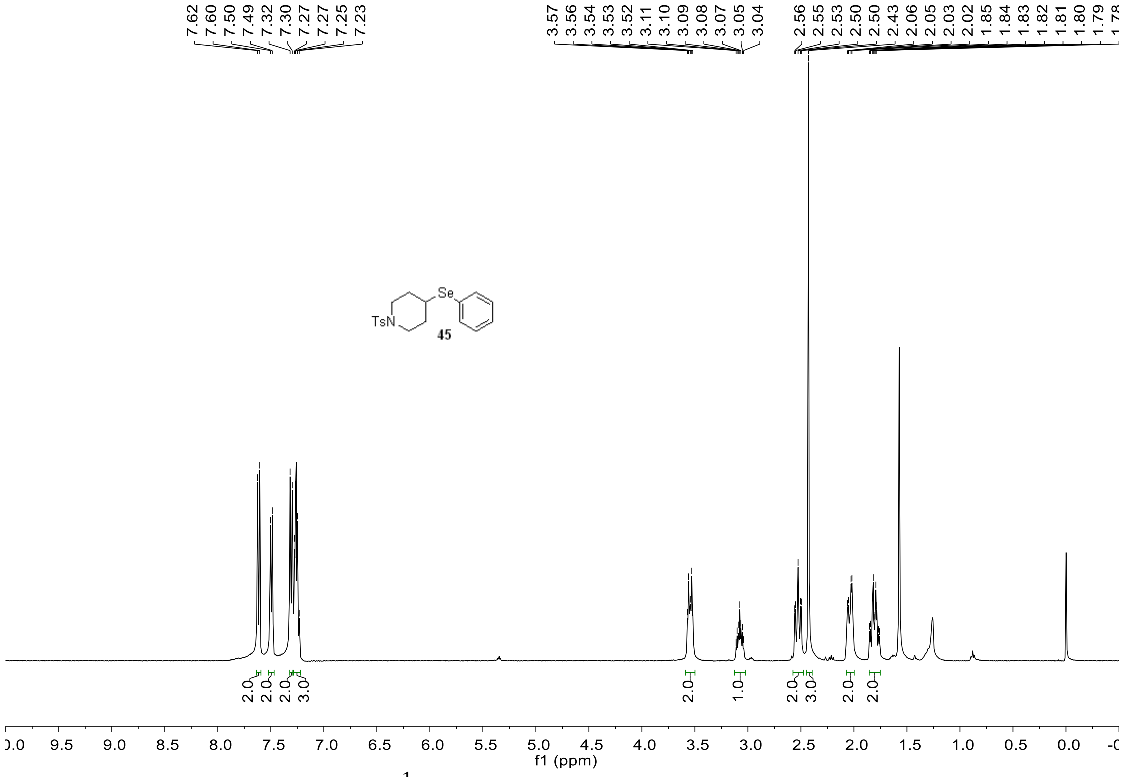

Supplementary Figure 126. ^{1}H NMR Spectra of compound **45**.

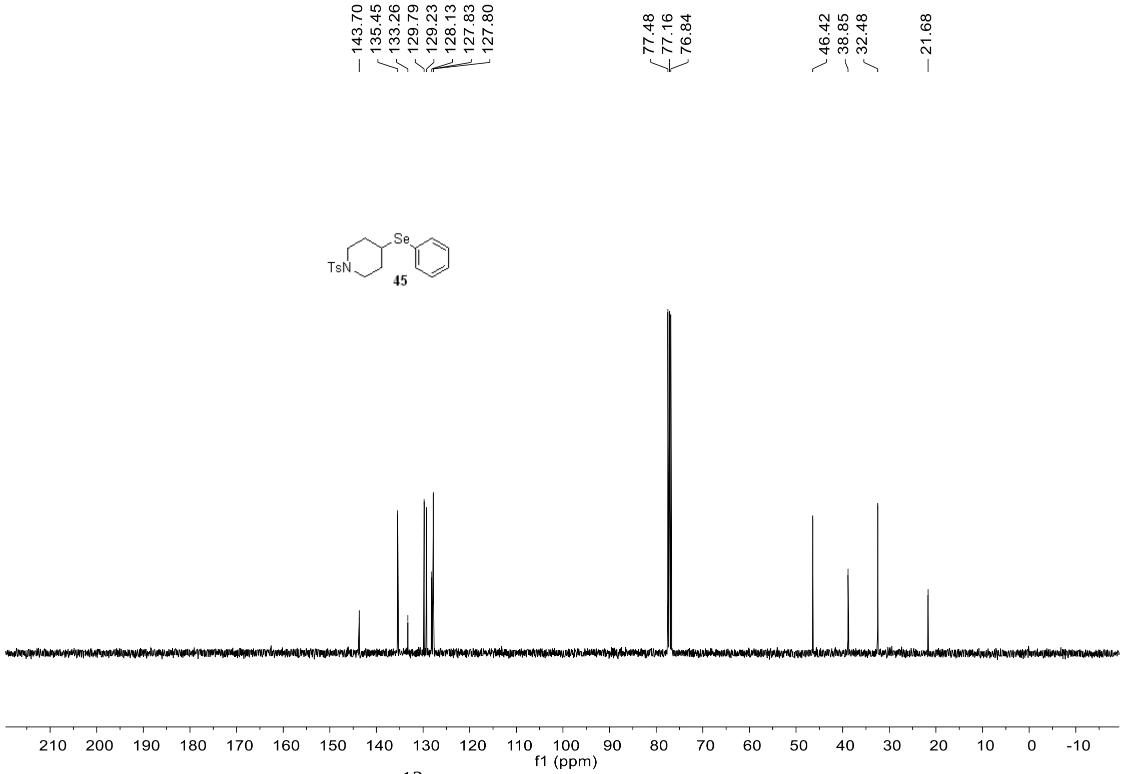

Supplementary Figure 127. ^{13}C NMR Spectra of compound **45**.

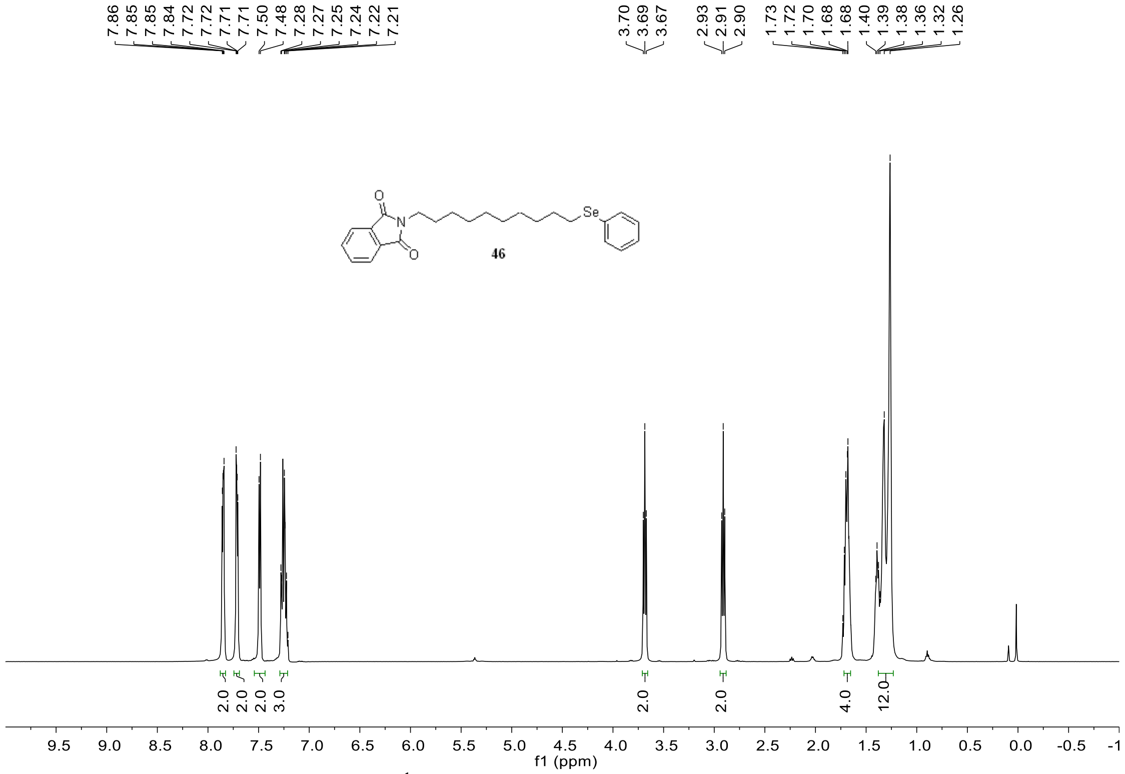

Supplementary Figure 128. ^{1}H NMR Spectra of compound **46**.

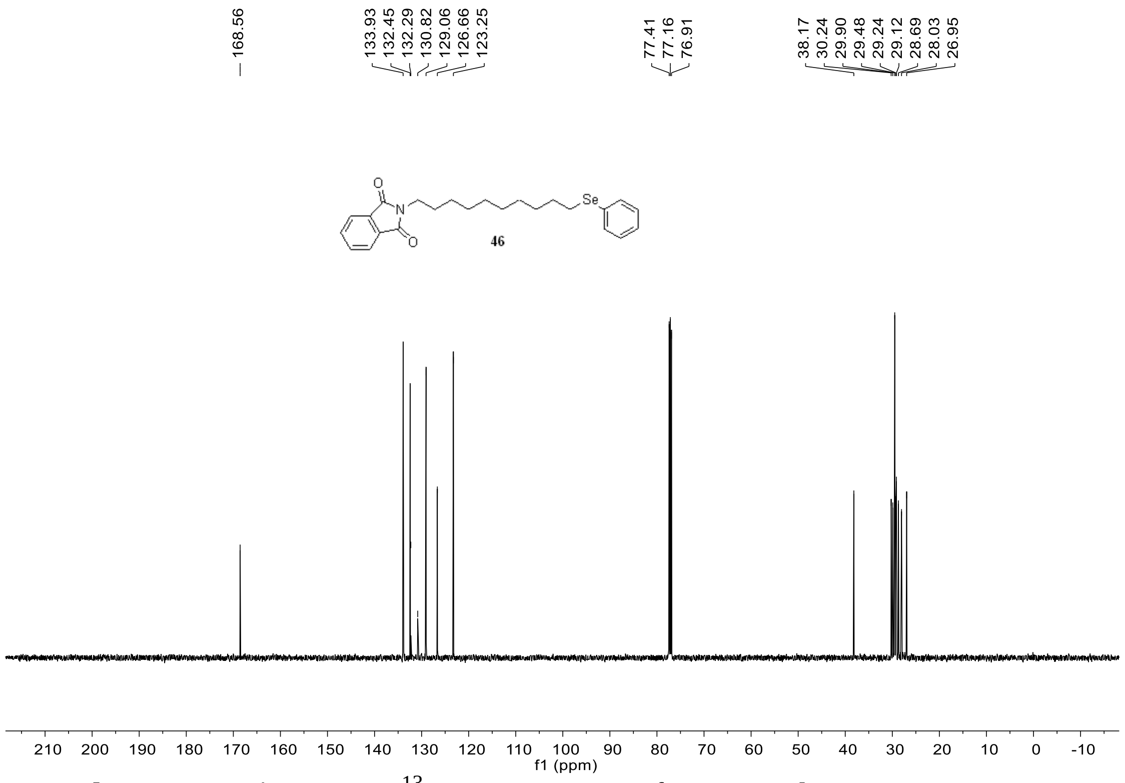

Supplementary Figure 129. ^{13}C NMR Spectra of compound **46**.

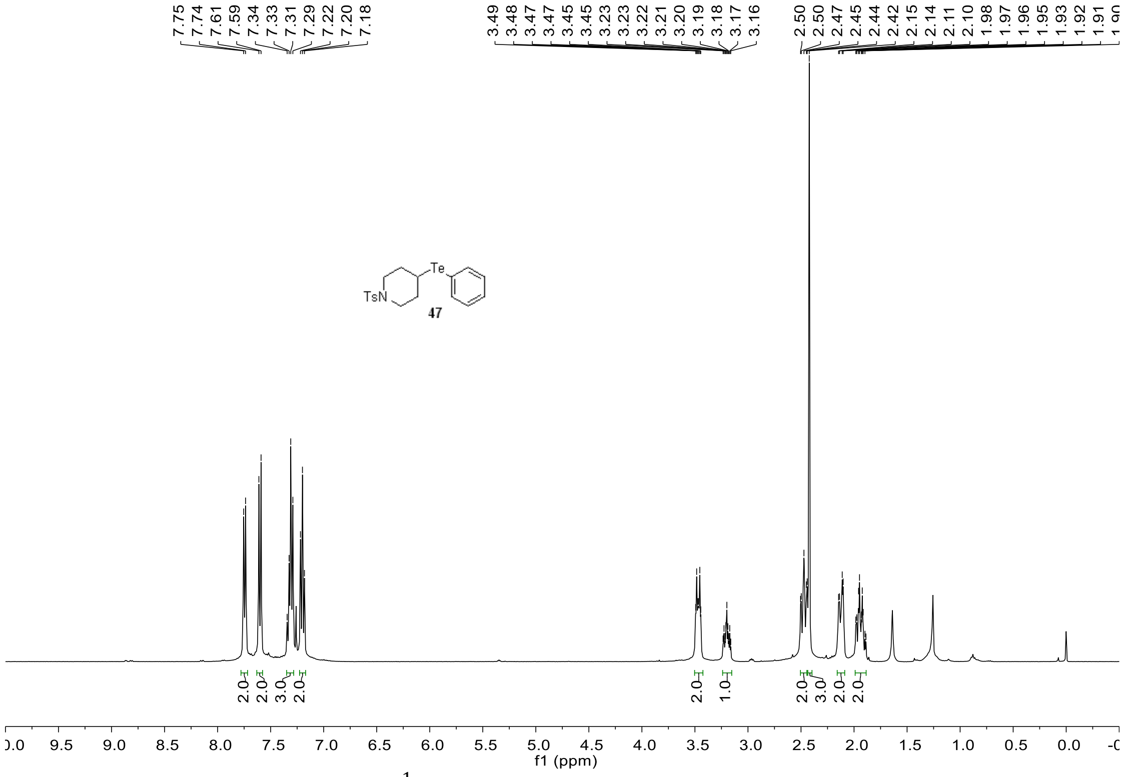

Supplementary Figure 130. ^{1}H NMR Spectra of compound **47**.

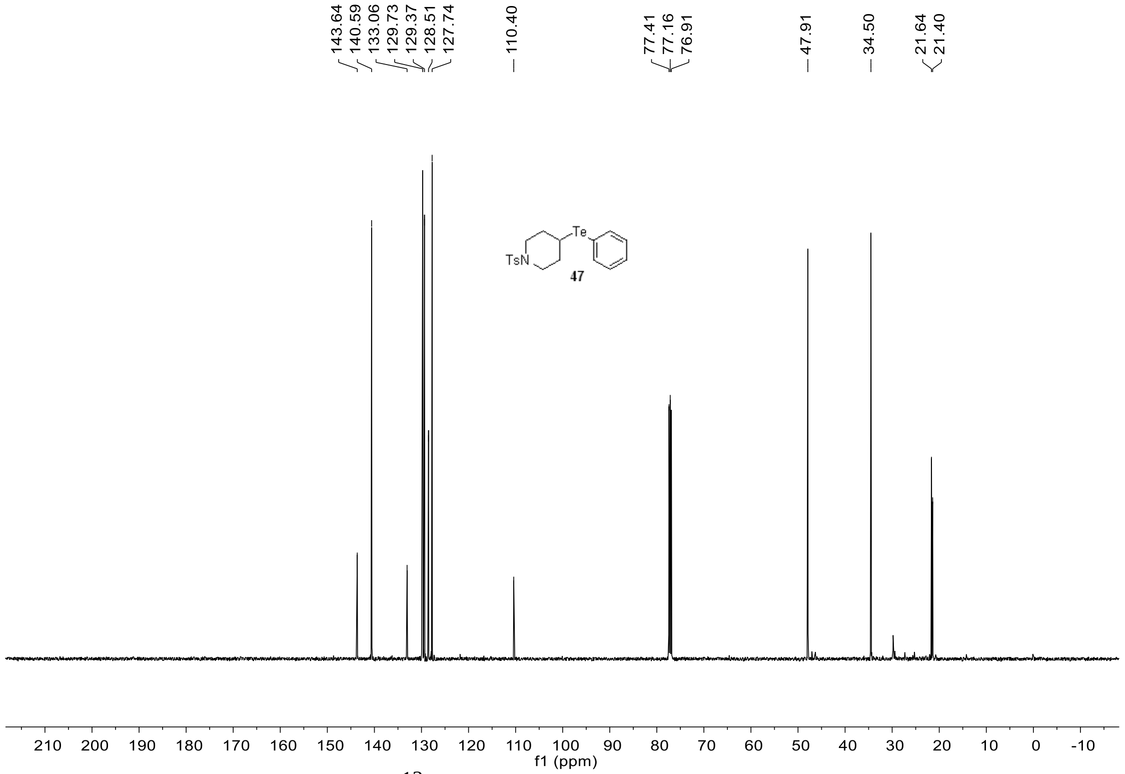

Supplementary Figure 131. ^{13}C NMR Spectra of compound **47**.

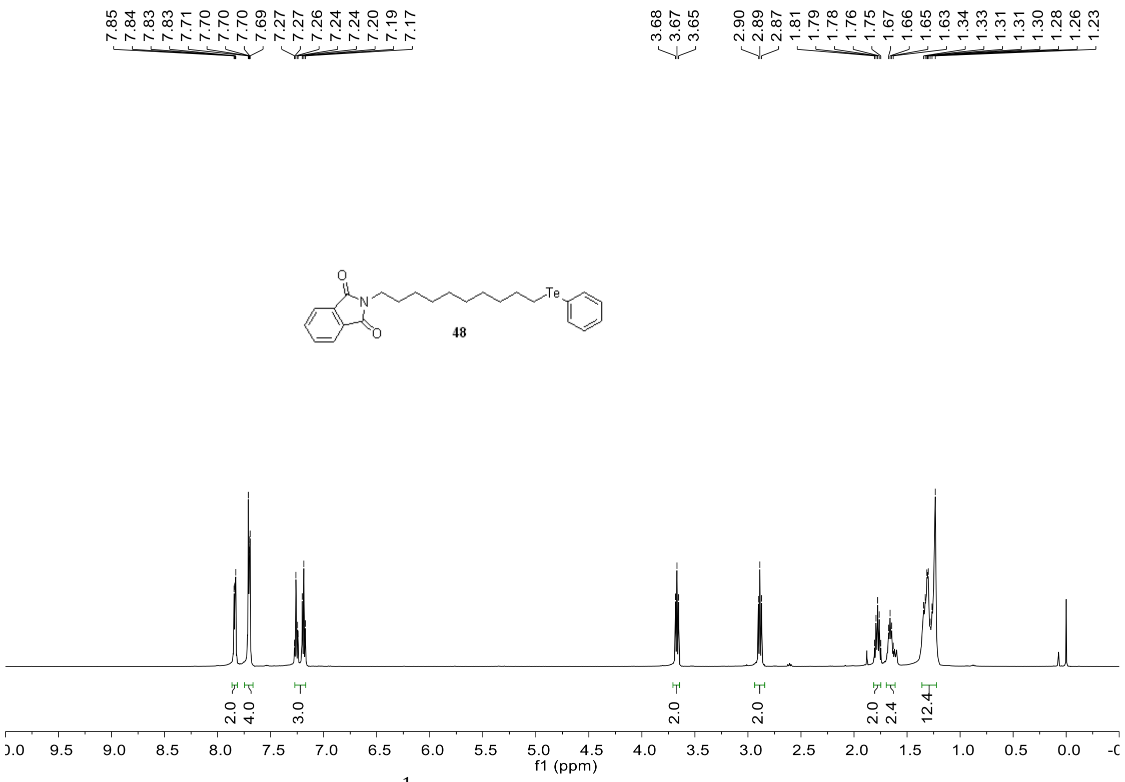

Supplementary Figure 132. ^{1}H NMR Spectra of compound **48**.

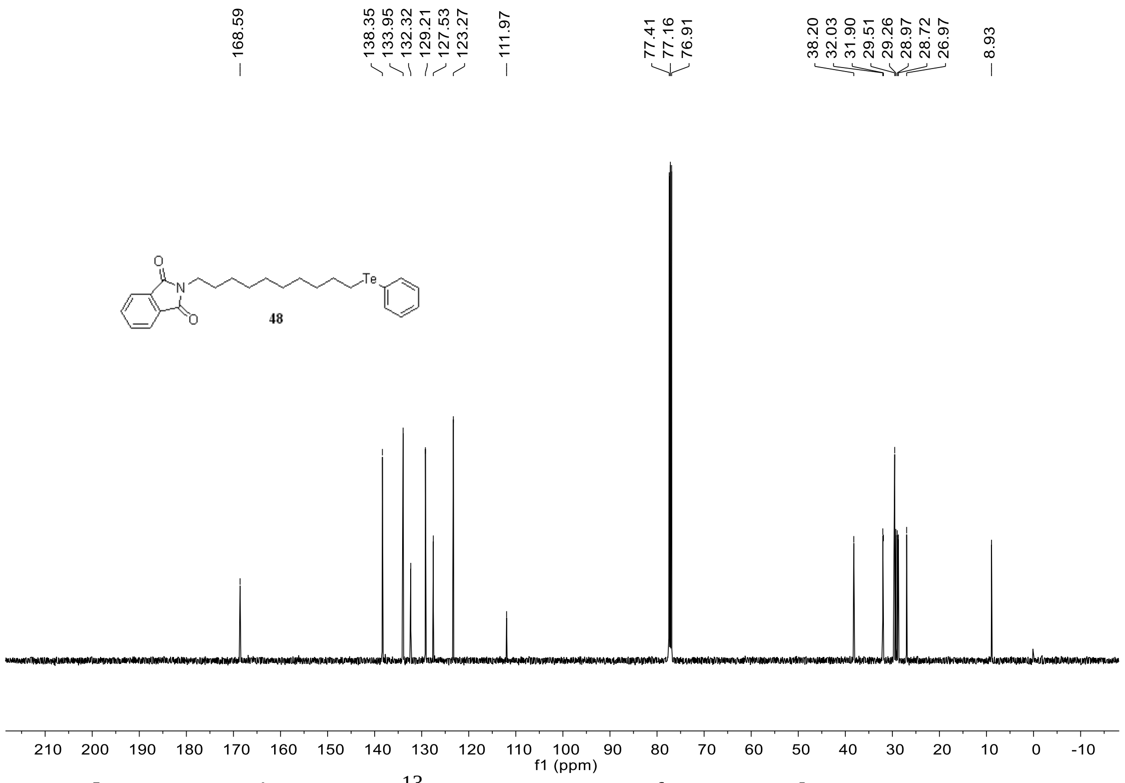

Supplementary Figure 133. ^{13}C NMR Spectra of compound **48**.

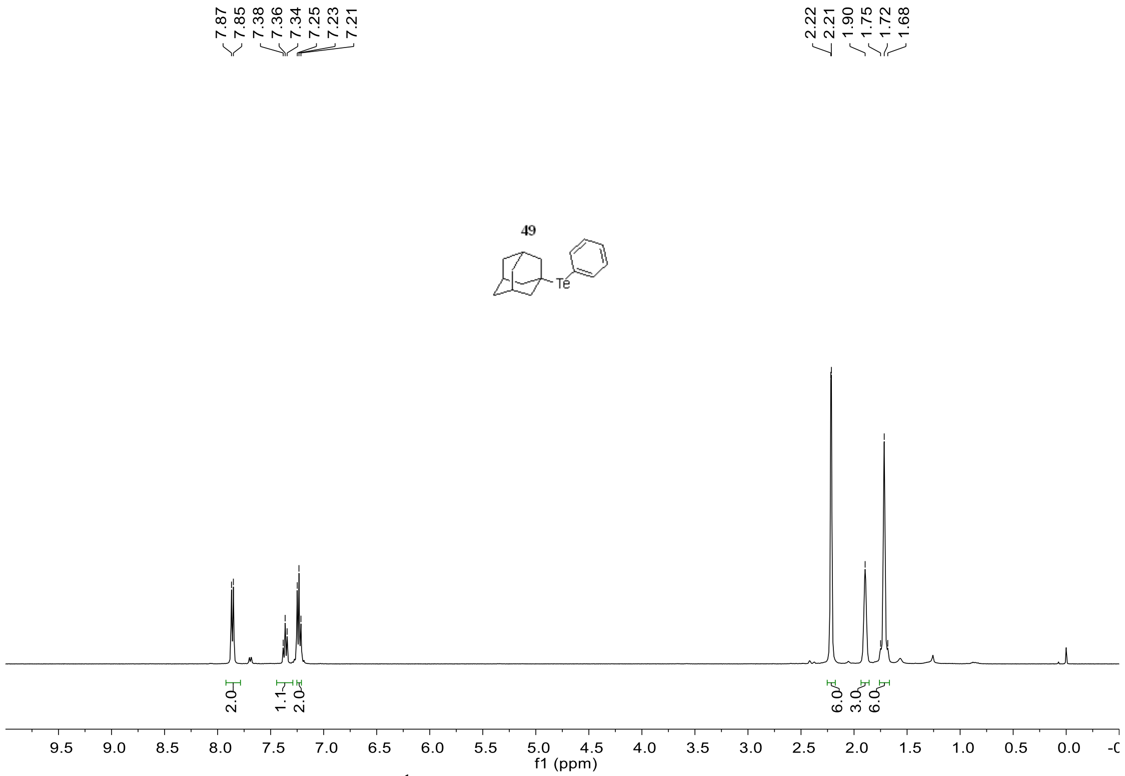

Supplementary Figure 134. ^{1}H NMR Spectra of compound **49**.

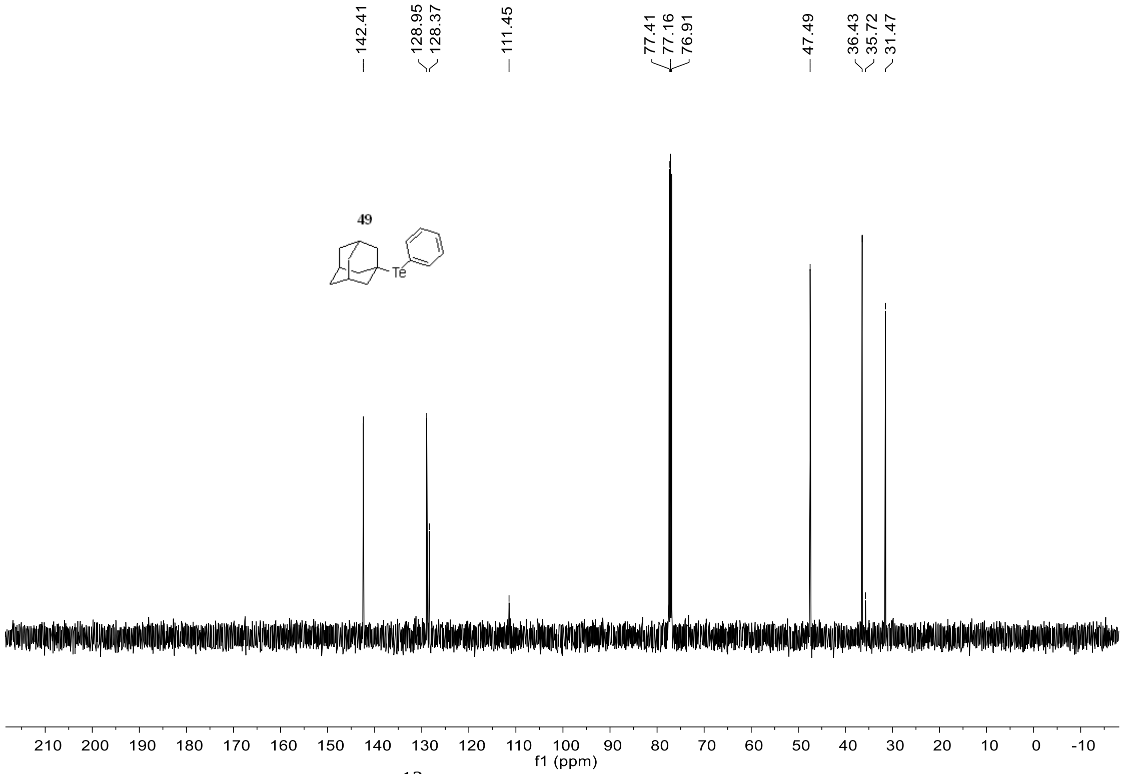

Supplementary Figure 135. ^{13}C NMR Spectra of compound **49**.

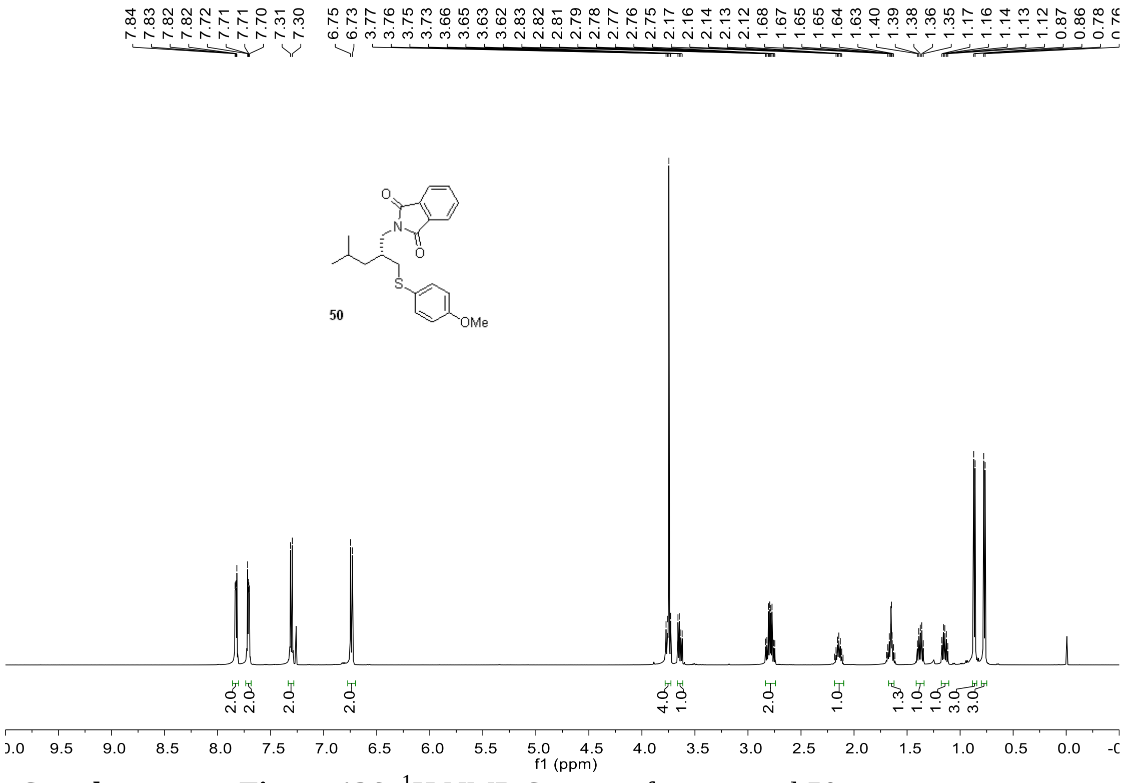

Supplementary Figure 136. ^{1}H NMR Spectra of compound **50**.

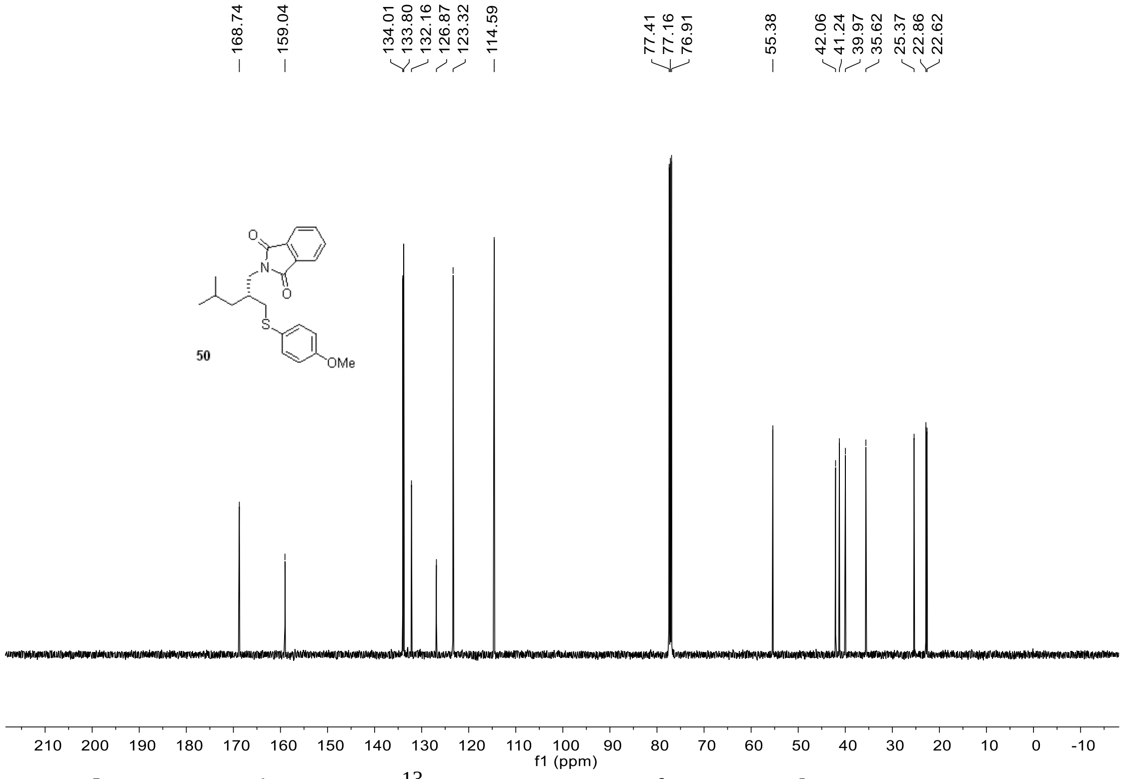

Supplementary Figure 137. ^{13}C NMR Spectra of compound **50**.

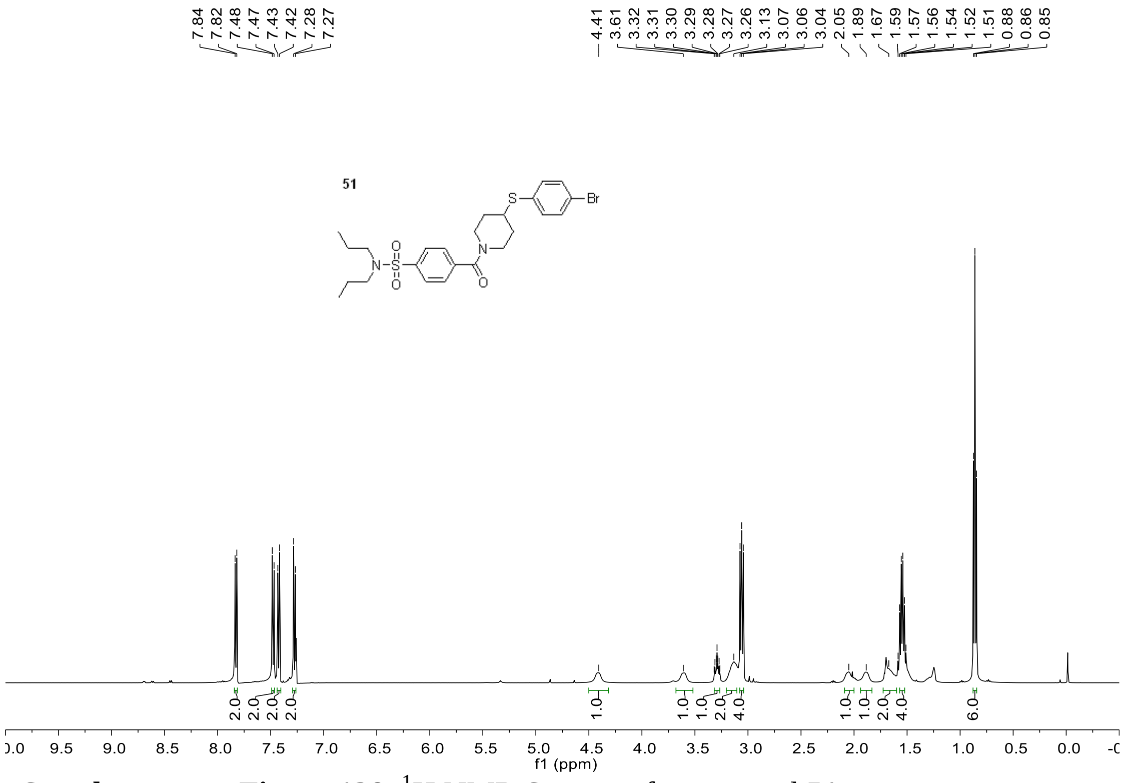

Supplementary Figure 138. ^{1}H NMR Spectra of compound **51**.

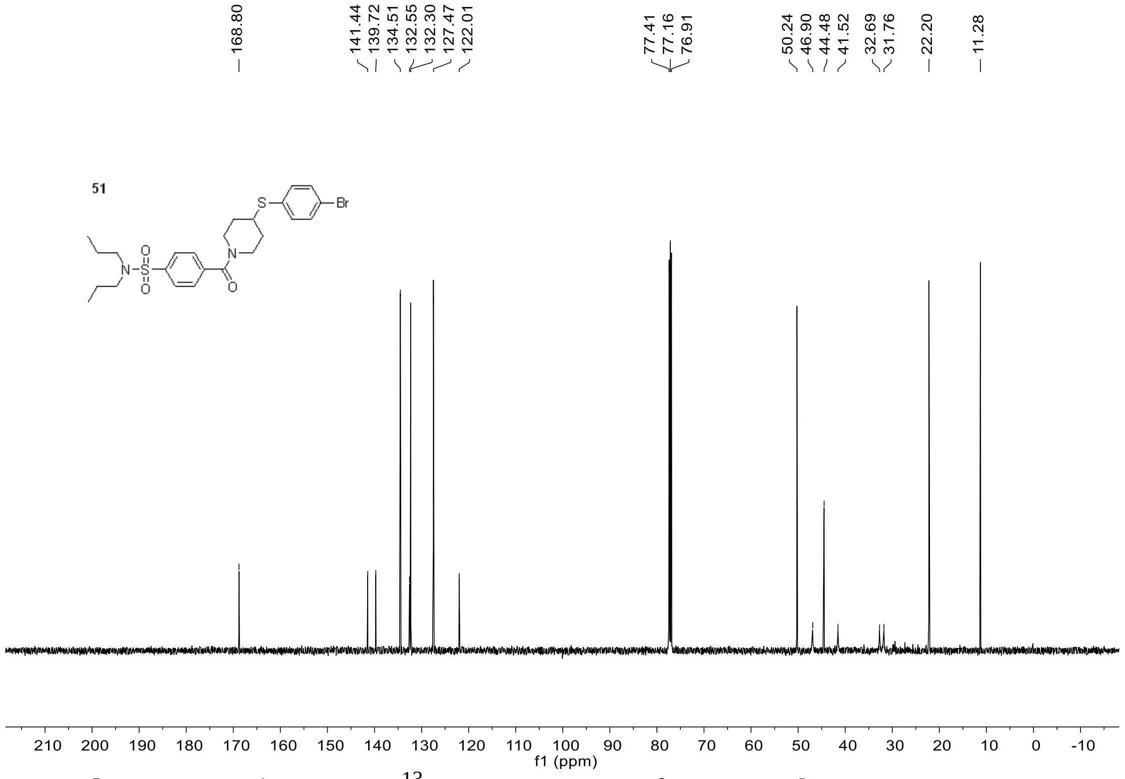

Supplementary Figure 139. ^{13}C NMR Spectra of compound **51**.

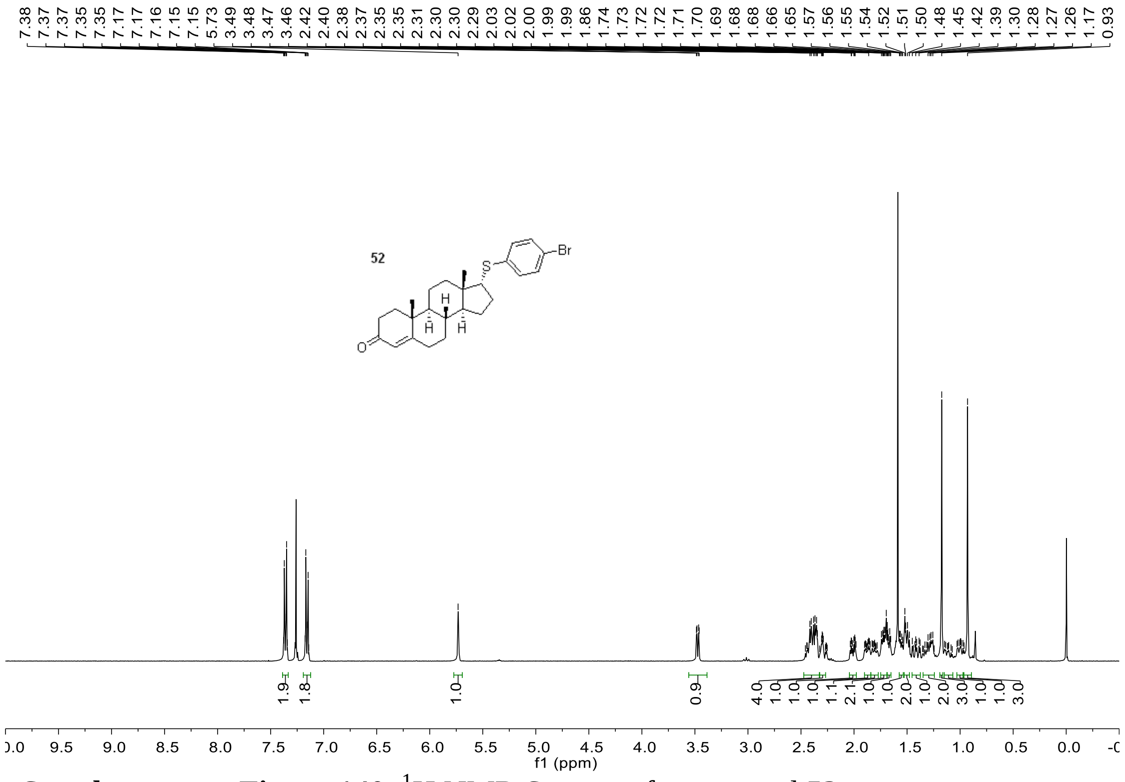

Supplementary Figure 140. ^{1}H NMR Spectra of compound **52**.

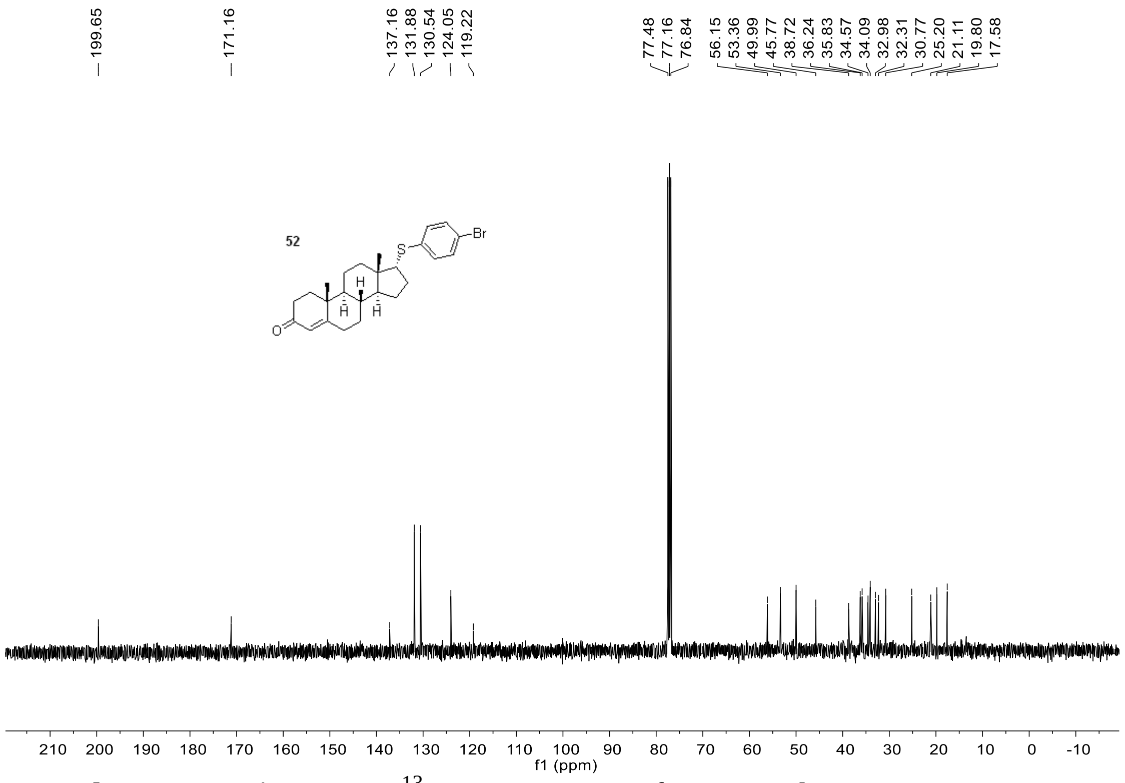

Supplementary Figure 141. ^{13}C NMR Spectra of compound **52**.

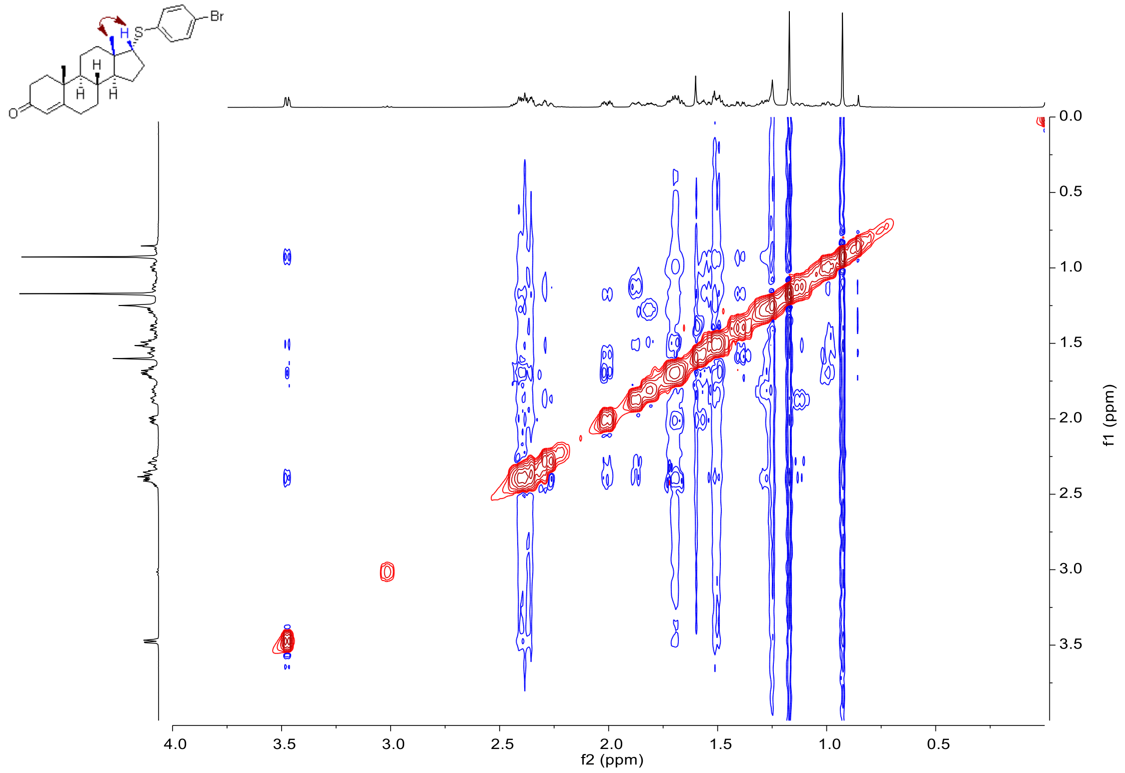

Supplementary Figure 142. NOESY NMR Spectra of compound **52**.

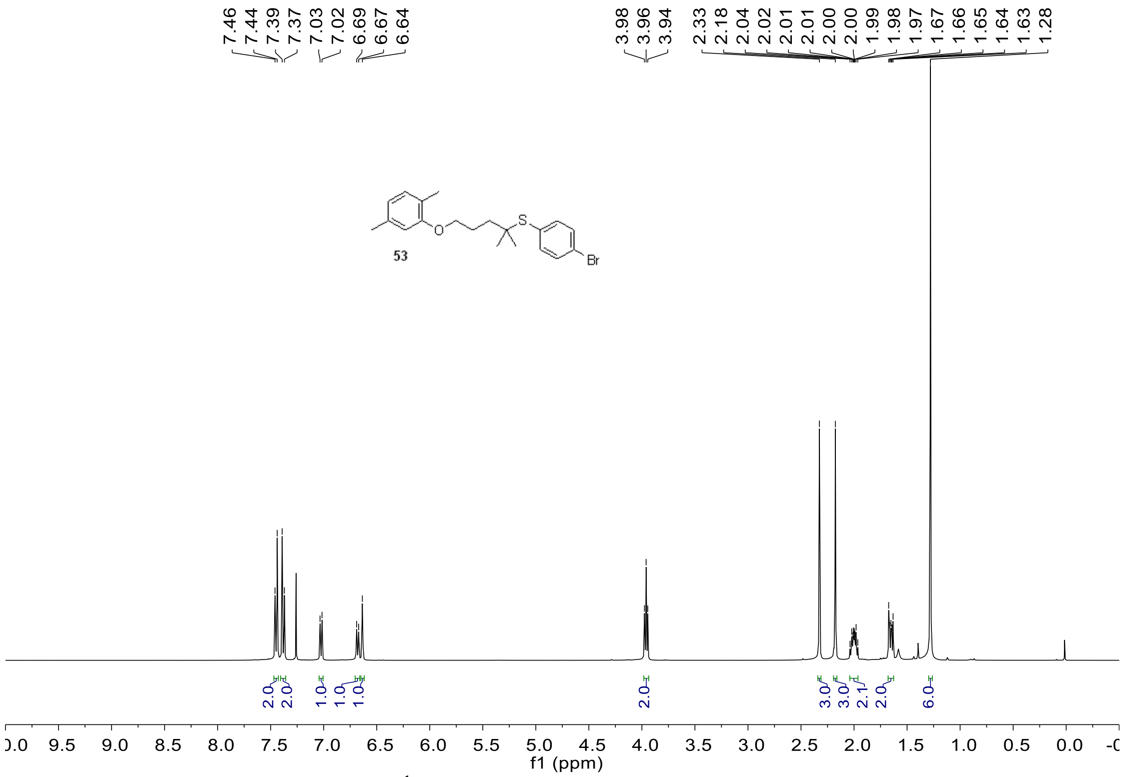

Supplementary Figure 143. ^{1}H NMR Spectra of compound **53**.

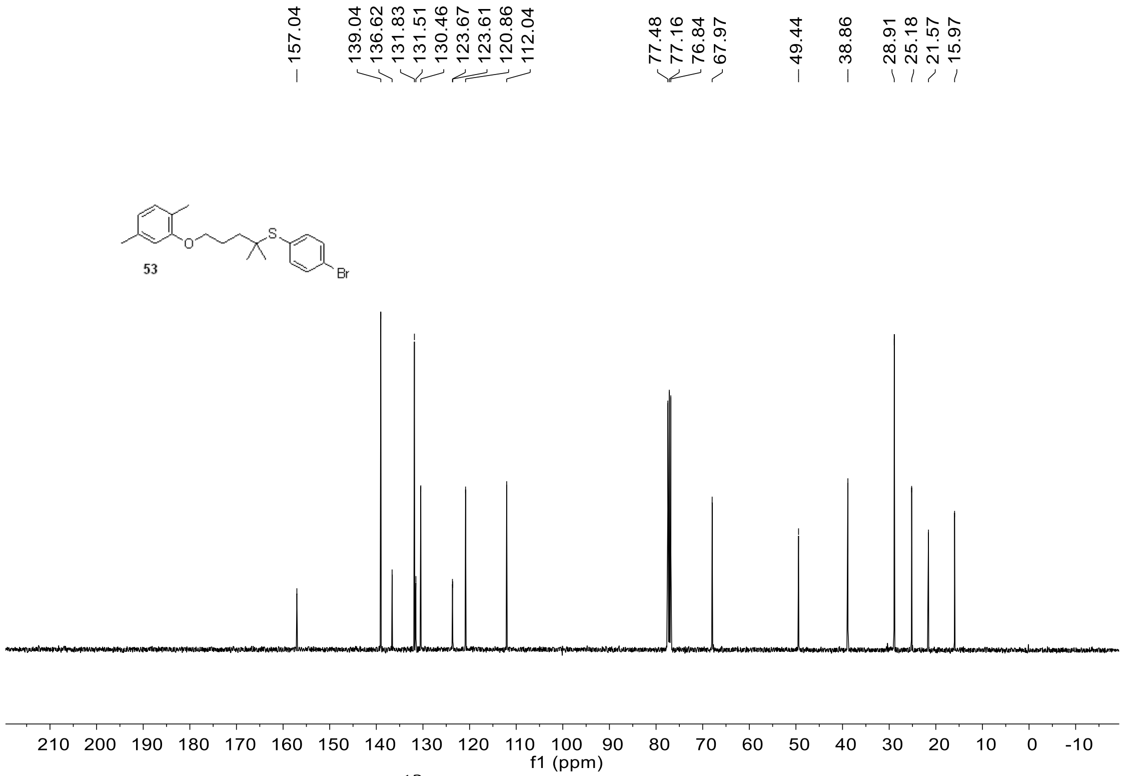

Supplementary Figure 144. ^{13}C NMR Spectra of compound **53**.

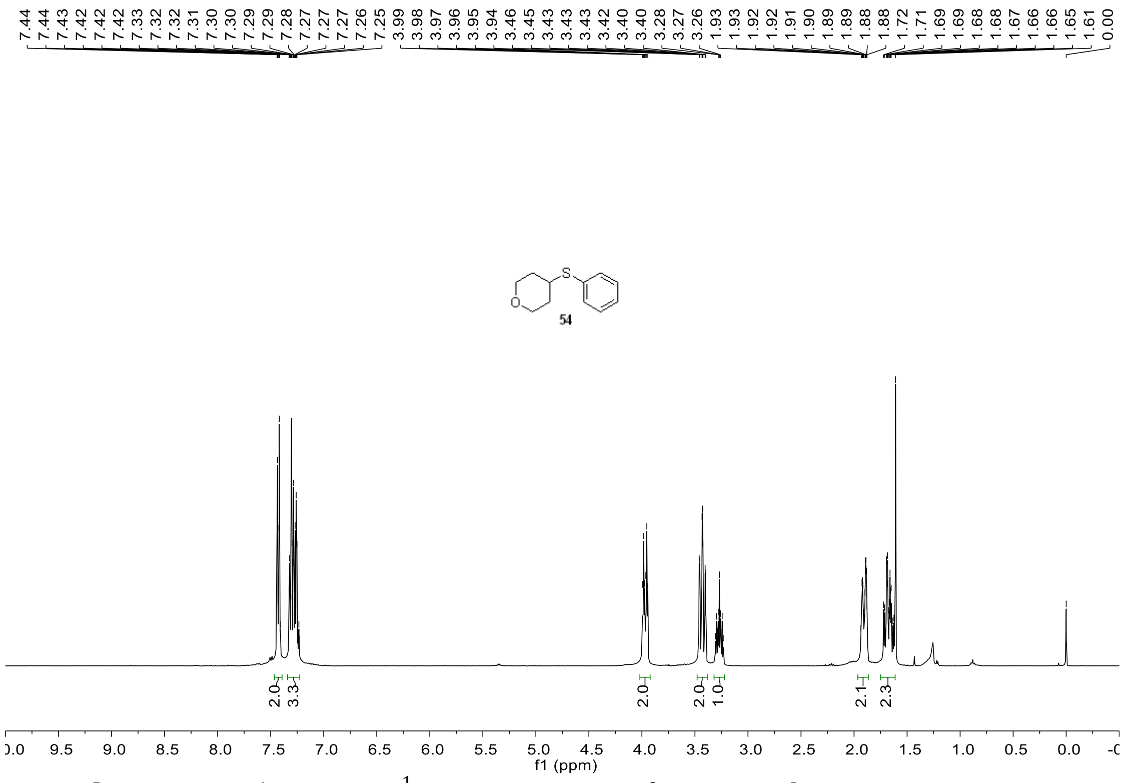

Supplementary Figure 145. ^{1}H NMR Spectra of compound **55**.

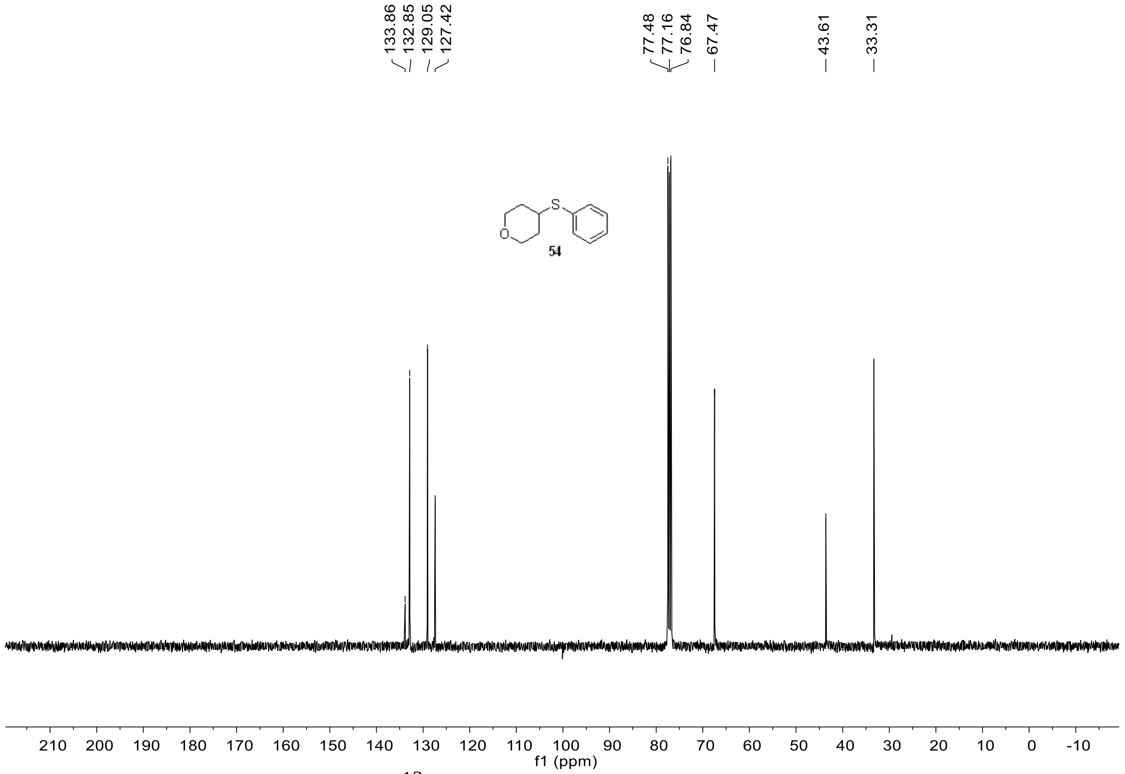

Supplementary Figure 146. ^{13}C NMR Spectra of compound **54**.

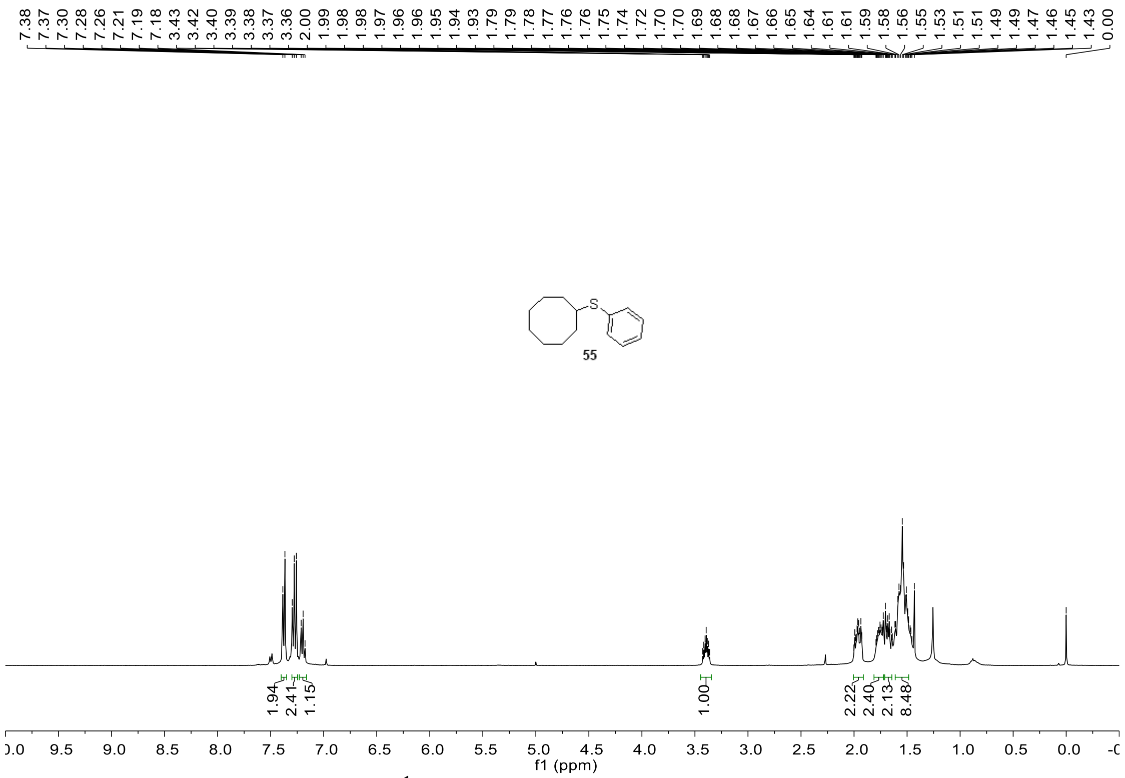

Supplementary Figure 147. ^{1}H NMR Spectra of compound **55**.

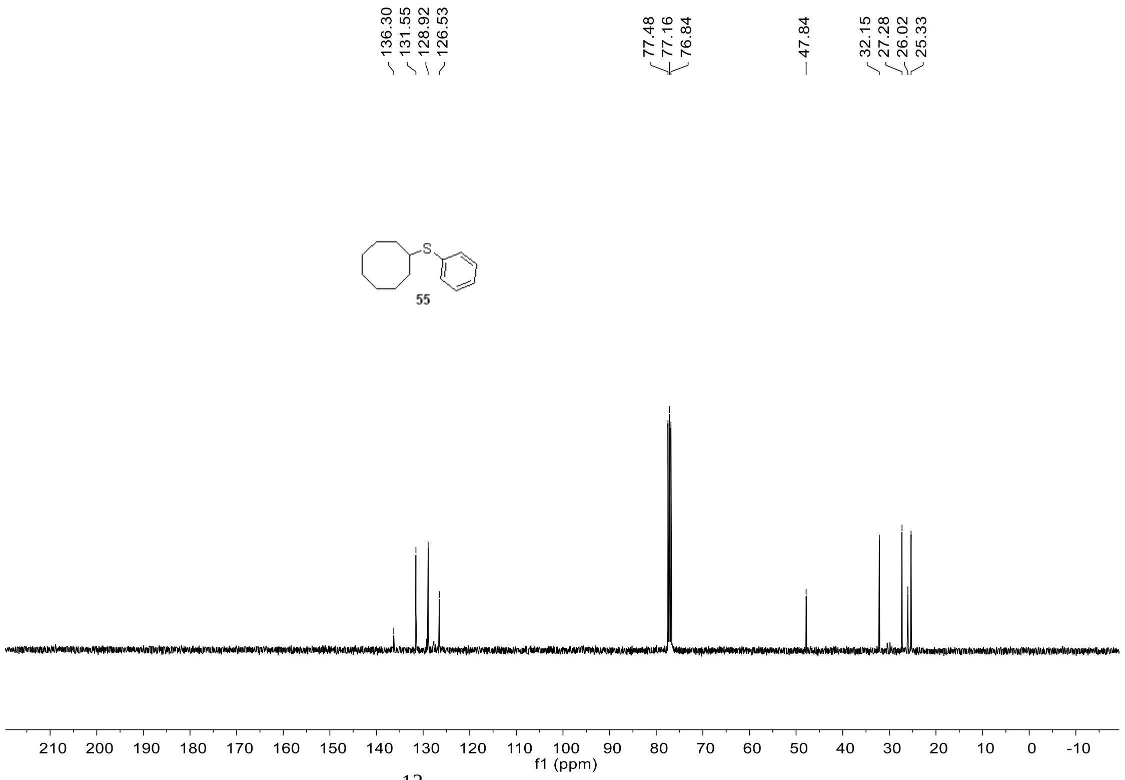

Supplementary Figure 148. ^{13}C NMR Spectra of compound **55**.

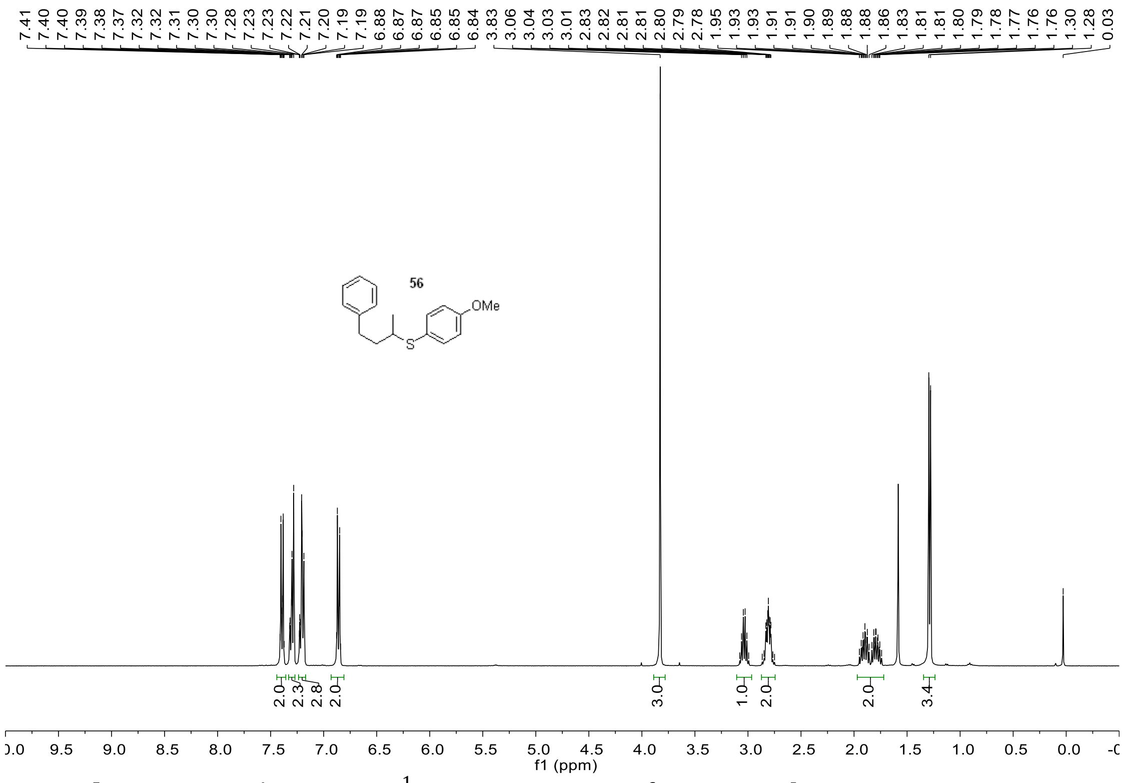

Supplementary Figure 149. ^{1}H NMR Spectra of compound **56**.

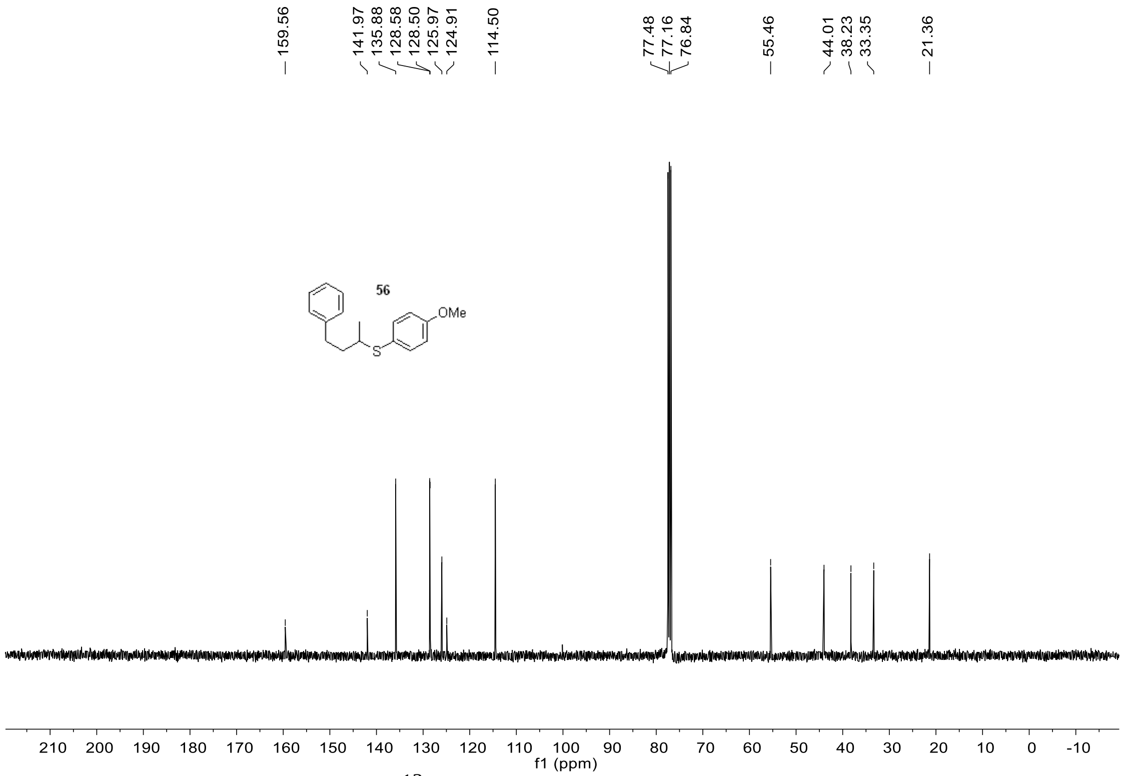

Supplementary Figure 150. ^{13}C NMR Spectra of compound **56**.

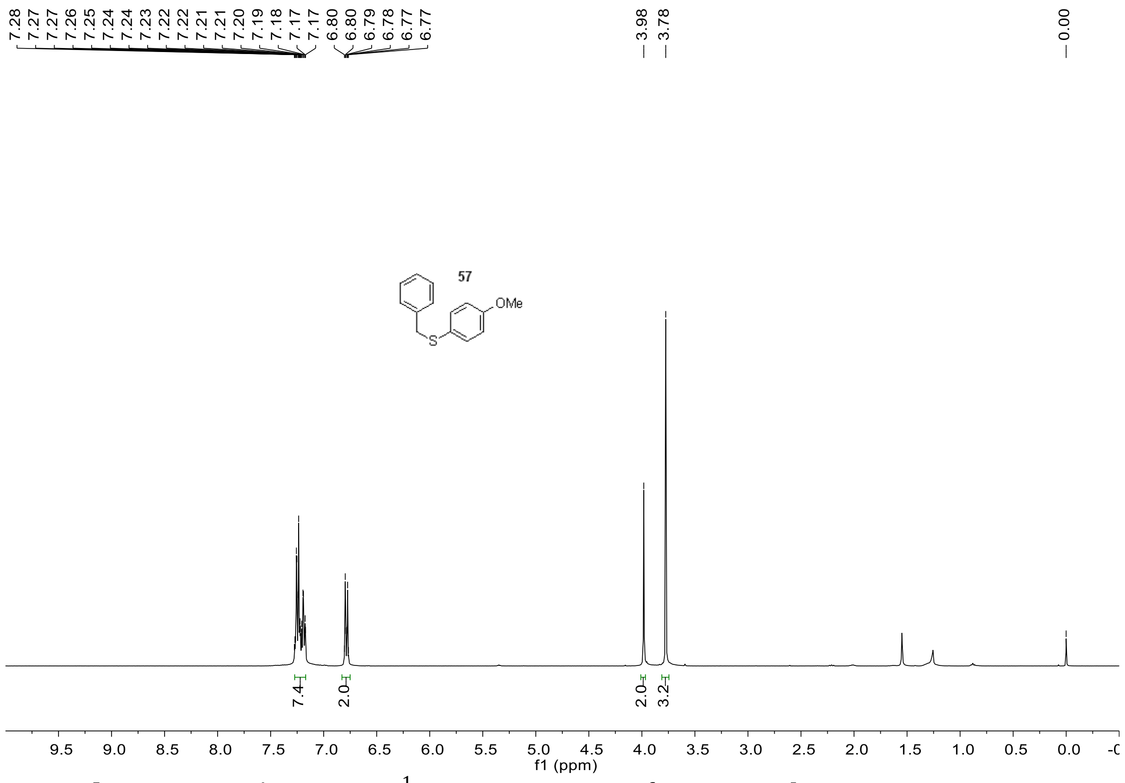

Supplementary Figure 151. ^{1}H NMR Spectra of compound **57**.

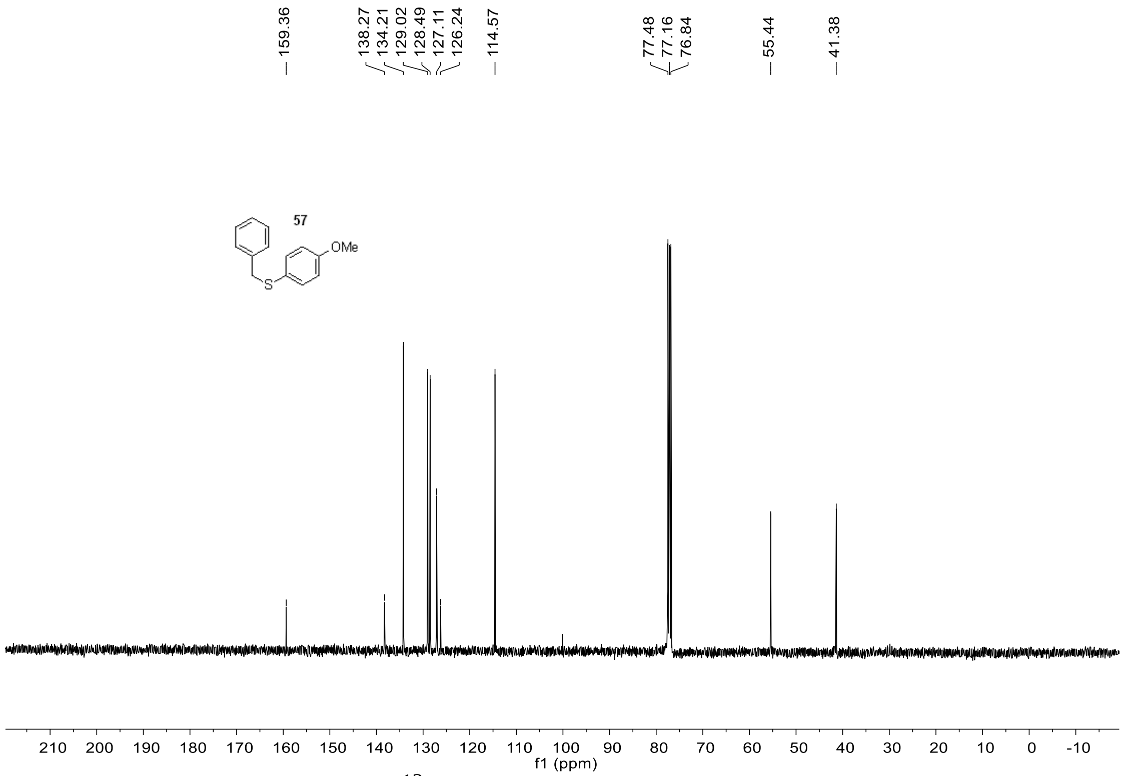

Supplementary Figure 152. ^{13}C NMR Spectra of compound **57**.

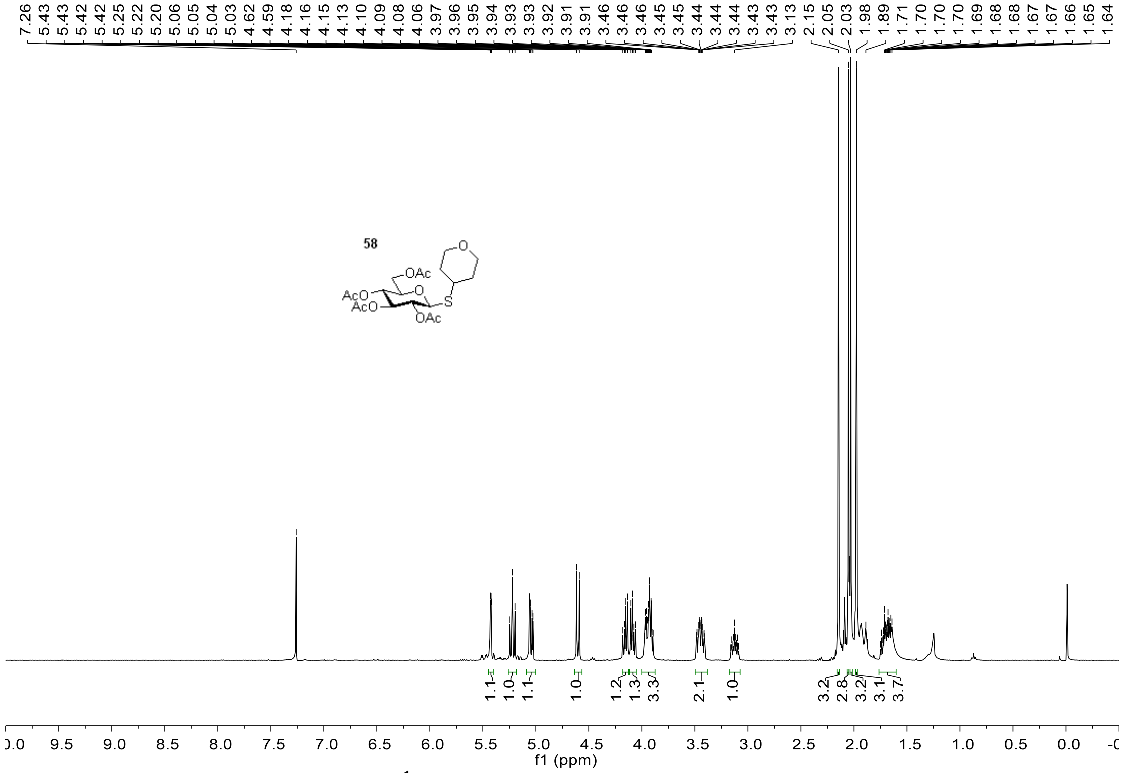

Supplementary Figure 153. ^{1}H NMR Spectra of compound **58**.

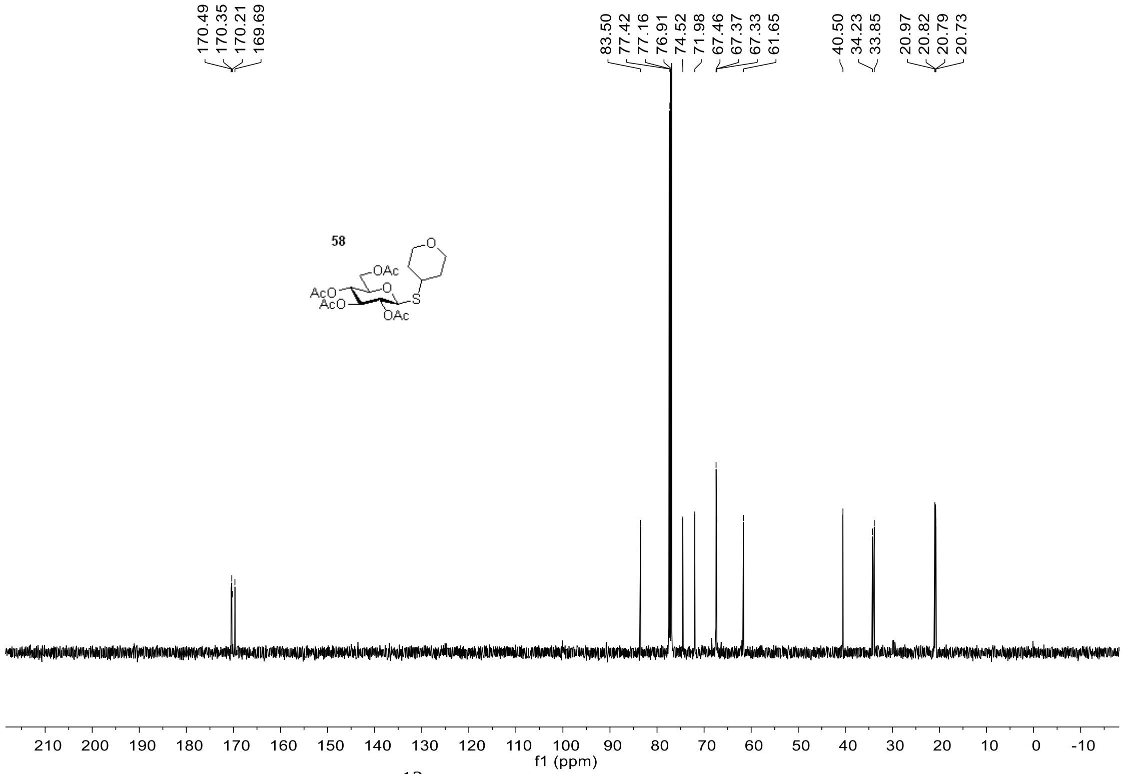

Supplementary Figure 154. ^{13}C NMR Spectra of compound **58**.

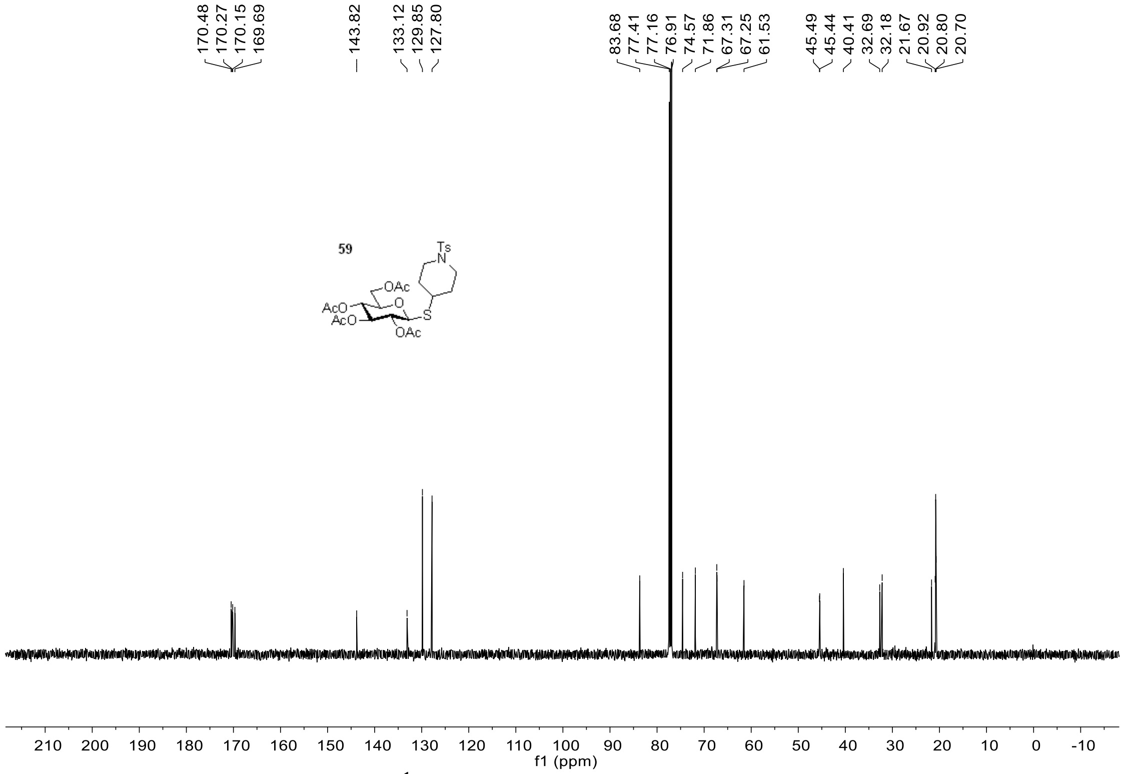

Supplementary Figure 155. ^{1}H NMR Spectra of compound **59**.

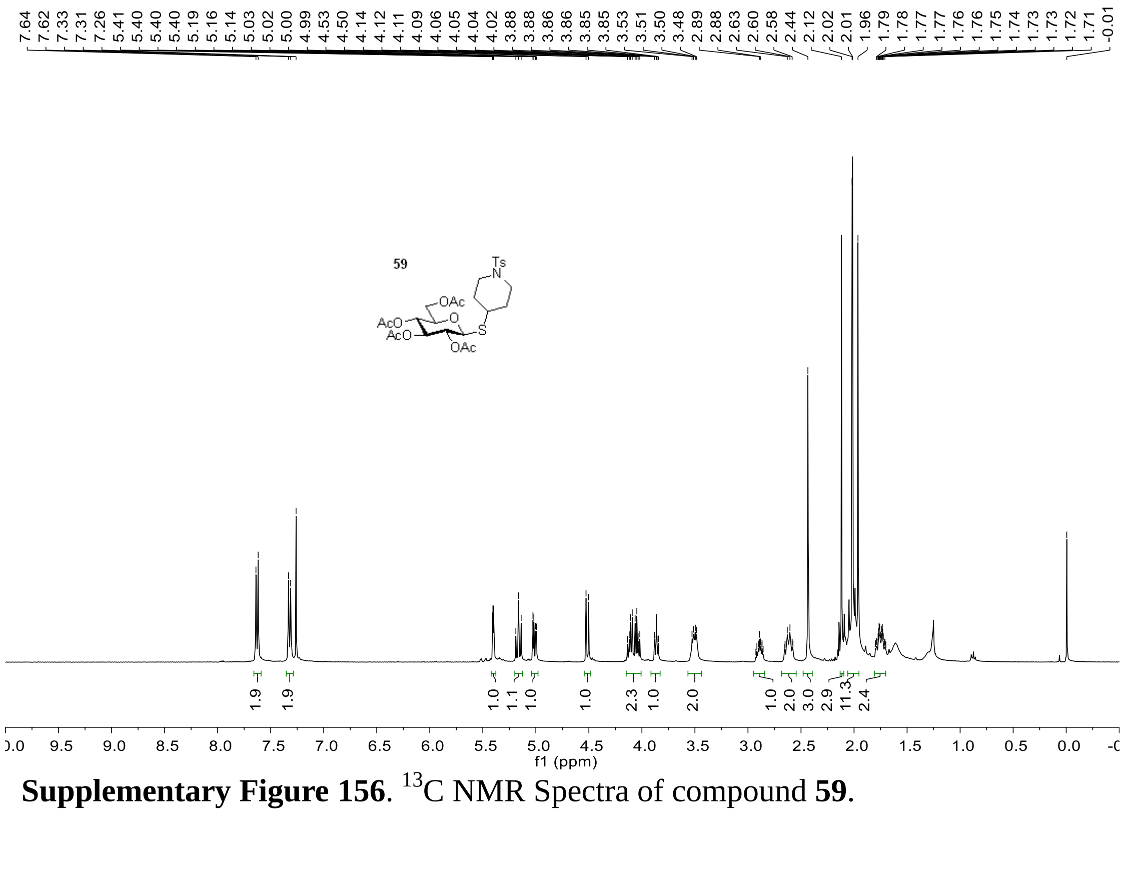

Supplementary Figure 156. ^{13}C NMR Spectra of compound **59**.

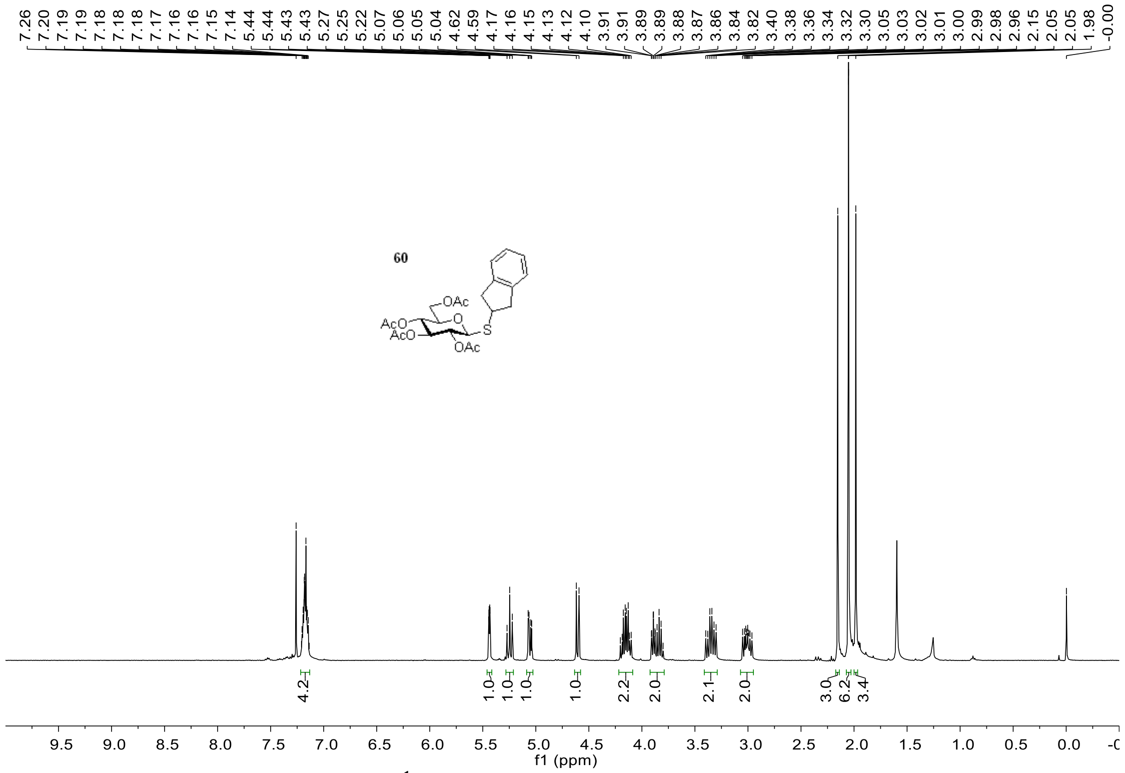

Supplementary Figure 157. ^{1}H NMR Spectra of compound **60**.

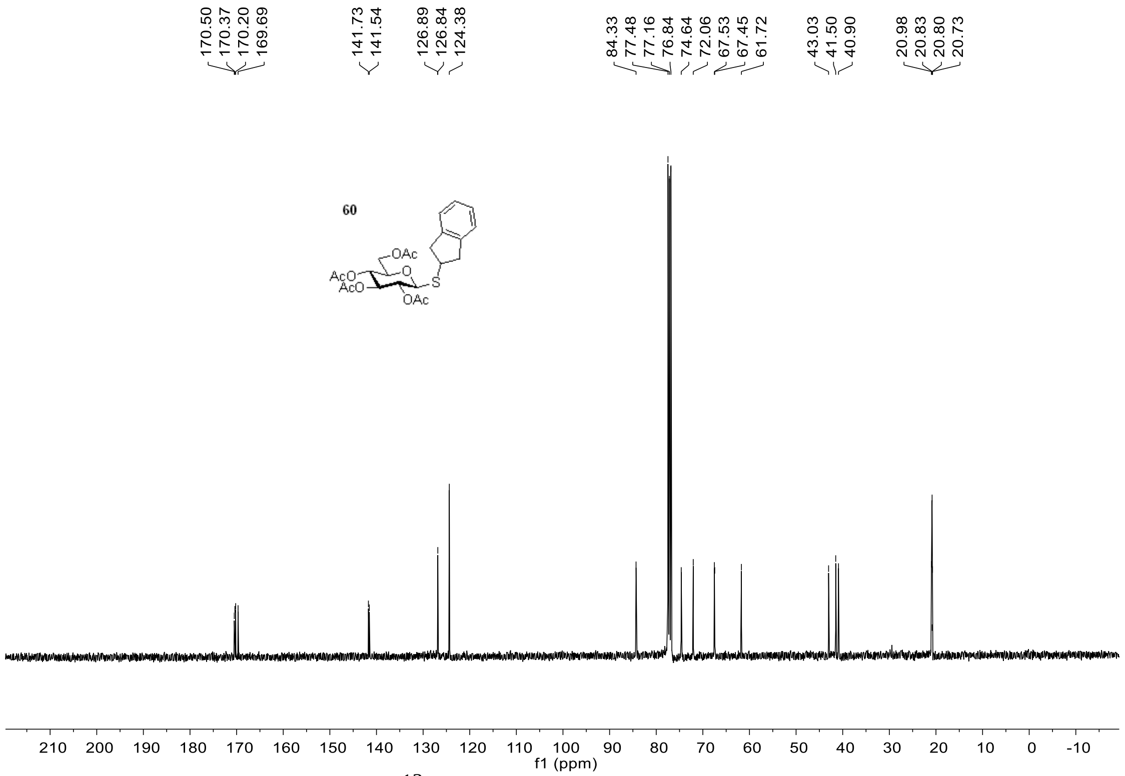

Supplementary Figure 158. ^{13}C NMR Spectra of compound **60**.

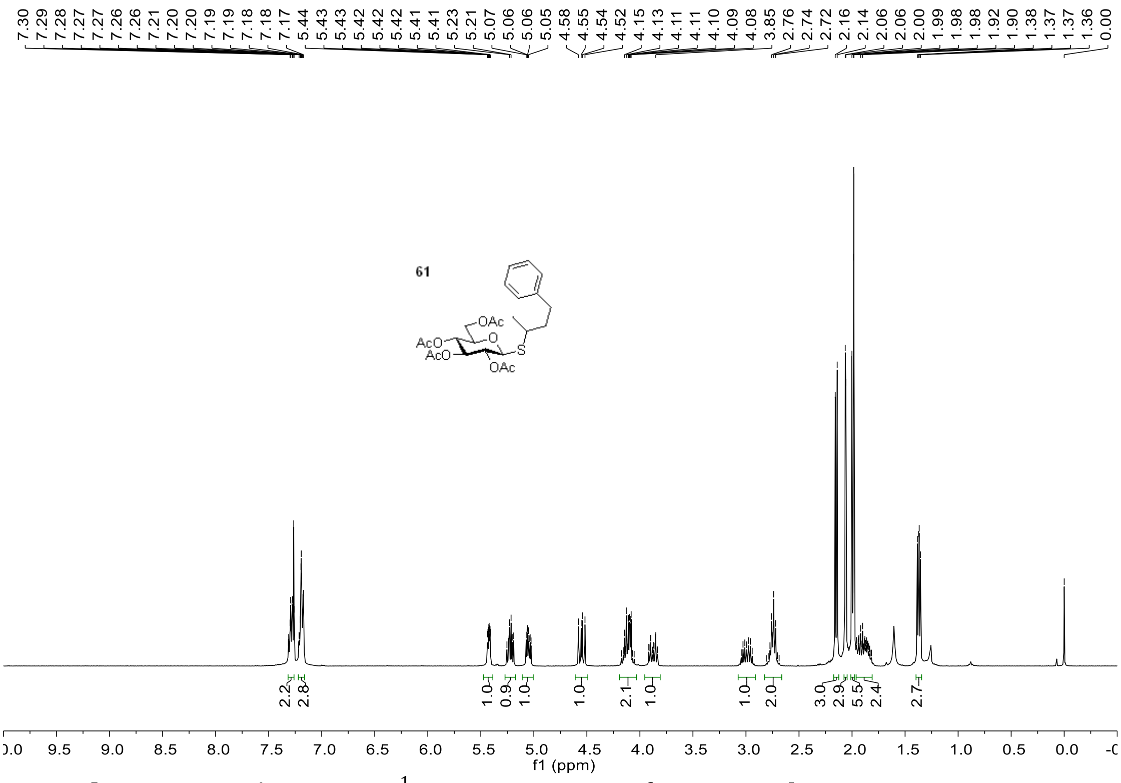

Supplementary Figure 159. ^{1}H NMR Spectra of compound **61**.

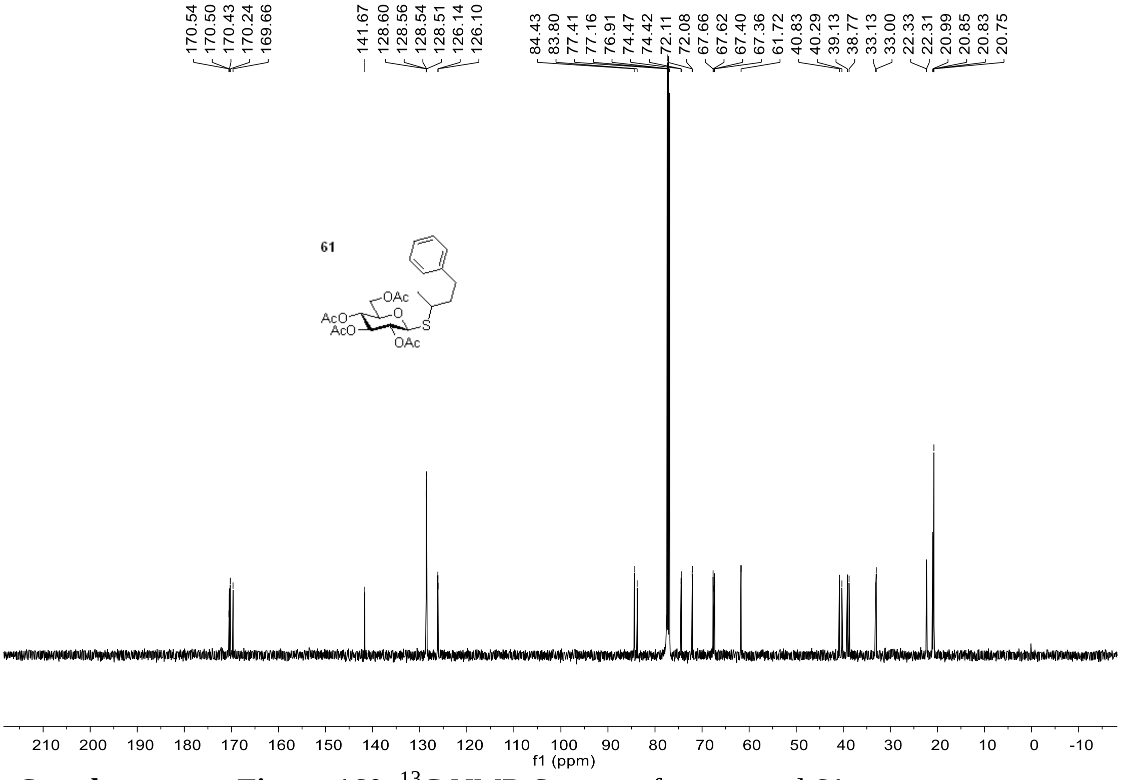

Supplementary Figure 160. ^{13}C NMR Spectra of compound **61**.

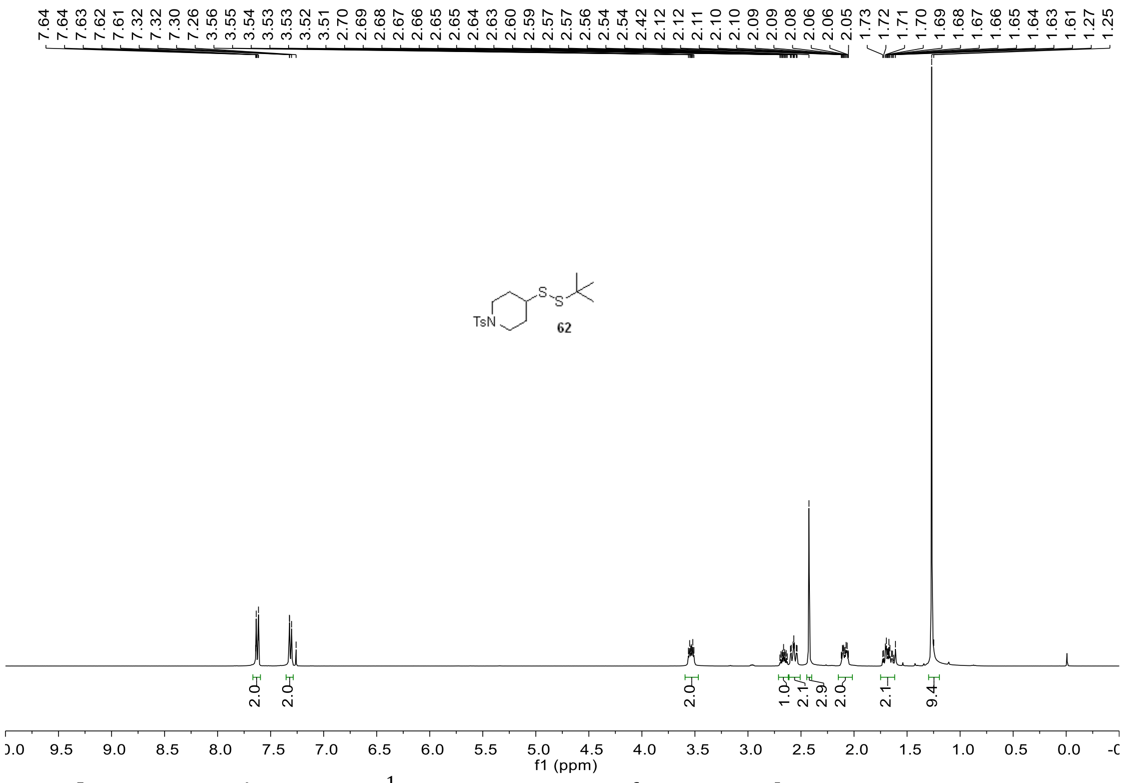

Supplementary Figure 161. ^{1}H NMR Spectra of compound **62**.

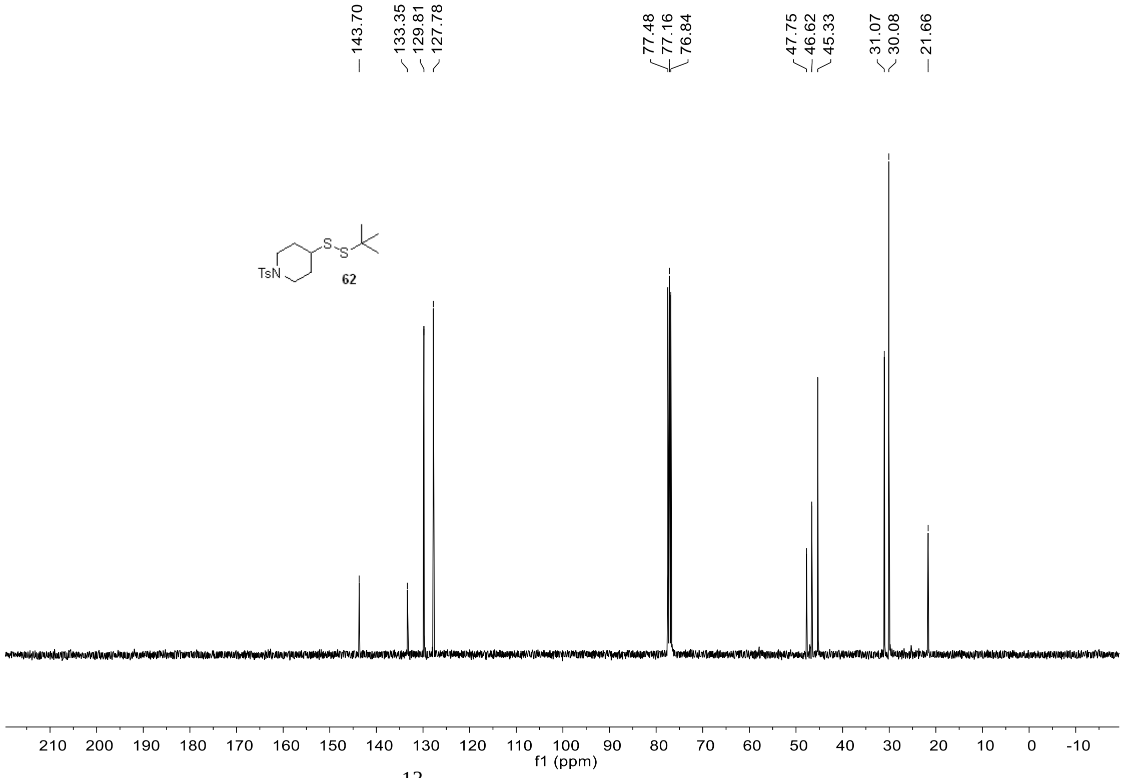

Supplementary Figure 162. ^{13}C NMR Spectra of compound **62**.

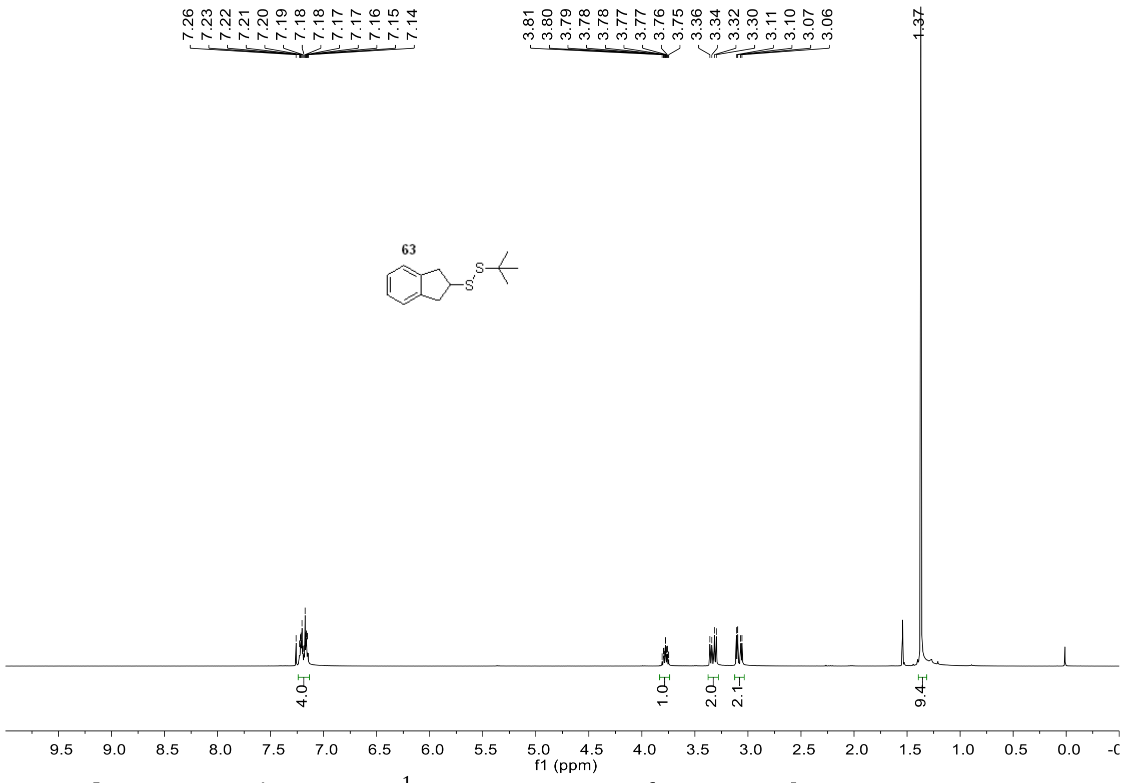

Supplementary Figure 163. ^{1}H NMR Spectra of compound **63**.

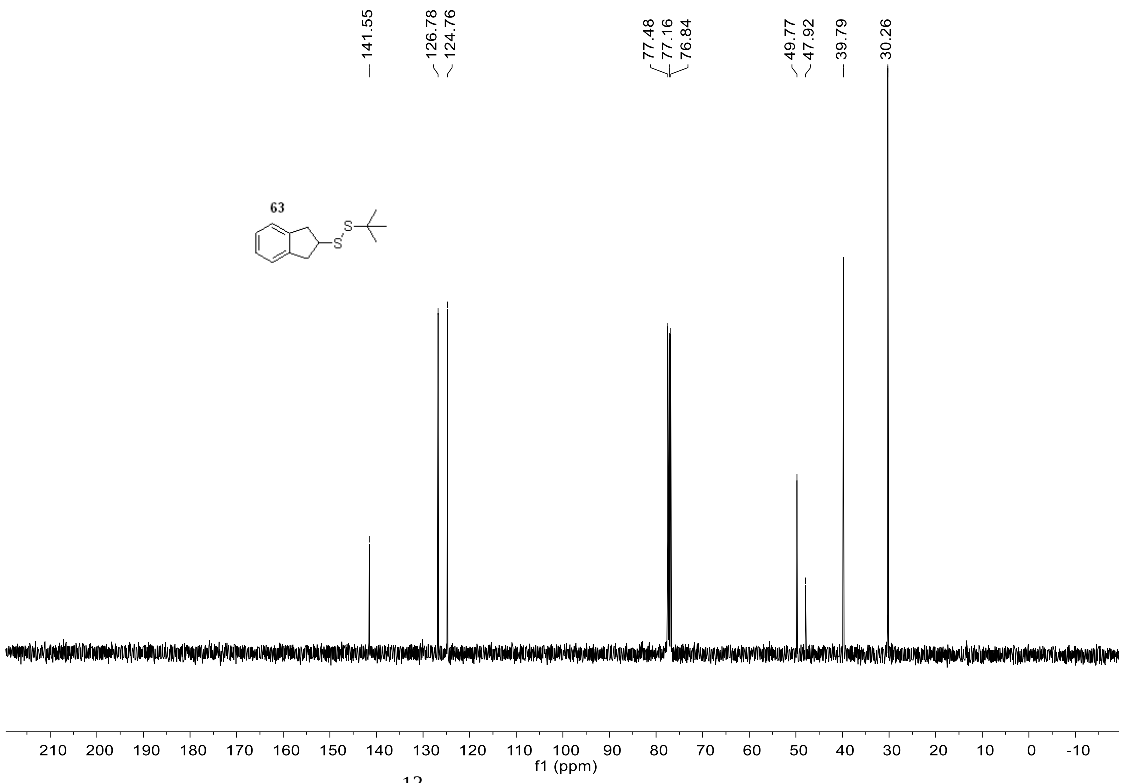

Supplementary Figure 164. ^{13}C NMR Spectra of compound **63**.

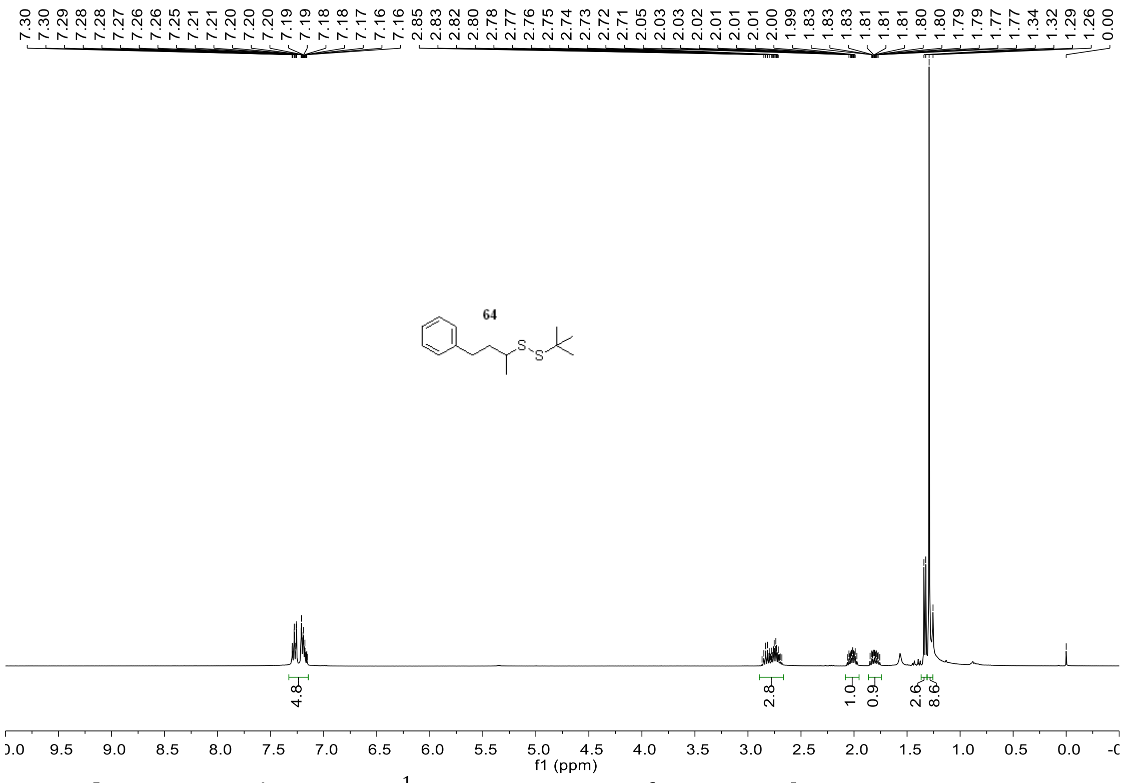

Supplementary Figure 165. ^{1}H NMR Spectra of compound **64**.

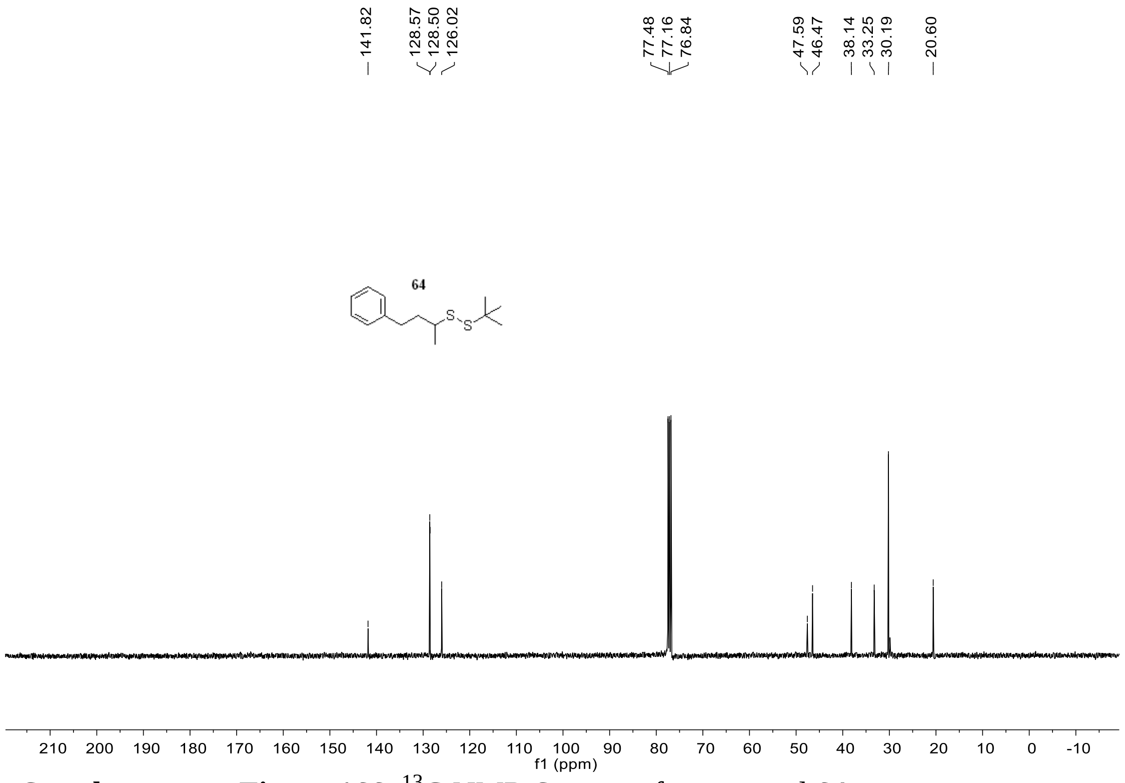

Supplementary Figure 166. ^{13}C NMR Spectra of compound **64**.

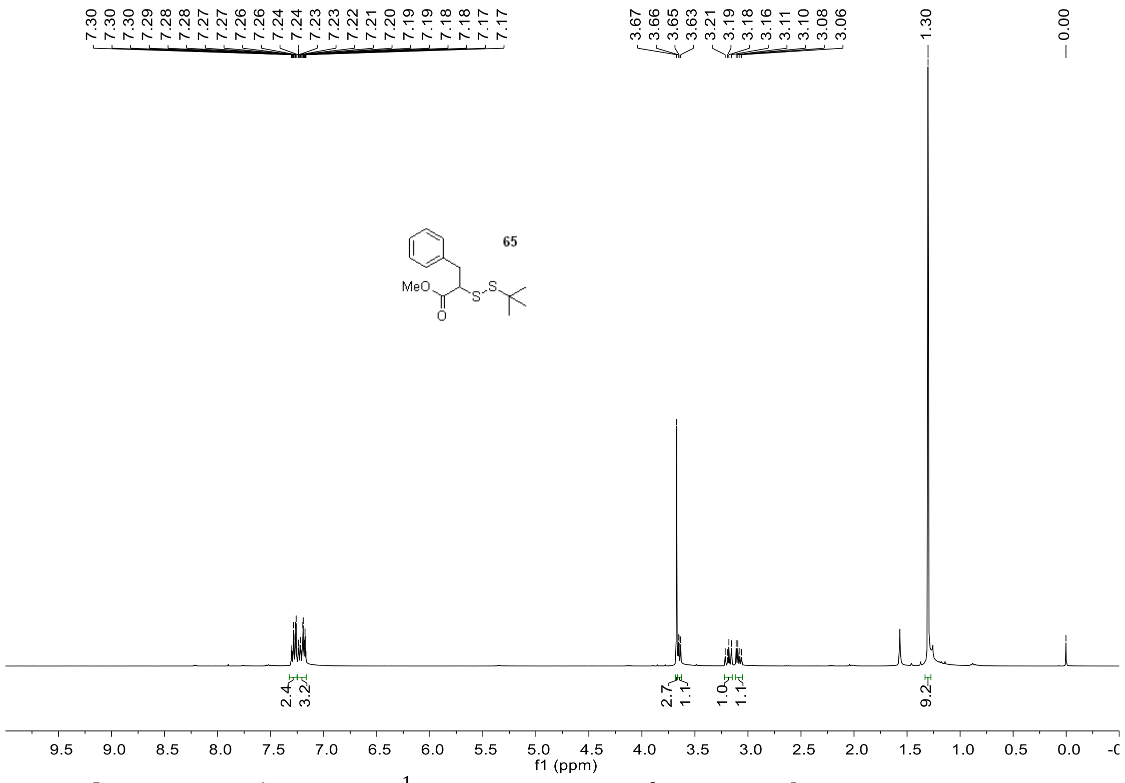

Supplementary Figure 167. ^{1}H NMR Spectra of compound **65**.

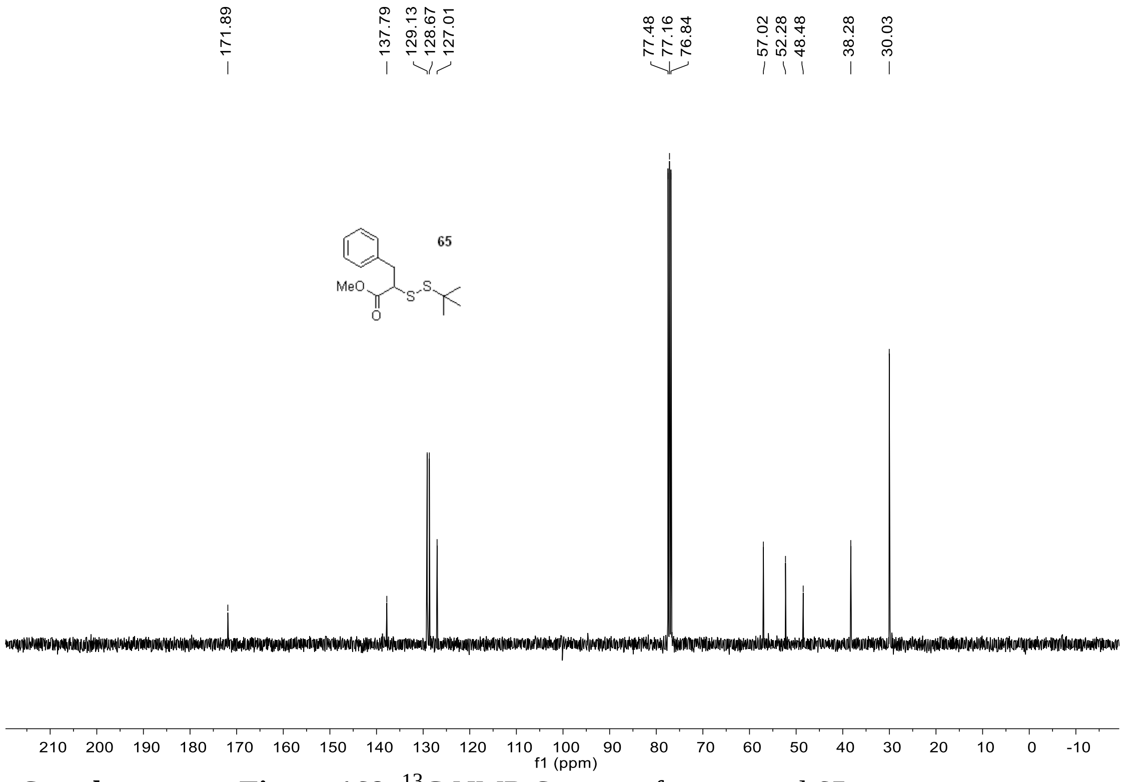

Supplementary Figure 168. ^{13}C NMR Spectra of compound **65**.

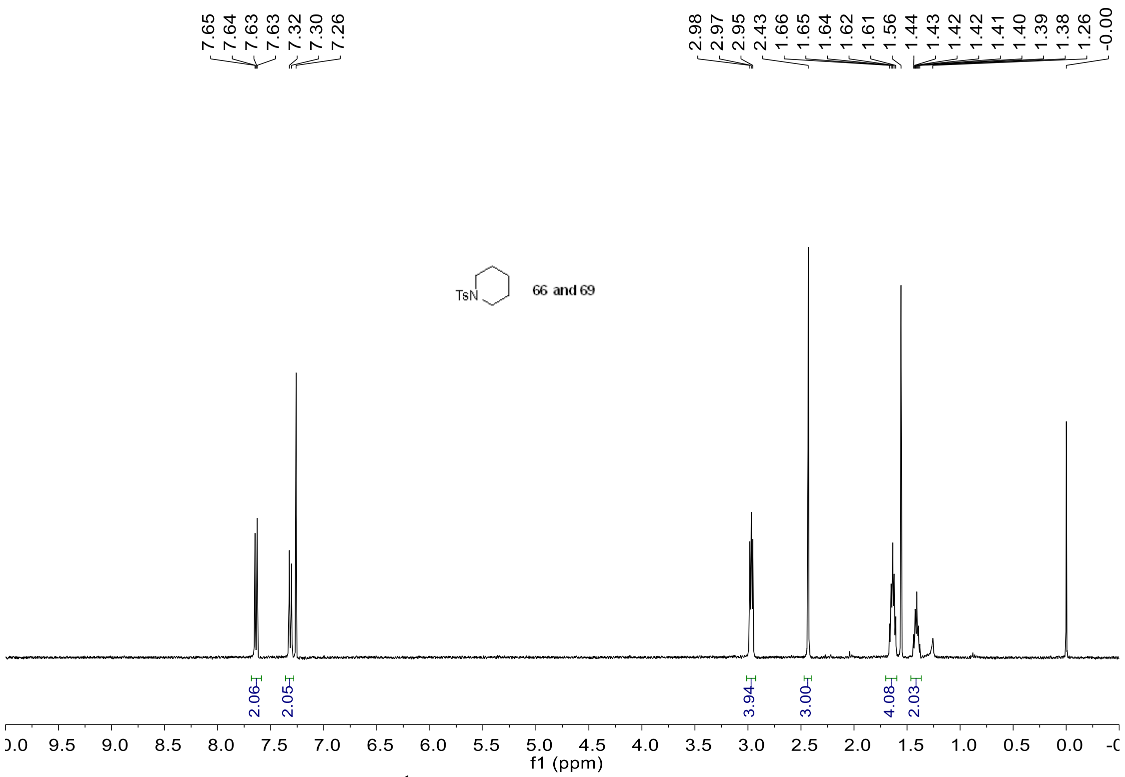

Supplementary Figure 169. ^{1}H NMR Spectra of compound **66 and 69**.

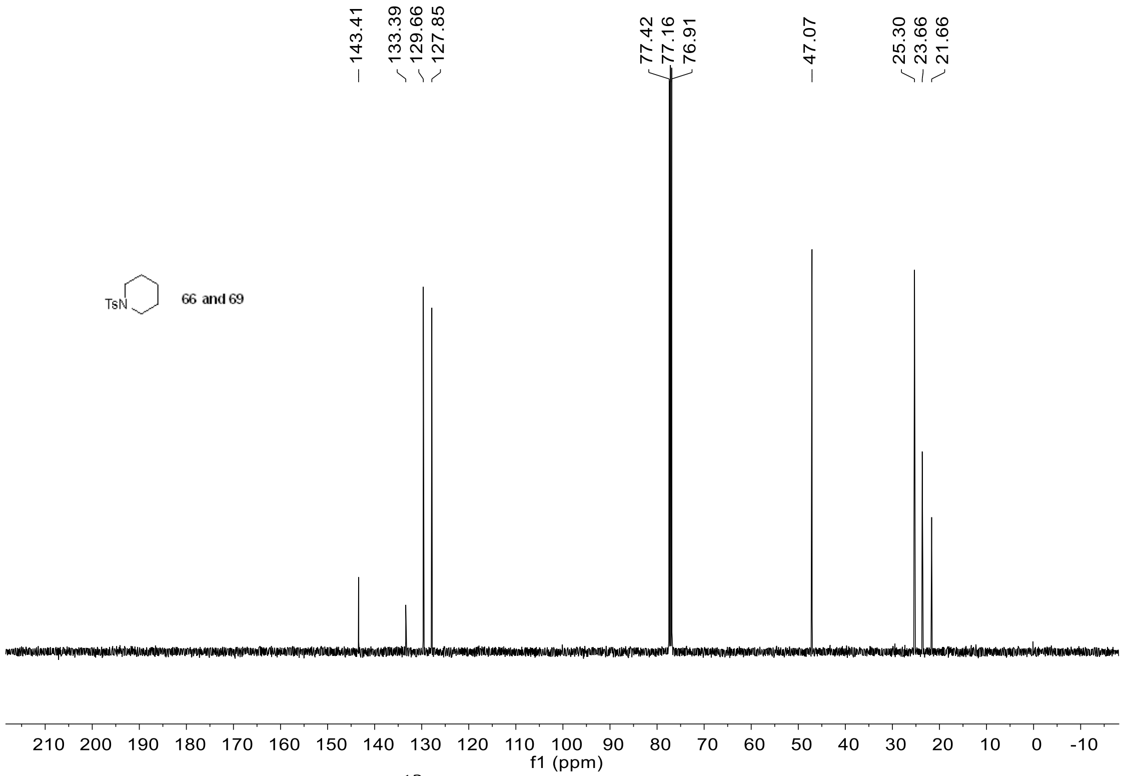

Supplementary Figure 170. ^{13}C NMR Spectra of compound **66 and 69**.

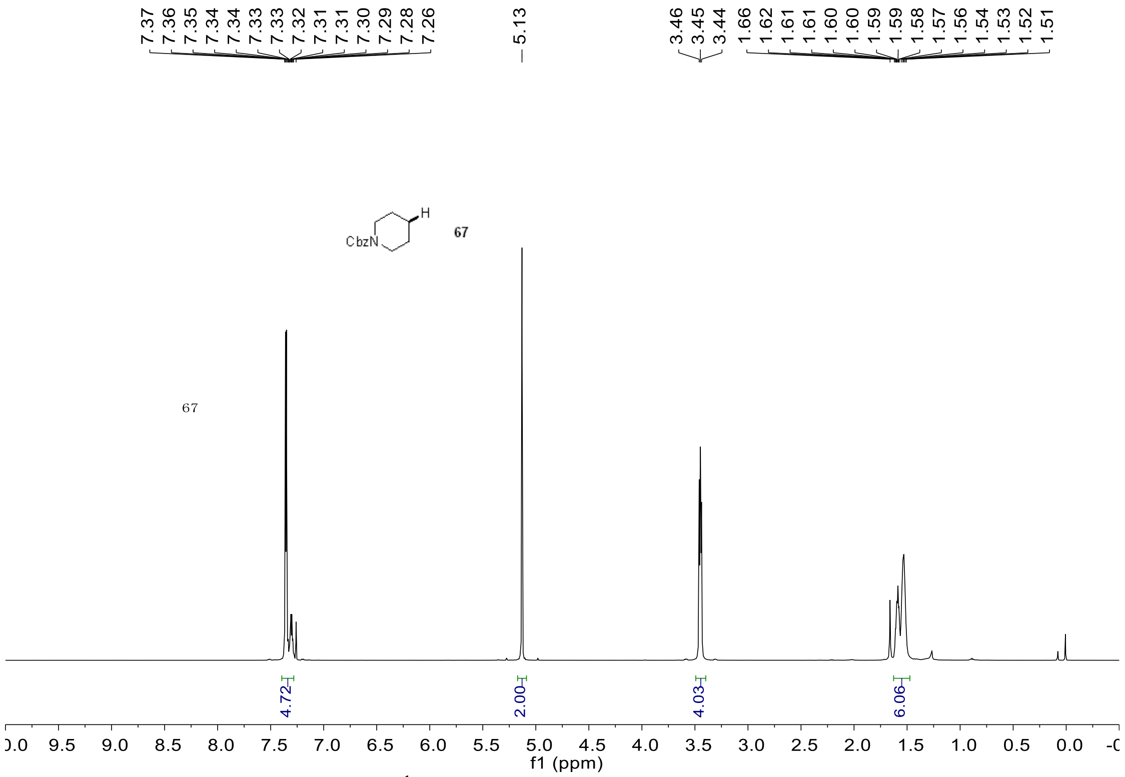

Supplementary Figure 171. ^{1}H NMR Spectra of compound **67**.

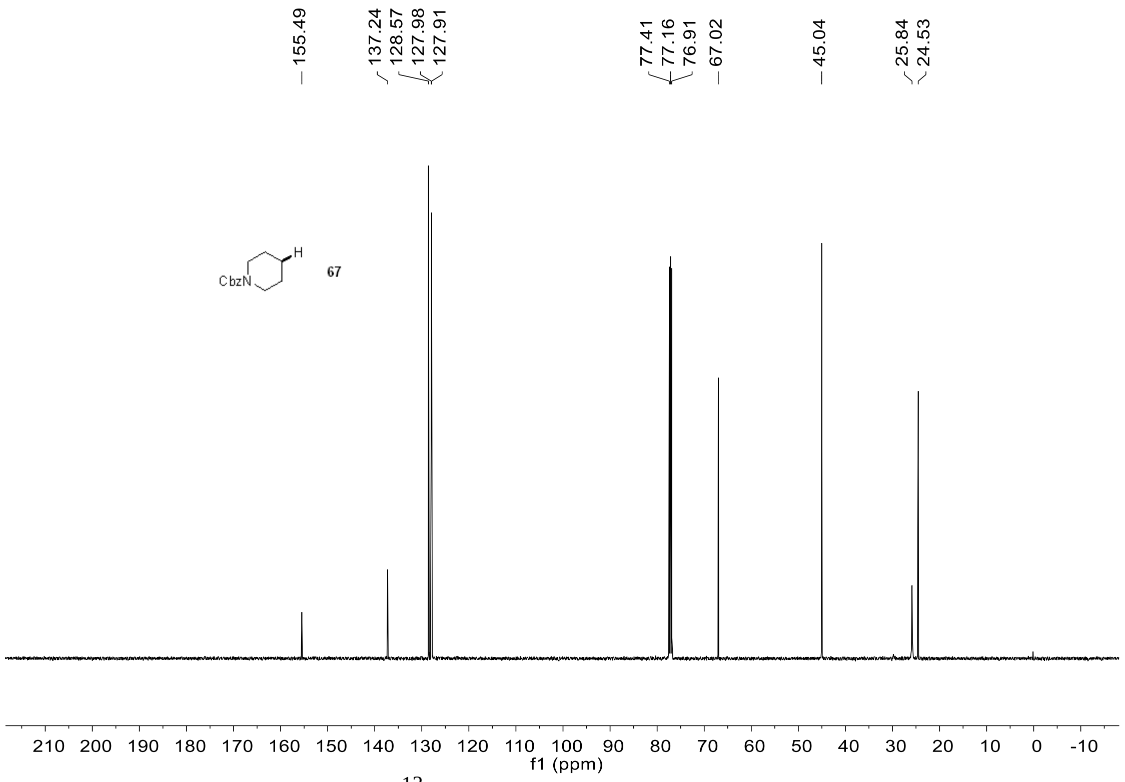

Supplementary Figure 172. ^{13}C NMR Spectra of compound **67**.

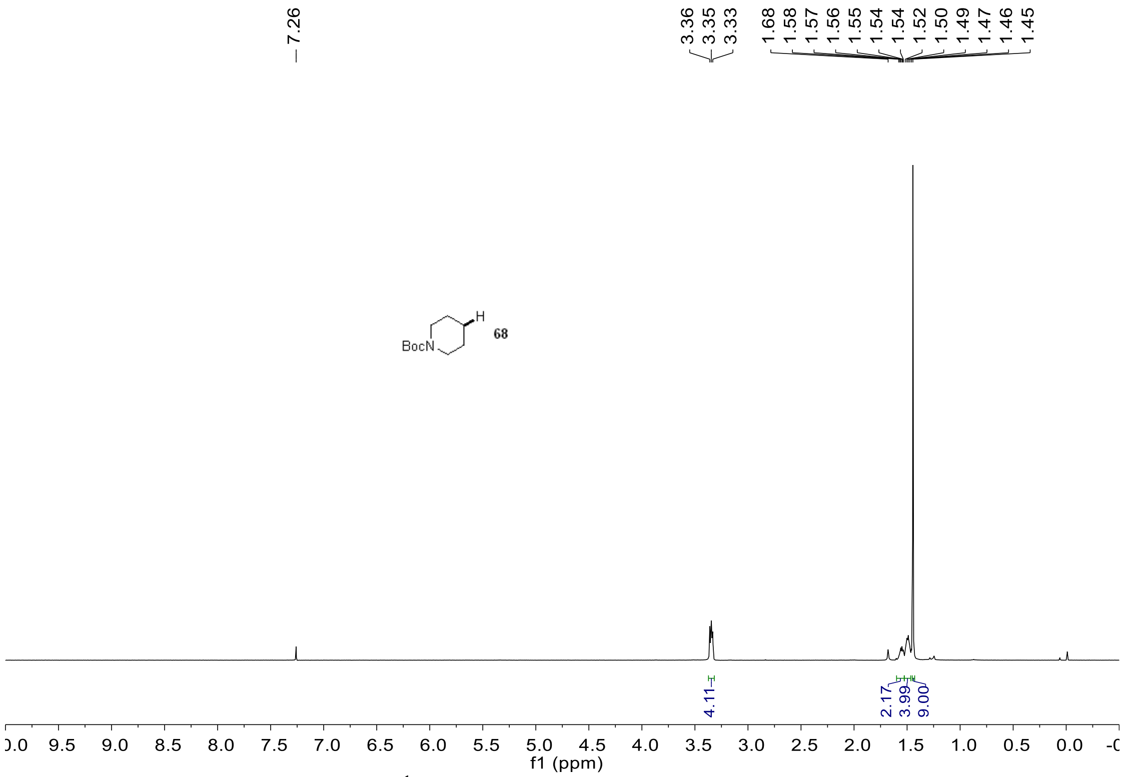

Supplementary Figure 173. ^{1}H NMR Spectra of compound **68**.

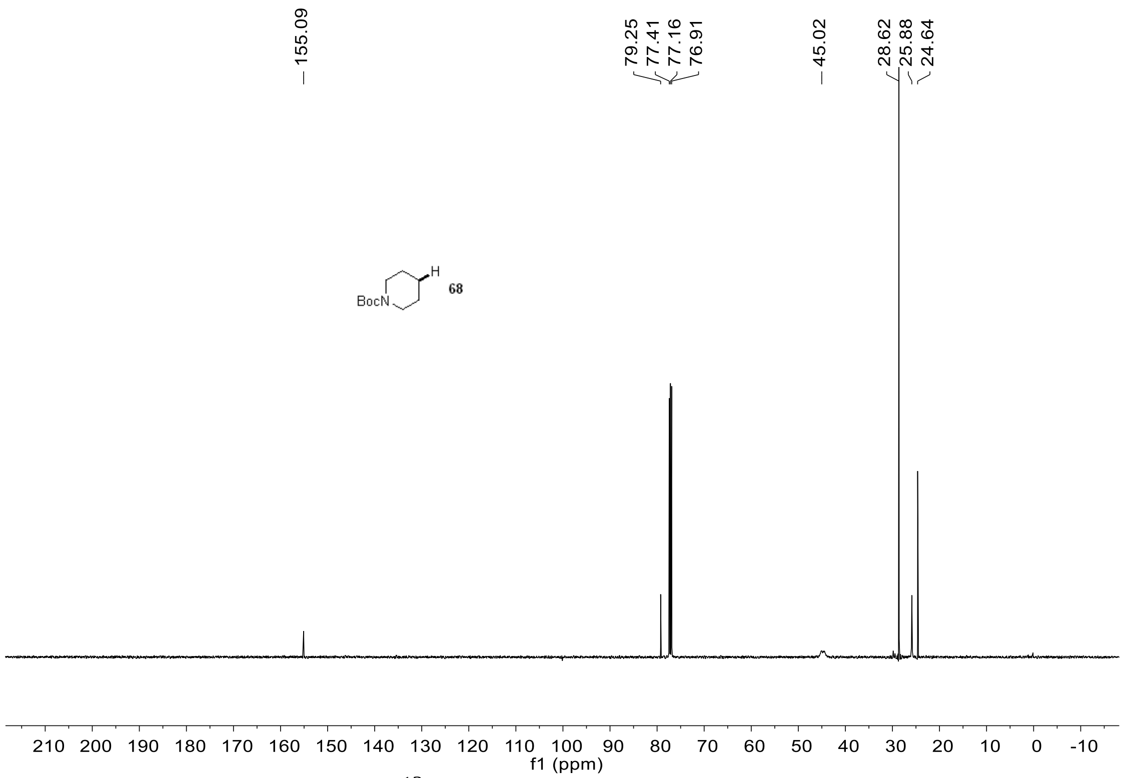

Supplementary Figure 174. ^{13}C NMR Spectra of compound **68**.

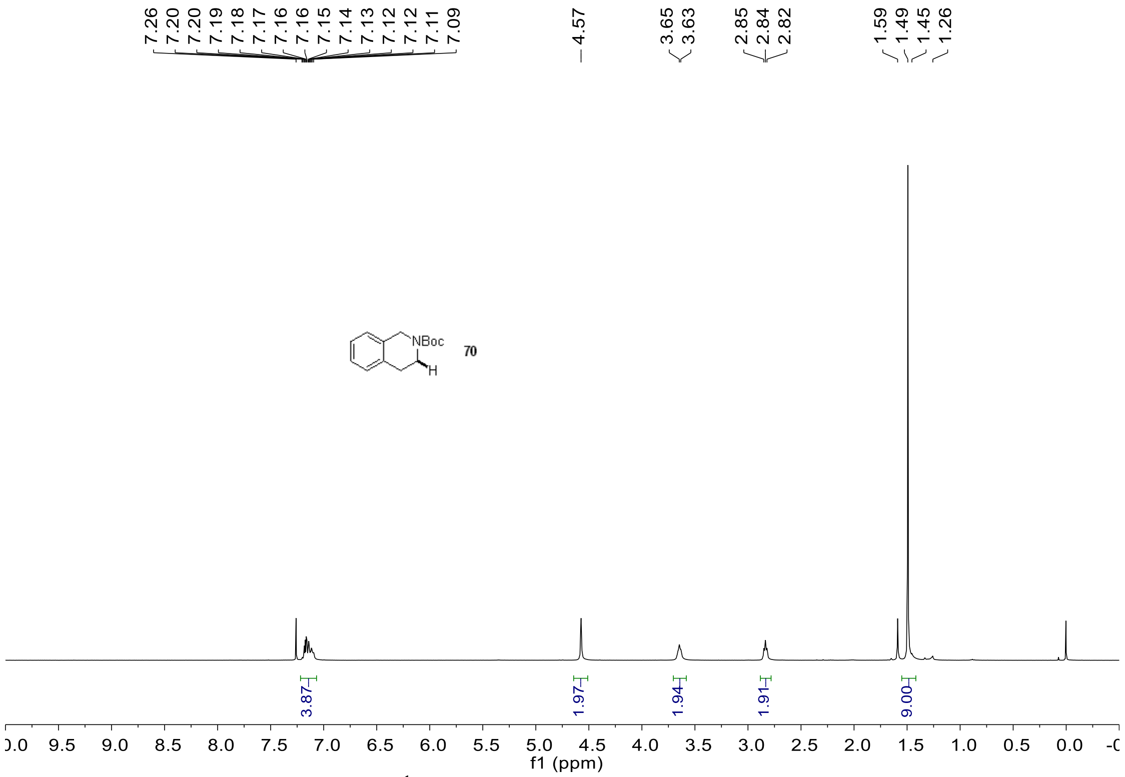

Supplementary Figure 175. ^{1}H NMR Spectra of compound **70**.

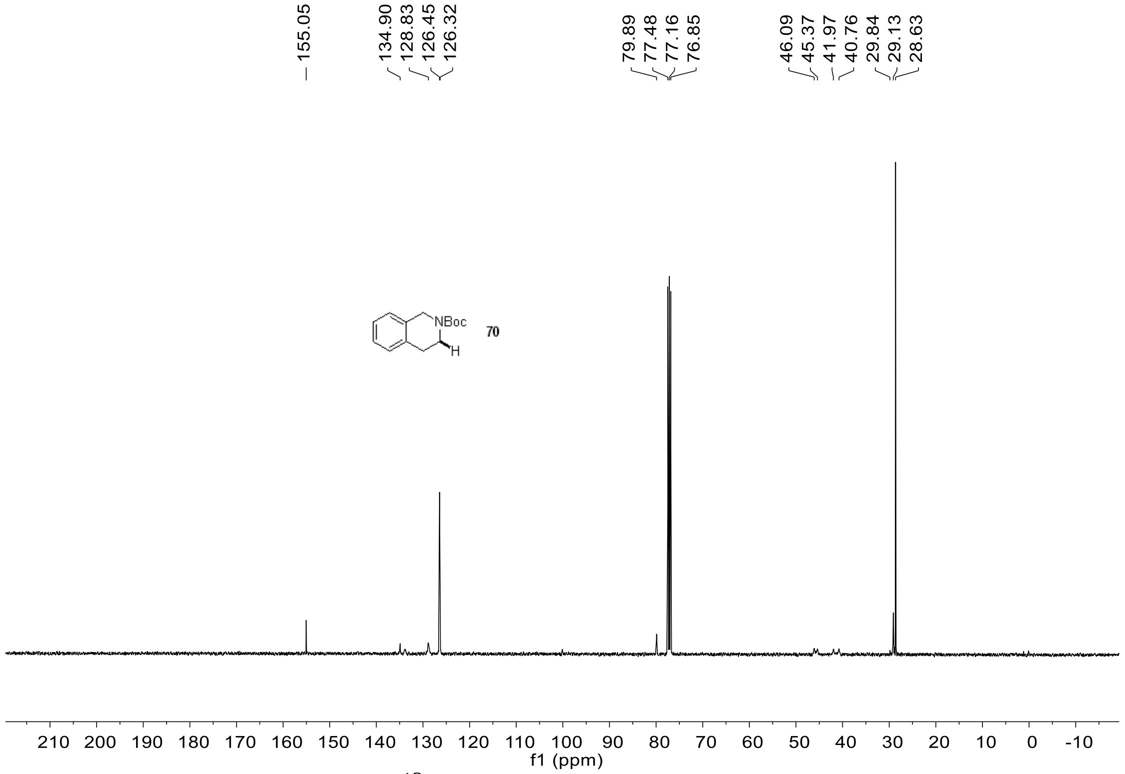

Supplementary Figure 176. ^{13}C NMR Spectra of compound **70**.

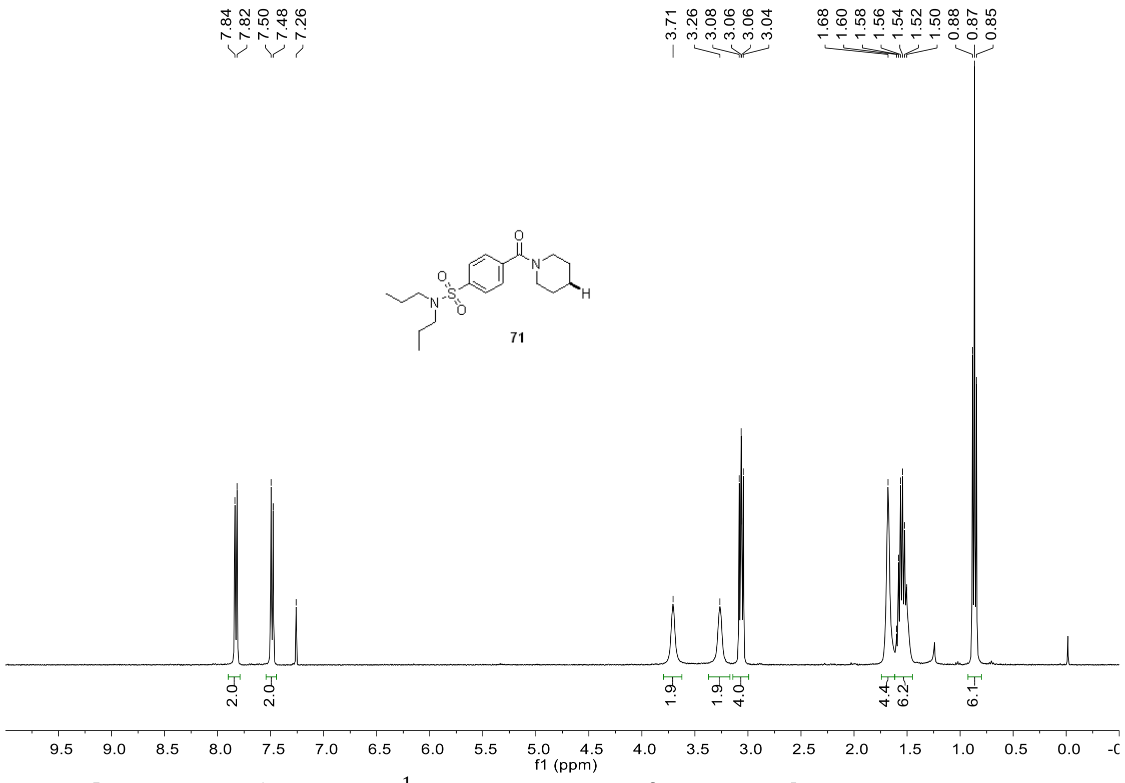

Supplementary Figure 177. ^{1}H NMR Spectra of compound **71**.

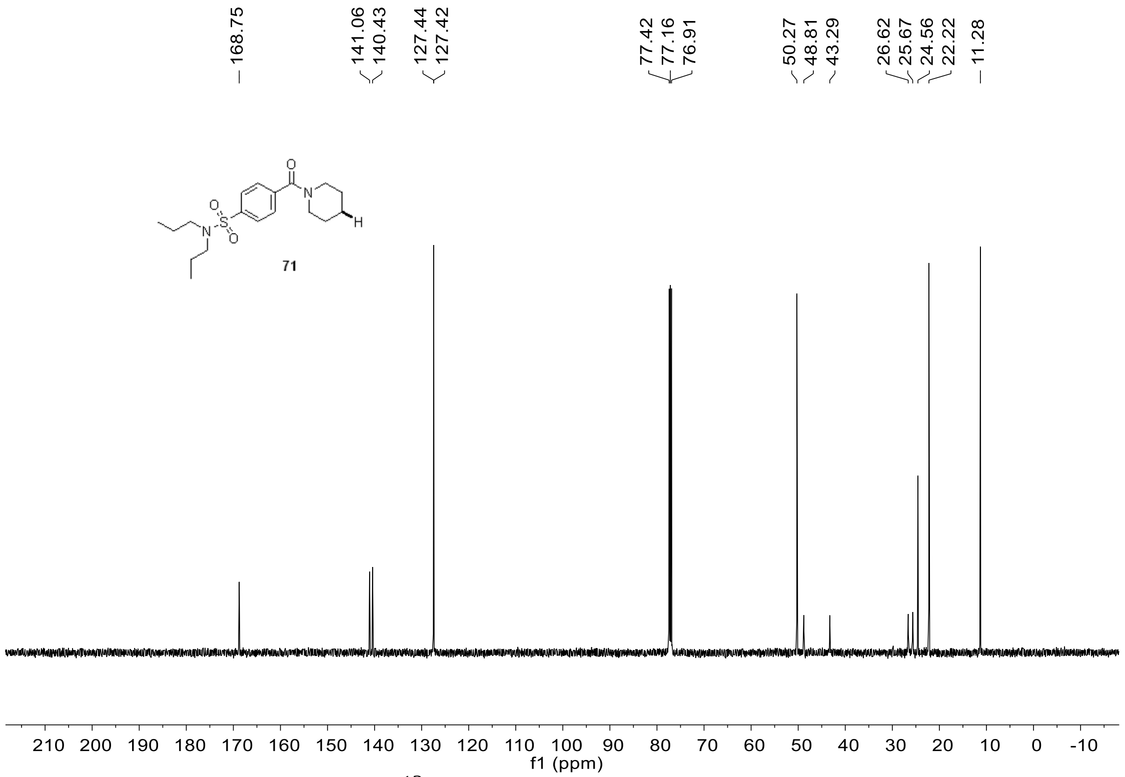

Supplementary Figure 178. ^{13}C NMR Spectra of compound **71**.

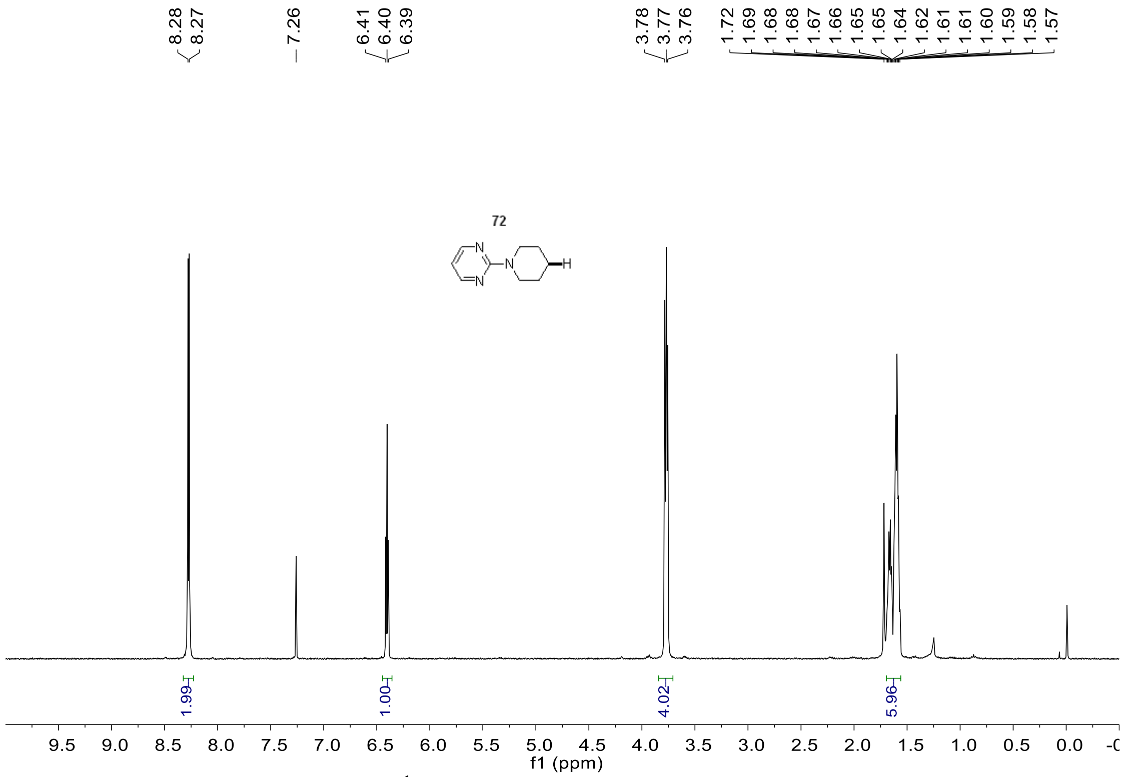

Supplementary Figure 179. ^{1}H NMR Spectra of compound **72**.

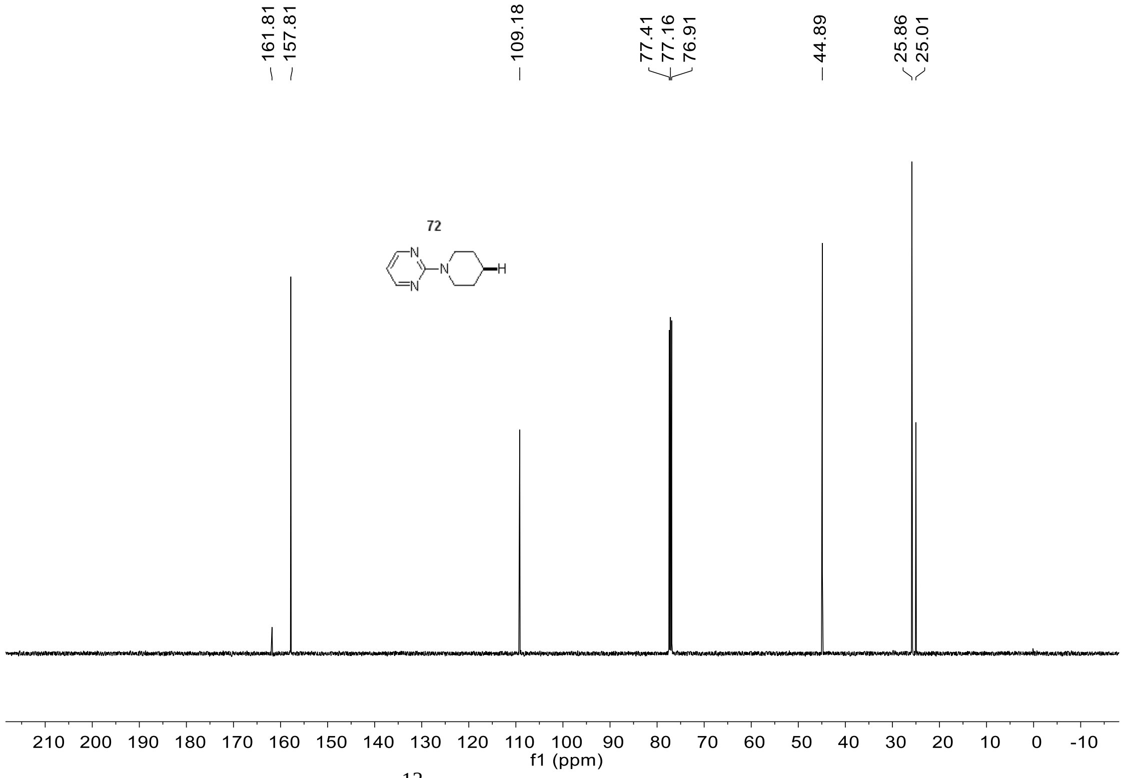

Supplementary Figure 180. ^{13}C NMR Spectra of compound **72**.

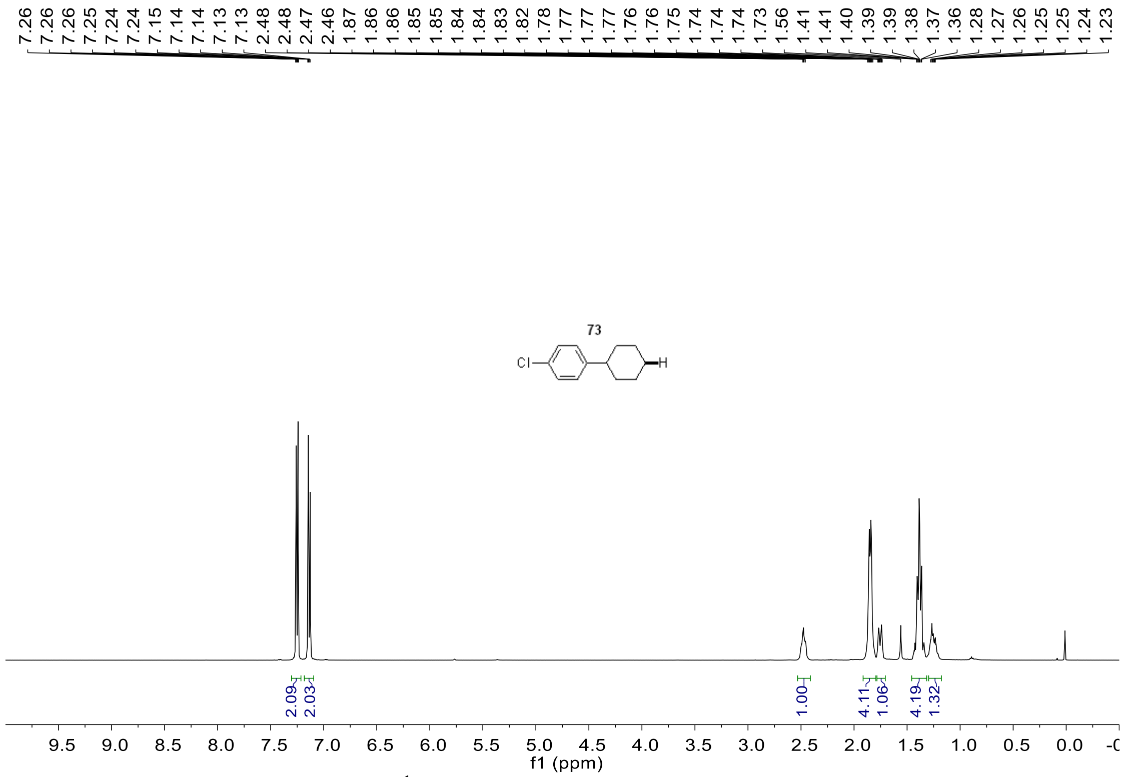

Supplementary Figure 181. ^{1}H NMR Spectra of compound **73**.

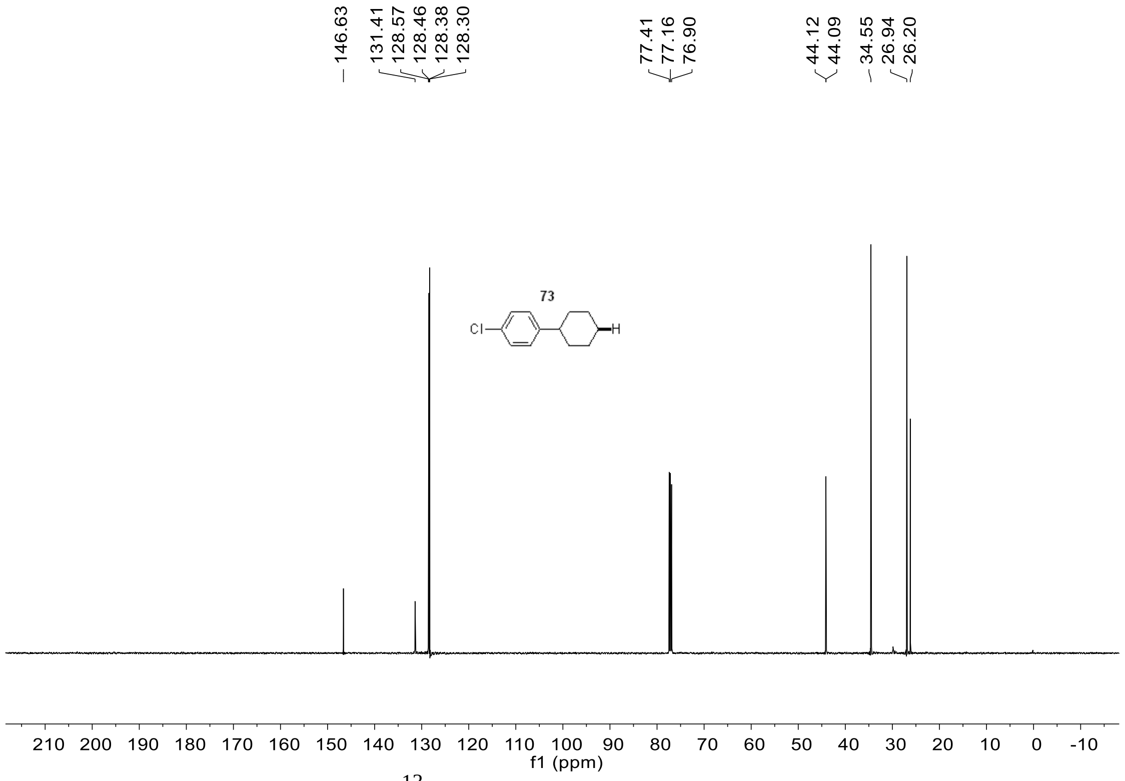

Supplementary Figure 182. ^{13}C NMR Spectra of compound **73**.

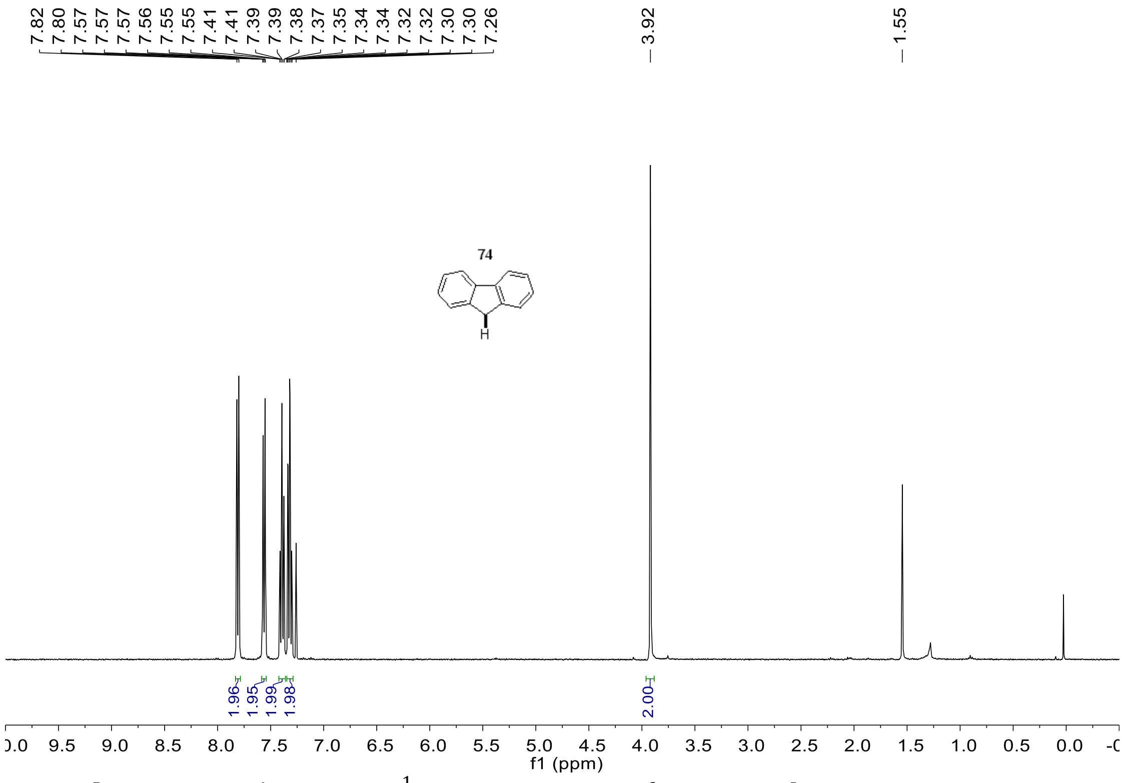

Supplementary Figure 183. ^{1}H NMR Spectra of compound **74**.

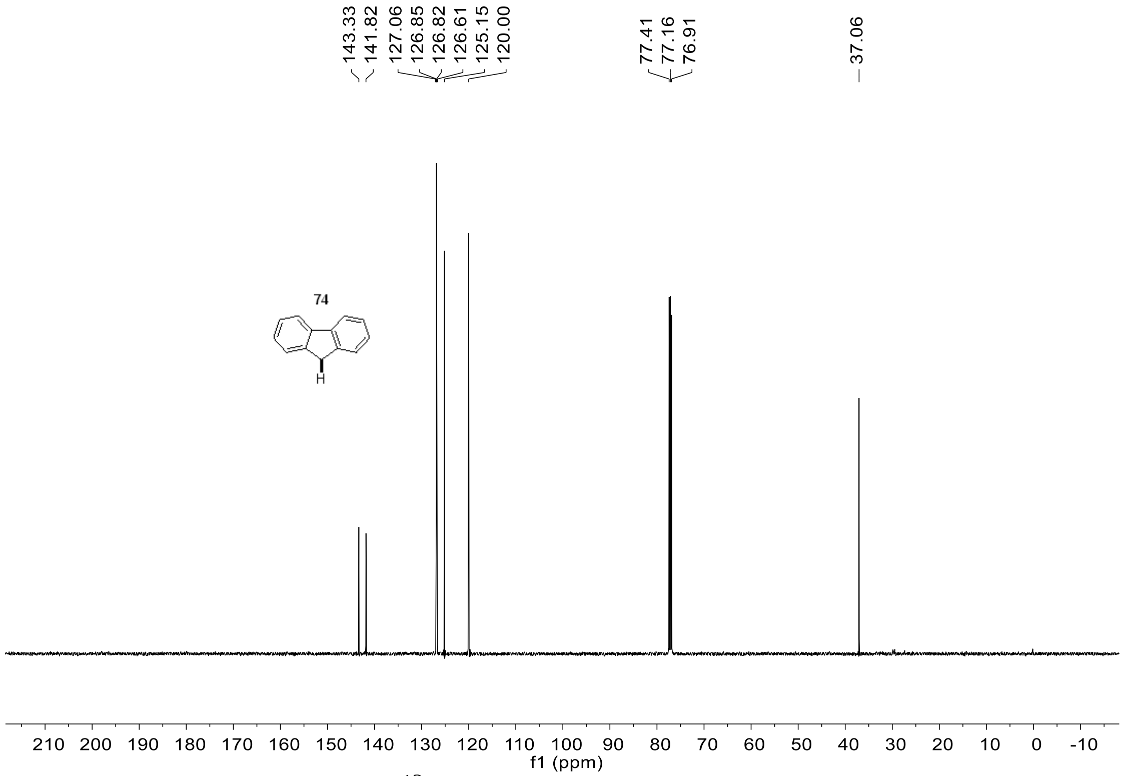

Supplementary Figure 184. ^{13}C NMR Spectra of compound **74**.

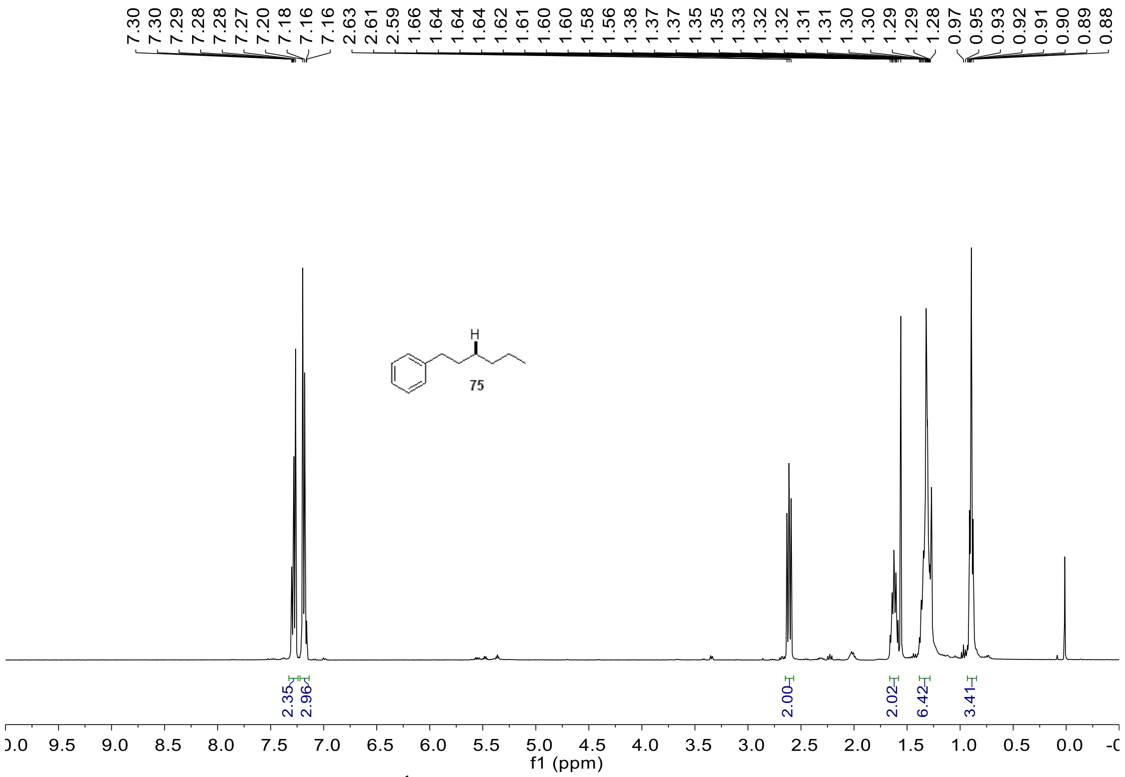

Supplementary Figure 185. ^{1}H NMR Spectra of compound **75**.

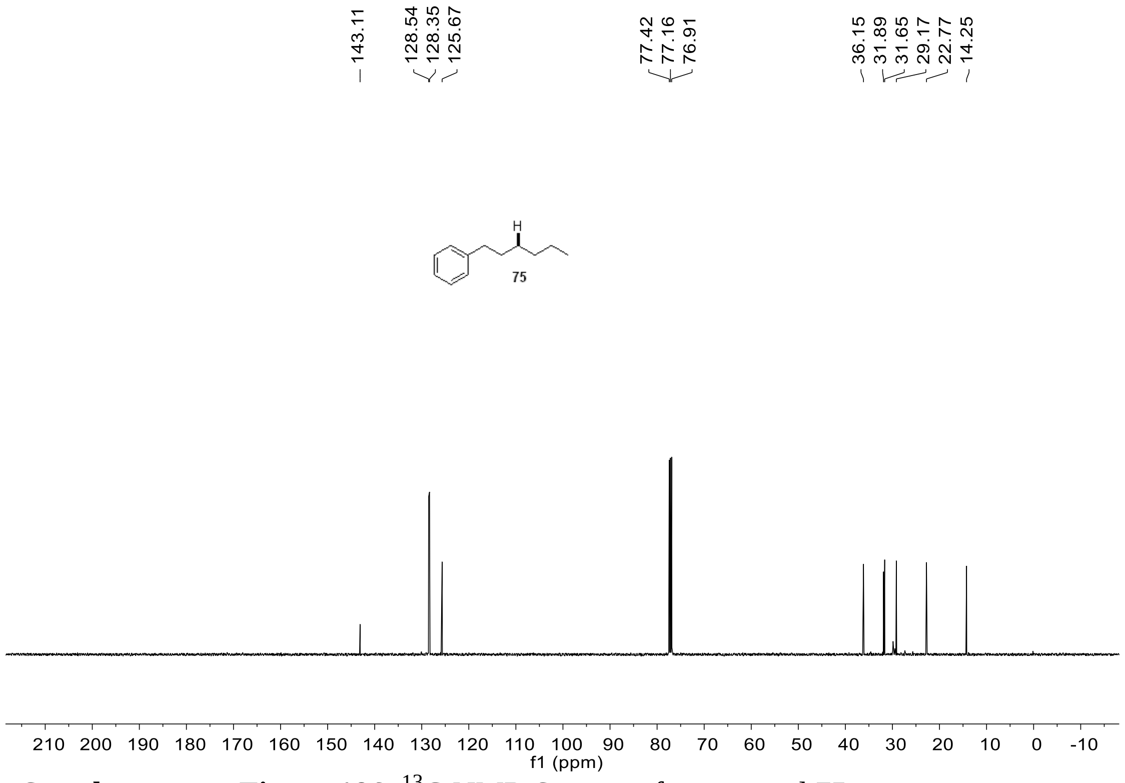

Supplementary Figure 186. ^{13}C NMR Spectra of compound **75**.

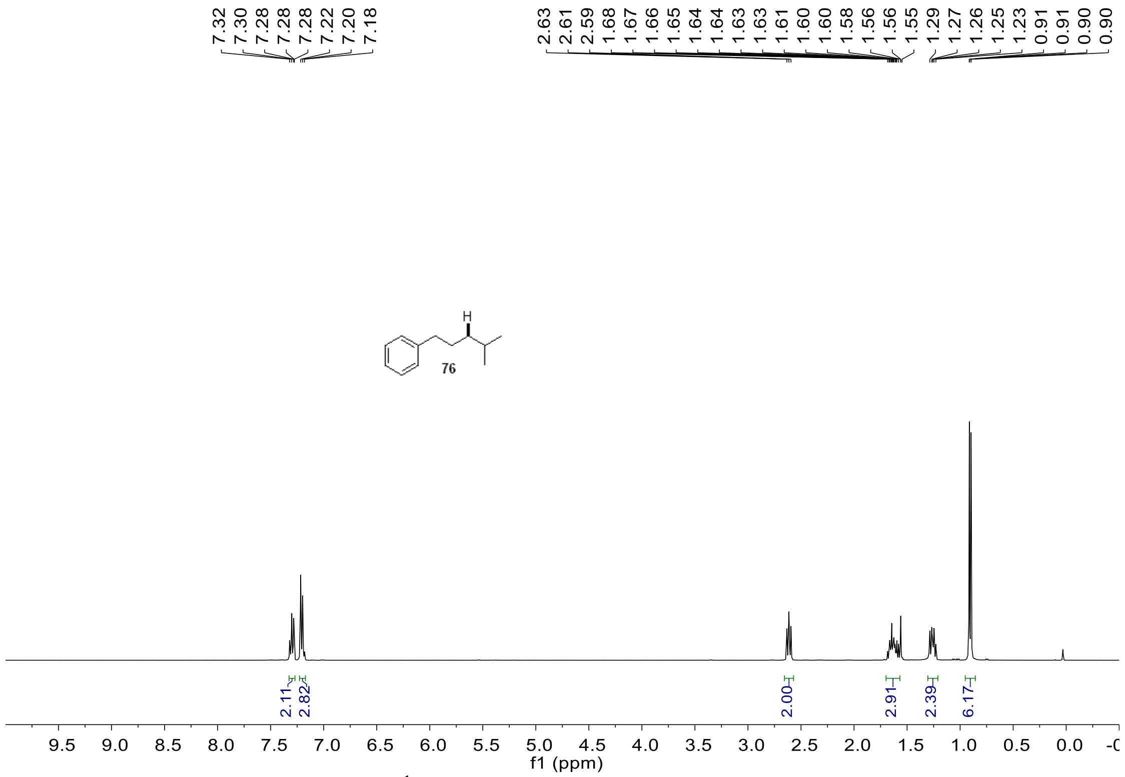

Supplementary Figure 187. ^{1}H NMR Spectra of compound **76**.

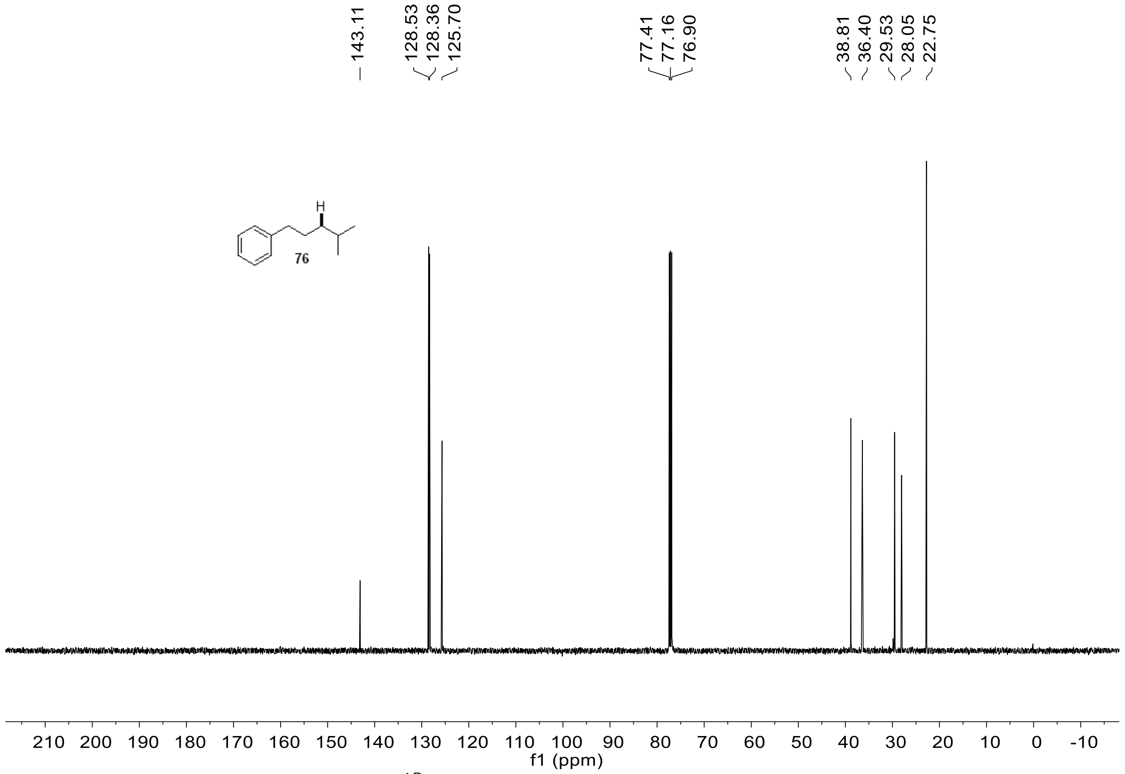

Supplementary Figure 188. ^{13}C NMR Spectra of compound **76**.

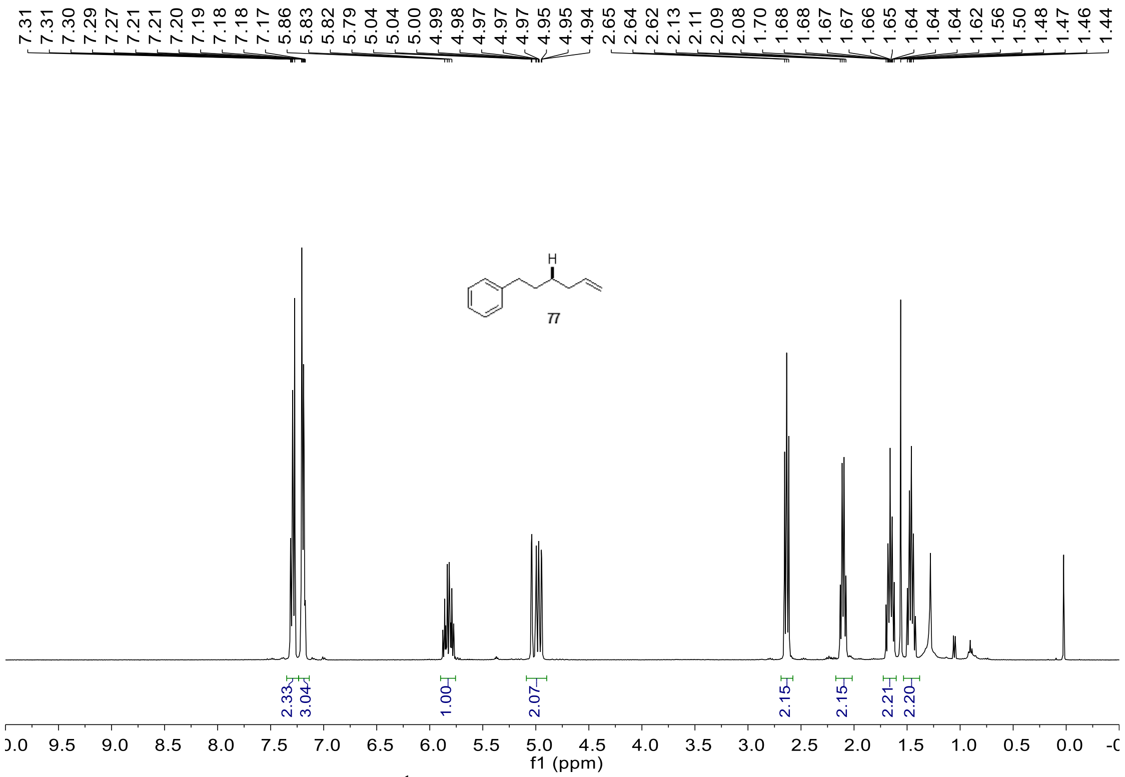

Supplementary Figure 189. ^{1}H NMR Spectra of compound **77**.

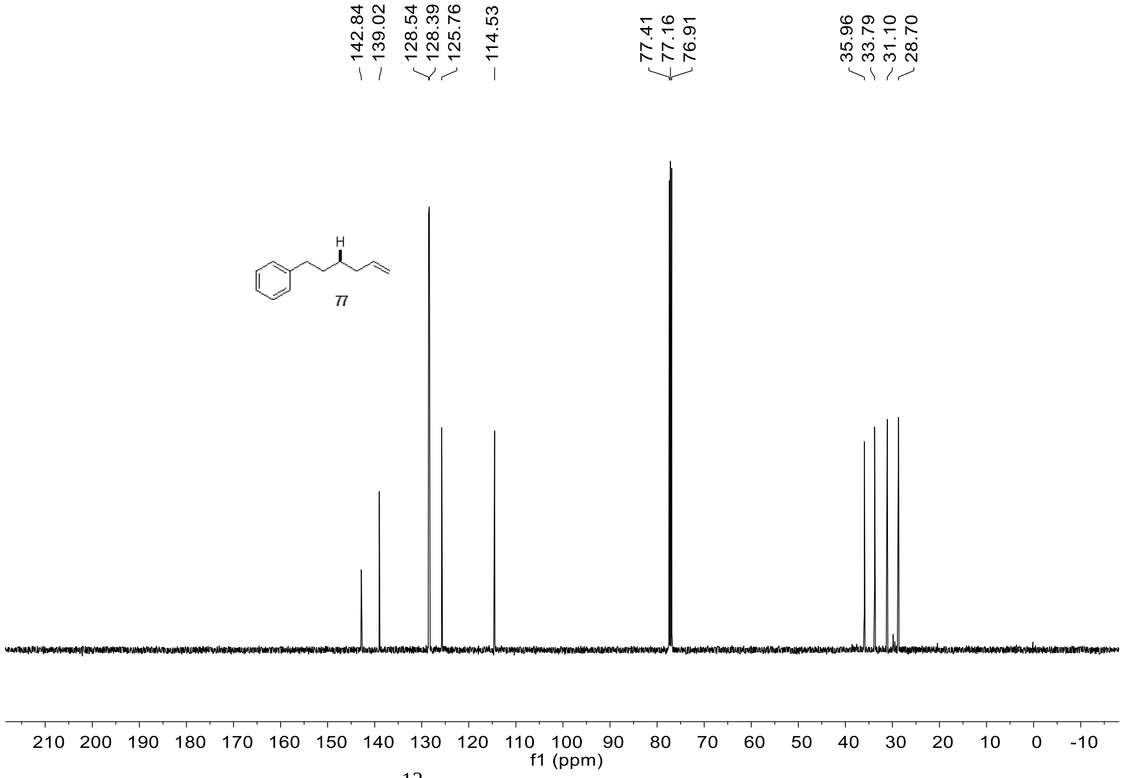

Supplementary Figure 190. ^{13}C NMR Spectra of compound **77**.

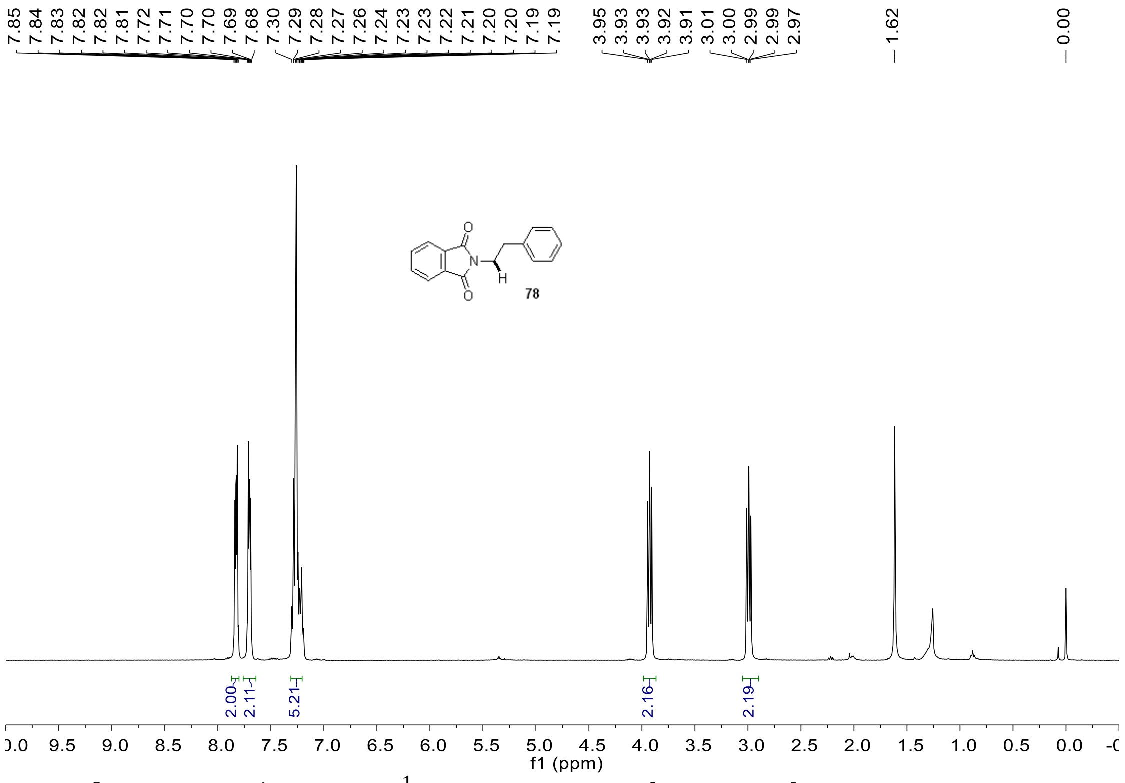

Supplementary Figure 191. ^{1}H NMR Spectra of compound **78**.

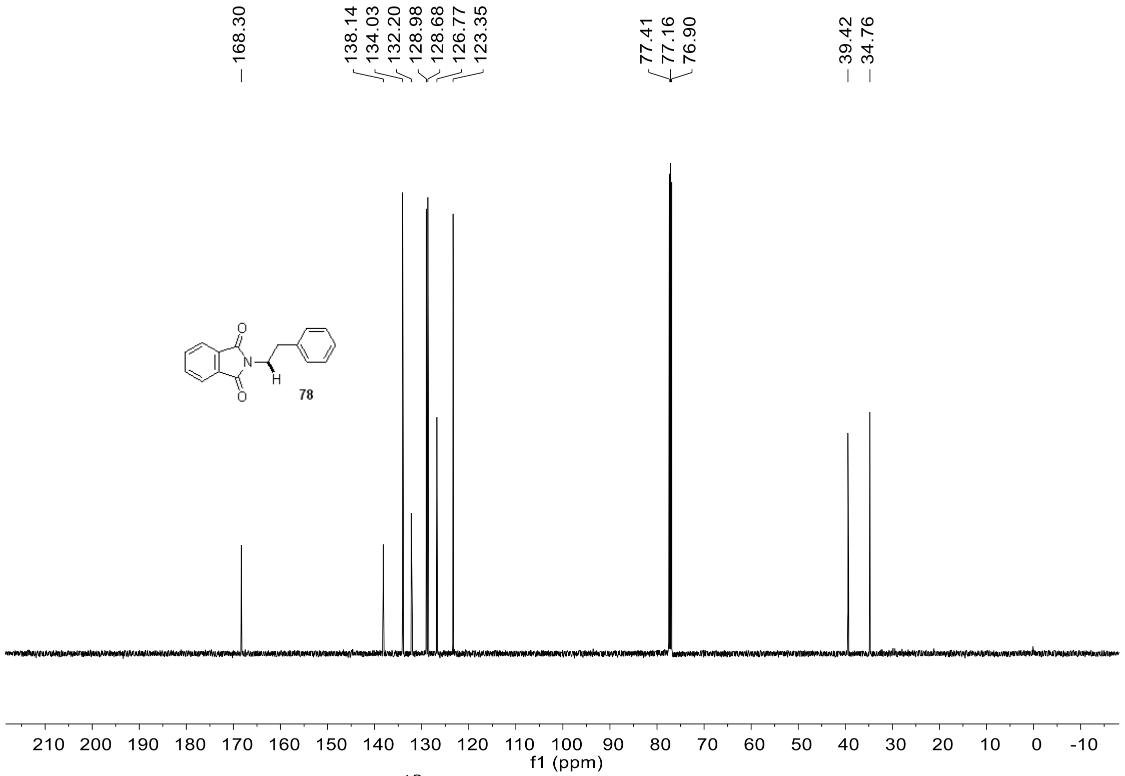

Supplementary Figure 192. ^{13}C NMR Spectra of compound **78**.

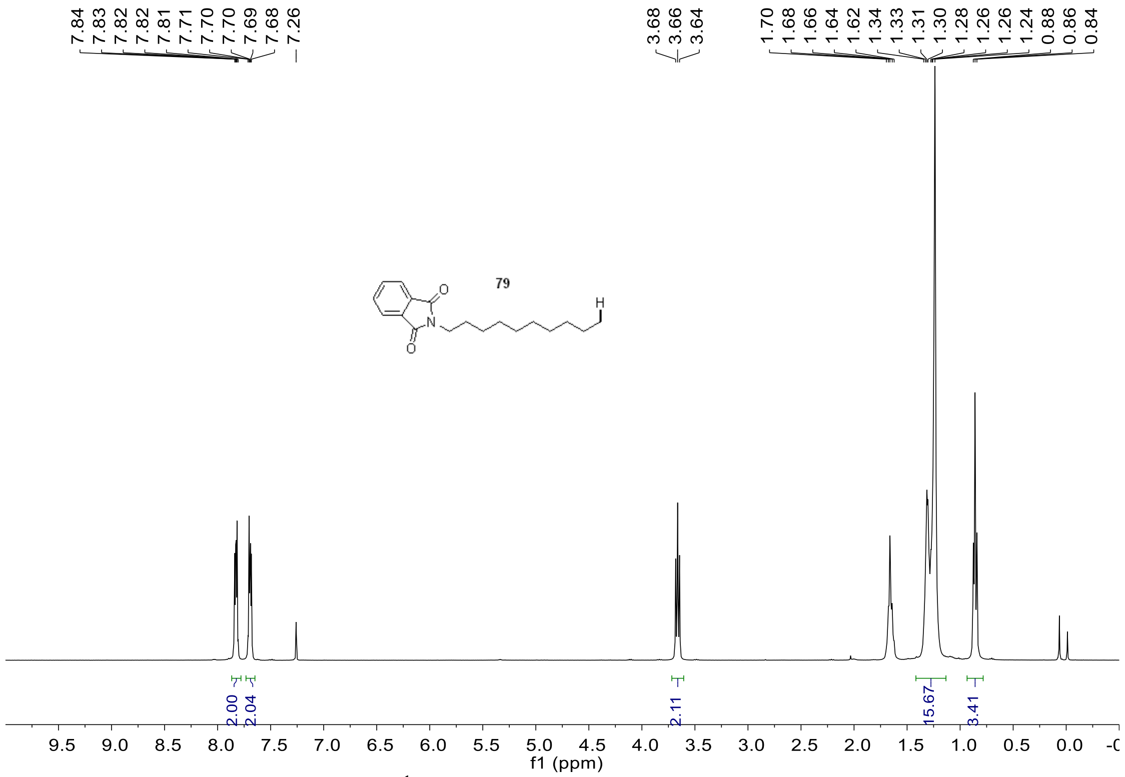

Supplementary Figure 193. ^{1}H NMR Spectra of compound **79**.

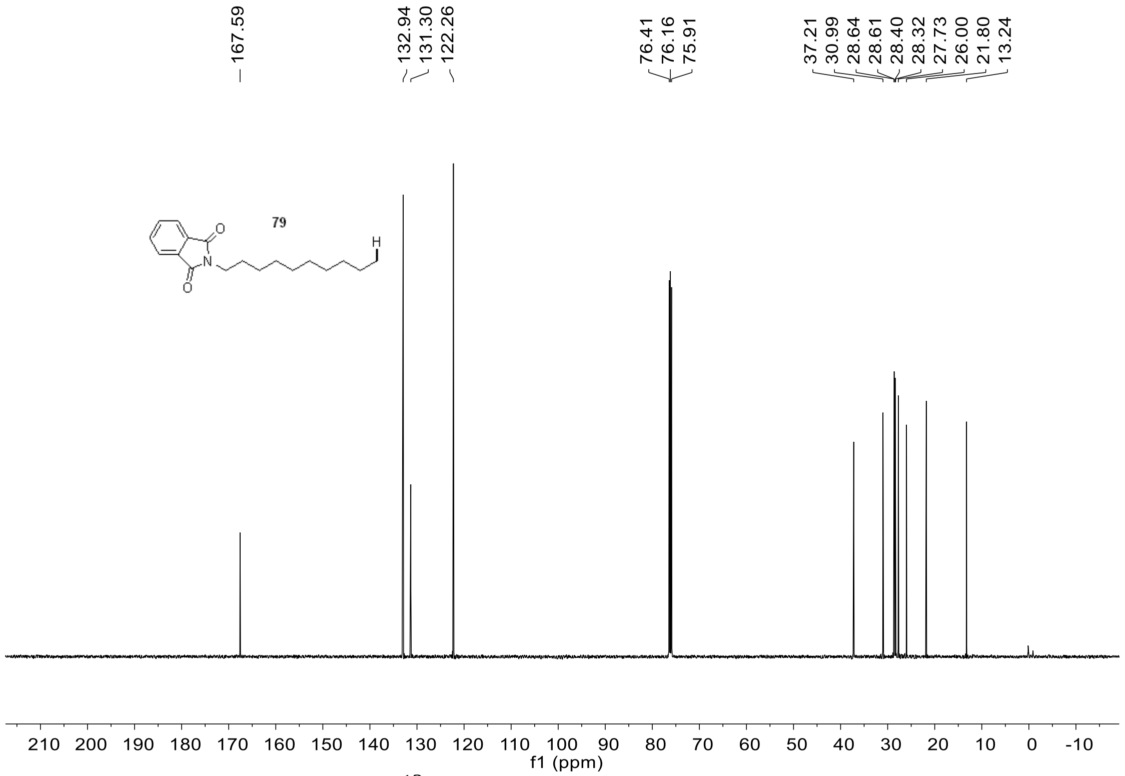

Supplementary Figure 194. ^{13}C NMR Spectra of compound **79**.

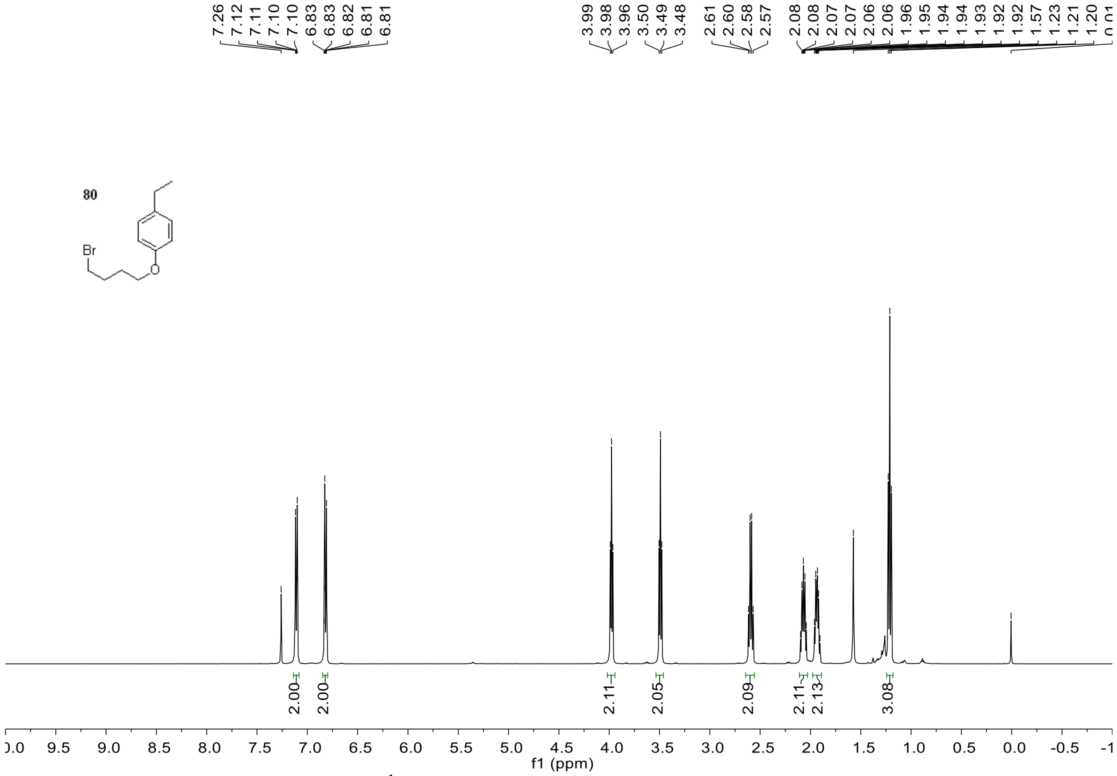

Supplementary Figure 195. ^{1}H NMR Spectra of compound **80**.

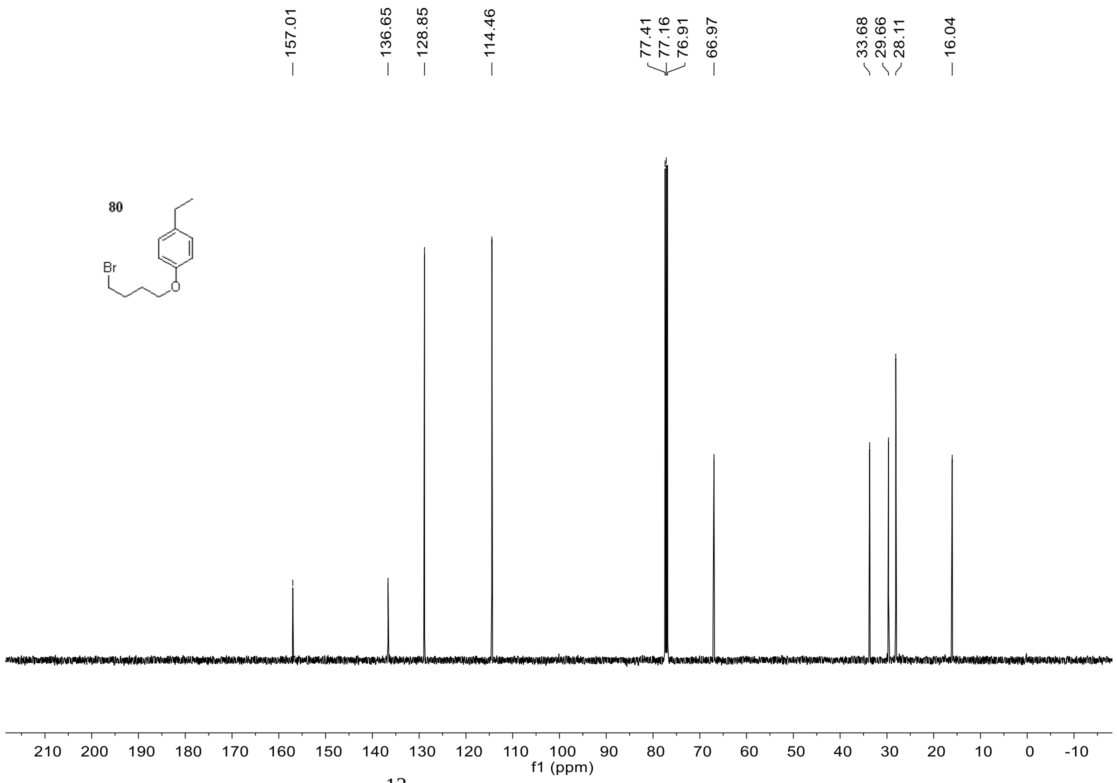

Supplementary Figure 196. ^{13}C NMR Spectra of compound **80**.

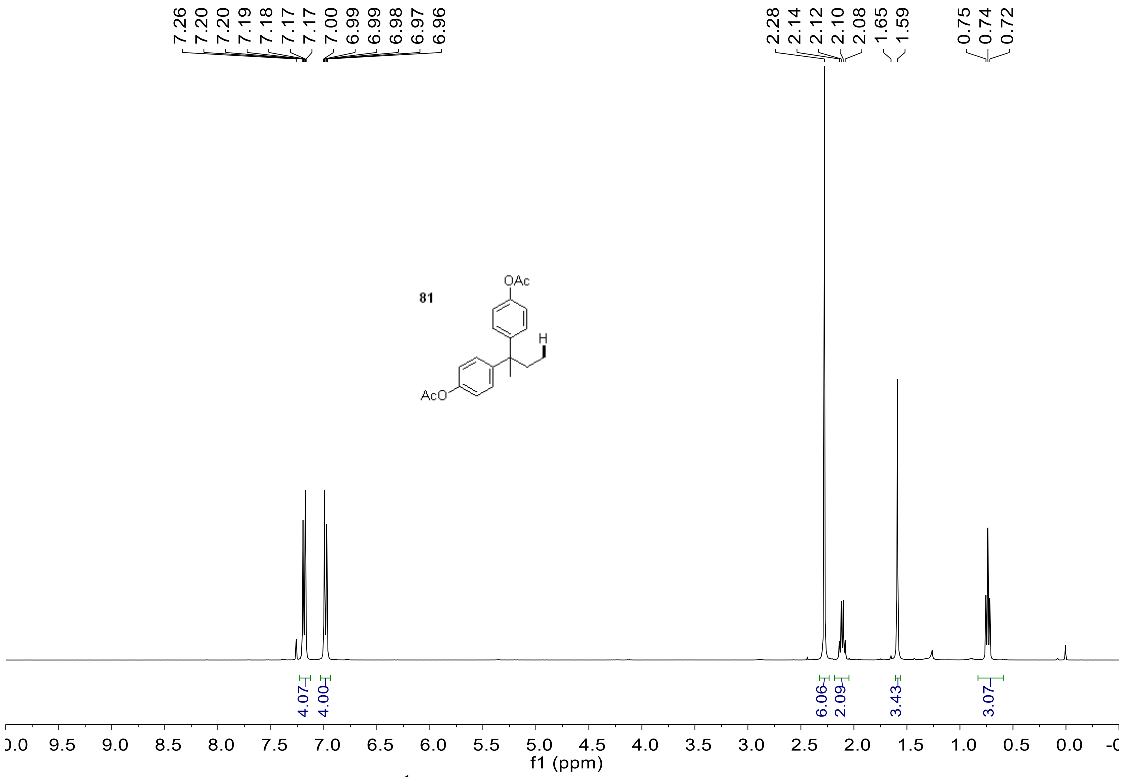

Supplementary Figure 197. ^{1}H NMR Spectra of compound **81**.

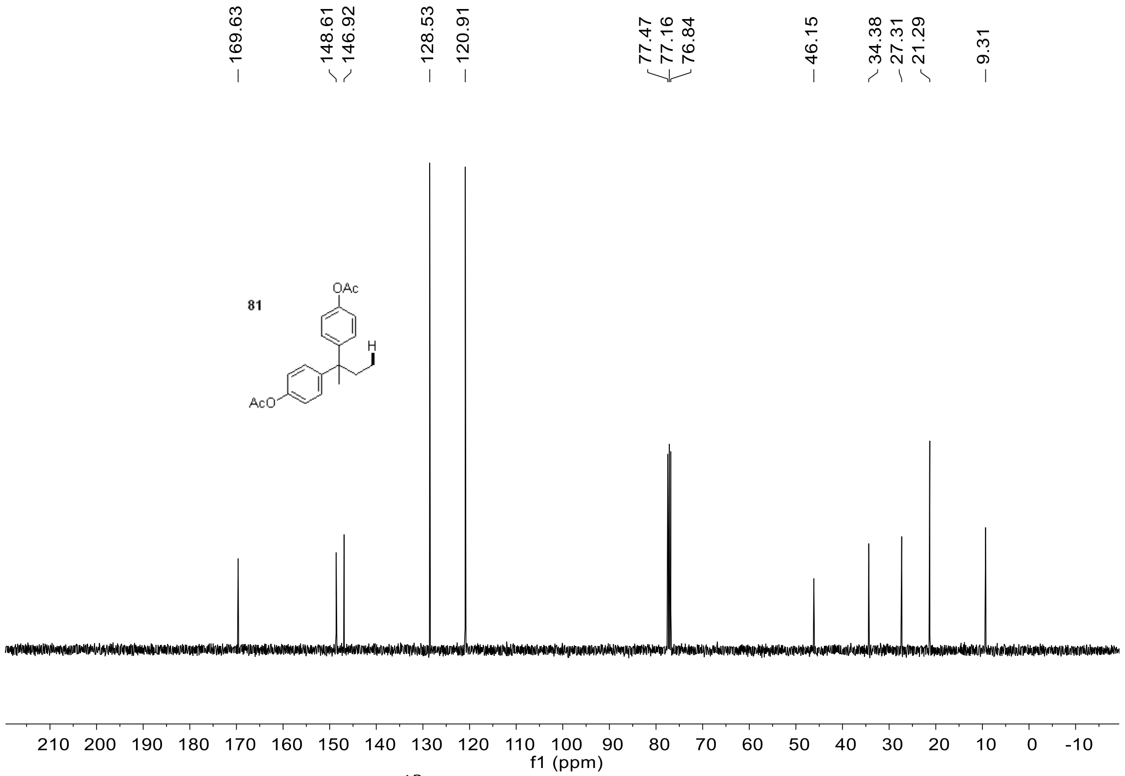

Supplementary Figure 198. ^{13}C NMR Spectra of compound **81**.

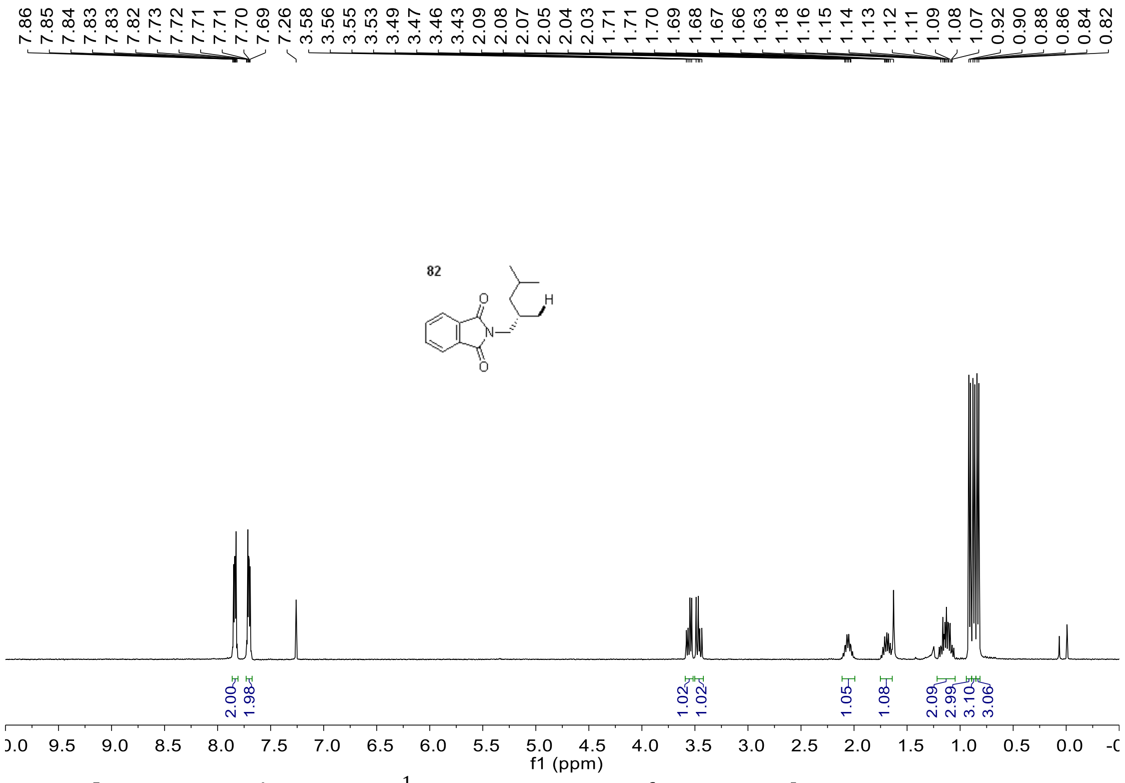

Supplementary Figure 199. ^{1}H NMR Spectra of compound **82**.

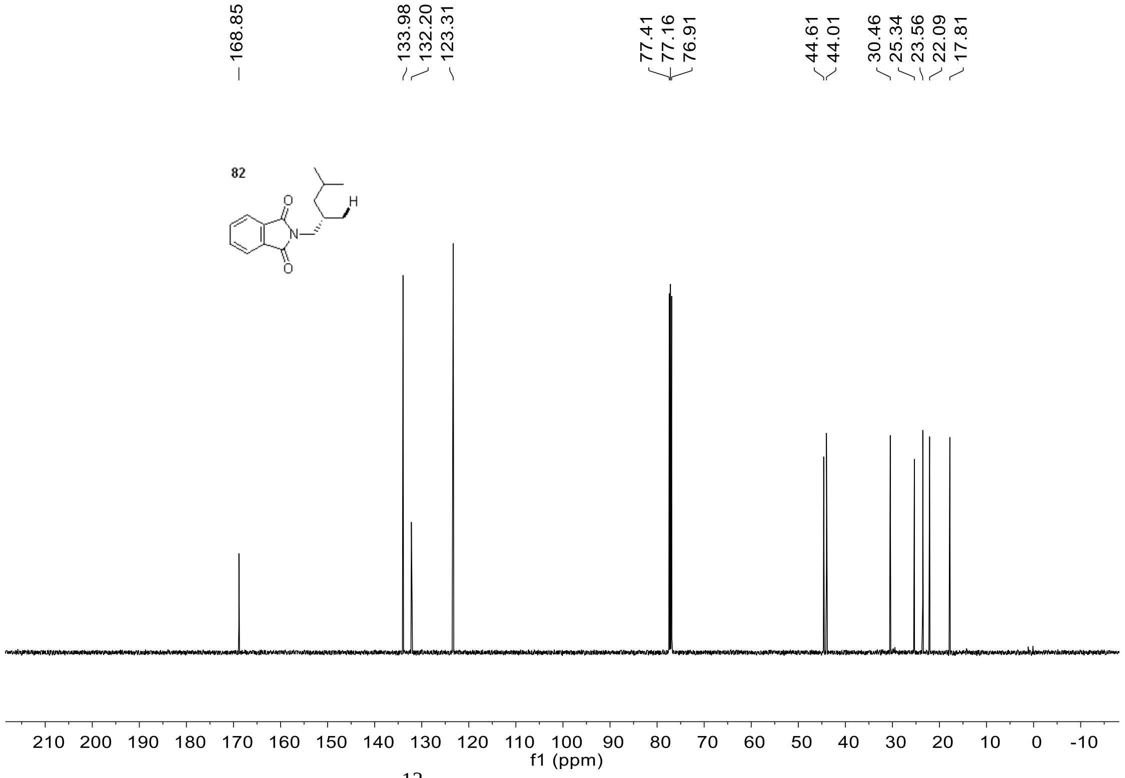

Supplementary Figure 200. ^{13}C NMR Spectra of compound **82**.

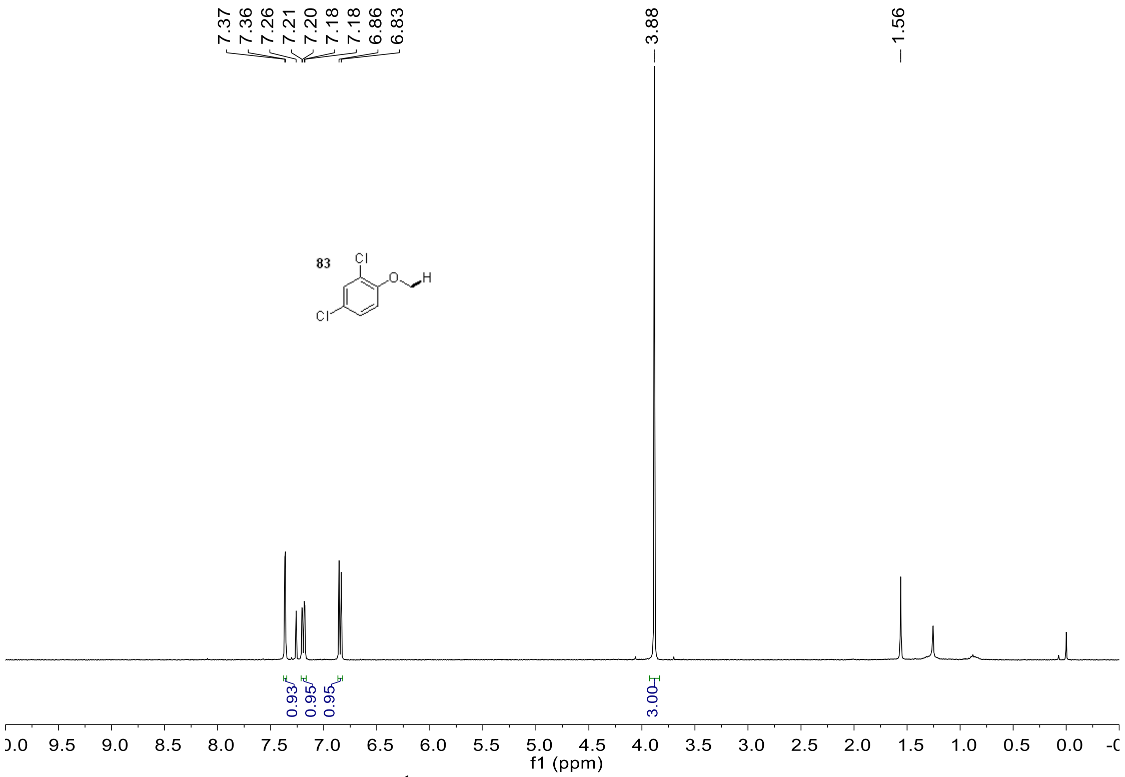

Supplementary Figure 201. ^{1}H NMR Spectra of compound **83**.

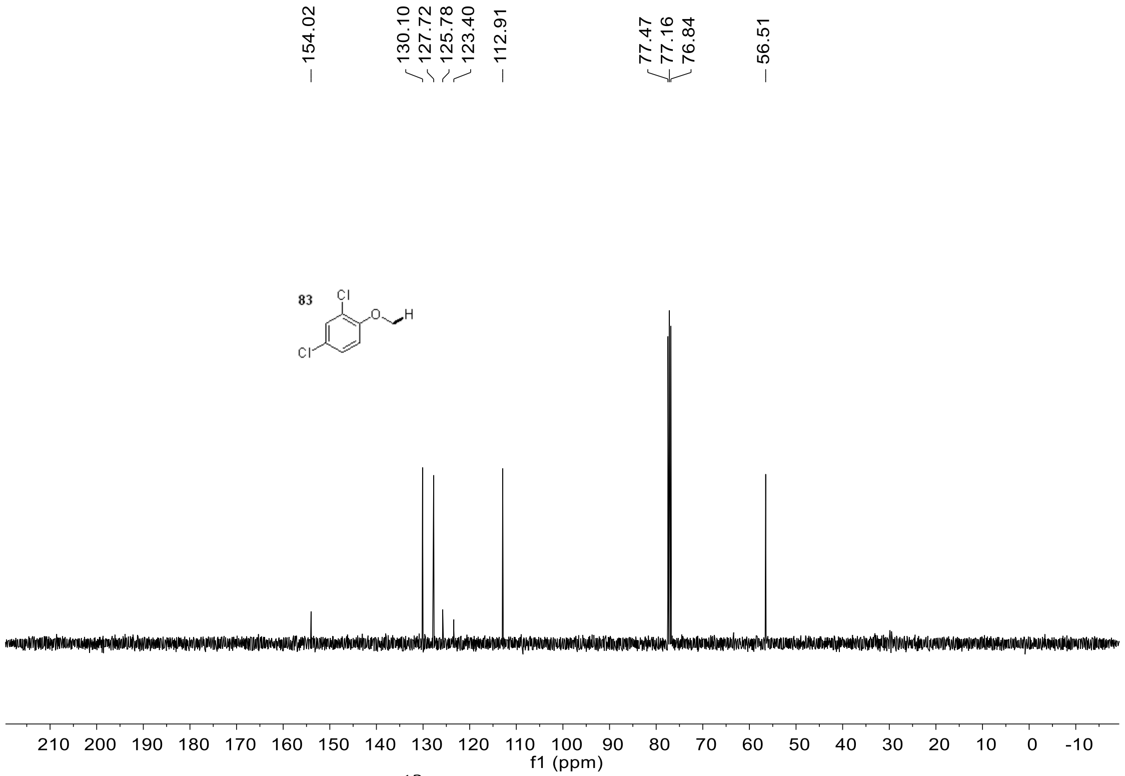

Supplementary Figure 202. ^{13}C NMR Spectra of compound **83**.

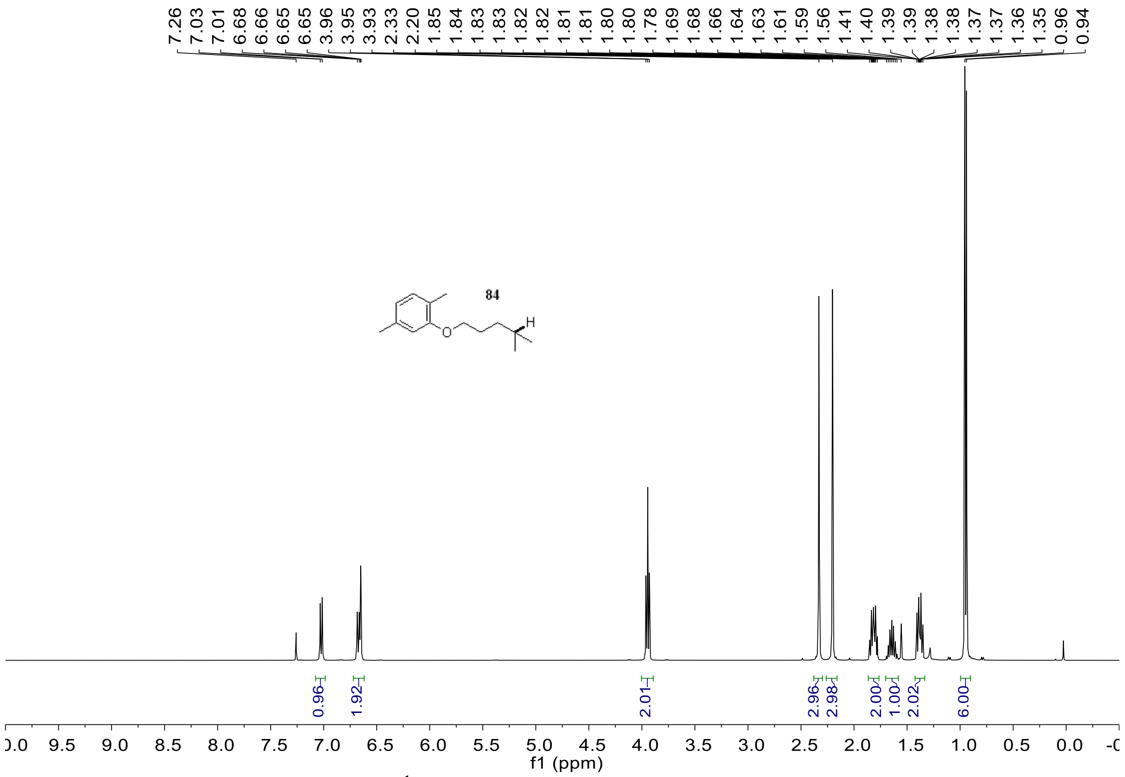

Supplementary Figure 203. ^{1}H NMR Spectra of compound **84**.

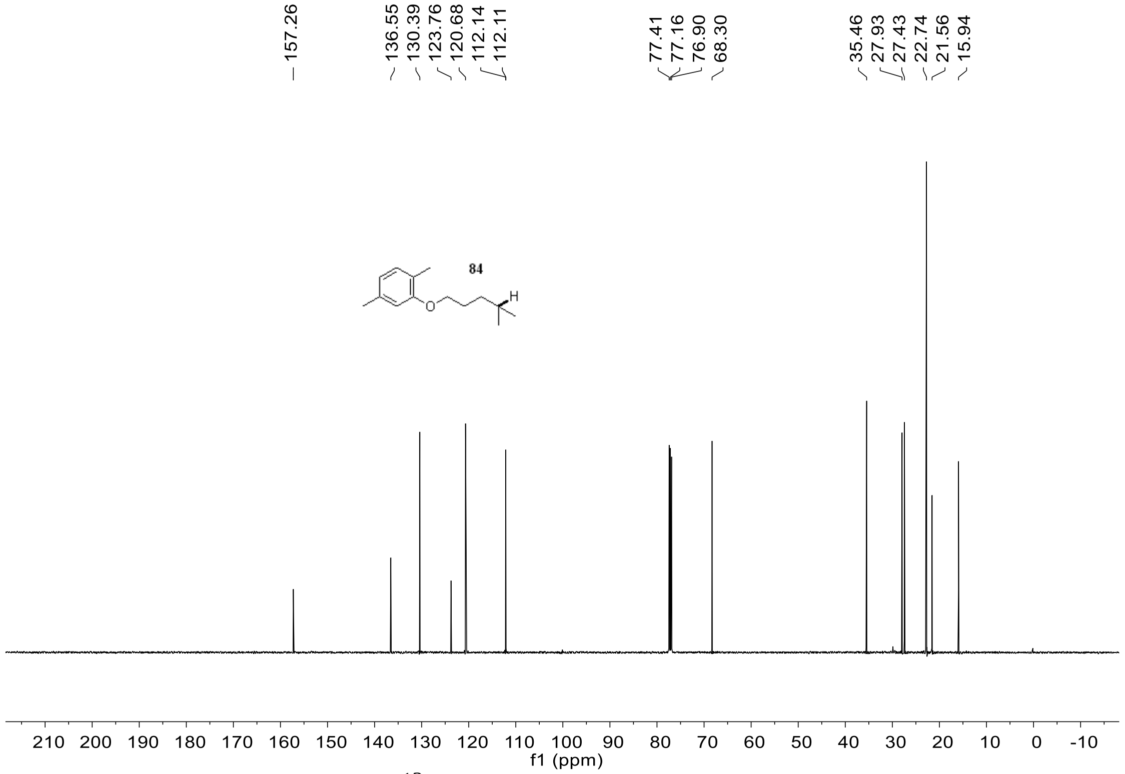

Supplementary Figure 204. ^{13}C NMR Spectra of compound **84**.

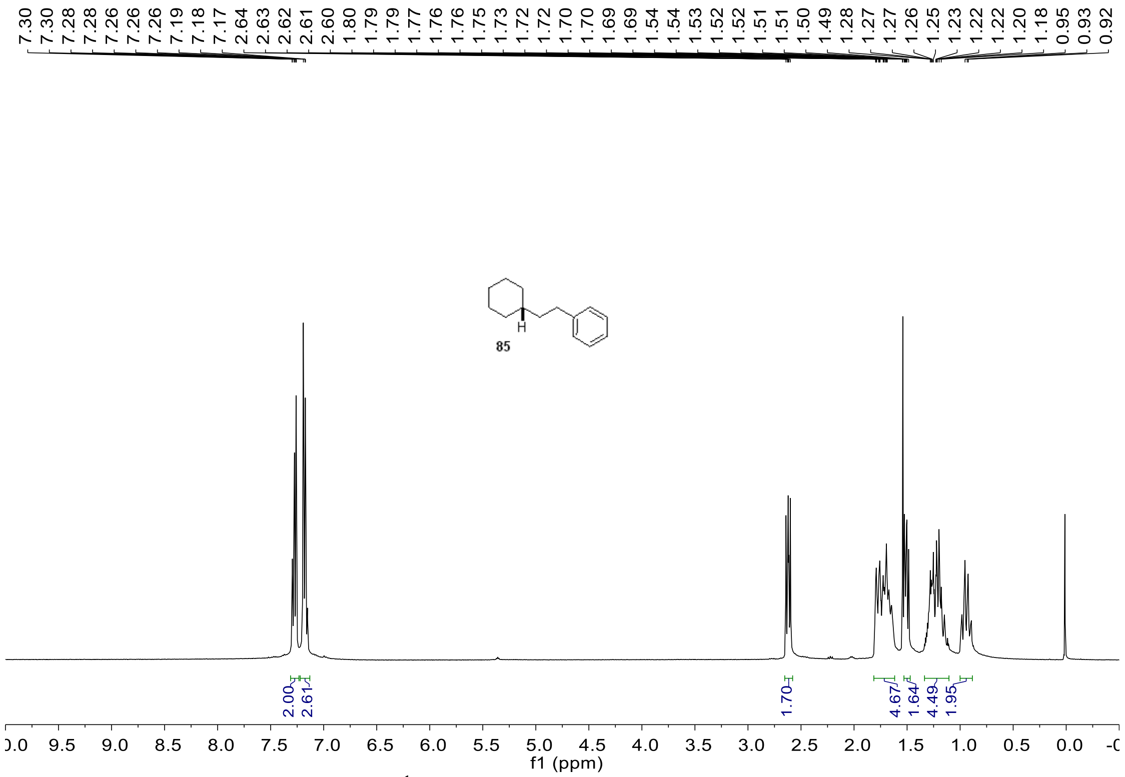

Supplementary Figure 205. ^{1}H NMR Spectra of compound **85**.

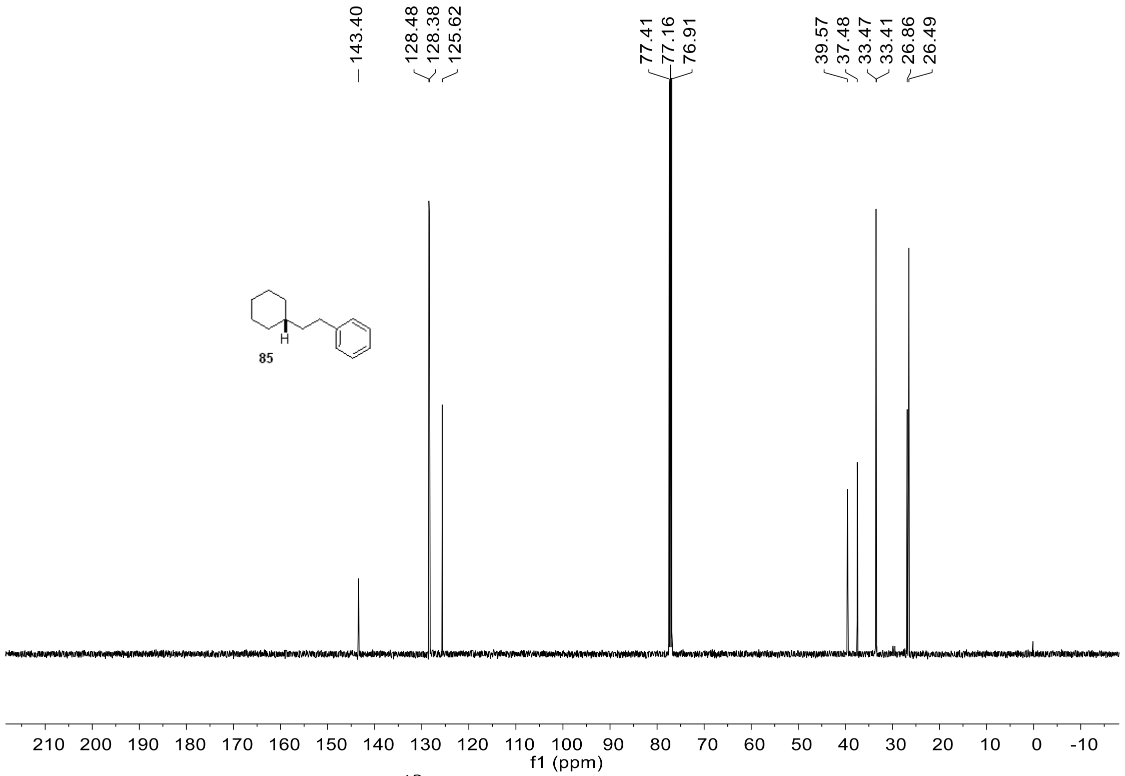

Supplementary Figure 206. ^{13}C NMR Spectra of compound **85**.

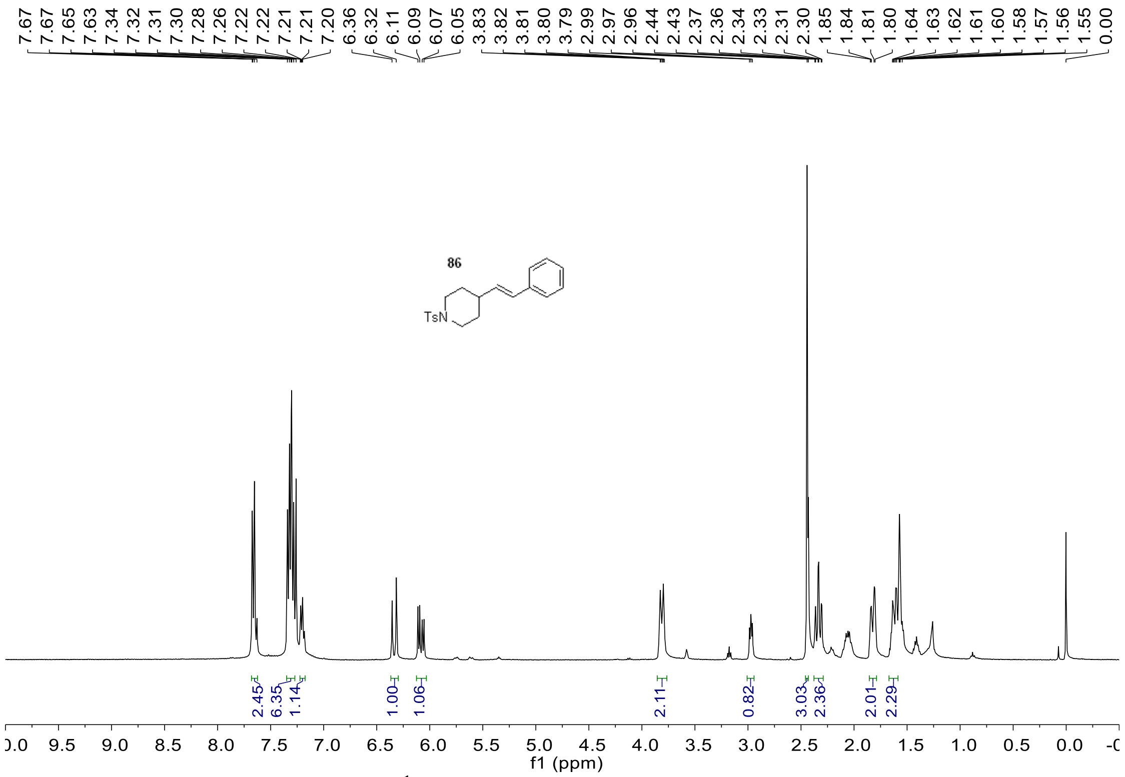

Supplementary Figure 207. ^{1}H NMR Spectra of compound **86**.

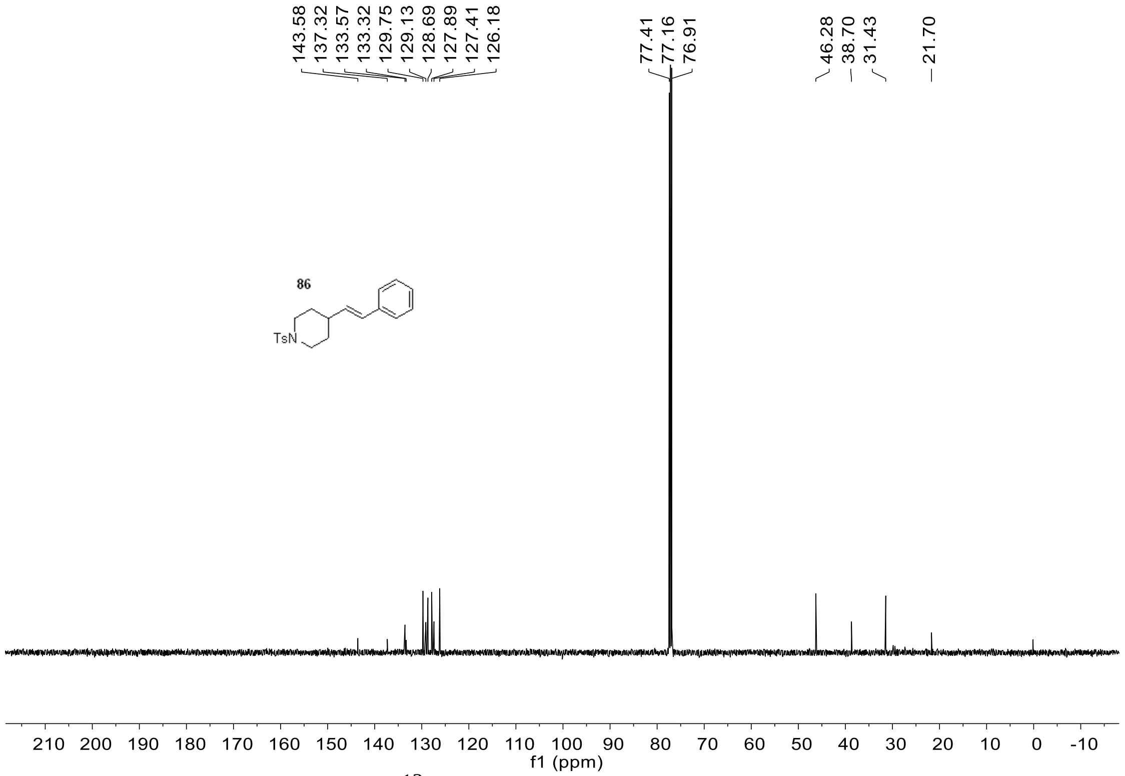

Supplementary Figure 208. ^{13}C NMR Spectra of compound **86**.

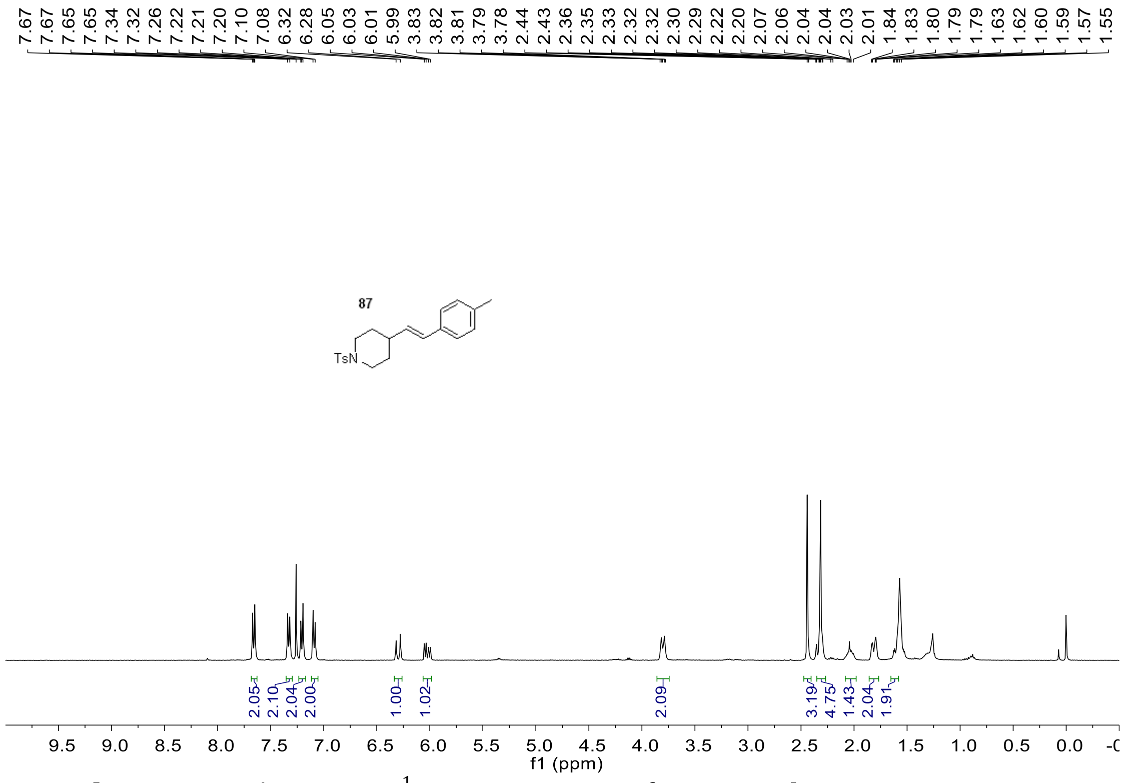

Supplementary Figure 209. ^{1}H NMR Spectra of compound **87**.

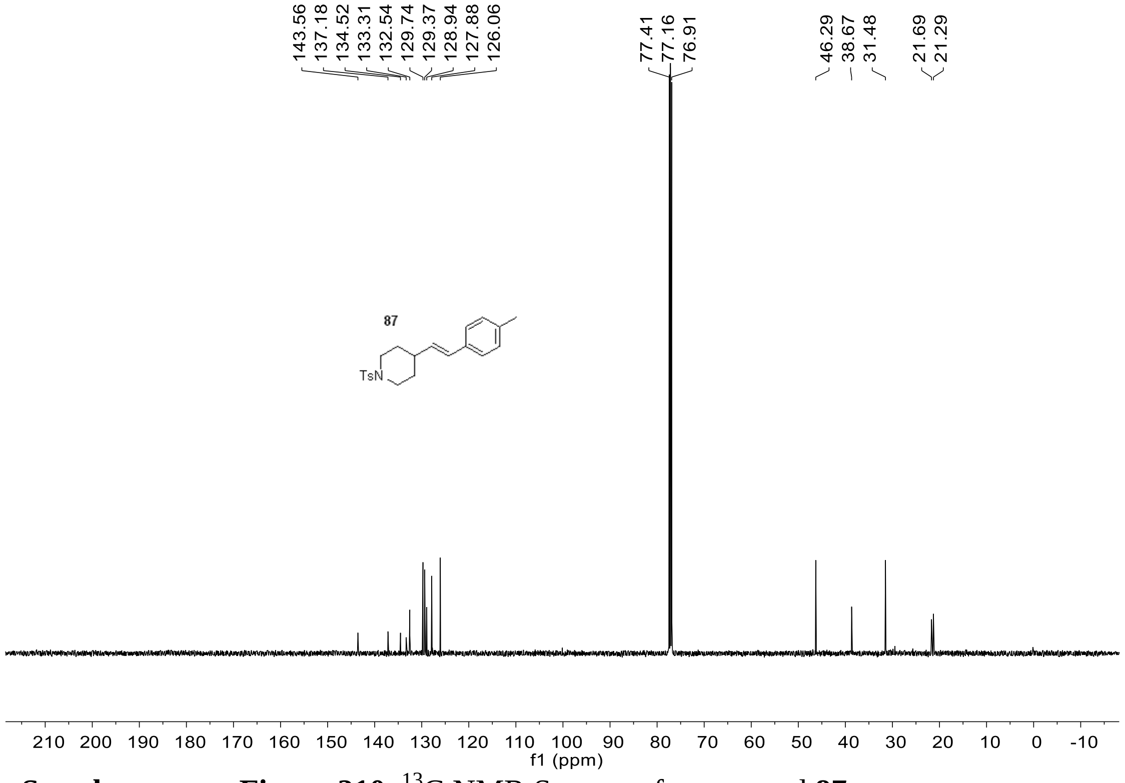

Supplementary Figure 210. ^{13}C NMR Spectra of compound **87**.

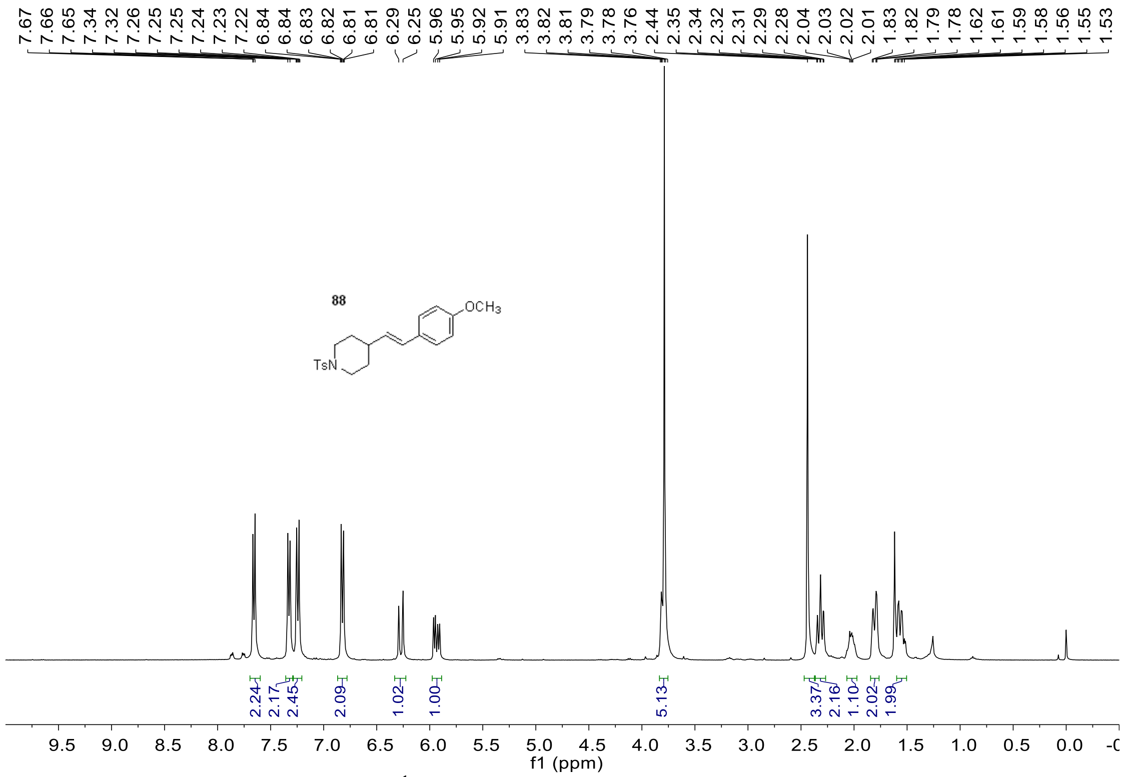

Supplementary Figure 211. ^{1}H NMR Spectra of compound **88**.

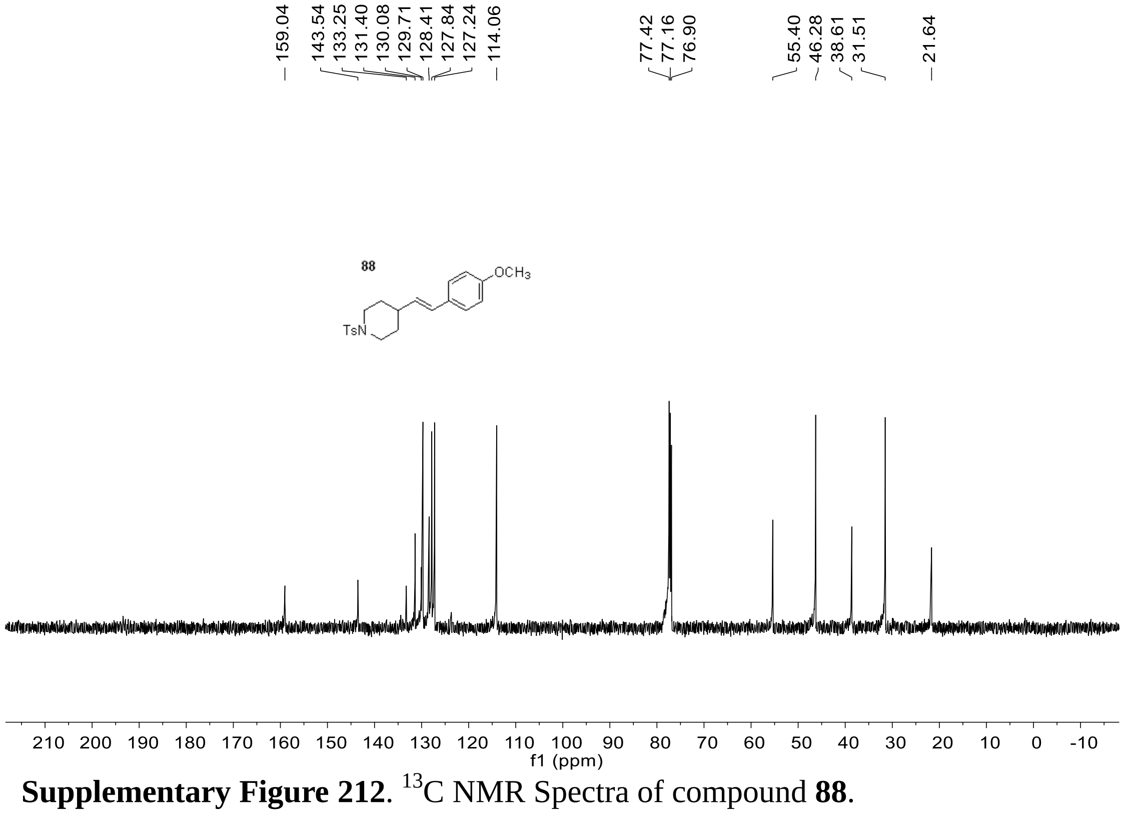

Supplementary Figure 212. ^{13}C NMR Spectra of compound **88**.

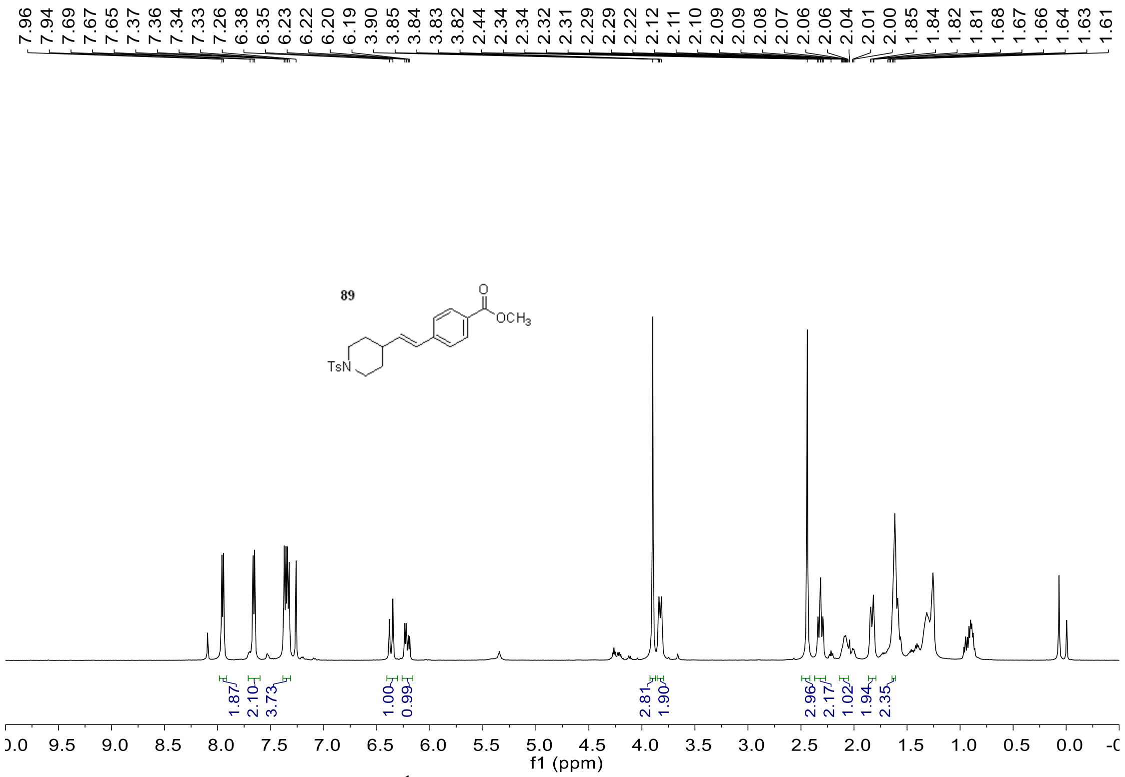

Supplementary Figure 213. ^{1}H NMR Spectra of compound **89**.

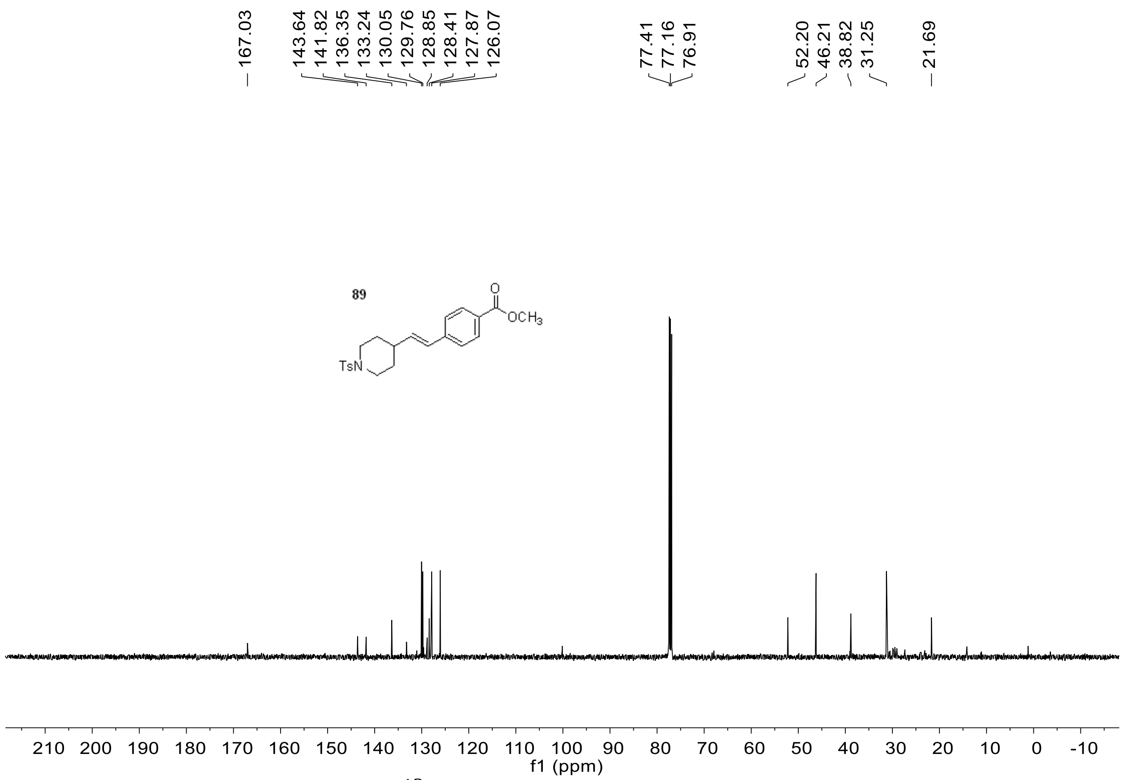

Supplementary Figure 214. ^{13}C NMR Spectra of compound **89**.

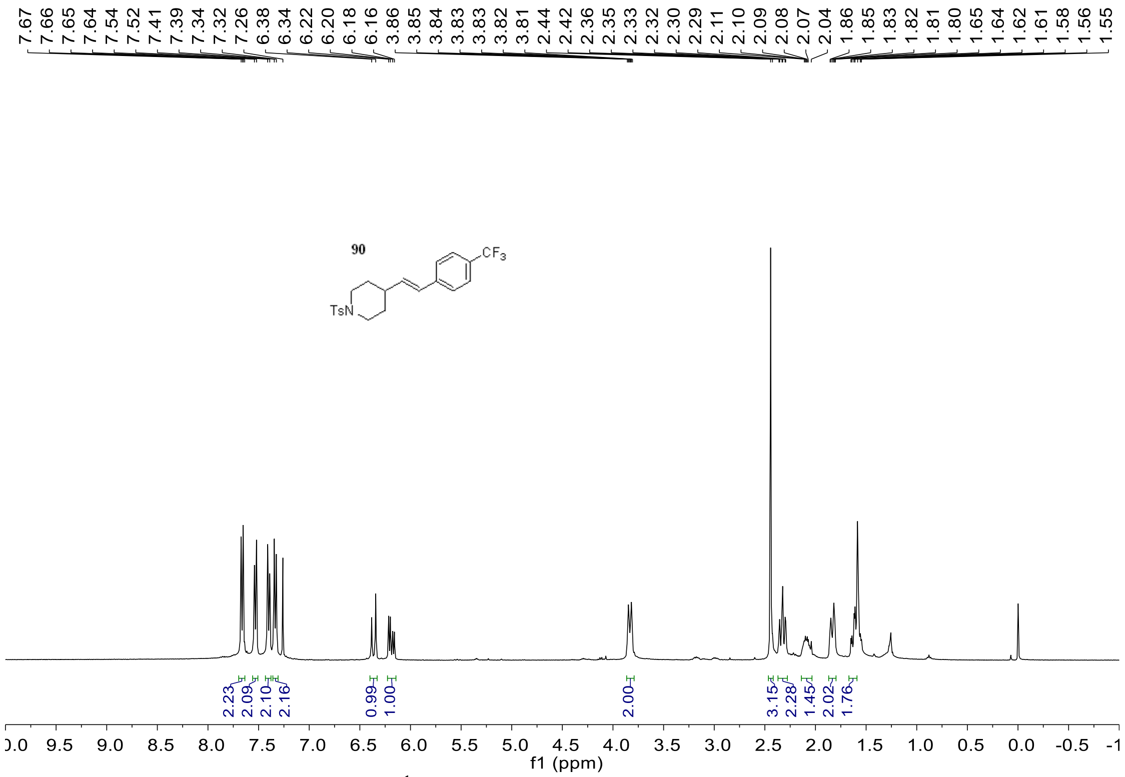

Supplementary Figure 215. ^{1}H NMR Spectra of compound **90**.

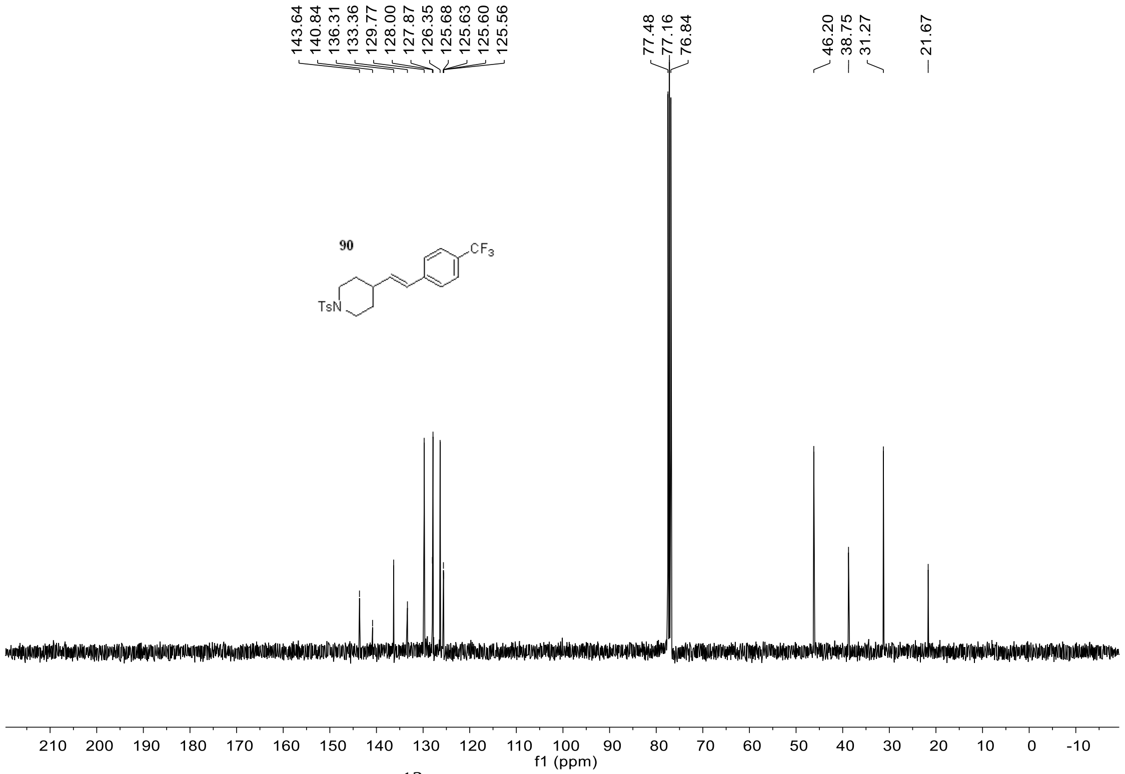

Supplementary Figure 216. ^{13}C NMR Spectra of compound **90**.

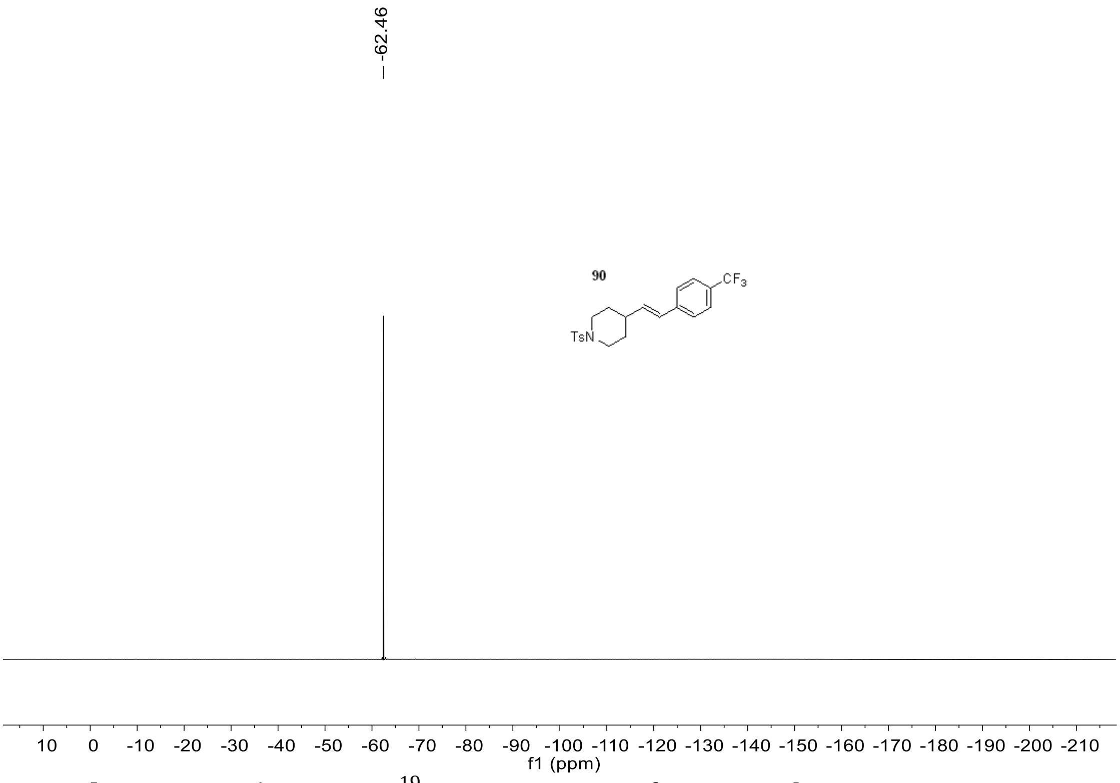

Supplementary Figure 217. ^{19}F NMR Spectra of compound **90**.

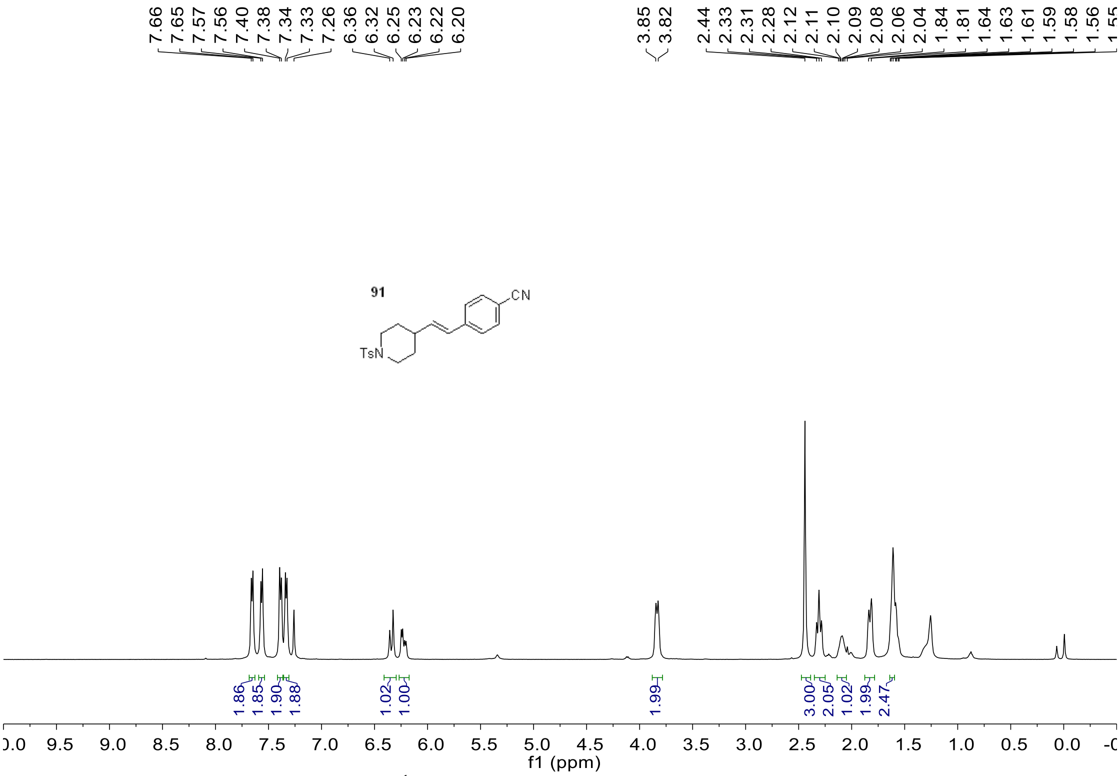

Supplementary Figure 218. ^{1}H NMR Spectra of compound **91**.

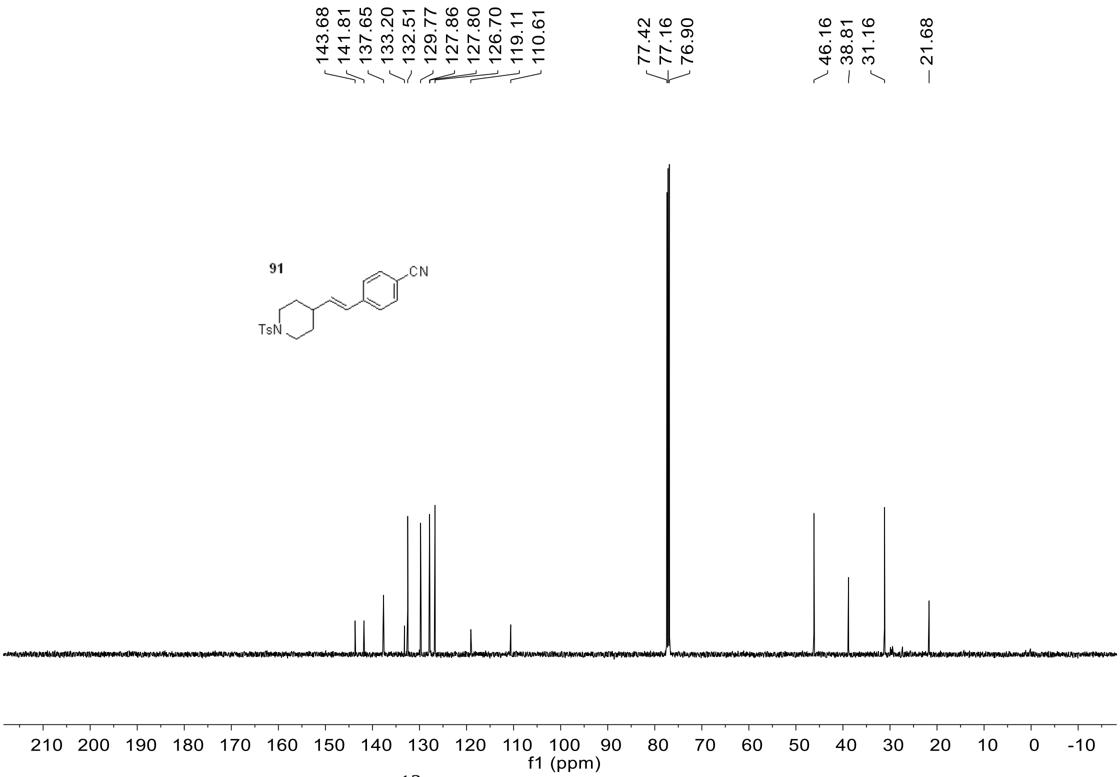

Supplementary Figure 219. ^{13}C NMR Spectra of compound **91**.

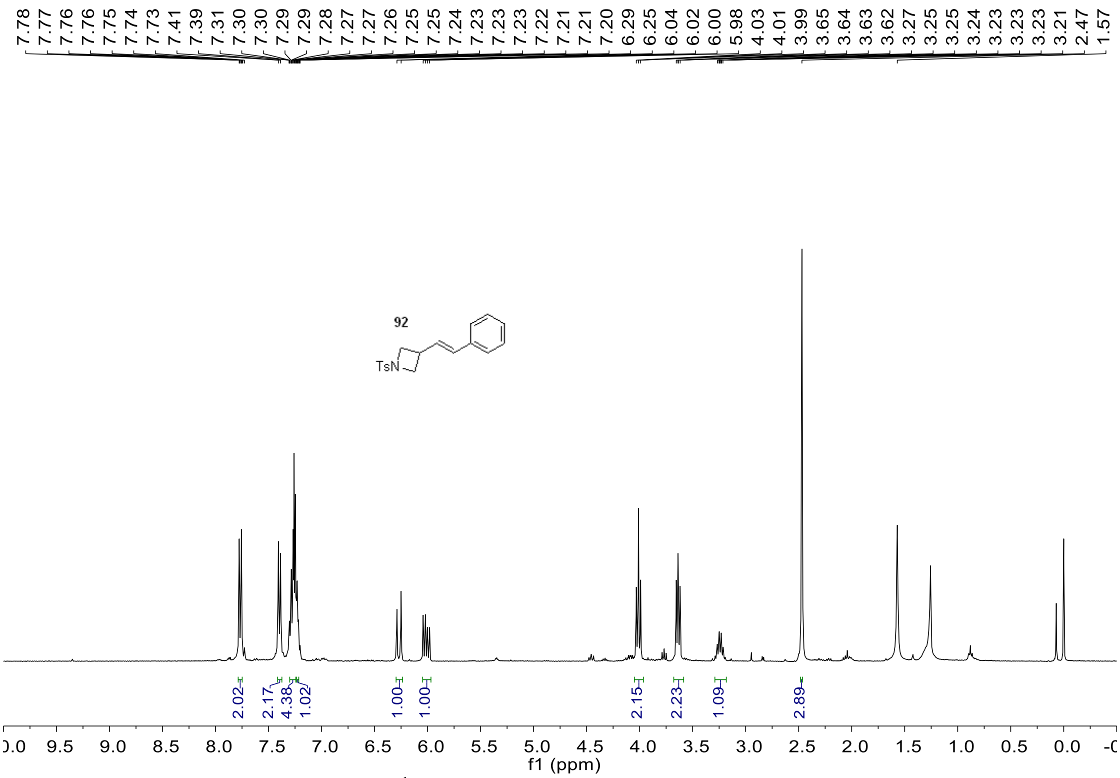

Supplementary Figure 220. ^{1}H NMR Spectra of compound **92**.

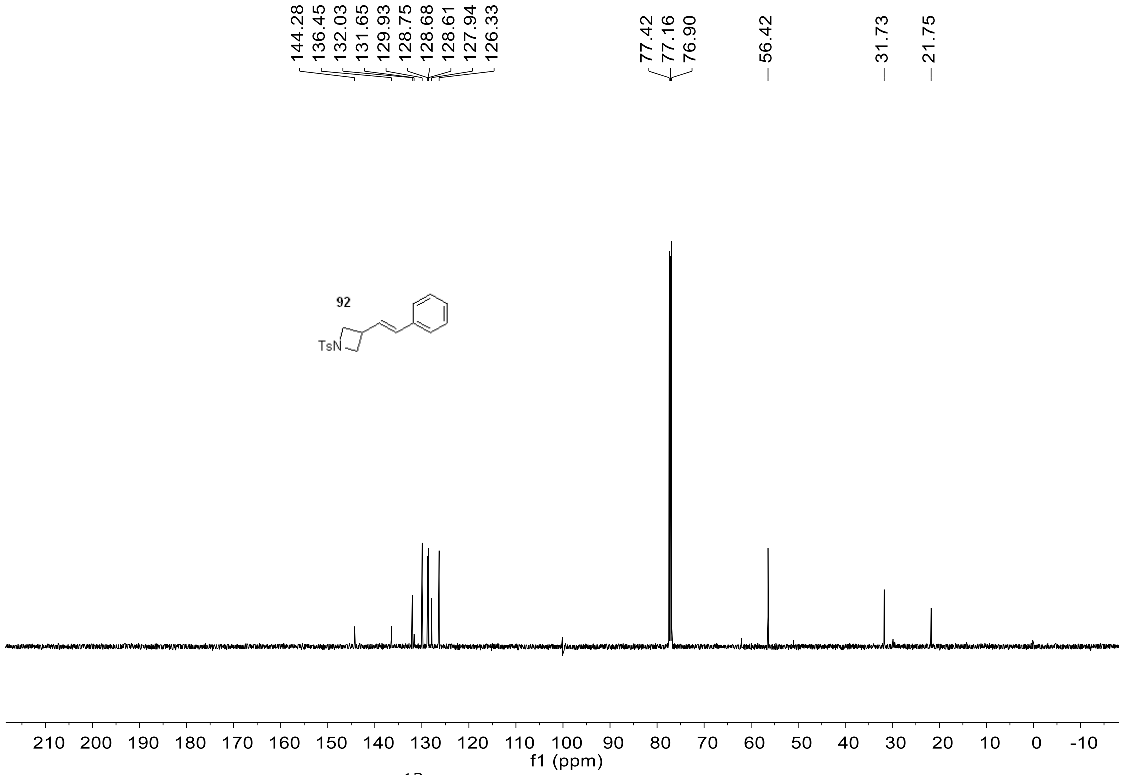

Supplementary Figure 221. ^{13}C NMR Spectra of compound **92**.

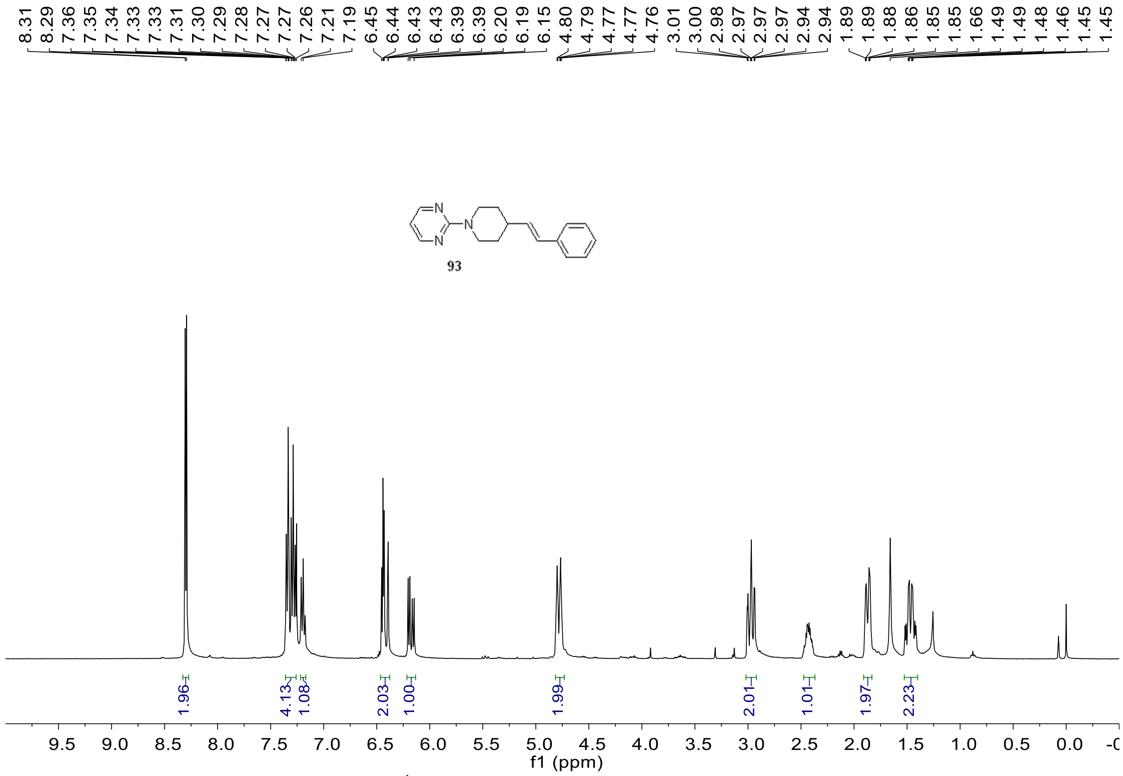

Supplementary Figure 222. ^{1}H NMR Spectra of compound **93**.

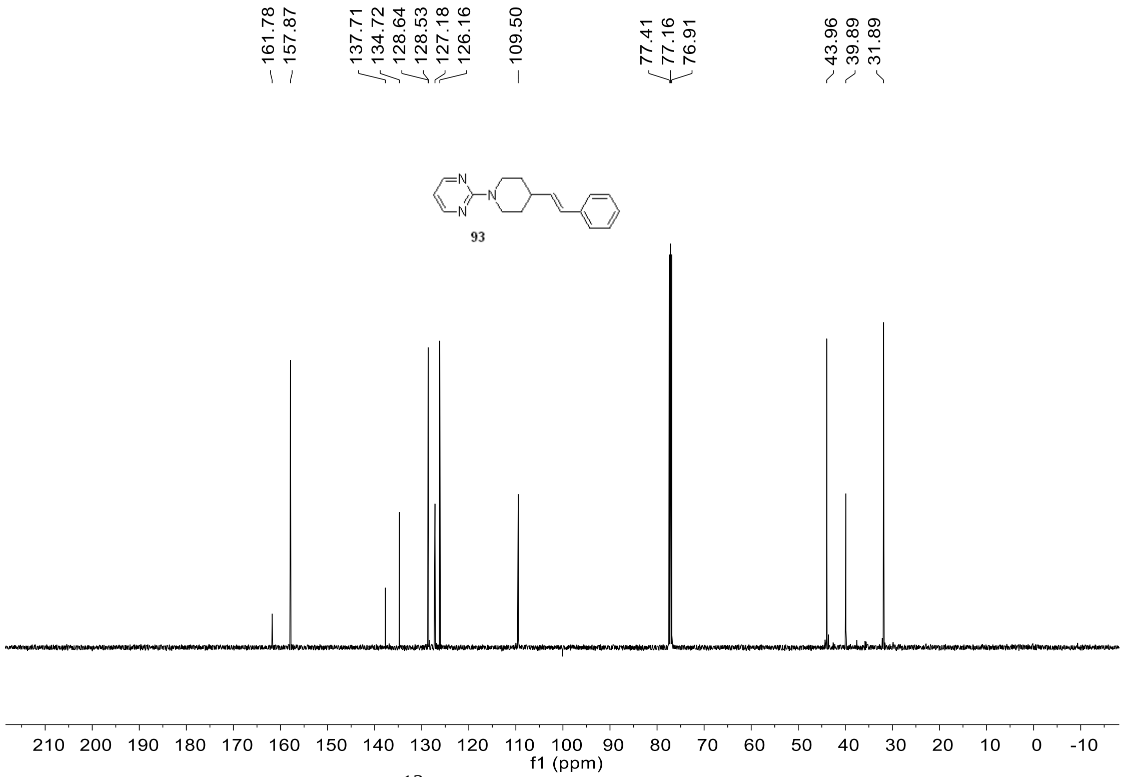

Supplementary Figure 223. ^{13}C NMR Spectra of compound **93**.

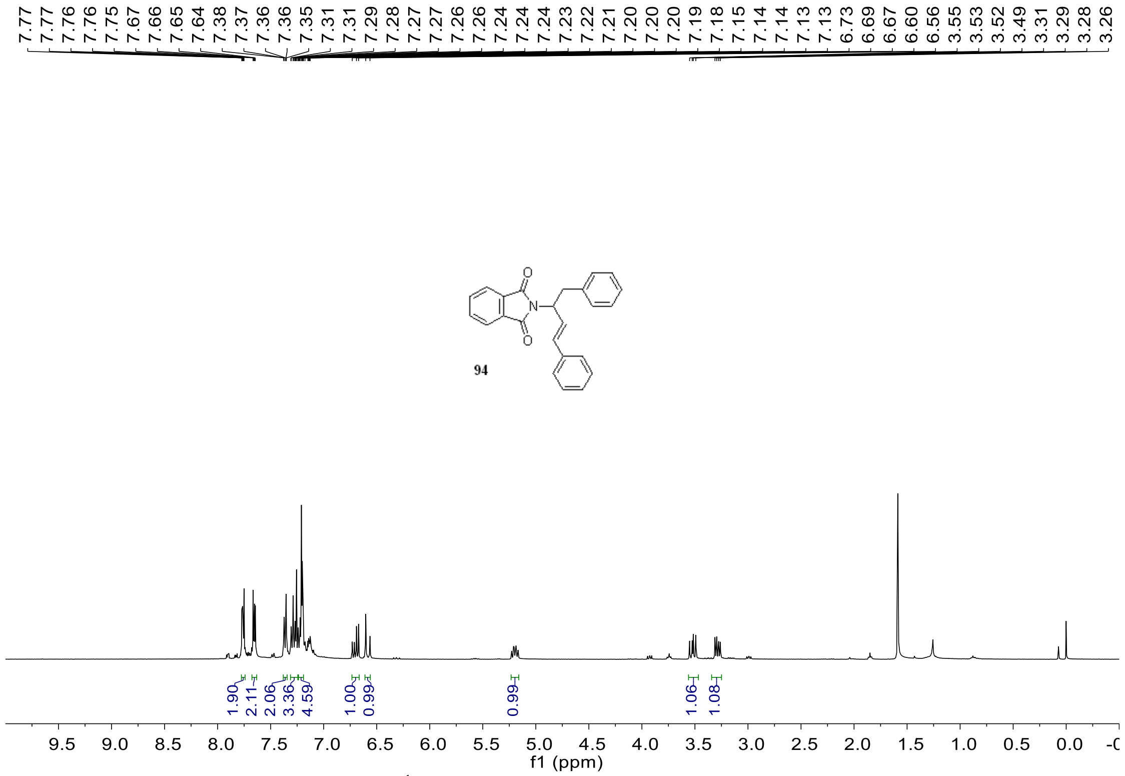

Supplementary Figure 224. ^{1}H NMR Spectra of compound **94**.

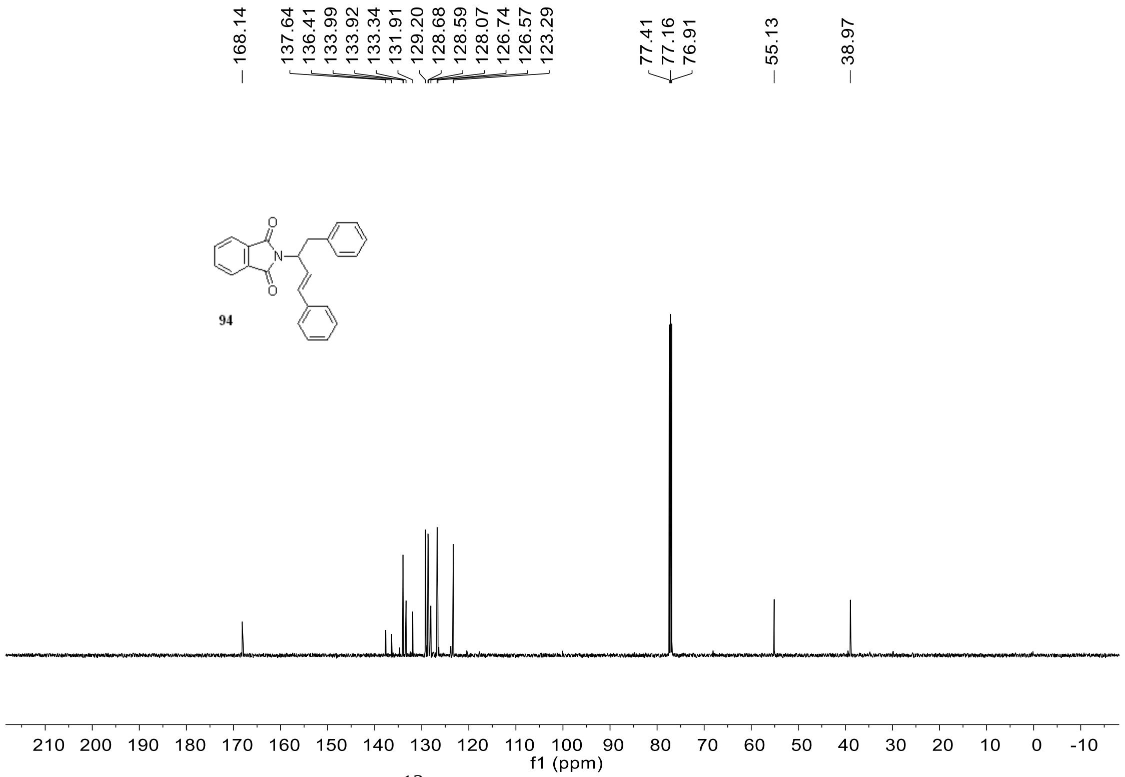

Supplementary Figure 225. ^{13}C NMR Spectra of compound **94**.

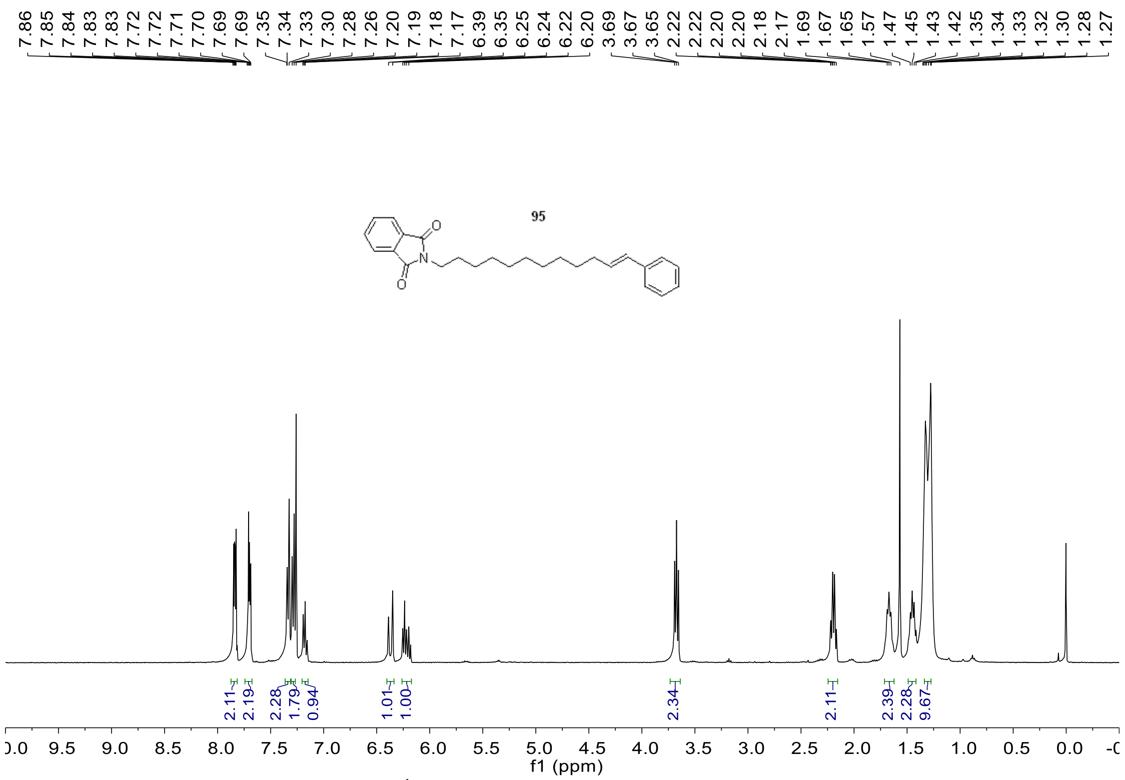

Supplementary Figure 226. ^{1}H NMR Spectra of compound **95**.

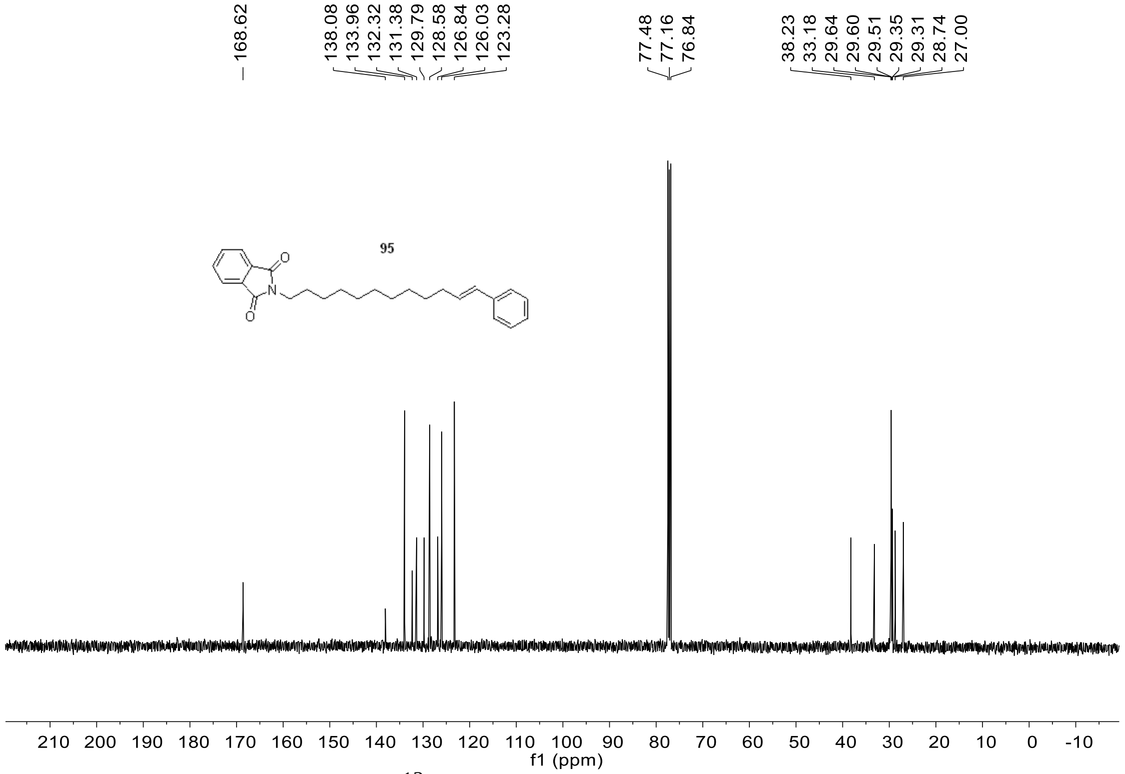

Supplementary Figure 227. ^{13}C NMR Spectra of compound **95**.

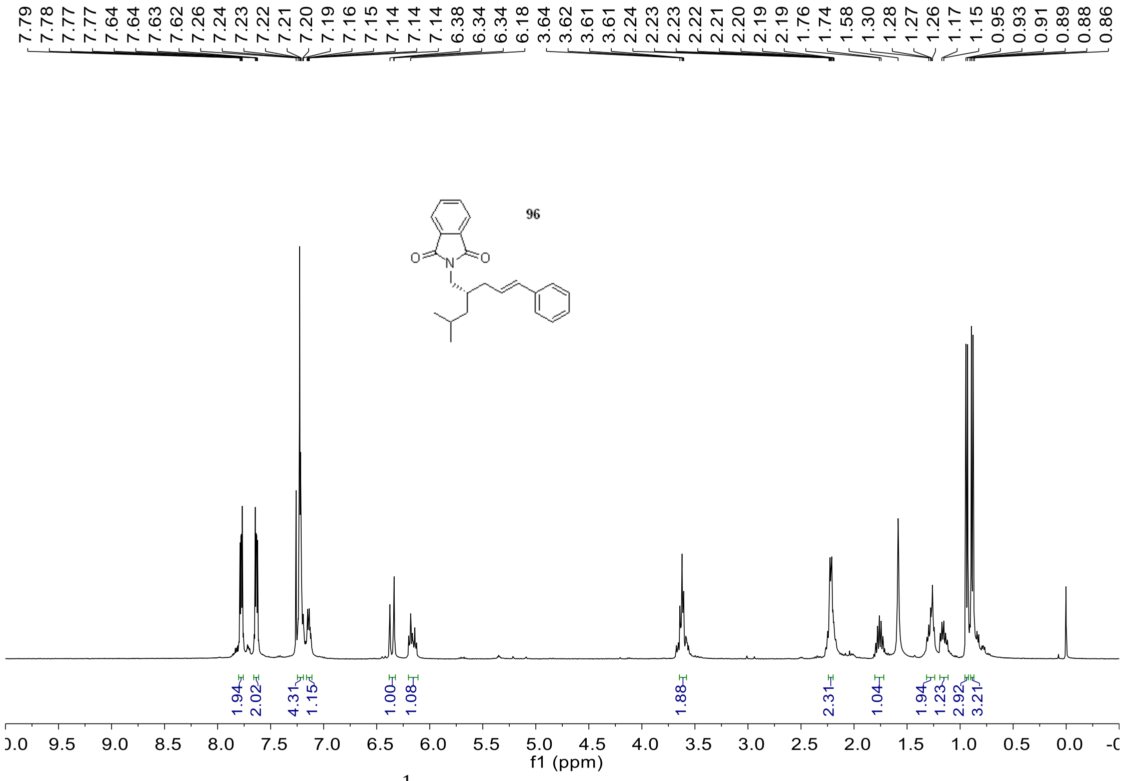

Supplementary Figure 228. ^{1}H NMR Spectra of compound **96**.

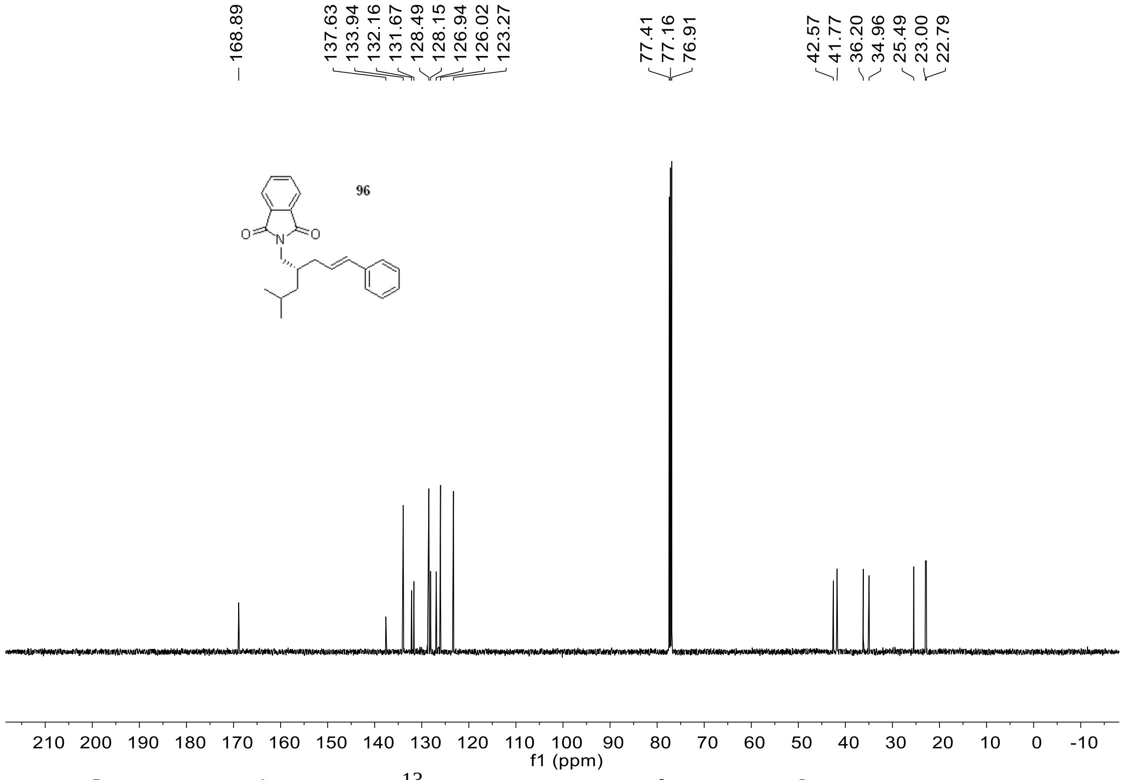

Supplementary Figure 229. ^{13}C NMR Spectra of compound **96**.

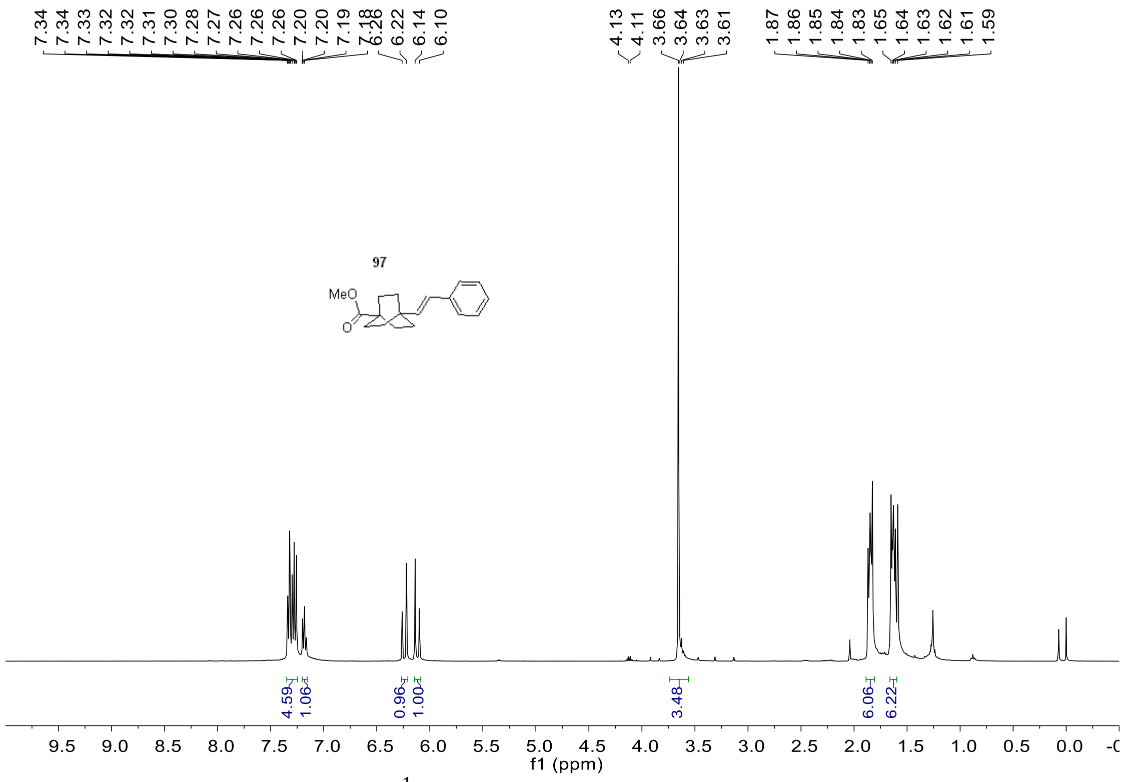

Supplementary Figure 230. ^{1}H NMR Spectra of compound **97**.

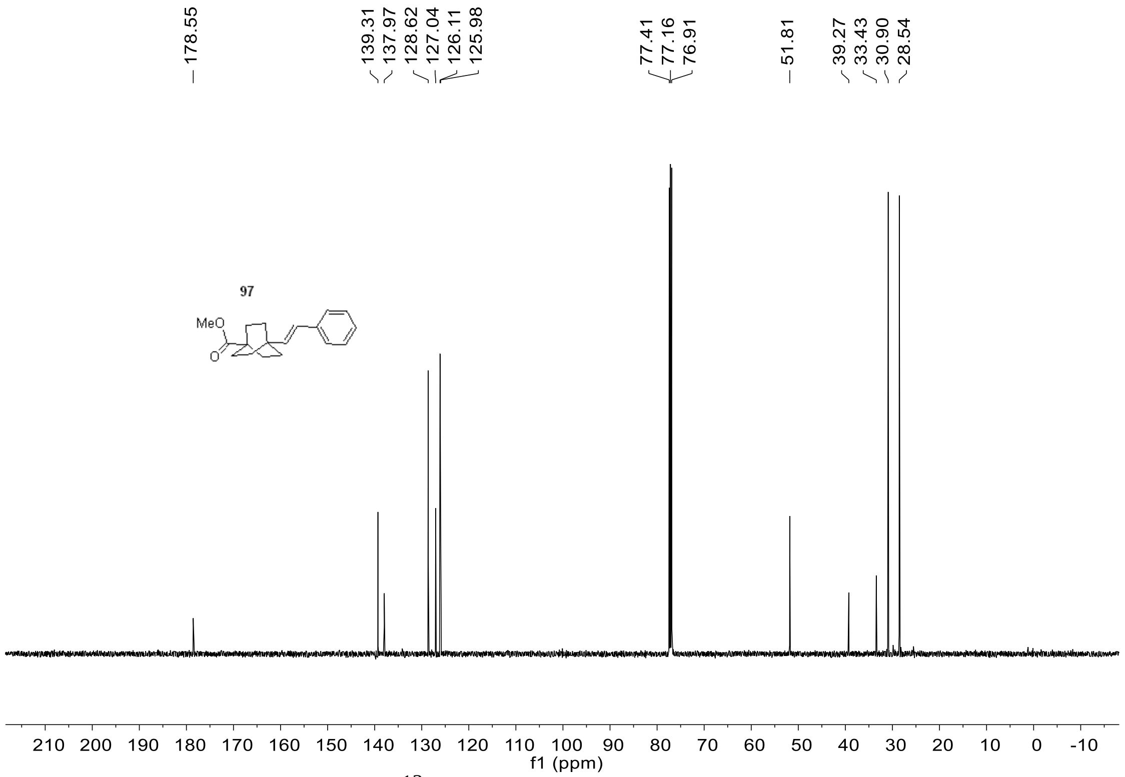

Supplementary Figure 231. ^{13}C NMR Spectra of compound **97**.

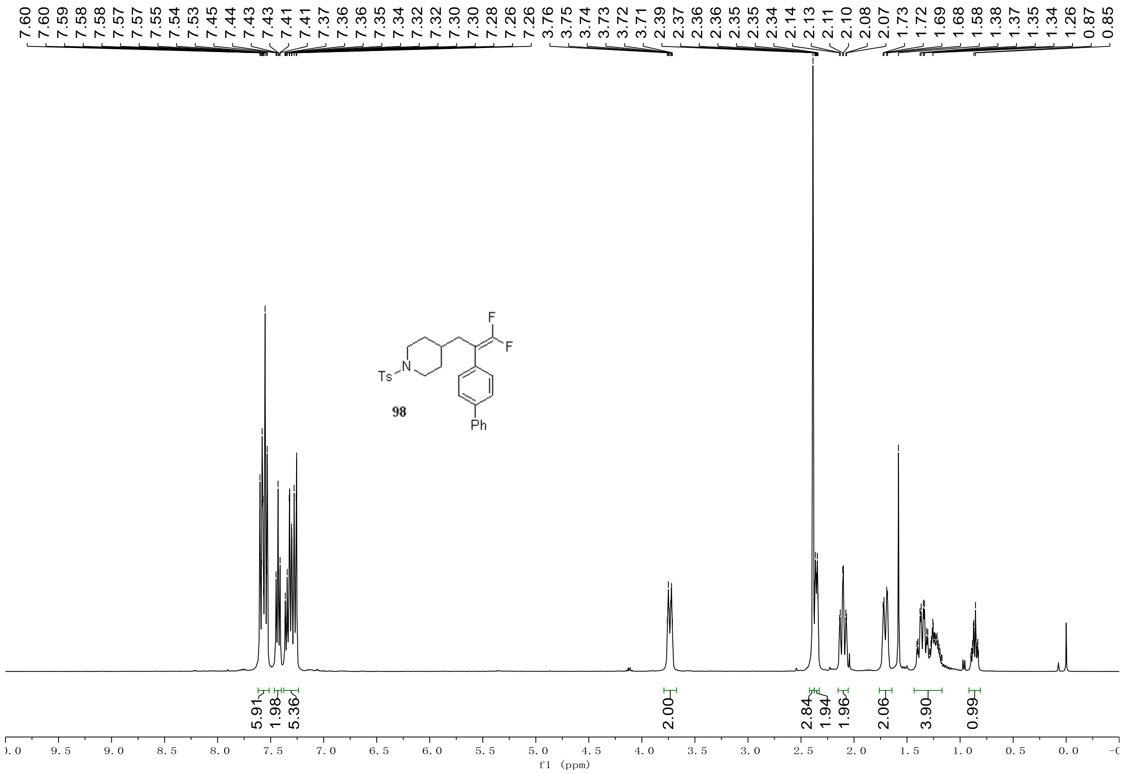

Supplementary Figure 232. ^{1}H NMR Spectra of compound **98**.

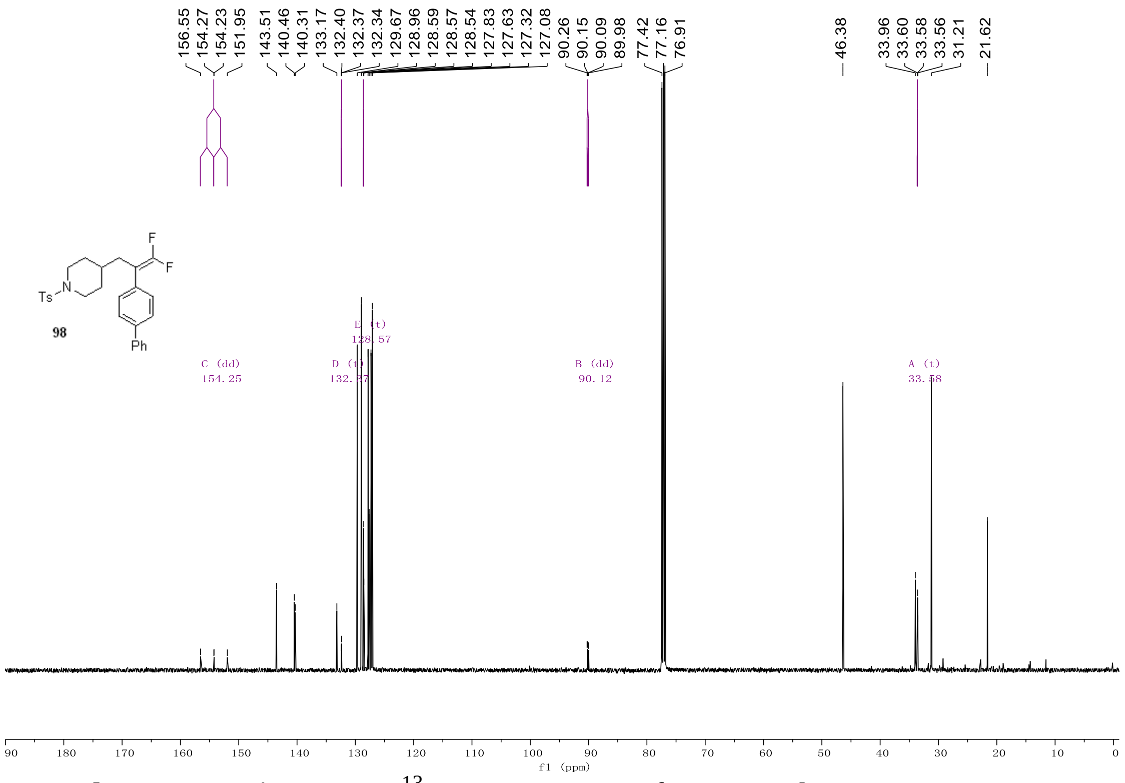

Supplementary Figure 233. ^{13}C NMR Spectra of compound **98**.

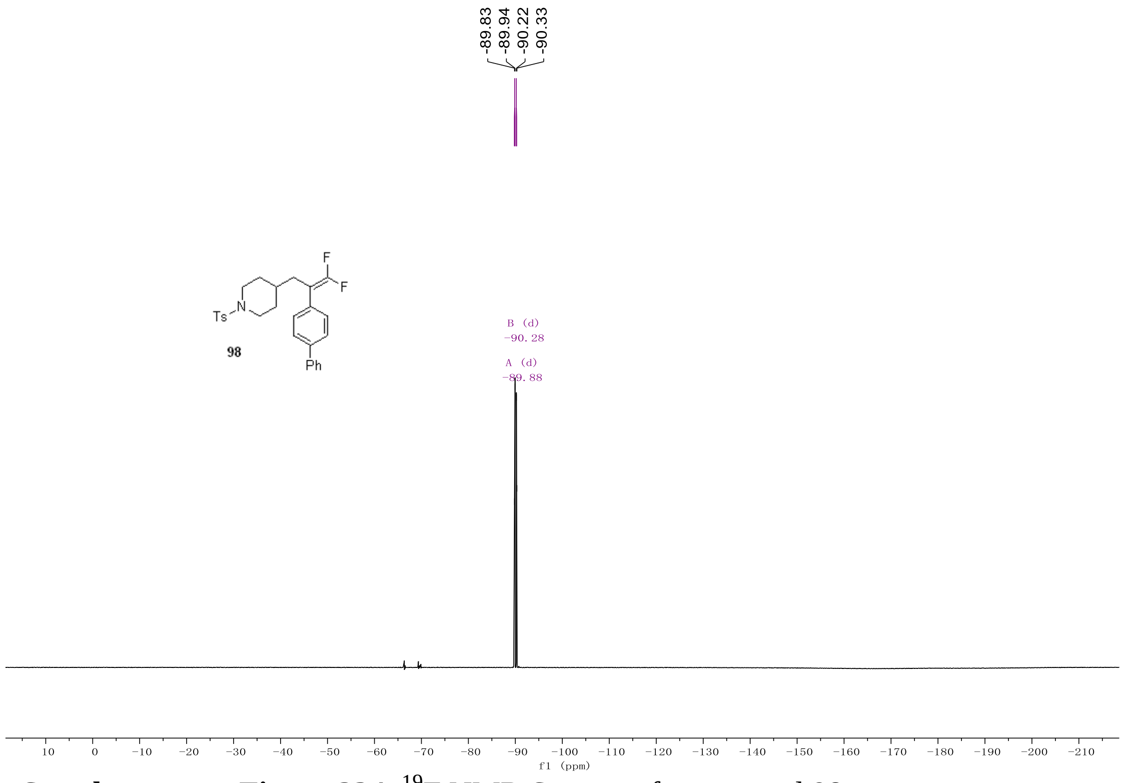

Supplementary Figure 234. ^{19}F NMR Spectra of compound **98**.

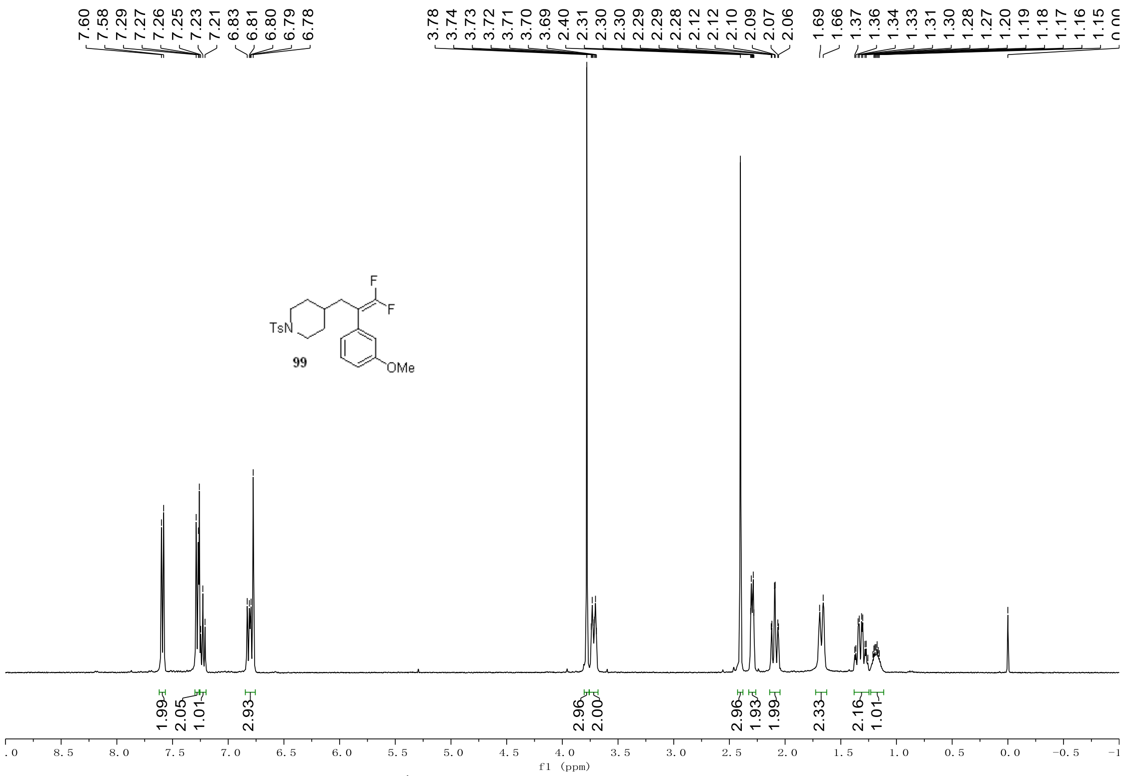

Supplementary Figure 235. ^{1}H NMR Spectra of compound **99**.

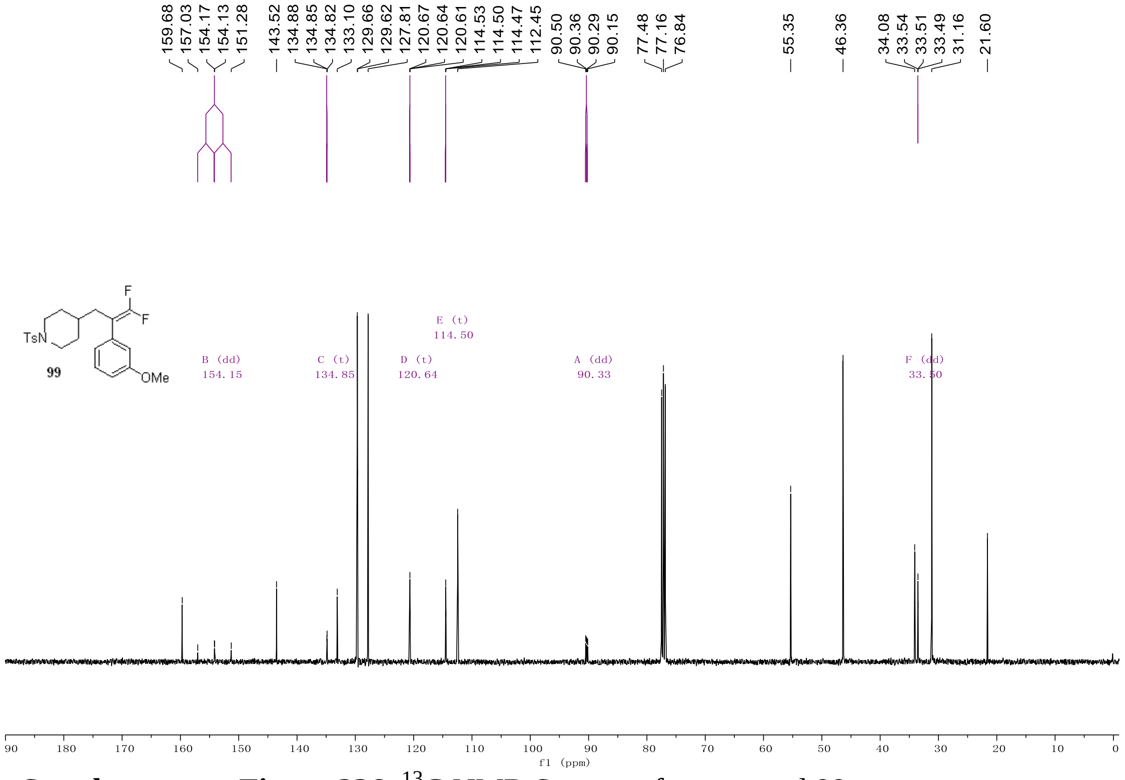

Supplementary Figure 236. ^{13}C NMR Spectra of compound **99**.

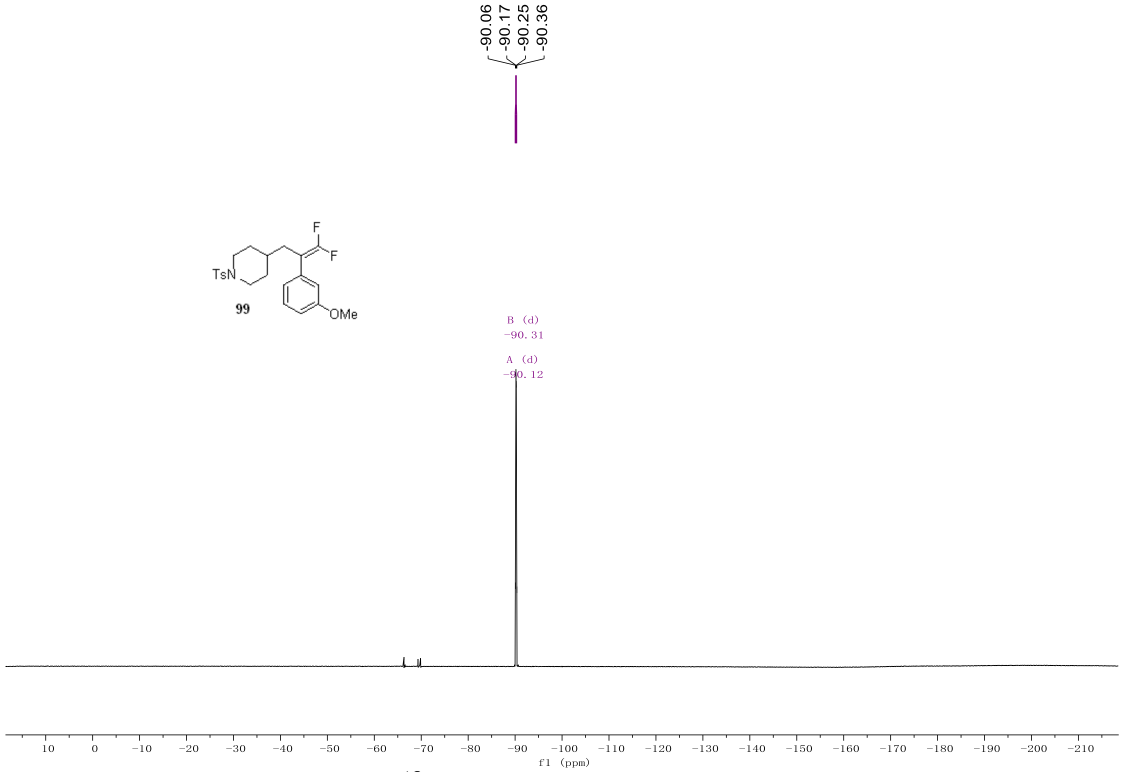

Supplementary Figure 237. ^{19}F NMR Spectra of compound **99**.

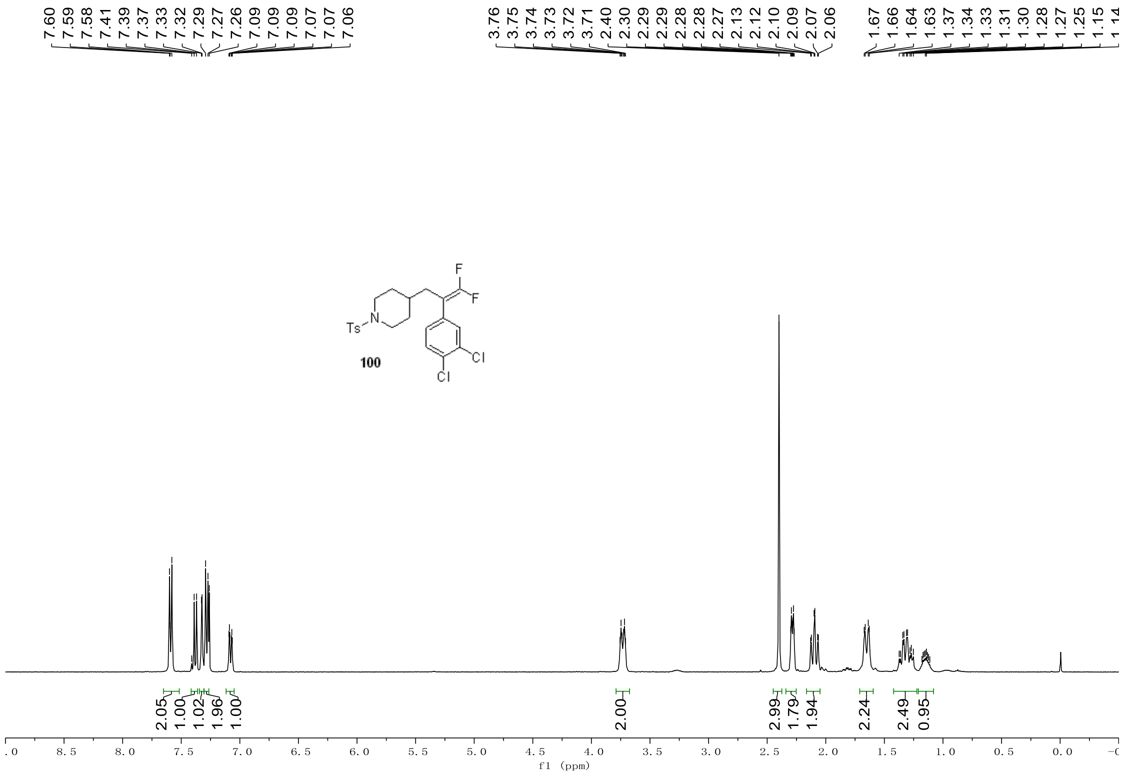

Supplementary Figure 238. ^{1}H NMR Spectra of compound **100**.

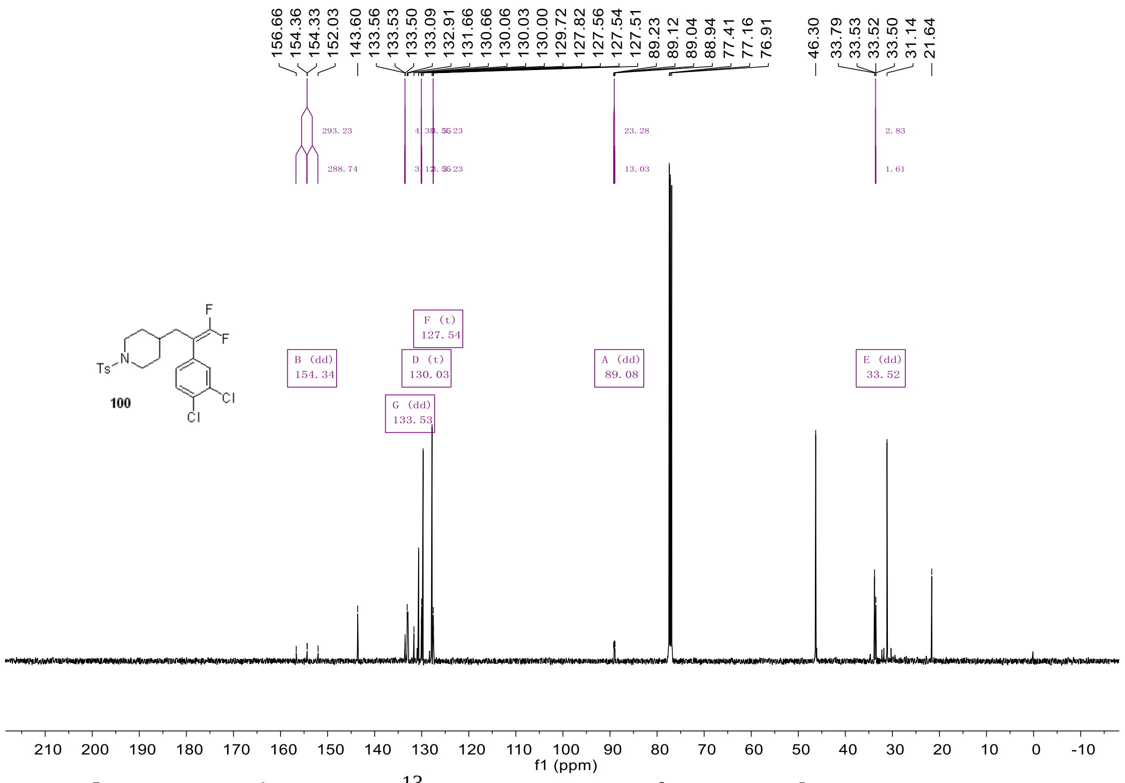

Supplementary Figure 239. ^{13}C NMR Spectra of compound **100**.

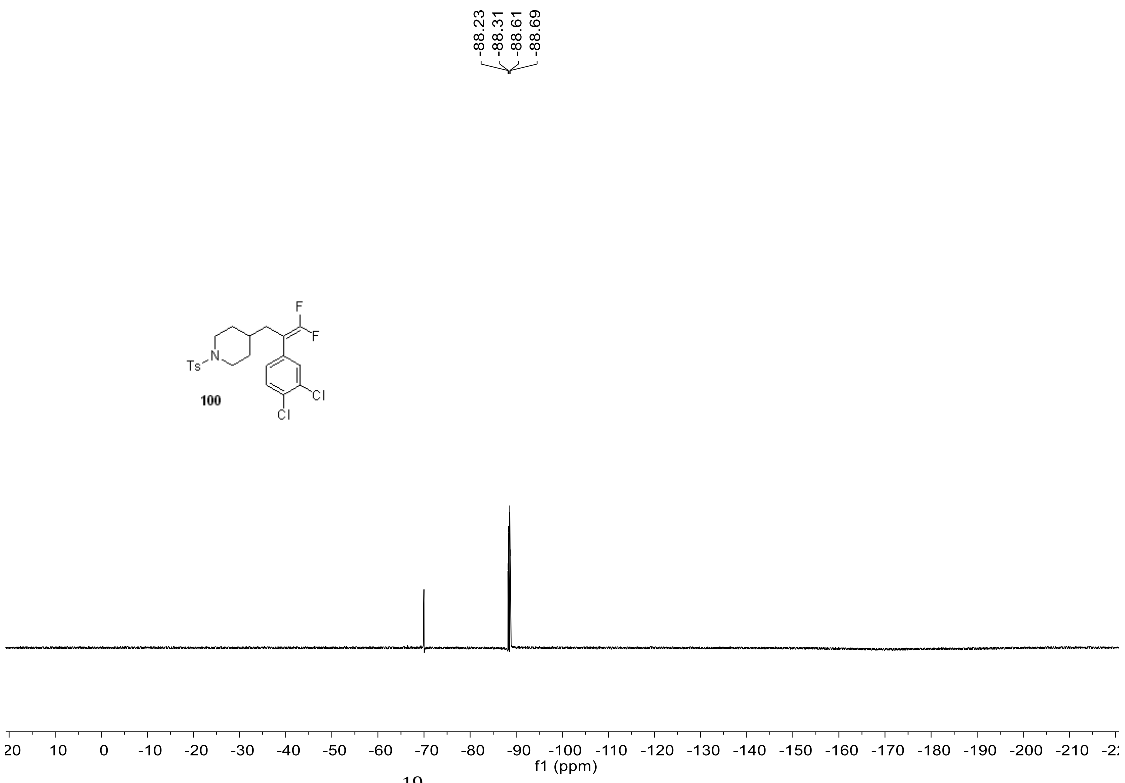

Supplementary Figure 240. ^{19}F NMR Spectra of compound **100**.

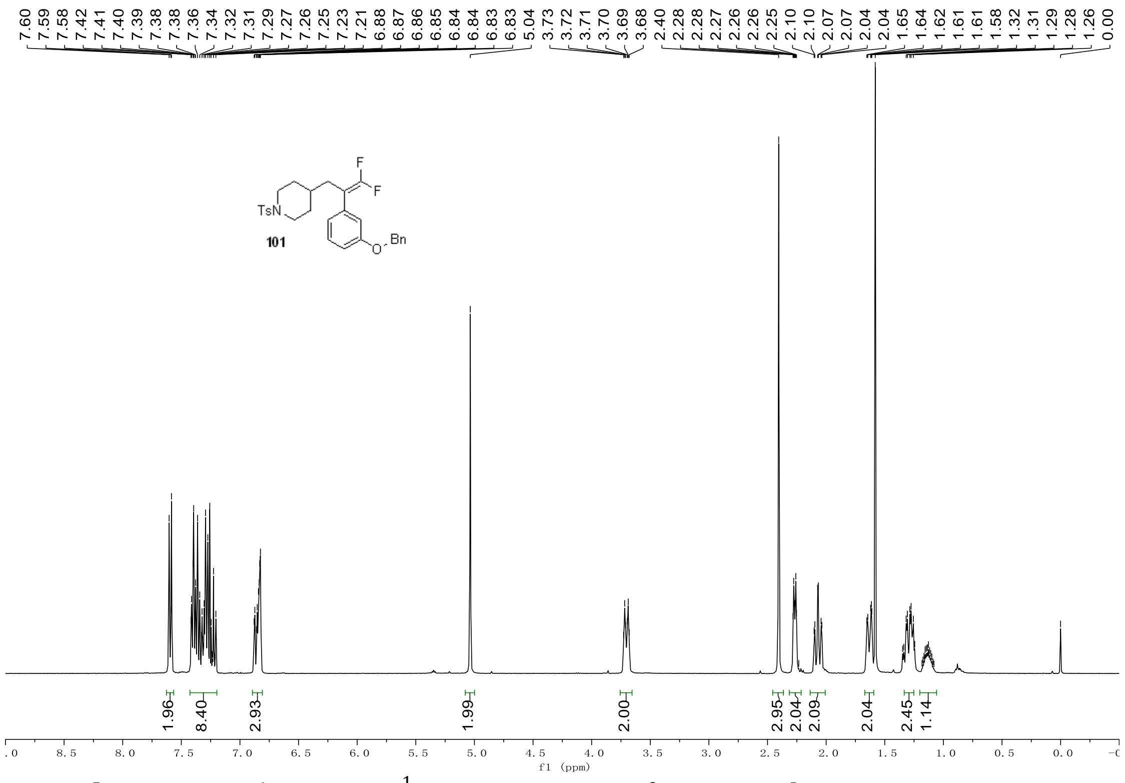

Supplementary Figure 241. ^{1}H NMR Spectra of compound **101**.

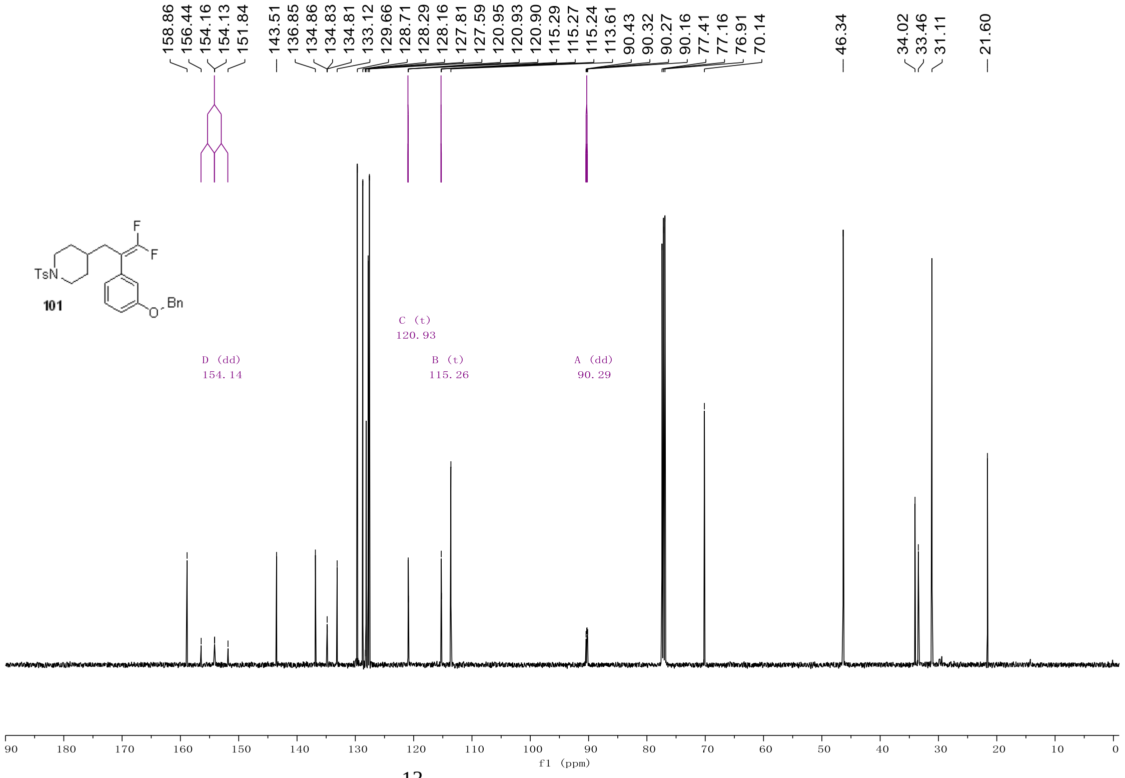

Supplementary Figure 242. ^{13}C NMR Spectra of compound **101**.

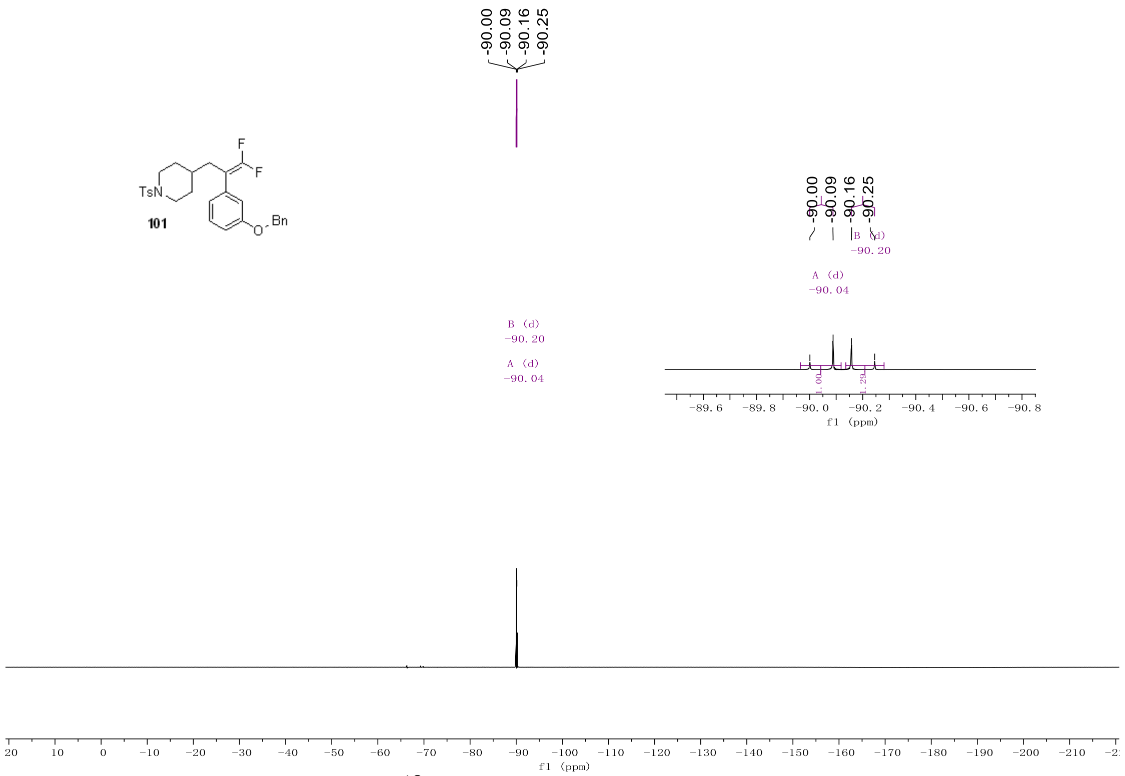

Supplementary Figure 243. ^{19}F NMR Spectra of compound **101**.

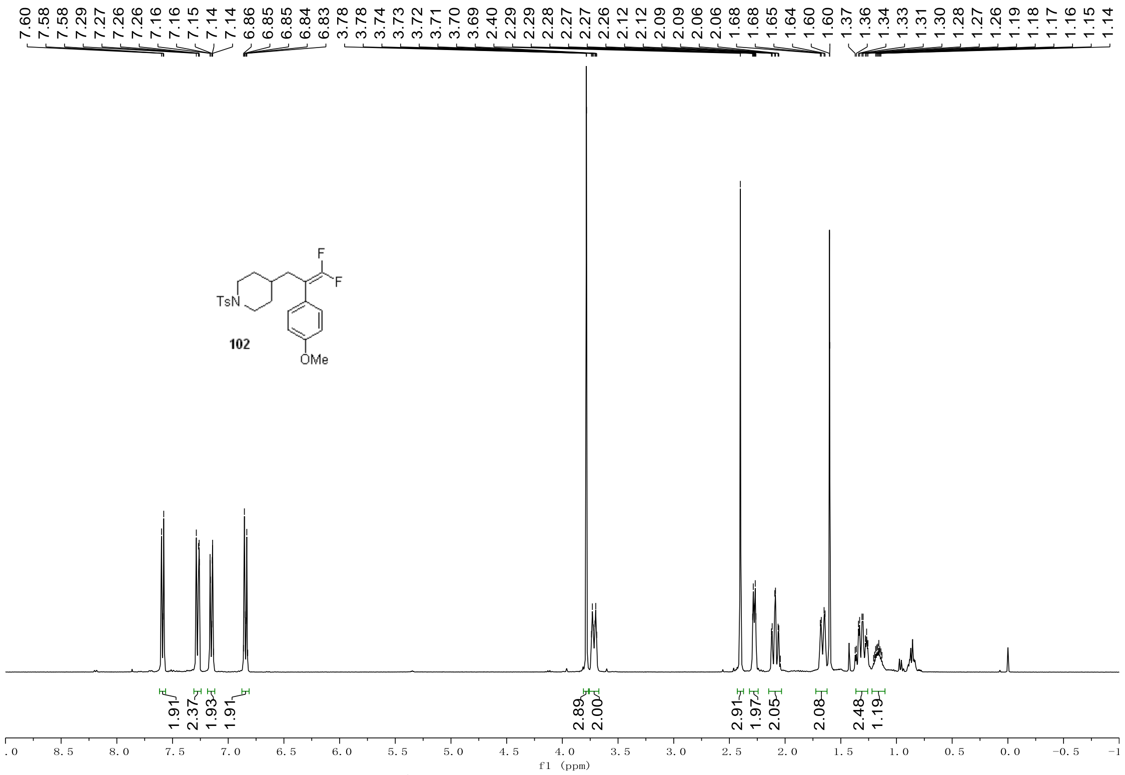

Supplementary Figure 244. ^{1}H NMR Spectra of compound **102**.

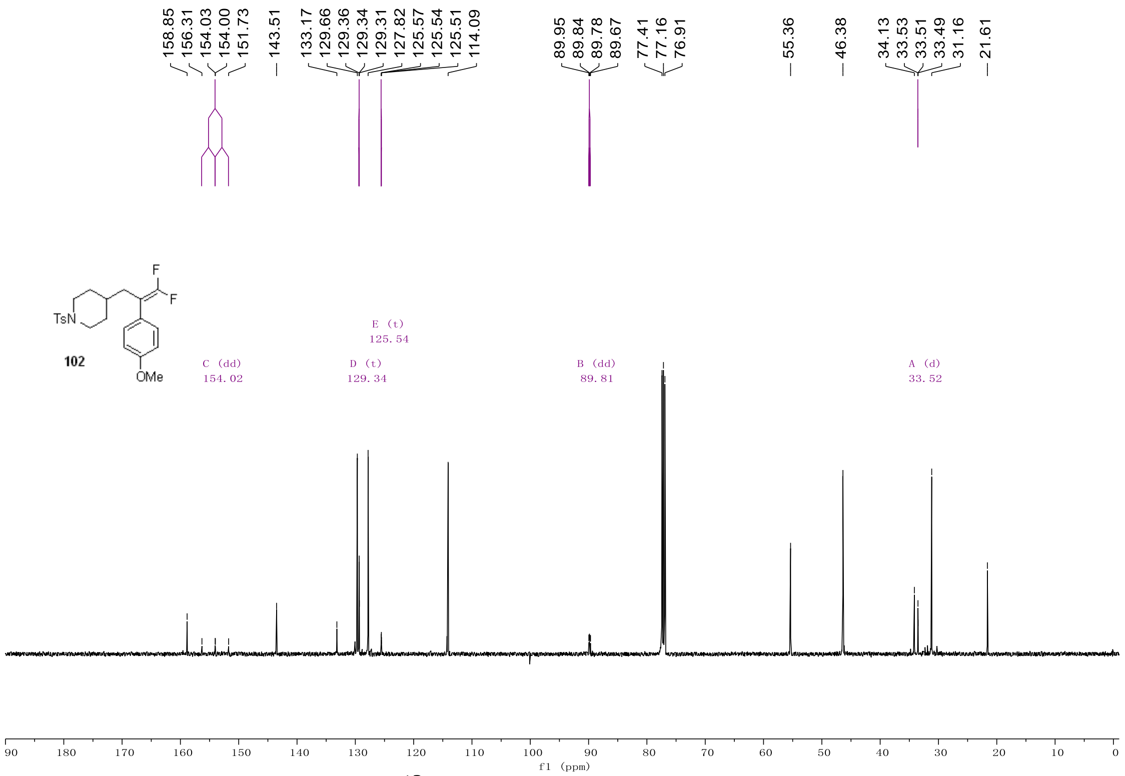

Supplementary Figure 245. ^{13}C NMR Spectra of compound **102**.

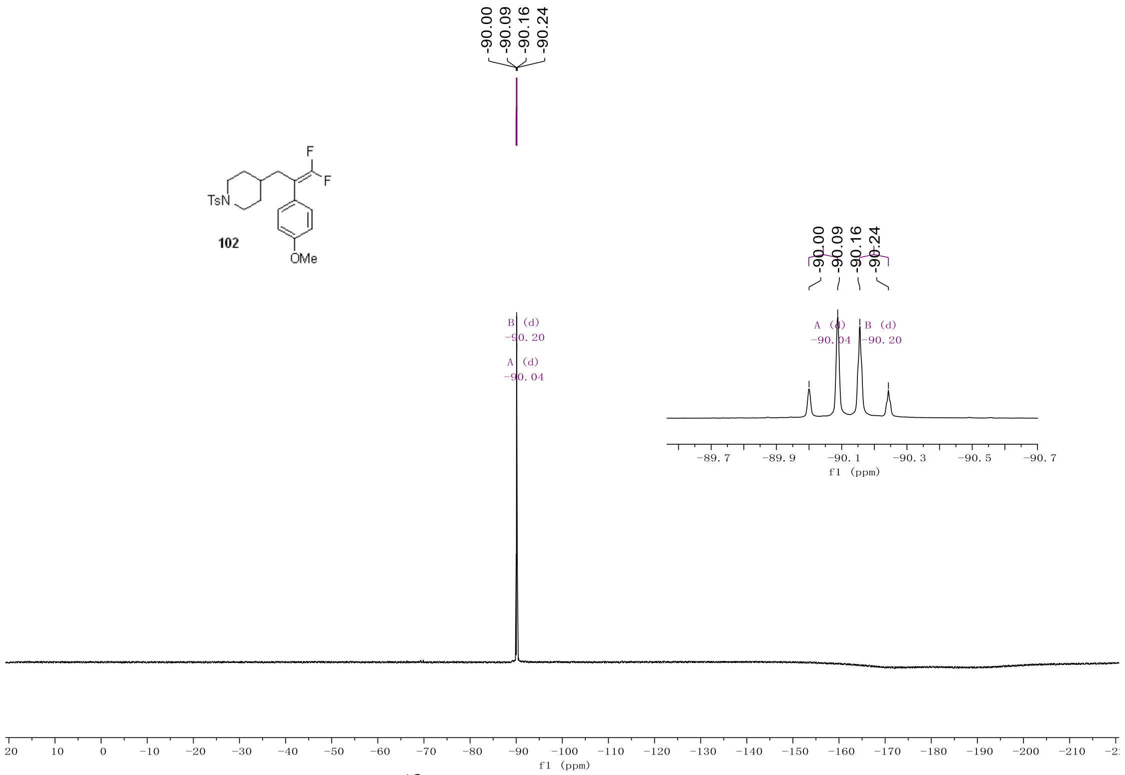

Supplementary Figure 246. ^{19}F NMR Spectra of compound **102**.

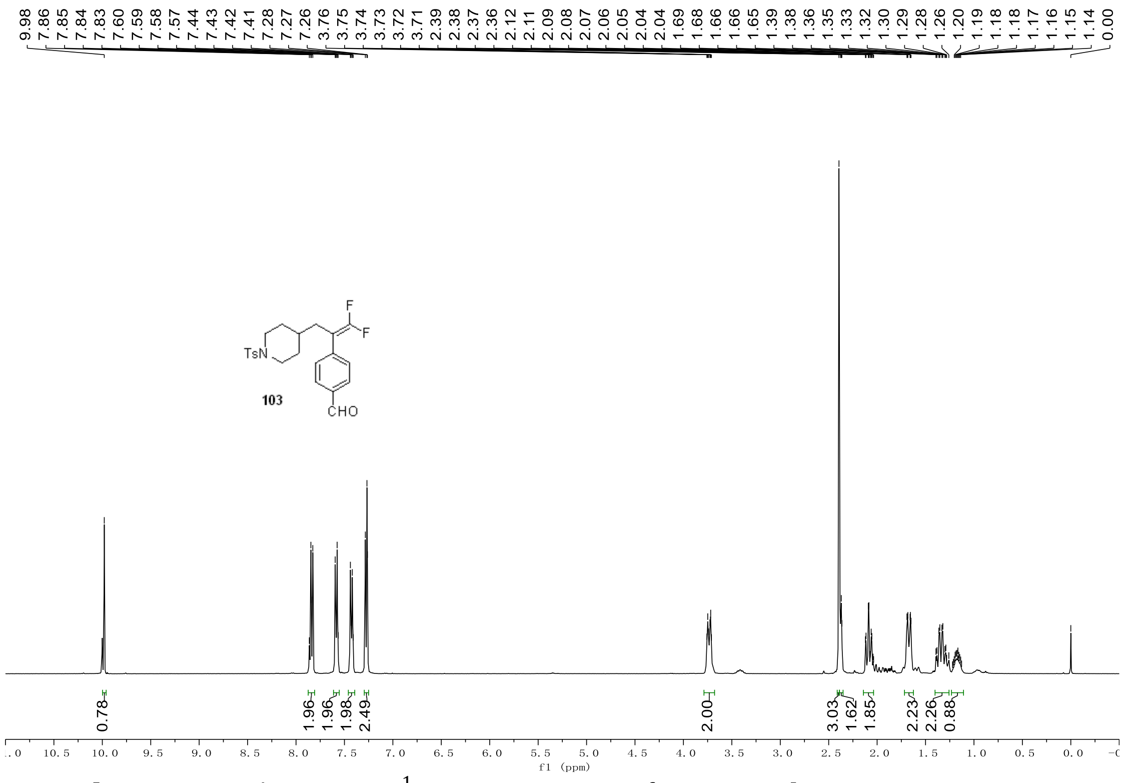

Supplementary Figure 247. ^{1}H NMR Spectra of compound **103**.

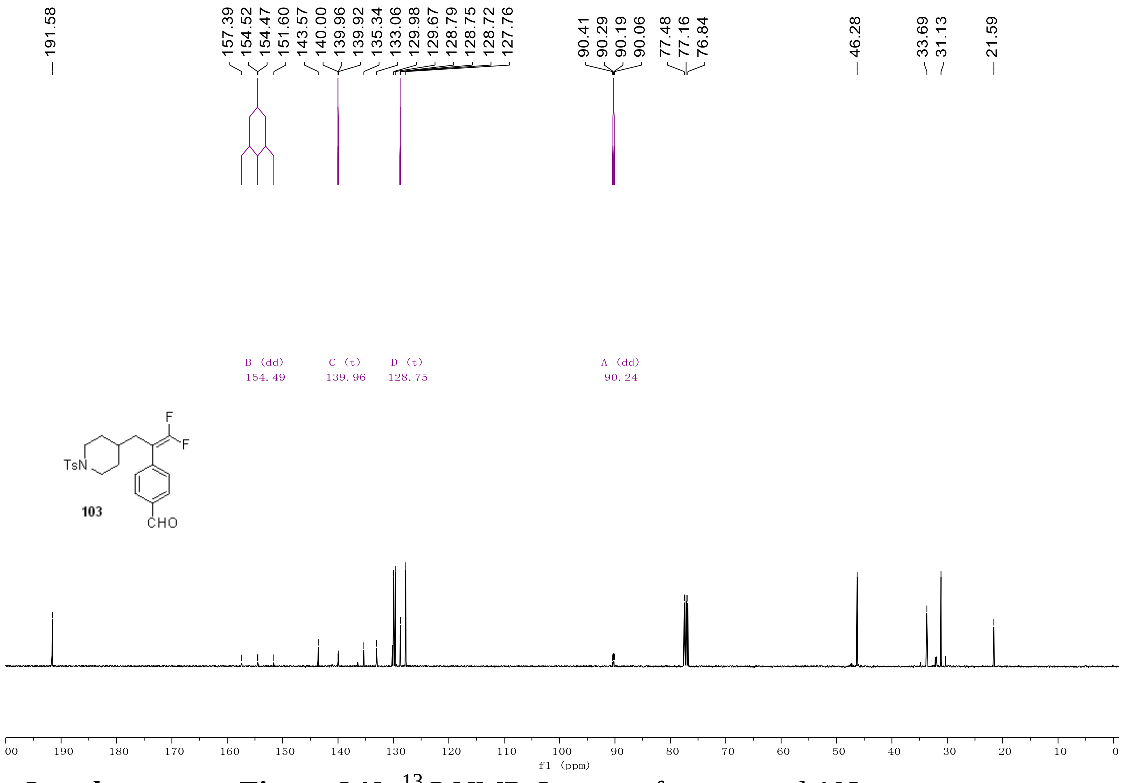

Supplementary Figure 248. ^{13}C NMR Spectra of compound **103**.

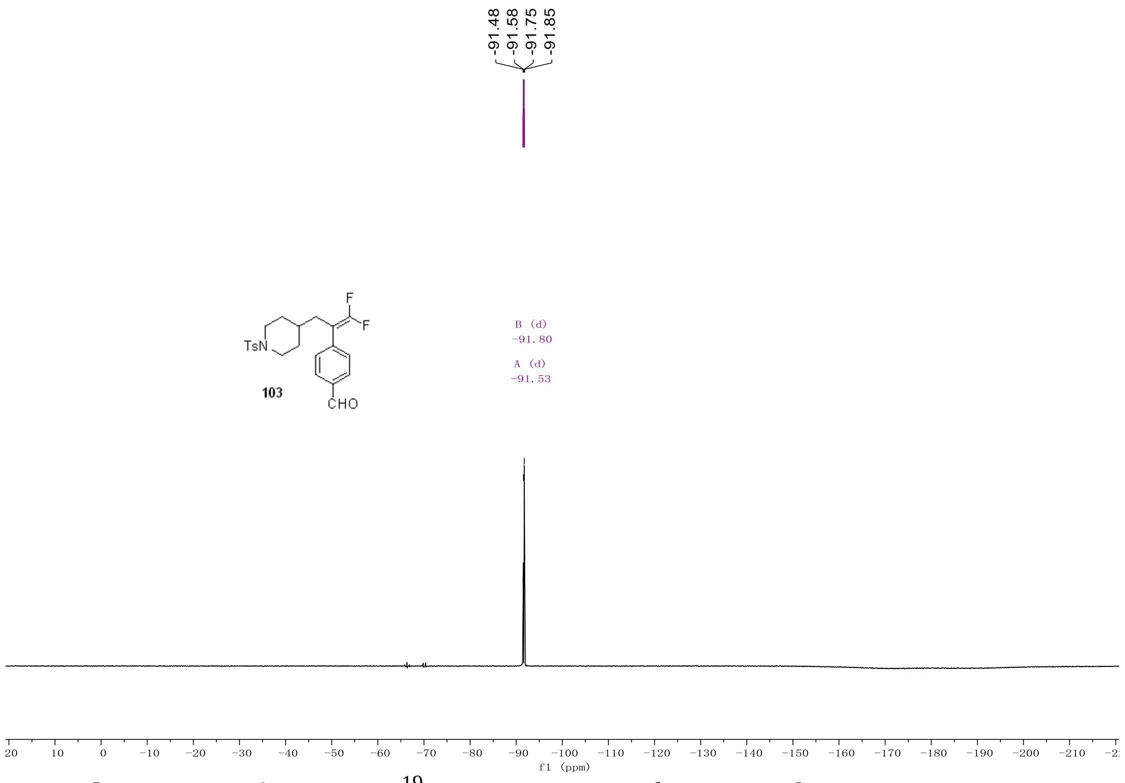

Supplementary Figure 249. ^{19}F NMR Spectra of compound **103**.

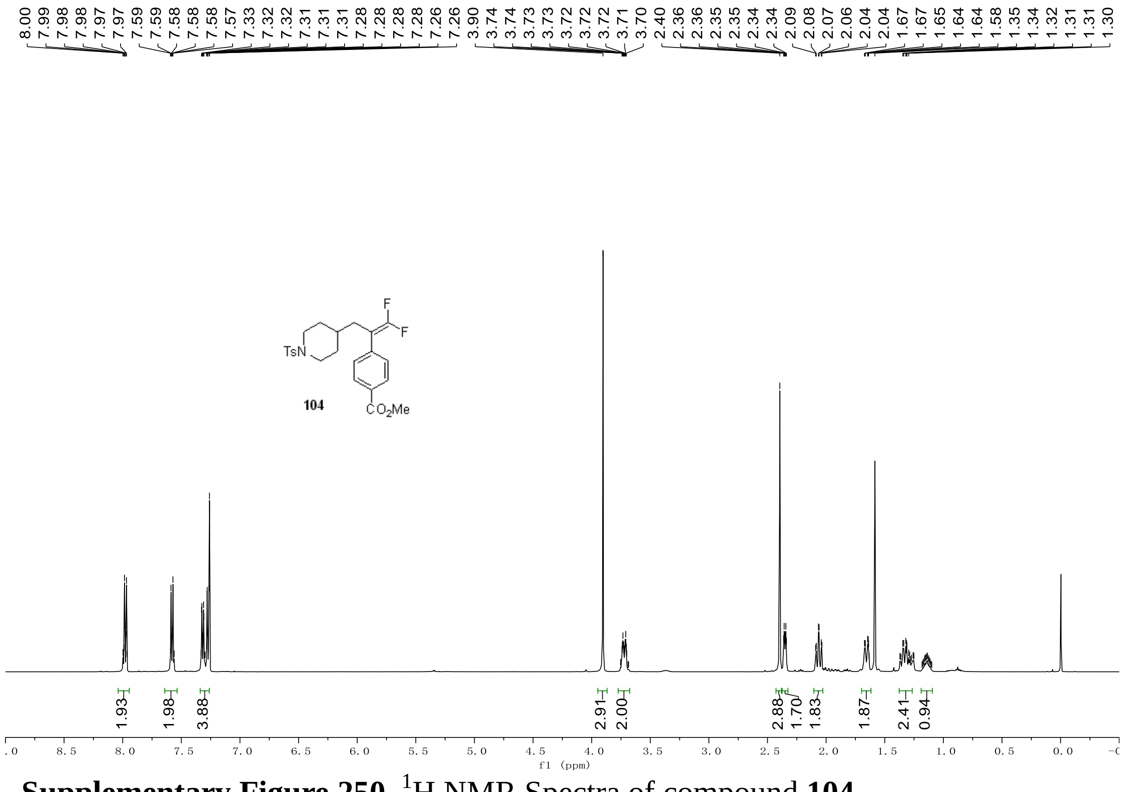

Supplementary Figure 250. ^{1}H NMR Spectra of compound **104**.

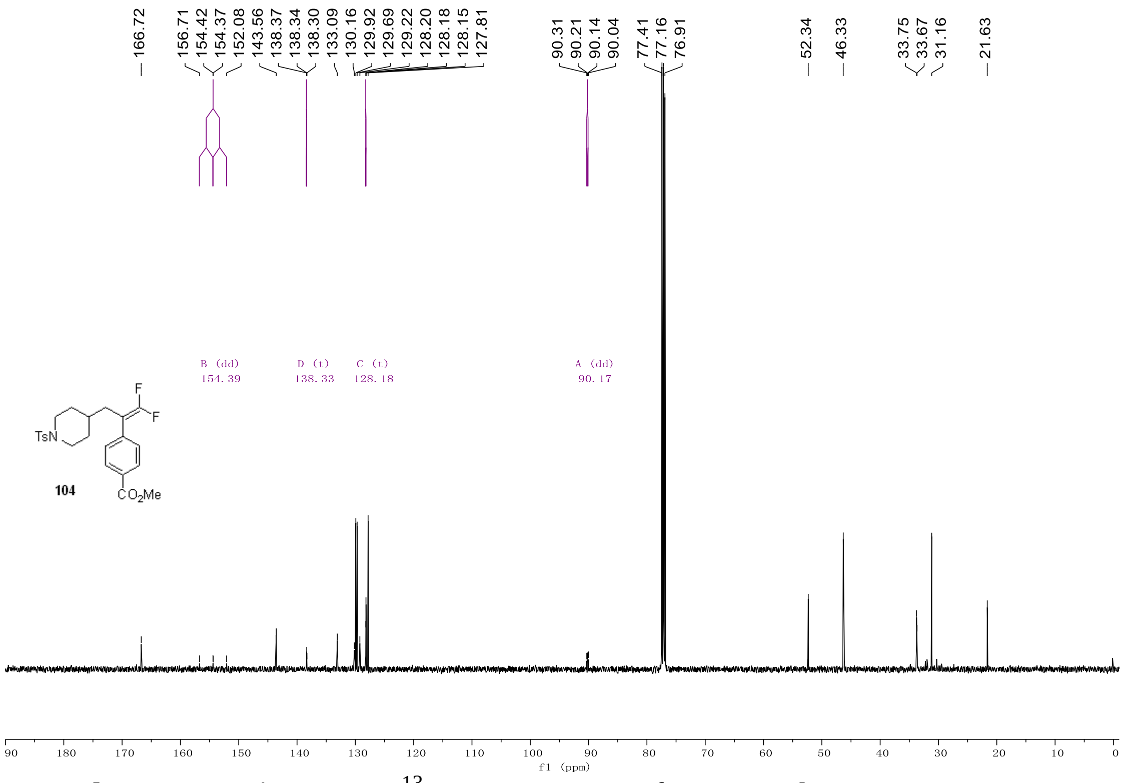

Supplementary Figure 251. ^{13}C NMR Spectra of compound **104**.

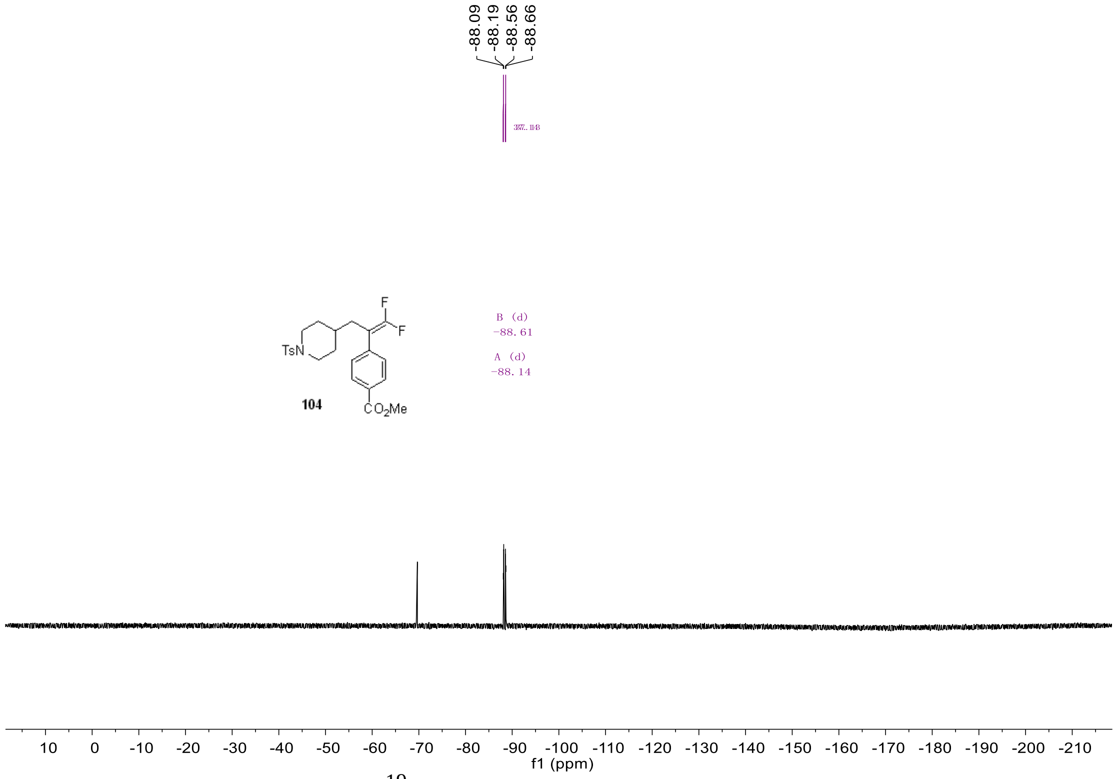

Supplementary Figure 252. ^{19}F NMR Spectra of compound **104**.

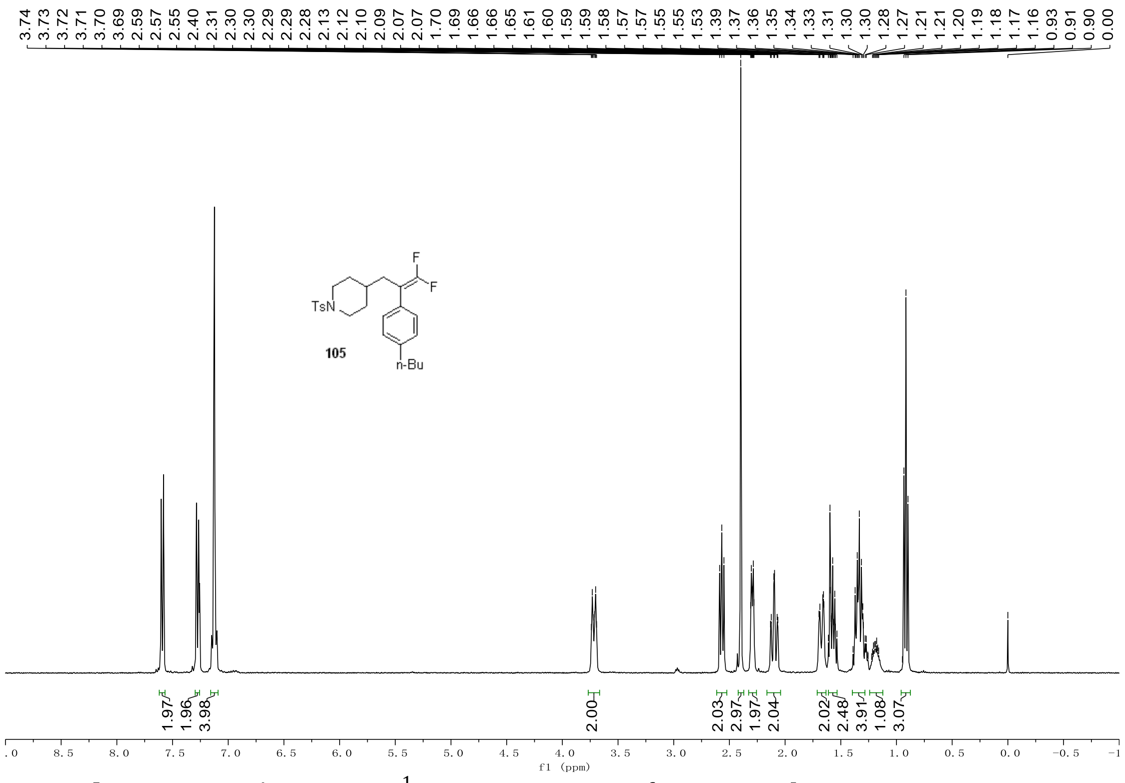

Supplementary Figure 253. ^{1}H NMR Spectra of compound **105**.

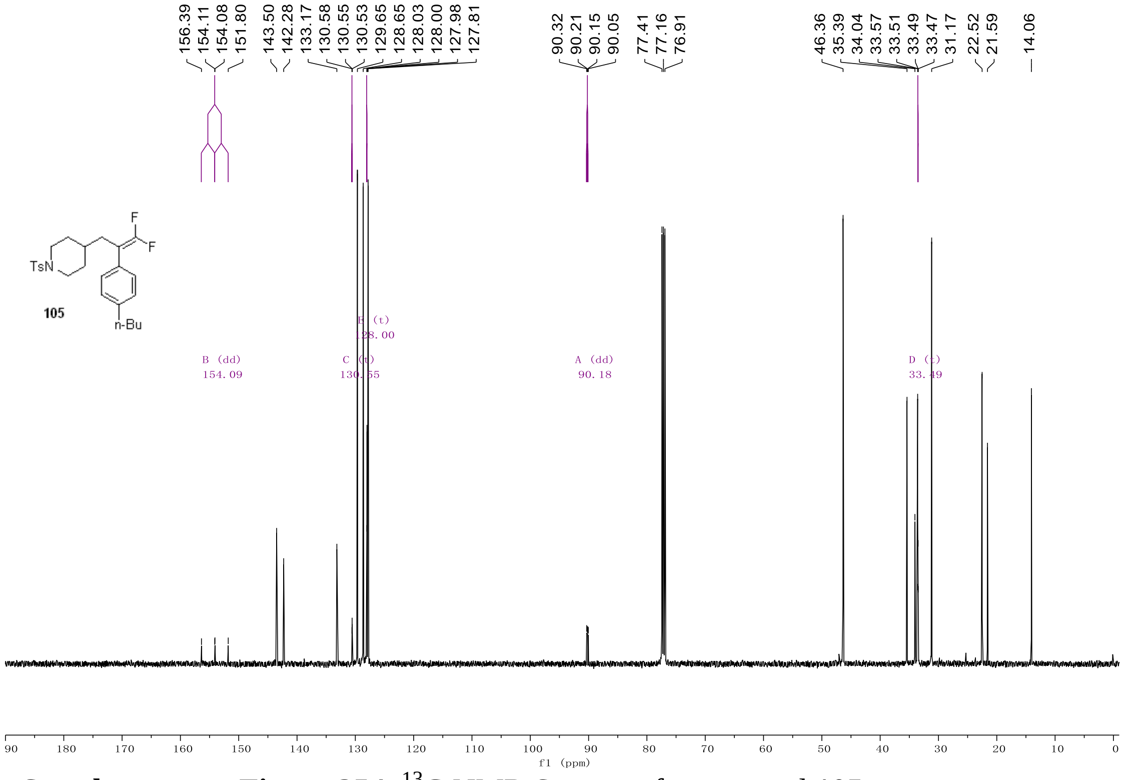

Supplementary Figure 254. ^{13}C NMR Spectra of compound **105**.

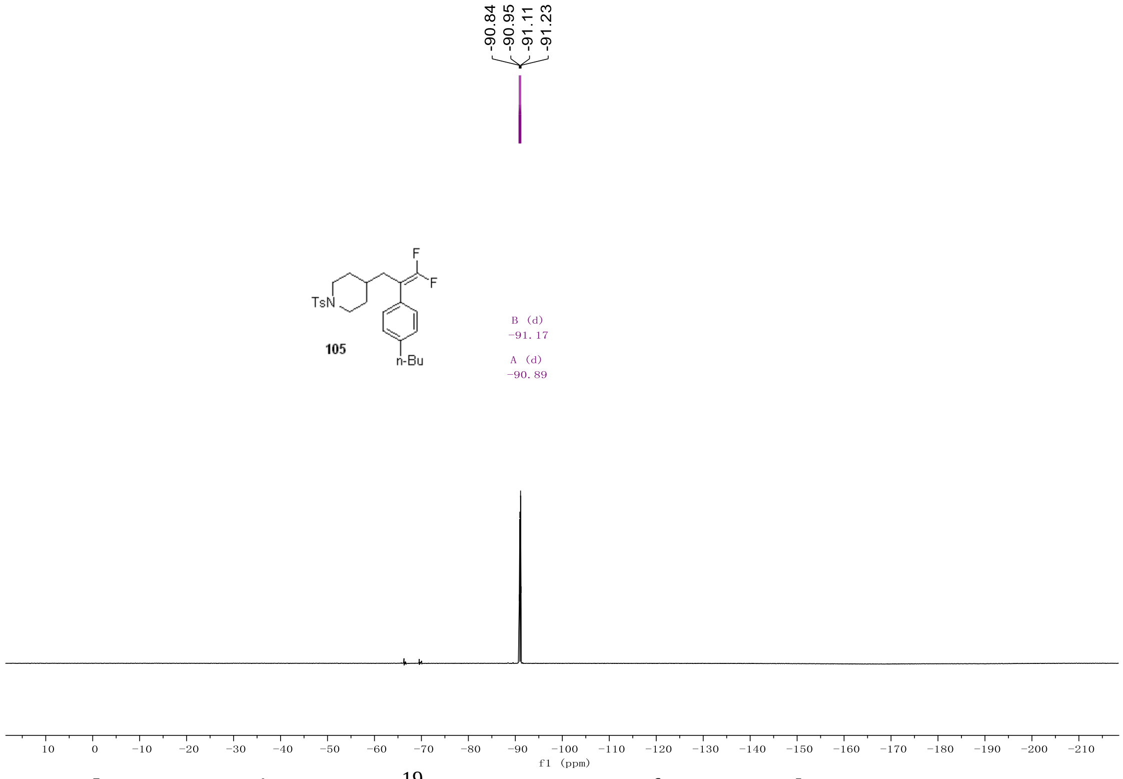

Supplementary Figure 255. ^{19}F NMR Spectra of compound **105**.

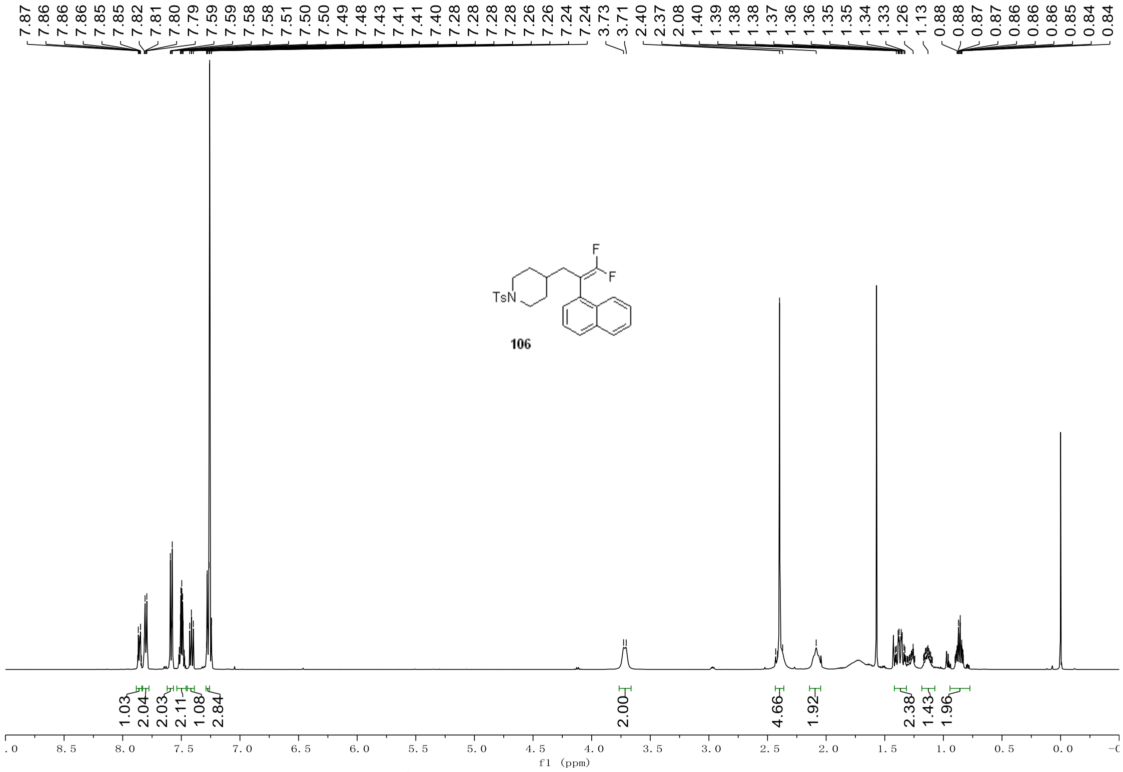

Supplementary Figure 256. ^{1}H NMR Spectra of compound **106**.

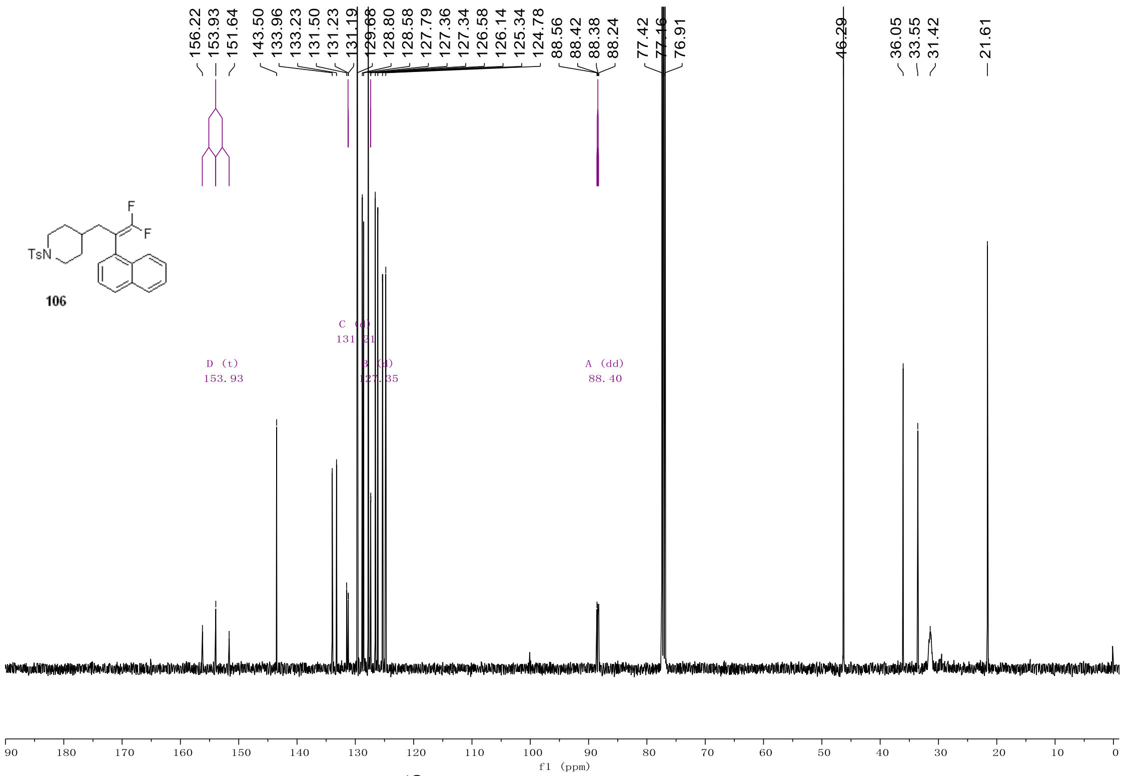

Supplementary Figure 257. ^{13}C NMR Spectra of compound **106**.

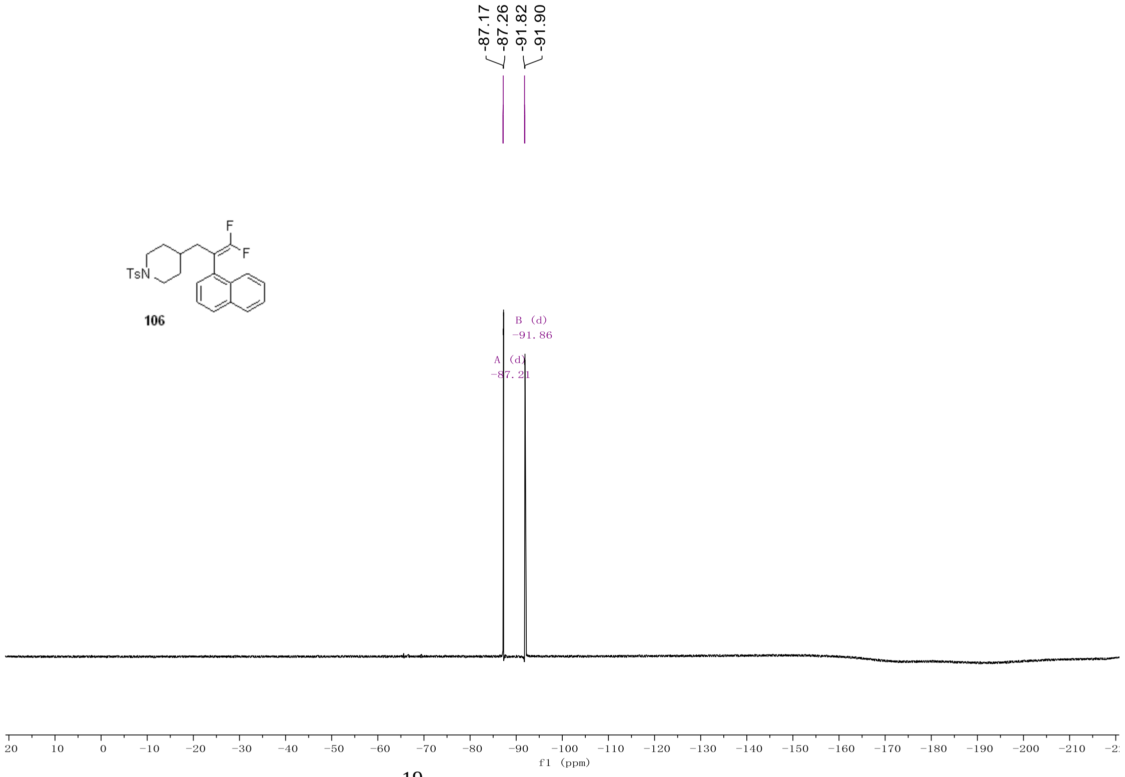

Supplementary Figure 258. ^{19}F NMR Spectra of compound **106**.

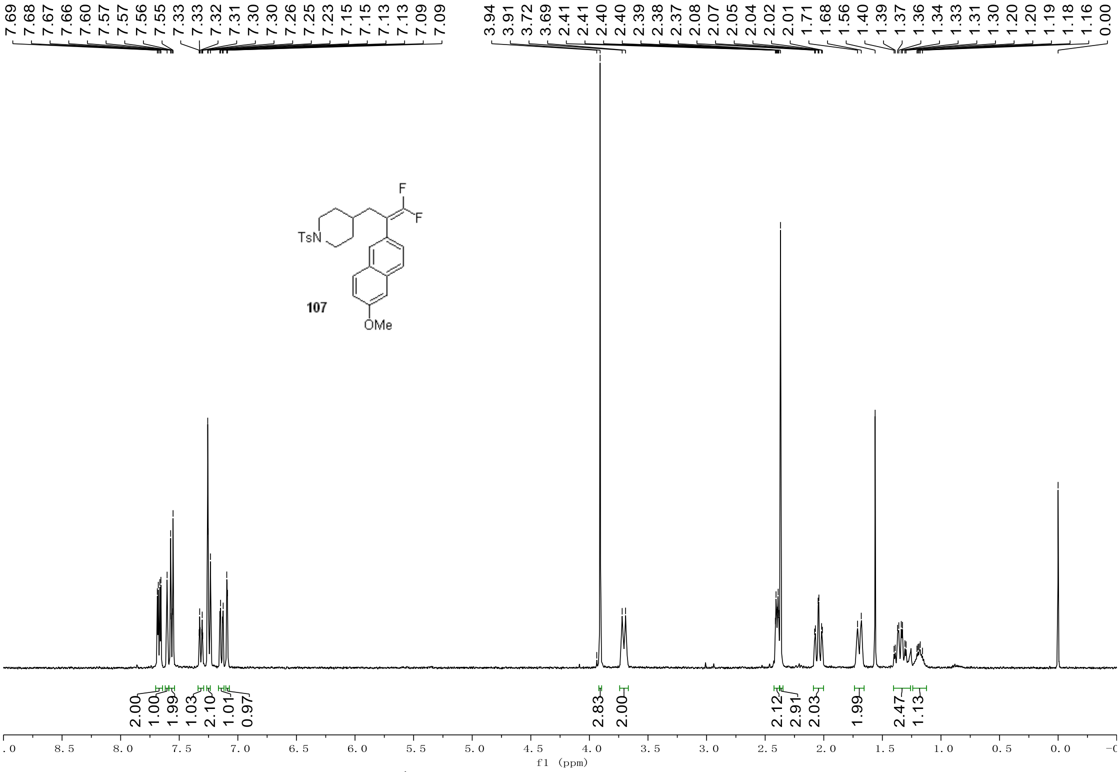

Supplementary Figure 259. ^{1}H NMR Spectra of compound **107**.

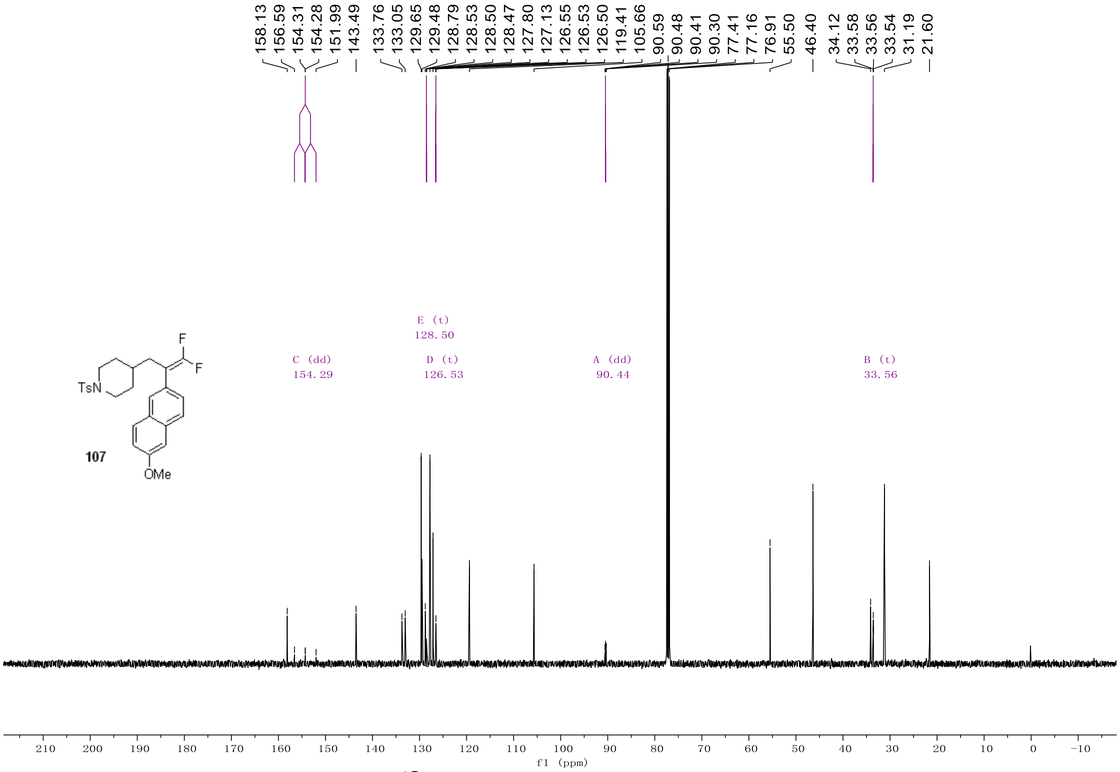

Supplementary Figure 260. ^{13}C NMR Spectra of compound **107**.

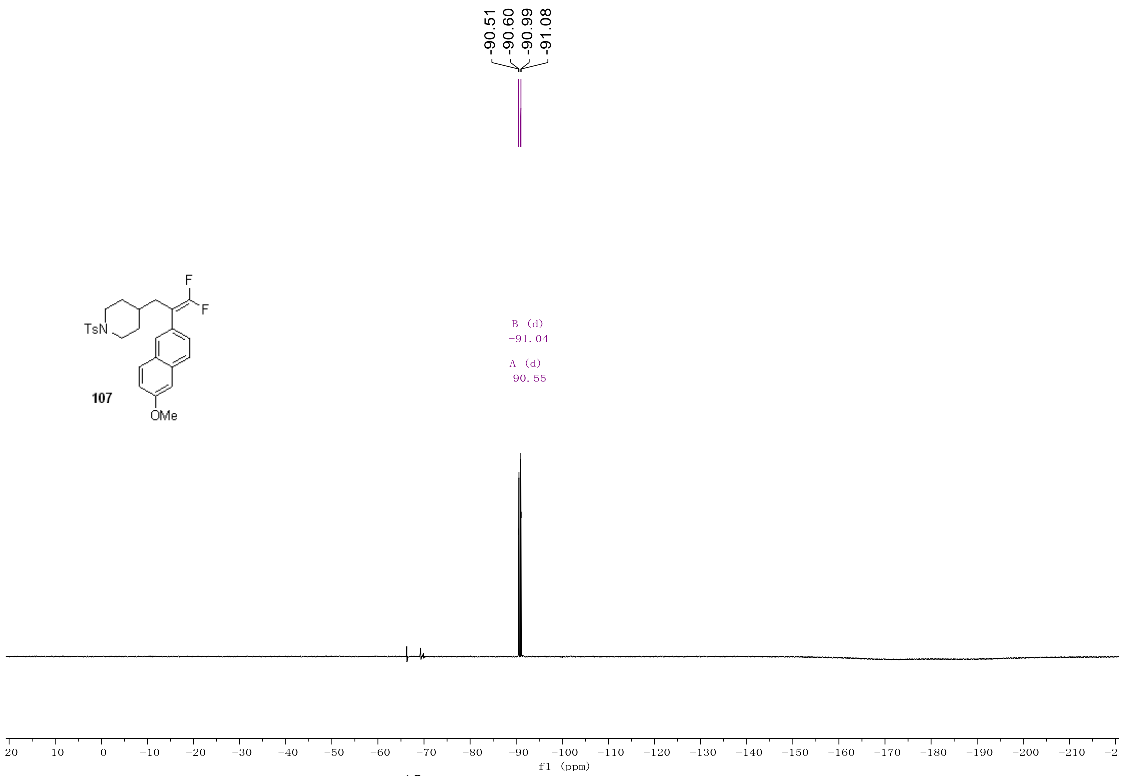

Supplementary Figure 261. ^{19}F NMR Spectra of compound **107**.

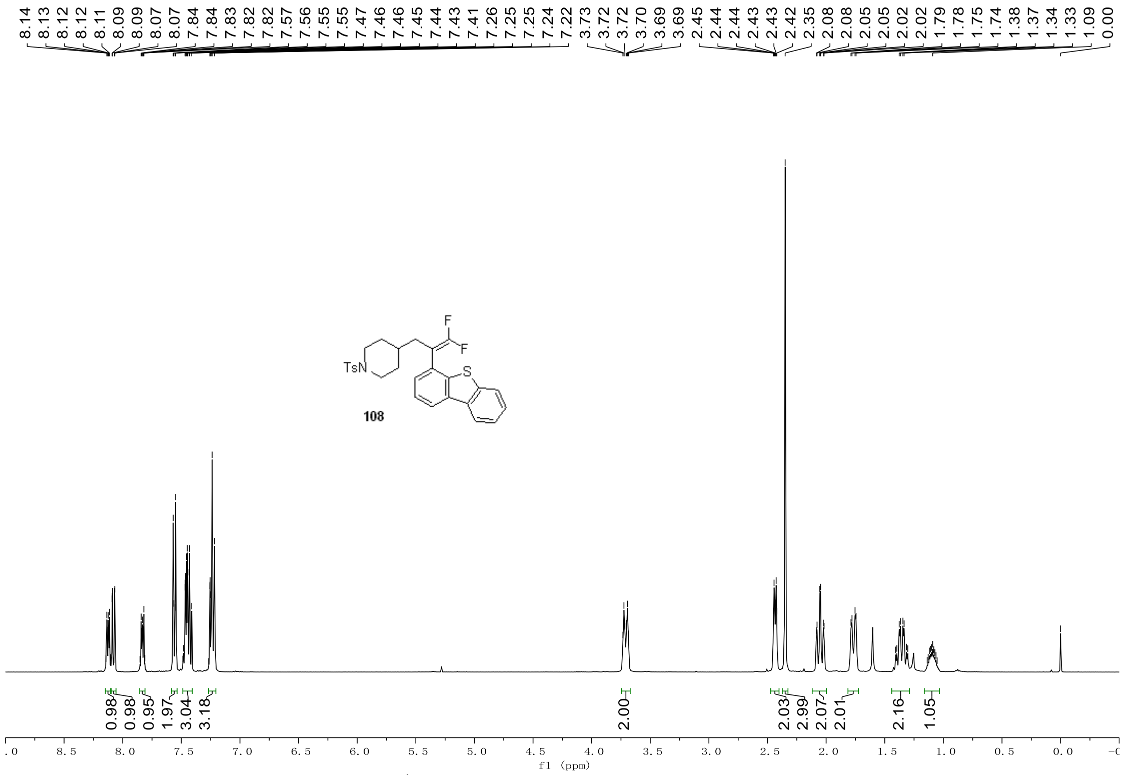

Supplementary Figure 262. ^{1}H NMR Spectra of compound **108**.

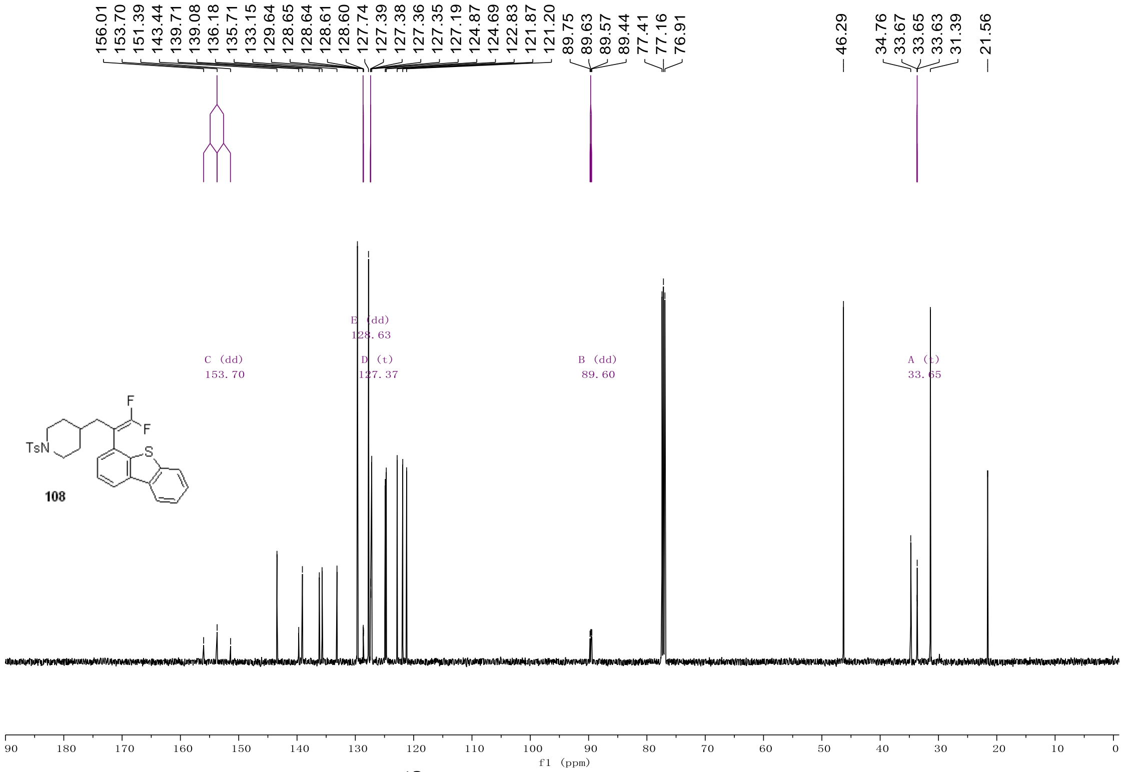

Supplementary Figure 263. ^{13}C NMR Spectra of compound **108**.

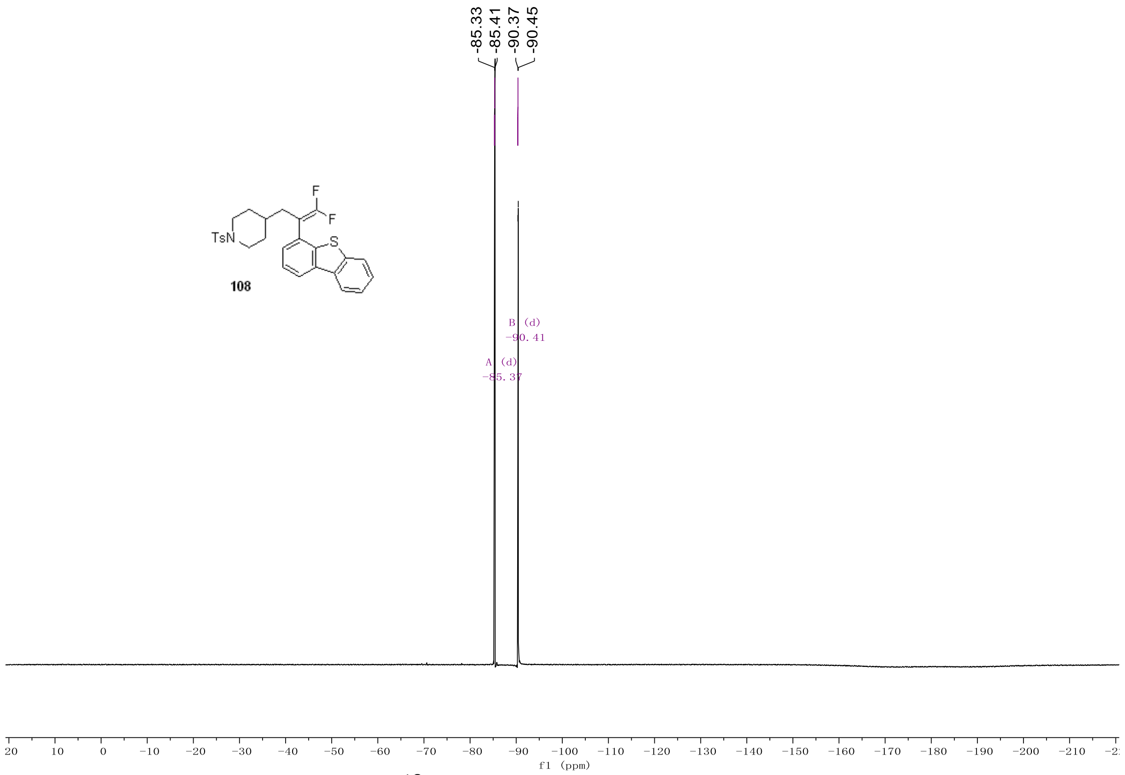

Supplementary Figure 264. ^{19}F NMR Spectra of compound **108**.

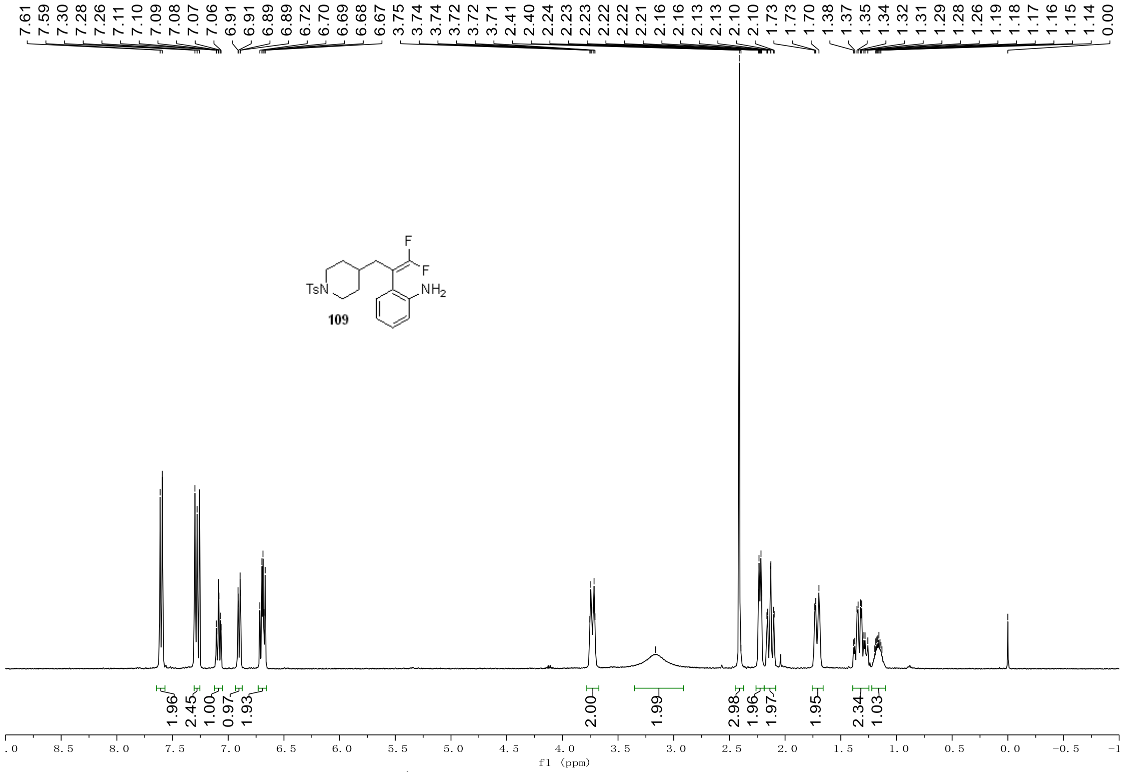

Supplementary Figure 265. ^{1}H NMR Spectra of compound **109**.

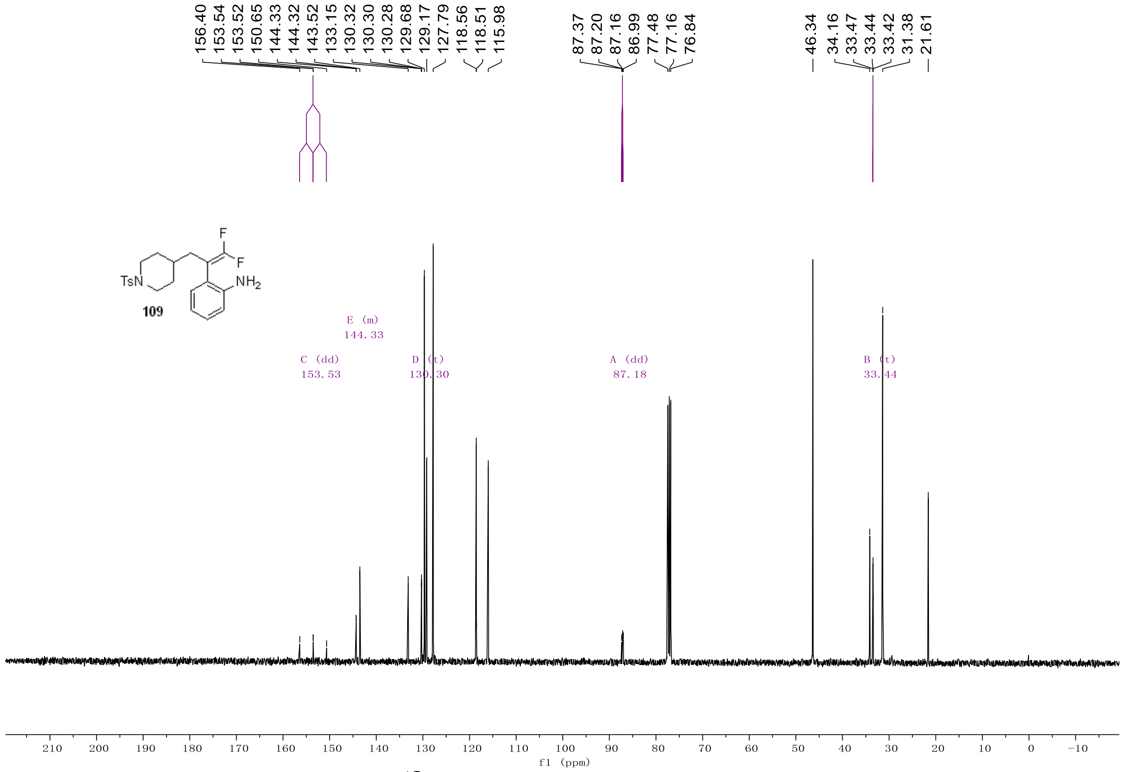

Supplementary Figure 266. ^{13}C NMR Spectra of compound **109**.

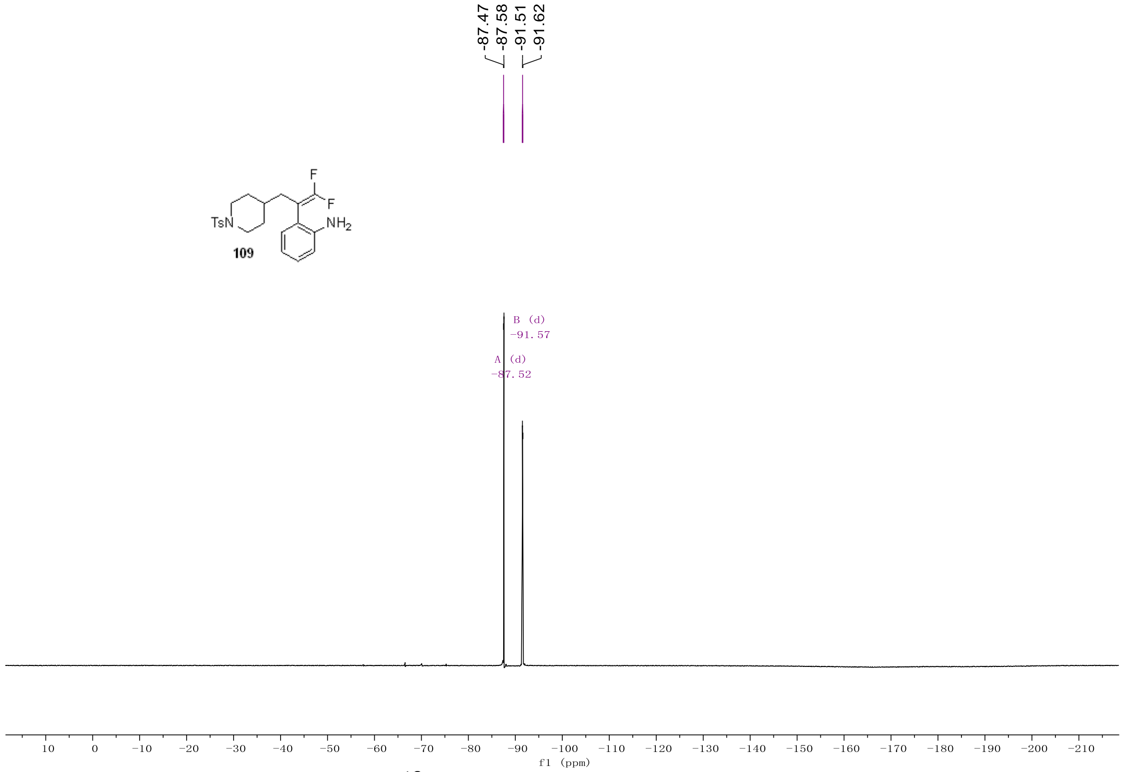

Supplementary Figure 267. ^{19}F NMR Spectra of compound **109**.

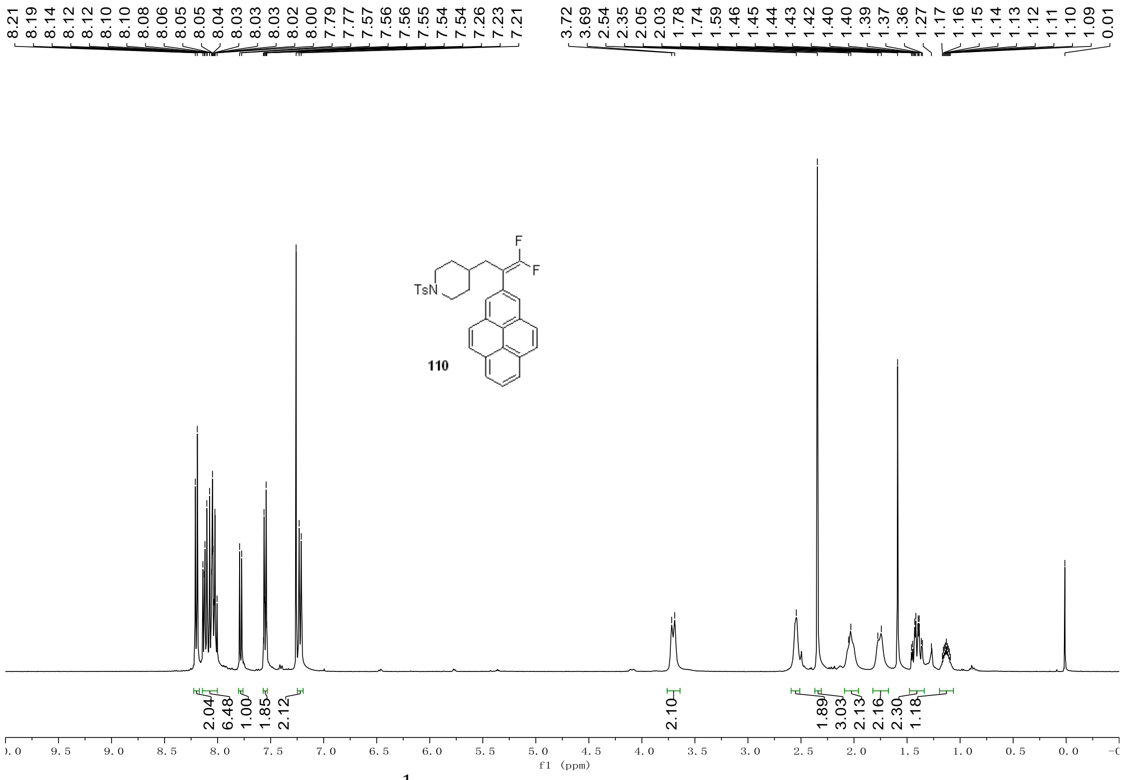

Supplementary Figure 268. ^{1}H NMR Spectra of compound **110**.

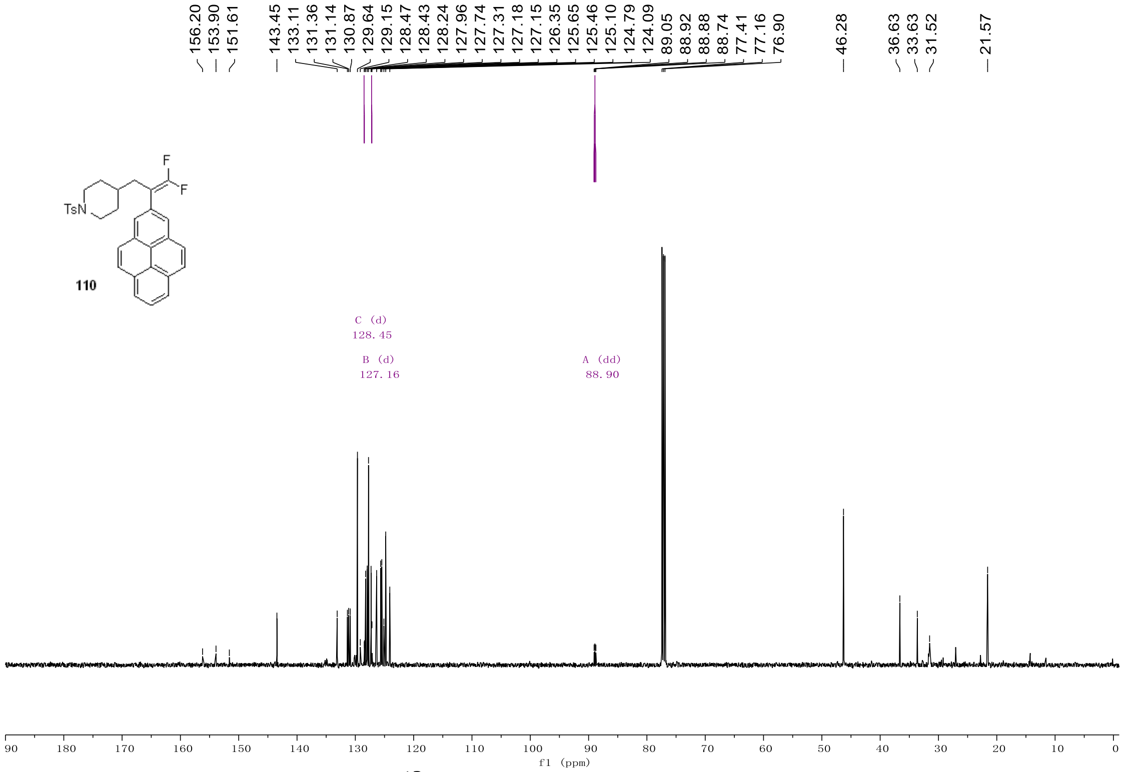

Supplementary Figure 269. ^{13}C NMR Spectra of compound **110**.

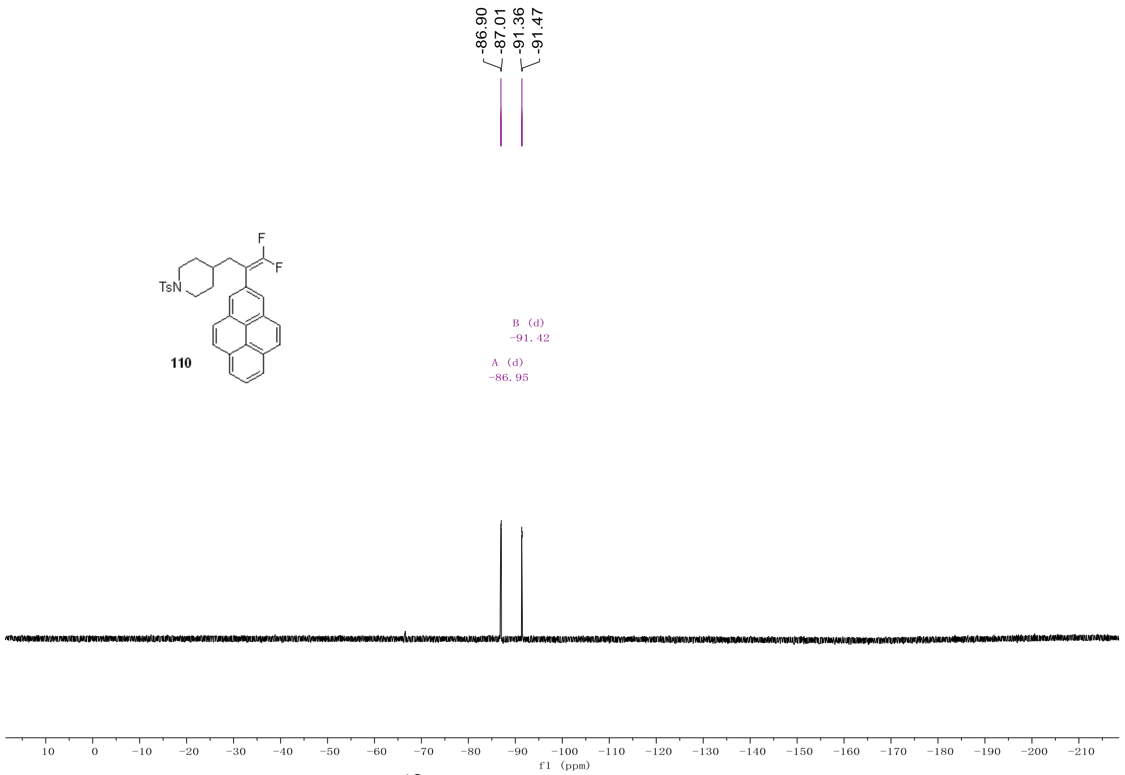

Supplementary Figure 270. ^{19}F NMR Spectra of compound **110**.

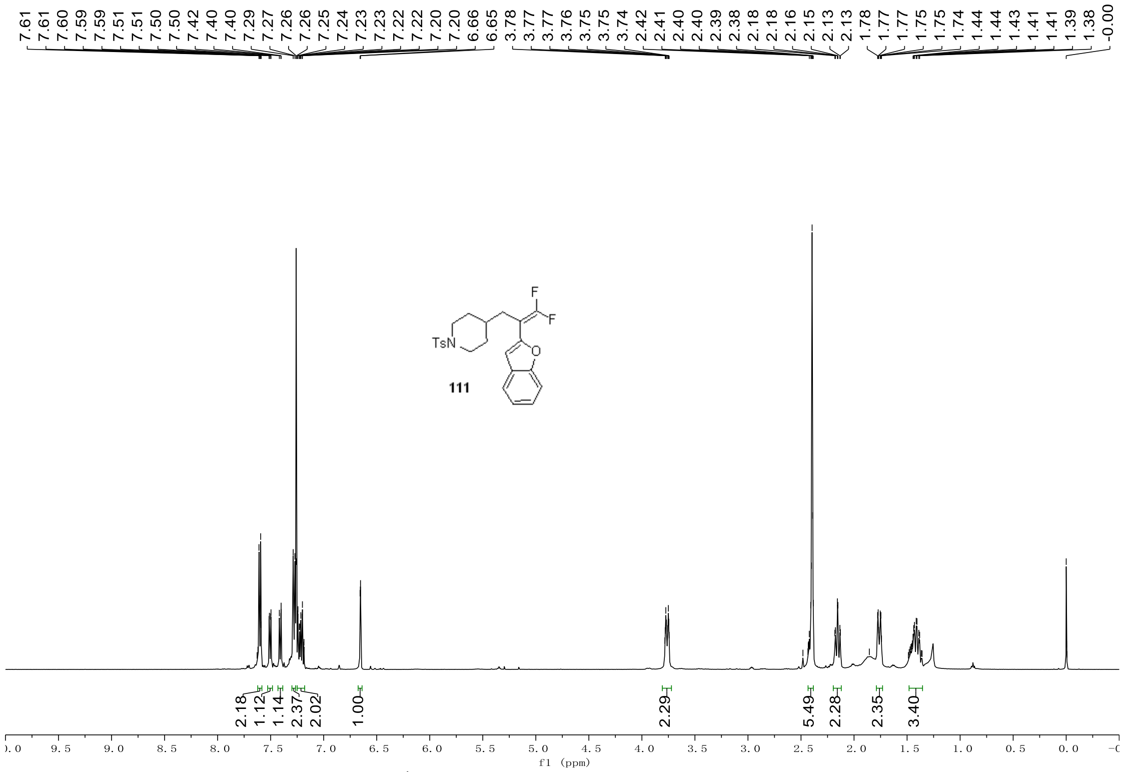

Supplementary Figure 271. ^{1}H NMR Spectra of compound **111**.

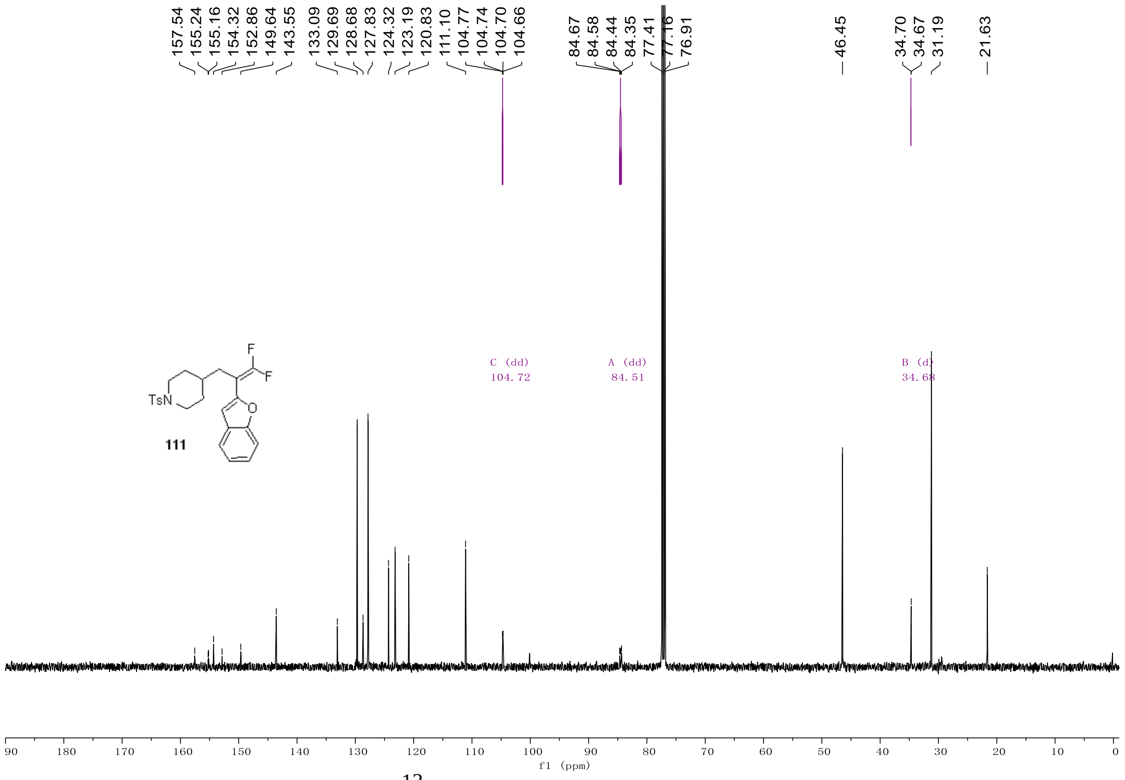

Supplementary Figure 272. ^{13}C NMR Spectra of compound **111**.

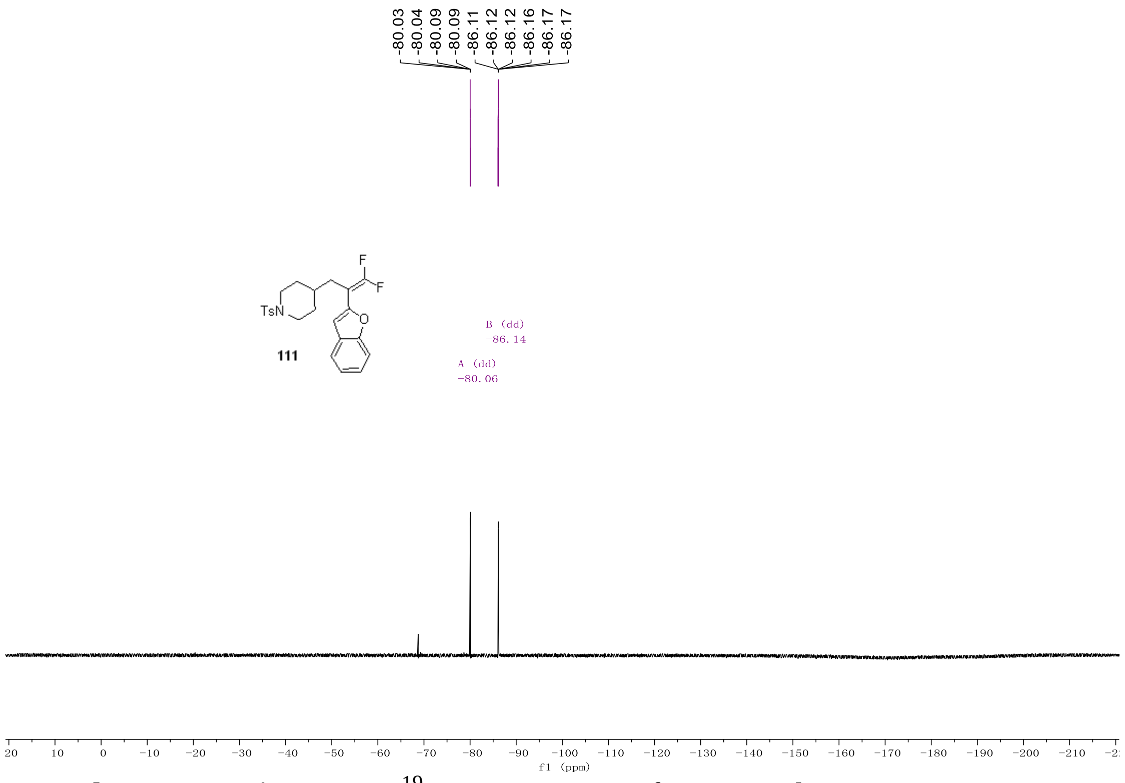

Supplementary Figure 273. ^{19}F NMR Spectra of compound **111**.

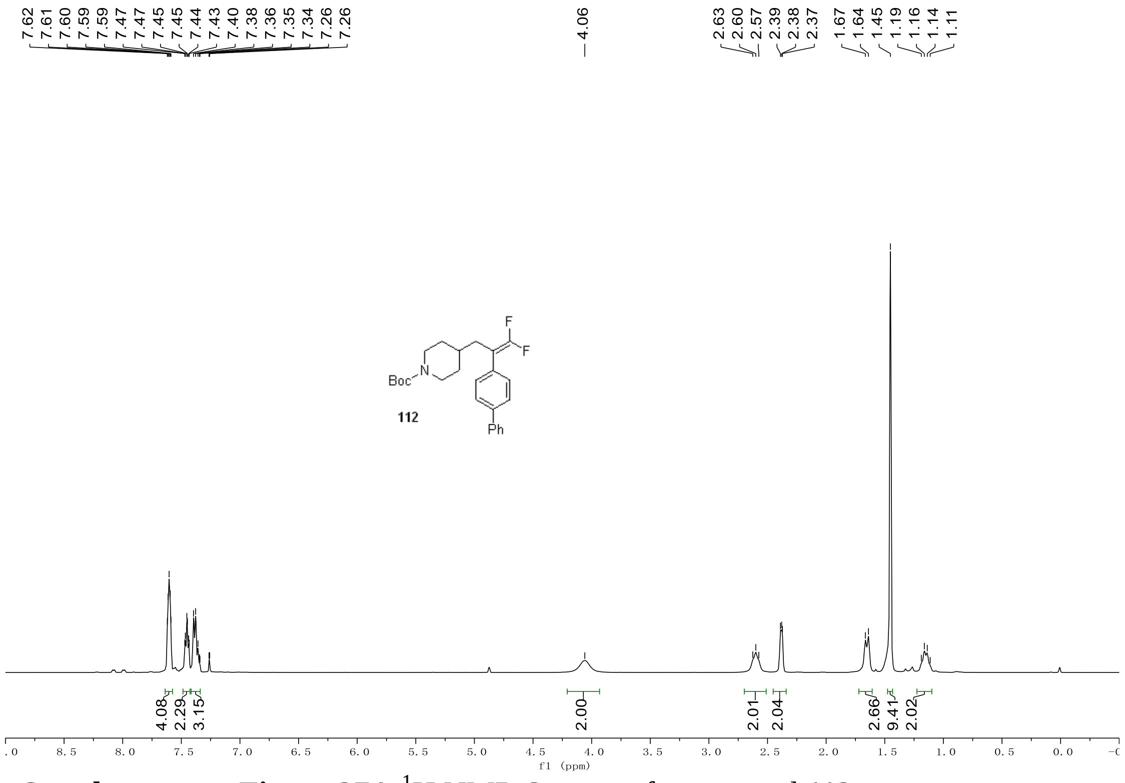

Supplementary Figure 274. ^{1}H NMR Spectra of compound **112**.

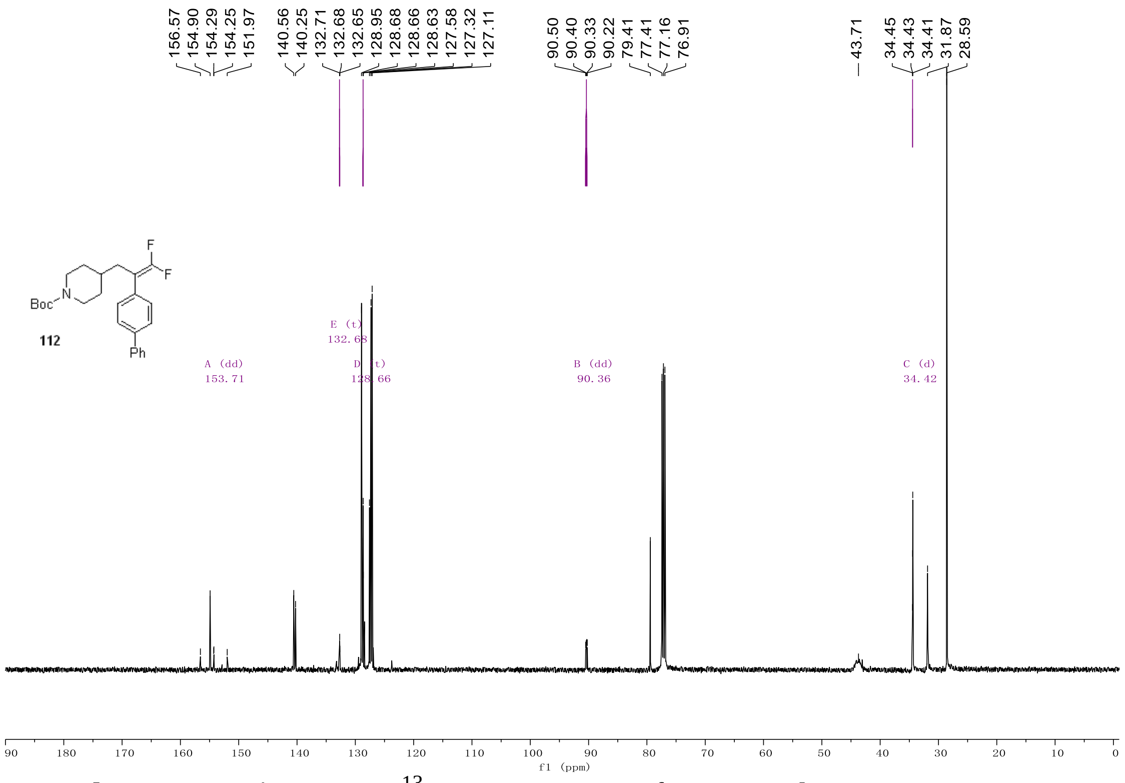

Supplementary Figure 275. ^{13}C NMR Spectra of compound **112**.

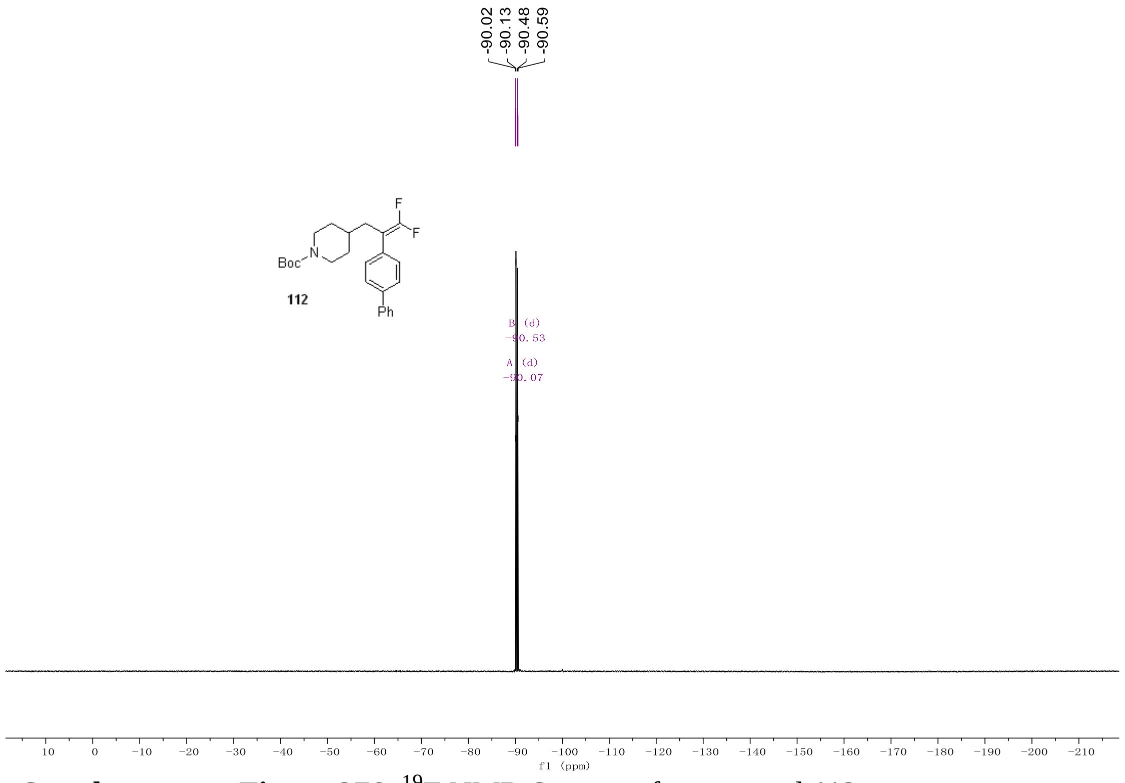

Supplementary Figure 276. ^{19}F NMR Spectra of compound **112**.

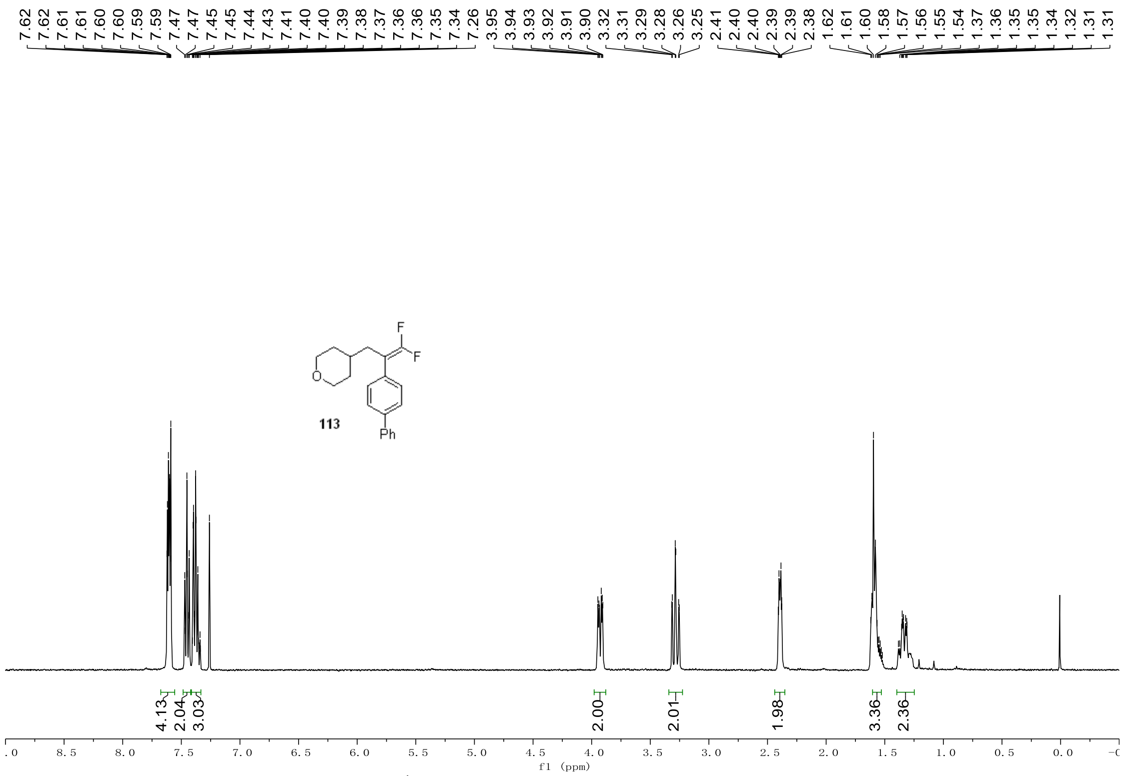

Supplementary Figure 277. ^{1}H NMR Spectra of compound **113**.

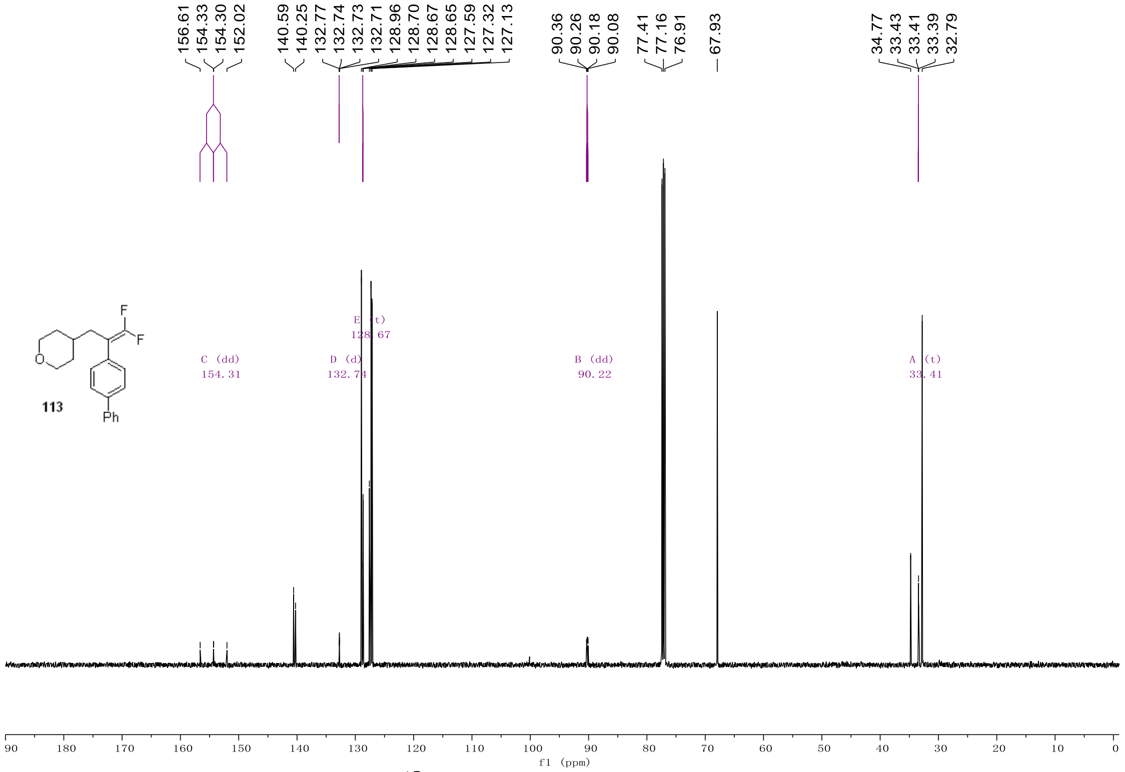

Supplementary Figure 278. ^{13}C NMR Spectra of compound **113**.

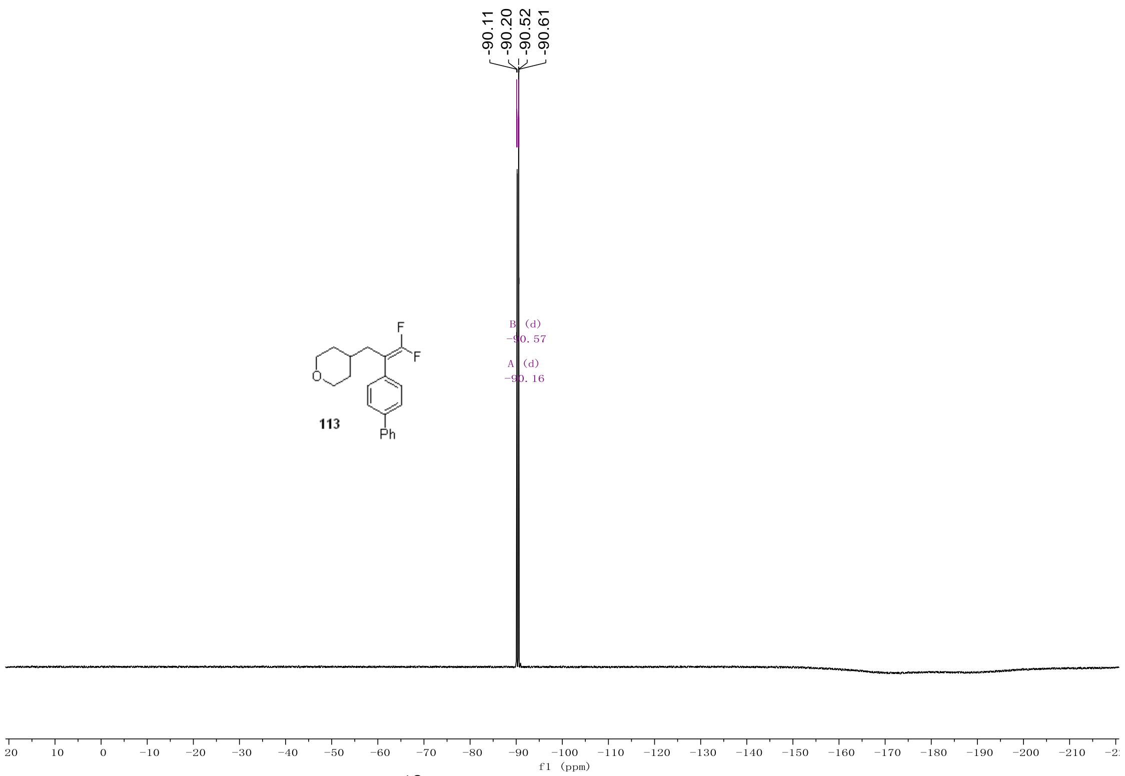

Supplementary Figure 279. ^{19}F NMR Spectra of compound **113**.

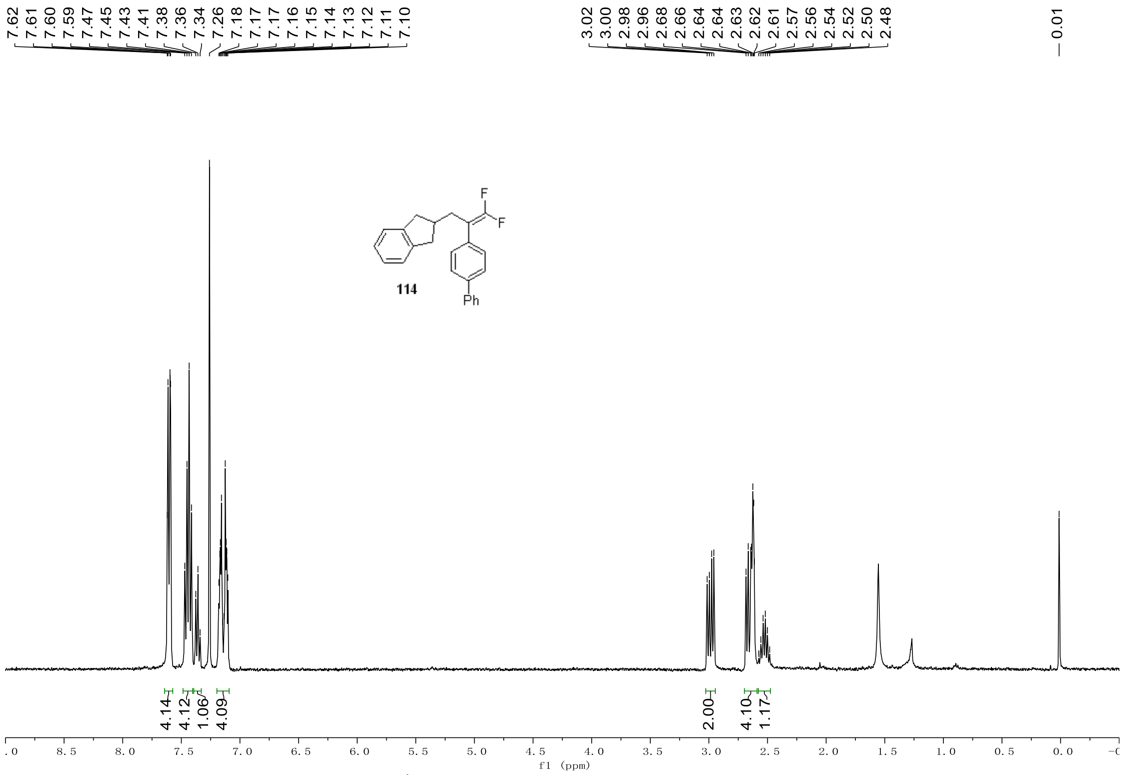

Supplementary Figure 280. ^{1}H NMR Spectra of compound **114**.

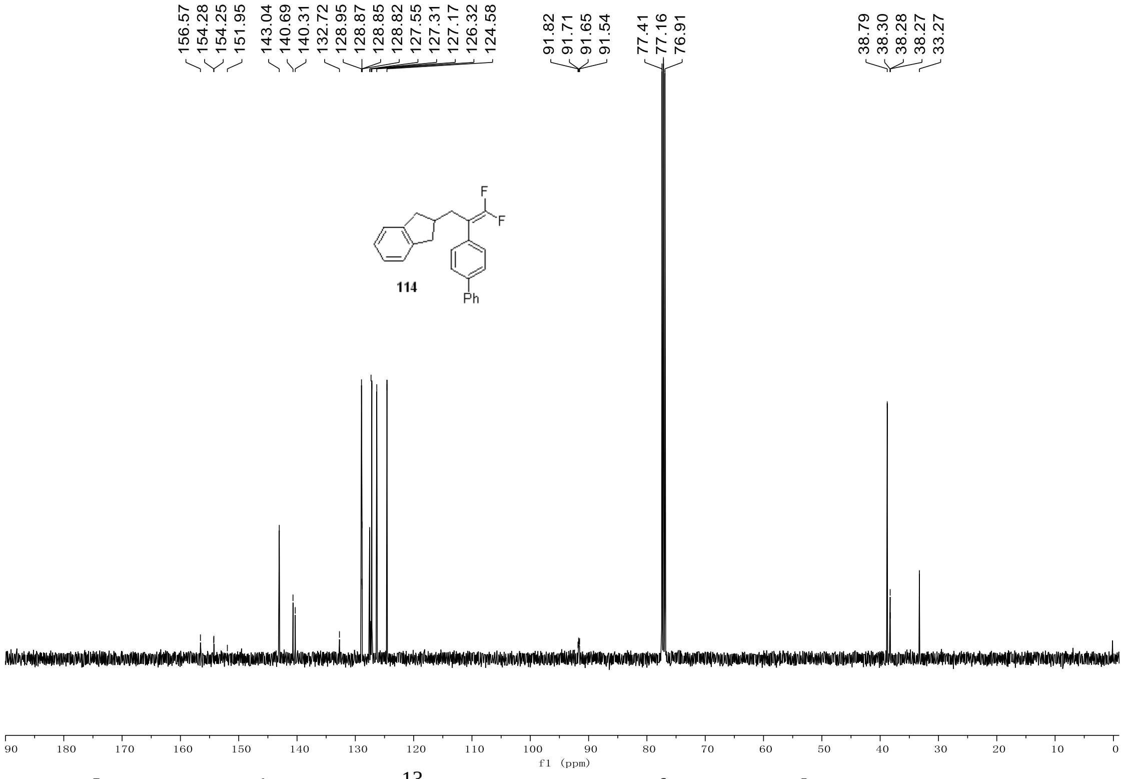

Supplementary Figure 281. ^{13}H NMR Spectra of compound **114**.

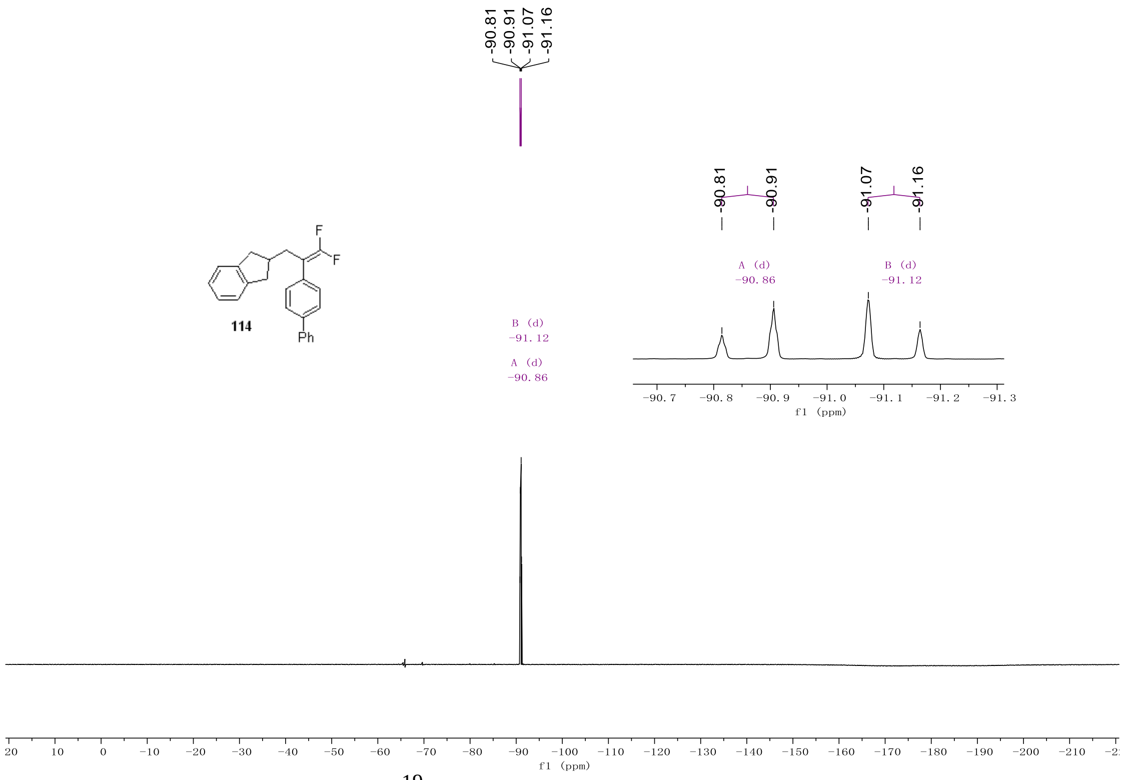

Supplementary Figure 282. ^{19}F NMR Spectra of compound **114**.

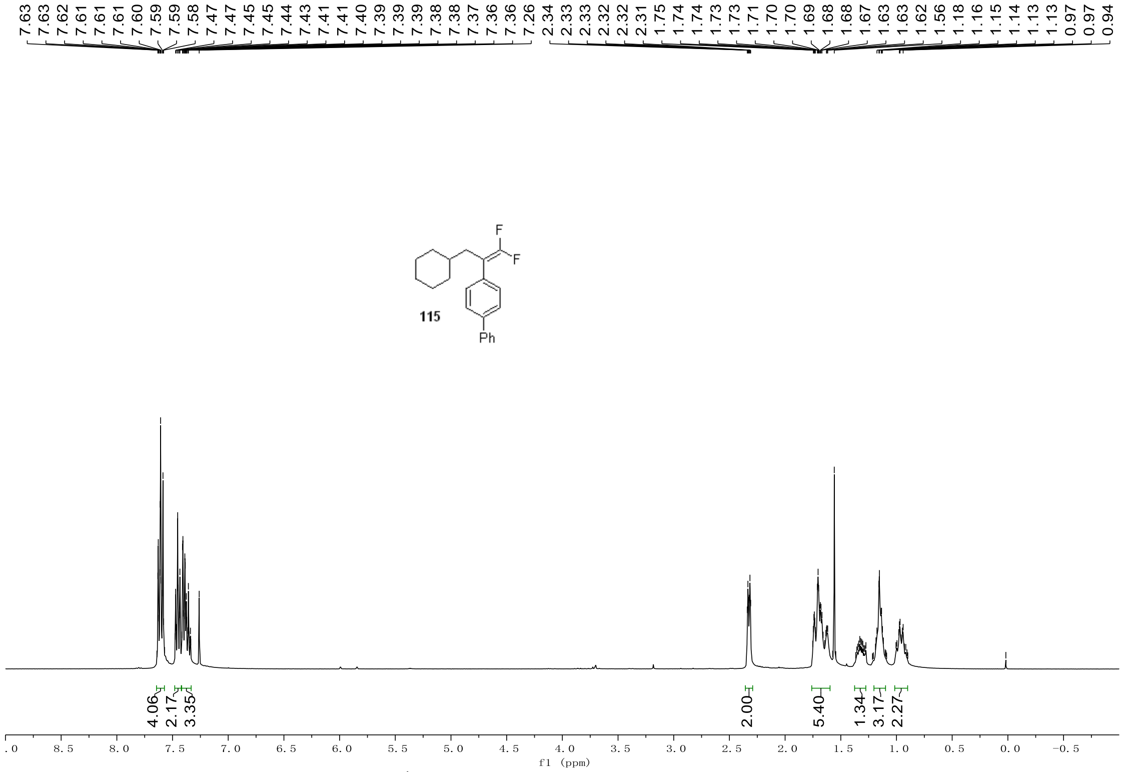

Supplementary Figure 283. ^{1}H NMR Spectra of compound **115**.

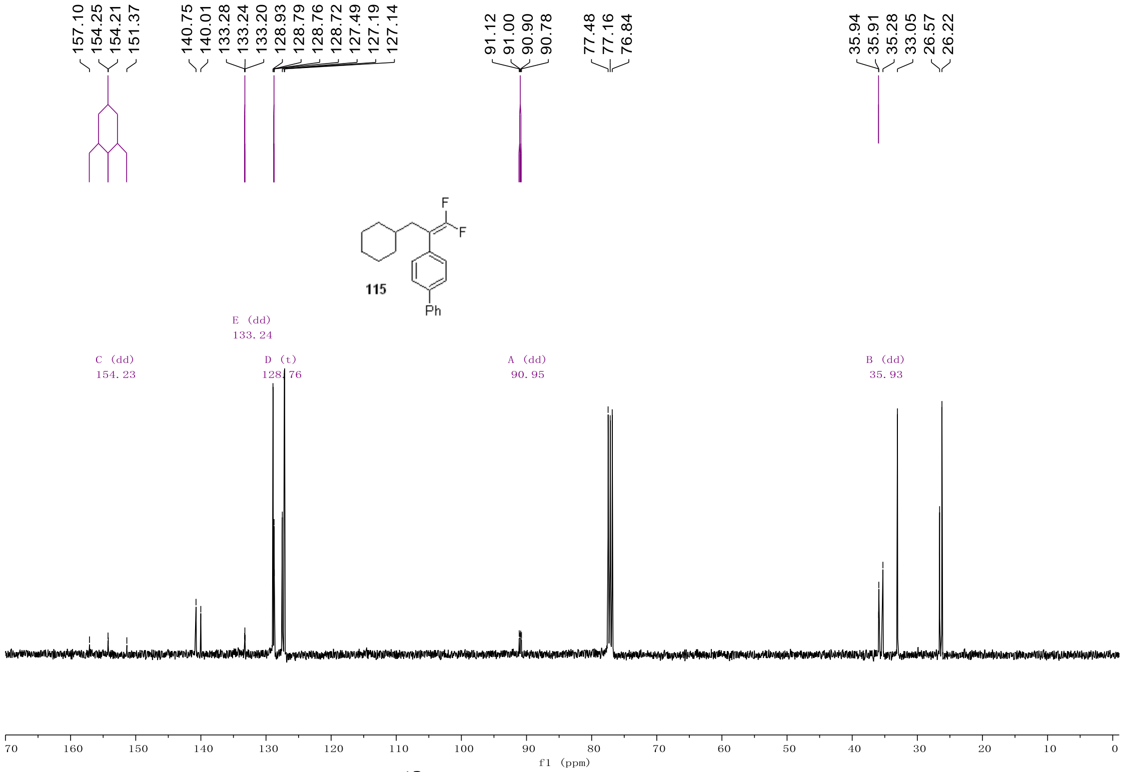

Supplementary Figure 284. ^{13}C NMR Spectra of compound **115**.

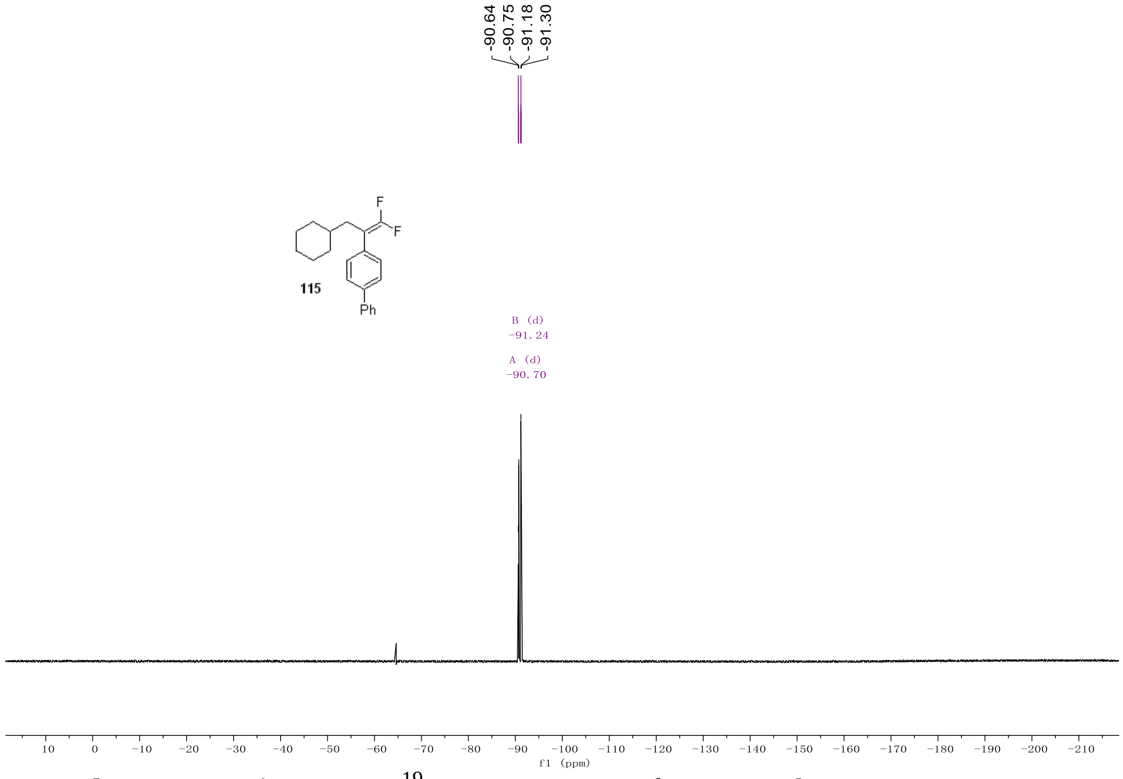

Supplementary Figure 285. ^{19}F NMR Spectra of compound **115**.

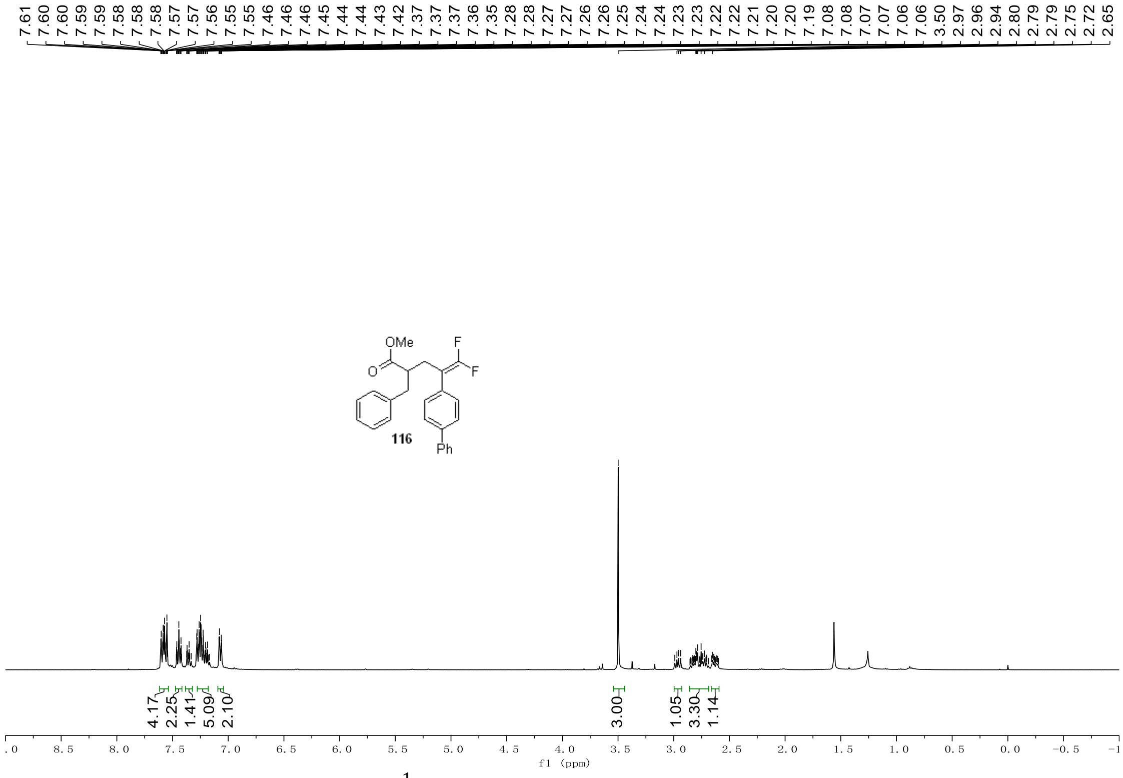

Supplementary Figure 286. ^{1}H NMR Spectra of compound **116**.

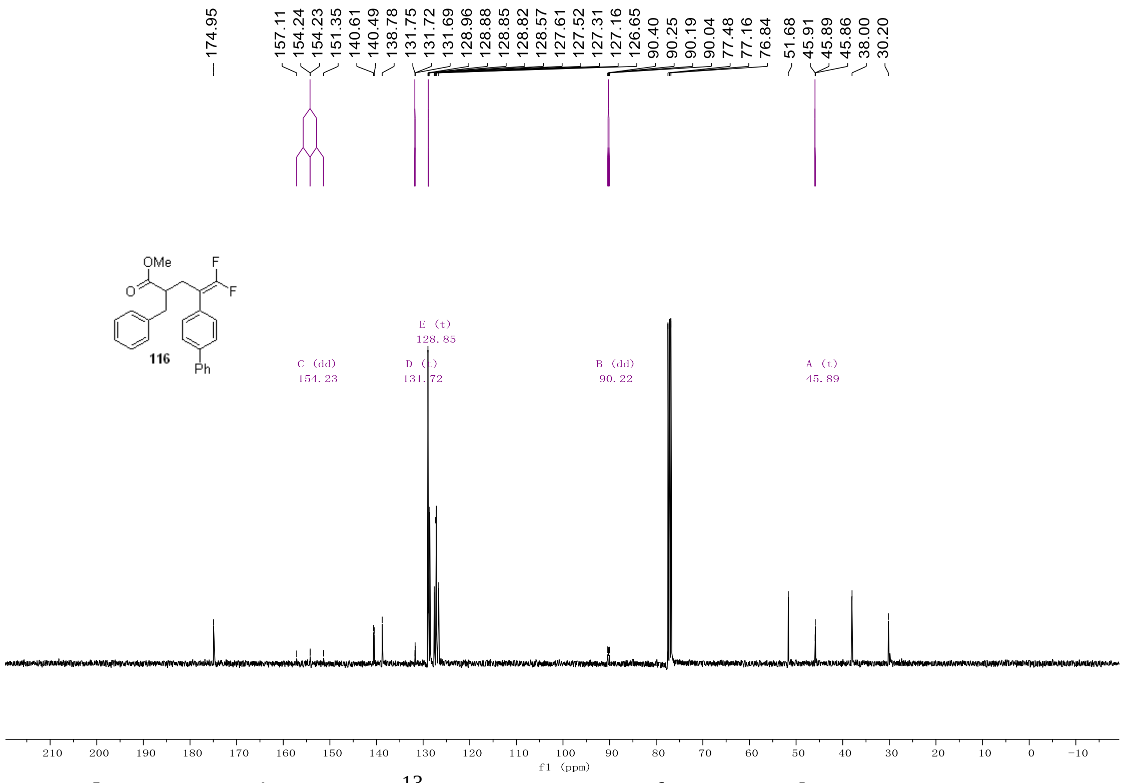

Supplementary Figure 287. ^{13}C NMR Spectra of compound **116**.

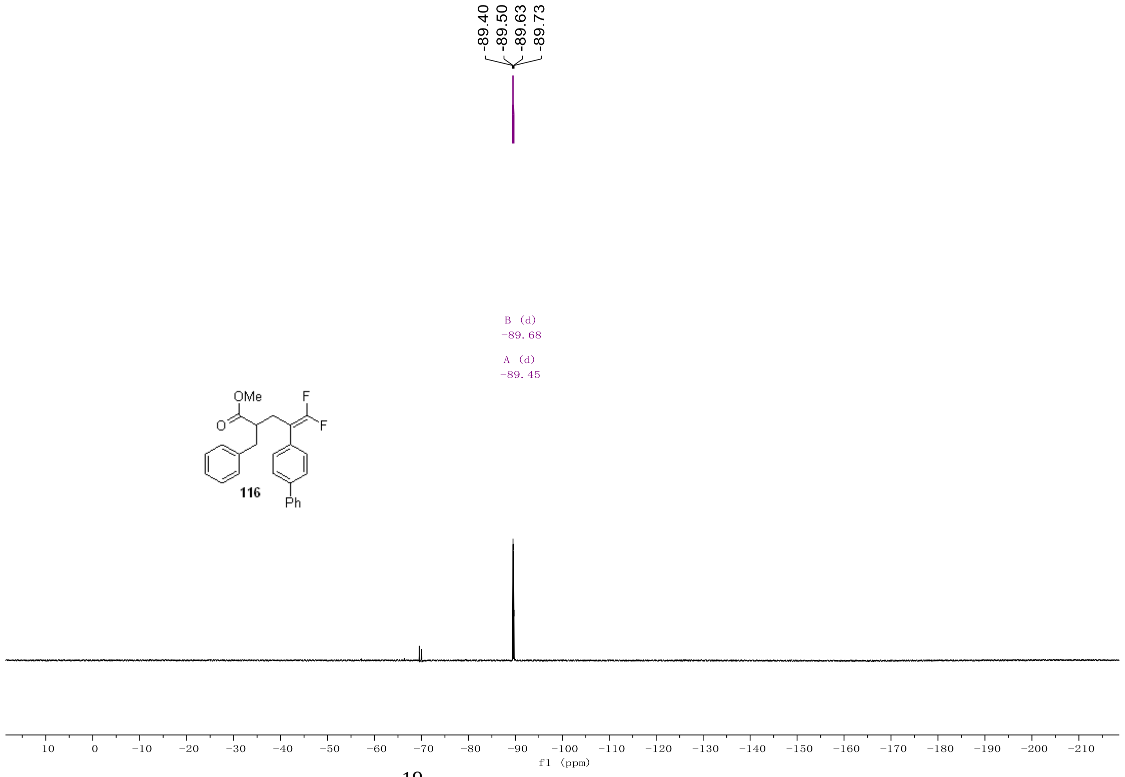

Supplementary Figure 288. ^{19}F NMR Spectra of compound **116**.

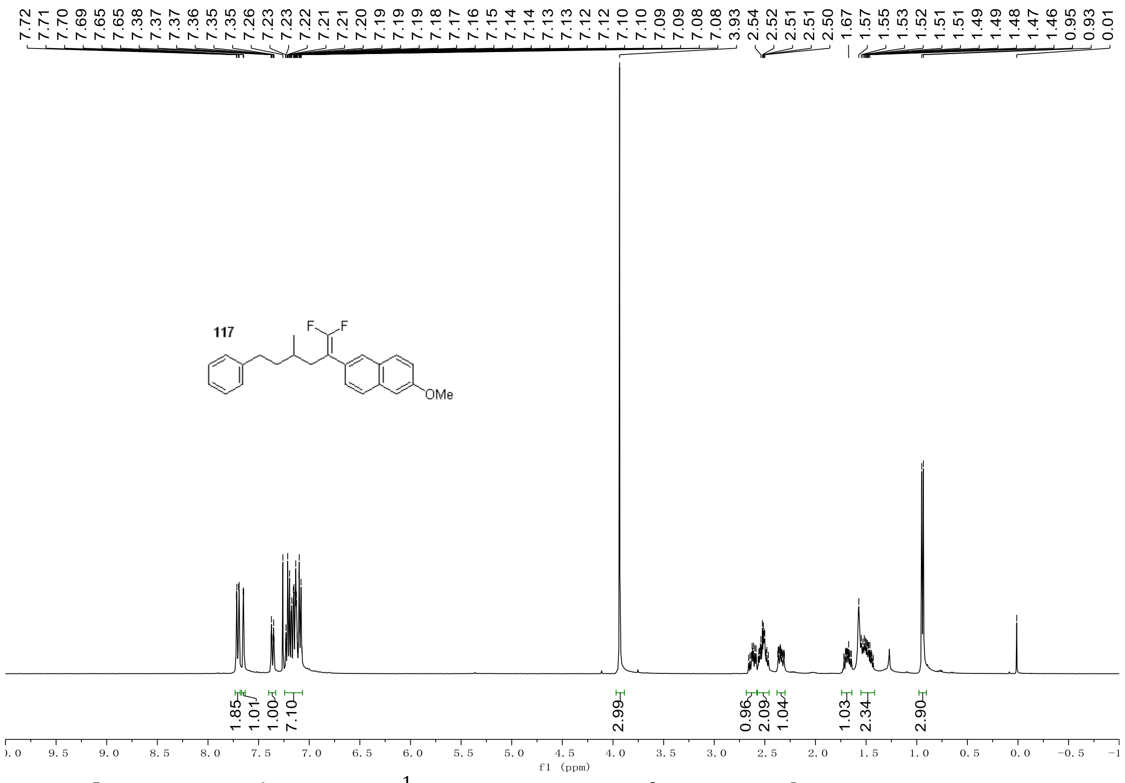

Supplementary Figure 289. ^{1}H NMR Spectra of compound **117**.

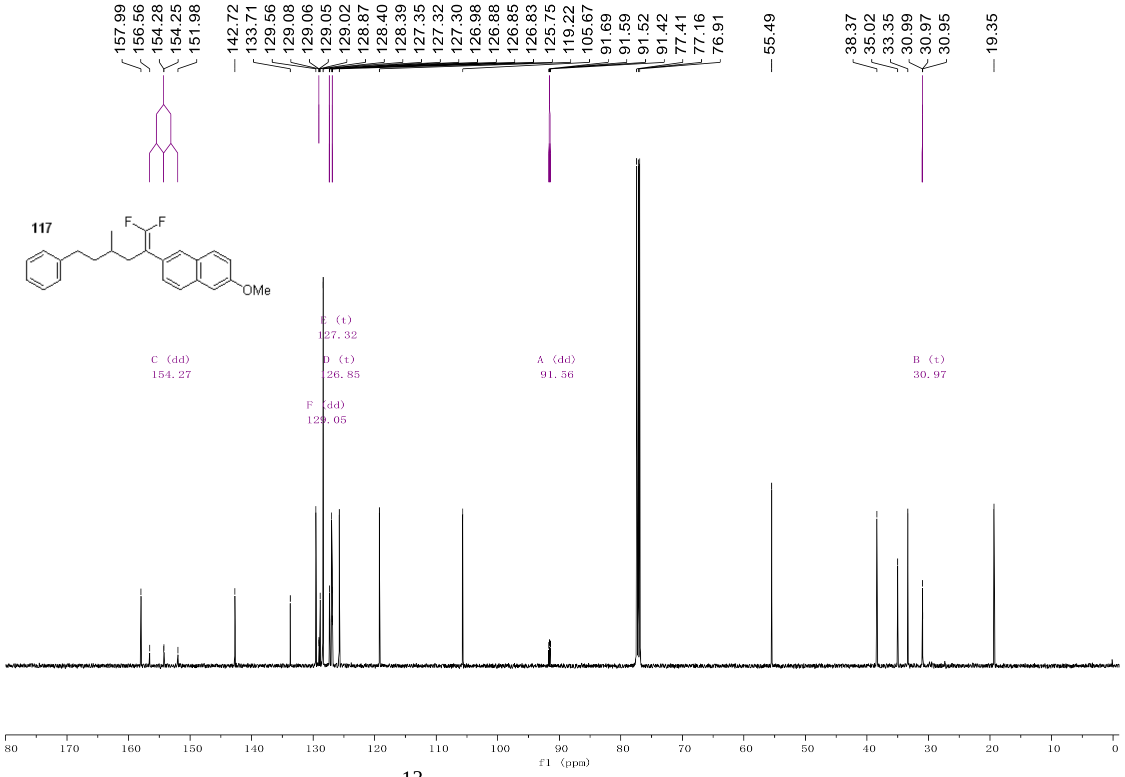

Supplementary Figure 290. ^{13}C NMR Spectra of compound **117**.

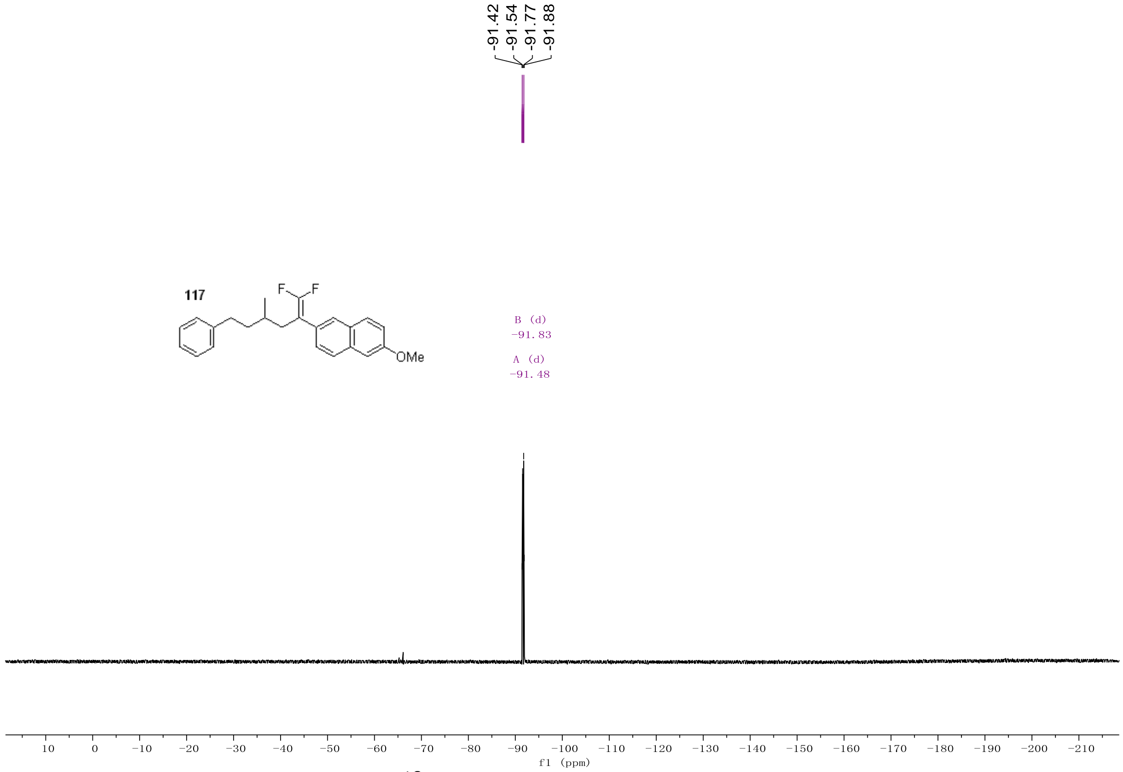

Supplementary Figure 291. ^{19}F NMR Spectra of compound **117**.

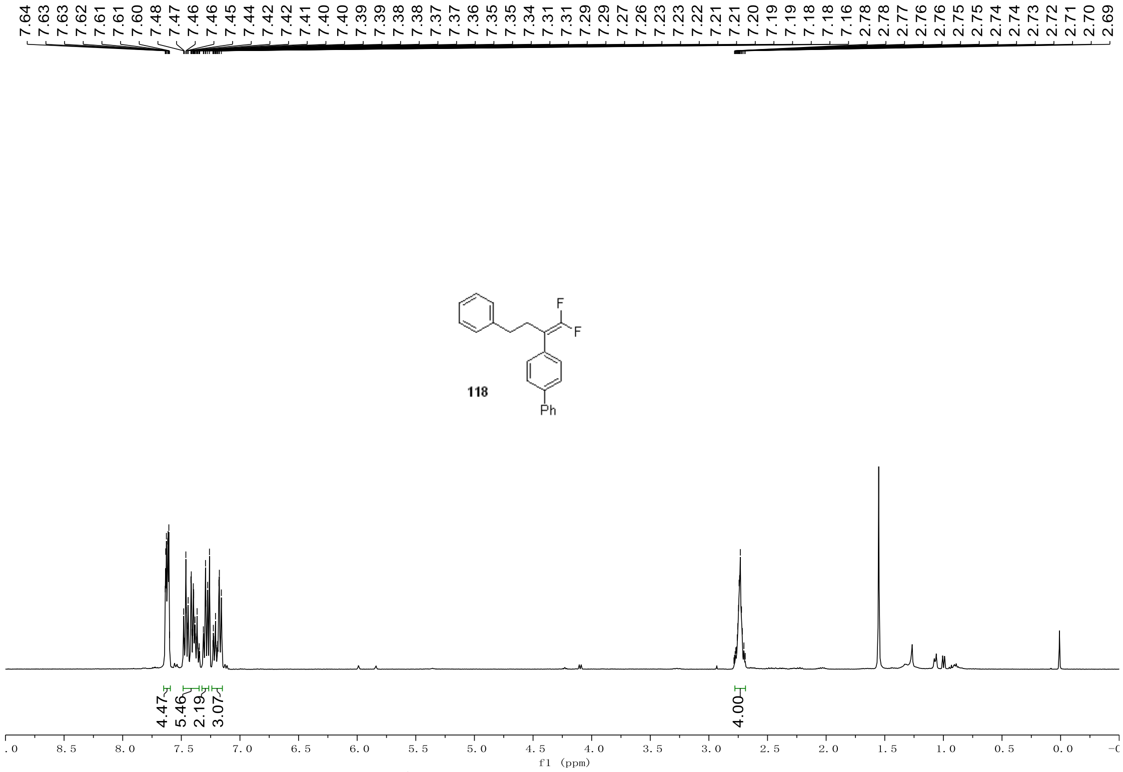

Supplementary Figure 292. ^{1}H NMR Spectra of compound **118**.

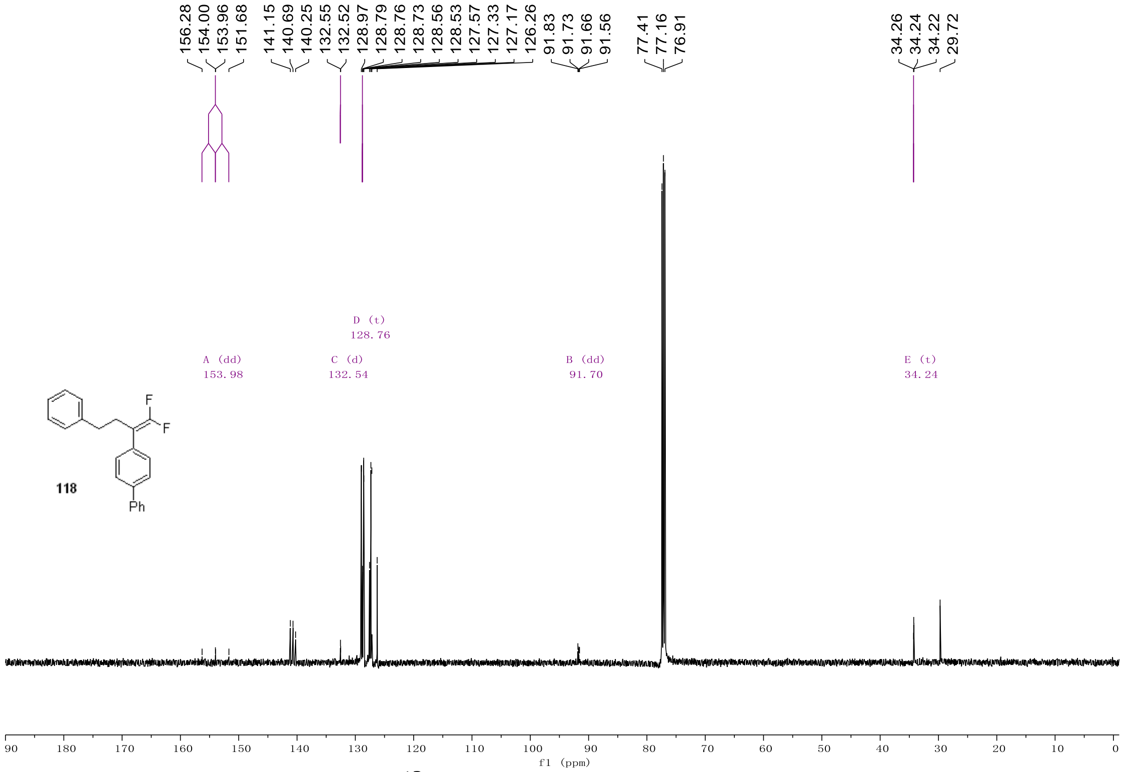

Supplementary Figure 293. ^{13}C NMR Spectra of compound **118**.

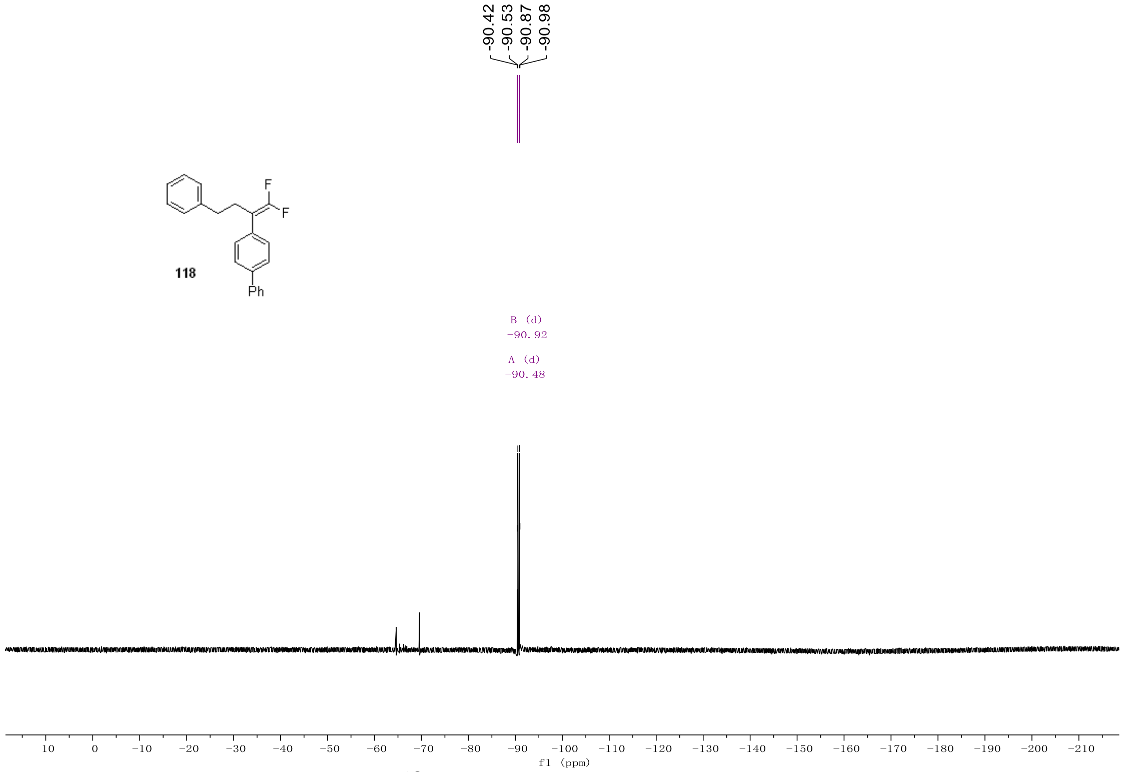

Supplementary Figure 294. ^{19}F NMR Spectra of compound **118**.

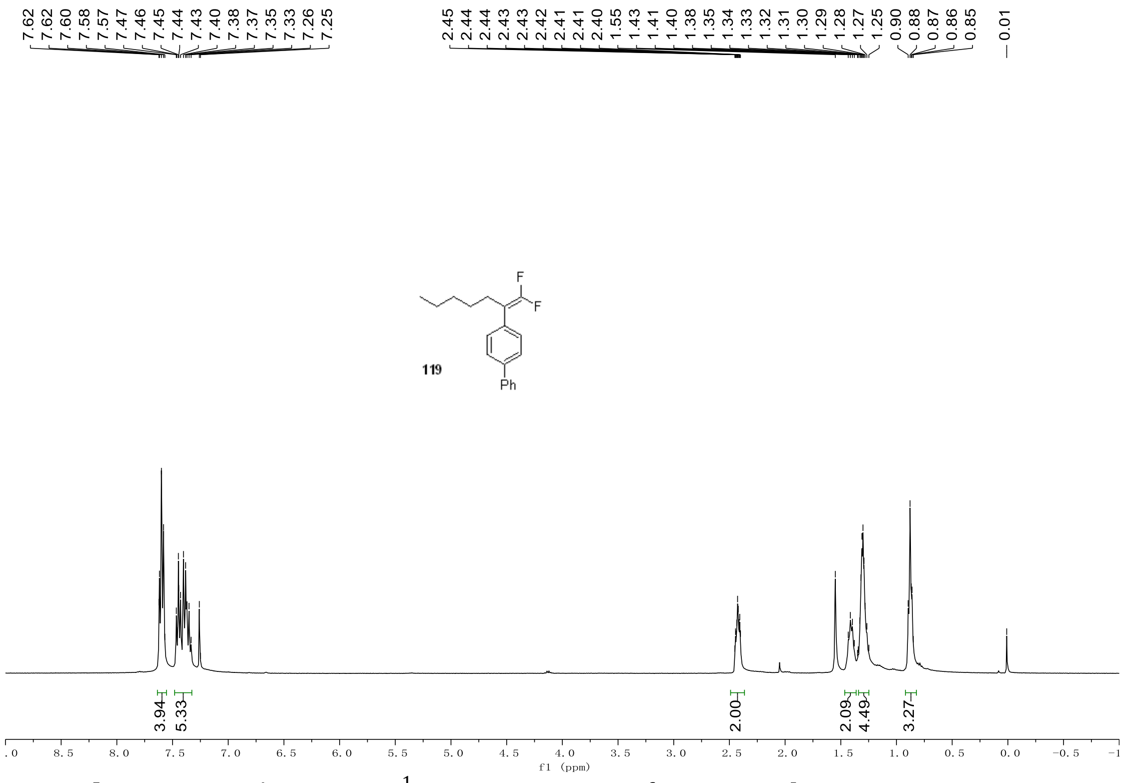

Supplementary Figure 295. ^{1}H NMR Spectra of compound **119**.

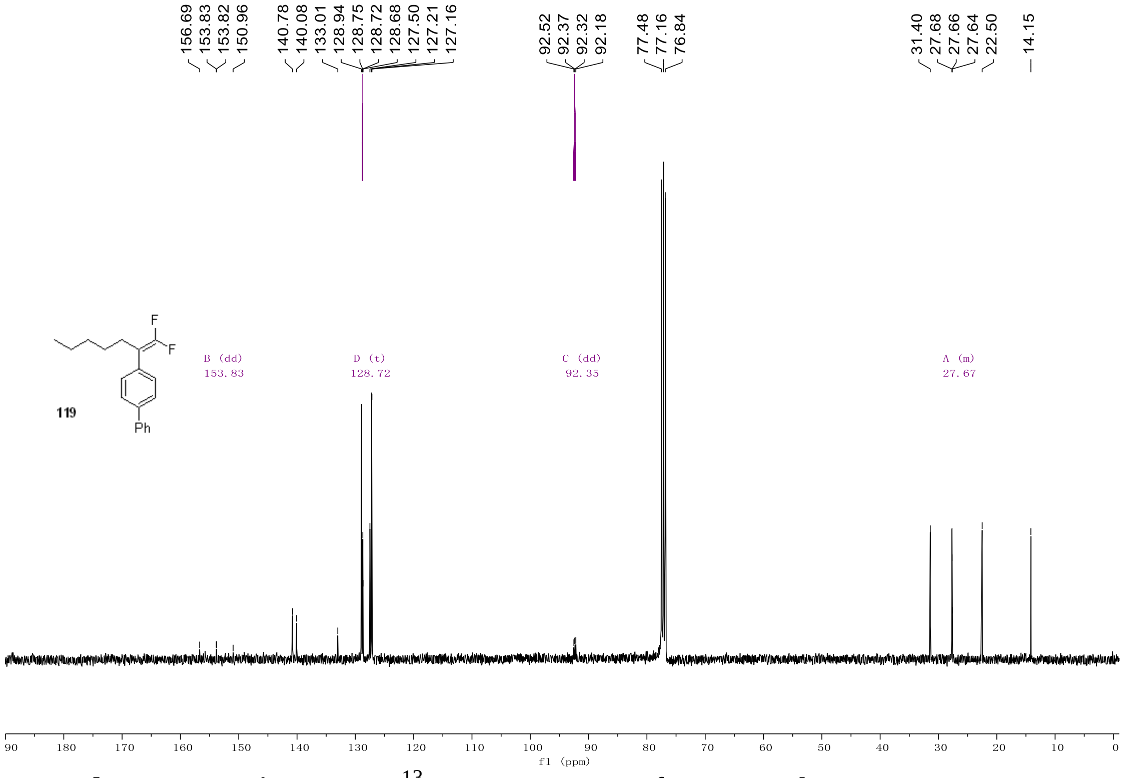

Supplementary Figure 296. ^{13}C NMR Spectra of compound **119**.

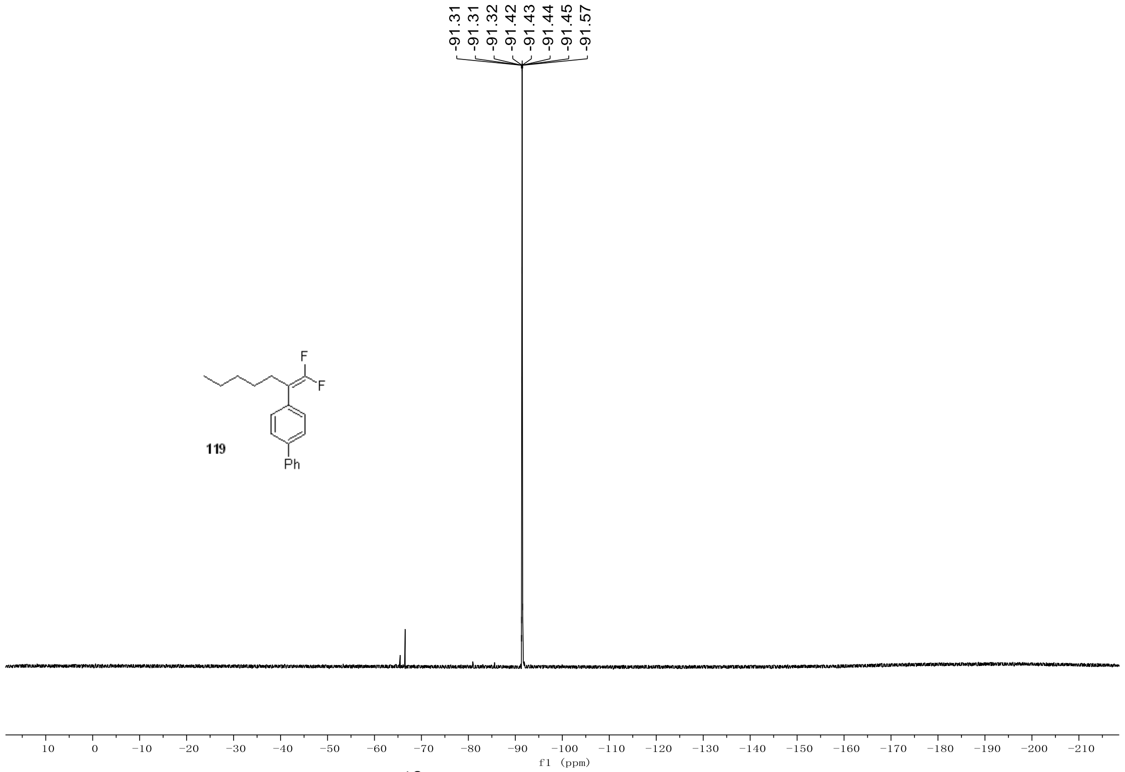

Supplementary Figure 297. ^{19}F NMR Spectra of compound **119**.

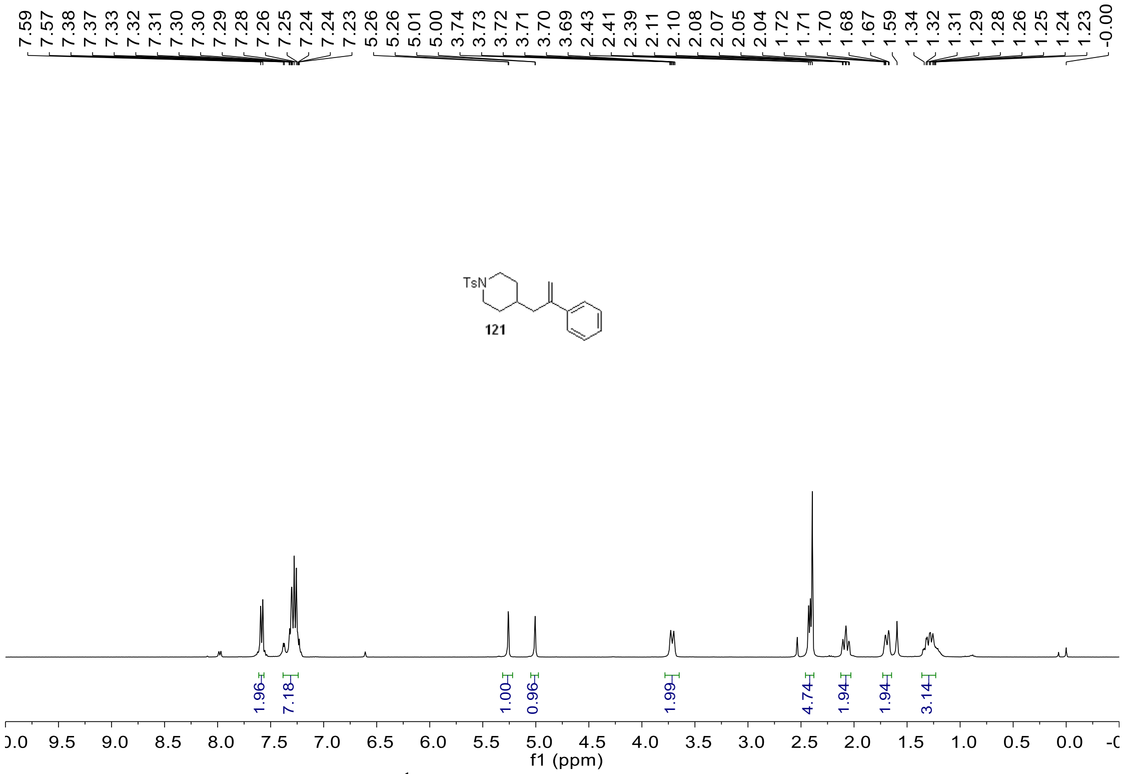

Supplementary Figure 298. ^{1}H NMR Spectra of compound **121**.

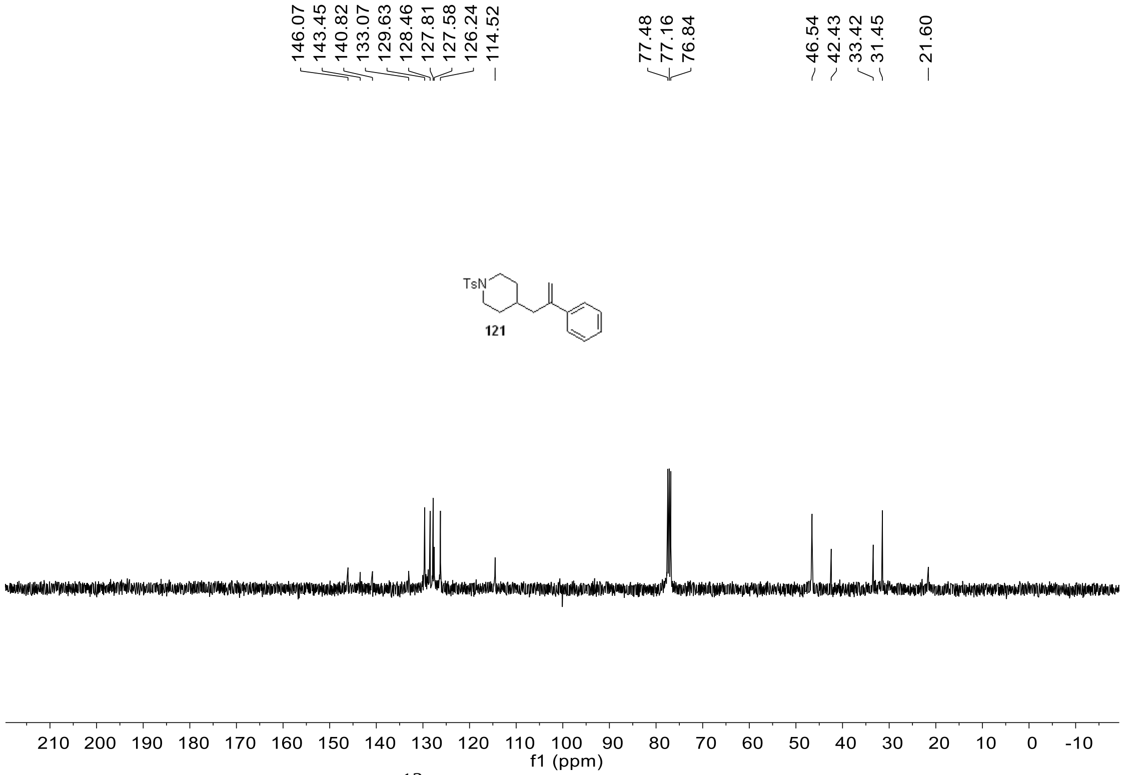

Supplementary Figure 299. ^{13}C NMR Spectra of compound **121**.

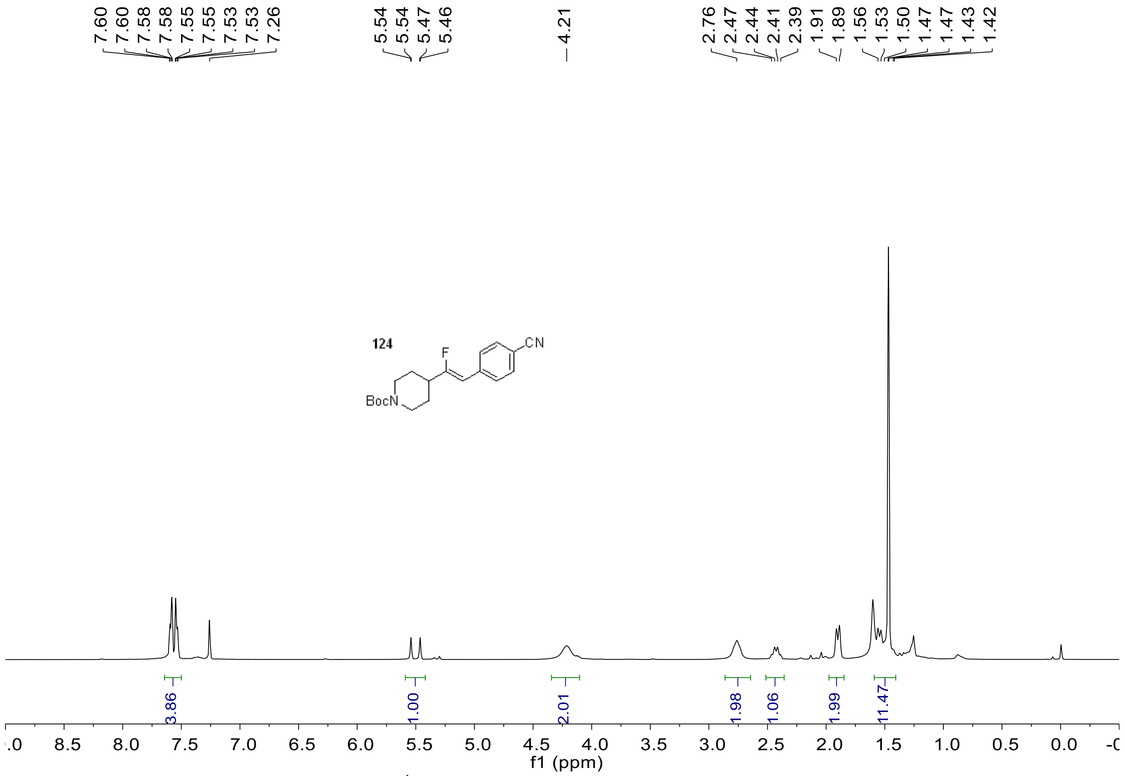

Supplementary Figure 300. ^{1}H NMR Spectra of compound **124**.

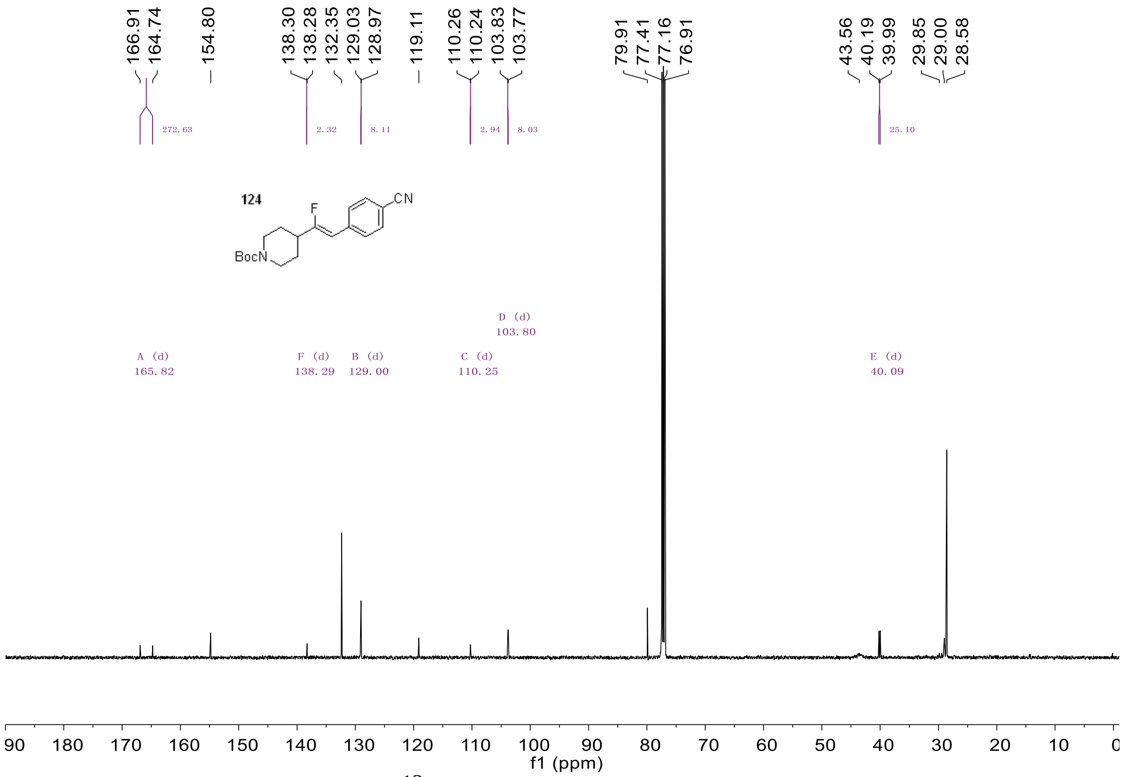

Supplementary Figure 301. ^{13}C NMR Spectra of compound **124**.

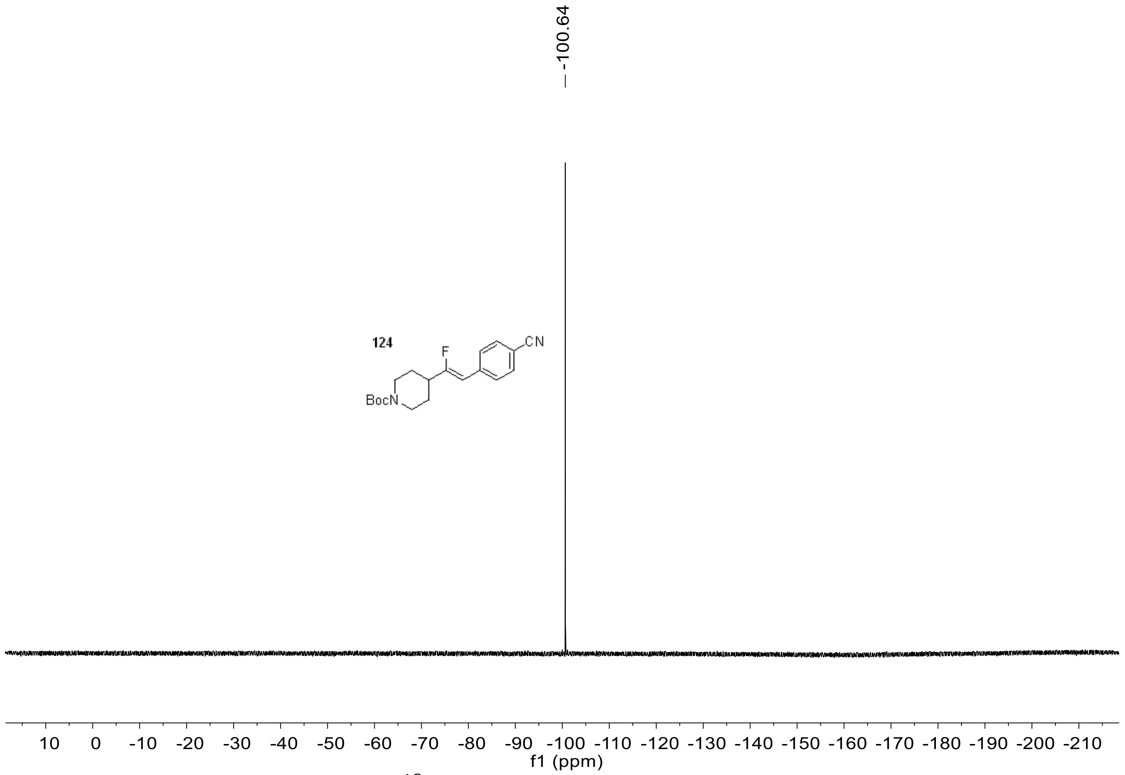

Supplementary Figure 302. ^{19}F NMR Spectra of compound **124**.

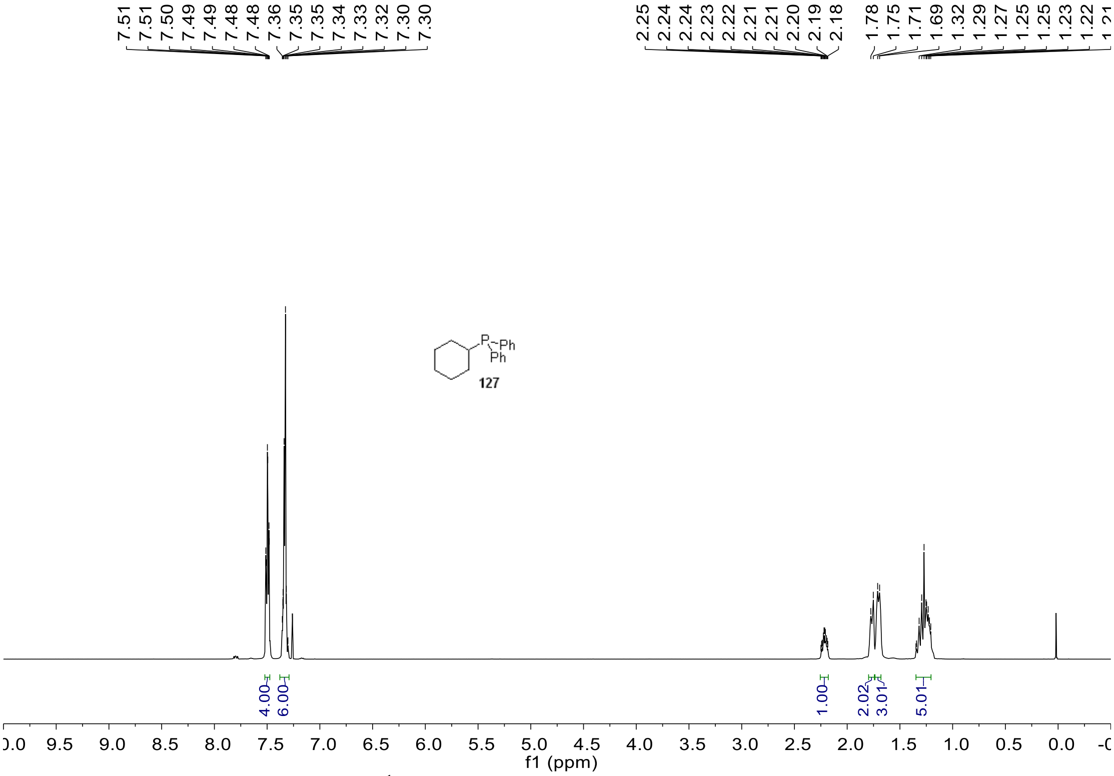

Supplementary Figure 303. ^{1}H NMR Spectra of compound **127**.

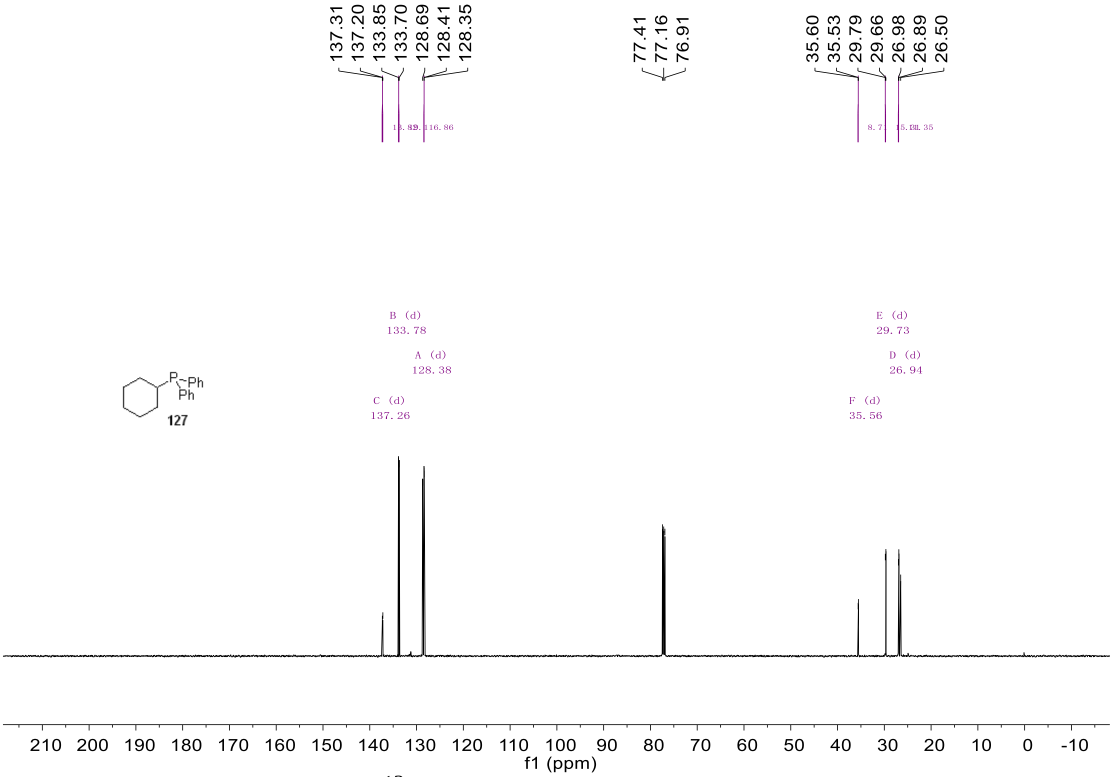

Supplementary Figure 304. ^{13}C NMR Spectra of compound **127**.

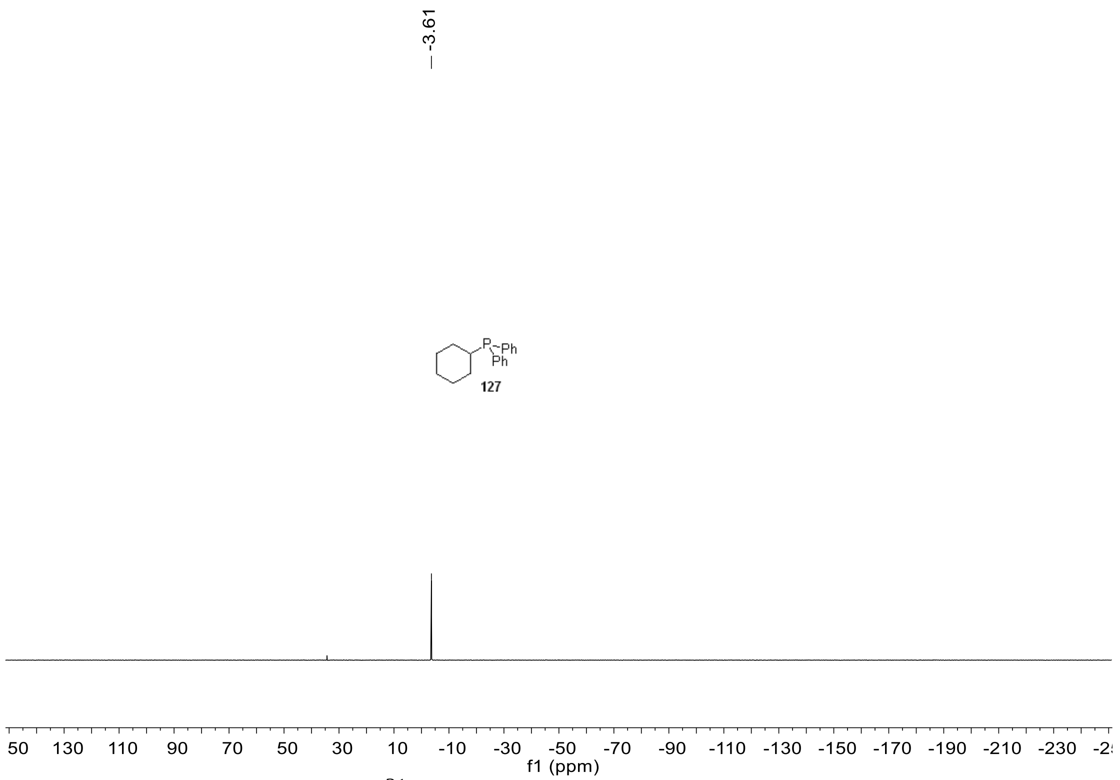

Supplementary Figure 305. ^{31}P NMR Spectra of compound **127**.

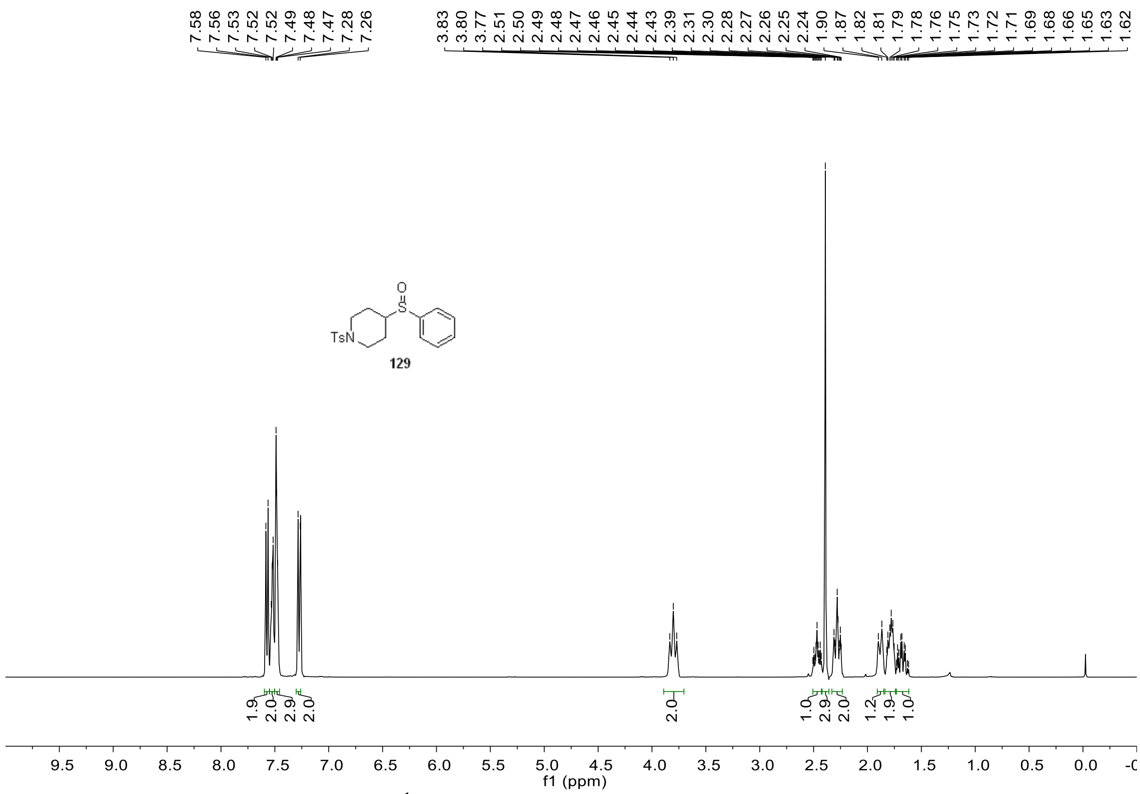

Supplementary Figure 306. ^{1}H NMR Spectra of compound **129**.

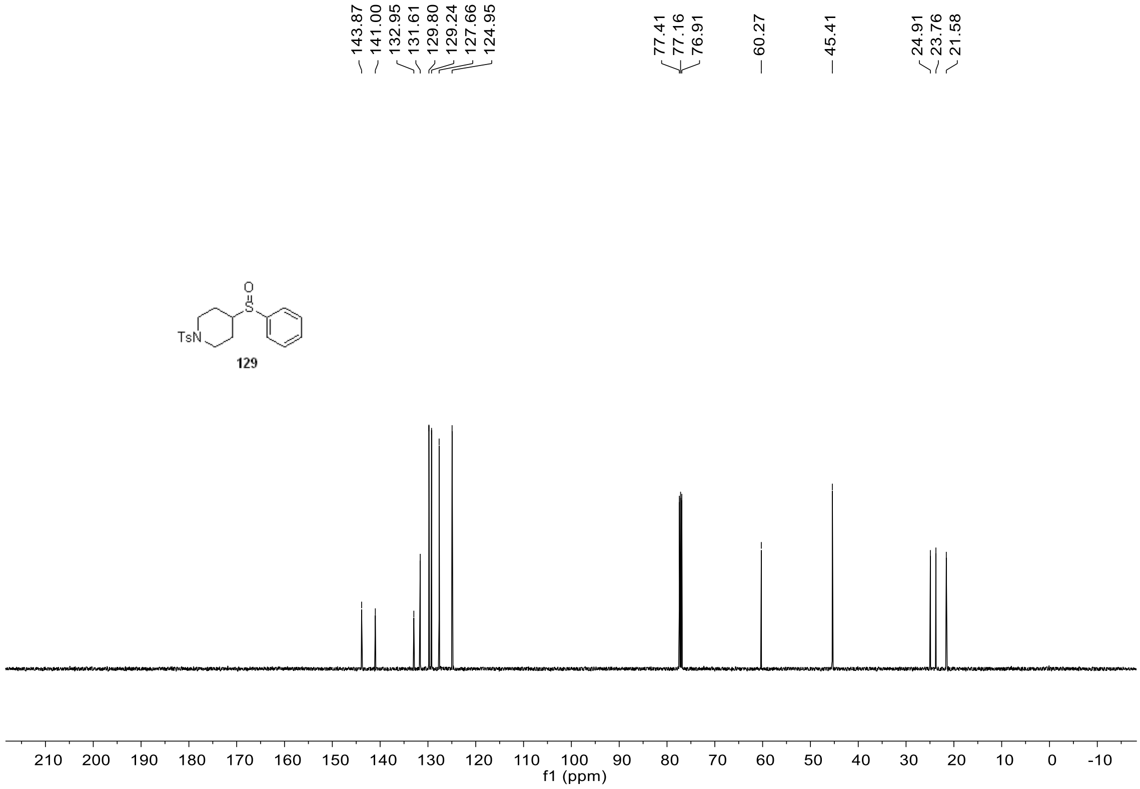

Supplementary Figure 307. ^{13}C NMR Spectra of compound **129**.

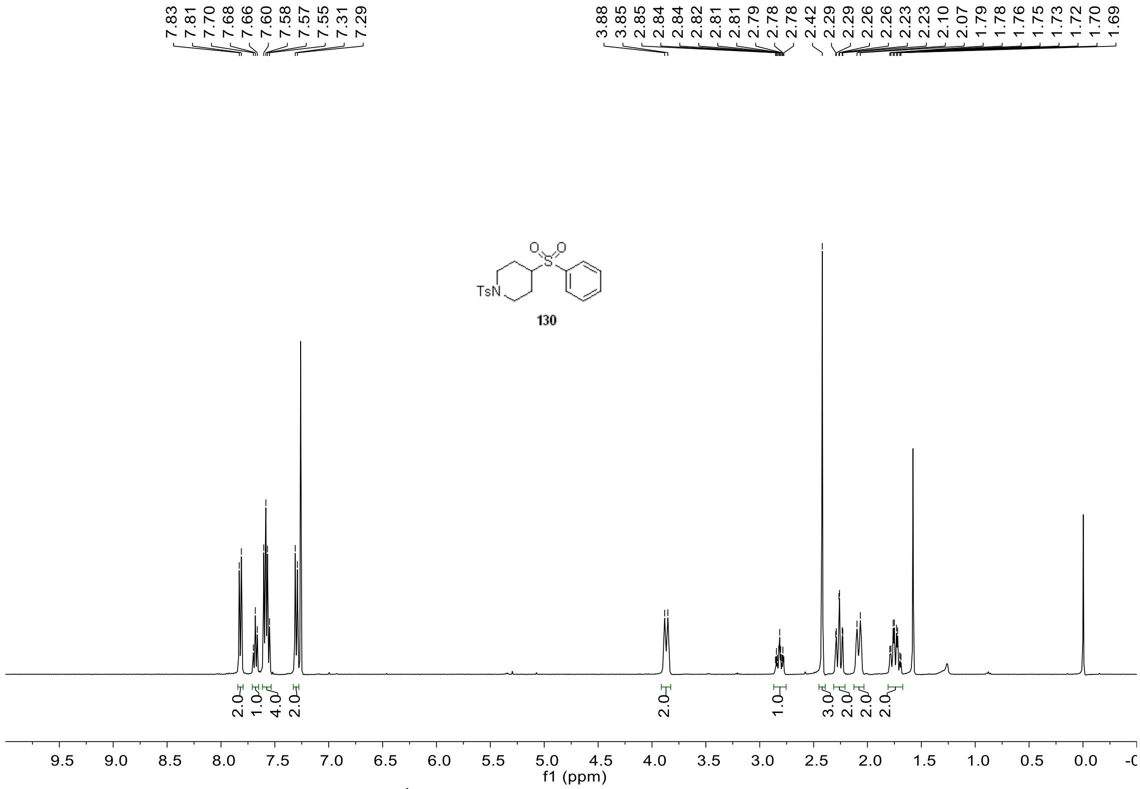

Supplementary Figure 308. ^{1}H NMR Spectra of compound **130**.

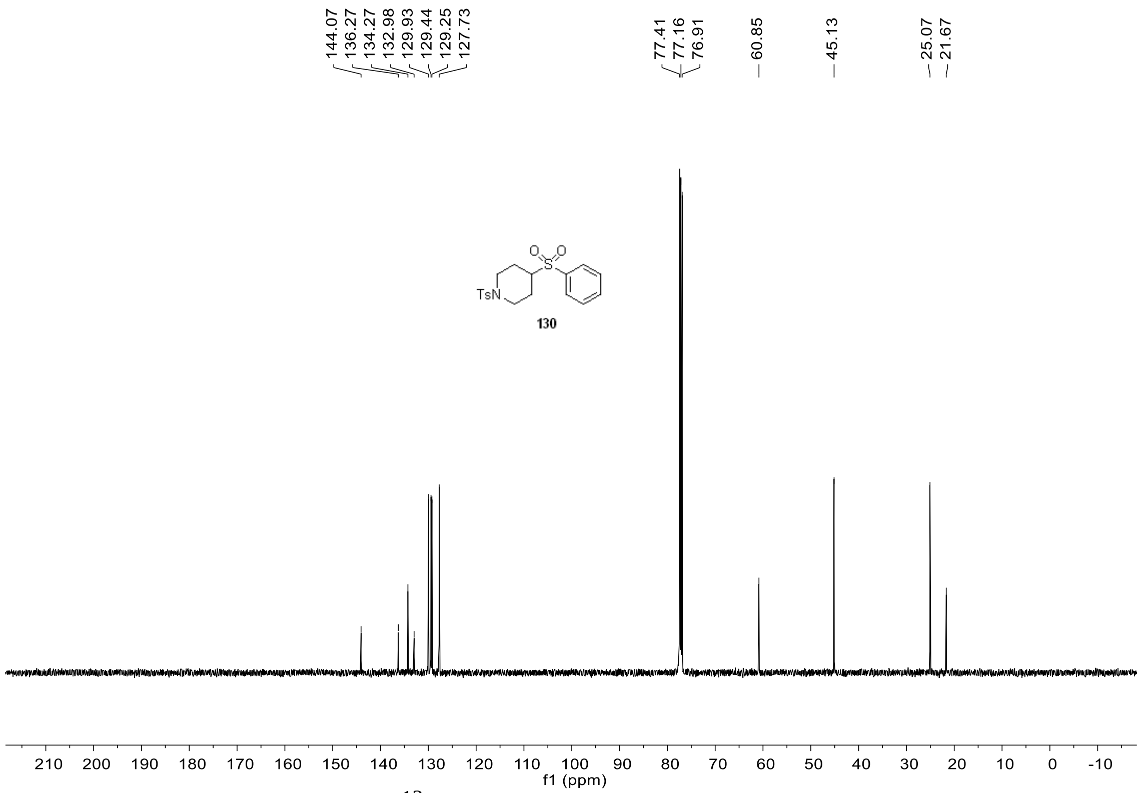

Supplementary Figure 309. ^{13}C NMR Spectra of compound **130**.

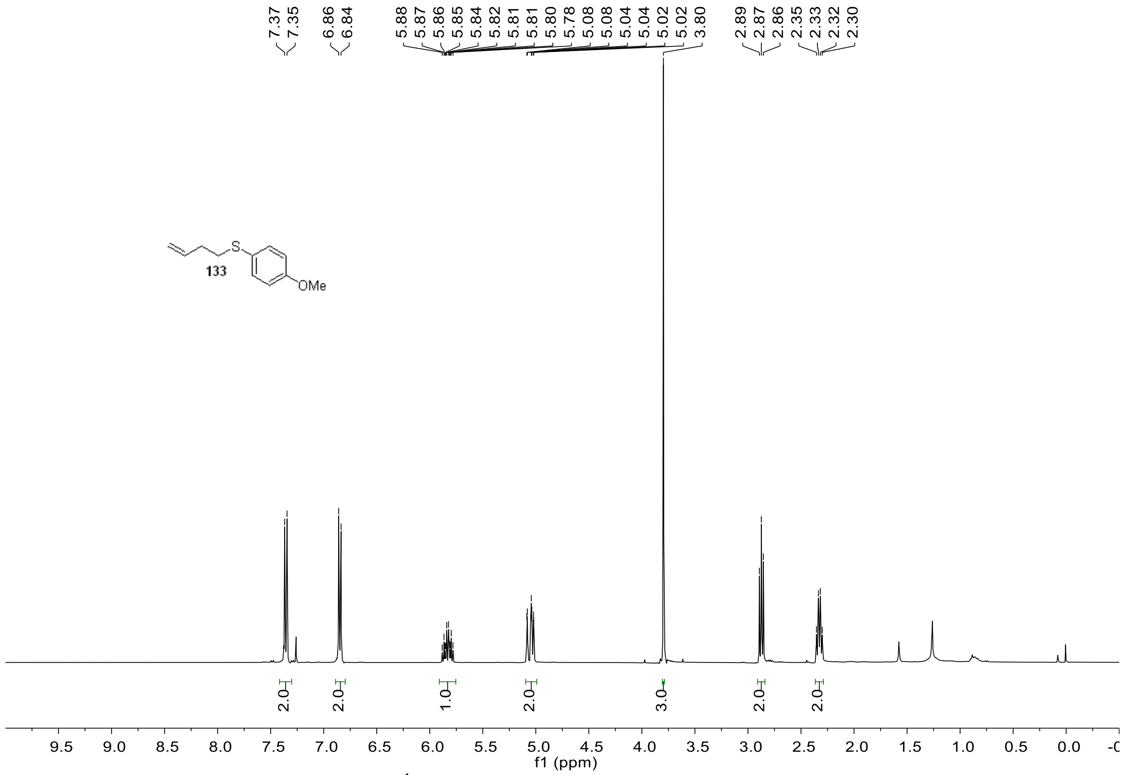

Supplementary Figure 310. ^{1}H NMR Spectra of compound **133**.

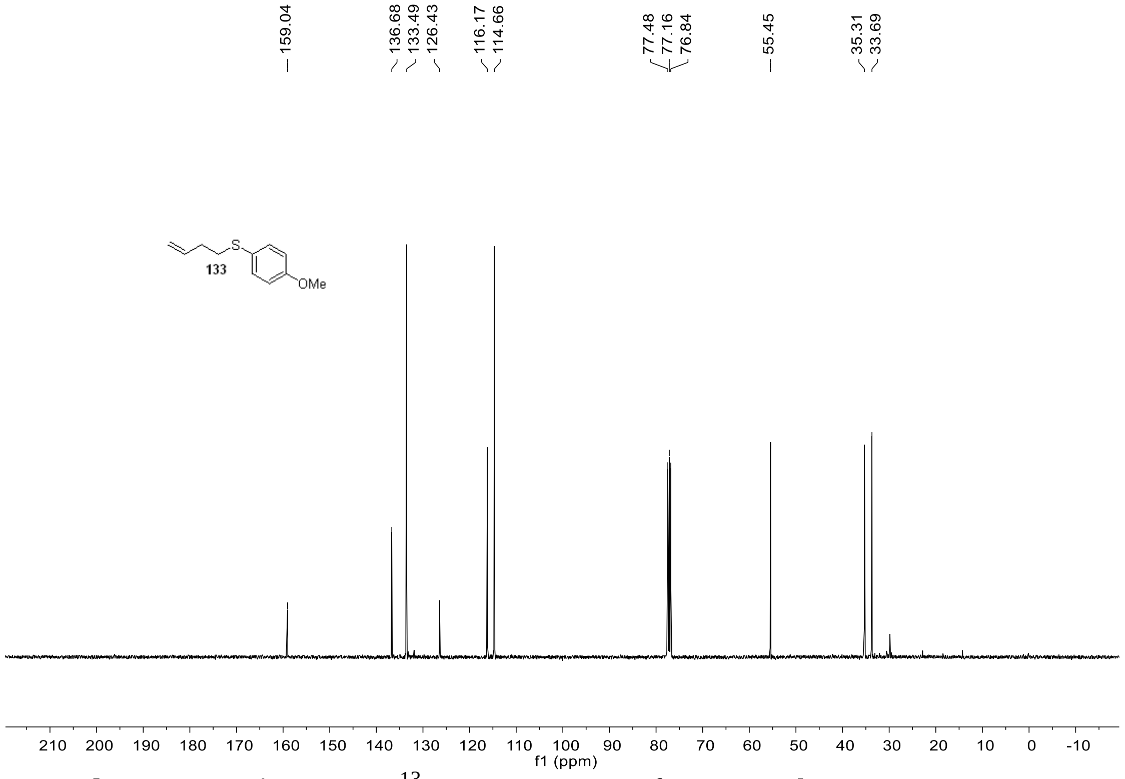

Supplementary Figure 311. ^{13}C NMR Spectra of compound **133**.

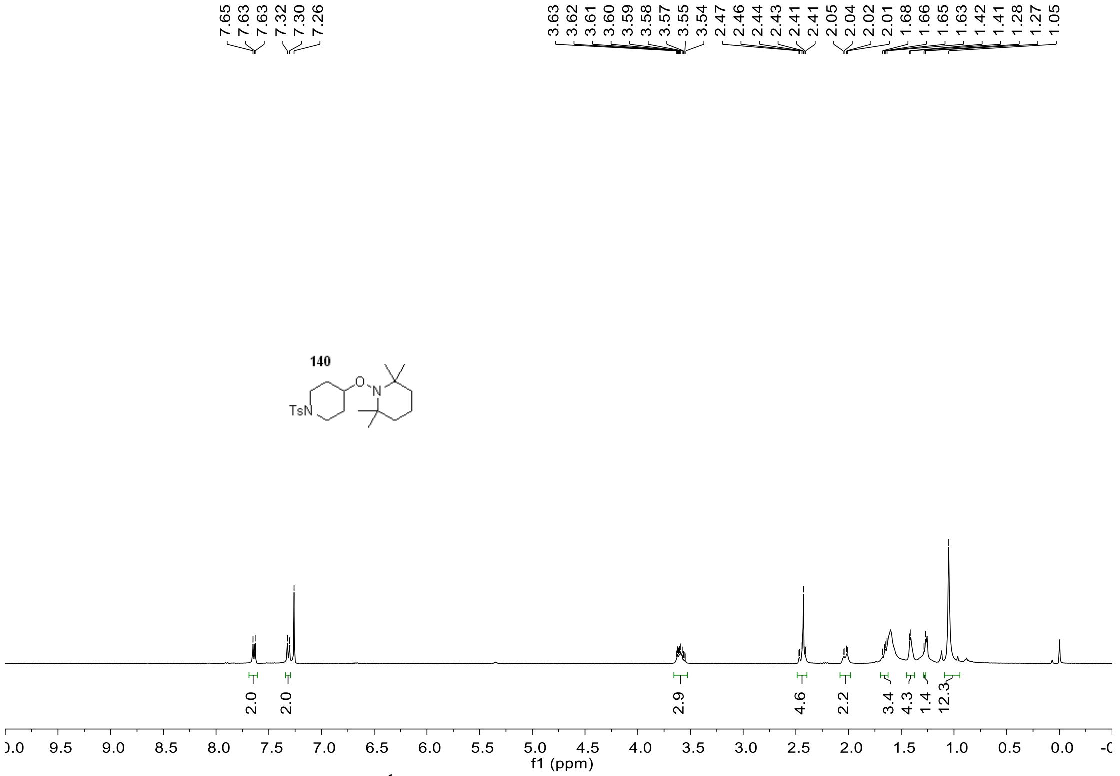

Supplementary Figure 312. ^{1}H NMR Spectra of compound **140**.

Supplementary References

[1] X.-G. Liu, C.-J. Zhou, E. Lin, X.-L. Han, S.-S. Zhang, Q. Li, H. Wang, Decarboxylative Negishi Coupling of Redox‐Active Aliphatic Esters by Cobalt Catalysis. *Angew. Chem. Int. Ed.* **57**, 13096 (2018)

[2] J. Cornella, J. T. Edwards, T. Qin, S. Kawamura, J. Wang, C.-M. Pan, R. Gianatassio, M. Schmidt, M. D. Eastgate, P. S. Baran, Practical Ni-Catalyzed Aryl–Alkyl Cross-Coupling of Secondary Redox-Active Esters. *J. Am. Chem. Soc.* **138**, 2174 (2016)

[3] F. Toriyama, J. Cornella, L. Wimmer, T.-G. Chen, D. D. Dixon, G. Creech, P. S. Baran, Redox-Active Esters in Fe-Catalyzed C–C Coupling. *J. Am. Chem. Soc.* **138**, 11132 (2016)

[4] A. Tlahuext-Aca, L. Candish, R. A. Garza-Sanchez, F. Glorius, Decarboxylative Olefination of Activated Aliphatic Acids Enabled by Dual Organophotoredox/Copper Catalysis. *ACS Catal.* **8**, 1715 (2018)

[5] A. Fawcett, J. Pradeilles, Y. Wang, T. Mutsuga, E. L. Myers, V. K. Aggarwal, Photoinduced decarboxylative borylation of carboxylic acids. *Science* **357**, 283 (2017)

[6] W. Zhao, R. P. Wurz, J. C. Peters, G. C. Fu, Photoinduced, Copper-Catalyzed Decarboxylative C–N Coupling to Generate Protected Amines: An Alternative to the Curtius Rearrangement. *J. Am. Chem. Soc.* **139**, 12153 (2017)

[7] C. Zheng, Y. Wang, Y. Xu, Z. Chen, G. Chen, S. H. Liang, Ru-Photoredox-Catalyzed Decarboxylative Oxygenation of Aliphatic Carboxylic Acids through N-(acyloxy)phthalimide. *Org. Lett*. **20**, 4824 (2018)

[8] Ze-Fan Zhu, Jia-Lin Tu, Feng Liu, Ni-Catalyzed deaminative hydroalkylation of internal alkynes. *Chem. Commun.* **55**, 11478 (2019)

[9] J. Wu, L. He, A. Noble, V. K. Aggarwal, Photoinduced Deaminative Borylation of Alkylamines. *J. Am. Chem. Soc*. **140**, 10700 (2018)

[10] J. Hu, G. Wang, S. Li, Z. Shi, Selective C−N Borylation of Alkyl Amines Promoted by Lewis Base. *Angew. Chem. Int. Ed*. **57**, 15227 (2018)

[11] S. Ni, C.-X. Li, Y, Mao, J. Han, Y. Wang, H. Yan, Y. Pan, Ni-catalyzed

deaminative cross-electrophile coupling of Katritzky salts with halides via C-N bond activation. *Sci. Adv.* **5**, eaaw9516 (2019)

[12] M. Li and J. “Joelle” Wang, Cobalt-Catalyzed Direct C–H Thiolation of Aromatic Amides with Disulfides: Application to the Synthesis of Quetiapine. *Org. Lett*. **20**, 6490 (2018)

[13] P. Salehi , A. Farrokhi M. Gholizadeh, OXIDATIVE COUPLING OF THIOLS BY PYRIDINIUM CHLOROCHROMATE IN SOLUTION AND SOLVENT FREE CONDITIONS. *Synthetic Commun*. **31**, 2777 (2001)

[14] P. Mampuys, Y. Zhu, S. Sergeyev, E. Ruijter, R. V. A. Orru, S. Van Doorslaer, B. U. W. Maes, Iodide-Catalyzed Synthesis of Secondary Thiocarbamates from Isocyanides and Thiosulfonates. *Org. Lett.* **18**, 2808 (2016)

[15] Y. Fang, T. Rogge, L. Ackermann, S.-Y. Wang, S.-J. Ji, Nickel-catalyzed reductive thiolation and selenylation of unactivated alkyl bromides. *Nat. Commun*. **9**, 2240 (2018)

[16] A. Sadhukhan, M. C. Hobbs, A. J. H. M. Meijer, I. Coldham, Highly enantioselective metallation–substitution alpha to a chiral nitrile. *Chem. Sci*. **8**, 1436 (2017)

[17] S. Huang, N. Thirupathi, C.-H. Tung, Z. Xu, Copper-Catalyzed Oxidative Trifunctionalization of Olefins: An Access to Functionalized β-Keto Thiosulfones. *J. Org. Chem*. **83**, 9449 (2018)

[18] W. Wang, Y. Lin, Y. Ma, C.-H. Tung, Z. Xu, Copper(I)-Catalyzed Three-Component Click/Persulfuration Cascade: Regioselective Synthesis of Triazole Disulfides. *Org. Lett*. **20**, 2956 (2018)

[19] D. P. Gamblin, P. Garnier, S. J. Ward, N. J. Oldham, A. J. Fairbanks, B. G. Davis, Glycosyl phenylthiosulfonates (Glyco-PTS): novel reagents for glycoprotein synthesis. *Org. Biomol. Chem.* **1**, 3642 (2003)

[20] A. H. Cherney, S. E. Reisman, Nickel-Catalyzed Asymmetric Reductive Cross-Coupling Between Vinyl and Benzyl Electrophiles. *J. Am. Chem. Soc.* **136**, 14365 (2014)

[21] Müller, D.; Alexakis, A. Copper-Catalyzed Asymmetric 1,4-Addition of Alkenyl Alanes to N-Substituted-2-3-dehydro-4-piperidones. *Org. Lett.* **14**, 1842 (2012)

[22] D. Müller, A. Alexakis, Formation of Quaternary Stereogenic Centers by Copper‐Catalyzed Asymmetric Conjugate Addition Reactions of Alkenylaluminums to Trisubstituted Enones. *Chem. Eur. J.* **19**, 15226 (2013)

[23] Y. Liu, Y. Zhou, Y. Zhao, J. Qu, Synthesis of gem-Difluoroallylboronates via FeCl2-Catalyzed Boration/β-Fluorine Elimination of Trifluoromethyl Alkenes. *Org. Lett*. **19**, 946 (2017)

[24] X. Ji, Y. Liu, H. Shi, S. Cao, Direct $C(sp^3)$−H difluoroallylation of diarylmethanes with α-(trifluoromethyl) styrenes at room temperature. *Tetrahedron* **74**, 4155 (2018)

[25] Y. Lan, F. Yang, C. Wang, Synthesis of gem-Difluoroalkenes via Nickel-Catalyzed Allylic Defluorinative Reductive Cross-Coupling. *ACS Catal*. **8**, 9245 (2018)

[26] B. M. Trost, L. Debien, Palladium-Catalyzed Trimethylenemethane Cycloaddition of Olefins Activated by the σ-Electron-Withdrawing Trifluoromethyl Group. *J. Am. Chem. Soc*. **137**, 11606 (2015)

[27] J.-J. Zhang, J.-C. Yang, L.-N. Guo, X.-H. Duan, Visible-Light-Mediated Dual Decarboxylative Coupling of Redox-Active Esters with α, β-Unsaturated Carboxylic Acids. *Chem. Eur. J*. **23**, 10259 (2017)
